# Enantioselective Synthesis of 2,3-Disubstituted Azetidines via Copper-Catalyzed Boryl Allylation of Azetines

**DOI:** 10.1021/jacs.5c07821

**Published:** 2025-06-24

**Authors:** Minghui Zhu, Jianwei Sun

**Affiliations:** Department of Chemistry and the Hong Kong Branch of Chinese National Engineering Research Centre for Tissue Restoration & Reconstruction, 58207 The Hong Kong University of Science and Technology, Clear Water Bay, Kowloon, Hong Kong SAR 999077, China

## Abstract

Disclosed here is a highly enantioselective difunctionalization of azetines for convenient access to chiral 2,3-disubstituted azetidines, a family of important scaffolds previously lacking general access. With Cu/bisphosphine as a catalyst, two versatile functionalities (boryl and allyl) were installed on azetine with concomitant construction of two new stereogenic centers. This represents a rare demonstration of Cu-catalyzed asymmetric boryl alkylation of electron-rich olefins and C=C bonds in strained heterocycles. The use of allyl phosphates proved critical not only to overcome the low reactivity of the borylated alkylcuprate intermediate toward alkylation but also to avoid competing side reactions. Remarkably, in almost all cases, single isomers were obtained with complete regio-, enantio-, and diastereoselectivies on the azetidine motif as well as excellent control on the double bond configuration. The mild conditions exhibited outstanding functional group compatibility and chemoselectivity. The versatile boryl and allyl functionalities allowed for easy transformations of the products to other useful chiral azetidines previously lacking straightforward access. Control experiments and kinetic studies indicated that the reaction proceeds by a fast boryl cupration of azetine followed by rate-determining allylation via an intrinsically controlled S_N_2′ pathway.

## Graphical abstract

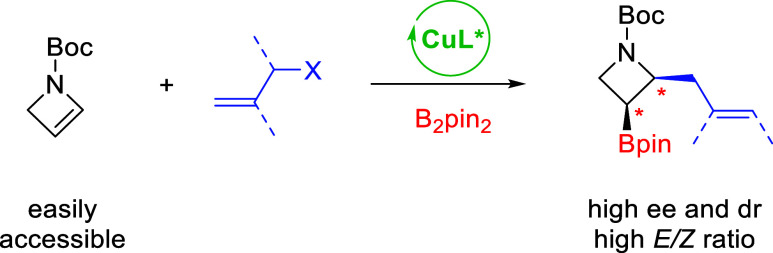

## Introduction

Saturated nitrogen heterocycles represent a family of the most prevalent scaffolds in biologically active compounds.^1^ Specifically, azetidine is a uniquely privileged unit present in numerous drug candidates and natural molecules.^2,3^ For example, it serves as a key pharmacophore of molecules with diverse biological activities (Scheme 1A).^2,3^ The incorporation of this strained heterocycle is beneficial to its pharmacokinetic properties. Moreover, chiral azetidines have also served as useful chiral ligands or building blocks in asymmetric synthesis.^4^

**Scheme 1:**
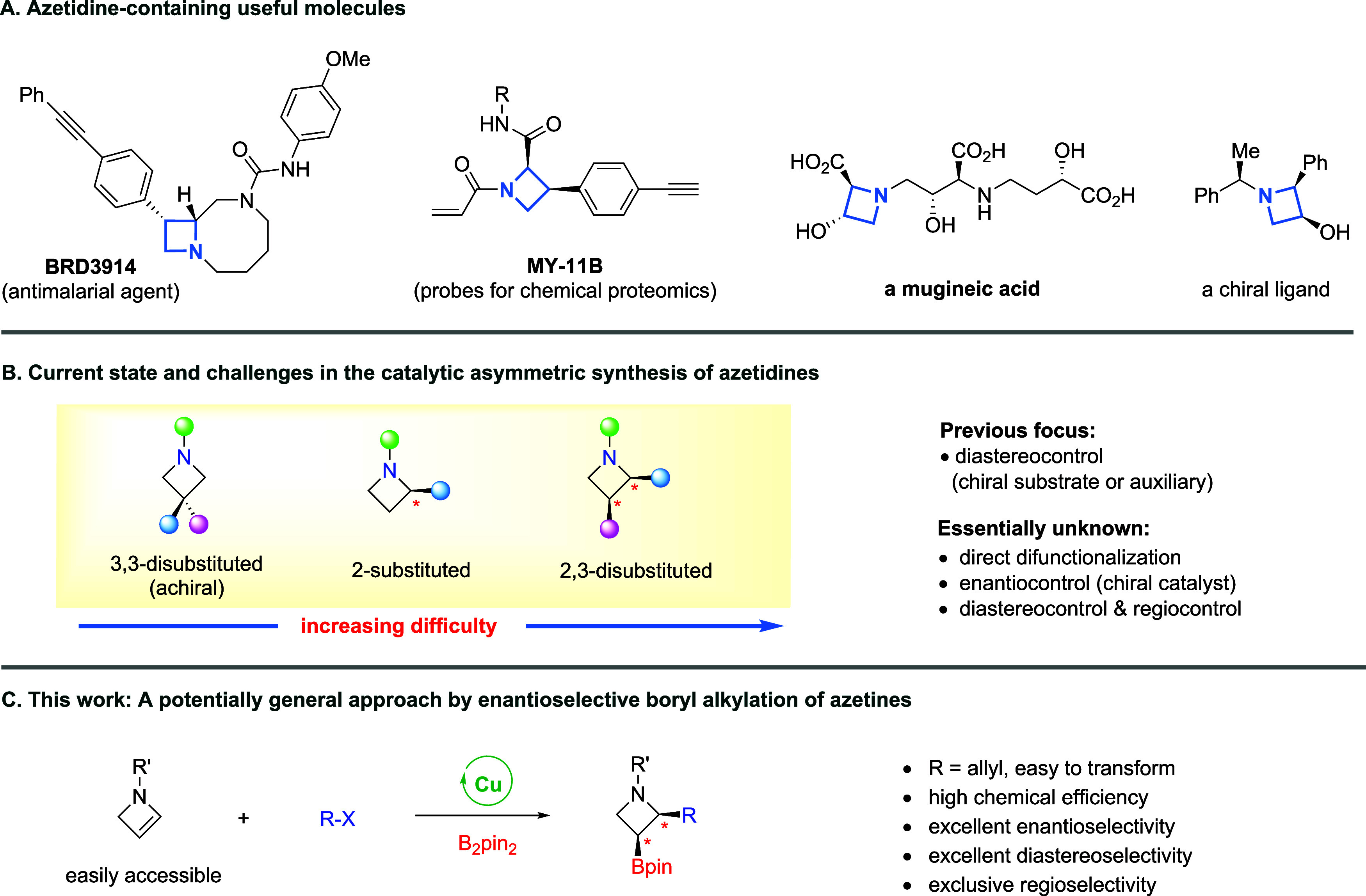
(A–C) Introduction to Chiral Azetidines and Reaction Design

**Table 1:**
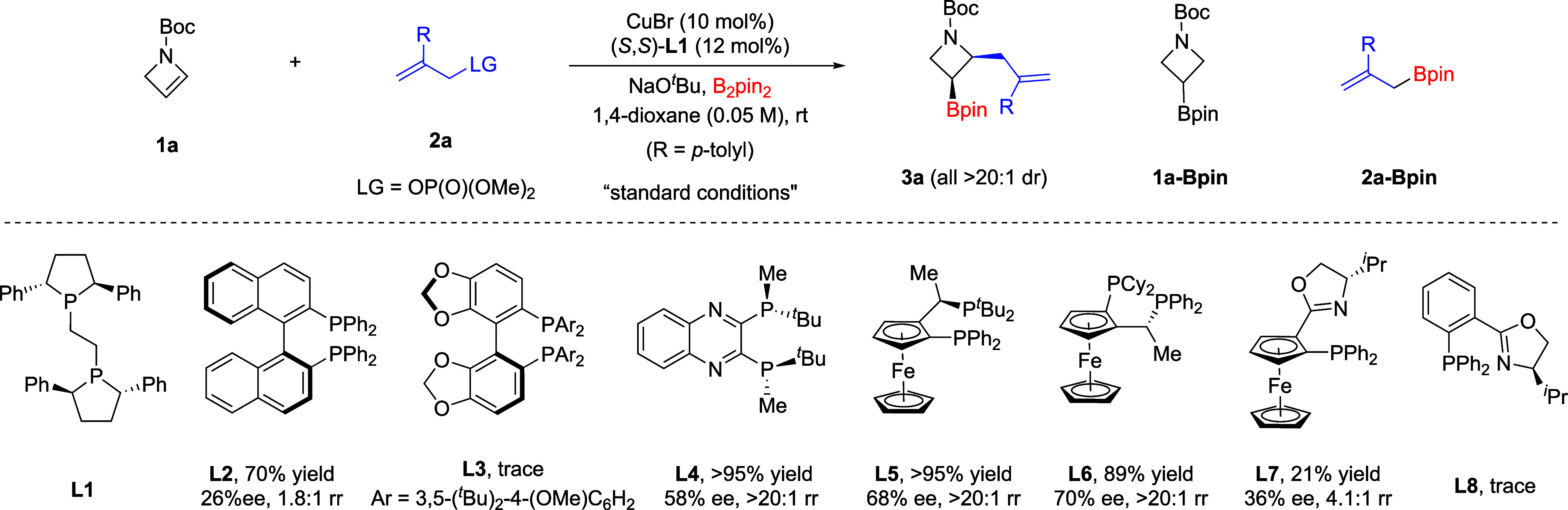
Evaluation of Conditions^a^

entry	deviation from the “standard conditions”	yield (%)	ee (%)	rr
1	LG = Br, OAc, and OBoc	trace		
2	LG = OP(O)(OPh)_2_	92	98	>20:1
3	no change	>95	>99	>20:1
4	**L2**–**L8**	for details, see structures		
5	MTBE as a solvent	80	>99	18:1
6	THF as a solvent	79	>99	>20:1
7	toluene as a solvent	77	>99	>20:1
8	DCM as a solvent	85	>99	>20:1
9	CuCl instead of CuBr	73	>99	19:1
10	Cu(CH_3_CN)_4_BF_4_ instead of CuBr	80	>99	>20:1
11	KO*^t^* Bu instead of NaO*^t^* Bu	75	99	>20:1
12	CuBr (5 mol %), **L1** (6 mol %)	>95	>99	>20:1

a Reaction conditions: **1a** (0.05 mmol), **2a** (0.075 mmol), CuBr (10 mol %), (*S*,*S*)-**L1** (12 mol %), B_2_pin_2_ (0.075 mmol), 1,4-dioxane (1 mL), rt, 12 h. Yield, dr, and rr values were determined by analysis of the ^1^H NMR spectra of the crude reaction mixture using CH_2_Br_2_ as an internal standard. The ee value was determined by chiral HPLC analysis.

Despite the broad utility of azetidines, methods for their synthesis have been underdeveloped as compared with the large ring homologues (e.g., pyrrolidines and piperidines), especially in enantioenriched forms.^5^ In particular, among different substitution patterns, the 2,3-disubstituted azetidines bearing two stereogenic centers are among the most challenging to construct.^5–8^ For a long time, the syntheses of enantioenriched azetidines have relied on diastereomeric induction from the existing chirality in a substrate or a stoichiometric chiral auxiliary.^3,7^ In contrast, direct catalytic enantioselective difunctionalization of an achiral precursor, at both the C-2 and C-3 positions with concomitant generation of two stereogenic centers, can be regarded as the most convenient approach. However, multiple challenges may be encountered in such transformations, other than achieving good reactivity. Indeed, effective controls over chemoselectivity, regioselectivity, enantioselectivity, and diastereoselectivity are all required. However, such an efficient protocol remains unavailable (Scheme 1B).

In continuation of our ongoing interests in the study of azetidines,^9^ we envisioned a potentially general approach to addressing the above unmet challenges (Scheme 1C). We hypothesized that direct enantioselective boryl allylation of azetines, a type of readily accessible substrate,^10^ would provide expedient access to diverse chiral 2,3-disubstituted azetidines since both boryl and allyl groups could be easily transformed to other functionalities. While copper-catalyzed enantioselective borylative difunctionalization of olefins has been established in various contexts,^11–13^ it proved not straightforward when applied to strained rings and electron-rich olefins.^14–17^ During the preparation of this manuscript, an elegant demonstration on strained cyclopropenes was reported by Liu and co-workers.^15^ However, there has been very limited success with strained heterocycles. The Brown laboratory pioneered a single example of boryl arylation of an azetine by Cu/Pd cocatalysis, but unfortunately with moderate enantioselectivity (74% ee).^17^ Compared with arylation, the formation of the C­(sp^3^)–C­(sp^3^) bond via alkylation with aliphatic electrophiles is expected to be more challenging due to low reactivity. Moreover, enantioselective difunctionalization of electron-rich double bonds (e.g., enamines and enamides) via a borylcupration mechanism remains largely unexplored in general.^11^ Herein, we report the first highly enantioselective boryl allylation of azetines, providing rapid access to diverse 2,3-disubstituted azetidines with high efficiency.

## Results and Discussion

Our study began with the model reaction between azetine **1a** and allylic electrophile **2a** with B_2_pin_2_ as the boron source (Table 1). After a comprehensive evaluation of various catalysts and reaction parameters, a combination of CuBr (10 mol %), the (*S*,*S*)-Ph-BPE ligand **L1** (12 mol %), and NaO^*t*^Bu (1.5 equiv) in 1,4-dioxane at room temperature was chosen as the standard conditions. Initial evaluation of some allylic electrophiles bearing a bromide (Br), acetate (OAc), or carbonate (OBoc) leaving group resulted in essentially no desired product formation (entry 1). In these cases, boryl azetidine **1a**-Bpin and allylboronate **2a**-Bpin were observed as the major products, which corroborated the challenge in forming the C­(sp^3^)–C­(sp^3^) bond in this type of difunctionalization reaction on strained electron-rich olefins. Nevertheless, to our delight, further screening indicated that phosphate is an ideal leaving group, forming the desired boryl allylation product **3a** in high yield with excellent enantio-, diastereo-, and regioselectivities (entries 2–3). Specifically, with dimethylphosphate as the leaving group, **3a** was formed essentially quantitatively as a single isomer in an enantiopure form (entry 3). A range of chiral bisphosphines and (P,N)-ligands were also examined, but they all led to inferior results (entry 4). For example, (*S*)-DTBM-Segphos **L3** and (*R*)-Phox **L8** failed to give the desired product, whereas­(*S*)-Binap **L2**, (*S*,*S*)-QuinoxP **L4**, (*R*,*S*_*p*_)-^*t*^Bu-Josiphos **L5**, (*S*,*R*_*p*_)-Josiphos **L6**, and (*S*,*S*_*p*_)-^*i*^Pr-Phosferrox **L7** led to a significant decrease in yield and/or selectivity. Notably, this reaction exhibited little sensitivity to the solvent. MTBE, THF, toluene, and DCM all gave complete control in enantio- and diastereo- and regioselectivities (>99% ee, >20:1 dr, and >20:1 rr), with a minor difference in reaction yield (entries 5–8). Similarly, different copper­(I) sources, including CuCl and Cu­(CH_3_CN)_4_BF_4_, maintained the high level of selectivities, albeit in slightly decreased yield (entries 9–10). The use of an alternative base, such as KO^*t*^Bu, led to a lower yield as well (entry 11). Finally, outstanding results could also be obtained at a reduced loading of catalyst/ligand, thus establishing the optimal conditions (entry 12). It is worth noting that this represents the first highly enantioselective Cu-catalyzed boryl alkylation of electron-rich olefins as well as strained heterocyclic olefins.

With the optimized conditions, we investigated the scope of the asymmetric boryl allylation with different 2-substituted allyl phosphates **2**, which resulted in rapid access to a range of *cis*-2,3-disubstituted azetidines **3** with a branched allyl group (Scheme 2). Allyl phosphates bearing different aryl (**3a**–**3f**) and alkenyl (**3g**) substituents were all effective partners in this three-component coupling. The structure and absolute configuration of product **3f** were unambiguously confirmed by X-ray crystallography. In addition, the simple allyl phosphate (**3l**) or those bearing an alkyl substituent (**3h**–**3k**) of varying steric demand also reacted efficiently. Notably, high chemoselectivity was observed in the reactions with other heterosubstituents in the allylic position (**3m**–**3o**), as they could be potentially labile toward additional substitution. It is remarkable that the desired products were uniformly obtained as a single isomer in enantiopure forms in all these cases (>99% ee, >20:1 dr, and >20:1 rr), thus highlighting the robustness of this difunctionalization process. We also evaluated other carbon-based electrophiles, such as simple alkyl, propargyl, and aryl halides or phosphates. Unfortunately, the corresponding boryl functionalization products were not obtained (see more details in the SI).

**Scheme 2:**
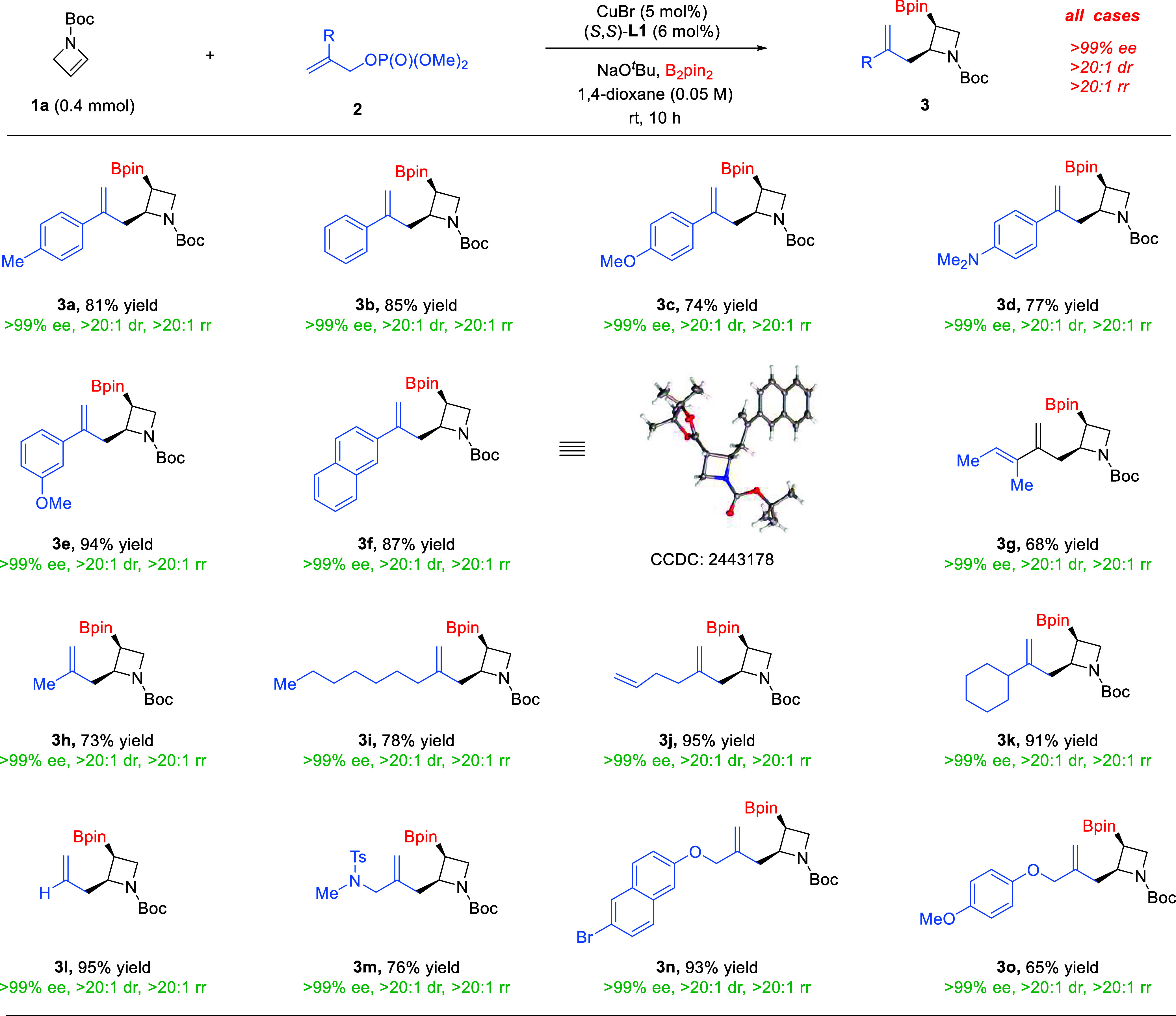
Branched Allylation Scope^a^

a Reaction conditions: **1a** (0.4 mmol), **2** (0.6 mmol), B_2_pin_2_ (0.6 mmol), NaO^*t*^Bu (0.6 mmol), CuBr (5 mol %), (*S*,*S*)-**L1** (6 mol %), 1,4-dioxane (8 mL), rt, 10 h. Isolated yield. Dr and rr values were determined by ^1^H NMR analysis of the crude product. The ee value was determined by HPLC on a chiral stationary phase.

The success of branched allylation further prompted us to explore the more challenging linear allylation reactions since the latter involves an additional selectivity control, i.e., *E*/*Z* ratio regarding the double bond configuration. A range of racemic allyl phosphates **4** bearing an allylic substituent (R^2^) were examined (Scheme 3). Notably, all these cases resulted in linear allylation products **5** with good to excellent site selectivity, suggesting that the substitution was in an exclusive S_N_2′ fashion. Again, all the products were formed with uniformly outstanding stereoselectivity as a single enantiomer, diastereomer, and regioisomer. In the presence of an additional substituent (**4i**), the corresponding trisubstituted olefin **5i** was also generated with high efficiency and good stereoselectivity. The mild conditions were compatible with different functional groups, including alkenes, thioethers, ethers, amines, and amides. Heterocycles could be successfully incorporated into the chiral azetidine products without an erosion in efficiency. Of note, this reaction exhibited good chemoselectivity when other C=C bonds were present in the substrates. Only the electron-rich azetine motif participated in boryl allylation. Finally, our protocol also permitted the facile modification of bioactive and natural molecules. Specifically, allylic phosphates derived from abietic acid, citronellal, and majantol all resulted in the corresponding azetidines **5j**–**5l** with good to excellent efficiency and stereoselectivities. The application in drug-like substrates and the potential to introduce azetidines in late-stage optimization of properties and potency are important.

**Scheme 3:**
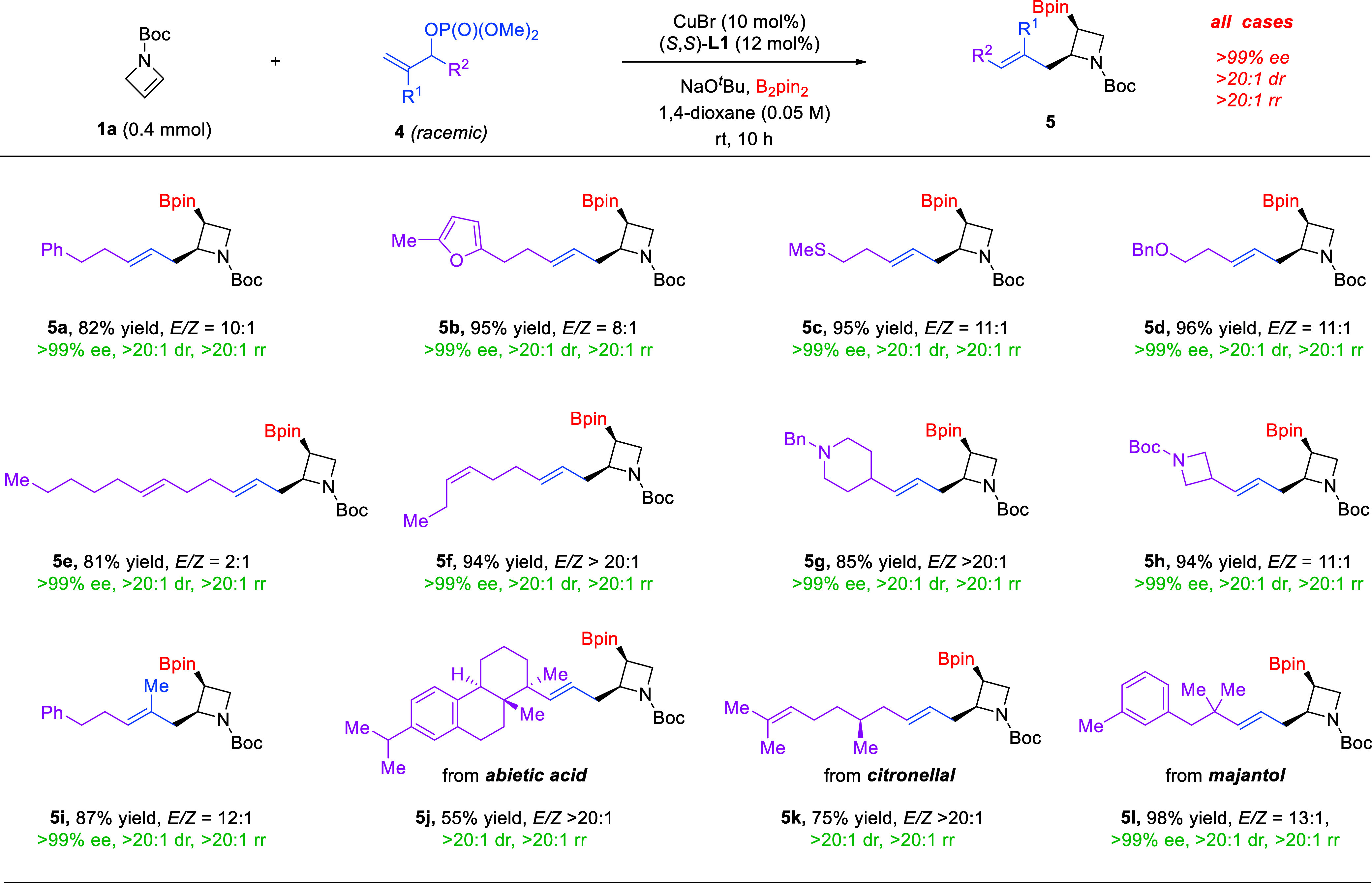
Linear Allylation Scope^a^

a Reaction conditions: **1a** (0.4 mmol), **4** (0.6 mmol), B_2_pin_2_ (0.6 mmol), NaO^*t*^Bu (0.6 mmol), CuBr (10 mol %), (*S*,*S*)-**L1** (12 mol %), 1,4-dioxane (8 mL), rt, 10 h. Isolated yield. Isolated yield. Dr, *E/Z*, and rr values were determined by ^1^H NMR analysis of the crude product. The ee value was determined by HPLC on a chiral stationary phase.

The present three-component coupling reaction permitted the convenient introduction of two versatile functionalities to the azetidine ring with complete absolute and relative stereocontrol. To further demonstrate its synthetic utility, we performed a gram-scale synthesis of azetidine **3e** by the optimized protocol (Scheme 4). Notably, the loading of CuBr and (*S*,*S*)-**L1** could be further reduced to 1 and 1.2 mol %, respectively, to achieve equally high efficiency and stereoselectivity (Scheme 4A). Next, some transformations of **3e** were performed. The Zweifel olefination^18^ of the boronate unit in **3e** with a vinyl Grignard reagent followed by ring-closing metathesis provided expedient access to enantiopure azabicyclo[3.2.0]­heptane **6**, a skeleton of significant medicinal value.^19^ The versatile boronate unit in **3e** could be easily transformed to other functionalities. For example, arylation with furan could be achieved with high stereospecificity in the presence of the *in situ* lithiated furan and NBS. Furthermore, homologative oxidation with CH_2_Br_2_ and *n*-BuLi smoothly afforded alcohol **8**. Alternatively, direct oxidation could lead to a secondary alcohol, which easily underwent bromination to form **9**. The boronate could be efficiently converted to potassium trifluoroborate salt **10**. A Pd-catalyzed Heck coupling with PhI was also successfully implemented, leading to exclusive C–C bond formation at the olefin terminal position but not at the Bpin unit. It could be envisioned that these molecules could serve as precursors to other functionalized azetidines after simple transformations. Notably, no erosion in the high enantiopurity was observed in these transformations.

**Scheme 4:**
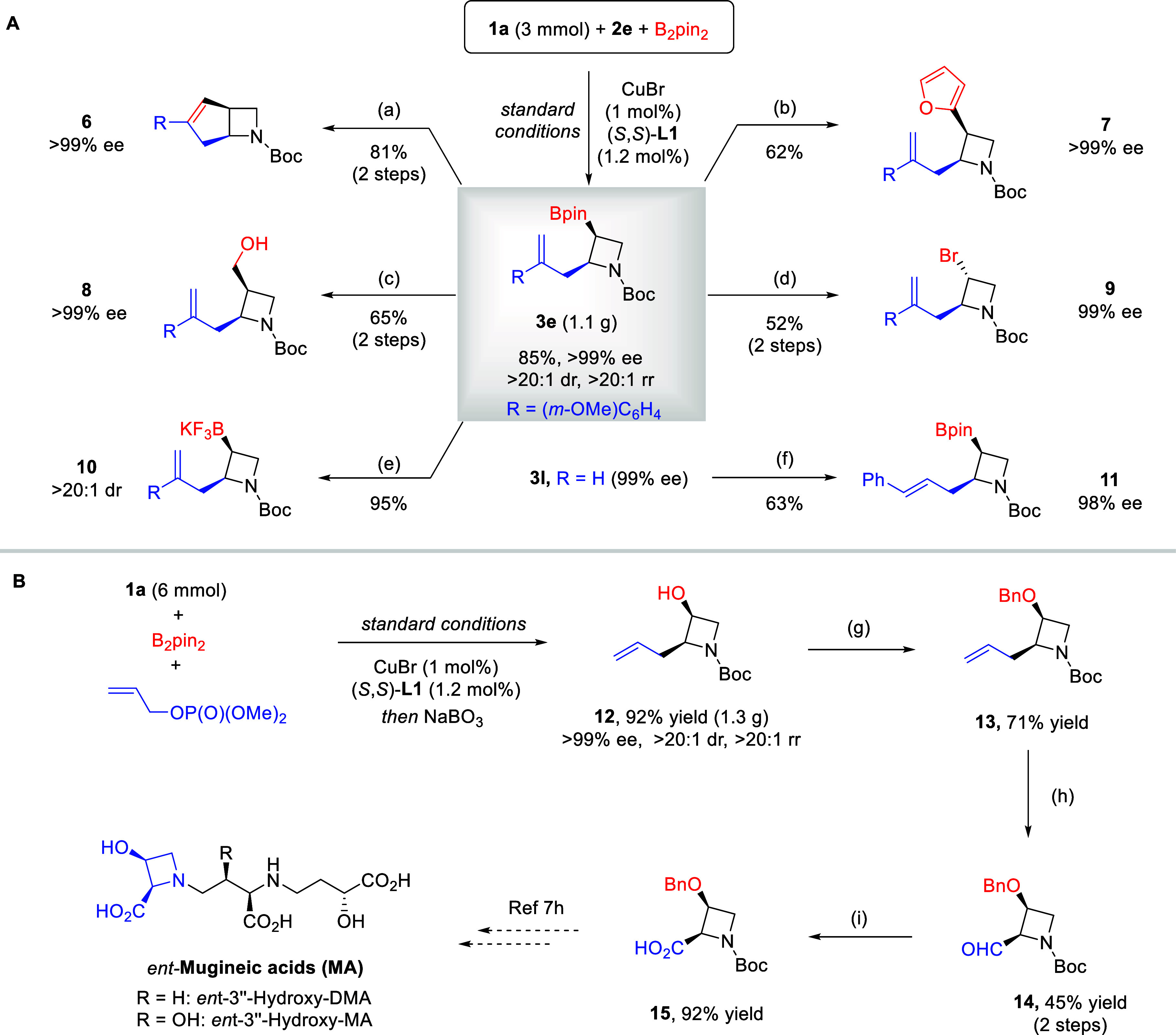
(A, B) Larger-Scale Reaction and Synthetic Applications^a^

a Reagents and conditions: (a) (vinyl)­MgBr, I_2_, NaOMe, −78 °C to rt; the Grubbs II catalyst, DCM, 60 °C. (b) Furan, *n-*BuLi, NBS, −78 °C to rt. (c) CH_2_Br_2_, *n-*BuLi, THF, −78 °C to rt; NaBO_3_ **·**4H_2_O, THF/H_2_O, rt. (d) NaBO_3_ **·**4H_2_O, THF/H_2_O, rt; PPh_3_, CBr_4_, toluene, 100 °C. (e) KHF_2_, MeOH/H_2_O, rt. (f) Pd­(dppf)­Cl_2_, PhI, Ag_2_O, Cs_2_CO_3_, THF/H_2_O, 80 °C. (g) BnBr, NaH, THF, 0 °C to rt. (h) the Grubbs II catalyst, CH_2_=CHOTMS, toluene, 120 °C; K_2_OsO_4_ **·**2H_2_O, NaIO_4_, NMO, ^*t*^BuOH, H_2_O, rt. (i) NaClO_2_, NaH_2_PO_4_, H_2_O_2_, MeCN, rt.

Our protocol can also provide access to advanced intermediates toward natural molecules (Scheme 4B). For example, a large-scale synthesis of **3l** followed by *in situ* oxidation delivered enantiopure 3-hydroxylazetidine **12**. After protection as a benzyl ether **13**, isomerization and oxidative cleavage of the double bond resulted in aldehyde **14**. Further oxidation then provided carboxylic acid **15**, an advanced intermediate leading to various stereoisomers of mugineic acids, known as phytosiderophores to facilitate iron uptake in plants.^7h^

Next, we performed experiments to gain some insight into the reaction mechanism (Figure 1). The addition of TEMPO did not affect the high efficiency, suggesting that this may not be a radical pathway (Figure 1A). The use of deuterated allylic electrophile **2b**-*d*_2_ resulted in **3b**-*d*_2_ with exclusive deuterium incorporation at the terminal position, indicating that this substitution is an intrinsic S_N_2′ process, but not by an S_N_2 pathway or via reductive elimination of a π-allyl species, which would lead to a mixture (Figure 1B). Furthermore, the product ee values showed a linear correlation with those of the ligand, thus consistent with the formation of a 1:1 adduct of the copper salt with the chiral bidentate ligand that dictates the enantio-determining bond formation (Figure 1C). Kinetic studies were also studied, which indicated that this process exhibits zeroth order in azetine and B_2_pin_2_, but first order in electrophile **2a** and the catalyst (Figure 1D).

**Figure 1:**
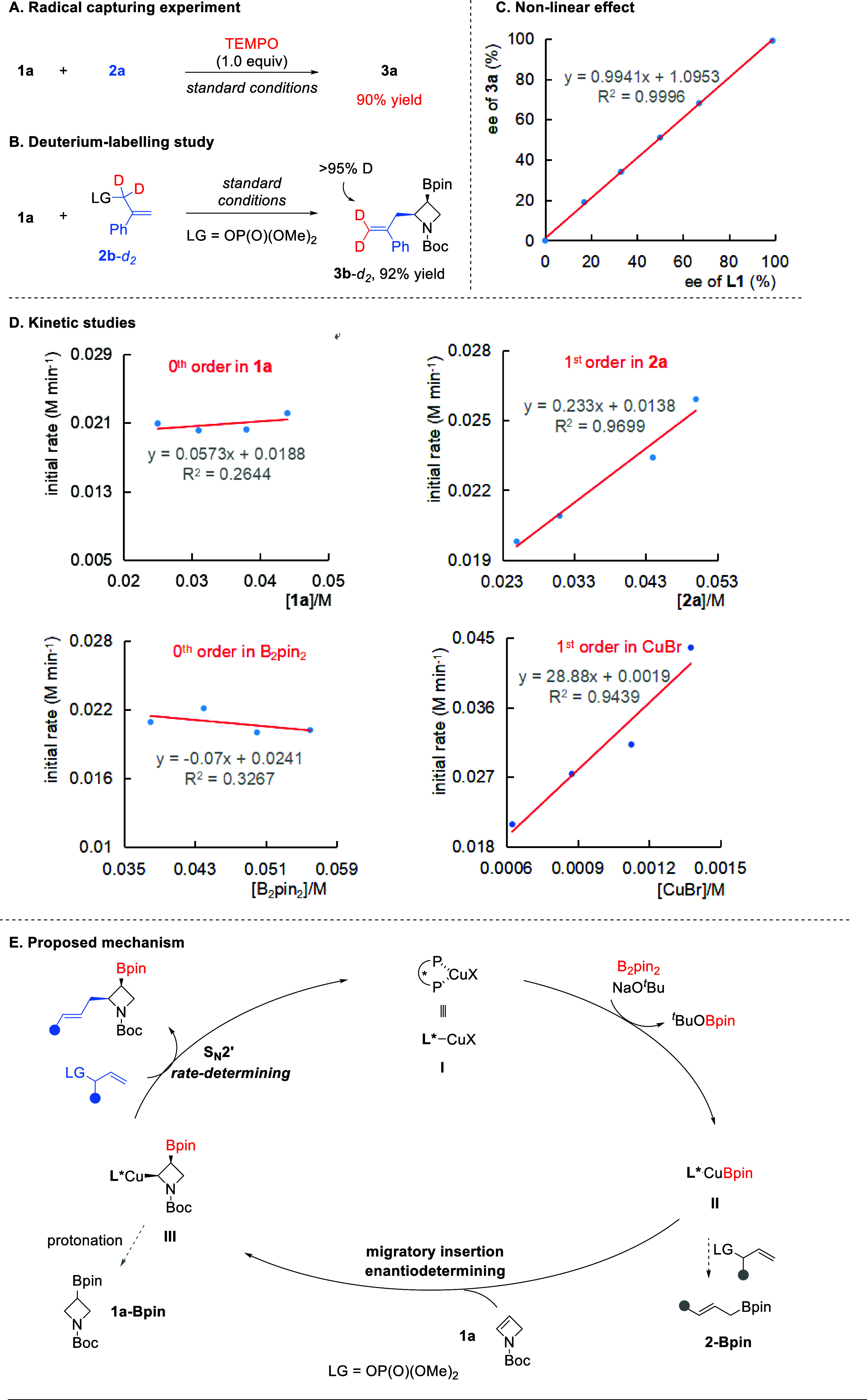
(A–E) Mechanistic studies and the proposed mechanism.

Based on these observations, we proposed a possible mechanism (Figure 1E). The reaction begins by forming Cu­(I)/bisphosphine complex **I**. Subsequent ligand exchange driven by the formation of a stable boronate ^*t*^BuO-Bpin generates the Cu-Bpin species **II**, which undergoes migratory insertion to the double bond of azetine **1a** to form the key alkyl cuprate **III**. The latter step is highly regioselective, with Bpin added exclusively to the 3-position. The *syn*-addition mechanism governs complete diastereoselectivity. The chiral catalyst also provides effective facial discrimination. Moreover, according to the kinetic data, this step is fast and thermodynamically favorable. This can also be regarded as fast saturation of the limiting Cu-Bpin species by azetine **1a**, resulting in pseudo zeroth order in **1a**. The subsequent C–C bond formation proceeds by nucleophilic attack to the less hindered terminal of the allylic electrophile in an S_N_2′ fashion, which is a slow step due to the low reactivity of the sterically hindered alkyl cuprate bearing an adjacent nitrogen atom. Therefore, the proper choice of an allylic phosphate electrophile is critical to ensure sufficient reactivity and to avoid competing protonation that would lead to **1a**-Bpin. It is also worth mentioning that the high reactivity of the azetine substrate is also critical to avoid direct addition of Cu-Bpin species **II** to the allylic electrophile, which would lead to side product **2**-Bpin. This also explains the high chemoselectivity even in the presence of other C=C bonds in the substrates.

## Conclusions

In summary, we have developed the first highly enantioselective direct difunctionalization of azetines for convenient access to chiral 2,3-disubstituted azetidines, a family of important scaffolds previously lacking general access. It also represents a rare demonstration of Cu-catalyzed asymmetric boryl alkylation of (heterosubstituted) electron-rich olefins and C=C bonds in strained heterocycles, despite the broad utility of this powerful olefin difunctionalization strategy. With the proper choice of a chiral bisphosphine ligand and allyl electrophiles, two versatile functionalities (boryl and allyl) were installed on the valuable azetidine ring with concomitant construction of two new stereogenic centers. The use of allyl phosphates proved critical not only to overcome the low reactivity of the borylcupration intermediate toward alkylation but also to avoid the side reactions such as direct functionalization of the allyl electrophile without involving azetine. It is remarkable that, in almost all the cases, single isomers were obtained with complete control over chemo-, regio-, enantio-, and diastereoselectivities in the azetidine motif as well as excellent control over the double bond configuration in the allyl group. The mild conditions exhibited outstanding functional group compatibility as well, leaving regular C=C bonds intact and thus showing great potential in facile modification of complex natural and drug molecules. The boryl and allyl units can be easily converted to other functionalities, thereby leading to other chiral azetidines that are not straightforward to access before. Control experiments and kinetic studies indicated that the reaction proceeds by a fast borylcupration of azetine followed by rate-determining allylation via an intrinsically controlled S_N_2′ pathway. Further extension of this efficient protocol is expected to address other challenges in organic synthesis.

## Supplementary Material



## Notes

Additional experimental and computational details and spectroscopic data of all compounds (PDF)
