# Correction to “Enantioselective Synthesis of 2,3-Disubstituted Azetidines via Copper-Catalyzed Boryl Allylation of Azetines”

**DOI:** 10.1021/jacs.5c22357

**Published:** 2026-01-19

**Authors:** Minghui Zhu, Jianwei Sun

The following errors in the original publication and its Supporting Information are hereby corrected. These changes do not alter the conclusions of the original work.

Page 24087. In Scheme 2, the single-crystal structure image for compound **3f** contained an error wherein a boron atom was erroneously replaced by a carbon atom. This has been corrected in the structure shown below:
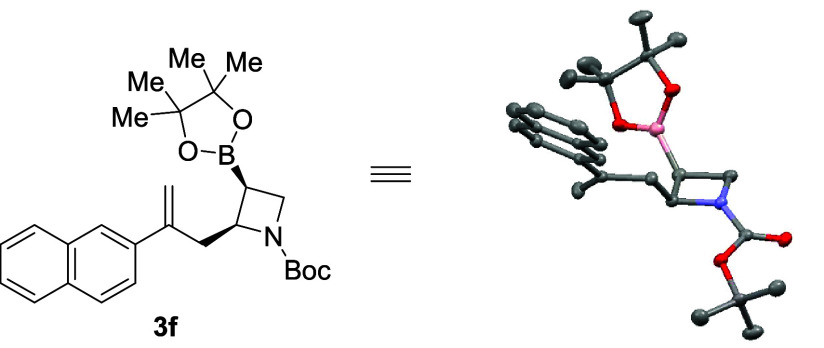


Detailed information regarding structural determination is provided in the revised Supporting Information, specifically in the Product Structure Determination section and Table S1 on pages S-61 and S-62.

Page 24091. The following sentence should be added to the Acknowledgments section: “The authors thank Dr. Herman H. Y. Sung for assistance in structure determination by X-ray crystallography.”

## Supplementary Material



## Notes

Additional experimental and computational details and spectroscopic data of all compounds (corrected) (PDF)

