# Supplementary material for: Enantioselective Synthesis of 2,3-Disubstituted Azetidines via Copper-Catalyzed Boryl Allylation of Azetines
Source: J Am Chem Soc. 2025 Jun 24;147(27):24085–94. doi: 10.1021/jacs.5c07821 (PMC12257529; doi:10.1021/jacs.5c07821)
Supplement: Supplementary file 1 [file ja5c07821_si_001.pdf]

Supporting Information

Enantioselective Synthesis of 2,3-Disubstituted Azetidines via Copper-Catalyzed Boryl Allylation of Azetines

Minghui Zhu and Jianwei Sun*

Department of Chemistry and the Hong Kong Branch of Chinese National Engineering Research Centre for Tissue Restoration & Reconstruction, The Hong Kong University of Science and Technology, Clear Water Bay, Kowloon, Hong Kong SAR, China

Table of Contents

I. General Information S-2

II. Preparation of Substrates S-3

III. Cu-Catalyzed Asymmetric Boryl Allylation Reaction S-18

IV. Gram-Scale Reaction S-39

V. Product Transformations S-40

VI. Asymmetric Synthesis of the Muginetic Acid Precursor S-47

VII. Mechanistic Experiments S-51

VIII. Product Structure Determination S-61

NMR Spectra and HPLC Traces

I. General Information

Flash column chromatography was performed over silica gel (200-300 or 300-400 mesh) purchased from Qindao Haiyang Co., China or SiliCycle® Inc., Canada. All air or moisture sensitive reactions were conducted in oven-dried glassware under nitrogen atmosphere using anhydrous solvents. Tetrahydrofuran was distilled from sodium/benzophenone. Anhydrous dichloromethane, methanol, toluene, acetonitrile, and *n*-hexane were purified by the Innovative® solvent purification system. Other anhydrous solvents were purchased from Sigma-Aldrich®, J&K® and Energy® and used as received. Chemicals were purchased from commercial suppliers, such as Sigma-Aldrich®, J&K®, Energy® and used without further purification unless otherwise stated. Bruker AVII, AVIII or NEO 400 spectrometer at 400 (^{1}H NMR), 101 MHz (^{13}C NMR), and 376 MHz (^{19}F NMR). Chemical shifts (δ values) were reported in ppm down field from an internal standard (^{1}H NMR: Me_4Si at 0.00 ppm and ^{13}C NMR: $CDCl_3$ at 77.00 ppm). Data for ^{1}H NMR were recorded as follows: chemical shift (δ, ppm), multiplicity (s = singlet; d = doublet; t = triplet; q = quarter; p = pentet; sept = septet; m = multiplet; br = broad), coupling constant (Hz), integration. The mass spectra were collected from a Waters Xevo G2-XS Tof, with ESI source; or a Waters GCT premier with EI/CI source. Optical rotations were measured on an AUTOPOL I Automatic polarimeter with $[\alpha]_D$ values reported in degrees; concentration (c) is in 10 mg/mL. Enantioselectivities were recorded on an Agilent HPLC instrument, using a chiral stationary phase column (Daicel CHIRALPAK® AD-3, AY-3, IA-3, IB N-3, IC-3, IE-3, CHIRALCEL® OD-3). The chiral HPLC methods were calibrated with the corresponding racemic mixtures.

II. Preparation of Substrates

The substrates were synthesized according to the following general procedures.

General Procedure A.

Linear allyl alcohol substrates **S1** were prepared according to the literature procedure.[1]

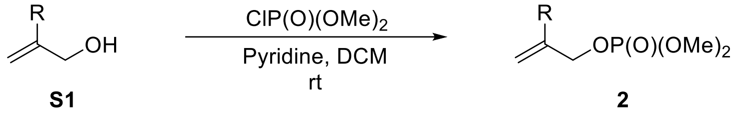

Under N_2, to an oven-dried flask charged with **S1** (5.0 mmol, 1.0 equiv) were added DCM (10 mL) and pyridine (0.6 mL, 7.5 mmol, 1.5 equiv). Then, dimethylphosphorchloridate (0.6 mL, 5.5 mmol, 1.1 equiv) was added dropwise at 0 ^{o}C. The reaction was stirred at room temperature and the progress was monitored by thin layer chromatography. Upon completion, water (20 mL) was added slowly to quench the reaction. The organic layer was separated, and the aqueous layer was extracted with DCM (30 mL × 3). The combined organic layers were dried over anhydrous Na_2SO_4 and concentrated under reduced pressure. The residue was purified by silica gel column chromatography to afford the desired product **2**.

---

(1) (a) Ching, J.; Jaschinski, M.; Choi, E. S.; Lautens, M. Synthesis of *α*-Quaternary Aldehydes via a Dual Ni/Rh-Catalyzed Tandem Isomerization-Propargylation Reaction. *ACS Catal.* **2024**, *14*, 15675–15682. (b) Zhao, X.; Gao, Xiang.; Zhao, F.; Wang, L.; Zhang, M.; Zhou, N. Substituent-Controlled Copper-Catalyzed Trifluoromethylation of 1,7-Dienes: Synthesis of Mono- and Bis-trifluoromethylated Benzoxepines. *Org. Lett.* **2024**, *26*, 7261–7266. (c) Morrison, S. D.; Liskamp, R. M. J.; Prunet, J. Tailoring Polyethers for Post-polymerization Functionalization by Cross Metathesis. *Org. Lett.* **2018**, *20*, 2253–2256.

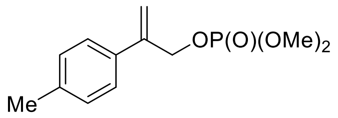

2a

Dimethyl (2-(*p*-tolyl)allyl) phosphate (2a) was prepared according to the General Procedure A as a yellow oil (chromatography eluent: *n*-hexane/EtOAc = 1:1) in 78% yield (998.8 mg).

^{1}H NMR (400 MHz, $CDCl_3$) δ 7.34 (d, *J* = 8.2 Hz, 2H), 7.15 (d, *J* = 7.9 Hz, 2H), 5.54 (s, 1H), 5.39 (s, 1H), 4.92 (d, *J* = 7.5 Hz, 2H), 3.71 (d, *J* = 11.1 Hz, 6H), 2.34 (s, 3H).

^{13}C NMR (101 MHz, $CDCl_3$) δ 142.6, 142.5, 138.2, 134.6, 129.3, 126.0, 114.8, 69.0 (*d*, *J* = 5.3 Hz), 54.42, 54.36 21.2.

HRMS (ESI) Calcd for $C_{12}H_{17}NaO_4P^+$ [M + Na]$^+$: 279.0757, Found:279.0761.

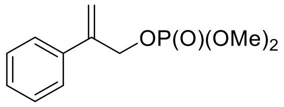

2b

Dimethyl (2-phenylallyl) phosphate (2b) was prepared according to the General Procedure A as a yellow oil (chromatography eluent: *n*-hexane/EtOAc = 1:1) in 75% yield (907.5 mg).

^{1}H NMR (400 MHz, $CDCl_3$) δ 7.46 – 7.44 (m, 2H), 7.37 – 7.29 (m, 3H), 5.58 (s, 1H), 5.45 (s, 1H), 4.95 (d, *J* = 7.5 Hz, 2H), 3.71 (d, *J* = 11.2 Hz, 6H).

^{13}C NMR (101 MHz, $CDCl_3$) δ 142.8, 142.7, 137.5, 128.6, 128.3, 126.1, 115.7, 69.0 (*d*, *J* = 5.4 Hz), 54.5, 54.4.

HRMS (ESI) Calcd for $C_{11}H_{15}NaO_4P^+$ [M + Na]$^+$: 265.0600, Found:265.0600.

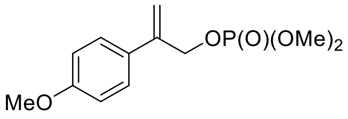

2c

2-(4-Methoxyphenyl)allyl dimethyl phosphate (2c) was prepared according to the General Procedure A as a yellow oil (chromatography eluent: *n*-

hexane/EtOAc = 1:1) in 68% yield (925.1 mg).

^{1}H NMR (400 MHz, $CDCl_3$) δ 7.39 (d, *J* = 8.8 Hz, 2H), 6.87 (d, *J* = 8.8 Hz, 2H), 5.49 (s, 1H), 5.34 (s, 1H), 4.90 (d, *J* = 8.0 Hz, 2H), 3.80 (s, 3H), 3.70 (d, *J* = 11.1 Hz, 6H).

^{13}C NMR (101 MHz, $CDCl_3$) δ 159.7, 142.1, 142.0, 130.0, 127.3, 114.1, 114.0, 69.15 (*d*, *J* = 5.4 Hz), 55.4, 54.5, 54.4.

HRMS (ESI) Calcd for $C_{12}H_{17}NaO_5P^+$ [M + Na]$^+$: 295.0706, Found:295.0711.

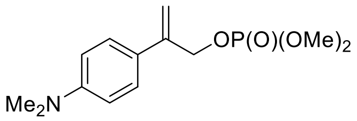

2d

2-(4-(Dimethylamino)phenyl)allyl dimethyl phosphate (2d) was prepared according to the General Procedure A as a yellow oil (chromatography eluent: *n*-hexane/EtOAc = 1:1) in 72% yield (1.0 g).

^{1}H NMR (400 MHz, $CDCl_3$) δ 7.35 (d, *J* = 8.9 Hz, 2H), 6.68 (d, *J* = 8.9 Hz, 2H), 5.45 (s, 1H), 5.25 (s, 1H), 4.91 (d, *J* = 7.5 Hz, 2H), 3.71 (d, *J* = 11.1 Hz, 6H), 2.95 (s, 6H).

^{13}C NMR (101 MHz, $CDCl_3$) δ 150.5, 142.1, 142.0, 126.8, 125.3, 112.2, 112.0, 69.2 (d, *J* = 5.4 Hz), 54.4, 54.3, 40.5.

HRMS (ESI) Calcd for $C_{13}H_{20}NNaO_4P^+$ [M + Na]$^+$: 308.1022, Found: 308.1031.

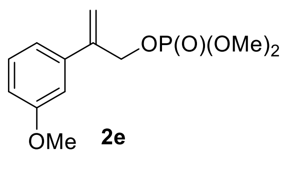

2e

2-**(3-Methoxyphenyl)allyl dimethyl phosphate (2e)** was prepared according to the General Procedure A as a yellow oil (chromatography eluent: *n*-hexane/EtOAc = 1:1) in 79% yield (1.1 g).

^{1}**H NMR** (400 MHz, $CDCl_3$) δ 7.27 (t, *J* = 7.9 Hz, 1H), 7.04 (d, *J* = 7.8 Hz, 1H), 6.99 (t, *J* = 2.1 Hz, 1H), 6.86 (dd, *J* = 8.1, 2.3 Hz, 1H), 5.59 (s, 1H), 5.45 (s, 1H), 4.92 (d, *J* = 7.5 Hz, 2H), 3.82 (s, 3H), 3.72 (d, *J* = 11.1 Hz, 6H).

^{13}**C NMR** (101 MHz, $CDCl_3$) δ 159.6, 142.5, 142.4, 138.8, 129.4, 118.4, 115.7, 113.5, 111.8, 68.8 (*d*, *J* = 5.4 Hz), 55.1, 54.24, 54.18.

HRMS (ESI) Calcd for $C_{12}H_{17}NaO_5P^+$ $[M + Na]^+$: 295.0706, Found:295.0709.

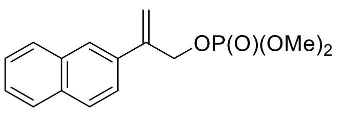

2f

Dimethyl (2-(naphthalen-2-yl)allyl) phosphate (2f) was prepared according to the General Procedure A as a yellow oil (chromatography eluent: *n*-hexane/EtOAc = 1:1) in 71% yield (1.0 g).

^{1}**H NMR** (400 MHz, $CDCl_3$) δ 7.88 (d, *J* = 1.9 Hz, 1H), 7.86 – 7.79 (m, 3H), 7.61 (dd, *J* = 8.6, 1.8 Hz, 1H), 7.48 (q, *J* = 5.2, 3.8 Hz, 2H), 5.74 (s, 1H), 5.55 (s, 1H), 5.07 (d, *J* = 8.1 Hz, 2H), 3.72 (d, *J* = 11.2 Hz, 6H).

^{13}**C NMR** (101 MHz, $CDCl_3$) δ 142.4, 142.3, 134.5, 133.2, 133.0, 128.2, 128.1, 127.5, 126.3, 126.2, 124.9, 124.0, 116.0, 68.9 (*d*, *J* = 5.3 Hz), 54.34, 54.28.

HRMS (ESI) Calcd for $C_{15}H_{17}NaO_4P^+$ $[M + Na]^+$: 315.0757, Found: 315.0761.

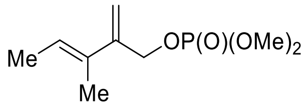

2g

(*E*)-Dimethyl (3-methyl-2-methylenepent-3-en-1-yl) phosphate (2g) was prepared according to the General Procedure A as a yellow oil (chromatography eluent: *n*-hexane/EtOAc = 1:1) in 82% yield (902.4 mg).

^{1}**H NMR** (400 MHz, $CDCl_3$) δ 5.42 (qt, *J* = 6.8, 1.5 Hz, 1H), 5.31 (p, *J* = 1.5 Hz, 1H), 4.94 (s, 1H), 4.52 (dd, *J* = 7.1, 1.2 Hz, 2H), 3.74 (dd, *J* = 11.1, 1.3 Hz, 6H), 1.80 – 1.76 (m, 3H), 1.60 (dt, *J* = 6.8, 1.5 Hz, 3H).

^{13}C NMR (101 MHz, $CDCl_3$) δ 143.7, 143.6, 134.3, 123.7, 114.9, 68.6 (*d*, *J* = 5.6 Hz), 54.4, 54.3, 23.3, 14.8.

HRMS (ESI) Calcd for $C_9H_{17}NaO_4P^+$ [M + Na]$^+$: 243.0757, Found: 243.0770.

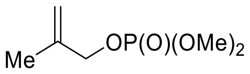

2h

Dimethyl (2-methylallyl) phosphate (2h) was prepared according to the General Procedure A as a yellow oil (chromatography eluent: *n*-hexane/EtOAc = 1:1) in 80% yield (720 mg).

^{1}H NMR (400 MHz, $CDCl_3$) δ 4.95 (s, 1H), 4.85 (s, 1H), 4.34 (s, 2H), 3.73 – 3.61 (m, 6H), 1.67 (s, 3H).

^{13}C NMR (101 MHz, $CDCl_3$) δ 139.94, 139.87, 113.3, 70.8 (*d*, *J* = 5.5 Hz), 54.2, 54.1, 18.8.

HRMS (ESI) Calcd for $C_6H_{13}NaO_4P^+$ [M + Na]$^+$: 203.0444, Found: 203.0444.

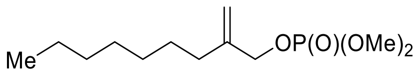

2i

Dimethyl (2-methylenenonyl) phosphate (2i) was prepared according to the General Procedure A as a yellow oil (chromatography eluent: *n*-hexane/EtOAc = 1:1) in 79% yield (1.0 g).

^{1}H NMR (400 MHz, $CDCl_3$) δ 5.05 (s, 1H), 4.91 (s, 1H), 4.43 (s, 2H), 3.73 (ddd, *J* = 11.0, 4.7, 2.0 Hz, 6H), 2.06 – 1.99 (m, 2H), 1.46 – 1.38 (m, 2H), 1.31 – 1.16 (m, 8H), 0.84 – 0.82 (m, 3H).

^{13}C NMR (101 MHz, $CDCl_3$) δ 144.3, 144.2, 112.5, 70.0 (*d*, *J* = 5.6 Hz), 54.34, 54.28, 32.6, 31.8, 29.3, 29.2, 27.5, 22.7, 14.1.

HRMS (ESI) Calcd for $C_{12}H_{25}NaO_4P^+$ [M + Na]$^+$: 287.1383, Found: 287.1388.

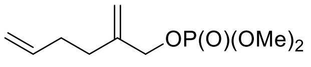

2j

Dimethyl (2-methylenehex-5-en-1-yl) phosphate (2i) was prepared according to the General Procedure A as a yellow oil (chromatography eluent: *n*-hexane/EtOAc = 1:1) in 84% yield (924 mg).

^{1}H NMR (400 MHz, $CDCl_3$) δ 5.72 (dddd, *J* = 19.3, 12.7, 6.2, 2.4 Hz, 1H), 5.04 (d, *J* = 5.3 Hz, 1H), 4.99 – 4.87 (m, 3H), 4.39 (t, *J* = 6.8 Hz, 2H), 3.72 – 3.67 (m, 6H), 2.18 – 2.08 (m, 4H).

^{13}C NMR (101 MHz, $CDCl_3$) δ 143.3, 143.2, 137.6, 115.0, 113.1, 69.9 (*d*, *J* = 5.4 Hz), 54.3, 54.2, 31.7, 31.5.

HRMS (ESI) Calcd for $C_9H_{17}NaO_4P^+$ $[M + Na]^+$: 243.0757, Found: 243.0769.

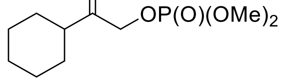

2k

2-Cyclohexylallyl dimethyl phosphate (2k) was prepared according to the General Procedure A as a yellow oil (chromatography eluent: *n*-hexane/EtOAc = 1:1) in 71% yield (880 mg).

^{1}H NMR (400 MHz, C) δ 5.05 (s, 1H), 4.92 (s, 1H), 4.47 (d, *J* = 6.8 Hz, 2H), 3.75 – 3.71 (m, 6H), 1.94 (t, *J* = 10.9 Hz, 1H), 1.75 (t, *J* = 10.9 Hz, 4H), 1.18 (dt, *J* = 32.3, 12.0 Hz, 6H).

^{13}C NMR (101 MHz, $CDCl_3$) δ 149.32, 149.25, 111.0, 69.4 (*d*, *J* = 5.6 Hz), 54.4, 54.3, 40.8, 32.2, 26.6, 26.3.

HRMS (ESI) Calcd for $C_{11}H_{21}NaO_4P^+$ $[M + Na]^+$: 271.1070, Found: 271.1079.

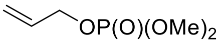

2l

Allyl dimethyl phosphate (2l) was prepared according to the General Procedure A as a yellow oil (chromatography eluent: *n*-hexane/EtOAc = 1:1) in

80% yield (664 mg).

^{1}H NMR (400 MHz, $CDCl_3$) δ 5.95 – 5.84 (m, 1H), 5.35 – 5.20 (m, 2H), 4.50 – 4.47 (m, 2H), 3.74 – 3.70 (m, 6H).

^{13}C NMR (101 MHz, $CDCl_3$) δ 132.5, 132.4, 118.4, 68.2 (*d*, *J* = 5.5 Hz), 54.34, 54.28.

HRMS (ESI) Calcd for $C_5H_{11}NaO_4P^+$ [M + Na]$^+$: 189.0287, Found: 189.0291.

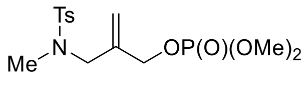

2m

2-(((*N*,4-Dimethylphenyl)sulfonamido)methyl)allyl dimethyl phosphate (2m) was prepared according to the General Procedure A as a yellow oil (chromatography eluent: *n*-hexane/EtOAc = 1:1) in 73% yield (1.3 g).

^{1}H NMR (400 MHz, $CDCl_3$) δ 7.67 (d, *J* = 8.3 Hz, 2H), 7.35 (d, *J* = 8.0 Hz, 2H), 5.37 (s, 1H), 5.20 (s, 1H), 4.56 (d, *J* = 7.2 Hz, 2H), 3.80 (d, *J* = 11.1 Hz, 6H), 3.62 (s, 2H), 2.63 (s, 3H), 2.45 (s, 3H).

^{13}C NMR (101 MHz, $CDCl_3$) δ 143.7, 138.6, 138.5, 133.7, 129.8, 127.5, 117.4, 67.5 (*d*, *J* = 5.4 Hz), 54.52, 54.46, 52.7, 34.5, 21.5.

HRMS (ESI) Calcd for $C_{14}H_{22}NNaO_6PS^+$ [M + Na]$^+$: 386.0798, Found: 386.0809.

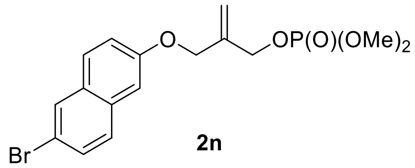

2n

2-(((6-Bromonaphthalen-2-yl)oxy)methyl)allyl dimethyl phosphate (2n) was prepared according to the General Procedure A as a yellow oil (chromatography eluent: *n*-hexane/EtOAc = 1:1) in 77% yield (1.5 g).

^{1}H NMR (400 MHz, $CDCl_3$) δ 7.90 (s, 1H), 7.63 (d, *J* = 9.0 Hz, 1H), 7.57 (d, *J* = 8.7 Hz, 1H), 7.48 (dd, *J* = 8.7, 1.8 Hz, 1H), 7.17 (dd, *J* = 9.0, 2.4 Hz, 1H), 7.11 (d, *J* = 2.1 Hz, 1H), 5.43 (d, *J* = 7.6 Hz, 2H), 4.70 – 4.68 (m, 4H), 3.75 (d, *J* = 11.1 Hz, 6H).

^{13}C NMR (101 MHz, $CDCl_3$) δ 156.7, 139.5, 139.4, 133.0, 130.2, 129.8, 129.7, 128.7,

128.5, 119.9, 117.4, 116.9, 107.2, 68.1, 67.9 (*d*, *J* = 5.4 Hz), 54.51, 54.45.

HRMS (ESI) Calcd for $C_{16}H_{18}BrNaO_5P^+$ [M + Na]$^+$: 422.9967, Found: 422.9973.

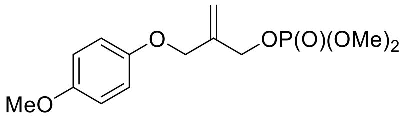

2o

2-((4-Methoxyphenoxy)methyl)allyl dimethyl phosphate (2o) was prepared according to the General Procedure A as a yellow oil (chromatography eluent: *n*-hexane/EtOAc = 1:1) in 71% yield (1.1 g).

^{1}H NMR (400 MHz, $CDCl_3$) δ 6.88 – 6.77 (m, 4H), 5.35 (s, 2H), 4.63 (d, *J* = 7.3 Hz, 2H), 4.51 (s, 2H), 3.76 – 3.71 (m, 9H).

^{13}C NMR (101 MHz, $CDCl_3$) δ 154.2, 152.6, 140.1, 140.0, 116.4, 115.8, 114.7, 68.8, 67.9 (*d*, *J* = 5.4 Hz), 55.8, 54.5, 54.4.

HRMS (ESI) Calcd for $C_{13}H_{19}NaO_6P^+$ [M + Na]$^+$: 325.0811, Found: 325.0816.

General Procedure B.

Branched allyl alcohol substrates **S2** were prepared according to the literature procedure.[2]

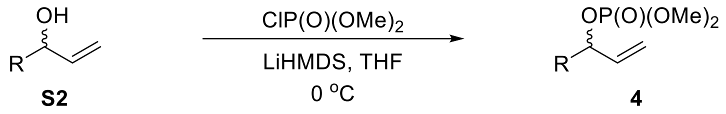

Under N_2, to a stirred suspension of **S2** (5.0 mmol, 1.0 equiv) in THF (20 mL) at 0 °C was slowly added LiHMDS (1.0 M solution in THF, 6.0 mL, 6.0 mmol). After stirring for 30 min at 0 °C, dimethylphosphorchloridate (0.6 mL, 5.5 mmol) was added dropwise. The reaction mixture was then allowed to warm to room temperature and the progress was monitored by thin layer chromatography. Upon completion (~1 h), water (20 mL) was added slowly to quench the reaction. The organic layer was separated, and the aqueous layer was extracted with ethyl acetate (30 mL × 3). The combined organic layers were dried over anhydrous Na_2SO_4 and concentrated under reduced pressure. The residue was purified by silica gel column chromatography to afford the desired product **4**.

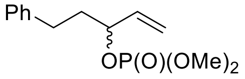

4a

Dimethyl (5-phenylpent-1-en-3-yl) phosphate (4a) was prepared according to the General Procedure B as a yellow oil (chromatography eluent: *n*-hexane/EtOAc = 1:1) in 89% yield (1.2 g).

H NMR (400 MHz, $CDCl_3$) δ 7.30 – 7.27 (m, 2H), 7.20 – 7.18 (m, 3H), 5.89 (ddd, *J* = 17.3, 10.4, 7.1 Hz, 1H), 5.38 – 5.26 (m, 2H), 4.81 (p, *J* = 6.9 Hz, 1H), 3.75 (dd, *J* = 11.1, 9.0 Hz, 6H), 2.77 – 2.64 (m, 2H), 2.12 – 2.03 (m, 2H), 2.00 – 1.90 (m, 2H).

---

(2) Zeng, X.; Yang, J.; Deng, W.; Feng, X.-T.; Zhao, H.-Y.; Wei, L.; Xue, X.-S.; Zhang, X. Copper Difluorocarbene Enables Catalytic Difluoromethylation. *J. Am. Chem. Soc.* **2024**, *146*, 16902–16911.

^{13}C NMR (101 MHz, $CDCl_3$) δ 141.3, 136.62, 136.58, 128.6, 128.5, 126.1, 117.9, 79.62, 79.56, 54.32, 54.31, 54.26, 54.25, 37.62, 37.56, 31.1.

HRMS (ESI) Calcd for $C_{13}H_{19}NaO_4P^+$ [M + Na]$^+$: 293.0913, Found: 293.0917.

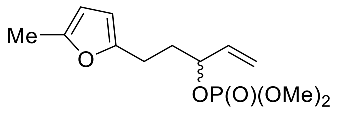

4b

Dimethyl (5-(5-methylfuran-2-yl)pent-1-en-3-yl) phosphate (4b) was prepared according to the General Procedure B as a yellow oil (chromatography eluent: *n*-hexane/EtOAc = 1:1) in 81% yield (1.1 g).

^{1}H NMR (400 MHz, $CDCl_3$) δ 5.88 – 5.80 (m, 3H), 5.32 (d, *J* = 17.2 Hz, 1H), 5.22 (d, *J* = 10.4 Hz, 1H), 4.79 – 4.76 (m, 1H), 3.75 – 3.69 (m, 6H), 2.63 (t, *J* = 7.7 Hz, 2H), 2.21 (s, 3H), 2.07 – 1.88 (m, 2H).

^{13}C NMR (101 MHz, $CDCl_3$) δ 152.9, 150.5, 136.4, 136.3, 117.9, 105.92, 105.85, 79.3, 79.2, 54.3, 54.2, 34.24, 34.18, 23.5, 13.5.

HRMS (ESI) Calcd for $C_{12}H_{19}NaO_5P^+$ [M + Na]$^+$: 297.0862, Found: 297.0874.

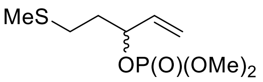

4c

Dimethyl (5-(methylthio)pent-1-en-3-yl) phosphate (4c) was prepared according to the General Procedure B as a yellow oil (chromatography eluent: *n*-hexane/EtOAc = 1:1) in 85% yield (1.0 g).

^{1}H NMR (400 MHz, $CDCl_3$) δ 5.87 – 5.75 (m, 1H), 5.34 – 5.19 (m, 2H), 4.82 (p, *J* = 6.9 Hz, 1H), 3.70 (dddd, *J* = 9.9, 8.7, 2.1, 1.0 Hz, 6H), 2.52 – 2.48 (m, 2H), 2.07 – 2.02 (m, 3H), 2.00 – 1.81 (m, 2H).

^{13}C NMR (101 MHz, $CDCl_3$) δ 136.13, 136.09, 118.0, 78.63, 78.57, 54.3, 54.2, 35.3, 35.2, 29.3, 15.4.

HRMS (ESI) Calcd for $C_8H_{17}NaO_4PS^+$ [M + Na]$^+$: 263.0477, Found: 263.0480.

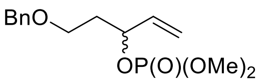

4d

5-(Benzyloxy)pent-1-en-3-yl dimethyl phosphate (4d) was prepared according to the General Procedure B as a yellow oil (chromatography eluent: *n*-hexane/EtOAc = 1:1) in 79% yield (1.2 g).

^{1}H NMR (400 MHz, $CDCl_3$) δ 7.39 – 7.27 (m, 5H), 5.86 (ddd, *J* = 17.4, 10.4, 7.1 Hz, 1H), 5.34 (d, *J* = 17.2 Hz, 1H), 5.22 (d, *J* = 10.4 Hz, 1H), 4.95 (p, *J* = 7.2 Hz, 1H), 4.50 (q, *J* = 11.8 Hz, 2H), 3.73 (dt, *J* = 11.1, 1.0 Hz, 6H), 3.62 – 3.51 (m, 2H), 2.09 – 1.90 (m, 2H).

^{13}C NMR (101 MHz, $CDCl_3$) δ 138.4, 136.63, 136.60, 128.5, 127.84, 127.75, 117.8, 77.6, 73.2, 66.0, 54.4, 54.36, 54.33, 54.31, 36.2, 36.1.

HRMS (ESI) Calcd for $C_{14}H_{21}NaO_5P^+$ $[M + Na]^+$: 323.1019, Found: 323.1026.

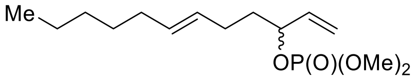

4e

(*E*)-Dodeca-1,6-dien-3-yl dimethyl phosphate (4e) was prepared according to the General Procedure B as a yellow oil (chromatography eluent: *n*-hexane/EtOAc = 1:1) in 88% yield (1.3 g).

^{1}H NMR (400 MHz, $CDCl_3$) δ 5.83 – 5.74 (m, 1H), 5.43 – 5.17 (m, 4H), 4.71 (p, *J* = 6.9, 6.5 Hz, 1H), 3.73 – 3.67 (m, 6H), 2.02 (q, *J* = 7.0 Hz, 2H), 1.92 (q, *J* = 6.5 Hz, 2H), 1.76 (dq, *J* = 13.9, 6.9, 6.4 Hz, 1H), 1.62 (dq, *J* = 13.9, 7.9 Hz, 1H), 1.32 – 1.23 (m, 6H), 0.85 – 0.82 (m, 3H).

^{13}C NMR (101 MHz, $CDCl_3$) δ 136.73, 136.70, 131.7, 128.5, 117.6, 79.8, 79.7, 54.21, 54.20, 54.15, 54.14, 35.8, 35.7, 32.6, 31.4, 29.2, 27.9, 22.6, 14.1.

HRMS (ESI) Calcd for $C_{14}H_{27}NaO_4P^+$ $[M + Na]^+$: 313.1539, Found: 313.1543.

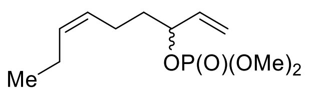

4f

(Z)-Dimethyl nona-1,6-dien-3-yl phosphate (4f) was prepared according to the General Procedure B as a yellow oil (chromatography eluent: *n*-hexane/EtOAc = 1:1) in 86% yield (1.1 g).

^{1}H NMR (400 MHz, $CDCl_3$) δ 5.79 (dddd, *J* = 17.4, 10.4, 7.1, 1.8 Hz, 1H), 5.38 – 5.16 (m, 4H), 4.71 (p, *J* = 7.5 Hz, 1H), 3.70 (ddd, *J* = 11.3, 9.9, 1.9 Hz, 6H), 2.11 – 1.93 (m, 4H), 1.79 – 1.70 (m, 1H), 1.67 – 1.57 (m, 1H), 0.90 (td, *J* = 7.5, 1.8 Hz, 3H).

^{13}C NMR (101 MHz, $CDCl_3$) δ 136.62, 136.59, 132.7, 127.5, 117.7, 79.80, 79.75, 54.21, 54.18, 54.15, 54.12, 35.92, 35.86, 22.5, 20.5, 14.3.

HRMS (ESI) Calcd for $C_{11}H_{21}NaO_4P^+$ [M + Na]$^+$: 271.1070, Found: 271.1079.

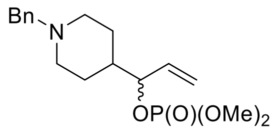

4g

1-(1-Benzylpiperidin-4-yl)allyl dimethyl phosphate (4g) was prepared according to the General Procedure B as a yellow oil (chromatography eluent: *n*-hexane/EtOAc = 1:2) in 65% yield (1.0 g).

^{1}H NMR (400 MHz, $CDCl_3$) δ 7.31 (d, *J* = 4.4 Hz, 4H), 7.28 - 7.25 (m, 1H), 5.81 (ddd, *J* = 17.2, 10.4, 7.7 Hz, 1H), 5.38 – 5.22 (m, 2H), 4.54 (q, *J* = 7.4 Hz, 1H), 3.74 (dd, J = 11.9, 11.2 Hz, 6H), 3.51 (s, 2H), 2.98 - 2.87 (m, 2H), 1.94 (t, *J* = 11.6 Hz, 2H), 1.70 – 1.52 (m, 3H), 1.50 – 1.32 (m, 2H).

^{13}C NMR (101 MHz, $CDCl_3$) δ 134.2, 132.2, 128.8, 128.3, 127.7, 118.4, 83.0, 62.4, 53.62, 53.56, 52.5, 40.1, 29.1, 28.7, 26.6.

HRMS (ESI) Calcd for$C_{17}H_{27}NO_4P^+$ [M + H]$^+$: 340.1672, Found: 340.1678.

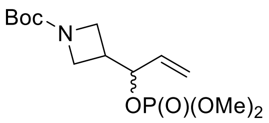

4h

***tert*-Butyl 3-(1-((dimethoxyphosphoryl)oxy)allyl)azetidine-1-carboxylate (4h)** was prepared according to the General Procedure B as a yellow oil (chromatography eluent: *n*-hexane/EtOAc = 1:1) in 71% yield (1.1 g).

^{1}H NMR (400 MHz, $CDCl_3$) δ 5.87 – 5.70 (m, 1H), 5.45 – 5.26 (m, 2H), 4.86 (q, *J* = 7.3 Hz, 1H), 3.98 – 3.84 (m, 3H), 3.82 – 3.67 (m, 6H), 2.77 (h, *J* = 7.4 Hz, 1H), 1.42 (s, 6H).

^{13}C NMR (101 MHz, $CDCl_3$) δ 156.2, 133.63, 133.61, 119.6, 80.54, 80.49, 79.6, 54.46, 54.44, 54.40, 54.38, 50.5, 32.94, 32.87, 28.4.

HRMS (ESI) Calcd for $C_{13}H_{24}NNaO_6P^+$ $[M + Na]^+$: 344.1233, Found: 344.1239.

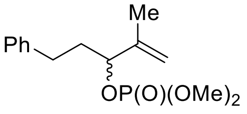

4i

Dimethyl (2-methyl-5-phenylpent-1-en-3-yl) phosphate (4i) was prepared according to the General Procedure B as a yellow oil (chromatography eluent: *n*-hexane/EtOAc = 1:1) in 81% yield (1.2 g).

^{1}H NMR (400 MHz, $CDCl_3$) δ 7.30 – 7.27 (m, 2H), 7.20 – 7.17 (m, 3H), 5.07 – 5.05 (m, 1H), 5.00 – 4.98 (m, 1H), 4.78 (q, *J* = 6.8 Hz, 1H), 3.74 (t, *J* = 11.1 Hz, 6H), 2.71 – 2.56 (m, 2H), 2.14 – 2.05 (m, 2H), 1.99 – 1.89 (m, 2H), 1.77 (s, 3H).

^{13}C NMR (101 MHz, $CDCl_3$) δ 142.81, 142.78, 141.3, 128.5, 128.4, 126.1, 114.6, 82.4, 82.3, 54.29, 54.28, 54.23, 54.22, 35.8, 35.7, 31.4, 16.9.

HRMS (ESI) Calcd for $C_{14}H_{21}NaO_4P^+$ $[M + Na]^+$: 307.1070, Found: 307.1071.

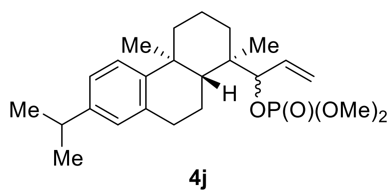

4j

((1*R*,4a*S*,10a*R*)-7-Isopropyl-1,4a-dimethyl-1,2,3,4,4a,9,10,10a-octahydrophe nanthren-1-yl)allyl dimethyl phosphate (4j) was prepared according to the General Procedure B as a yellow oil (chromatography eluent: *n*-hexane/EtOAc = 1:1) in 61% yield (1.3 g).

^{1}H NMR (400 MHz, $CDCl_3$) δ 7.16 (d, *J* = 8.2 Hz, 1H), 6.98 (dd, J = 8.1, 2.1 Hz, 1H), 6.86 (d, *J* = 2.0 Hz, 1H), 5.91 (ddd, *J* = 17.1, 10.4, 8.7 Hz, 1H), 5.43 – 5.29 (m, 2H), 4.80 (dd, *J* = 8.7, 7.2 Hz, 1H), 3.73 (dd, *J* = 27.0, 11.1 Hz, 6H), 2.86 – 2.66 (m, 3H), 2.27 (dd, *J* = 12.9, 1.8 Hz, 1H), 1.78 – 1.52 (m, 7H), 1.37 – 1.28 (m, 2H), 1.23 – 1.15 (m, 11H).

^{13}C NMR (101 MHz, $CDCl_3$) δ 147.1, 145.6, 134.4, 133.4, 126.8, 124.3, 123.9, 120.1, 87.7, 87.6, 54.2, 54.12, 54.11, 54.06, 44.2, 41.0, 40.9, 38.2, 37.4, 33.4, 30.5, 29.7, 25.5, 23.9, 18.7, 18.2, 17.9.

HRMS (ESI) Calcd for $C_{24}H_{37}NaO_4P^+$ $[M + Na]^+$: 443.2322, Found: 443.2325.

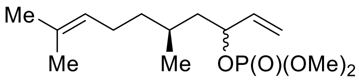

4k

(4*S*)-4,8-Dimethylnona-1,7-dien-3-yl dimethyl phosphate (4k) was prepared according to the General Procedure B as a yellow oil (chromatography eluent: *n*-hexane/EtOAc = 1:1) in 84% yield (1.2 g).

^{1}H NMR (400 MHz, $CDCl_3$) δ 5.86 – 5.73 (m, 1H), 5.32 – 5.16 (m, 2H), 5.06 – 5.03 (m, 1H), 4.79 (dp, *J* = 14.0, 7.2 Hz, 1H), 3.73 – 3.67 (m, 6H), 1.92 (tt, *J* = 14.4, 7.6 Hz, 2H), 1.77 – 1.49 (m, 8H), 1.41 – 1.09 (m, 3H), 0.90 (dd, *J* = 16.3, 6.5 Hz, 3H).

^{13}C NMR (101 MHz, $CDCl_3$) δ 137.5, 137.4, 137.02, 136.99, 131.4, 131.3, 124.60, 124.58, 117.8, 117.3, 79.0, 78.9, 78.54, 78.48, 54.2, 54.14, 54.09, 43.4, 43.3, 43.1, 43.0, 37.3, 36.9, 28.7, 28.5, 25.8, 25.4, 25.3, 19.6, 19.3, 17.7.

HRMS (ESI) Calcd for $C_{14}H_{27}NaO_4P^+$ [M + Na]$^+$: 313.1539, Found: 313.1543.

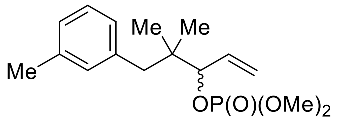

4l

4,4-Dimethyl-5-(*m*-tolyl)pent-1-en-3-yl dimethyl phosphate (4l) was prepared according to the General Procedure B as a yellow oil (chromatography eluent: *n*-hexane/EtOAc = 1:1) in 75% yield (1.2 g).

^{1}H NMR (400 MHz, $CDCl_3$) δ 7.16 (t, *J* = 7.5 Hz, 1H), 7.03 (d, *J* = 7.6 Hz, 1H), 6.94 (d, *J* = 8.7 Hz, 2H), 6.00 – 5.91 (m, 1H), 5.40 – 5.35 (m, 2H), 4.52 (t, *J* = 7.5 Hz, 1H), 3.76 (dd, *J* = 18.1, 11.2 Hz, 6H), 2.58 (q, *J* = 13.0 Hz, 2H), 2.33 (s, 3H), 0.90 (s, 3H), 0.84 (s, 3H).

^{13}C NMR (101 MHz, $CDCl_3$) δ 138.0, 137.4, 133.9, 133.8, 131.9, 128.1, 127.8, 126.9, 120.2, 87.8, 87.7, 54.4, 54.33, 54.31, 54.27, 43.7, 38.9, 38.8, 22.8, 22.4, 21.6.

HRMS (ESI) Calcd for $C_{16}H_{25}NaO_4P^+$ [M + Na]$^+$: 335.1383, Found: 335.1389.

III. Cu-Catalyzed Asymmetric Boryl Allylation Reaction

General Procedure C.

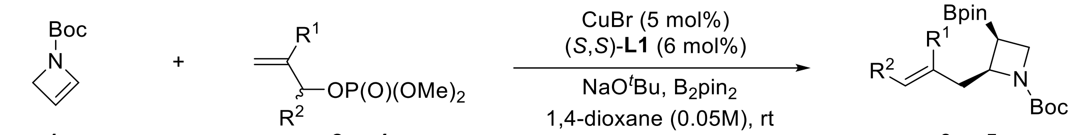

1a **2** or **4** **3** or **5**

In a glove box, a mixture of CuBr (2.9 mg, 0.02 mmol, 5.0 mol%) and the chiral ligand (*S*,*S*)-**L1** (12.2 mg, 0.024 mmol, 6.0 mol%) in dry 1,4-dioxane (0.05 M, 8.0 mL) was stirred at room temperature for 0.5 h. Then, azetine **1a** (62.0 mg, 0.4 mmol, 1.0 equiv), B_2pin_2 (152.3 mg, 0.6 mmol, 1.5 equiv), NaO^tBu (57.7 mg, 0.6 mmol, 1.5 equiv), and allyl phosphate **2** or **4** (0.6 mmol, 1.5 equiv) were added sequentially. The mixture was stirred at room temperature for 10 h. Next, the mixture was concentrated *in vacuo*, and the crude product was used to determine the regioselectivity and diastereoselectivity by ^1H NMR analysis. Finally, the residue was purified by silica gel column chromatography to afford the desired product **3** or **5**.

The racemic samples were prepared according to the General Procedure C at a 0.1-mmol scale with *rac*-BINAP as the ligand.

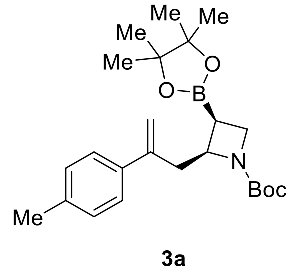

3a

***tert*-Butyl(2*S*,3*S*)-3-(4,4,5,5-tetramethyl-1,3,2-dioxaborolan-2-yl)-2-(2-(*p*-tolyl) allyl)azetidine-1-carboxylate (3a)** was prepared according to the General Procedure C as a yellow oil (chromatography eluent: *n*-hexane/EtOAc = 10:1) in 81% yield (133.9 mg, >99% ee, >20:1 rr).

[α]$_D^{25}$: +57.2 (c = 3.3, $CHCl_3$). HPLC analysis of the product: Daicel CHIRALPAK® OX-3 column; 10% *i*-PrOH in *n*-hexane; 1.0 mL/min; retention times: 5.2 min (major), 6.2 min (minor).

^{1}H NMR (400 MHz, $CDCl_3$) δ 7.34 (d, *J* = 8.2 Hz, 2H), 7.11 (d, *J* = 8.0 Hz, 2H), 5.31 (s, 1H), 4.86 (s, 1H), 4.66 – 4.52 (m, 1H), 3.93 (dd, *J* = 9.9, 7.8 Hz, 1H), 3.82 (dd, *J* = 7.8, 6.1 Hz, 1H), 3.08 (dt, *J* = 26.4, 15.6 Hz, 2H), 2.33 (s, 3H), 2.21 (td, *J* = 9.6, 6.0 Hz, 1H), 1.43 (s, 9H), 1.18 (s, 12H).

^{13}C NMR (101 MHz, $CDCl_3$) δ 156.7, 144.4, 138.3, 137.2, 129.0, 125.9, 111.7, 83.7, 79.3, 61.6, 48.3, 37.7, 28.6, 25.3, 24.7, 21.2, 16.9.

HRMS (ESI) Calcd for $C_{24}H_{36}BNNaO_4^+$ [M + Na]$^+$: 436.2630, Found: 436.2638.

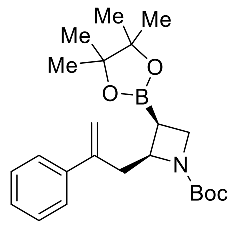

3b

***tert*-Butyl(2*S*,3*S*)-2-(2-phenylallyl)-3-(4,4,5,5-tetramethyl-1,3,2-dioxaborolan-2-yl)azetidine-1-carboxylate (3b)** was prepared according to the General Procedure C as a yellow oil (chromatography eluent: *n*-hexane/EtOAc = 10:1) in 85% yield (135.7 mg, >99% ee, >20:1 rr).

[α]$_D^{25}$: +11.0 (c = 3.6, $CHCl_3$). HPLC analysis of the product: Daicel CHIRALPAK® IC-3 column; 10% *i*-PrOH in *n*-hexane; 1.0 mL/min; retention times: 7.1 min (major), 8.1 min (minor).

^{1}H NMR (400 MHz, $CDCl_3$) δ 7.47 – 7.44 (m, 2H), 7.33 – 7.24 (m, 3H), 5.36 (s, 1H), 4.92 (s, 1H), 4.67 – 4.55 (m, 1H), 3.94 (dd, *J* = 9.9, 7.9 Hz, 1H), 3.84 – 3.81 (m, 1H), 3.18 – 3.02 (m, 1H), 2.23 (td, *J* = 9.6, 6.1 Hz, 1H), 1.43 (s, 9H), 1.18 (s, 12H).

^{13}C NMR (101 MHz, $CDCl_3$) δ 156.8, 144.7, 141.3, 128.3, 127.6, 126.0, 112.6, 83.8, 79.4, 61.6, 48.3, 37.7, 28.6, 25.3, 24.8, 16.8.

HRMS (ESI) Calcd for $C_{23}H_{34}BNNaO_4^+$ [M + Na]$^+$: 422.2473, Found: 422.2482.

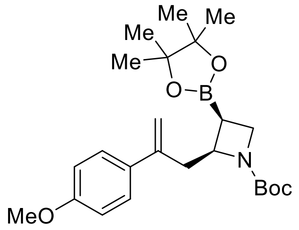

3c

***tert*-Butyl(2*S*,3*S*)-2-(2-(4-methoxyphenyl)allyl)-3-(4,4,5,5-tetramethyl-1,3,2-dioxaborolan-2-yl)azetidine-1-carboxylate (3c)** was prepared according to the General Procedure C as a yellow oil (chromatography eluent: *n*-hexane/EtOAc = 10:1) in 74% yield (127.1 mg, >99% ee, >20:1 rr).

$[\alpha]_D^{25}$: +34.6 (c = 3.2, $CHCl_3$). HPLC analysis of the product: Daicel CHIRALPAK® OX-3 column; 10% *i*-PrOH in *n*-hexane; 1.0 mL/min; retention times: 6.5 min (major), 8.3 min (minor).

^{1}H NMR (400 MHz, $CDCl_3$) δ 7.38 (d, *J* = 8.8 Hz, 2H), 6.83 (d, *J* = 8.8 Hz, 2H), 5.26 (s, 1H), 4.81 (s, 1H), 4.67 – 4.53 (m, 1H), 3.92 (dd, *J* = 9.8, 8.0 Hz, 1H), 3.82 – 3.78 (m, 4H), 3.05 (dt, *J* = 26.3, 14.1 Hz, 2H), 2.21 (td, *J* = 9.6, 6.1 Hz, 1H), 1.41 (s, 9H), 1.16 (s, 12H).

^{13}C NMR (101 MHz, $CDCl_3$) δ 159.2, 156.7, 143.8, 133.6, 127.0, 113.6, 110.8, 83.7, 79.3, 61.6, 55.3, 48.0, 37.6, 28.6, 25.2, 24.7, 17.0.

HRMS (ESI) Calcd for $C_{24}H_{36}BNNaO_5^+$ $[M + Na]^+$: 452.2579, Found: 452.2585.

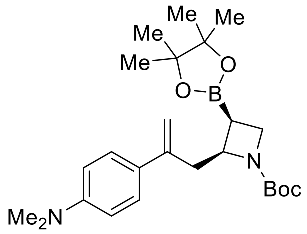

3d

***tert*-Butyl(2*S*,3*S*)-2-(2-(4-(dimethylamino)phenyl)allyl)-3-(4,4,5,5-tetramethyl-1,3,2-dioxaborolan-2-yl)azetidine-1-carboxylate (3d)** was prepared according to the General Procedure C as a yellow oil (chromatography eluent: *n*-hexane/EtOAc = 10:1) in 77% yield (136.2 mg, >99% ee, >20:1 rr).

$[\alpha]_D^{25}$: +46.7 (c = 1.3, $CHCl_3$). HPLC analysis of the product: Daicel CHIRALPAK® ID-3 column; 10% *i*-PrOH in *n*-hexane; 1.0 mL/min; retention times: 7.2 min (major), 6.2 min (minor).

^{1}H NMR (400 MHz, $CDCl_3$) δ 7.36 (d, *J* = 8.9 Hz, 2H), 6.67 (d, *J* = 8.9 Hz, 2H), 5.24 (d, *J* = 1.3 Hz, 1H), 4.74 (s, 1H), 4.68 – 4.57 (m, 1H), 3.93 (dd, *J* = 10.0, 7.8 Hz, 1H), 3.82 (dd, *J* = 7.8, 6.1 Hz, 1H), 3.11 – 2.91 (m, 8H), 2.21 (td, *J* = 9.6, 6.1 Hz, 1H), 1.43 (s, 9H), 1.18 (s, 12H).

^{13}C NMR (101 MHz, $CDCl_3$) δ 156.8, 150.1, 144.0, 129.2, 126.7, 112.2, 109.2, 83.7, 79.3, 61.6, 48.0, 40.6, 37.5, 28.6, 25.3, 24.7, 16.9.

HRMS (ESI) Calcd for $C_{25}H_{39}BN_2NaO_4^+$ $[M + Na]^+$: 465.2895, Found:465.2903.

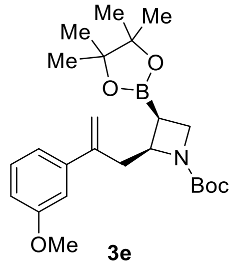

3e

***tert*-Butyl(2*S*,3*S*)-2-(2-(3-methoxyphenyl)allyl)-3-(4,4,5,5-tetramethyl-1,3,2-dioxaborolan-2-yl)azetidine-1-carboxylate (3e)** was prepared according to the General Procedure C as a white solid (chromatography eluent: *n*-hexane/EtOAc = 10:1) in 94% yield (161.4 mg, >99% ee, >20:1 rr).

$[\alpha]_D^{25}$: +67.6 (c = 3.0, $CHCl_3$). HPLC analysis of the product: Daicel CHIRALPAK® OZ-3 column; 10% *i*-PrOH in *n*-hexane; 1.0 mL/min; retention times: 5.4 min (major), 7.3 min (minor).

^{1}H NMR (400 MHz, $CDCl_3$) δ 7.21 (t, *J* = 7.9 Hz, 1H), 7.03 (d, *J* = 7.8 Hz, 1H), 6.98 (s, 1H), 6.80 (dd, *J* = 8.1, 2.1 Hz, 1H), 5.34 (s, 1H), 4.90 (s, 1H), 4.64 – 4.54 (m, 1H), 3.93 (dd, *J* = 9.7, 8.0 Hz, 1H), 3.83 – 3.80 (m, 4H), 3.05 (q, *J* = 16.9, 16.1 Hz, 2H), 2.22 (td, *J* = 9.6, 6.1 Hz, 1H), 1.42 (s, 9H), 1.17 (s, 12H).

^{13}C NMR (101 MHz, $CDCl_3$) δ 159.6, 156.7, 144.6, 142.8, 129.2, 118.6, 112.9, 112.7, 111.9, 83.7, 79.4, 61.6, 55.3, 48.3, 37.8, 28.6, 25.2, 24.7, 16.8.

HRMS (ESI) Calcd for $C_{24}H_{36}BNNaO_5^+$ [M + Na]$^+$: 452.2579, Found: 452.2586.

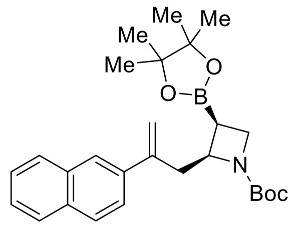

3f

***tert*-Butyl(2*S*,3*S*)-2-(2-(naphthalen-2-yl)allyl)-3-(4,4,5,5-tetramethyl-1,3,2-dioxaborolan-2-yl)azetidine-1-carboxylate (3f)** was prepared according to the General Procedure C as a white solid (chromatography eluent: *n*-hexane/EtOAc = 10:1) in 87% yield (156.3 mg, >99% ee, >20:1 rr).

$[\alpha]_D^{25}$: +67.3 (c = 3.0, $CHCl_3$). HPLC analysis of the product: Daicel CHIRALPAK® ID-3 column; 10% *i*-PrOH in *n*-hexane; 1.0 mL/min; retention times: 5.8 min (major), 5.4 min (minor).

^{1}H NMR (400 MHz, $CDCl_3$) δ 7.91 (s, 1H), 7.85 – 7.77 (m, 3H), 7.63 (dd, *J* = 8.6, 1.8 Hz, 1H), 7.45 (ddt, *J* = 9.5, 6.9, 3.5 Hz, 2H), 5.52 (s, 1H), 5.02 (s, 1H), 4.77 – 4.63 (m, 1H), 3.98 (dd, *J* = 9.9, 7.9 Hz, 1H), 3.89 – 3.86 (m, 1H), 3.23 (q, *J* = 15.9, 14.5 Hz, 3H), 2.28 (td, *J* = 9.6, 6.1 Hz, 1H), 1.45 (s, 9H), 1.17 (d, *J* = 2.9 Hz, 12H).

^{13}C NMR (101 MHz, $CDCl_3$) δ 156.7, 144.4, 138.5, 133.4, 132.9, 128.3, 127.8, 127.6, 126.2, 125.9, 124.7, 124.5, 113.0, 83.8, 79.4, 61.5, 48.1, 37.6, 28.6, 25.2, 24.7, 17.0.

HRMS (ESI) Calcd for $C_{27}H_{36}BNNaO_4^+$ [M + Na]$^+$: 472.2630, Found: 472.2638.

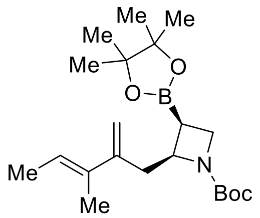

3g

***tert*-Butyl(2*S*,3*S*)-2-((*E*)-3-methyl-2-methylenepent-3-en-1-yl)-3-(4,4,5,5-tetramethyl-1,3,2-dioxaborolan-2-yl)azetidine-1-carboxylate (3g)** was prepared according to the General Procedure C as a yellow oil

(chromatography eluent: *n*-hexane/EtOAc = 10:1) in 68% yield (102.6 mg, >99% ee, >20:1 rr).

$[\alpha]_D^{25}$: +2.4 (c = 2.6, $CHCl_3$). HPLC analysis of the product: Daicel CHIRALPAK® IC-3 column; 10% *i*-PrOH in *n*-hexane; 1.0 mL/min; retention times: 5.6 min (major), 6.2 min (minor).

^{1}H NMR (400 MHz, $CDCl_3$) δ 5.31 – 5.26 (m, 1H), 4.91 (s, 1H), 4.74 (s, 1H), 4.46 – 4.33 (m, 1H), 3.91 (dd, *J* = 9.8, 7.8 Hz, 1H), 3.75 – 3.71 (m, 1H), 2.81 – 2.69 (m, 2H), 2.09 (td, *J* = 9.5, 6.1 Hz, 1H), 1.76 (s, 3H), 1.58 – 1.56 (m, 3H), 1.41 (s, 9H), 1.25 (d, *J* = 12.0 Hz, 12H).

^{13}C NMR (101 MHz, $CDCl_3$) δ 156.9, 145.7, 137.9, 121.3, 113.6, 83.7, 79.3, 61.5, 48.7, 38.0, 28.6, 25.5, 24.7, 23.4, 16.9, 14.8.

HRMS (ESI) Calcd for $C_{21}H_{36}BNNaO_4^+$ [M + Na]$^+$: 400.2630, Found: 400.2636.

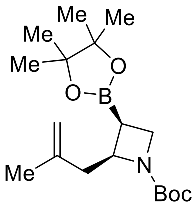

3h

***tert*-Butyl(2*S*,3*S*)-2-(2-methylallyl)-3-(4,4,5,5-tetramethyl-1,3,2-dioxaborolan-2-yl)azetidine-1-carboxylate (3h)** was prepared according to the General Procedure C as a yellow oil (chromatography eluent: *n*-hexane/EtOAc = 10:1) in 73% yield (98.5 mg, >99% ee, >20:1 rr).

$[\alpha]_D^{25}$: +46.2 (c = 2.5, $CHCl_3$). HPLC analysis of the product: Daicel CHIRALPAK® AY-3 column; 10% *i*-PrOH in *n*-hexane; 1.0 mL/min; retention times: 6.6 min (major), 5.1 min (minor). The ee was determined by its benzyloxy derivative.

^{1}H NMR (400 MHz, $CDCl_3$) δ 4.70 (s, 1H), 4.54 – 4.49 (m, 2H), 3.89 (dd, *J* = 10.0, 7.9 Hz, 1H), 3.80 (dd, *J* = 7.7, 6.5 Hz, 1H), 2.61 – 2.47 (m, 2H), 2.22 (td, *J* = 9.7, 6.4 Hz, 1H), 1.70 (s, 3H), 1.40 (s, 9H), 1.21 (d, *J* = 8.1 Hz, 12H).

^{13}C NMR (101 MHz, $CDCl_3$) δ 156.7, 142.6, 110.7, 83.7, 79.2, 61.3, 48.3, 40.9, 28.5,

25.1, 24.8, 23.7, 17.2.

HRMS (ESI) Calcd for $C_{18}H_{32}BNNaO_4^+$ [M + Na]$^+$: 360.2317, Found: 360.2323.

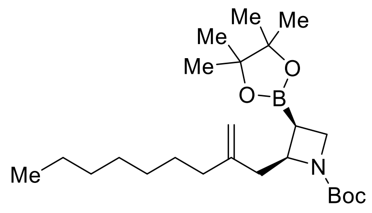

3i

***tert*-Butyl(2*S*,3*S*)-2-(2-methylenenonyl)-3-(4,4,5,5-tetramethyl-1,3,2-dioxaborolan-2-yl)azetidine-1-carboxylate (3i)** was prepared according to the General Procedure C as a yellow oil (chromatography eluent: *n*-hexane/EtOAc = 10:1) in 78% yield (131.4 mg, >99% ee, >20:1 rr).

$[\alpha]_D^{25}$: +43.5 (c = 4.2, $CHCl_3$). HPLC analysis of the product: Daicel CHIRALPAK® IC3 column; 10% *i*-PrOH in *n*-hexane; 1.0 mL/min; retention times: 6.1 min (major), 5.5 min (minor). The ee was determined by its benzyloxy derivative.

^{1}H NMR (400 MHz, $CDCl_3$) δ 4.68 (s, 1H), 4.53 – 4.46 (m, 2H), 3.87 (dd, *J* = 9.9, 7.9 Hz, 1H), 3.78 – 3.74 (m, 1H), 2.63 – 2.45 (m, 2H), 2.18 (td, *J* = 9.7, 6.3 Hz, 1H), 1.97 – 1.93 (m, 2H), 1.38 (s, 9H), 1.21 – 1.18 (m, 20H), 0.83 (t, J = 6.8 Hz, 3H).

^{13}C NMR (101 MHz, $CDCl_3$) δ 156.5, 146.5, 109.2, 83.5, 79.1, 61.3, 48.2, 38.8, 37.3, 29.3, 29.1, 28.4, 27.6, 25.1, 24.6, 22.6, 16.9, 14.0.

HRMS (ESI) Calcd for $C_{24}H_{44}BNNaO_4^+$ [M + Na]$^+$: 444.3256, Found: 444.3261.

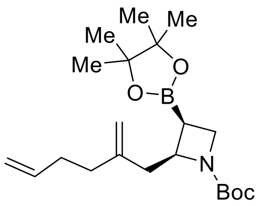

3j

***tert*-Butyl(2*S*,3*S*)-2-(2-methylenehex-5-en-1-yl)-3-(4,4,5,5-tetramethyl-1,3,2-dioxaborolan-2-yl)azetidine-1-carboxylate (3j)** was prepared according to the

General Procedure C as a yellow oil (chromatography eluent: *n*-hexane/EtOAc = 10:1) in 95% yield (143.3 mg, >99% ee, >20:1 rr).

$[\alpha]_D^{25}$: +21.7 (c = 3.6, $CHCl_3$). HPLC analysis of the product: Daicel CHIRALPAK® OZ-3 column; 10% *i*-PrOH in *n*-hexane; 1.0 mL/min; retention times: 13.4 min (major), 18.2 min (minor). The ee was determined by its tosylate derivative.

^{1}H NMR (400 MHz, $CDCl_3$) δ 5.78 (ddt, *J* = 16.8, 10.2, 6.4 Hz, 1H), 5.01 – 4.90 (m, 2H), 4.73 (s, 1H), 4.55 – 4.45 (m, 2H), 3.89 (dd, *J* = 9.9, 7.9 Hz, 1H), 3.78 (dd, *J* = 7.8, 6.3 Hz, 1H), 2.65 – 2.48 (m, 2H), 2.24 – 2.05 (m, 5H), 1.39 (s, 9H), 1.20 (d, *J* = 8.8 Hz, 12H).

^{13}C NMR (101 MHz, $CDCl_3$) δ 156.6, 145.8, 138.4, 114.6, 109.9, 83.7, 79.2, 61.4, 48.3, 39.1, 36.6, 32.0, 28.5, 25.2, 24.8, 17.2.

HRMS (ESI) Calcd for $C_{21}H_{36}BNNaO_4^+$ [M + Na]$^+$: 400.2630, Found: 400.2637.

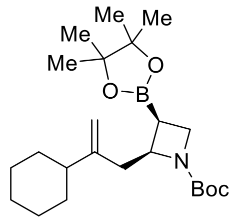

3k

***tert*-Butyl(2*S*,3*S*)-2-(2-cyclohexylallyl)-3-(4,4,5,5-tetramethyl-1,3,2-dioxaborolan-2-yl)azetidine-1-carboxylate (3k)** was prepared according to the General Procedure C as a yellow oil (chromatography eluent: *n*-hexane/EtOAc = 10:1) in 91% yield (147.4 mg, >99% ee, >20:1 rr).

$[\alpha]_D^{25}$: +39.5 (c = 3.7, $CHCl_3$). HPLC analysis of the product: Daicel CHIRALPAK® OX-3 column; 5% *i*-PrOH in *n*-hexane; 1.0 mL/min; retention times: 5.2 min (major), 7.1 min (minor).

^{1}H NMR (400 MHz, $CDCl_3$) δ 4.69 (s, 1H), 4.54 – 4.43 (m, 2H), 3.90 (dd, *J* = 9.8, 7.9 Hz, 1H), 3.78 – 3.74 (m, 1H), 2.69 – 2.51 (m, 2H), 2.19 (td, *J* = 9.6, 6.2 Hz, 1H), 1.81 – 1.61 (m, 6H), 1.40 (s, 9H), 1.21 – 1.03 (m, 18H).

^{13}C NMR (101 MHz, $CDCl_3$) δ 156.7, 151.7, 107.3, 83.7, 79.1, 61.9, 48.5, 45.1, 37.3, 32.2, 28.6, 26.8, 26.8, 26.5, 25.3, 24.7, 17.4.

HRMS (ESI) Calcd for $C_{23}H_{40}BNNaO_4^+$ [M + Na]$^+$: 428.2943, Found: 428.2949.

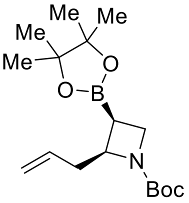

3l

***tert*-Butyl(2*S*,3*S*)-2-allyl-3-(4,4,5,5-tetramethyl-1,3,2-dioxaborolan-2-yl)azetidine-1-carboxylate (3l)** was prepared according to the General Procedure C as a yellow oil (chromatography eluent: *n*-hexane/EtOAc = 10:1) in 95% yield (122.7 mg, >99% ee, >20:1 rr).

$[\alpha]_D^{25}$: +41.5 (c = 3.1, $CHCl_3$). HPLC analysis of the product: Daicel CHIRALPAK® IC3 column; 10% *i*-PrOH in *n*-hexane; 1.0 mL/min; retention times: 8.5 min (major), 7.8 min (minor). The ee was determined by its benzyloxy derivative.

^{1}H NMR (400 MHz, $CDCl_3$) δ 5.81 (ddt, *J* = 17.0, 10.3, 6.7 Hz, 1H), 5.05 – 4.99 (m, 2H), 4.33 (q, *J* = 8.8 Hz, 1H), 3.88 – 3.79 (m, 2H), 2.62 – 2.47 (m, 2H), 2.18 (td, *J* = 9.7, 6.8 Hz, 1H), 1.39 (s, 9H), 1.23 (d, *J* = 4.1 Hz, 12H).

^{13}C NMR (101 MHz, $CDCl_3$) δ 156.5, 134.7, 116.9, 83.8, 79.2, 62.8, 48.2, 38.3, 28.5, 25.1, 25.0, 16.7.

HRMS (ESI) Calcd for $C_{17}H_{30}BNNaO_4^+$ [M + Na]$^+$: 346.2160, Found: 346.2170.

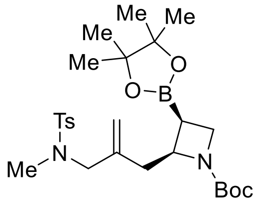

3m

***tert*-Butyl(2*S*,3*S*)-2-(2-(((*N*,4-dimethylphenyl)sulfonamido)methyl)allyl)-3-**

(4,4,5,5-tetramethyl-1,3,2-dioxaborolan-2-yl)azetidine-1-carboxylate (3m) was prepared according to the General Procedure C as a yellow oil (chromatography eluent: *n*-hexane/EtOAc = 10:1) in 76% yield (157.2 mg, >99% ee, >20:1 rr).

$[\alpha]_D^{25}$: +25.5 (c = 4.7, $CHCl_3$). HPLC analysis of the product: Daicel CHIRALPAK® IC-3 column; 20% *i*-PrOH in *n*-hexane; 1.0 mL/min; retention times: 6.5 min (major), 7.7 min (minor).

^{1}H NMR (400 MHz, $CDCl_3$) δ 7.63 (d, *J* = 8.3 Hz, 2H), 7.29 (d, *J* = 8.0 Hz, 2H), 4.93 (s, 1H), 4.82 (s, 1H), 4.54 (td, *J* = 8.7, 5.9 Hz, 1H), 3.89 (dd, *J* = 10.1, 7.8 Hz, 1H), 3.80 (t, *J* = 7.1 Hz, 1H), 3.68 (d, *J* = 14.1 Hz, 1H), 3.29 (d, *J* = 14.2 Hz, 1H), 2.63 – 2.48 (m, 5H), 2.40 (s, 3H), 2.23 (td, *J* = 9.7, 6.4 Hz, 1H), 1.38 (s, 9H), 1.21 (d, *J* = 10.0 Hz, 12H).

^{13}C NMR (101 MHz, $CDCl_3$) δ 156.7, 143.4, 140.8, 134.2, 129.7, 127.6, 114.2, 83.8, 79.3, 61.0, 55.8, 48.7, 37.3, 34.3, 28.5, 25.1, 24.8, 21.6, 17.2.

HRMS (ESI) Calcd for $C_{26}H_{41}BN_2NaO_6S^+$ $[M + Na]^+$: 543.2671, Found: 543.2677.

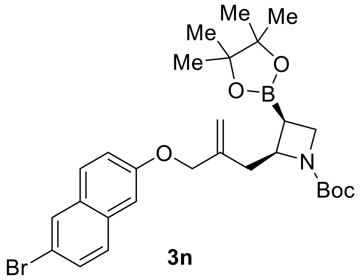

3n

***tert*-Butyl(2*S*,3*S*)-2-(2-(((6-bromonaphthalen-2-yl)oxy)methyl)allyl)-3-(4,4,5,5-tetramethyl-1,3,2-dioxaborolan-2-yl)azetidine-1-carboxylate (3n)** was prepared according to the General Procedure C as a yellow oil (chromatography eluent: *n*-hexane/EtOAc = 10:1) in 93% yield (207.2 mg, >99% ee, >20:1 rr).

$[\alpha]_D^{25}$: +21.2 (c = 5.2, $CHCl_3$). HPLC analysis of the product: Daicel CHIRALPAK® IC3 column; 10% *i*-PrOH in *n*-hexane; 1.0 mL/min; retention times: 8.3 min (major), 10.4 min (minor).

^{1}**H NMR** (400 MHz, $CDCl_3$) δ 7.88 (d, *J* = 1.7 Hz, 1H), 7.59 (dd, *J* = 21.1, 8.9 Hz, 2H), 7.47 (dd, *J* = 8.7, 2.0 Hz, 1H), 7.17 (dd, *J* = 9.0, 2.5 Hz, 1H), 7.09 (d, *J* = 2.3 Hz, 1H), 5.23 (s, 1H), 4.94 (s, 1H), 4.63 – 4.52 (m, 3H), 43.97 – 3.85 (m, 2H), 2.74 (h, *J* = 9.5, 8.5 Hz, 2H), 2.30 (td, *J* = 9.7, 6.5 Hz, 1H), 1.41 (s, 9H), 1.23 (d, *J* = 8.4 Hz, 12H).

^{13}**C NMR** (101 MHz, $CDCl_3$) δ 157.0, 156.7, 141.5, 133.1, 130.1, 129.7, 129.6, 128.5, 128.5, 120.1, 117.1, 113.0, 107.1, 83.8, 79.4, 71.2, 61.4, 48.4, 37.1, 28.5, 25.1, 24.8, 17.4.

HRMS (ESI) Calcd for $C_{28}H_{37}BBrNNaO_5^+$ $[M + Na]^+$: 580.1840, Found: 580.1849.

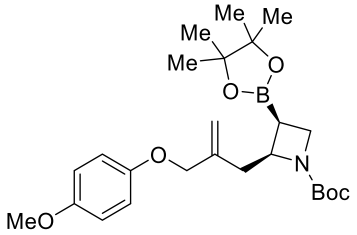

3o

***tert*-Butyl(2*S*,3*S*)-2-(2-((4-methoxyphenoxy)methyl)allyl)-3-(4,4,5,5-tetramethyl-1,3,2-dioxaborolan-2-yl)azetidine-1-carboxylate (3o)** was prepared according to the General Procedure C as a yellow oil (chromatography eluent: *n*-hexane/EtOAc = 10:1) in 65% yield (119.3 mg, >99% ee, >20:1 rr).

$[\alpha]_D^{25}$: +39.4 (c = 3.0, $CHCl_3$). HPLC analysis of the product: Daicel CHIRALPAK® IC-3 column; 10% *i*-PrOH in *n*-hexane; 1.0 mL/min; retention times: 11.6 min (major), 13.0 min (minor).

^{1}**H NMR** (400 MHz, $CDCl_3$) δ 6.84 – 6.78 (m, 4H), 5.15 (s, 1H), 4.86 (s, 1H), 4.57 (q, *J* = 8.7 Hz, 1H), 4.42 – 4.34 (m, 2H), 3.92 (dd, *J* = 9.9, 8.0 Hz, 1H), 3.83 (dd, *J* = 7.7, 6.6 Hz, 1H), 3.74 (s, 3H), 2.67 (h, *J* = 9.6, 8.7 Hz, 2H), 2.27 (td, *J* = 9.7, 6.4 Hz, 1H), 1.40 (s, 9H), 1.22 (d, *J* = 8.3 Hz, 12H).

^{13}**C NMR** (101 MHz, $CDCl_3$) δ 156.7, 153.9, 153.0, 142.1, 115.8, 114.6, 112.4, 83.8, 79.4, 71.8, 61.4, 55.8, 48.3, 36.9, 28.5, 25.2, 24.8, 17.3.

HRMS (ESI) Calcd for $C_{25}H_{38}BNNaO_6^+$ $[M + Na]^+$: 482.2684, Found: 482.2696.

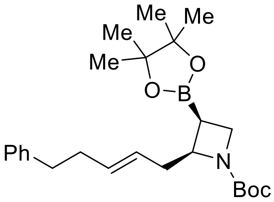

5a

***tert*-Butyl(2*S*,3*S*)-2-((*E*)-5-phenylpent-2-en-1-yl)-3-(4,4,5,5-tetramethyl-1,3,2-dioxaborolan-2-yl)azetidine-1-carboxylate (5a)** was prepared according to the General Procedure C (with 10 mol% of CuBr and 12 mol% of **L1**) as a yellow oil (chromatography eluent: *n*-hexane/EtOAc = 10:1) in 82% yield (140.1 mg, >99% ee, *E*/*Z* = 10:1).

$[\alpha]_D^{25}$: +38.0 (c = 3.4, $CHCl_3$). HPLC analysis of the product: Daicel CHIRALPAK® IC3 column; 10% *i*-PrOH in *n*-hexane; 1.0 mL/min; retention times: 6.5 min (major), 7.8 min (minor).

^{1}H NMR (400 MHz, $CDCl_3$) δ 7.28 – 7.25 (m, 2H), 7.18 – 7.15 (m, 3H), 5.50 (q, *J* = 5.1 Hz, 2H), 4.30 (q, *J* = 8.8 Hz, 1H), 3.90 – 3.81 (m, 2H), 2.68 – 2.64 (m, 2H), 2.60 – 2.35 (m, 2H), 2.33 – 2.27 (m, 2H), 2.19 (td, *J* = 9.7, 6.8 Hz, 1H), 1.43 (s, 9H), 1.25 (d, *J* = 4.1 Hz, 12H).

^{13}C NMR (101 MHz, $CDCl_3$) δ 156.5, 142.1, 131.9, 128.5, 128.3, 126.4, 125.8, 83.8, 79.1, 63.3, 48.1, 37.0, 36.0, 34.8, 28.6, 25.1, 17.0.

HRMS (ESI) Calcd for $C_{25}H_{38}BNNaO_4^+$ [M + Na]$^+$: 450.2786, Found: 450.2793.

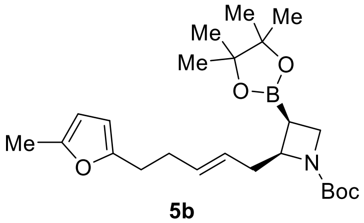

5b

***tert*-Butyl(2*S*,3*S*)-2-((*E*)-5-(5-methylfuran-2-yl)pent-2-en-1-yl)-3-(4,4,5,5-tetramethyl-1,3,2-dioxaborolan-2-yl)azetidine-1-carboxylate (5b)** was prepared according to the General Procedure C (with 10 mol% of CuBr and 12 mol% of **L1**) as a yellow oil (chromatography eluent: *n*-hexane/EtOAc = 10:1) in

95% yield (198.4 mg, >99% ee, *E*/*Z* = 8:1).

[α]$_D^{25}$: +1.8 (c = 1.0, $CHCl_3$). HPLC analysis of the product: Daicel CHIRALPAK® IC-3 column; 10% *i*-PrOH in *n*-hexane; 1.0 mL/min; retention times: 6.2 min (major), 7.2 min (minor).

^{1}H NMR (400 MHz, $CDCl_3$) δ 5.84 – 5.81 (m, 2H), 5.52 – 5.47 (m, 2H), 4.30 (q, *J* = 8.8 Hz, 1H), 3.90 – 3.81 (m, 2H), 2.63 – 2.59 (m, 2H), 2.51 – 2.16 (m, 8H), 1.43 (s, 9H), 1.26 (d, *J* = 3.7 Hz, 12H).

^{13}C NMR (101 MHz, $CDCl_3$) δ 156.5, 154.0, 150.2, 131.6, 126.5, 105.8, 105.5, 83.8, 79.1, 63.2, 48.3, 37.0, 31.5, 28.6, 28.1, 25.0, 16.8, 13.6.

HRMS (ESI) Calcd for $C_{24}H_{38}BNNaO_5^+$ [M + Na]$^+$: 454.2735, Found: 454.2742.

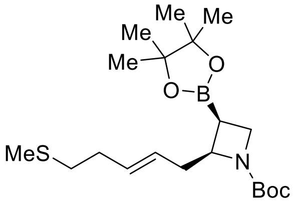

5c

***tert*-Butyl(2*S*,3*S*)-2-((*E*)-5-(methylthio)pent-2-en-1-yl)-3-(4,4,5,5-tetramethyl-1,3,2-dioxaborolan-2-yl)azetidine-1-carboxylate (5c)** was prepared according to the General Procedure C (with 10 mol% of CuBr and 12 mol% of **L1**) as a yellow oil (chromatography eluent: *n*-hexane/EtOAc = 10:1) in 95% yield (150.9 mg, >99% ee, *E*/*Z* = 11:1).

[α]$_D^{25}$: +3.3 (c = 1.7, $CHCl_3$). HPLC analysis of the product: Daicel CHIRALPAK® IC3 column; 10% *i*-PrOH in *n*-hexane; 1.0 mL/min; retention times: 7.7 min (major), 9.2 min (minor).

^{1}H NMR (400 MHz, $CDCl_3$) δ 5.54 – 5.42 (m, 2H), 4.32 – 4.26 (m, 1H), 3.88 – 3.79 (m, 2H), 2.58 – 2.42 (m, 4H), 2.28 – 2.15 (m, 3H), 2.07 (s, 9H), 1.24 (d, *J* = 3.7 Hz, 12H).

^{13}C NMR (101 MHz, $CDCl_3$) δ 156.5, 130.6, 127.5, 83.8, 79.1, 63.1, 48.1, 37.1, 34.2, 32.8, 28.6, 25.1, 16.9, 15.7.

HRMS (ESI) Calcd for $C_{20}H_{36}BNNaO_4S^+$ [M + Na]$^+$: 420.2350, Found: 420.2357.

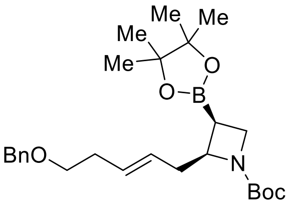

5d

***tert*-Butyl(2*S*,3*S*)-2-((*E*)-5-(benzyloxy)pent-2-en-1-yl)-3-(4,4,5,5-tetramethyl-1,3,2-dioxaborolan-2-yl)azetidine-1-carboxylate (5d)** was prepared according to the General Procedure C (with 10 mol% of CuBr and 12 mol% of **L1**) as a yellow oil (chromatography eluent: *n*-hexane/EtOAc = 10:1) in 96% yield (175.5 mg, >99% ee, *E*/*Z* = 11:1).

$[\alpha]_D^{25}$: +18.6 (c = 4.7, $CHCl_3$). HPLC analysis of the product: Daicel CHIRALPAK® IE-3 column; 10% *i*-PrOH in *n*-hexane; 1.0 mL/min; retention times: 8.5 min (major), 9.0 min (minor).

^{1}H NMR (400 MHz, $CDCl_3$) δ 7.33 – 7.24 (m, 5H), 5.58 – 5.43 (m, 1H), 4.50 (s, 1H), 4.34 – 4.28 (m, 1H), 3.90 – 3.81 (m, 1H), 3.47 (t, *J* = 7.0 Hz, 2H), 2.61 – 2.45 (m, 2H), 2.32 (q, *J* = 6.6 Hz, 2H), 2.20 (td, *J* = 9.7, 6.8 Hz, 1H), 1.42 (s, 9H), 1.25 (d, *J* = 4.5 Hz, 12H).

^{13}C NMR (101 MHz, $CDCl_3$) δ 156.4, 138.5, 128.7, 128.4, 128.0, 127.7, 127.5, 83.7, 79.0, 72.9, 70.1, 63.1, 48.0, 37.1, 33.4, 28.5, 25.0, 16.9.

HRMS (ESI) Calcd for $C_{26}H_{40}BNNaO_5^+$ [M + Na]$^+$: 480.2892, Found: 480.2899.

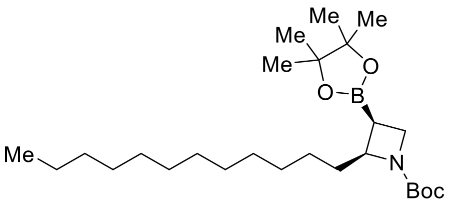

5e'

***tert*-Butyl(2*S*,3*S*)-2-dodecyl-3-(4,4,5,5-tetramethyl-1,3,2-dioxaborolan-2-yl) azetidine-1-carboxylate (5e′)** was prepared according to the General Procedure C (with 10 mol% of CuBr and 12 mol% of **L1**) as a yellow oil (chromatography eluent: *n*-hexane/EtOAc = 10:1) in 81% yield (146.1 mg, >99% ee, *E*/*Z* = 2:1). Since

the obtained product was an *E*/*Z* mixture, hydrogenation of compound **5e** yielded the pure product **5e'**.

[α]$_D^{25}$: +22.7 (c = 3.0, $CHCl_3$). HPLC analysis of the product (**5e**): Daicel CHIRALPAK® IC-3 column; 5% *i*-PrOH in *n*-hexane; 1.0 mL/min; retention times: 6.2 min (major), 7.5 min (minor).

^{1}H NMR (400 MHz, $CDCl_3$) δ 4.25 (q, *J* = 8.7 Hz, 1H), 3.86 – 3.80 (m, 2H), 2.16 (td, *J* = 9.5, 7.3 Hz, 1H), 1.90 – 1.75 (m, 1H), 1.68 – 1.61 (m, 1H), 1.40 (s, 9H), 1.24 – 1.22 (m, 32H), 0.85 (t, *J* = 6.8 Hz, 3H).

^{13}C NMR (101 MHz, $CDCl_3$) δ 156.6, 83.6, 78.9, 63.5, 48.0, 34.6, 31.9, 29.7, 29.63, 29.62, 29.3, 28.5, 25.5, 24.9, 22.7, 17.1, 14.1.

HRMS (ESI) Calcd for $C_{26}H_{50}BNNaO_4^+$ [M + Na]$^+$: 474.3725, Found: 474.3735.

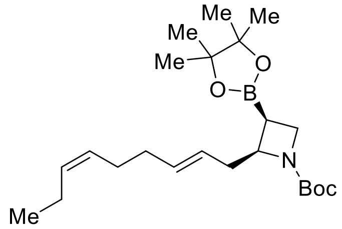

5f

***tert*-Butyl(2*S*,3*S*)-2-((2*E*,6*Z*)-nona-2,6-dien-1-yl)-3-(4,4,5,5-tetramethyl-1,3,2-dioxaborolan-2-yl)azetidine-1-carboxylate (5f)** was prepared according to the General Procedure C (with 10 mol% of CuBr and 12 mol% of **L1**) as a yellow oil (chromatography eluent: *n*-hexane/EtOAc = 10:1) in 94% yield (152.3 mg, >99% ee, *E*/*Z* > 20:1).

[α]$_D^{25}$: +24.7 (c = 4.5, $CHCl_3$). HPLC analysis of the product: Daicel CHIRALPAK® AY-3 column; 10% *i*-PrOH in *n*-hexane; 1.0 mL/min; retention times: 6.7 min (major), 5.0 min (minor). The ee was determined by its benzyloxy derivative.

^{1}H NMR (400 MHz, $CDCl_3$) δ 5.42 (t, *J* = 3.5 Hz, 2H), 5.36 – 5.24 (m, 2H), 4.27 (q, *J* = 8.8 Hz, 1H), 3.86 – 3.77 (m, 2H), 2.56 – 2.41 (m, 2H), 2.17 (td, *J* = 9.7, 6.8 Hz, 1H), 2.05 – 1.95 (m, 6H), 1.39 (s, 9H), 1.22 (s, 12H), 0.91 (t, *J* = 7.5 Hz, 3H).

^{13}**C NMR** (101 MHz, $CDCl_3$) δ 156.5, 132.3, 131.9, 128.5, 126.0, 83.7, 79.0, 63.1, 48.0, 36.9, 33.0, 28.5, 27.1, 25.0, 20.6, 16.8, 14.4.

HRMS (ESI) Calcd for $C_{23}H_{40}BNNaO_4^+$ [M + Na]$^+$: 428.2943, Found: 428.2947.

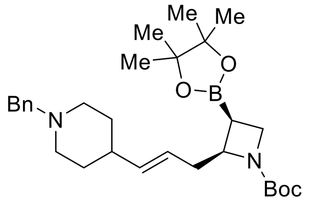

5g

***tert*-Butyl(2*S*,3*S*)-2-((*E*)-3-(1-benzylpiperidin-4-yl)allyl)-3-(4,4,5,5-tetramethyl-1,3,2-dioxaborolan-2-yl)azetidine-1-carboxylate (5g)** was prepared according to the General Procedure C (with 10 mol% of CuBr and 12 mol% of **L1**) as a yellow oil (chromatography eluent: *n*-hexane/EtOAc = 10:1) in 85% yield (161.2 mg, >99% ee, *E*/*Z* >20: 1).

$[\alpha]_D^{25}$: +25.5 (c = 0.5, $CHCl_3$). HPLC analysis of the product: Daicel CHIRALPAK® IC3 column; 10% *i*-PrOH in *n*-hexane; 1.0 mL/min; retention times: 7.8 min (major), 10.2 min (minor).

^{1}**H NMR** (400 MHz, $CDCl_3$) δ 7.32 – 7.22 (m, 5H), 5.47 – 5.34 (m, 2H), 4.29 (q, *J* = 8.4 Hz, 1H), 3.89 – 3.80 (m, 2H), 3.48 (s, 2H), 2.86 (d, *J* = 11.3 Hz, 2H), 2.58 – 2.43 (m, 2H), 2.19 (td, *J* = 9.6, 6.7 Hz, 1H), 1.99 – 1.87 (m, 3H), 1.64 – 1.60 (m, 2H), 1.44 – 1.38 (m, 11H), 1.26 (d, *J* = 3.5 Hz, 12H).

^{13}**C NMR** (101 MHz, $CDCl_3$) δ 156.5, 138.6, 137.6, 129.3, 128.2, 127.0, 123.9, 83.8, 79.1, 75.1, 63.6, 53.7, 48.3, 39.2, 37.0, 32.2, 28.6, 25.1, 25.0, 16.9.

HRMS (ESI) Calcd for $C_{29}H_{46}BN_2O_4^+$ [M + H]$^+$: 497.3545, Found: 497.3552.

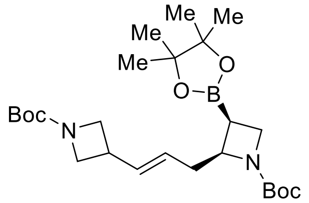

5h

***tert*-Butyl(2*S*,3*S*)-2-((*E*)-3-(1-(tert-butoxycarbonyl)azetidin-3-yl)allyl)-3-**

(4,4,5,5-tetramethyl-1,3,2-dioxaborolan-2-yl)azetidine-1-carboxylate (5h) was prepared according to the General Procedure C (with 10 mol% of CuBr and 12 mol% of **L1**) as a yellow oil (chromatography eluent: *n*-hexane/EtOAc = 10:1) in 94% yield (179.7 mg, >99% ee, *E*/*Z* = 11:1).

$[\alpha]_D^{25}$: +16.2 (c = 4.5, $CHCl_3$). HPLC analysis of the product: Daicel CHIRALPAK® IC3 column; 5% *i*-PrOH in *n*-hexane; 1.0 mL/min; retention times: 19.8 min (major), 21.9 min (minor).

^{1}H NMR (400 MHz, $CDCl_3$) δ 5.60 – 5.45 (m, 2H), 4.26 (q, *J* = 8.5 Hz, 1H), 3.99 (t, *J* = 8.4 Hz, 1H), 3.85 – 3.76 (m, 2H), 3.64 (ddd, *J* = 8.5, 6.0, 2.7 Hz, 2H), 3.13 – 3.03 (m, 1H), 2.57 – 2.40 (m, 2H), 2.16 (td, *J* = 9.7, 6.8 Hz, 1H), 1.37 (d, *J* = 3.3 Hz, 18H), 1.20 (d, *J* = 4.5 Hz, 12H).

^{13}C NMR (101 MHz, $CDCl_3$) δ 156.4, 156.3, 132.6, 127.8, 83.8, 79.2, 79.1, 62.7, 54.8, 48.0, 36.9, 31.5, 28.5, 28.4, 25.01, 24.98, 17.0.

HRMS (ESI) Calcd for $C_{25}H_{43}BN_2NaO_6^+$ $[M + Na]^+$: 501.3106, Found: 501.3112.

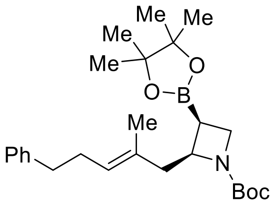

5i

***tert*-Butyl(2*S*,3*S*)-2-((*E*)-3-methyl-5-phenylpent-2-en-1-yl)-3-(4,4,5,5-tetramethyl-1,3,2-dioxaborolan-2-yl)azetidine-1-carboxylate (5i)** was prepared according to the General Procedure C (with 10 mol% of CuBr and 12 mol% of **L1**) as a yellow oil (chromatography eluent: *n*-hexane/EtOAc = 10:1) in 87% yield (153.5 mg, >99% ee, *E*/*Z* = 12:1).

$[\alpha]_D^{25}$: +33.5 (c = 3.7, $CHCl_3$). HPLC analysis of the product: Daicel CHIRALPAK® IC-3 column; 10% *i*-PrOH in *n*-hexane; 1.0 mL/min; retention times: 6.2 min (major), 7.4 min (minor).

^{1}H NMR (400 MHz, $CDCl_3$) δ 7.29 – 7.25 (m, 2H), 7.19 – 7.15 (m, 3H), 5.05 (t, *J* = 6.5 Hz, 1H), 4.53 – 4.47 (m, 1H), 3.93 – 3.81 (m, 2H), 2.69 – 2.45 (m, 4H), 2.31 –

2.19 (m, 3H), 1.58 (s, 3H), 1.42 (s, 9H), 1.23 (d, *J* = 5.8 Hz, 12H).

^{13}C NMR (101 MHz, $CDCl_3$) δ 156.8, 142.4, 132.6, 128.5, 128.3, 125.8, 124.1, 83.7, 79.1, 61.4, 48.3, 42.9, 30.3, 28.6, 25.1, 24.9, 17.3, 17.0.

HRMS (ESI) Calcd for $C_{26}H_{40}BNNaO_4^+$ [M + Na]$^+$: 464.2943, Found: 464.2947.

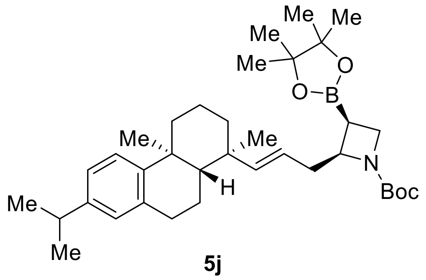

5j

***tert*-Butyl(2*S*,3*S*)-2-((E)-3-((1*S*,4a*S*,10a*S*)-7-isopropyl-1,4a-dimethyl-1,2,3,4,4a,9,10,10a-octahydrophenanthren-1-yl)allyl)-3-(4,4,5,5-tetramethyl-1,3,2-dioxaborolan-2-yl)azetidine-1-carboxylate (5j)** was prepared according to the General Procedure C (with 10 mol% of CuBr and 12 mol% of **L1**) as a yellow oil (chromatography eluent: *n*-hexane/EtOAc = 10:1) in 95% yield (126.9 mg, >20:1 dr, *E*/*Z* > 20:1).

$[\alpha]_D^{25}$: +22.7 (c = 5.2, $CHCl_3$).

^{1}H NMR (400 MHz, $CDCl_3$) δ 7.16 (d, *J* = 8.2 Hz, 1H), 6.97 (dd, *J* = 8.1, 1.7 Hz, 1H), 6.87 (s, 1H), 5.43 – 5.28 (m, 2H), 4.35 – 4.23 (m, 1H), 3.89 – 3.81 (m, 2H), 2.84 – 2.78 (m, 2H), 2.50 (tq, *J* = 14.5, 8.9, 5.7 Hz, 3H), 2.34 – 2.14 (m, 2H), 1.78 -1.70 (m, 2H), 1.63 – 1.59 (m, 2H), 1.45 – 1.37 (m, 15H), 1.26 (s, 12H), 1.22 (s, 3H), 1.20 (s, 6H), 1.05 (s, 3H), 0.99 – 0.95 (m, 1H), 0.90 - 0.81 (m, 1H).

^{13}C NMR (101 MHz, $CDCl_3$) δ 156.4, 147.7, 146.3, 145.5, 135.1, 127.0, 124.2, 123.8, 121.6, 83.8, 79.0, 63.5, 49.8, 48.4, 40.3, 39.7, 38.6, 37.3, 37.0, 33.5, 30.4, 28.6, 25.5, 25.1, 24.09, 24.07, 19.8, 19.1, 16.8.

HRMS (ESI) Calcd for $C_{36}H_{56}BNNaO_4^+$ [M + Na]$^+$: 600.4195, Found: 600.4207.

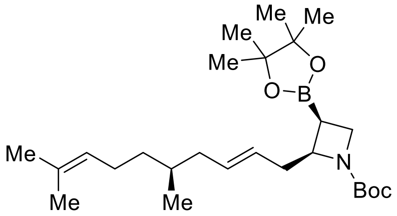

5k

***tert*-Butyl(2*S*,3*S*)-2-((*S*,*E*)-4,8-dimethylnona-2,7-dien-1-yl)-3-(4,4,5,5-tetramethyl-1,3,2-dioxaborolan-2-yl)azetidine-1-carboxylate (5k)** was prepared according to the General Procedure C (with 10 mol% of CuBr and 12 mol% of **L1**) as a yellow oil (chromatography eluent: *n*-hexane/EtOAc = 10:1) in 75% yield (134.1 mg, >20:1 dr, *E*/*Z* >20:1).

$[\alpha]_D^{25}$: +28.2 (c = 3.3, $CHCl_3$).

^{1}H NMR (400 MHz, $CDCl_3$) δ 5.45 – 5.35 (m, 1H), 5.08 (t, *J* = 7.1 Hz, 1H), 4.31 – 4.22 (m, 1H), 3.87 – 3.79 (m, 2H), 2.58 – 2.43 (m, 2H), 2.17 (td, *J* = 9.7, 6.8 Hz, 1H), 1.99 – 1.89 (m, 2H), 1.82 – 1.76 (m, 1H), 1.65 (s, 3H), 1.57 (s, 3H), 1.40 (s, 9H), 1.35 – 1.27 (m, 1H), 1.24 (s, 12H), 1.13 – 1.04 (m, 1H), 0.82 (d, *J* = 6.6 Hz, 3H).

^{13}C NMR (101 MHz, $CDCl_3$) δ 156.5, 131.5, 131.1, 126.8, 125.0, 83.8, 79.1, 63.3, 48.2, 40.4, 36.8, 32.8, 28.6, 25.8, 25.7, 25.1, 19.5, 17.7, 16.9.

HRMS (ESI) Calcd for $C_{26}H_{46}BNNaO_4^+$ $[M + Na]^+$: 470.3412, Found: 470.3419.

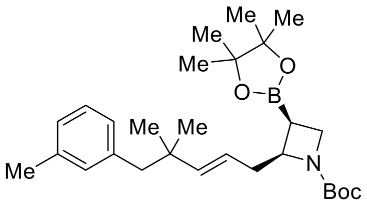

5l

***tert*-Butyl(2*S*,3*S*)-2-((*E*)-4,4-dimethyl-5-(*m*-tolyl)pent-2-en-1-yl)-3-(4,4,5,5-tetramethyl-1,3,2-dioxaborolan-2-yl)azetidine-1-carboxylate (5l)** was prepared according to the General Procedure C (with 10 mol% of CuBr and 12 mol% of **L1**) as a yellow oil (chromatography eluent: *n*-hexane/EtOAc = 10:1) in 98% yield (183.8 mg, >99% ee, *E*/*Z* = 13:1).

$[\alpha]_D^{25}$: +18.4 (c = 4.7, $CHCl_3$). HPLC analysis of the product: Daicel

CHIRALPAK® IC-3 column; 1% *i*-PrOH in *n*-hexane; 1.0 mL/min; retention times: 30.5 min (major), 50.0 min (minor).

^{1}H NMR (400 MHz, $CDCl_3$) δ 7.11 (t, *J* = 7.4 Hz, 1H), 6.98 (d, *J* = 7.5 Hz, 1H), 6.92 – 6.87 (m, 2H), 5.51 (d, *J* = 15.6 Hz, 1H), 5.30 – 5.23 (m, 1H), 4.29 – 4.23 (m, 1H), 3.89 – 3.80 (m, 2H), 2.62 – 2.44 (m, 4H), 2.31 (s, 3H), 2.17 (td, *J* = 9.6, 6.7 Hz, 1H), 1.43 (s, 9H), 1.23 (d, *J* = 5.6 Hz, 12H), 0.96 (d, *J* = 6.3 Hz, 6H).

^{13}C NMR (101 MHz, $CDCl_3$) δ 156.4, 142.6, 138.9, 136.8, 131.5, 127.8, 127.4, 126.5, 121.3, 83.7, 79.0, 63.4, 49.3, 48.0, 37.0, 28.6, 27.0, 25.1, 25.0, 21.5, 16.8.

HRMS (ESI) Calcd for $C_{28}H_{44}BNNaO_4^+$ [M + Na]$^+$: 492.3256, Found: 492.3263.

Unsuccessful examples:

We also evaluated other carbon-based electrophiles, including simple alkyl, propargyl and aryl ones. However, no desired boryl alkylation or arylation was observed. Shown below are some details.

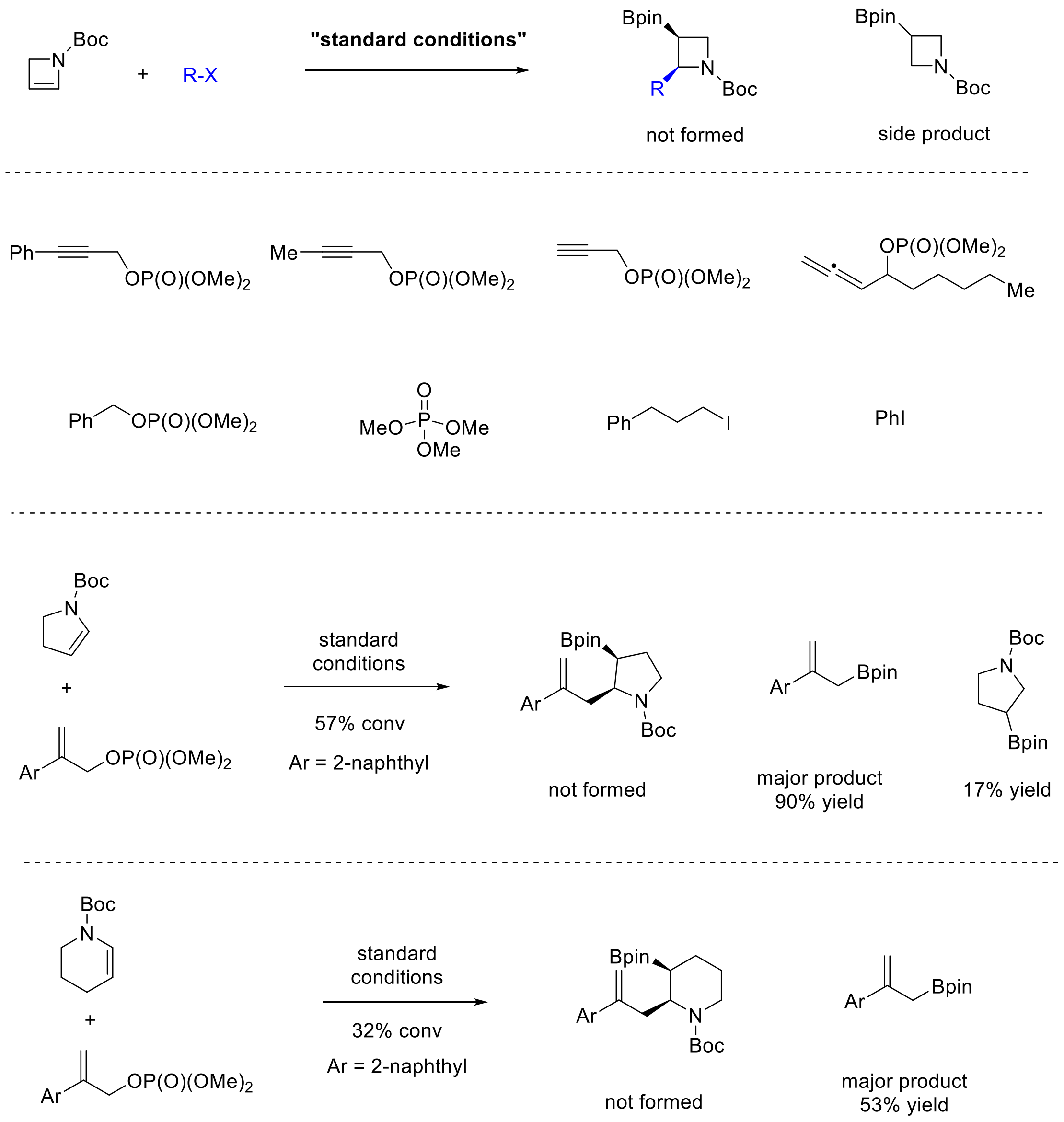

IV. Gram-Scale Reaction

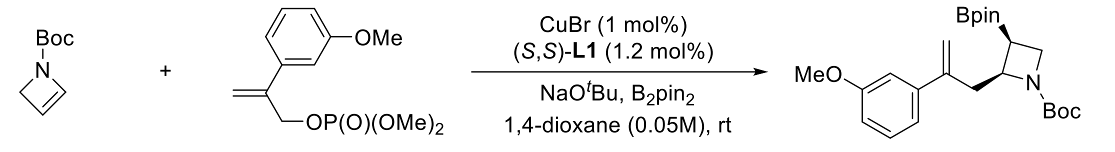

In a glove box, a mixture of CuBr (4.3 mg, 0.03 mmol, 1.0 mol%) and the chiral ligand (*S*,*S*)-**L1** (18.2 mg, 0.036 mmol, 1.2 mol%) in dry 1,4-dioxane (0.05 M, 60 mL) was stirred at room temperature for 30 min. Then, azetine **1a** (465.3 mg, 3.0 mmol, 1.0 equiv), B_2pin_2 (1.1 g, 4.5 mmol, 1.5 equiv), NaO^tBu (432.5 mg, 4.5 mmol, 1.5 equiv), and allyl phosphate **2e** (408.0 mg, 4.5 mmol, 1.5 equiv) were added sequentially. The mixture was stirred at room temperature for 10 h. Next, the mixture was concentrated *in vacuo*, and the crude product was used to determine the regioselectivity and diastereoselectivity by ^1H NMR analysis. Finally, the crude product was purified by silica gel column chromatography (chromatography eluent: *n*-hexane/EtOAc = 10:1) to afford the product **3e** (85% yield, 1.1 g, >99% ee, >20:1 rr).

V. Product Transformations

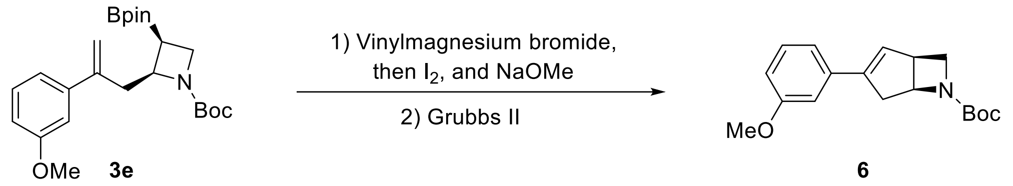

***tert*-Butyl(1*S*,5*S*)-3-(3-methoxyphenyl)-6-azabicyclo[3.2.0]hept-2-ene-6-carboxylate (6).** Under nitrogen, to a solution of **3e** (85.8 mg, 0.2 mmol, 1.0 equiv) in anhydrous THF (2 mL) was added vinyl magnesium bromide (0.8 mL, 0.8 mmol, 1.0 M in THF, 4.0 equiv) at 0 °C. The reaction mixture was stirred for 30 min at room temperature. After cooling to -78 °C, a solution of iodine (101.5 mg, 0.8 mmol, 4.0 equiv) in anhydrous THF (2 mL) was added dropwise. The mixture was stirred for 20 min before a solution of NaOMe (54.0 mg, 1.0 mmol, 5.0 equiv) in methanol (1 mL) was added. Then, the mixture was stirred at room temperature for 6 h. After **3e** was completely consumed, a saturated NH_4Cl solution (10 mL) was added. The layers were separated, and the aqueous layer was extracted with ethyl acetate (5 mL × 3). The combined organic layers were dried over Na_2SO_4, filtered, and concentrated. The residue was used for the next step without further purification.

Under nitrogen, to an over-dried flask charged with the above-prepared crude product were added DCM (4 mL) and the Grubbs 2nd generation catalyst (17.0 mg, 0.02 mmol, 10 mol%). The mixture was stirred at 60 °C for 24 h and then concentrated. The residue was purified by silica gel column chromatography (chromatography eluent: *n*-hexane/EtOAc = 10:1) to afford the desired product **6** (48.8 mg, 81% yield for two steps, >99% ee, >20:1 dr).
$[\alpha]_D^{25}$: +158.3 (c = 0.8, $CHCl_3$). HPLC analysis of the product: Daicel CHIRALPAK® AS-3 column; 10% *i*-PrOH in *n*-hexane; 1.0 mL/min; retention times: 6.2 min (major), 4.6 min (minor).

^{1}H NMR (400 MHz, $CDCl_3$) δ 7.30 – 7.26 (m, 1H), 7.11 (d, *J* = 7.7 Hz, 1H), 7.04 – 7.03 (m, 1H), 6.85 (dd, *J* = 8.2, 2.2 Hz, 1H), 6.26 (q, *J* = 2.2 Hz, 1H), 4.97 – 4.82 (m, 1H), 4.13 (t, *J* = 7.6 Hz, 1H), 3.85 (s, 3H), 3.62 (dd, *J* = 8.0, 3.1 Hz, 1H), 3.47 (tdt, *J* = 7.2, 5.5, 2.5 Hz, 1H), 3.14 – 3.02 (m, 1H), 2.93 (dd, *J* = 17.1, 6.4 Hz, 1H), 1.46 (s, 9H).

^{13}C NMR (101 MHz, $CDCl_3$) δ 159.7, 156.2, 143.8, 137.4, 125.9, 118.8, 113.4, 111.8, 79.4, 64.9, 64.0, 56.5, 55.4, 55.2, 41.4, 39.7, 28.6.

HRMS (ESI) Calcd for $C_{18}H_{23}NNaO_3^+$ [M + Na]$^+$: 324.1570, Found: 324.1573.

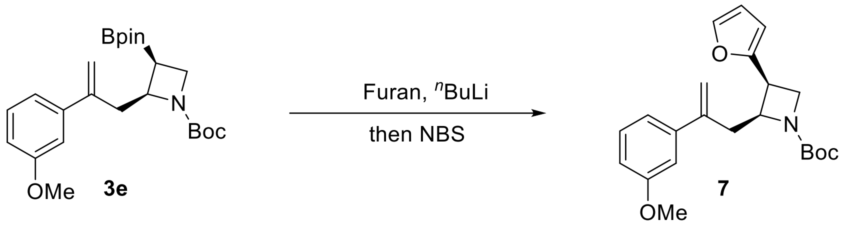

***tert*-Butyl(2*S*,3*S*)-3-(furan-2-yl)-2-(2-(3-methoxyphenyl)allyl)azetidine-1-carboxylate (7)**. To an over-dried flask charged with furan (109.0 mg, 1.6 mmol, 8 equiv) and THF (0.5 mL) was added ^{n}BuLi (0.16 mL, 1.6 mmol, 8 equiv, 2.5 M in hexane) at -78 °C. The reaction was then allowed to warm to room temperature and stir for 1 h. The mixture was then cooled to -78 °C and a solution of **3e** (85.8 mg, 0.2 mmol, 1 equiv) in THF (0.5 mL) was added dropwise. The mixture was allowed to stir at -78 °C for 1 h. A solution of NBS (284.8 mg, 1.6 mmol, 8 equiv) in THF (0.5 mL) was added dropwise. The reaction was stirred for 2 h at -78 °C. Upon completion, a saturated aqueous $Na_2S_2O_3$ solution (2 mL) was added. The mixture was allowed to warm to room temperature and the aqueous layer was extracted with ethyl acetate (5 mL × 3). The combined organic layers were dried over Na_2SO_4 and concentrated *in vacuo*. The residue was purified by silica gel column chromatography (chromatography eluent: *n*-hexane/EtOAc = 10:1) to afford the product **7** as a yellow oil (45.8 mg, 62% yield, >99% ee, >20:1 dr).

$[\alpha]_D^{25}$: -15.0 (c = 1.0, $CHCl_3$). HPLC analysis of the product: Daicel

CHIRALPAK® AY-3 column; 10% *i*-PrOH in *n*-hexane; 1.0 mL/min; retention times: 6.4 min (major), 5.8 min (minor).

^{1}H NMR (400 MHz, $CDCl_3$) δ 7.37 (d, *J* = 1.8 Hz, 1H), 7.21 (t, *J* = 7.9 Hz, 1H), 6.93 (d, *J* = 7.7 Hz, 1H), 6.87 (s, 1H), 6.79 (dd, *J* = 8.1, 2.2 Hz, 1H), 6.35 – 6.34 (m, 1H), 6.21 (d, *J* = 3.1 Hz, 1H), 5.04 (s, 1H), 4.60 – 4.49 (m, 1H), 4.45 (s, 1H), 4.15 (t, *J* = 9.0 Hz, 1H), 4.04 (dd, *J* = 8.6, 6.0 Hz, 1H), 3.84 – 3.78 (m, 4H), 3.15 (d, *J* = 13.9 Hz, 1H), 2.68 (dd, *J* = 14.8, 10.9 Hz, 1H), 1.48 (s, 9H).

^{13}C NMR (101 MHz, $CDCl_3$) δ 159.7, 156.5, 152.7, 143.4, 142.2, 141.8, 129.3, 118.8, 114.2, 112.7, 112.3, 110.6, 108.6, 79.9, 63.8, 55.3, 51.3, 36.4, 31.5, 28.6.

HRMS (ESI) Calcd for $C_{22}H_{27}NNaO_4^+$ [M + Na]$^+$: 392.1832, Found: 392.1834.

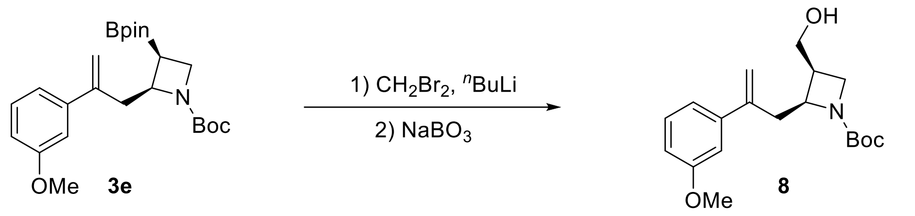

***tert*-Butyl(2*S*,3*S*)-3-(hydroxymethyl)-2-(2-(3-methoxyphenyl)allyl)azetidine-1-carboxylate (8)**. Under nitrogen, to an over-dried flask charged with **3e** (85.8 mg, 0.2 mmol, 1.0 equiv), CH_2Br_2 (35.0 uL, 0.5 mmol, 2.5 equiv), and THF (2 mL) was added ^{n}BuLi (0.16 mL, 0.4 mmol, 2.0 equiv) at -78 °C. The resulting mixture was allowed to stir at the same temperature for 30 min and then at room temperature for 5 h. After full consumption of the starting material, a saturated NH_4Cl solution (10 mL) was added. The layers were separated, and the aqueous layer was extracted with ethyl acetate (5 mL × 3). The combined organic layers were dried over Na_2SO_4, filtered, and concentrated. The residue was used for the next step without further purification.

To a mixture of the above-prepared crude product in THF/H_2O (5.0 mL, v/v = 1:1) was added $NaBO_3{\cdot}4H_2O$ (101.8 mg, 1.0 mmol, 5.0 equiv). The reaction mixture was stirred at room temperature for 2 h before it was diluted with H_2O

(5 mL) and extracted with ethyl acetate (5 mL × 3). The combined organic layers were dried over Na_2SO_4 and concentrated *in vacuo*. The residue was purified by silica gel column chromatography (chromatography eluent: *n*-hexane/EtOAc = 2:1) to afford the product **8** as a yellow oil (43.3 mg, 65% yield for two steps, >99% ee, >20:1 dr).

$[\alpha]_D^{25}$: +2.8 (c = 1.5, $CHCl_3$). HPLC analysis of the product: Daicel CHIRALPAK® AD-3 column; 10% *i*-PrOH in *n*-hexane; 1.0 mL/min; retention times: 10.3 min (major), 7.2 min (minor).

^{1}H NMR (400 MHz, $CDCl_3$) δ 7.27 – 7.23 (m, 1H), 7.02 (d, *J* = 7.8 Hz, 1H), 6.97 – 6.96 (m, 1H), 6.84 (dd, *J* = 8.0, 2.2 Hz, 1H), 5.37 (d, *J* = 1.3 Hz, 1H), 5.09 (d, *J* = 1.9 Hz, 1H), 4.51 (t, *J* = 8.4 Hz, 1H), 3.98 (dd, *J* = 11.1, 6.6 Hz, 1H), 3.91 (t, *J* = 8.6 Hz, 1H), 3.82 – 3.74 (m, 4H), 3.57 (dd, *J* = 8.7, 4.8 Hz, 1H), 3.26 (d, *J* = 15.8 Hz, 1H), 2.85 (dd, *J* = 15.8, 11.2 Hz, 1H), 2.73 – 2.64 (m, 1H), 1.46 (s, 9H), 1.23 (s, 3H).

^{13}C NMR (101 MHz, $CDCl_3$) δ 159.8, 157.1, 146.0, 142.0, 129.5, 118.6, 113.8, 113.1, 112.2, 79.8, 61.6, 61.4, 55.4, 50.0, 34.4, 28.6, 24.9.

HRMS (ESI) Calcd for $C_{19}H_{27}NNaO_4^+$ $[M + Na]^+$: 356.1832, Found: 356.1832.

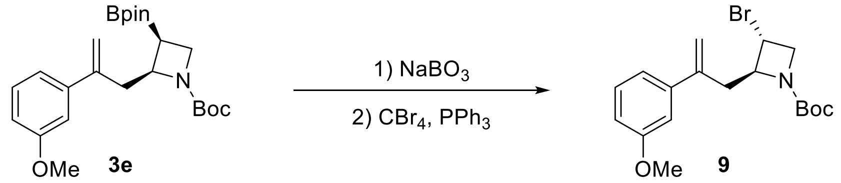

***tert*-Butyl(2*S*,3*R*)-3-bromo-2-(2-(3-methoxyphenyl)allyl)azetidine-1-carboxylate (9)**. To a solution of **3e** (85.8 mg, 0.2 mmol, 1.0 equiv) in THF/H_2O (5.0 mL, v/v = 1:1) was added $NaBO_3 \cdot 4H_2O$ (101.8 mg, 1.0 mmol, 5.0 equiv). The reaction mixture was stirred at room temperature for 2 h before it was diluted with H_2O (5 mL) and extracted with ethyl acetate (5 mL × 3). The combined organic layers were dried over Na_2SO_4, filtered, and concentrated. The residue was used for the next step without further purification.

To a mixture of the above-prepared crude product in toluene (2 mL) were added PPh_3 (157.2 mg, 0.6 mmol, 2 equiv) and CBr_4 (198.6 mg, 0.6 mmol, 2 equiv). The resulting mixture was stirred at 100 °C for 1 h. The mixture was cooled to room temperature and quenched by the addition of water. The layers were separated, and the aqueous layer was extracted with ethyl acetate (5 mL × 3). The combined organic layers were dried over Na_2SO_4 and concentrated *in vacuo*. The residue was purified by silica gel column chromatography (chromatography eluent: *n*-hexane/EtOAc = 10:1) to afford the product **9** as a yellow oil (39.6 mg, 52% yield, 99% ee, >20:1 dr).

$[\alpha]_D^{25}$: -33.5 (c = 0.7, $CHCl_3$). HPLC analysis of the product: Daicel CHIRALPAK® IJ-3 column; 10% *i*-PrOH in *n*-hexane; 1.0 mL/min; retention times: 5.6 min (major), 6.6 min (minor).

^{1}H NMR (400 MHz, $CDCl_3$) δ 7.25 (t, *J* = 7.9 Hz, 1H), 7.02 (d, *J* = 7.8 Hz, 1H), 6.97 – 6.96 (m, 1H), 6.84 (dd, *J* = 8.1, 2.2 Hz, 1H), 5.41 (d, *J* = 1.2 Hz, 1H), 5.18 (d, *J* = 1.3 Hz, 1H), 4.49 – 4.46 (m, 1H), 4.31 (ddd, *J* = 9.5, 7.0, 1.2 Hz, 1H), 4.09 – 3.99 (m, 2H), 3.82 (s, 3H), 3.31 (dd, *J* = 14.2, 3.3 Hz, 1H), 2.69 (dd, *J* = 14.4, 10.0 Hz, 1H), 1.46 (s, 3H).

^{13}C NMR (101 MHz, $CDCl_3$) δ 159.8, 155.8, 143.0, 142.1, 129.5, 118.8, 116.0, 113.2, 112.4, 80.4, 71.6, 57.2, 55.4, 40.3, 37.2, 28.5.

HRMS (ESI) Calcd for $C_{18}H_{24}BrNNaO_3^+$ $[M + Na]^+$: 404.0832, Found: 404.0834.

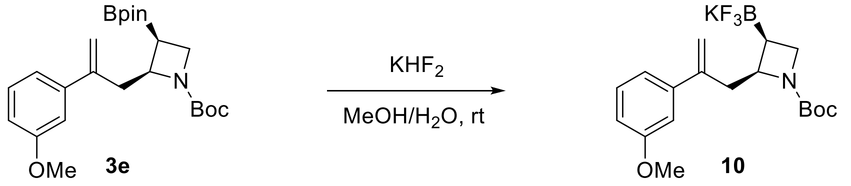

Potassium *tert*-Butyl(2*S*,3*S*)-2-(2-(3-methoxyphenyl)allyl)-3-(trifluoro-l4-boranyl)azetidine-1-carboxylate (10). To a solution of **3e** (85.8 mg, 0.2 mmol, 1.0 equiv) in $MeOH/H_2O$ (2.2 mL, v/v = 10:1) was added KHF_2 (156.2 mg, 2.0 mmol, 10.0 equiv). The mixture was stirred at room temperature for 3 h and

then concentrated *in vacuo*. Next, acetone was added, and the mixture was filtered and concentrated *in vacuo*. The residue was recrystallized from *n*-hexane and Et_2O to afford the desired product **10** as a colorless solid (77.7 mg, 95% yield, >20:1 dr).

$[\alpha]_D^{25}$: +37.2 (c = 2.0, $CHCl_3$).

^{1}H NMR (400 MHz, acetone-*d*$_6$) δ 7.21 (t, *J* = 7.9 Hz, 1H), 7.14 - 7.12 (m, 2H), 6.82 – 6.79 (m, 1H), 5.32 (s, 1H), 5.17 (s, 1H), 4.34 – 4.28 (m, 1H), 3.81 (s, 3H), 3.70 – 3.64 (m, 2H), 3.22 (dd, *J* = 15.1, 7.4 Hz, 1H), 3.06 (dd, *J* = 15.1, 5.0 Hz, 1H), 1.70 - 1.59 (m, 1H), 1.36 (s, 9H).

^{13}C NMR (101 MHz, acetone-*d*$_6$) δ 159.7, 156.6, 146.2, 143.4, 128.8, 118.6, 112.7, 112.3, 112.0, 77.3, 63.1, 54.6, 49.4, 38.1, 27.9, 24.4.

^{19}F NMR (377 MHz, acetone-*d*$_6$) δ -141.45.

HRMS (ESI) Calcd for $C_{18}H_{24}BF_3KNO_3$ $[M - K]^-$: 370.1807, Found: 370.1809.

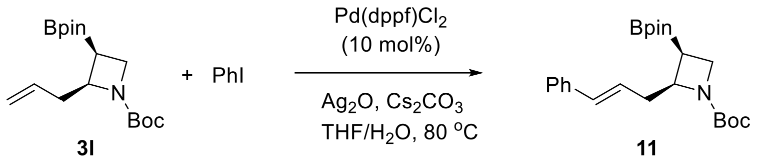

***tert*-Butyl(2*S*,3*S*)-2-cinnamyl-3-(4,4,5,5-tetramethyl-1,3,2-dioxaborolan-2-yl)azetidine-1-carboxylate (11).** At room temperature, to a solution of **3l** (64.6 mg, 0.2 mmol, 1.0 equiv) in THF/H_2O (2.2 mL, v/v = 10:1), were added Pd(dppf)Cl_2 (16.3 mg, 10 mol%), Ag_2O (92.5 mg, 0.4 mmol, 2.0 equiv), PhI (33.6 uL, 3.0 mmol, 1.5 equiv), and Cs_2CO_3 (130.5 mg, 0.4 mmol, 2.0 equiv). The mixture was stirred at 80 °C for 12 h and then cooled to room temperature before it was quenched by the addition of water. The layers were separated, and the aqueous layer was extracted with ethyl acetate (5 mL × 3). The combined organic layers were dried over Na_2SO_4 and concentrated *in vacuo*. The residue was purified by silica gel column chromatography (chromatography eluent: *n*-hexane/EtOAc = 10:1) to afford the product **11** as a yellow oil (50.3 mg, 63% yield, 98% ee, >20:1 dr).

$[\alpha]_D^{25}$: 44.5 (c = 1.0, $CHCl_3$). HPLC analysis of the product: Daicel CHIRALPAK® IC-3 column; 10% *i*-PrOH in *n*-hexane; 1.0 mL/min; retention times: 7.4 min (major), 8.7 min (minor).

^{1}H NMR (400 MHz, $CDCl_3$) δ 7.37 – 7.26 (m, 4H), 7.20 (t, *J* = 7.1 Hz, 1H), 6.44 (d, *J* = 16.0 Hz, 1H), 6.27 (dt, *J* = 15.8, 6.8 Hz, 1H), 4.52 – 4.39 (m, 1H), 3.93 (p, *J* = 7.8 Hz, 2H), 2.77 (ddt, *J* = 21.5, 13.9, 8.1 Hz, 2H), 2.30 (td, *J* = 9.7, 7.1 Hz, 1H), 1.43 (s, 9H), 1.28 (d, *J* = 7.1 Hz, 12H).

^{13}C NMR (101 MHz, $CDCl_3$) δ 156.4, 137.7, 132.0, 128.4, 127.0, 126.2, 126.0, 83.8, 79.2, 62.9, 48.2, 37.4, 28.5, 25.0, 16.9.

HRMS (ESI) Calcd for $C_{23}H_{34}BNNaO_4^+$ $[M + Na]^+$: 422.2473, Found: 422.2486.

VI. Asymmetric Synthesis of the Muginetic Acid Precursor

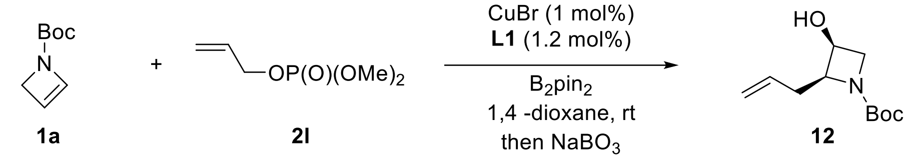

***tert*-Butyl (2*S*,3*S*)-2-allyl-3-hydroxyazetidine-1-carboxylate (12).** In a glove box, a mixture of CuBr (8.6 mg, 0.06 mmol, 1.0 mol%) and (*S*,*S*)-**L1** (36.5 mg, 0.072 mmol, 1.2 mol%) in dry 1,4-dioxane (0.1 M, 60 mL) was stirred at room temperature for 30 min. Then, azetine **1a** (0.93 g, 6.0 mmol, 1.0 equiv), B_2pin_2 (2.3 g, 9.0 mmol, 1.5 equiv), NaO^{t}Bu (0.86 g, 9.0 mmol, 1.5 equiv), and allyl phosphate **2l** (1.5 g, 9.0 mmol, 1.5 equiv) were added sequentially. The mixture was stirred at room temperature for 24 h before it was concentrated *in vacuo*. The residue was used for the next step without further purification.

To a mixture of the above-prepared crude product in THF/H_2O (40 mL, v/v = 1:1) was added $NaBO_3{\cdot}4H_2O$ (4.6 g, 30.0 mmol, 5.0 equiv). The reaction mixture was stirred at room temperature for 2 h before it was diluted with H_2O (20 mL) and extracted with ethyl acetate (30 mL × 3). The combined organic layers were dried over Na_2SO_4 and concentrated *in vacuo*. The residue was purified by silica gel column chromatography (chromatography eluent: *n*-hexane/EtOAc = 2:1) to afford the product **12** as a yellow oil (1.2 g, 92% yield, >20:1 rr).

$[\alpha]_D^{25}$: +120.4 (c = 1.5, $CHCl_3$).

^{1}H NMR (400 MHz, $CDCl_3$) δ 5.92 (ddt, *J* = 17.1, 10.3, 6.8 Hz, 1H), 5.16 (dq, *J* = 17.3, 1.7 Hz, 1H), 5.09 (ddd, *J* = 11.4, 2.4, 1.1 Hz, 2H), 4.62 (td, *J* = 6.9, 4.3 Hz, 1H), 4.36 – 4.24 (m, 1H), 4.13 (dd, *J* = 9.8, 7.1 Hz, 1H), 3.70 (ddd, *J* = 9.8, 4.3, 1.1 Hz, 1H), 2.75 – 2.52 (m, 2H), 1.44 (s, 9H).

^{13}C NMR (101 MHz, CDCl3) δ 156.2, 135.2, 117.0, 79.6, 66.1, 63.7, 57.0, 32.8, 28.4.

HRMS (ESI) Calcd for $C_{11}H_{19}NNaO_3^+$ [M + Na]$^+$: 236.1257, Found: 236.1256.

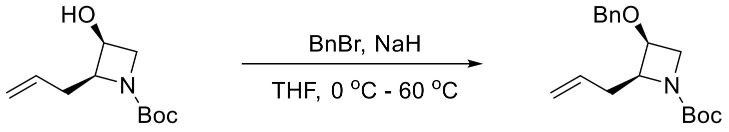

***tert*-Butyl (2*S*,3*S*)-2-allyl-3-(benzyloxy)azetidine-1-carboxylate (13).** To a solution of **12** (1.1 g, 5.0 mmol, 1.0 equiv) in THF (10 mL) was added NaH (0.3 g, 7.5 mmol, 60 wt% in mineral oil, 1.5 equiv) at 0 °C. The mixture was stirred at room temperature for 30 min before BnBr (0.8 mL, 6.5 mmol, 1.3 equiv) was added. The reaction mixture was stirred at 60 °C for 18 h and then allowed to cool to room temperature. A saturated aqueous NH_4Cl solution (20 mL) was added. The layers were separated, and the aqueous layer was extracted with ethyl acetate (30 mL × 3). The combined organic layers were dried over Na_2SO_4 and concentrated under reduced pressure. The residue was purified by silica gel column chromatography (chromatography eluent: *n*-hexane/EtOAc = 10:1) to afford the desired product **13** as a yellow oil (1.1 g, 71% yield, >99% ee, >20:1 rr).

$[\alpha]_D^{25}$: +53.7 (c = 3.0, $CHCl_3$). HPLC analysis of the product: Daicel CHIRALPAK® IN-3 column; 10% *i*-PrOH in *n*-hexane; 1.0 mL/min; retention times: 6.6 min (major), 6.2 min (minor).

^{1}H NMR (400 MHz, $CDCl_3$) δ 7.38 – 7.27 (m, 5H), 5.92 (ddt, *J* = 17.2, 10.2, 7.0 Hz, 1H), 5.13 – 5.03 (m, 2H), 4.46 (s, 2H), 4.37 – 4.30 (m, 2H), 4.03 – 3.99 (m, 1H), 3.76 (dd, *J* = 9.5, 3.3 Hz, 1H), 2.72 – 2.57 (m, 2H), 1.43 (s, 9H).

^{13}C NMR (101 MHz, CDCl3) δ 156.0, 137.6, 135.1, 128.4, 127.8, 127.4, 116.8, 79.5, 71.6, 69.3, 66.4, 55.2, 33.3, 28.4.

HRMS (ESI) Calcd for $C_{18}H_{25}NNaO_3^+$ [M + Na]$^+$: 326.1727, Found: 326.1732.

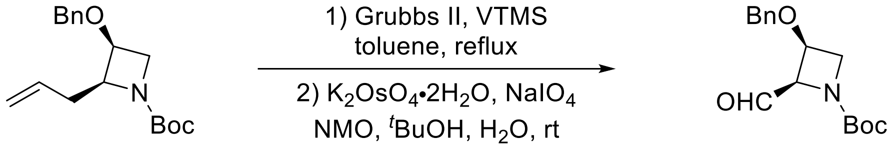

13 **14**

***tert*-Butyl (2*R*,3*S*)-3-(benzyloxy)-2-formylazetidine-1-carboxylate (14)**. To a solution of **13** (606.0 mg, 2.0 mmol, 1.0 equiv) and vinyloxy trimethylsilane (3.0 mL, 20.0 mmol, 10.0 equiv) in toluene (80 mL) was added the Grubbs 2nd generation catalyst (84.9 mg, 0.1 mmol, 5.0 mol%) at room temperature. The purple reaction mixture was immersed in an oil bath and refluxed for 24 h. The reaction mixture was then concentrated under reduced pressure to afford the desired disubstituted alkene as a dark brown oil, which was used in the next step without further purification.

To a mixture of the above-prepared crude product in ${}^tBuOH/H_2O$ (12 mL/12 mL), $K_2OsO_4 \cdot 2H_2O$ (11.1 mg, 0.03 mmol, 1.5 mol%), NMO (702.9 mg, 6.0 mmol, 3.0 equiv), and $NaIO_4$ (1.3 g, 6.0 mmol, 3.0 equiv) were added sequentially at room temperature and stirred for 5 h. The mixture was then diluted with water (30 mL) and extracted with ethyl acetate (10 mL x 3). The combined organic layers were dried over Na_2SO_4 and concentrated under reduced pressure. The residue was purified by silica gel column chromatography (chromatography eluent: *n*-hexane/EtOAc = 2:1) to afford the product **14** as a yellow oil (582 mg, 45% yield for two steps, >20:1 dr).

$[\alpha]_D^{25}$: +83.1 (c = 2.6, $CHCl_3$).

^{1}H NMR (400 MHz, $CDCl_3$) δ 9.77 (s, 1H), 7.38 – 7.26 (m, 5H), 4.72 – 4.60 (m, 1H), 4.54 – 4.41 (m, 3H), 4.16 (dd, *J* = 9.3, 6.7 Hz, 1H), 4.01 (dd, *J* = 9.3, 4.6 Hz, 1H), 1.43 (s, 9H).

^{13}C NMR (101 MHz, $CDCl_3$) δ 199.2, 155.1, 136.3, 128.5, 128.1, 127.8, 80.5, 72.0, 71.8, 69.6, 55.5, 28.1.

HRMS (ESI) Calcd for $C_{16}H_{21}NNaO_4^+$ [M + Na]$^+$: 314.1363, Found: 314.1367.

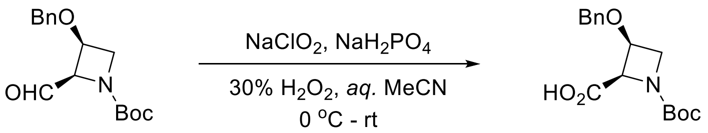

14 **15**

(2*R*,3*S*)-3-(Benzyloxy)-1-(*tert*-butoxycarbonyl)azetidine-2-carboxylic acid (14). To a stirred solution of **12** (116.4 mg, 0.4 mmol, 1.0 equiv) in acetonitrile (4 mL) were added a solution of sodium dihydrogen phosphate (9.6 mg, 0.08 mmol, 20 mol%) in water (0.2 mL) and 30% H_2O_2 (11.1 uL, 0.5 mmol, 1.2 equiv). The mixture was cooled to 0 °C, and $NaClO_2$ (54.2 mg, 0.6 mmol, 1.5 equiv) in water (0.2 mL) was added dropwise over 2 min. The reaction mixture was warmed to room temperature and stirred for 2 h. Then, the reaction mixture was queched by the addition of a small amount of Na_2SO_3 (0.1 g) and 1-2 drops of a HCl solution (10%) followed by extraction with ethyl acetate (5 mL x 3). The combined organic layers were dried over Na_2SO_4 and concentrated *in vacuo*. The residue was purified by silica gel column chromatography (chromatography eluent: *n*-hexane/EtOAc = 1:1) to afford the product **15** as a yellow oil (113.0 mg, 92% yield, >20:1 dr).

$[\alpha]_D^{25}$: +17.2 (c = 2.8, $CHCl_3$).

^{1}H NMR (400 MHz, $CDCl_3$) δ 10.54 (s, 1H), 7.32 – 7.22 (m, 5H), 4.85 - 4.78 (m, 1H), 4.63 (d, *J* = 11.6 Hz, 1H), 4.51 – 4.42 (m, 2H), 4.05 – 3.98 (m, 2H), 1.42 (s, 9H).

^{13}C NMR (101 MHz, CDCl3) δ 172.6, 155.6, 136.7, 128.4, 127.9, 127.8, 80.8, 72.2, 67.8, 67.5, 55.7, 28.2.

HRMS (ESI) Calcd for $C_{16}H_{21}NNaO_5^+$ [M + Na]$^+$: 330.1312, Found: 330.1318.

VII. Mechanistic Experiments

(a) Radical trapping experiment

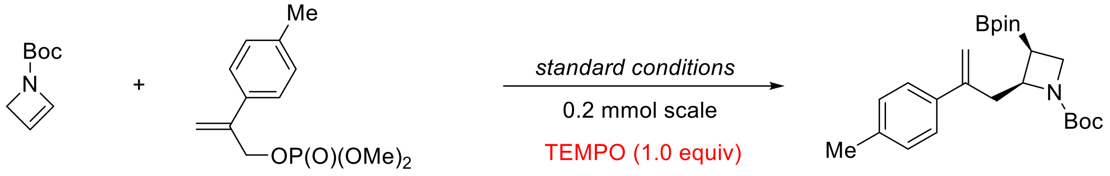

1a **2a** **3a**, 90% NMR yield

In a glove box, a mixture of CuBr (1.4 mg, 0.01 mmol, 5.0 mol%) and the chiral ligand (*S*,*S*)-**L1** (6.1 mg, 0.012 mmol, 6.0 mol%) in dry 1,4-dioxane (0.05 M, 4.0 mL) was stirred at room temperature for 0.5 h. Then, azetine **1a** (31.0 mg, 0.2 mmol, 1.0 equiv), B_2pin_2 (76.2 mg, 0.3 mmol, 1.5 equiv), NaO^tBu (28.9 mg, 0.3 mmol, 1.5 equiv), allyl phosphate **2a** (76.8 mg, 0.3 mmol, 1.5 equiv) and TEMPO (31.3 mg, 0.2 mmol, 1.0 equiv) were added sequentially. The mixture was stirred at room temperature for 10 h. Next, the mixture was concentrated *in vacuo*, and ^1H NMR analysis of the crude product indicated that the desired product was formed in 90% yield.

(b) Deuterium-labeling study

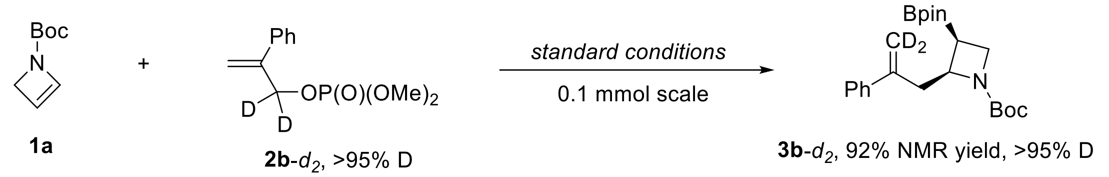

1a **2b**-d_2, >95% D **3b**-d_2, 92% NMR yield, >95% D

General procedure C was scaled down to 0.1 mmol scale. In a glove box, a mixture of CuBr (0.7 mg, 0.005 mmol, 5.0 mol%) and the chiral ligand (*S*,*S*)-**L1** (3.0 mg, 0.006 mmol, 6.0 mol%) in dry 1,4-dioxane (0.05 M, 2.0 mL) was stirred at room temperature for 0.5 h. Then, azetine **1a** (15.5 mg, 0.1 mmol, 1.0 equiv), B_2pin_2 (38.1 mg, 0.15 mmol, 1.5 equiv), NaO^tBu (14.4 mg, 0.15 mmol, 1.5 equiv), and allyl phosphate **2b**-d_2 (36.6 mg, 0.15 mmol, 1.5 equiv) were added sequentially. The mixture was stirred at room temperature for 10 h. Next, the mixture was concentrated *in vacuo*, and the crude product was then subjected

to ^{1}H NMR analysis for yield and isotopic distribution. An aliquot of the crude product was purified by preparative thin layer chromatography.

^{1}H NMR (400 MHz, $CDCl_3$) δ 7.47 – 7.44 (m, 2H), 7.34 – 7.23 (m, 3H), 4.68 - 4.55 (s, 1H), 3.95 (dd, *J* = 10.0, 7.8 Hz, 1H), 3.83 (dd, *J* = 7.7, 6.1 Hz, 1H), 3.14 – 3.02 (m, 2H), 2.23 (td, *J* = 9.6, 6.1 Hz, 1H), 1.43 (s, 9H), 1.18 (s, 12H).

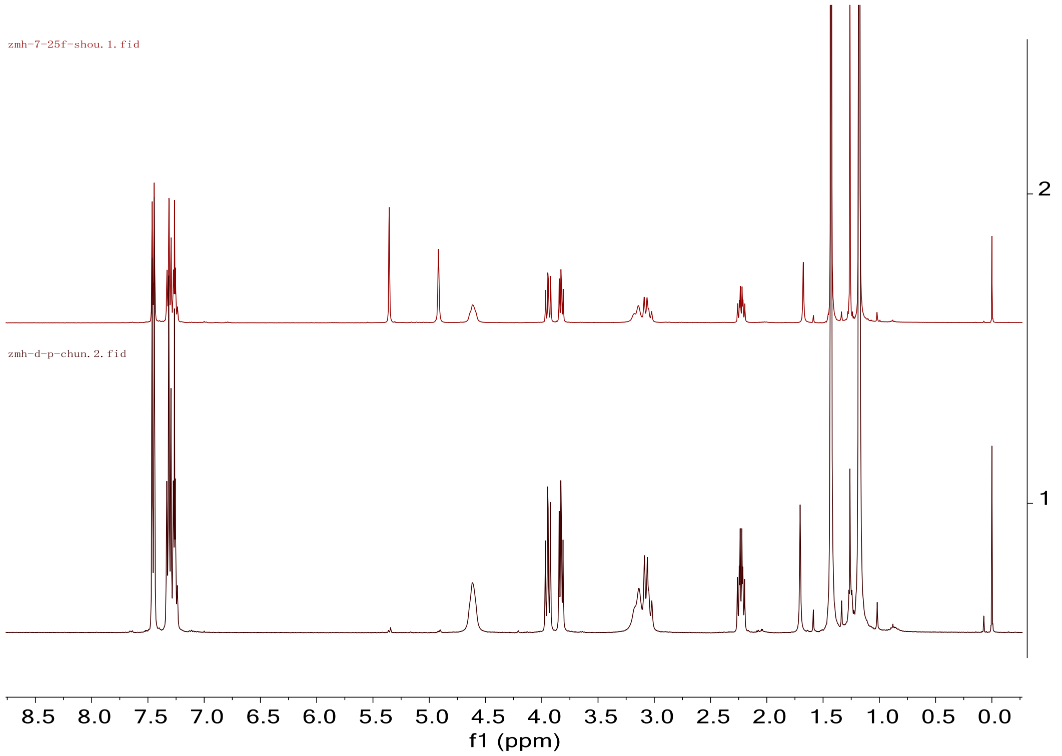

Figure S1. Stacked NMR plots of **3b** (top) and **3b-*d*$_2$** (bottom)

(c) Non-linear effects

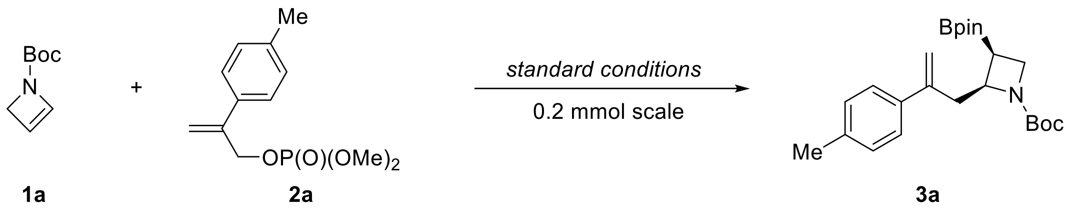

Under the standard conditions, the reaction was carried out using **L1** with different enantiopurities (1st run: 0% ee, 2nd run: 17% ee; 3rd run: 33% ee; 4th run: 50% ee; 5th run: 67% ee; 6th run: 100% ee). The ee values of the product **3a** were determined by HPLC.

ee% (**L1**)	0	17	33	50	67	100
ee% (**3a**)	0	19	34	51	68	99

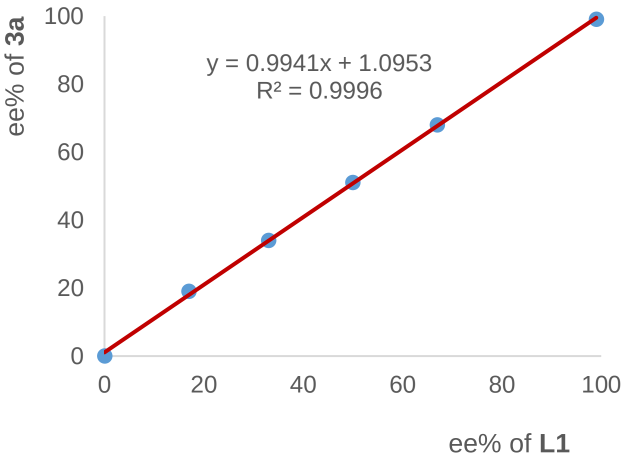

(d) Kinetic studies

Reaction kinetic analysis of the substrate 1a

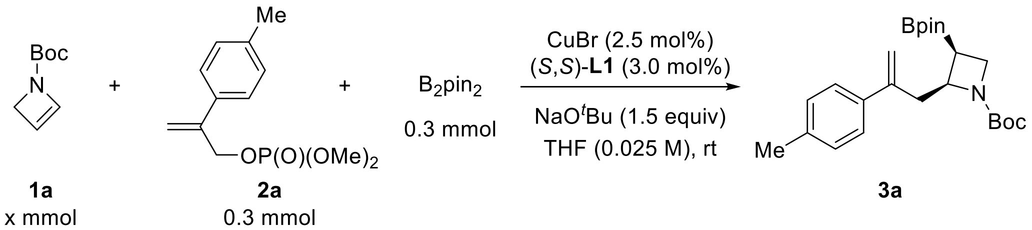

Yield (%)	5 min	8 min	10 min	12 min
0.025 M	12	18	24	26
0.031 M	9	14	16	24
0.038 M	10	13	16	25
0.044 M	14	20	26	29

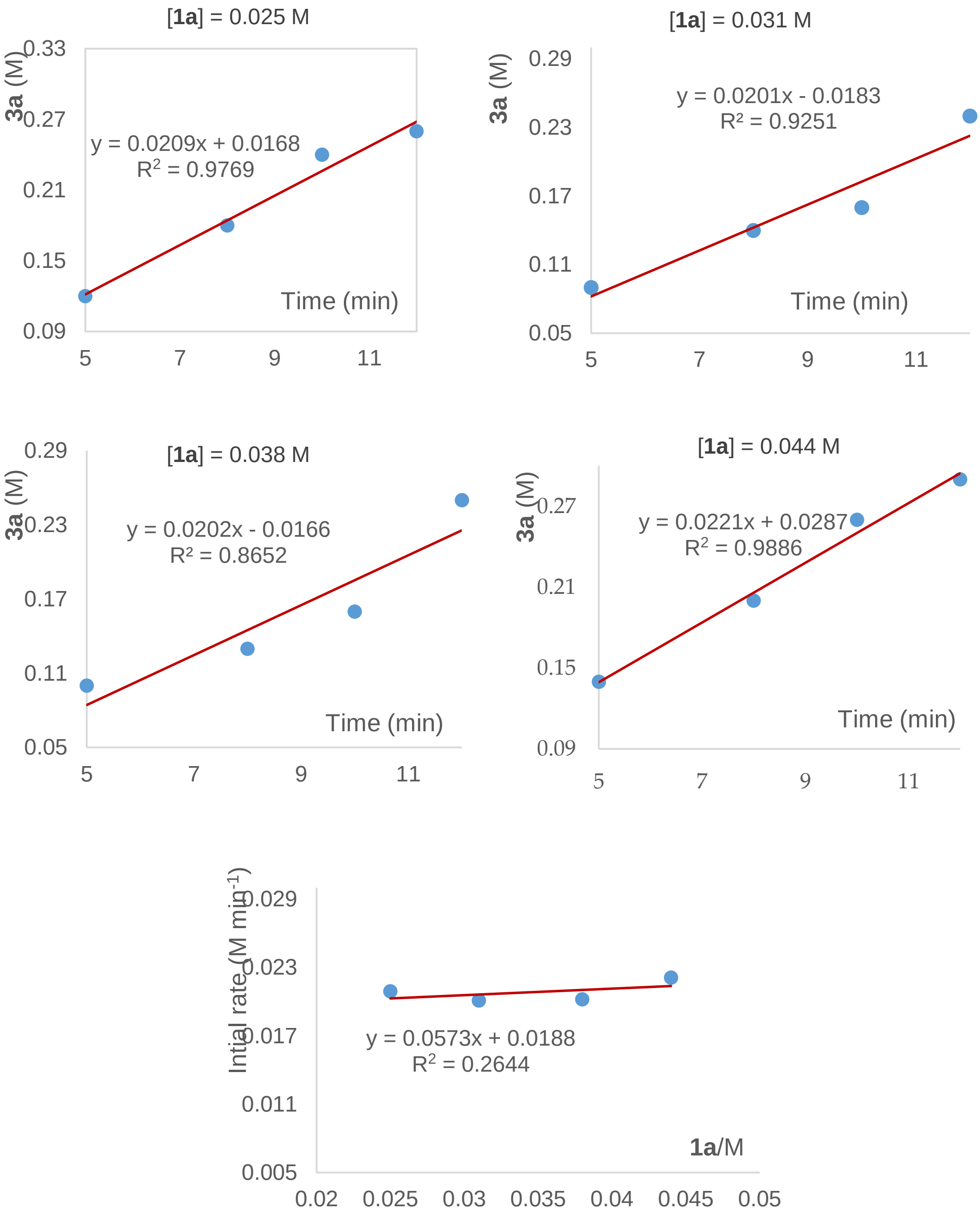

Figure S2. Kinetic profiles of different initial concentrations of **1a** (from 0.025 M to 0.044 M). The plot of reaction rate vs [**1a**] indicated a liner relationship, which indicates a zero-order kinetic dependence in **1a**.

Reaction kinetic analysis of the substrate 2a

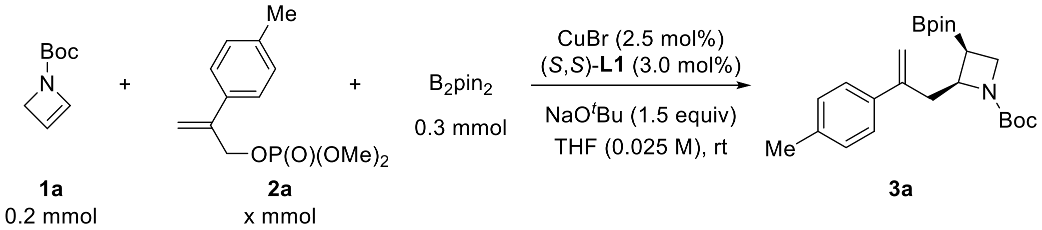

Yield (%)	5 min	8 min	10 min	12 min
0.031 M	11	16	20	25
0.038 M	12	18	24	26
0.044 M	16	22	28	32
0.050 M	11	23	25	31

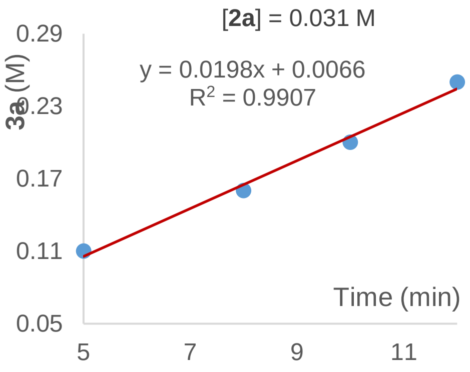

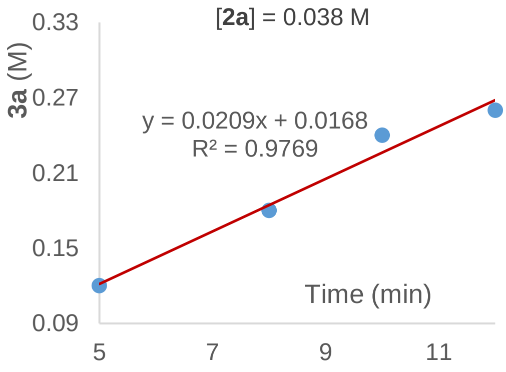

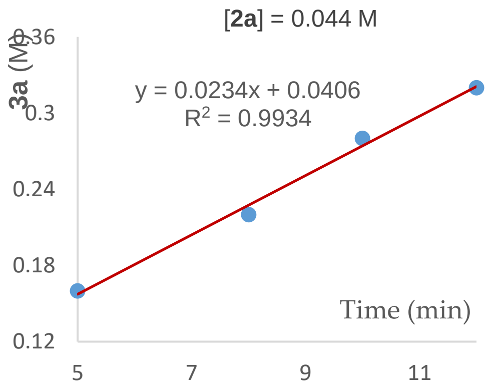

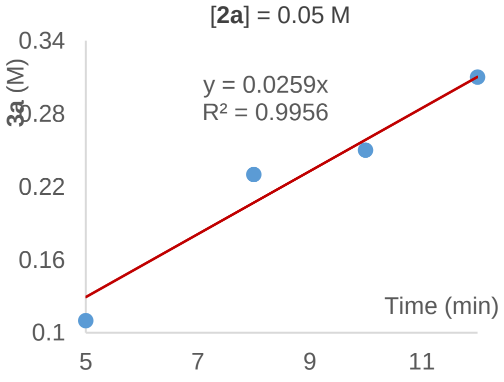

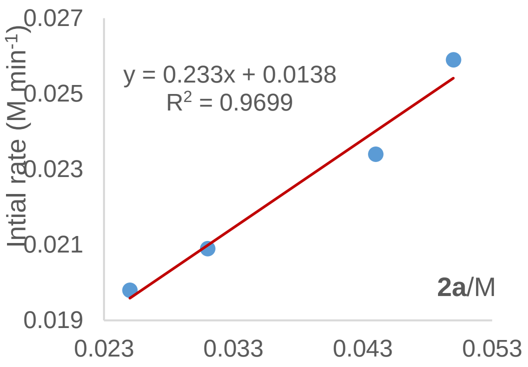

Figure S3. Kinetic profiles of different initial concentrations of **2a** (from 0.031 M to 0.050 M). The plot of reaction rate vs [**2a**] indicated a liner relationship, which indicated a first-order kinetic dependence in **2a**.

Reaction kinetic analysis of the substrate B_2pin_2

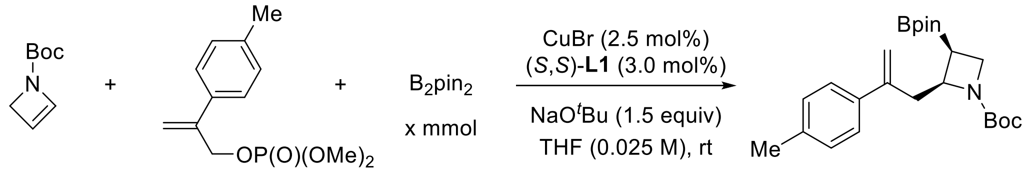

1a 0.2 mmol | **2a** 0.3 mmol | **3a**

Yield (%)	5 min	8 min	10 min	12 min
0.038 M	12	18	24	26
0.044 M	16	22	28	31
0.050 M	14	16	19	29
0.056 M	14	22	23	29

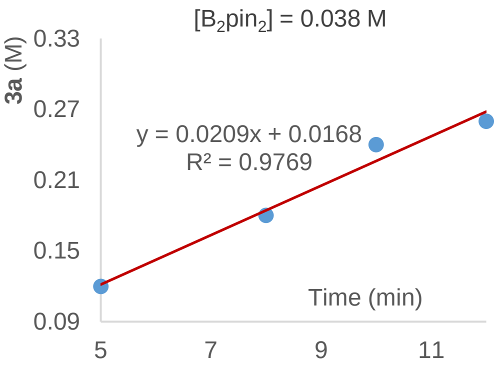

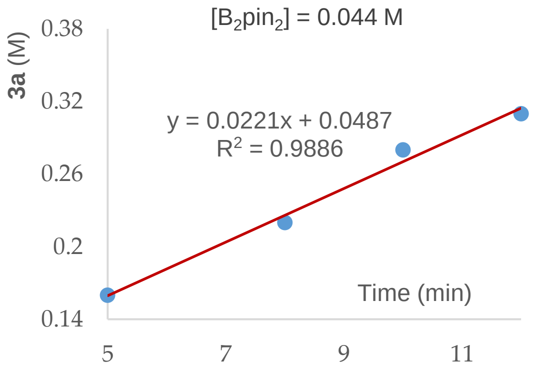

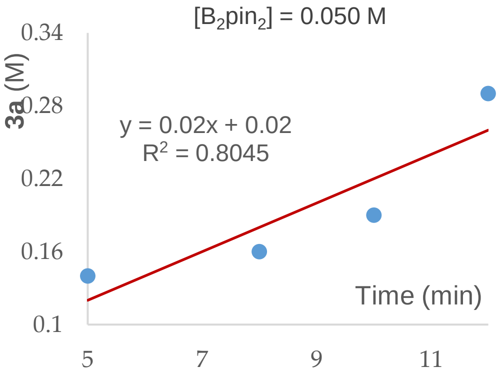

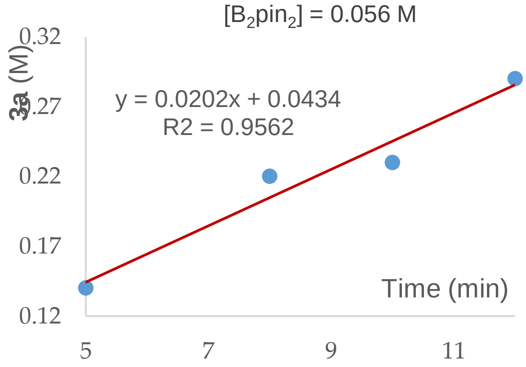

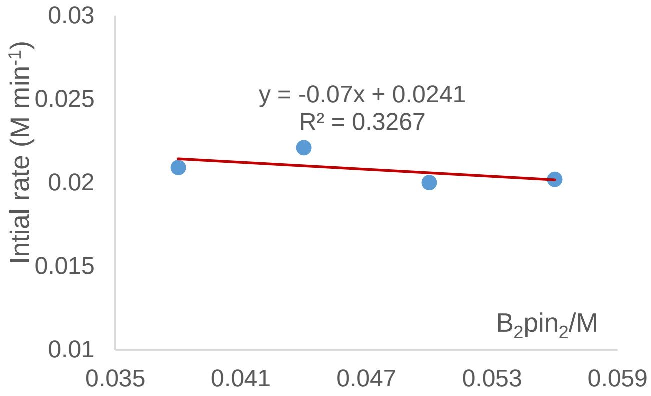

Figure S4. Kinetic profiles of different initial concentrations of [B_2pin_2] (from 0.038 M to 0.056 M). The plot of reaction rate vs [B_2pin_2] indicated a liner relationship, which indicated a zero-order kinetic dependence in B_2pin_2.

Reaction kinetic analysis of CuBr

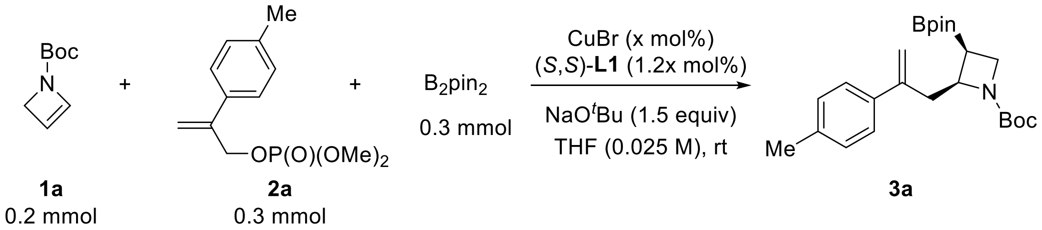

Yield (%)	5 min	8 min	10 min	12 min
0.000625 M	12	18	24	26
0.000875 M	14	22	28	33
0.001125 M	20	34	38	42
0.00110 M	26	36	45	57

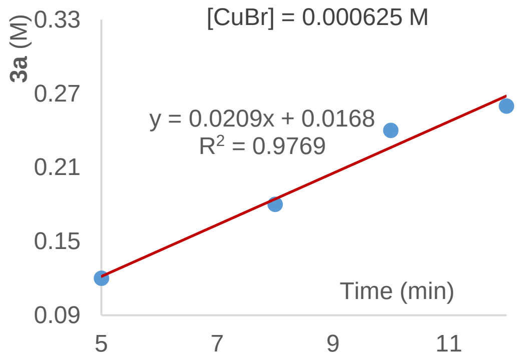

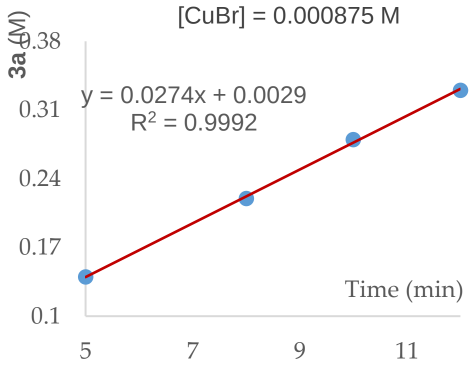

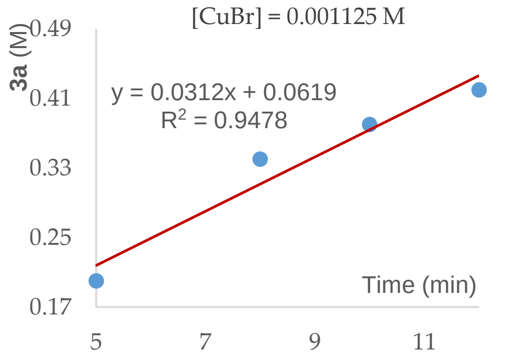

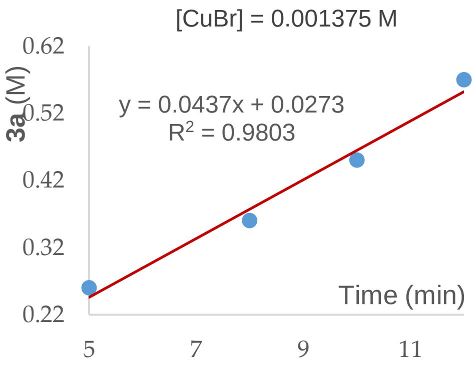

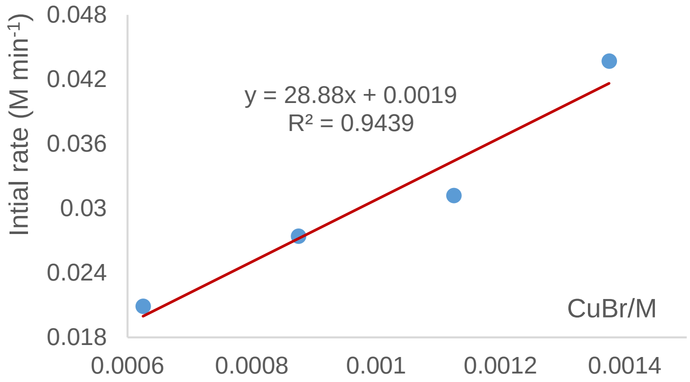

Figure S5. Kinetic profiles of different initial concentrations of [Cu/L] (from 0.000625 M to 0.00110 M), the plot of reaction rate vs [Cu/L] indicated a liner relationship, which indicated a first-order kinetic dependence in Cu/L.

VIII. Product Structure Determination

The structures of products **3f** was determined by X-ray crystallography. The X-ray data have been deposited at the Cambridge Crystallographic Data Center (CCDC 2443178). The structures of other products were assumed by analogy.

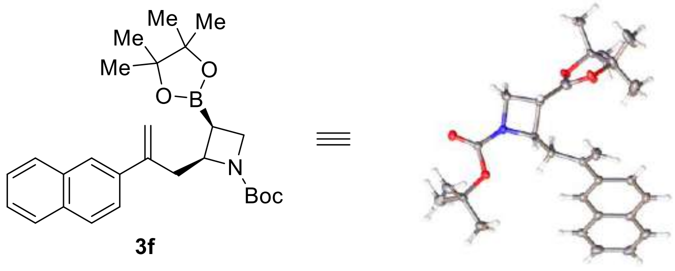

3f

Table S1. Crystal Data and Structure Refinement for 3f.

Identification code	**3f**
Empirical formula	$C_{28}H_{36}NO_4$
Formula weight	450.58
Temperature/K	100.00(10)
Crystal system	orthorhombic
Space group	$P2_12_12_1$
a/Å	6.45514(9)
b/Å	16.57745(16)
c/Å	24.0133(3)
α/°	90
β/°	90
γ/°	90
Volume/Å^3	2569.65(5)
Z	4
ρ_{calc}g/cm^3	1.165
μ/mm^{-1}	0.612

F(000)	972.0
Crystal size/mm^3	0.285 × 0.285 × 0.28
Radiation	CuKα (λ = 1.54184)
2Θ range for data collection/	7.362 to 149.798
Index ranges	$-7 \le h \le 7$, $-20 \le k \le 13$, $-29 \le l \le 28$
Reflections collected	8035
Independent reflections	5008 [R_{int} = 0.0163, R_{sigma} = 0.0235]
Data/restraints/parameters	5008/176/381
Goodness-of-fit on F^2	1.054
Final R indexes [I>=2σ (I)]	R_1 = 0.0341, wR_2 = 0.0893
Final R indexes [all data]	R_1 = 0.0355, wR_2 = 0.0903
Largest diff. peak/hole / e Å^{-3}	0.24/-0.36
Flack parameter	-0.11(7)

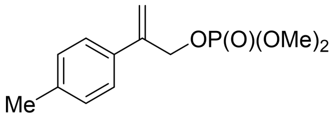
OP(O)(OMe)2
Me

2a

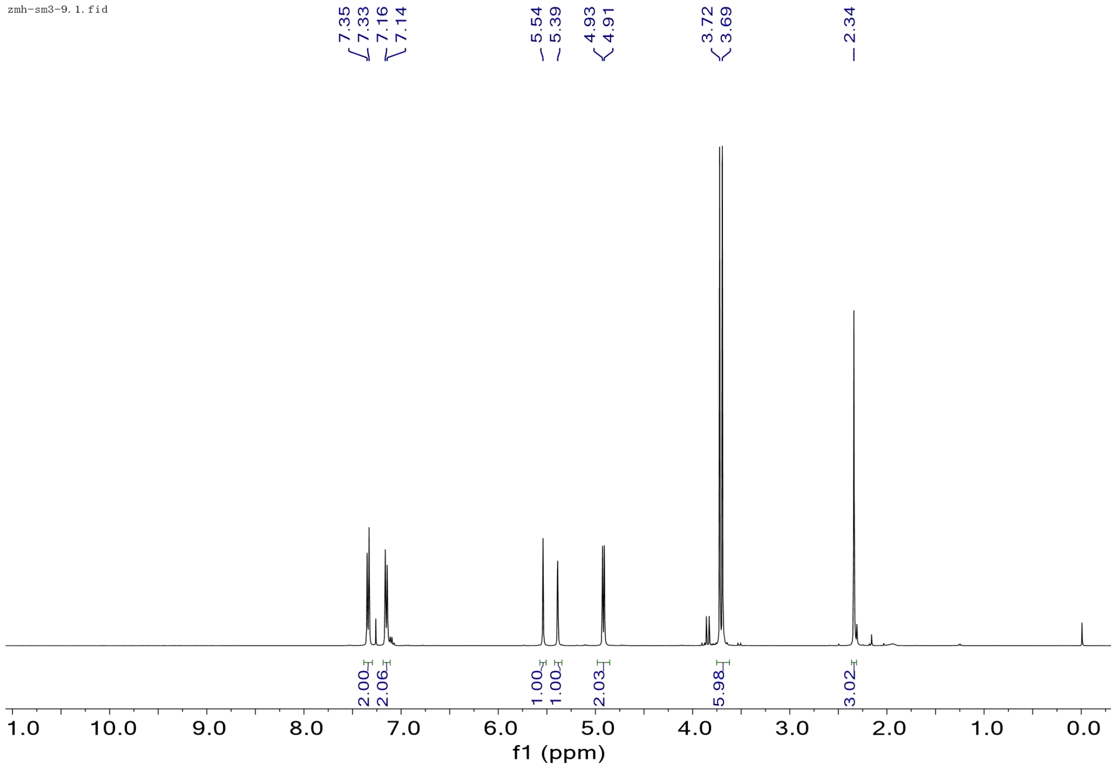
zmh-sm3-9.1.fid
7.35
7.33
7.16
7.14
5.54
5.39
4.93
4.91
3.72
3.69
2.34
2.00
2.06
1.00
1.00
2.03
5.98
3.02
1.0 10.0 9.0 8.0 7.0 6.0 5.0 4.0 3.0 2.0 1.0 0.0
f1 (ppm)

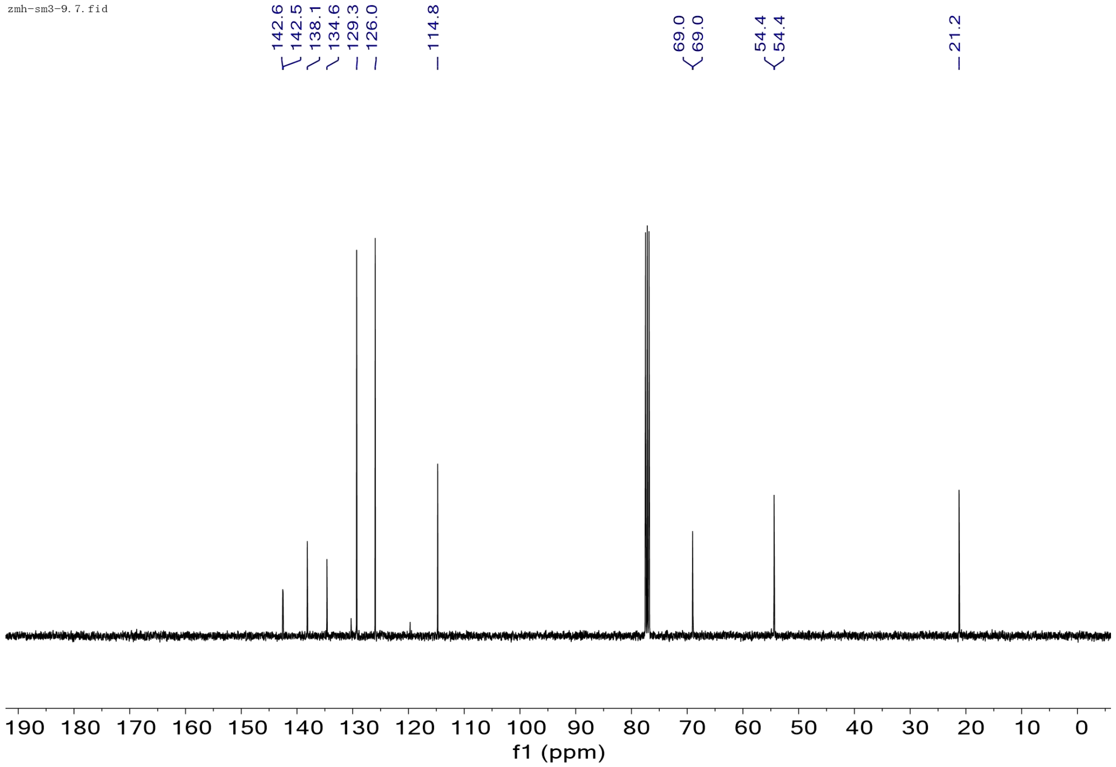
zmh-sm3-9.7.fid
142.6
142.5
138.1
134.6
129.3
126.0
114.8
69.0
69.0
54.4
54.4
21.2
190 180 170 160 150 140 130 120 110 100 90 80 70 60 50 40 30 20 10 0
f1 (ppm)

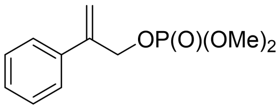
OP(O)(OMe)2

2b

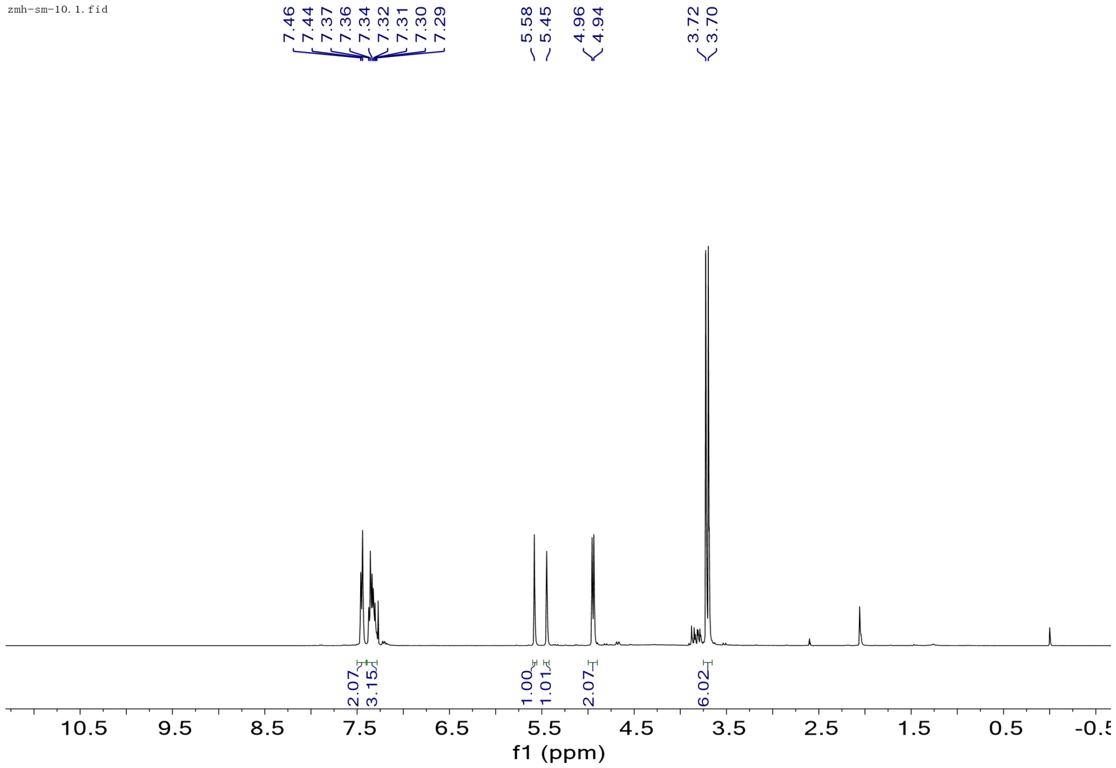
zmh-sm-10.1.fid
7.46
7.44
7.37
7.36
7.34
7.32
7.31
7.30
7.29
5.58
5.45
4.96
4.94
3.72
3.70
2.07
3.15
1.00
1.01
2.07
6.02
10.5 9.5 8.5 7.5 6.5 5.5 4.5 3.5 2.5 1.5 0.5
f1 (ppm)

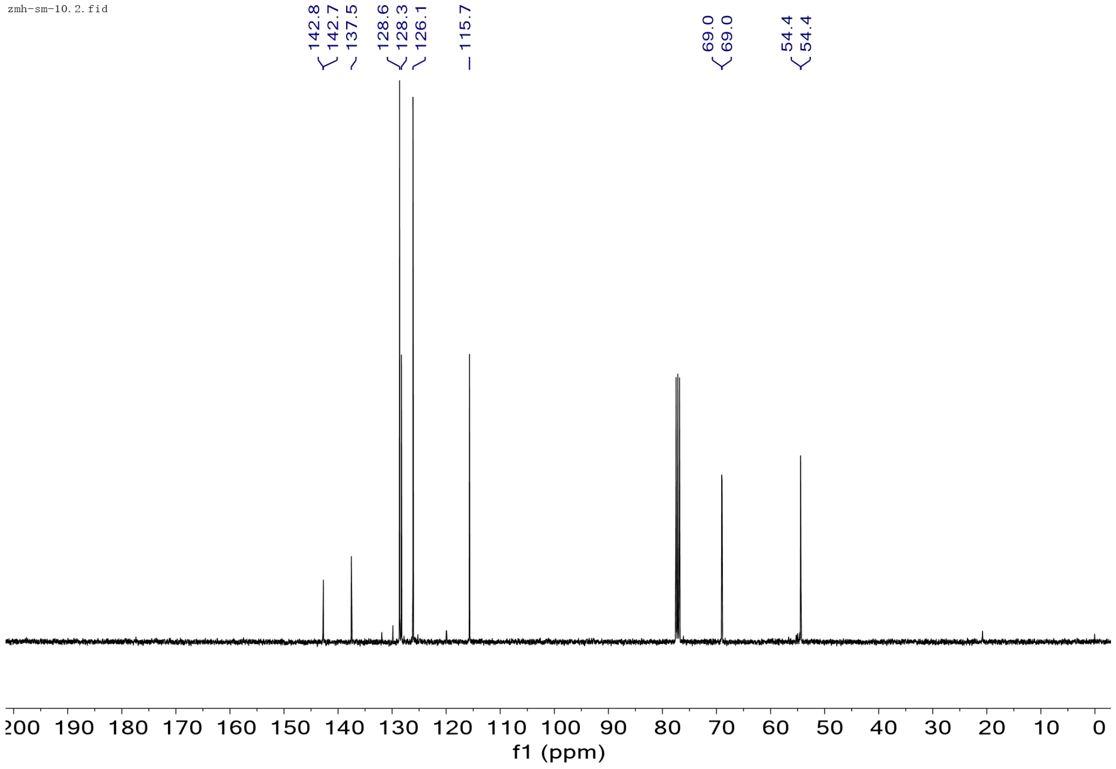
zmh-sm-10.2.fid
142.8
142.7
137.5
128.6
128.3
126.1
115.7
69.0
69.0
54.4
54.4
190 180 170 160 150 140 130 120 110 100 90 80 70 60 50 40 30 20 10 0
f1 (ppm)

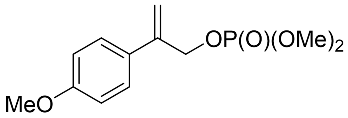
OP(O)(OMe)2
MeO

2c

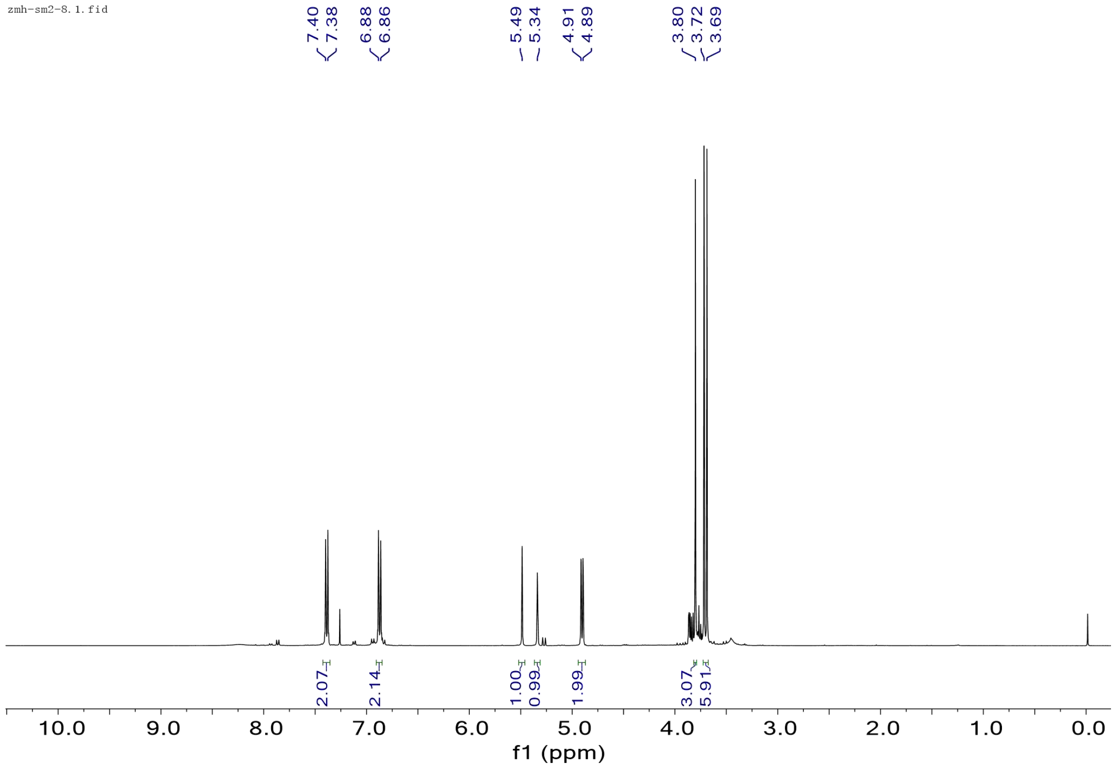
zmh-sm2-8.1.fid
7.40
7.38
6.88
6.86
5.49
5.34
4.91
4.89
3.80
3.72
3.69
2.07
2.14
1.00
0.99
1.99
3.07
5.91
10.0 9.0 8.0 7.0 6.0 5.0 4.0 3.0 2.0 1.0 0.0
f1 (ppm)

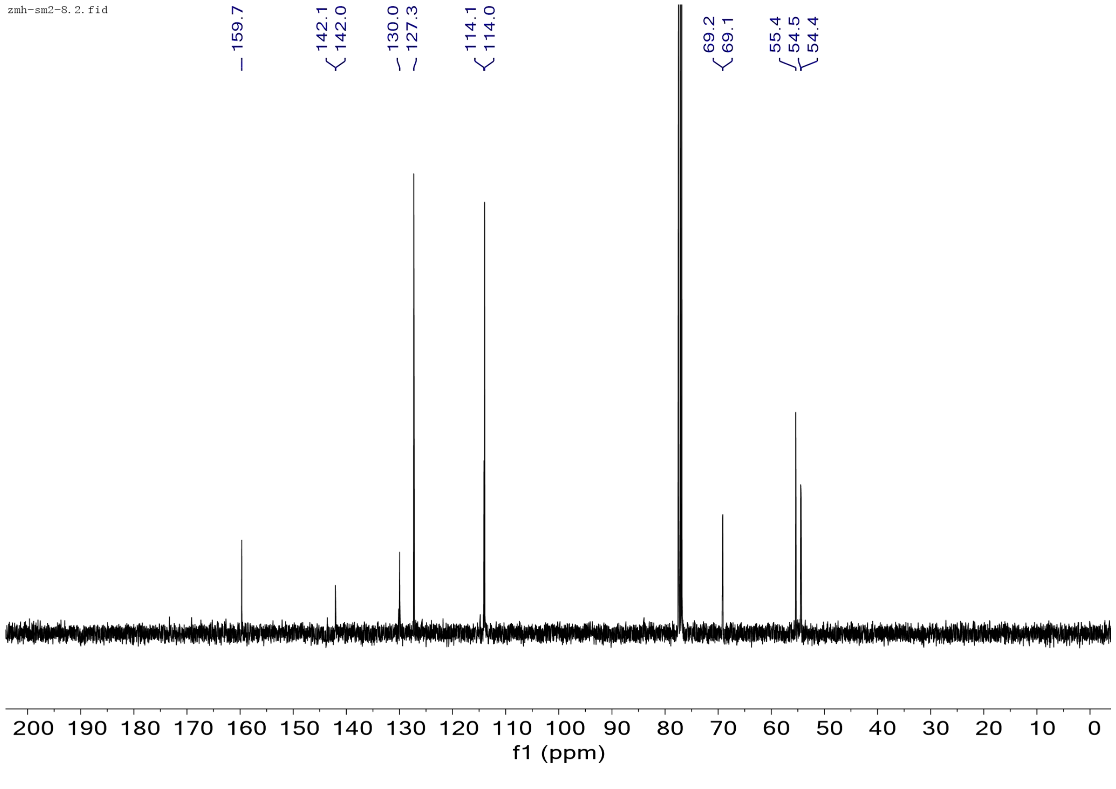
zmh-sm2-8.2.fid
159.7
142.1
142.0
130.0
127.3
114.1
114.0
69.2
69.1
55.4
54.5
54.4
200 190 180 170 160 150 140 130 120 110 100 90 80 70 60 50 40 30 20 10 0
f1 (ppm)

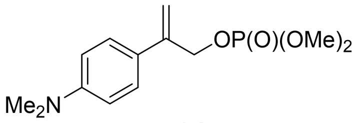
OP(O)(OMe)2
Me2N

2d

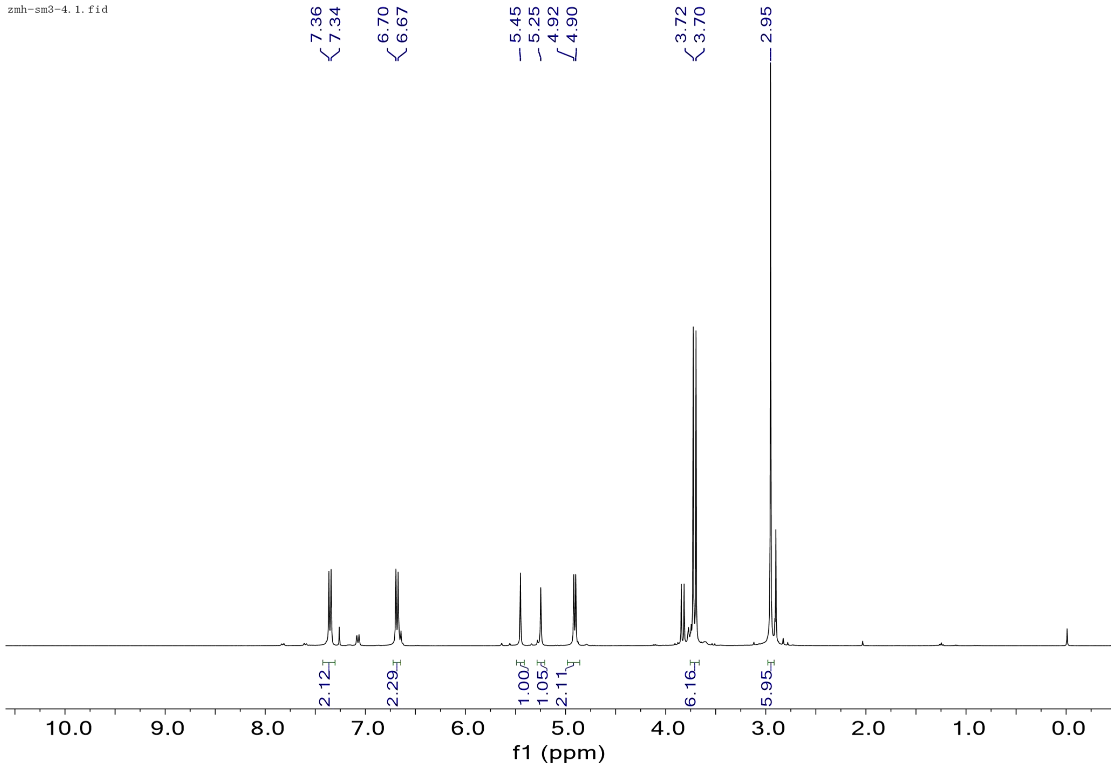
zmh-sm3-4.1.fid
7.36
7.34
6.70
6.67
5.45
5.25
4.92
4.90
3.72
3.70
2.95
2.12
2.29
1.00
1.05
2.11
6.16
5.95
10.0
9.0
8.0
7.0
6.0
5.0
4.0
3.0
2.0
1.0
0.0
f1 (ppm)

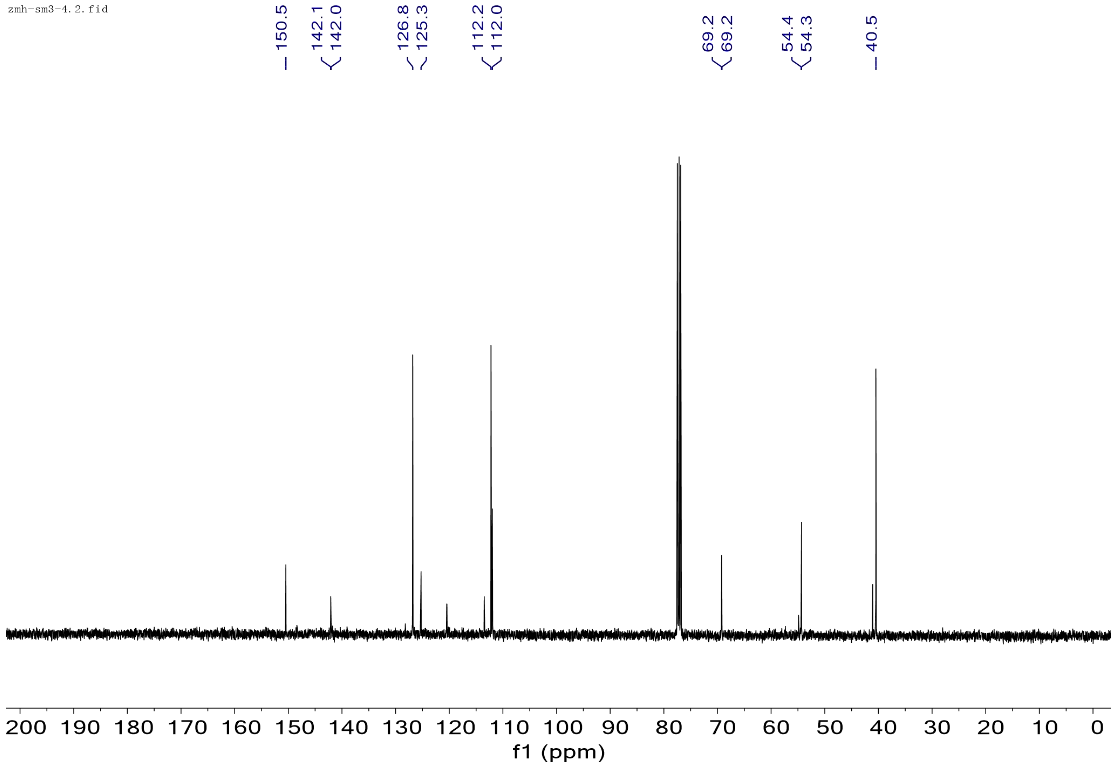
zmh-sm3-4.2.fid
150.5
142.1
142.0
126.8
125.3
112.2
112.0
69.2
69.2
54.4
54.3
40.5
200
190
180
170
160
150
140
130
120
110
100
90
80
70
60
50
40
30
20
10
0
f1 (ppm)

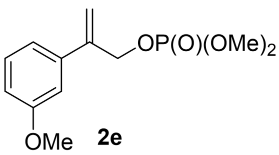
OP(O)(OMe)2
OMe

2e

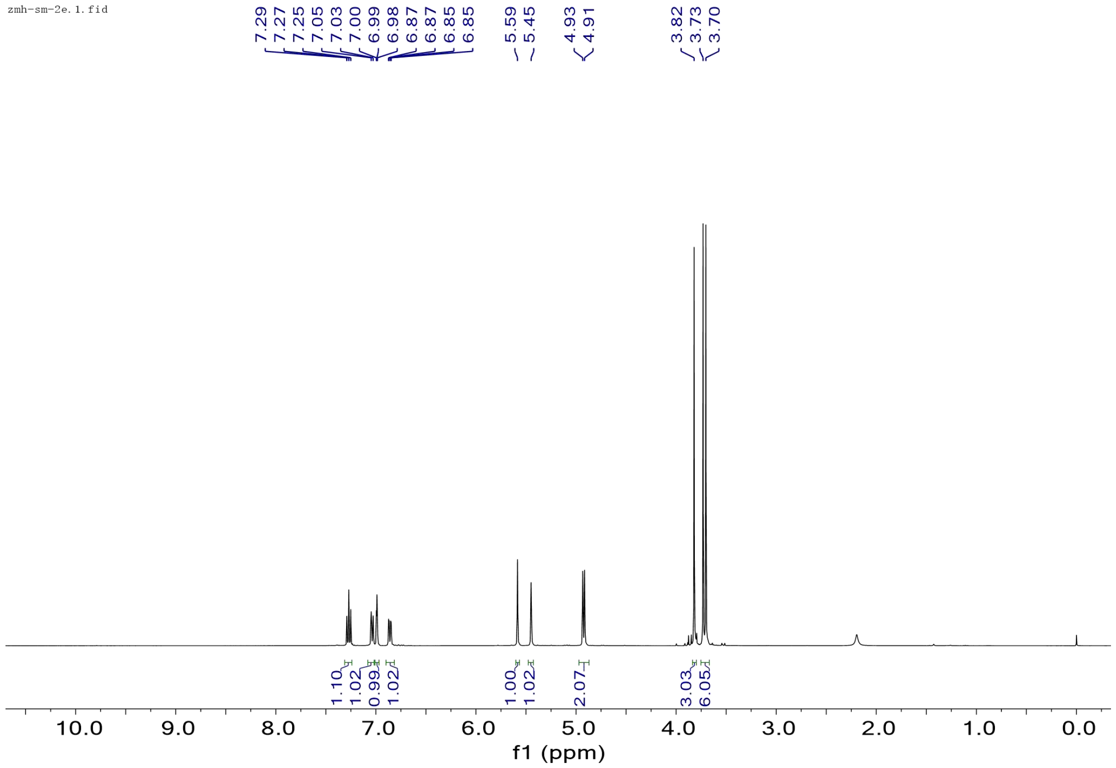
zmh-sm-2e.1.fid
7.29
7.27
7.25
7.05
7.03
7.00
6.99
6.98
6.87
6.87
6.85
6.85
5.59
5.45
4.93
4.91
3.82
3.73
3.70
1.10
1.02
0.99
1.02
1.00
1.02
2.07
3.03
6.05
10.0
9.0
8.0
7.0
6.0
5.0
4.0
3.0
2.0
1.0
0.0
f1 (ppm)

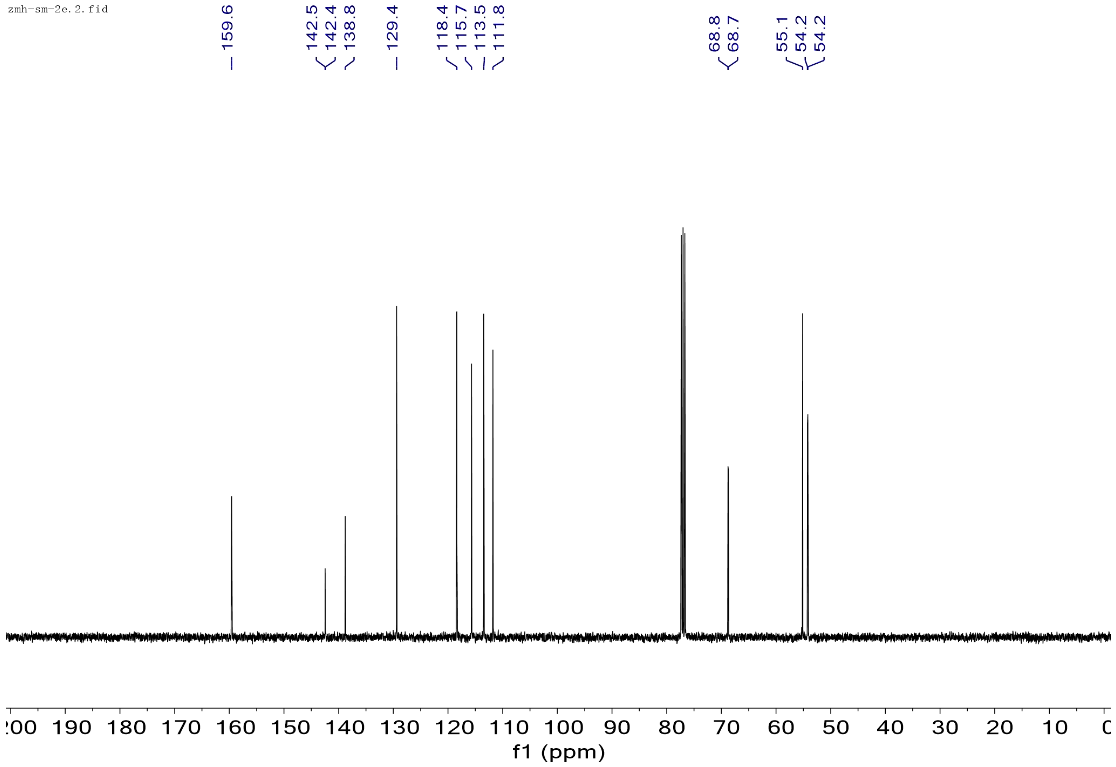
zmh-sm-2e.2.fid
159.6
142.5
142.4
138.8
129.4
118.4
115.7
113.5
111.8
68.8
68.7
55.1
54.2
54.2
190
180
170
160
150
140
130
120
110
100
90
80
70
60
50
40
30
20
10
f1 (ppm)

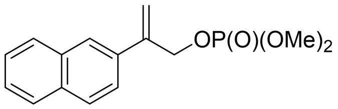
OP(O)(OMe)2

2f

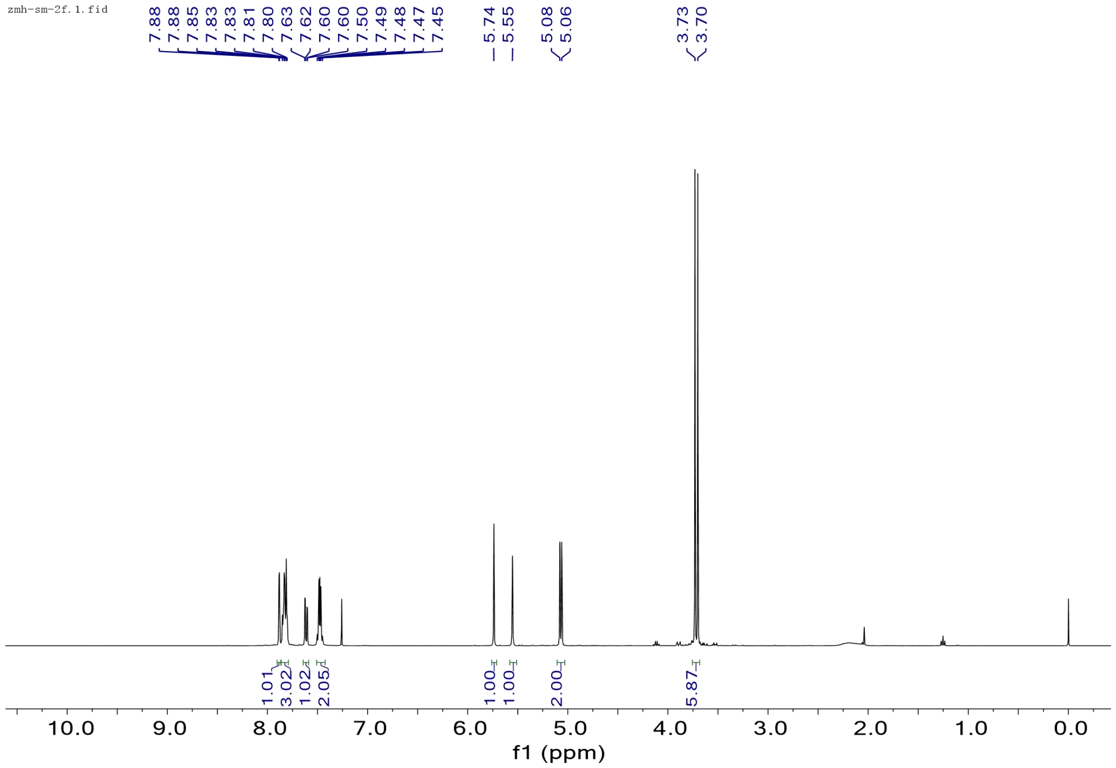
zmh-sm-2f.1.fid
7.88
7.88
7.85
7.83
7.83
7.81
7.80
7.63
7.62
7.60
7.60
7.50
7.49
7.48
7.47
7.45
5.74
5.55
5.08
5.06
3.73
3.70
1.01
3.02
1.02
2.05
1.00
1.00
2.00
5.87
10.0
9.0
8.0
7.0
6.0
5.0
4.0
3.0
2.0
1.0
0.0
f1 (ppm)

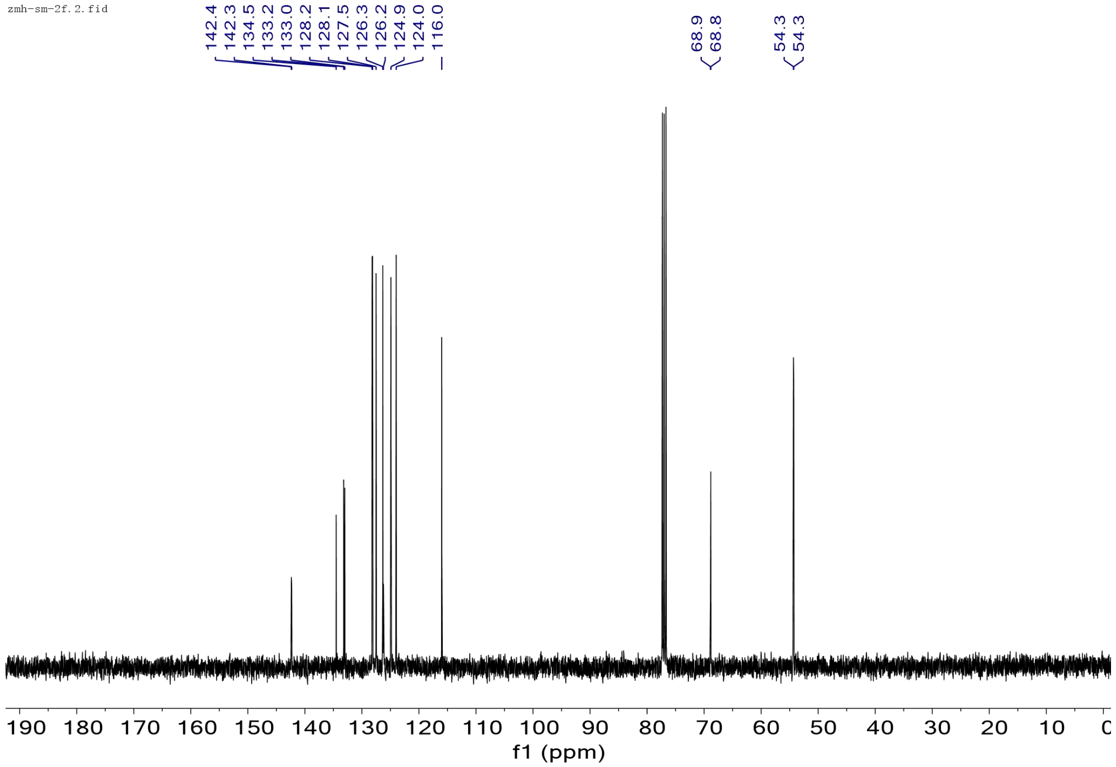
zmh-sm-2f.2.fid
142.4
142.3
134.5
133.2
133.0
128.2
128.1
127.5
126.3
126.2
124.9
124.0
116.0
68.9
68.8
54.3
54.3
190
180
170
160
150
140
130
120
110
100
90
80
70
60
50
40
30
20
10
f1 (ppm)

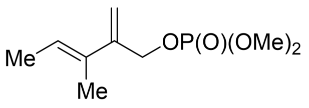
OP(O)(OMe)2
Me
Me

2g

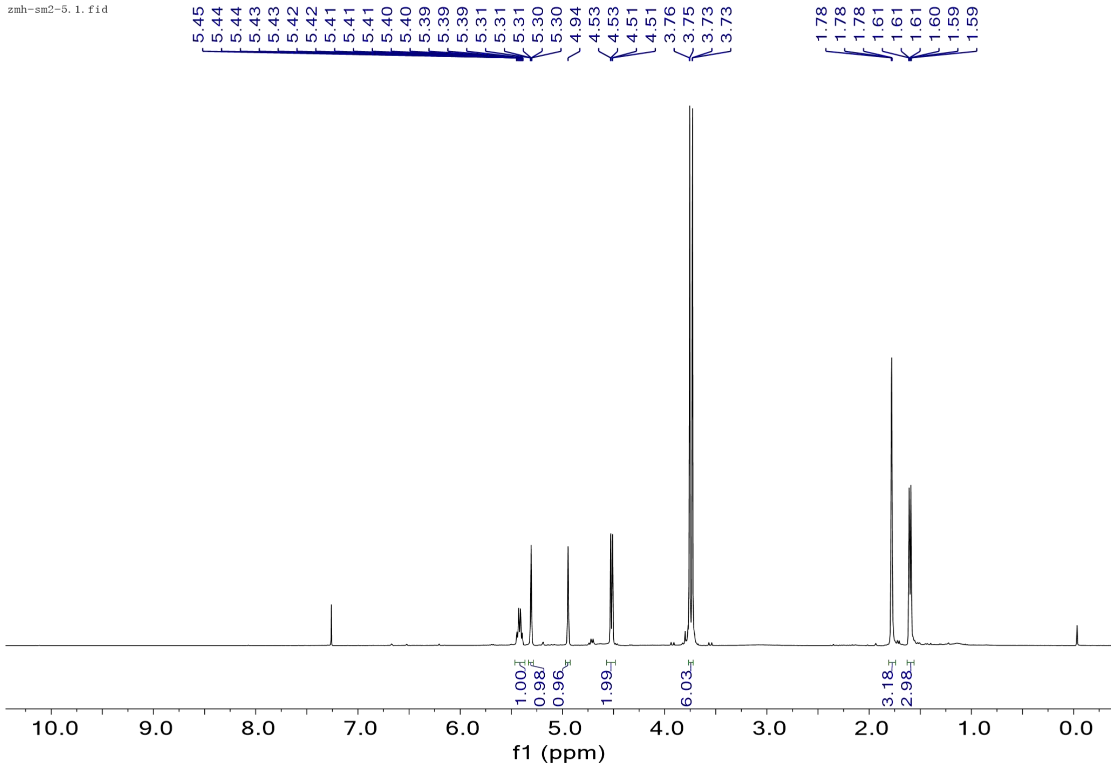
zmh-sm2-5.1.fid
5.45 5.44 5.44 5.43 5.43 5.42 5.42 5.41 5.41 5.40 5.40 5.39 5.39 5.39 5.31 5.31 5.30 5.30 4.94 4.53 4.53 4.51 4.51 3.76 3.75 3.73 3.73 1.78 1.78 1.78 1.61 1.61 1.61 1.60 1.59 1.59
1.00 0.98 0.96 1.99 6.03 3.18 2.98
10.0 9.0 8.0 7.0 6.0 5.0 4.0 3.0 2.0 1.0 0.0
f1 (ppm)

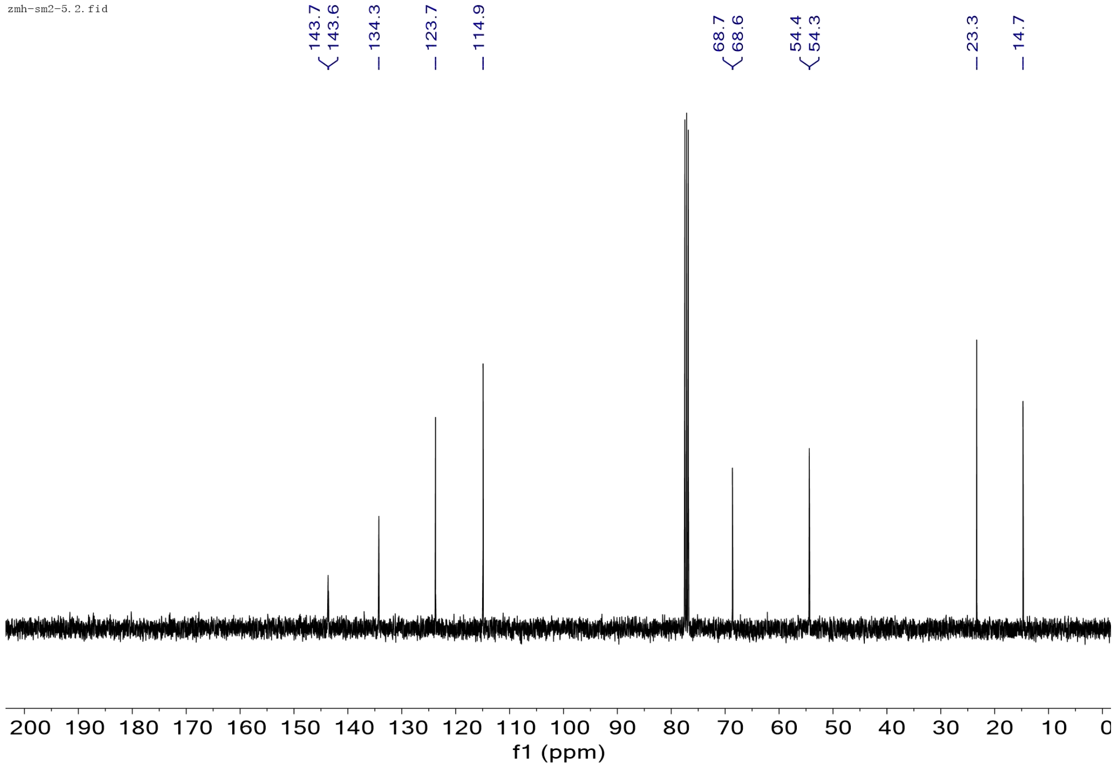
zmh-sm2-5.2.fid
143.7 143.6 134.3 123.7 114.9 68.7 68.6 54.4 54.3 23.3 14.7
200 190 180 170 160 150 140 130 120 110 100 90 80 70 60 50 40 30 20 10 0
f1 (ppm)

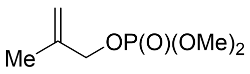
Me
OP(O)(OMe)2

2h

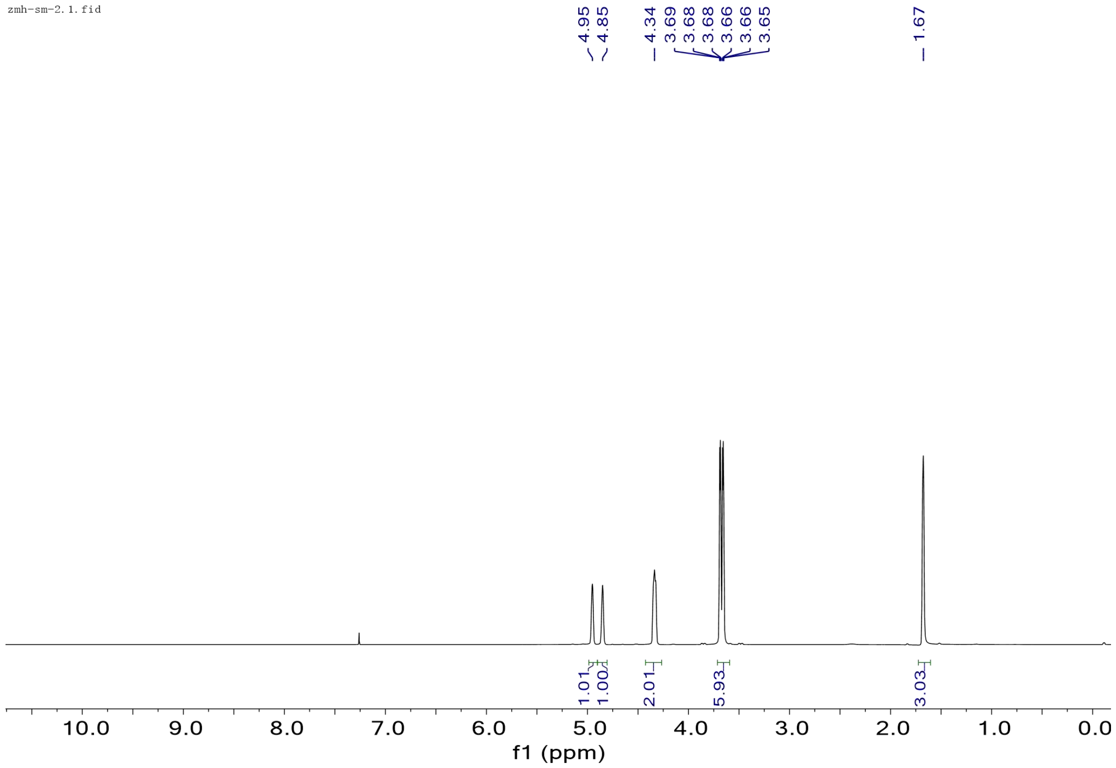
zmh-sm-2.1.fid
4.95
4.85
4.34
3.69
3.68
3.68
3.66
3.66
3.65
1.67
1.01
1.00
2.01
5.93
3.03
10.0 9.0 8.0 7.0 6.0 5.0 4.0 3.0 2.0 1.0 0.0
f1 (ppm)

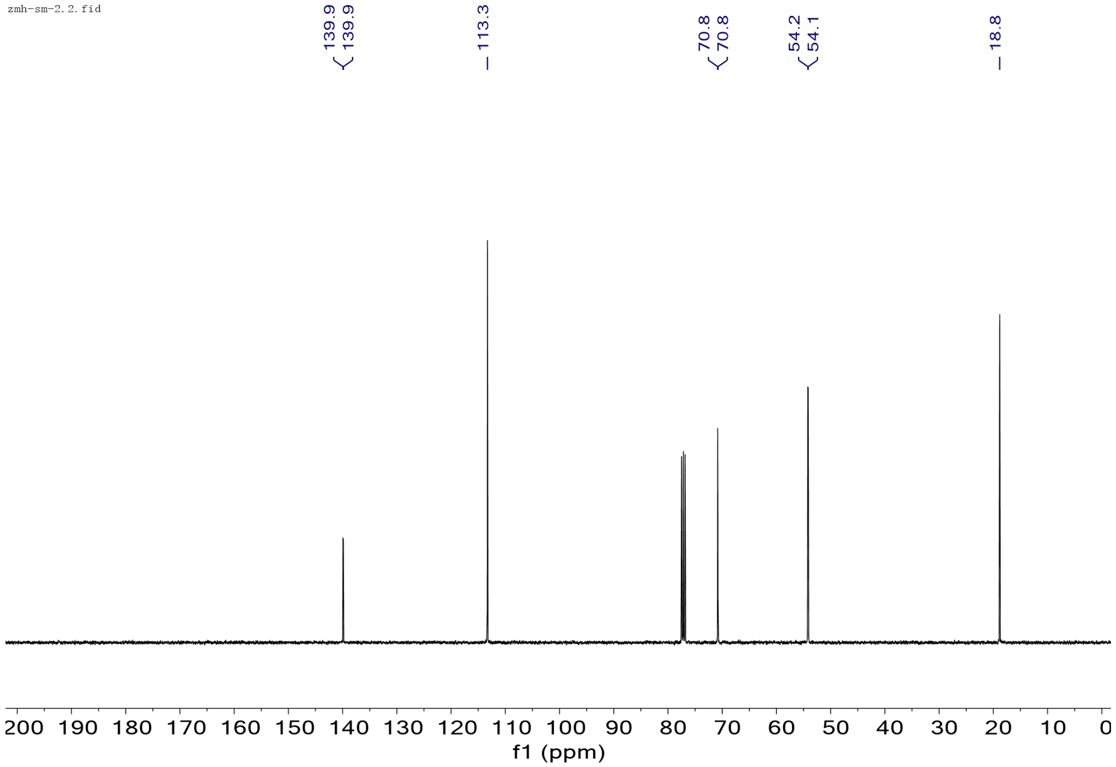
zmh-sm-2.2.fid
139.9
139.9
113.3
70.8
70.8
54.2
54.1
18.8
200 190 180 170 160 150 140 130 120 110 100 90 80 70 60 50 40 30 20 10 0
f1 (ppm)

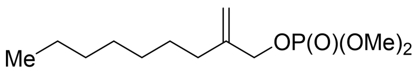
Me
OP(O)(OMe)2

2i

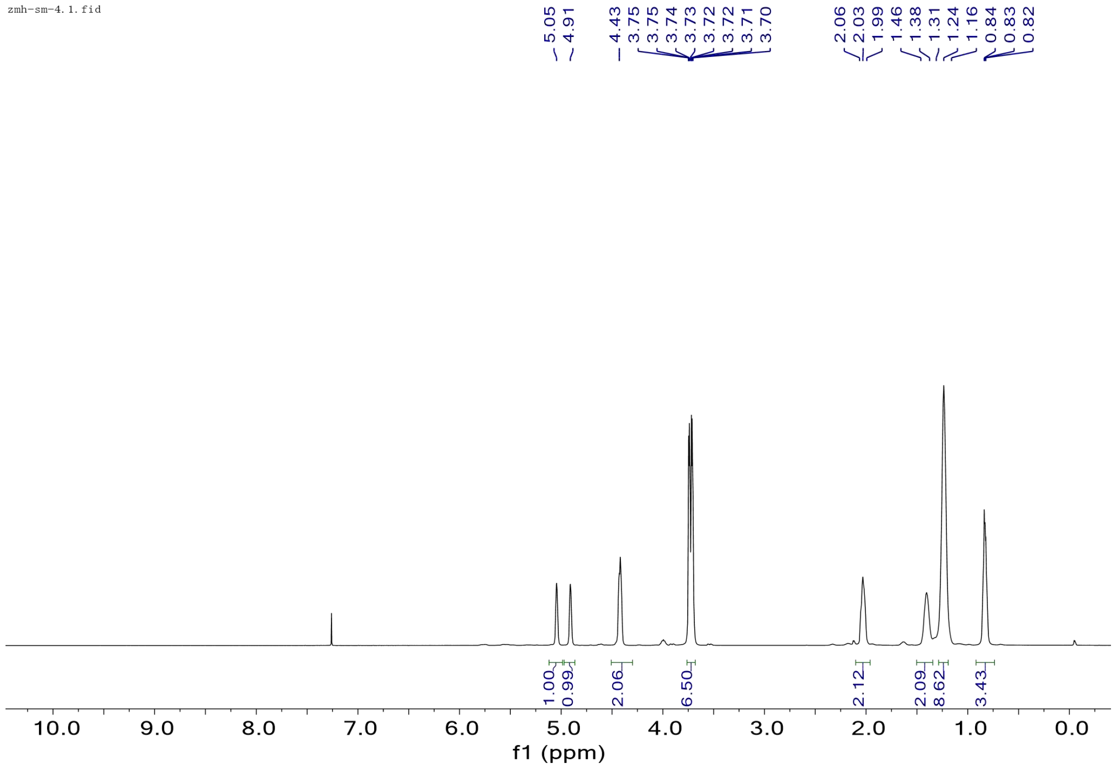
zmh-sm-4.1.fid
5.05
4.91
4.43
3.75
3.74
3.73
3.72
3.72
3.71
3.70
2.06
2.03
1.99
1.46
1.38
1.31
1.24
1.16
0.84
0.83
0.82
1.00
0.99
2.06
6.50
2.12
2.09
8.62
3.43
10.0
9.0
8.0
7.0
6.0
5.0
4.0
3.0
2.0
1.0
0.0
f1 (ppm)

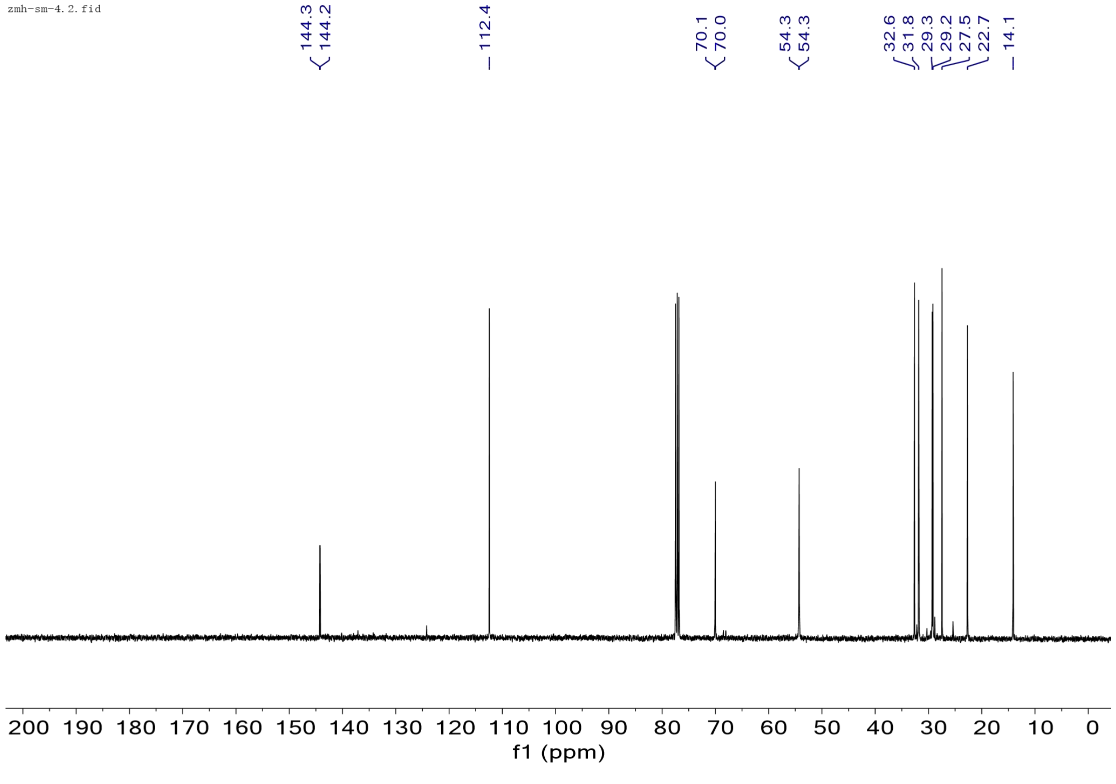
zmh-sm-4.2.fid
144.3
144.2
112.4
70.1
70.0
54.3
54.3
32.6
31.8
29.3
29.2
27.5
22.7
14.1
200
190
180
170
160
150
140
130
120
110
100
90
80
70
60
50
40
30
20
10
0
f1 (ppm)

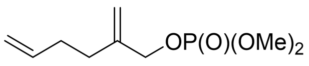
OP(O)(OMe)2

2j

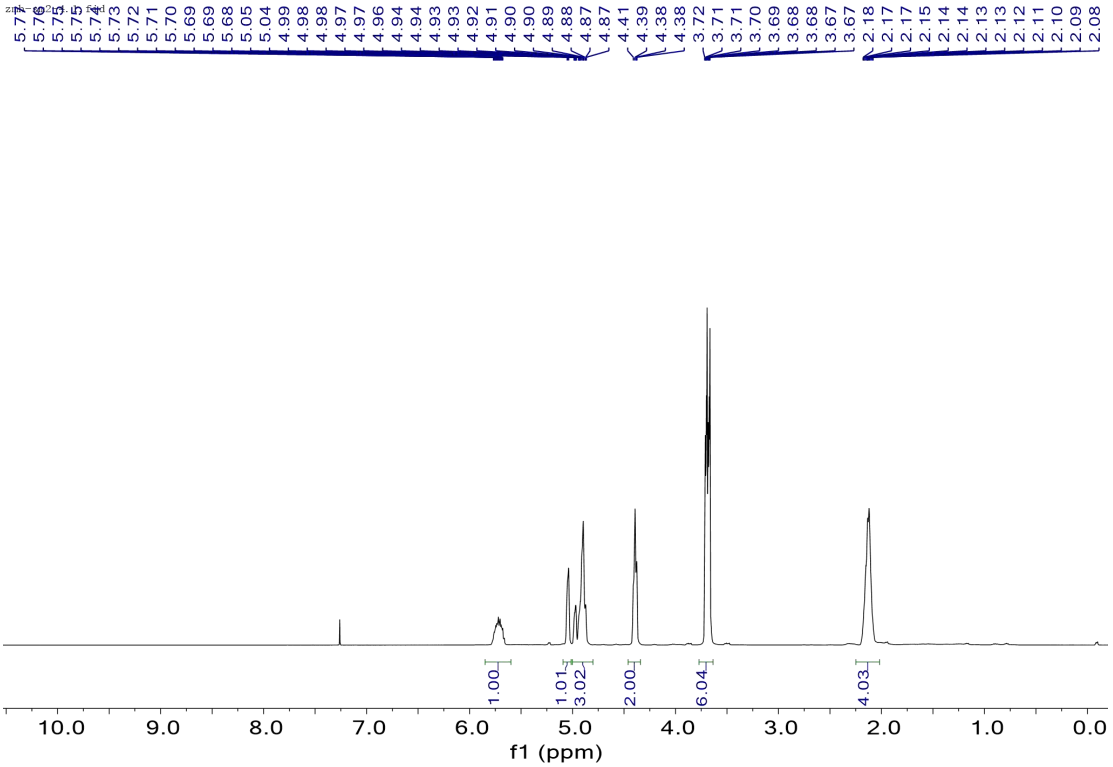
5.77 5.76 5.75 5.75 5.74 5.73 5.72 5.71 5.70 5.69 5.69 5.68 5.05 5.04 4.99 4.98 4.98 4.97 4.97 4.96 4.94 4.94 4.93 4.93 4.92 4.91 4.90 4.90 4.89 4.88 4.87 4.87 4.41 4.39 4.38 4.38 3.72 3.71 3.71 3.70 3.69 3.68 3.68 3.67 3.67 2.18 2.17 2.17 2.15 2.14 2.14 2.13 2.13 2.13 2.12 2.11 2.10 2.09 2.08
1.00
1.01
3.02
2.00
6.04
4.03
10.0 9.0 8.0 7.0 6.0 5.0 4.0 3.0 2.0 1.0 0.0
f1 (ppm)

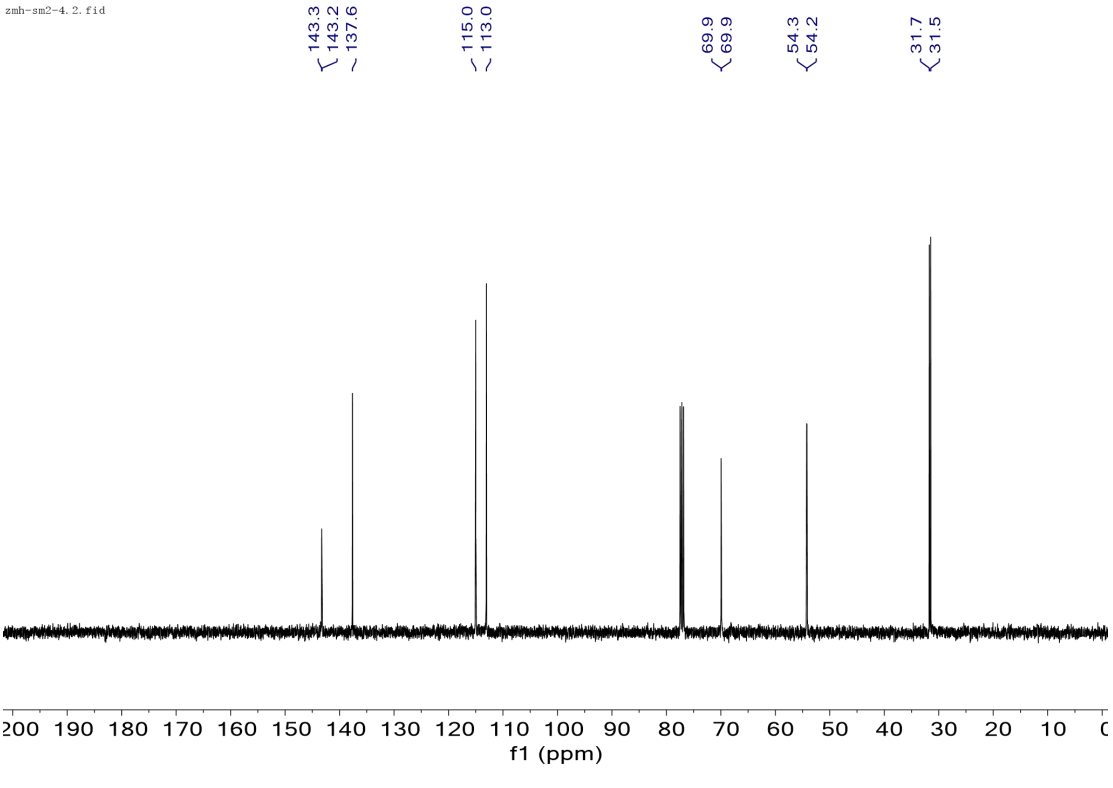
zmh-sm2-4.2.fid
143.3 143.2 137.6
115.0 113.0
69.9 69.9
54.3 54.2
31.7 31.5
200 190 180 170 160 150 140 130 120 110 100 90 80 70 60 50 40 30 20 10
f1 (ppm)

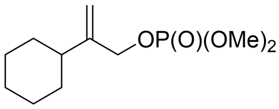
OP(O)(OMe)2

2k

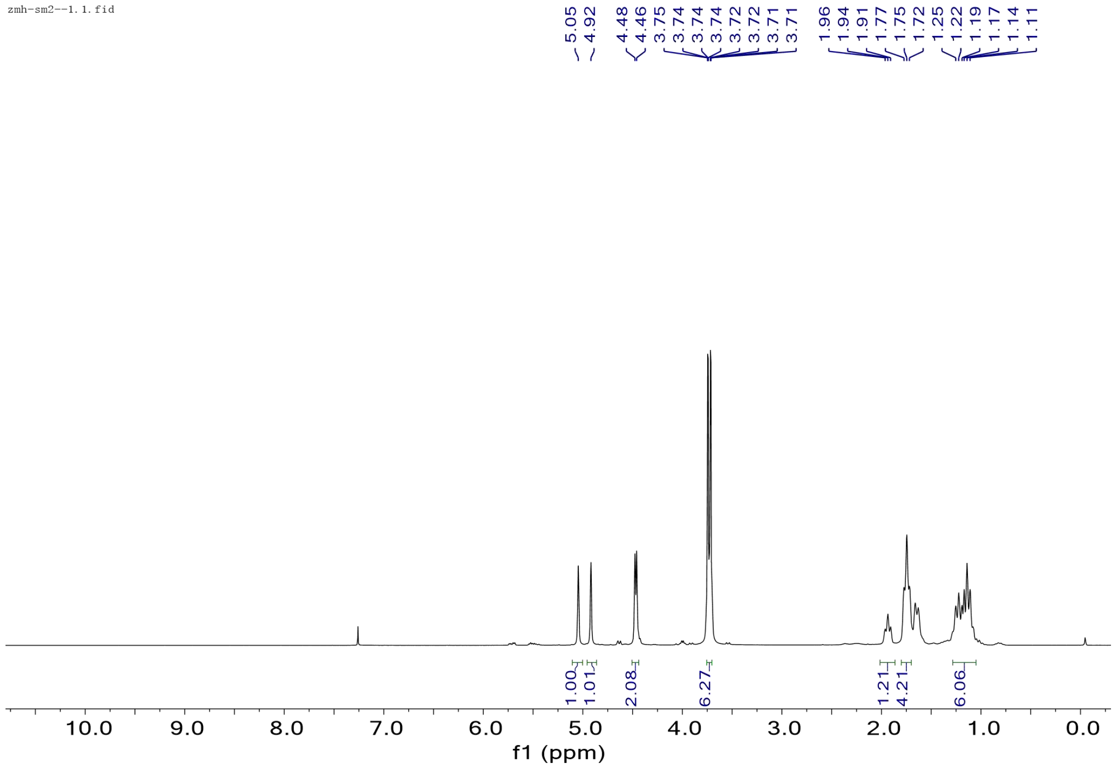
zmh-sm2--1.1.fid
5.05
4.92
4.48
4.46
3.75
3.74
3.74
3.74
3.72
3.72
3.71
3.71
3.71
1.96
1.94
1.91
1.77
1.75
1.72
1.25
1.22
1.19
1.17
1.14
1.11
1.00
1.01
2.08
6.27
1.21
4.21
6.06
10.0 9.0 8.0 7.0 6.0 5.0 4.0 3.0 2.0 1.0 0.0
f1 (ppm)

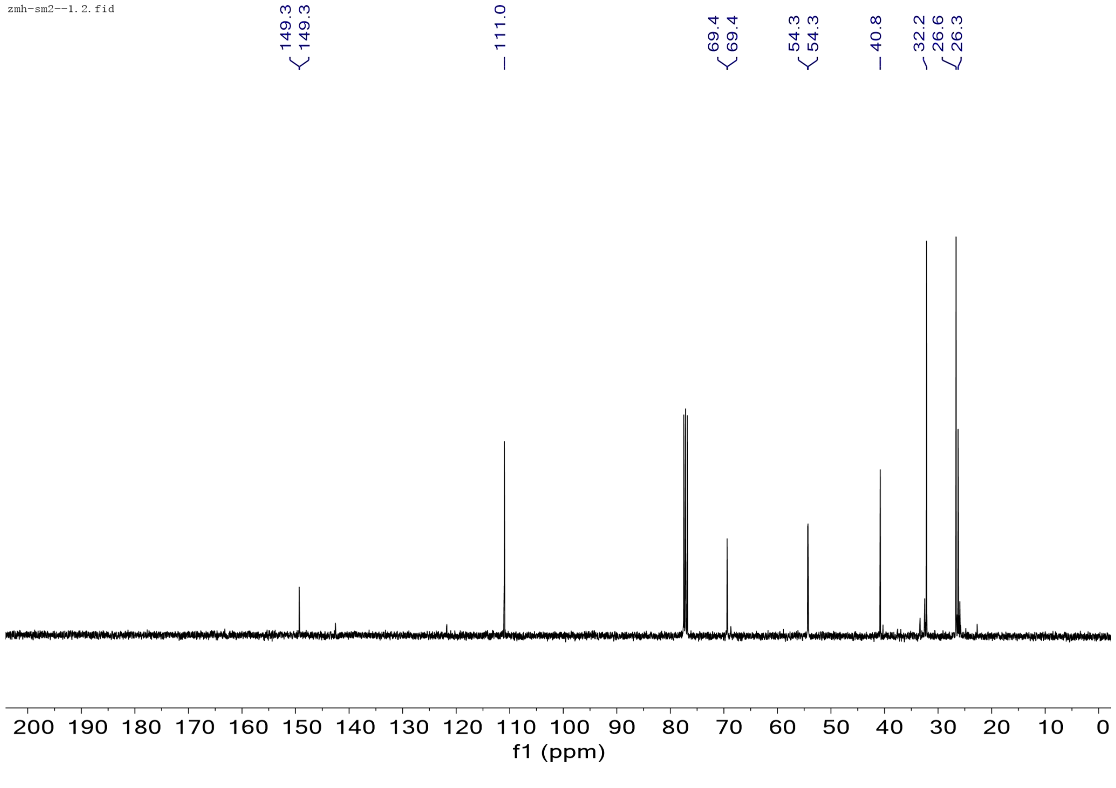
zmh-sm2--1.2.fid
149.3
149.3
111.0
69.4
69.4
54.3
54.3
40.8
32.2
26.6
26.3
200 190 180 170 160 150 140 130 120 110 100 90 80 70 60 50 40 30 20 10 0
f1 (ppm)

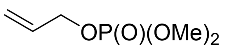
OP(O)(OMe)2

2l

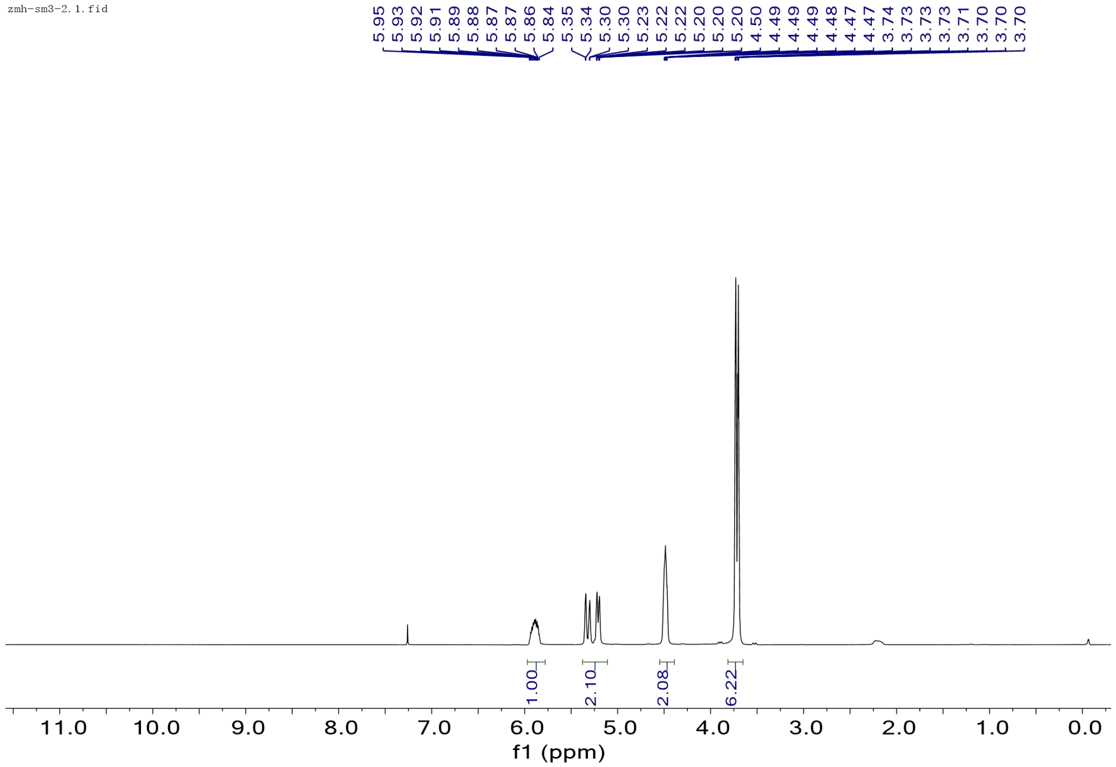
zmh-sm3-2.1.fid
5.95
5.93
5.92
5.91
5.89
5.88
5.87
5.87
5.86
5.84
5.35
5.34
5.30
5.30
5.23
5.22
5.22
5.20
5.20
5.20
4.50
4.49
4.49
4.49
4.48
4.47
4.47
3.74
3.73
3.73
3.73
3.71
3.70
3.70
3.70
1.00
2.10
2.08
6.22
11.0 10.0 9.0 8.0 7.0 6.0 5.0 4.0 3.0 2.0 1.0 0.0
f1 (ppm)

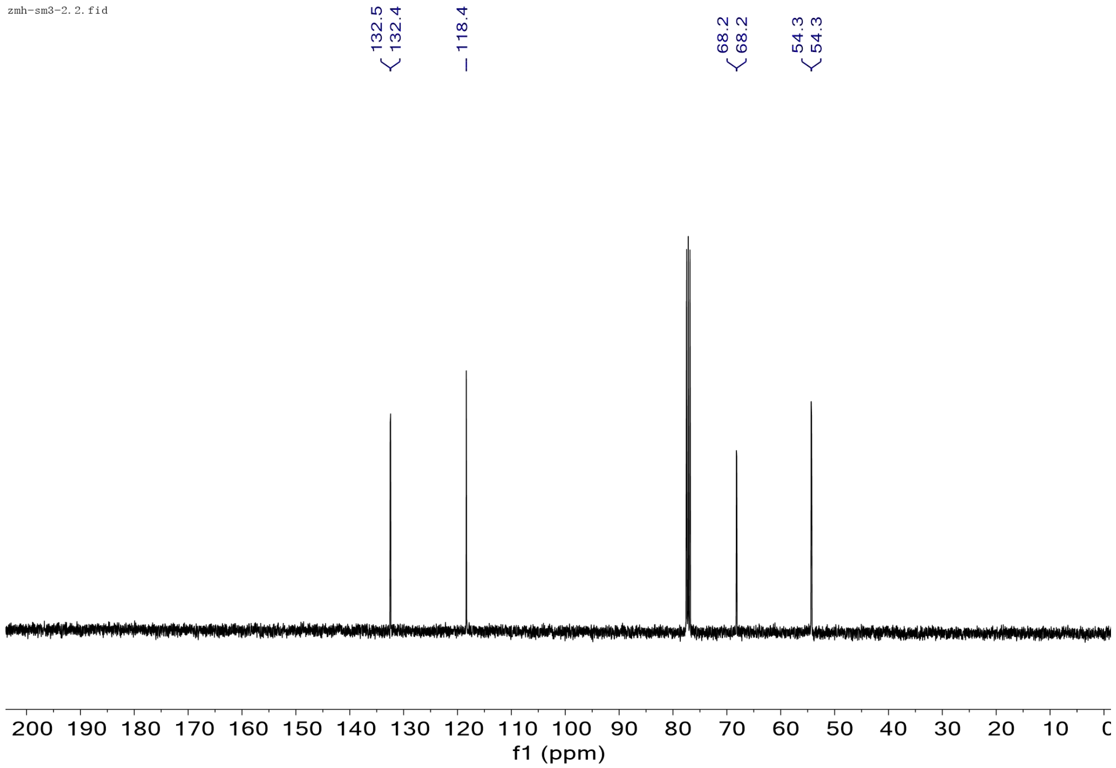
zmh-sm3-2.2.fid
132.5
132.4
118.4
68.2
68.2
54.3
54.3
200 190 180 170 160 150 140 130 120 110 100 90 80 70 60 50 40 30 20 10 0
f1 (ppm)

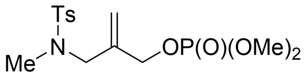
Ts
N
Me
OP(O)(OMe)2

2m

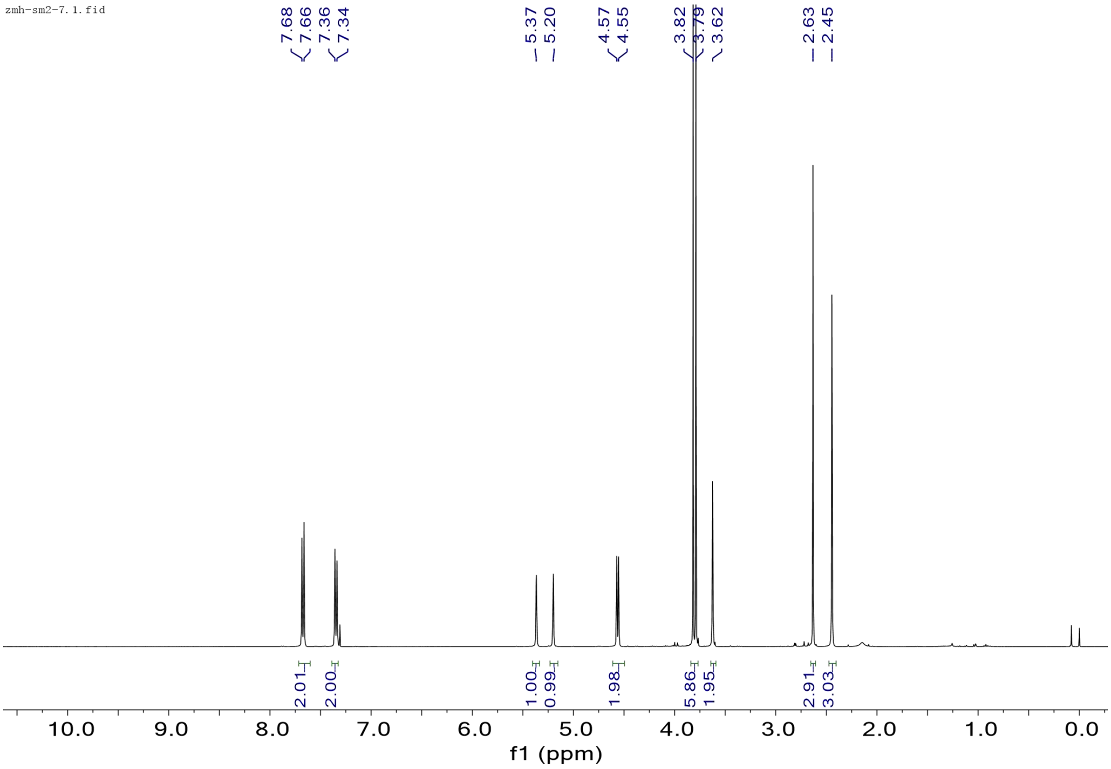
zmh-sm2-7.1.fid
7.68
7.66
7.36
7.34
5.37
5.20
4.57
4.55
3.82
3.79
3.62
2.63
2.45
2.01
2.00
1.00
0.99
1.98
5.86
1.95
2.91
3.03
10.0 9.0 8.0 7.0 6.0 5.0 4.0 3.0 2.0 1.0 0.0
f1 (ppm)

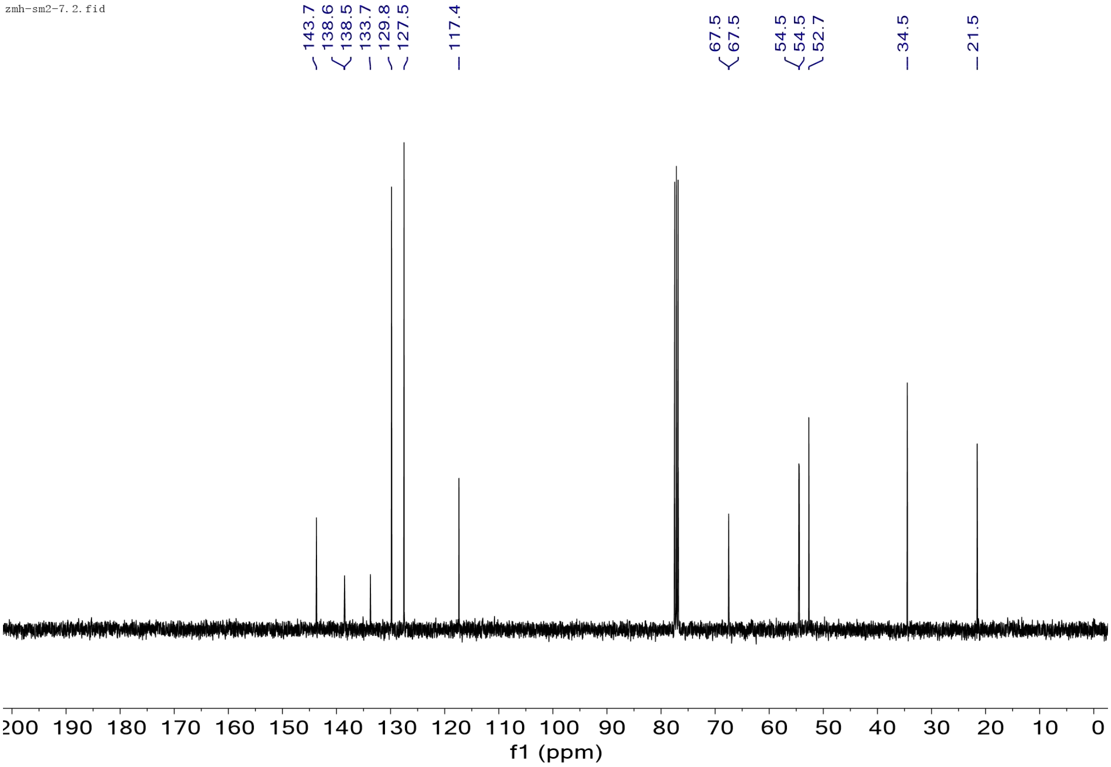
zmh-sm2-7.2.fid
143.7
138.6
138.5
133.7
129.8
127.5
117.4
67.5
67.5
54.5
54.5
52.7
34.5
21.5
200 190 180 170 160 150 140 130 120 110 100 90 80 70 60 50 40 30 20 10 0
f1 (ppm)

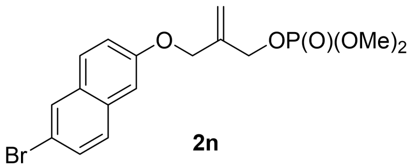

Br
O
OP(O)(OMe)2

2n

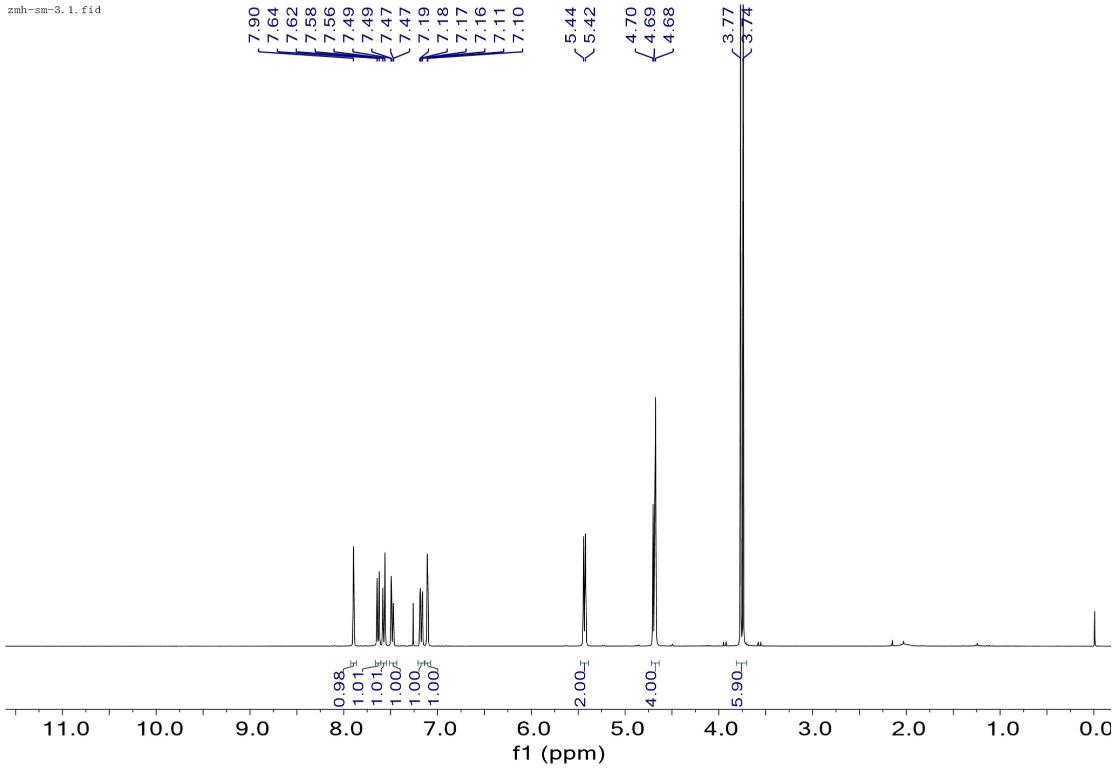

zmh-sm-3.1.fid
7.90 7.64 7.62 7.58 7.56 7.49 7.49 7.47 7.47 7.19 7.18 7.17 7.16 7.11 7.10
5.44 5.42
4.70 4.69 4.68
3.77 3.74
0.98 1.01 1.01 1.00 1.00 1.00
2.00
4.00
5.90
11.0 10.0 9.0 8.0 7.0 6.0 5.0 4.0 3.0 2.0 1.0 0.0
f1 (ppm)

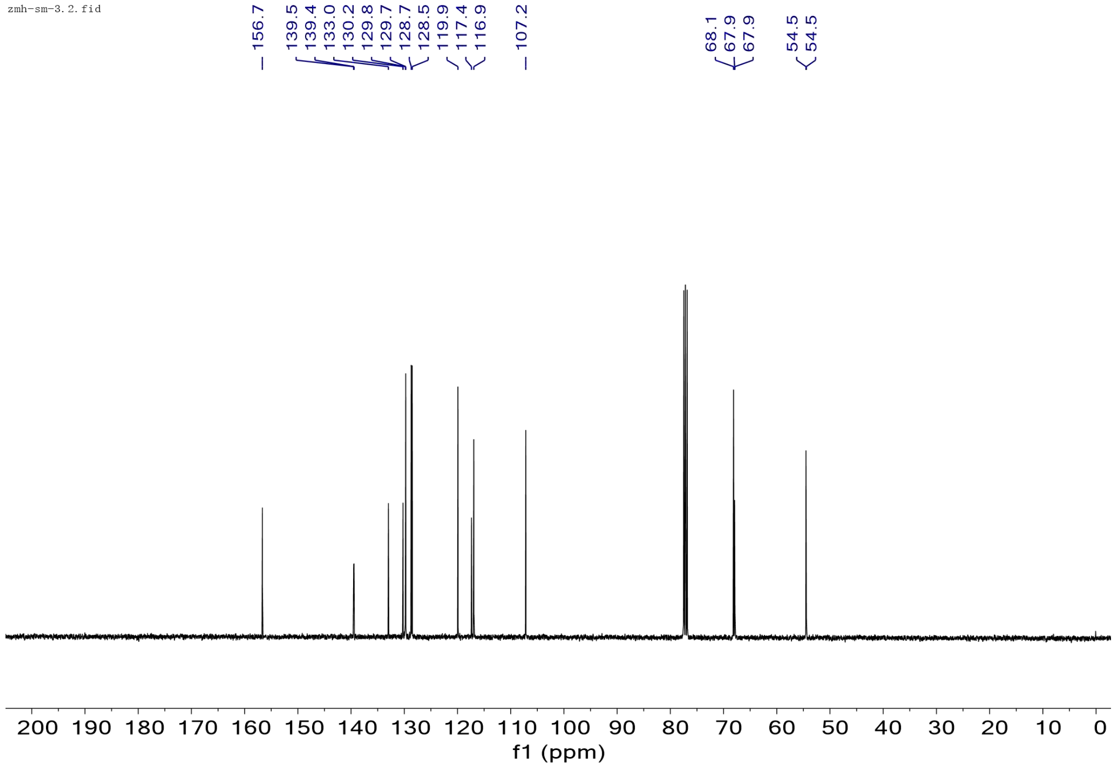

zmh-sm-3.2.fid
156.7
139.5 139.4 133.0 130.2 129.8 129.7 128.7 128.5
119.9 117.4 116.9
107.2
68.1 67.9 67.9
54.5 54.5
200 190 180 170 160 150 140 130 120 110 100 90 80 70 60 50 40 30 20 10 0
f1 (ppm)

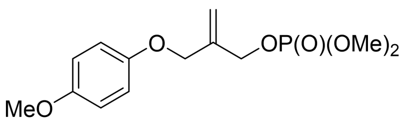
OP(O)(OMe)2
MeO

2o

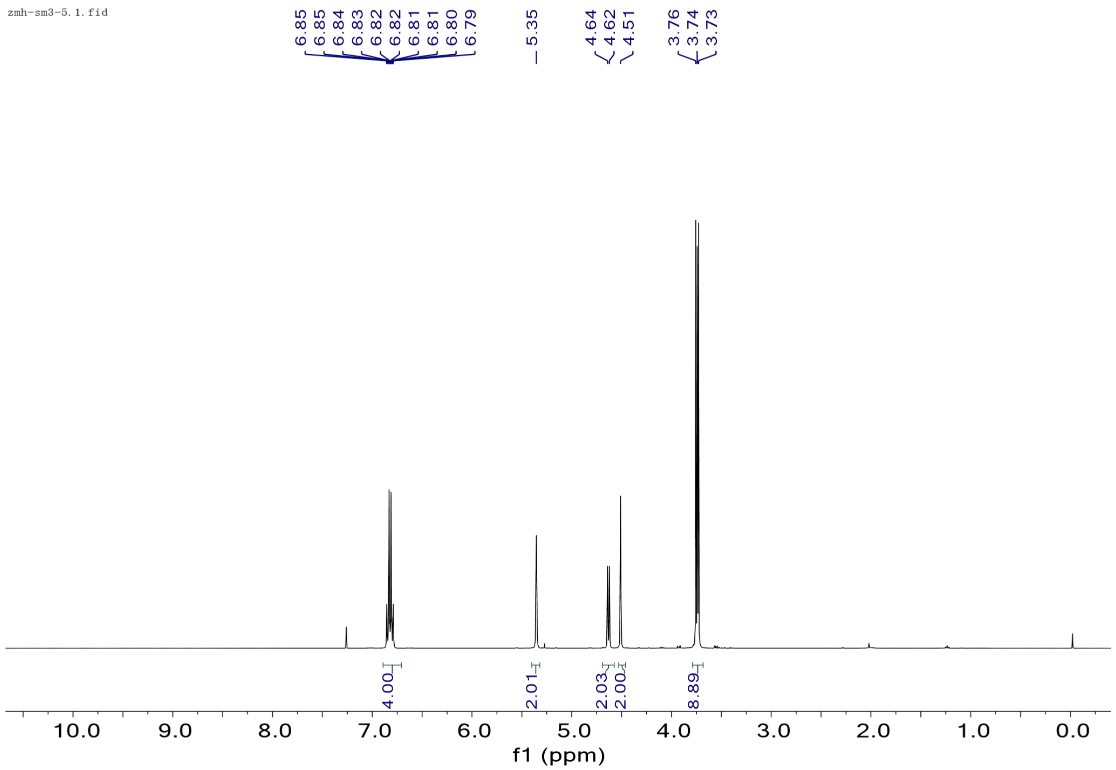
zmh-sm3-5.1.fid
6.85
6.85
6.84
6.83
6.82
6.82
6.81
6.81
6.80
6.79
5.35
4.64
4.62
4.51
3.76
3.74
3.73
4.00
2.01
2.03
2.00
8.89
10.0 9.0 8.0 7.0 6.0 5.0 4.0 3.0 2.0 1.0 0.0
f1 (ppm)

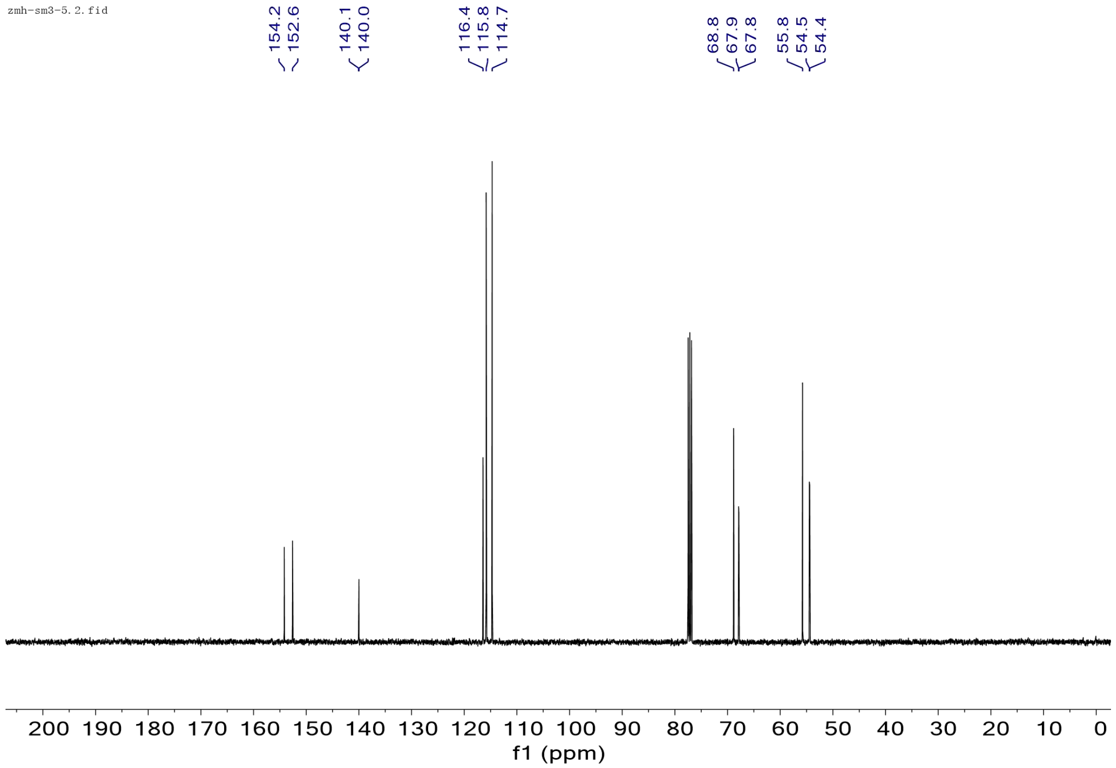
zmh-sm3-5.2.fid
154.2
152.6
140.1
140.0
116.4
115.8
114.7
68.8
67.9
67.8
55.8
54.5
54.4
200 190 180 170 160 150 140 130 120 110 100 90 80 70 60 50 40 30 20 10 0
f1 (ppm)

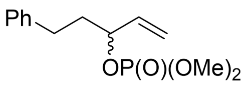
Ph
OP(O)(OMe)2

4a

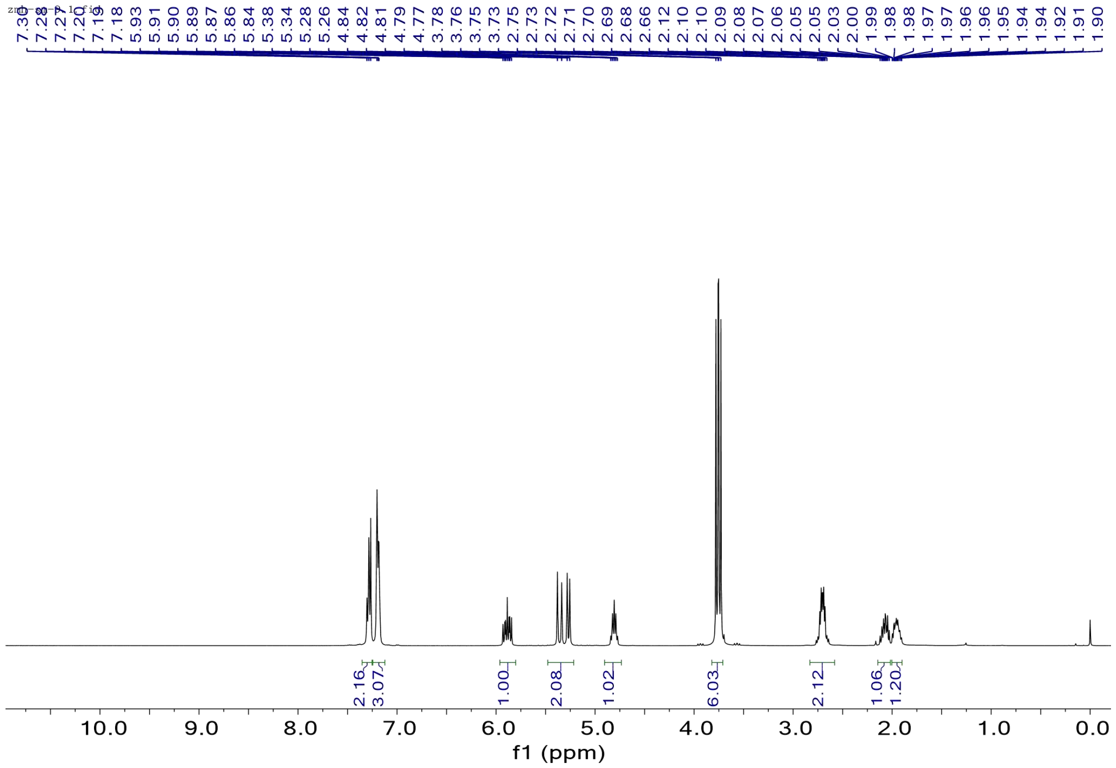
7.30 7.28 7.27 7.20 7.19 7.18 5.93 5.91 5.90 5.89 5.87 5.86 5.84 5.38 5.34 5.28 5.26 4.84 4.82 4.81 4.79 4.77 3.78 3.76 3.75 3.73 2.75 2.73 2.72 2.71 2.70 2.69 2.68 2.66 2.12 2.10 2.10 2.09 2.08 2.07 2.06 2.05 2.05 2.03 2.00 1.99 1.98 1.98 1.97 1.97 1.96 1.95 1.94 1.92 1.91 1.90
2.16 3.07 1.00 2.08 1.02 6.03 2.12 1.06 1.20
10.0 9.0 8.0 7.0 6.0 5.0 4.0 3.0 2.0 1.0 0.0
f1 (ppm)

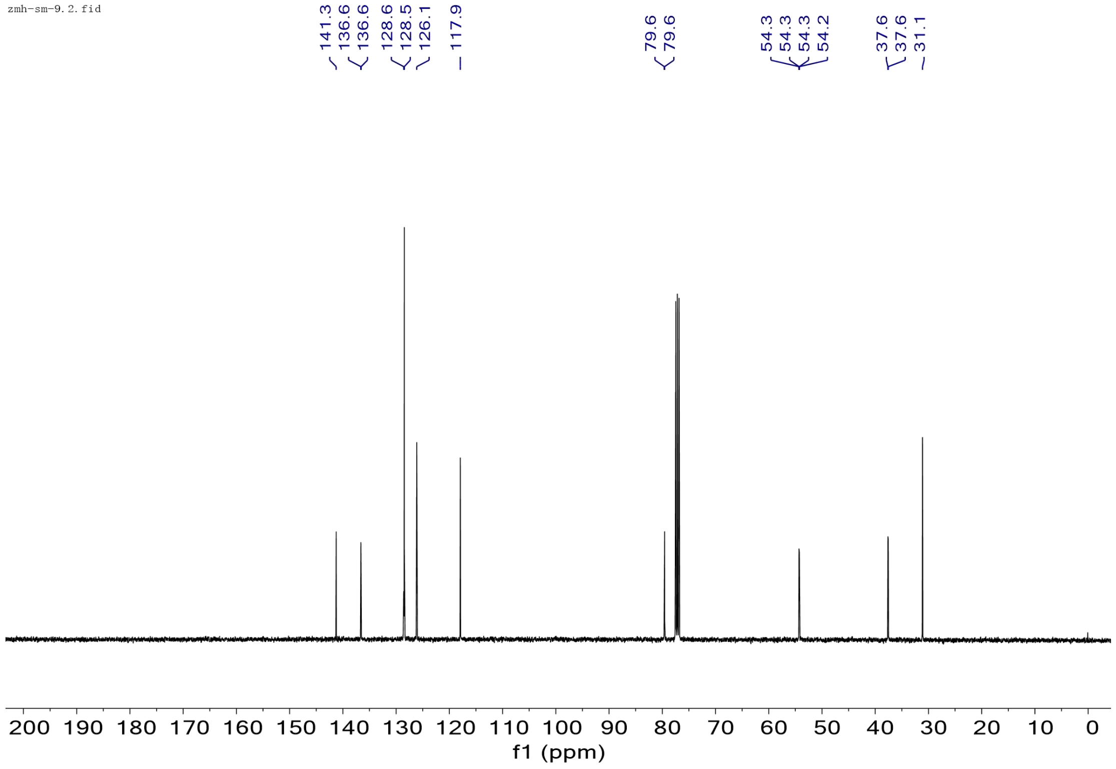
zmh-sm-9.2.fid
141.3 136.6 136.6 128.6 128.5 126.1 117.9
79.6 79.6
54.3 54.3 54.3 54.2
37.6 37.6 31.1
200 190 180 170 160 150 140 130 120 110 100 90 80 70 60 50 40 30 20 10 0
f1 (ppm)

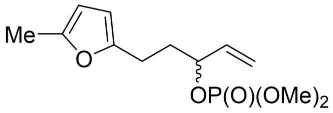
Me
O
OP(O)(OMe)2

4b

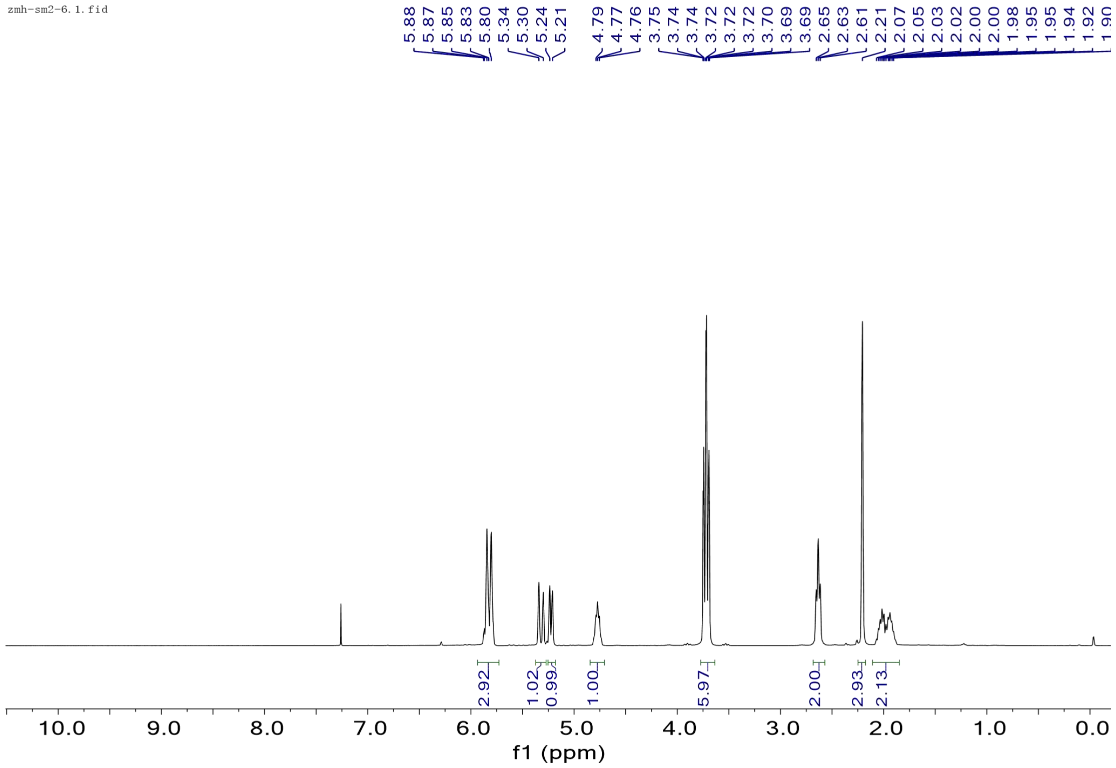
zmh-sm2-6.1.fid
5.88 5.87 5.85 5.83 5.80 5.34 5.30 5.24 5.21 4.79 4.77 4.76 3.75 3.74 3.74 3.72 3.72 3.72 3.70 3.69 3.69 2.65 2.63 2.61 2.21 2.07 2.05 2.03 2.02 2.00 2.00 1.98 1.95 1.95 1.94 1.92 1.90
2.92 1.02 0.99 1.00 5.97 2.00 2.93 2.13
10.0 9.0 8.0 7.0 6.0 5.0 4.0 3.0 2.0 1.0 0.0
f1 (ppm)

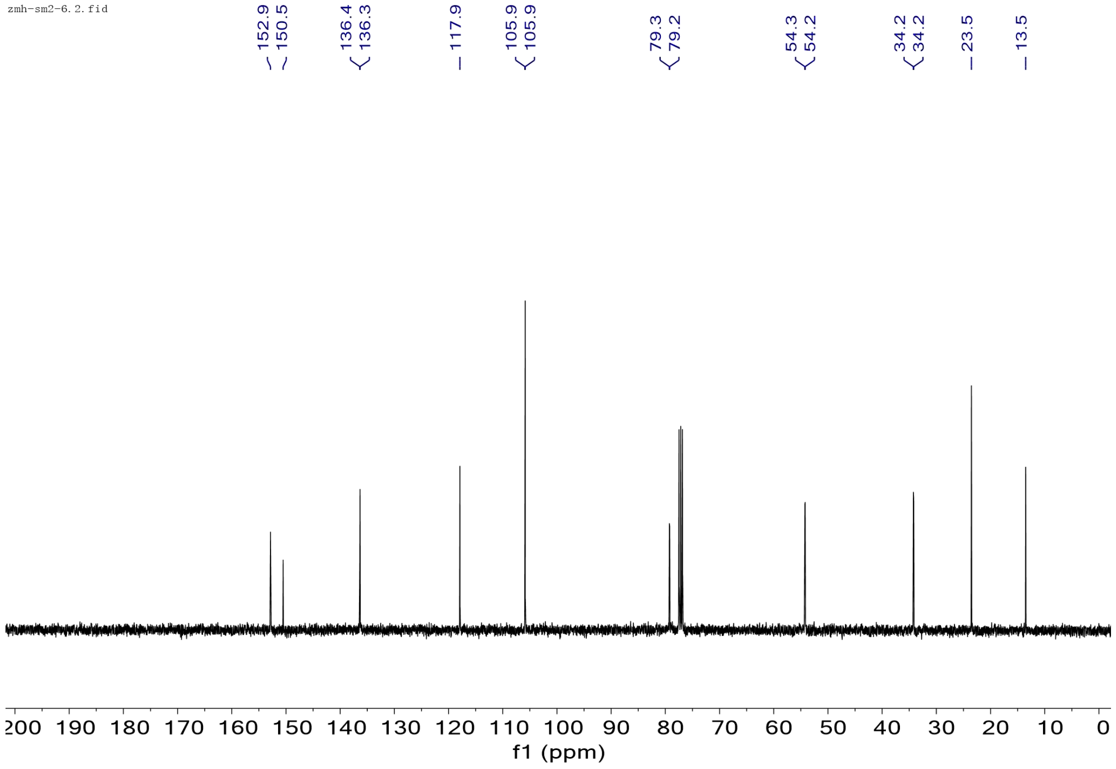
zmh-sm2-6.2.fid
152.9 150.5 136.4 136.3 117.9 105.9 105.9 79.3 79.2 54.3 54.2 34.2 34.2 23.5 13.5
200 190 180 170 160 150 140 130 120 110 100 90 80 70 60 50 40 30 20 10 0
f1 (ppm)

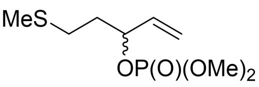

MeS
OP(O)(OMe)2

4c

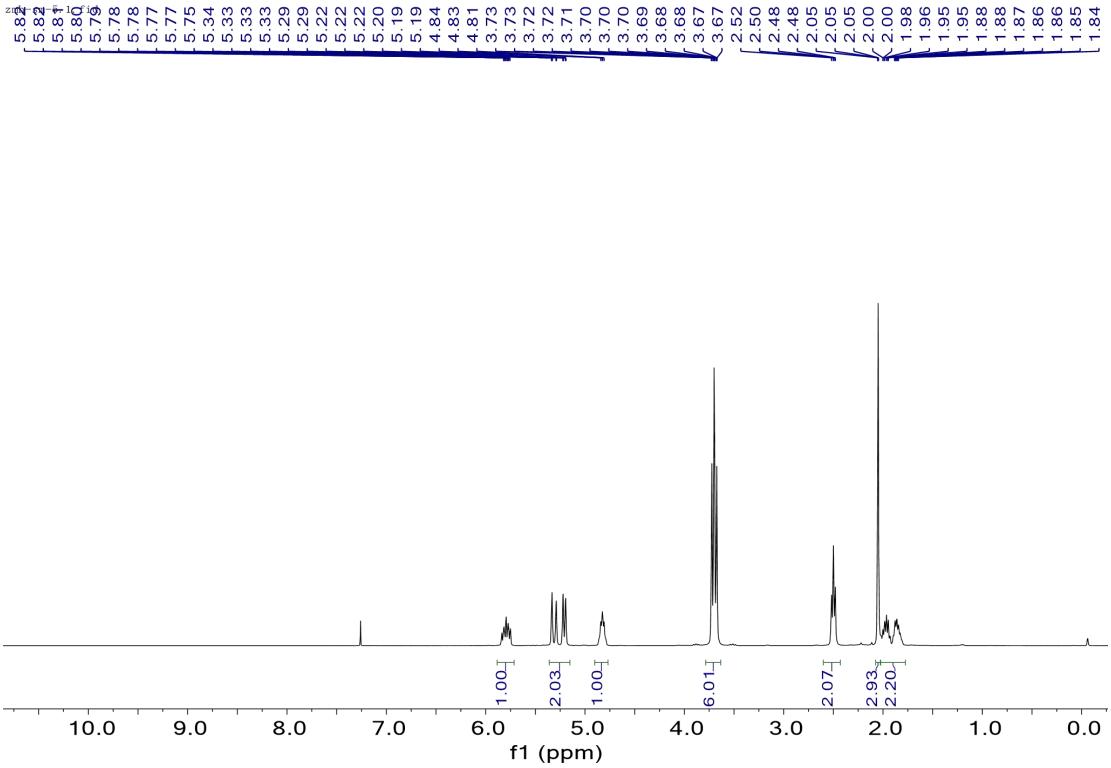

5.82
5.82
5.81
5.80
5.79
5.78
5.78
5.77
5.77
5.75
5.34
5.33
5.33
5.33
5.29
5.29
5.22
5.22
5.22
5.20
5.19
5.19
4.84
4.83
4.81
3.73
3.73
3.72
3.71
3.70
3.70
3.70
3.69
3.68
3.68
3.67
3.67
2.52
2.50
2.48
2.05
2.05
2.05
2.00
2.00
1.98
1.96
1.95
1.95
1.88
1.88
1.87
1.86
1.86
1.85
1.84
1.00
2.03
1.00
6.01
2.07
2.93
2.20
10.0 9.0 8.0 7.0 6.0 5.0 4.0 3.0 2.0 1.0 0.0
f1 (ppm)

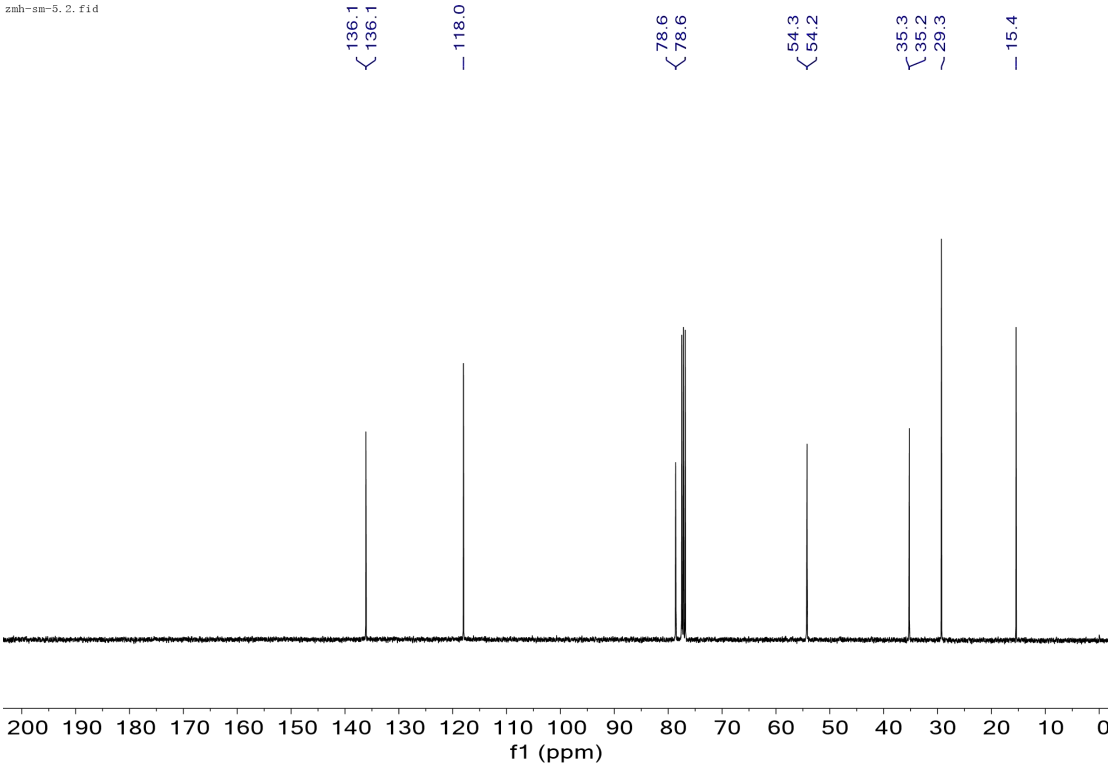

zmh-sm-5.2.fid
136.1
136.1
118.0
78.6
78.6
54.3
54.2
35.3
35.2
29.3
15.4
200 190 180 170 160 150 140 130 120 110 100 90 80 70 60 50 40 30 20 10 0
f1 (ppm)

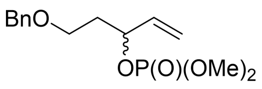
BnO
OP(O)(OMe)2

4d

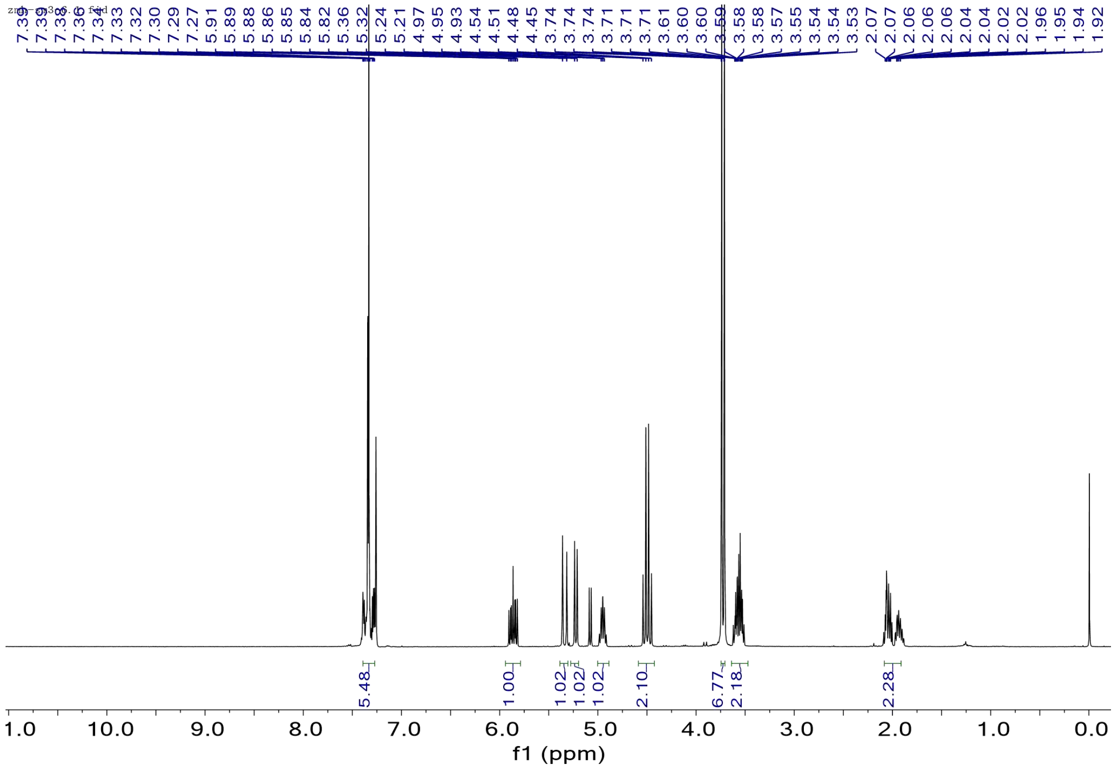
7.39
7.39
7.36
7.36
7.34
7.33
7.32
7.30
7.29
7.27
5.91
5.89
5.88
5.86
5.85
5.84
5.82
5.36
5.32
5.24
5.21
4.97
4.95
4.93
4.54
4.51
4.48
4.45
3.74
3.74
3.74
3.71
3.71
3.71
3.61
3.60
3.60
3.59
3.58
3.58
3.57
3.55
3.54
3.54
3.53
2.07
2.07
2.06
2.06
2.04
2.04
2.02
2.02
1.96
1.95
1.94
1.92
5.48
1.00
1.02
1.02
1.02
2.10
6.77
2.18
2.28
1.0
10.0
9.0
8.0
7.0
6.0
5.0
4.0
3.0
2.0
1.0
0.0
f1 (ppm)

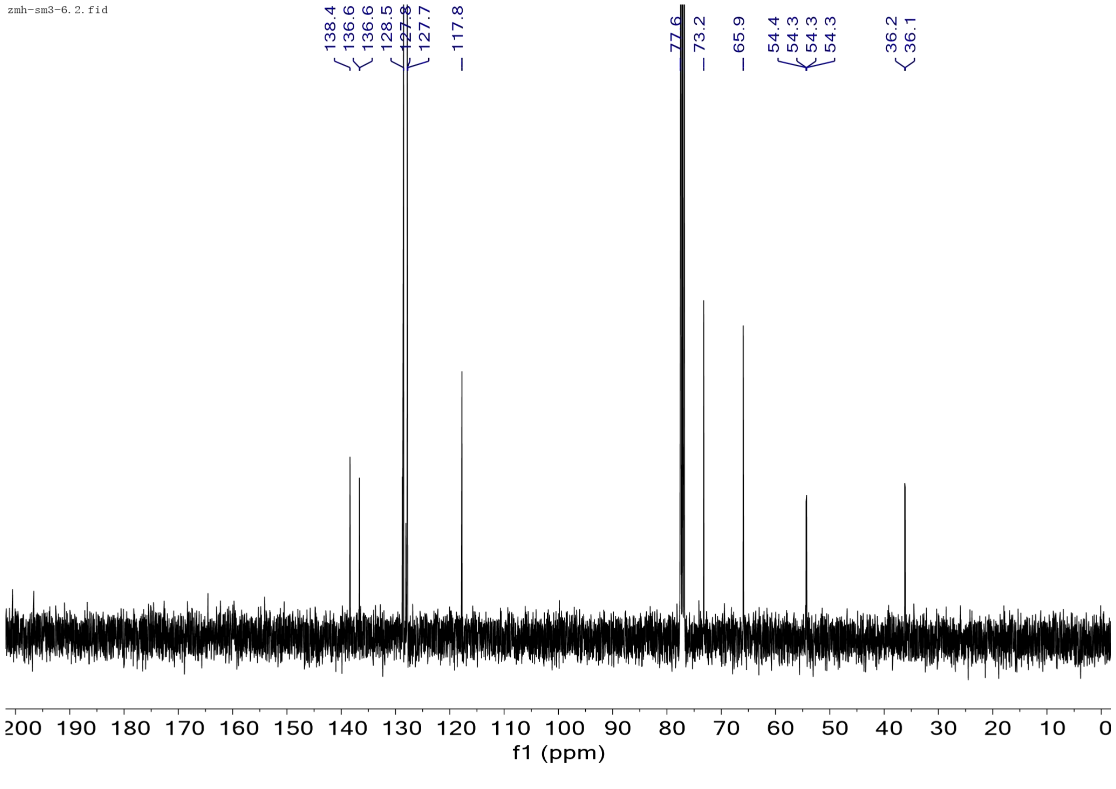
zmh-sm3-6.2.fid
138.4
136.6
136.6
128.5
127.8
127.7
117.8
77.6
73.2
65.9
54.4
54.3
54.3
54.3
36.2
36.1
200
190
180
170
160
150
140
130
120
110
100
90
80
70
60
50
40
30
20
10
0
f1 (ppm)

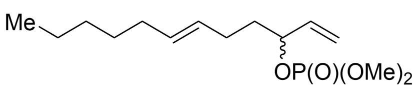
Me
OP(O)(OMe)2

4e

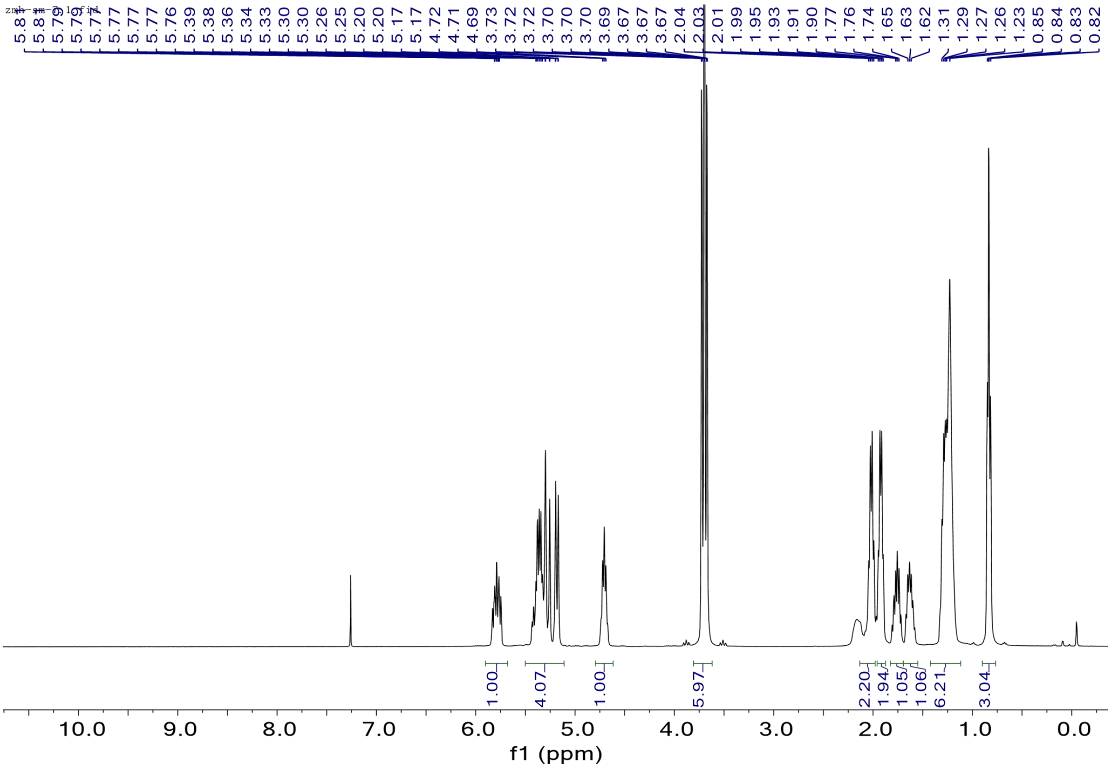
5.77
5.77
5.77
5.76
5.39
5.38
5.36
5.34
5.33
5.30
5.30
5.26
5.25
5.20
5.20
5.17
5.17
4.72
4.71
4.69
3.73
3.72
3.72
3.70
3.70
3.70
3.69
3.67
3.67
3.67
2.04
2.03
2.01
1.99
1.95
1.93
1.91
1.91
1.90
1.77
1.76
1.74
1.65
1.63
1.62
1.31
1.29
1.27
1.26
1.23
0.85
0.84
0.83
0.82
1.00
4.07
1.00
5.97
2.20
1.94
1.05
1.06
6.21
3.04
10.0 9.0 8.0 7.0 6.0 5.0 4.0 3.0 2.0 1.0 0.0
f1 (ppm)

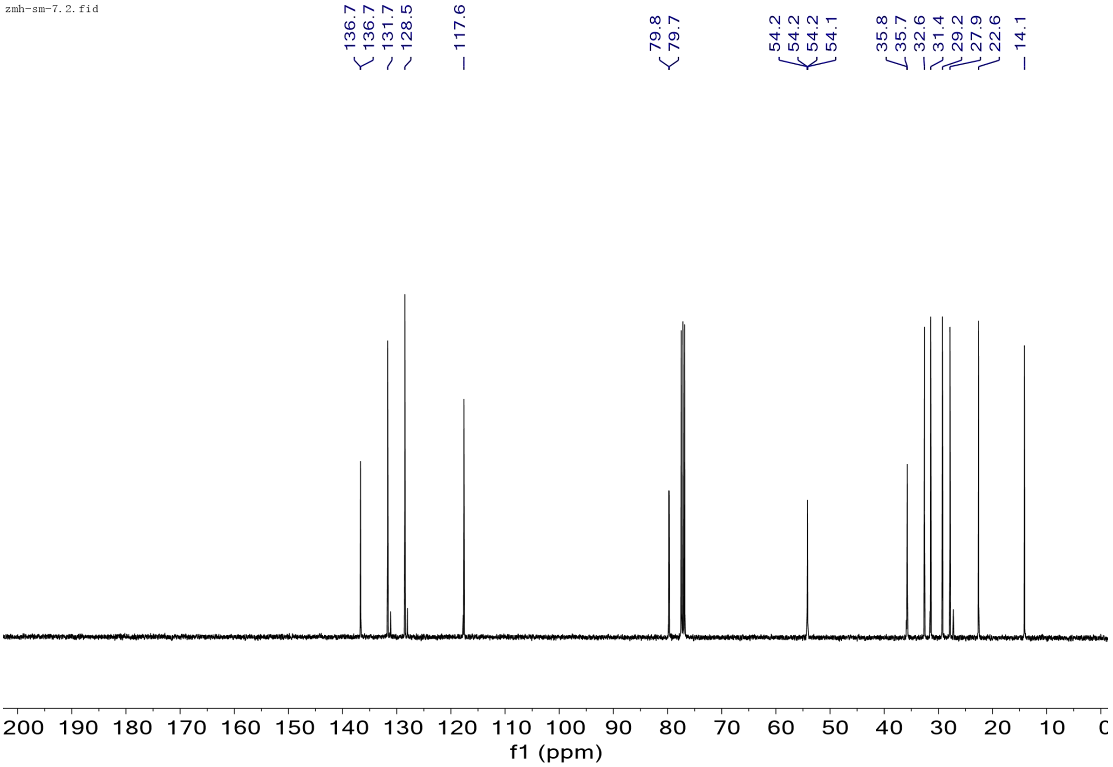
zmh-sm-7.2.fid
136.7
136.7
131.7
128.5
117.6
79.8
79.7
54.2
54.2
54.2
54.1
35.8
35.7
32.6
31.4
29.2
27.9
22.6
14.1
200 190 180 170 160 150 140 130 120 110 100 90 80 70 60 50 40 30 20 10
f1 (ppm)

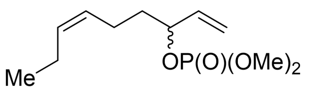
Me
OP(O)(OMe)$_2$

4f

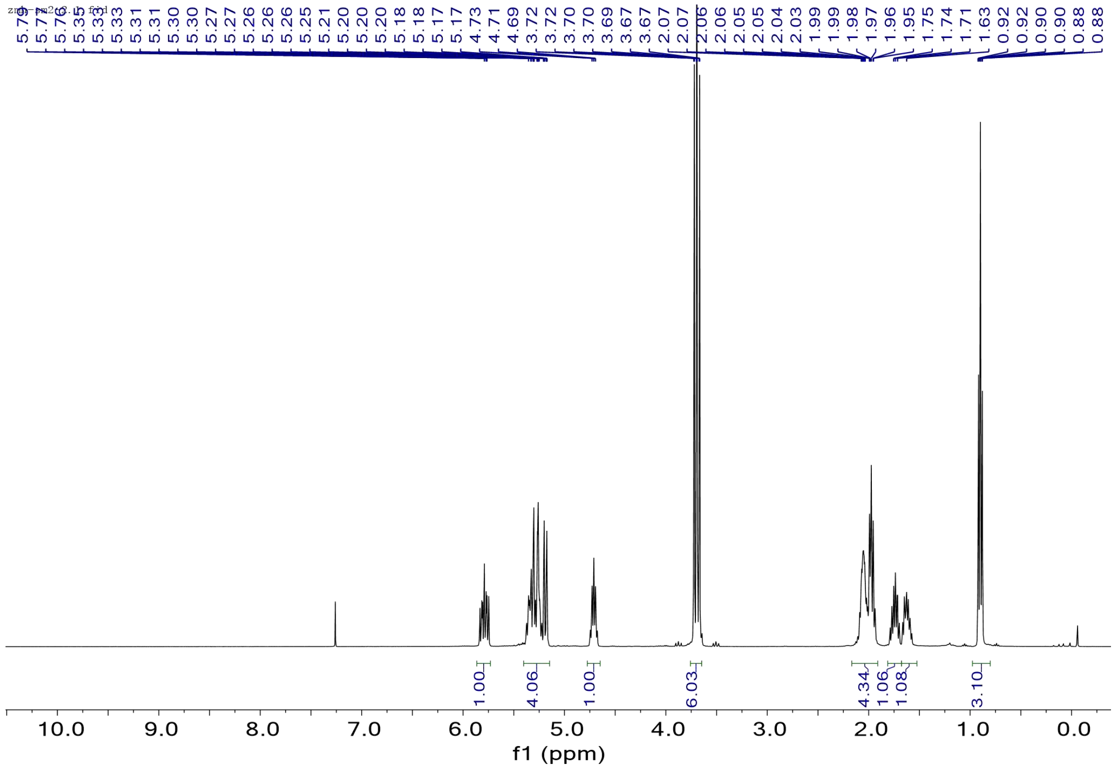
zmh-sm2-2.1.fid
5.79 5.77 5.76 5.35 5.33 5.33 5.31 5.31 5.30 5.30 5.27 5.27 5.26 5.26 5.25 5.21 5.20 5.20 5.20 5.18 5.18 5.17 5.17 4.73 4.71 4.69 3.72 3.72 3.70 3.70 3.69 3.67 3.67 2.07 2.07 2.06 2.06 2.05 2.05 2.04 2.03 1.99 1.99 1.98 1.97 1.96 1.95 1.75 1.74 1.71 1.63 0.92 0.92 0.90 0.90 0.88 0.88
1.00 4.06 1.00 6.03 4.34 1.06 1.08 3.10
10.0 9.0 8.0 7.0 6.0 5.0 4.0 3.0 2.0 1.0 0.0
f1 (ppm)

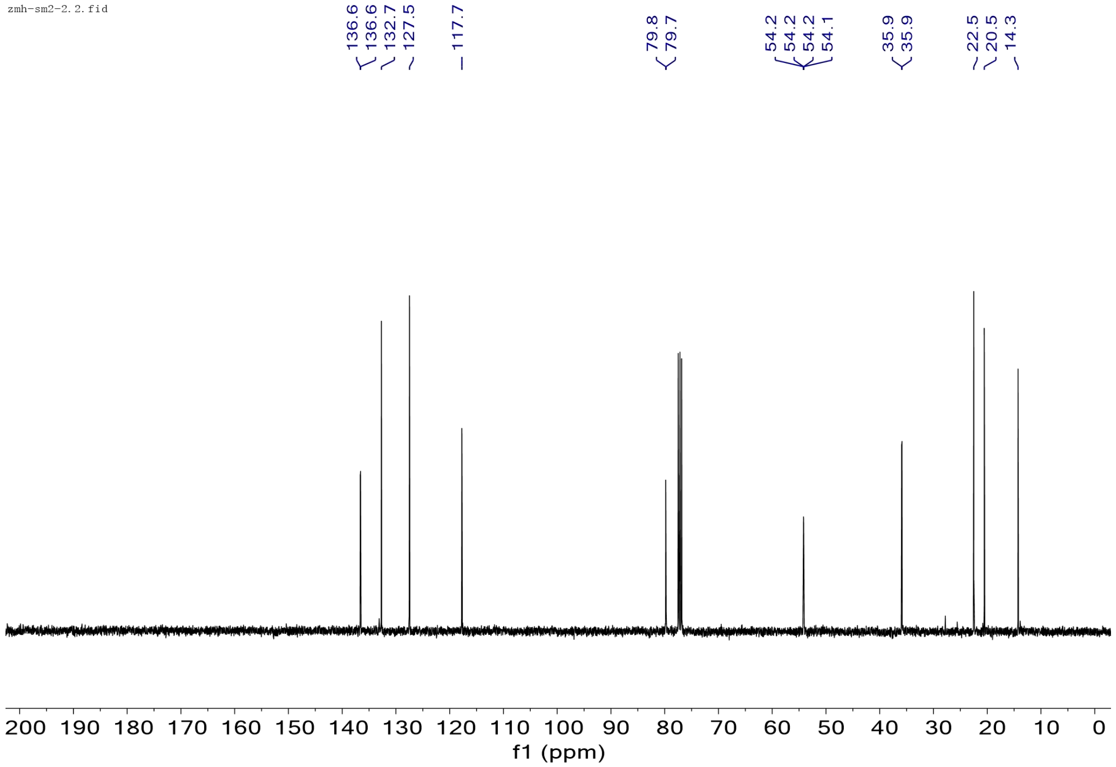
zmh-sm2-2.2.fid
136.6 136.6 132.7 127.5 117.7 79.8 79.7 54.2 54.2 54.2 54.1 35.9 35.9 22.5 20.5 14.3
200 190 180 170 160 150 140 130 120 110 100 90 80 70 60 50 40 30 20 10 0
f1 (ppm)

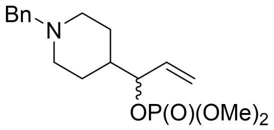
Bn
N
OP(O)(OMe)2

4g

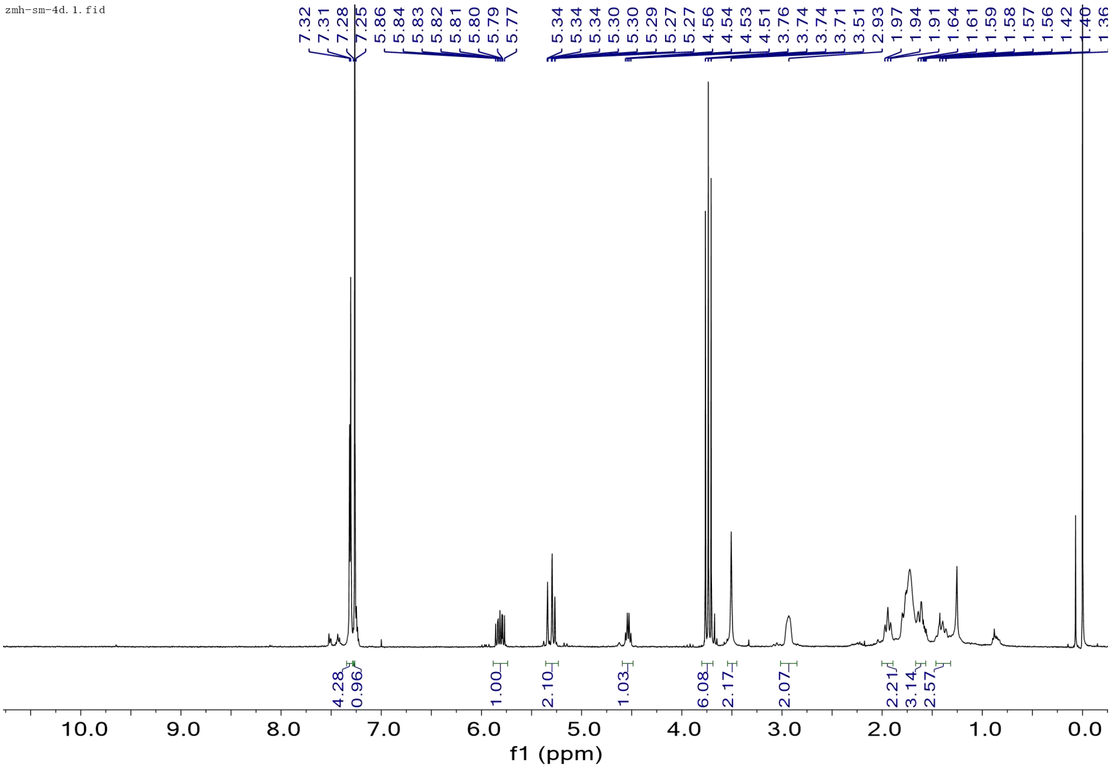
zmh-sm-4d.1.fid
7.32
7.31
7.28
7.25
5.86
5.84
5.83
5.82
5.81
5.80
5.79
5.77
5.34
5.34
5.34
5.30
5.30
5.29
5.27
5.27
4.56
4.54
4.53
4.51
3.76
3.74
3.74
3.71
3.51
2.93
1.97
1.94
1.91
1.64
1.61
1.59
1.58
1.57
1.56
1.42
1.40
1.36
4.28
0.96
1.00
2.10
1.03
6.08
2.17
2.07
2.21
3.14
2.57
10.0 9.0 8.0 7.0 6.0 5.0 4.0 3.0 2.0 1.0 0.0
f1 (ppm)

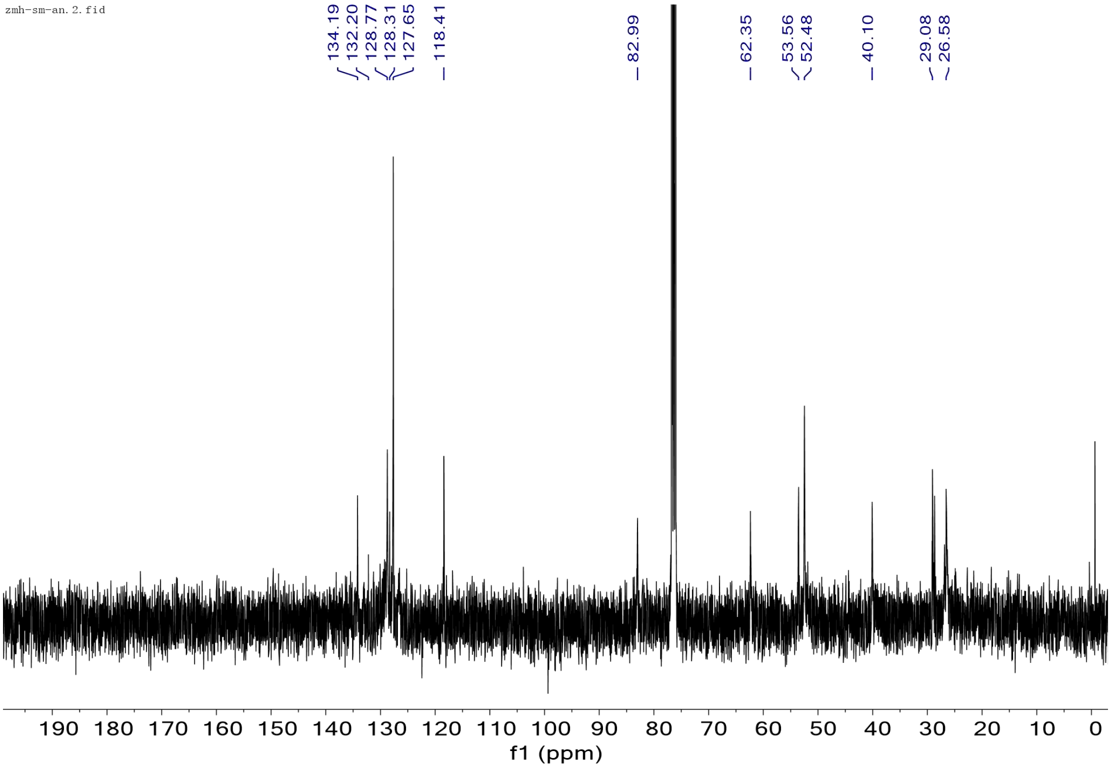
zmh-sm-an.2.fid
134.19
132.20
128.77
128.31
127.65
118.41
82.99
62.35
53.56
52.48
40.10
29.08
26.58
190 180 170 160 150 140 130 120 110 100 90 80 70 60 50 40 30 20 10 0
f1 (ppm)

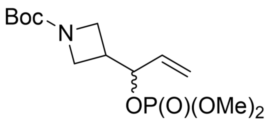
Boc
N
OP(O)(OMe)2

4h

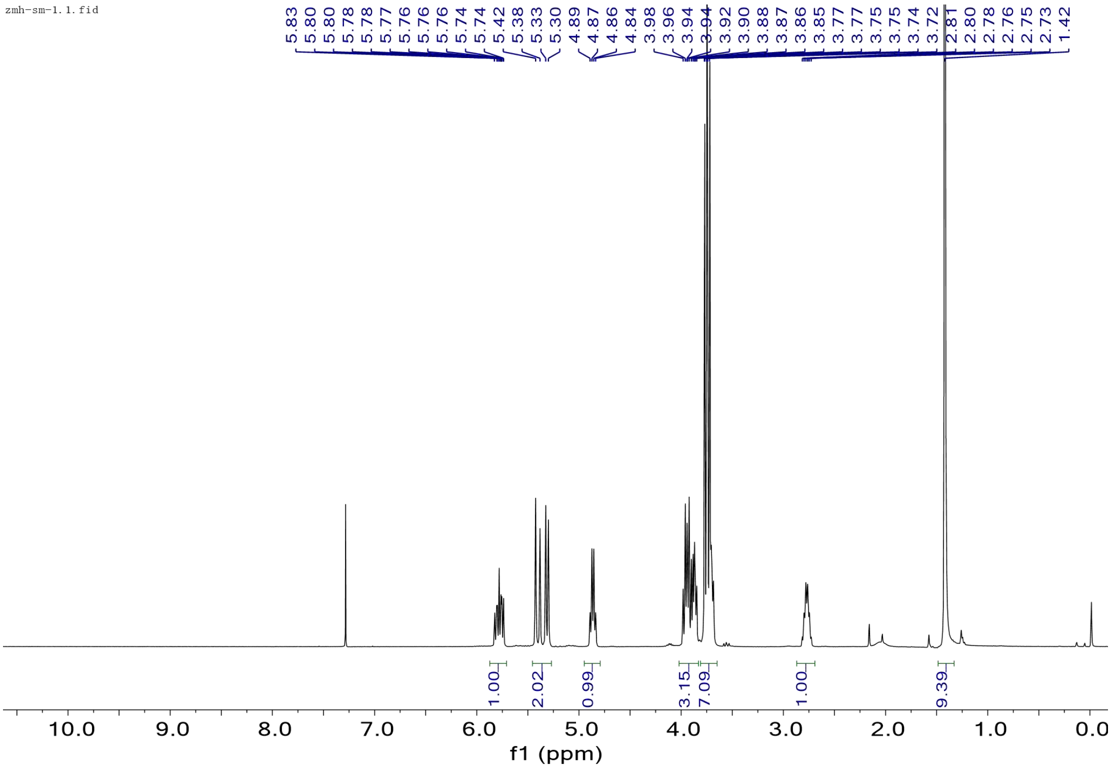
zmh-sm-1.1.fid
5.83 5.80 5.80 5.78 5.78 5.77 5.76 5.76 5.76 5.74 5.74 5.42 5.38 5.33 5.30 4.89 4.87 4.86 4.84 3.98 3.96 3.94 3.94 3.92 3.90 3.88 3.87 3.86 3.85 3.77 3.77 3.75 3.75 3.74 3.72 2.81 2.80 2.78 2.76 2.75 2.73 1.42
1.00 2.02 0.99 3.15 7.09 1.00 9.39
10.0 9.0 8.0 7.0 6.0 5.0 4.0 3.0 2.0 1.0 0.0
f1 (ppm)

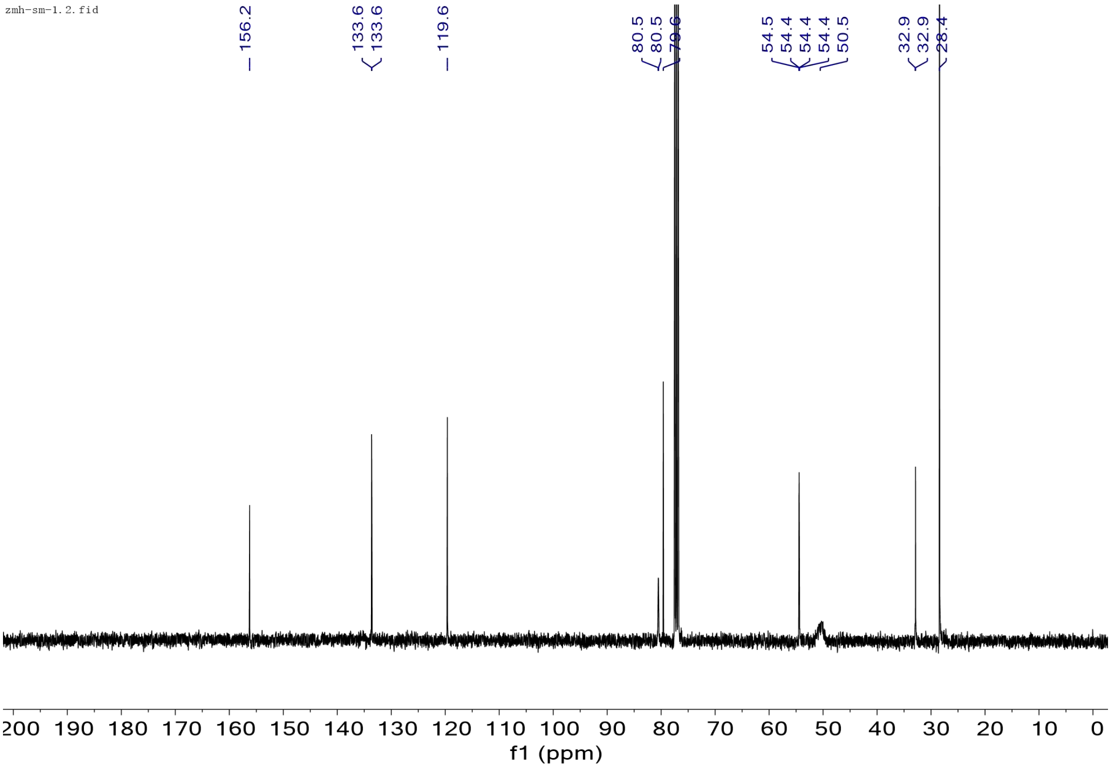
zmh-sm-1.2.fid
156.2
133.6 133.6
119.6
80.5 80.5 79.6
54.5 54.4 54.4 54.4 50.5
32.9 32.9 28.4
200 190 180 170 160 150 140 130 120 110 100 90 80 70 60 50 40 30 20 10 0
f1 (ppm)

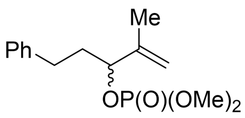
Me
Ph
OP(O)(OMe)2

4i

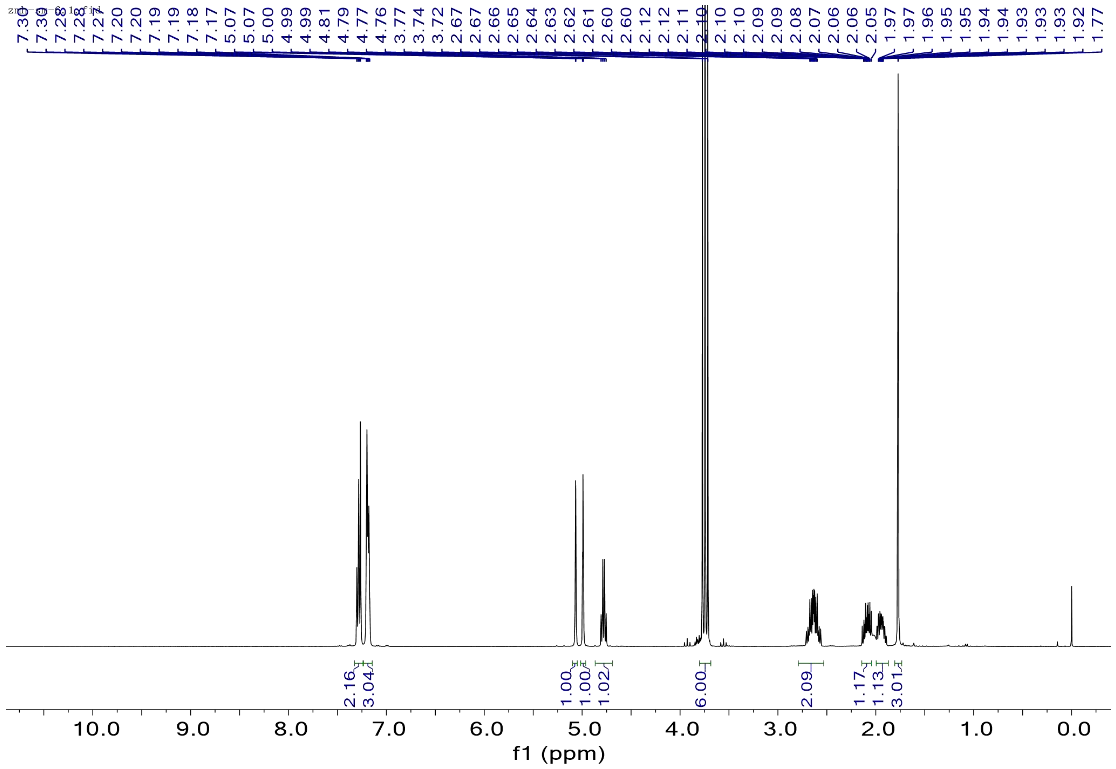
7.30
7.30
7.28
7.28
7.21
7.20
7.20
7.19
7.19
7.18
7.17
5.07
5.07
5.00
4.99
4.99
4.81
4.79
4.77
4.76
3.77
3.74
3.72
2.67
2.67
2.66
2.65
2.64
2.63
2.62
2.61
2.60
2.60
2.12
2.12
2.11
2.10
2.10
2.10
2.09
2.09
2.08
2.07
2.06
2.06
2.05
1.97
1.96
1.95
1.95
1.94
1.94
1.93
1.93
1.93
1.92
1.77
2.16
3.04
1.00
1.00
1.02
6.00
2.09
1.17
1.13
3.01
10.0
9.0
8.0
7.0
6.0
5.0
4.0
3.0
2.0
1.0
0.0
f1 (ppm)

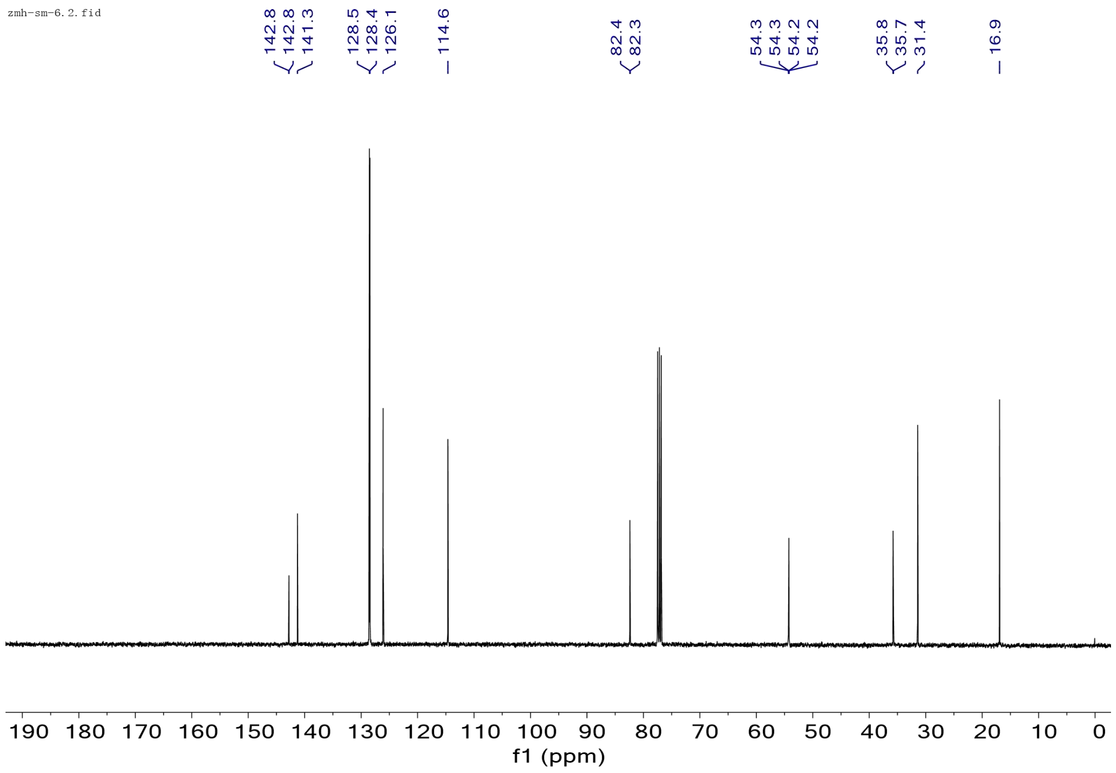
zmh-sm-6.2.fid
142.8
142.8
141.3
128.5
128.4
126.1
114.6
82.4
82.3
54.3
54.3
54.2
54.2
35.8
35.7
31.4
16.9
190
180
170
160
150
140
130
120
110
100
90
80
70
60
50
40
30
20
10
0
f1 (ppm)

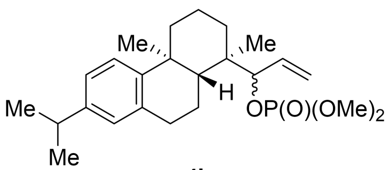
Me
Me
H
Me
Me
OP(O)(OMe)2

4j

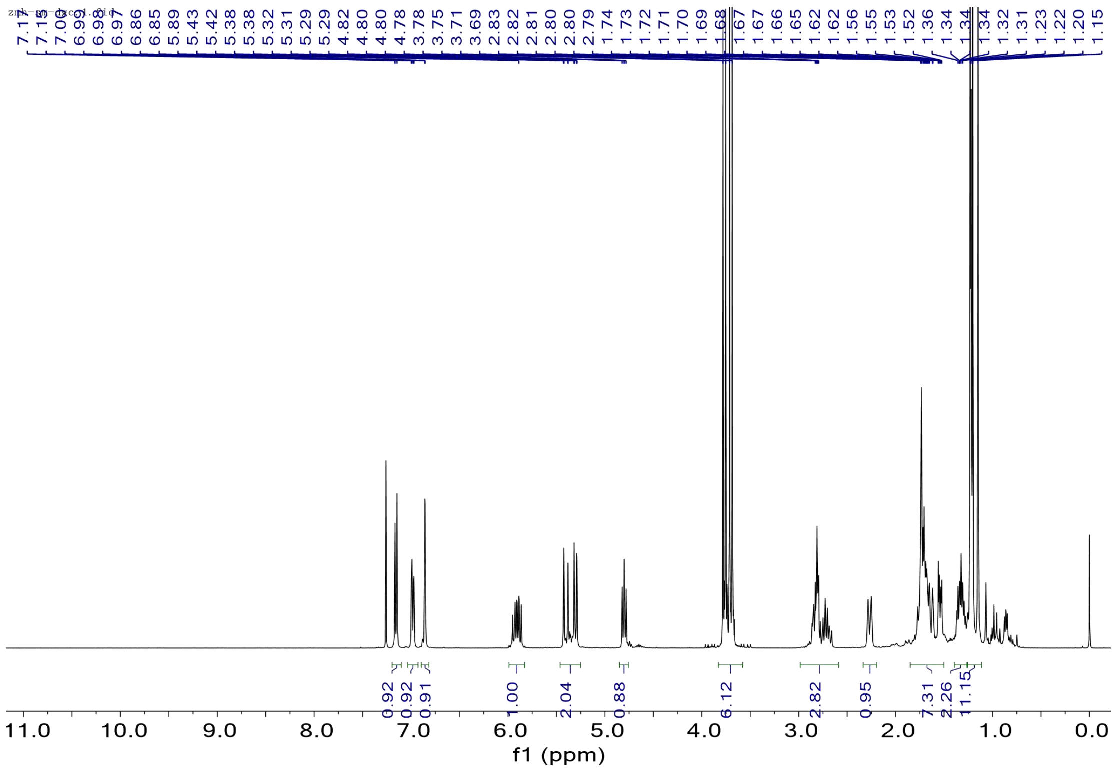
f1 (ppm)

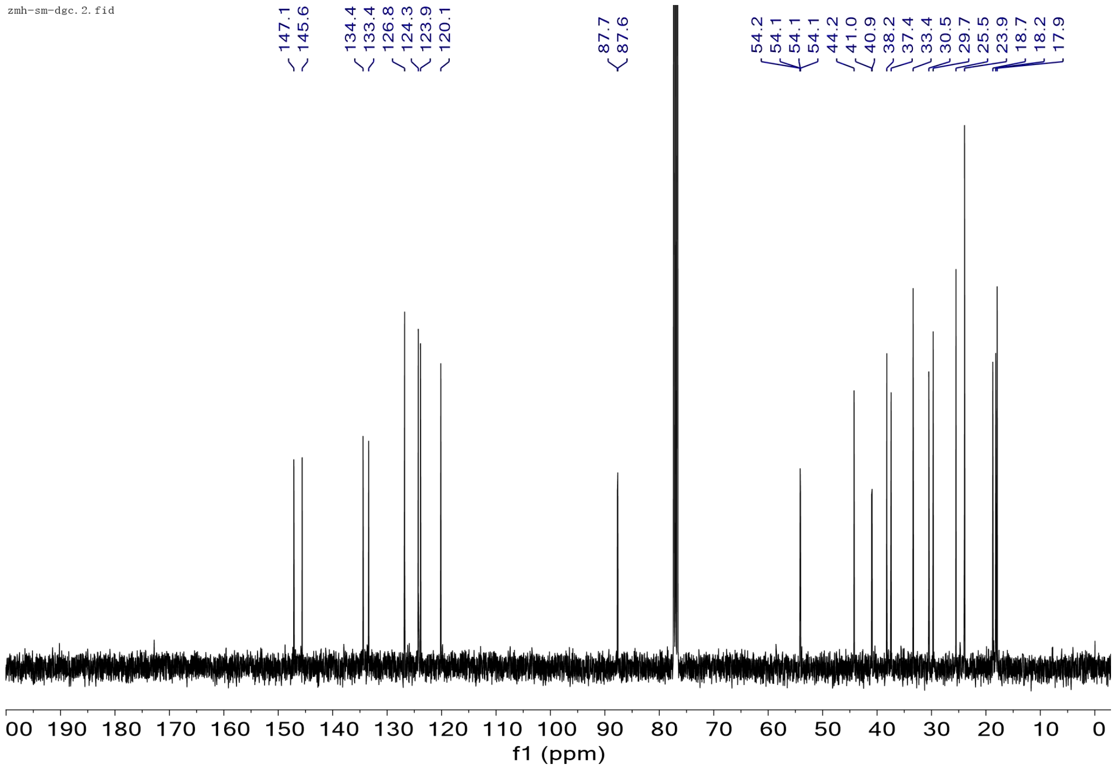
zmh-sm-dgc.2.fid
147.1
145.6
134.4
133.4
126.8
124.3
123.9
120.1
87.7
87.6
54.2
54.1
54.1
54.1
44.2
41.0
40.9
38.2
37.4
33.4
30.5
29.7
25.5
23.9
18.7
18.2
17.9
f1 (ppm)

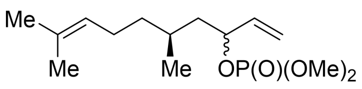

4k

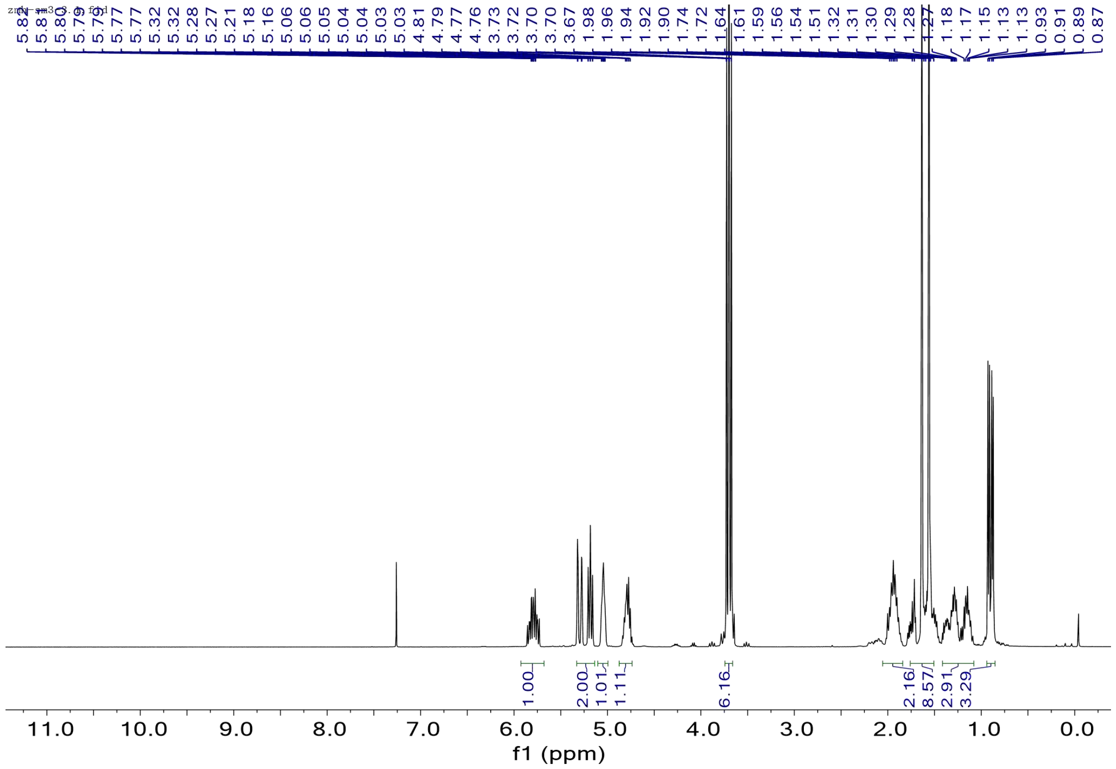

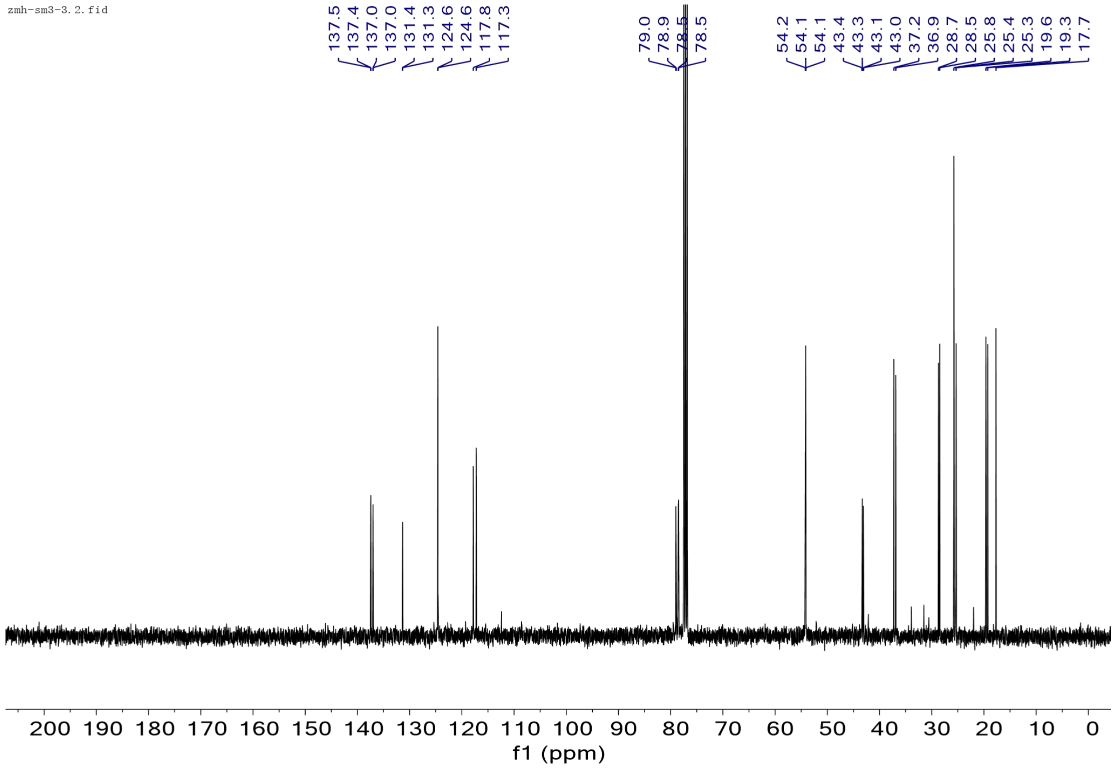

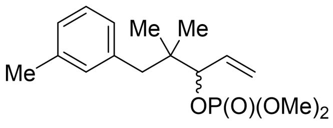

Me
Me
Me
OP(O)(OMe)2

4l

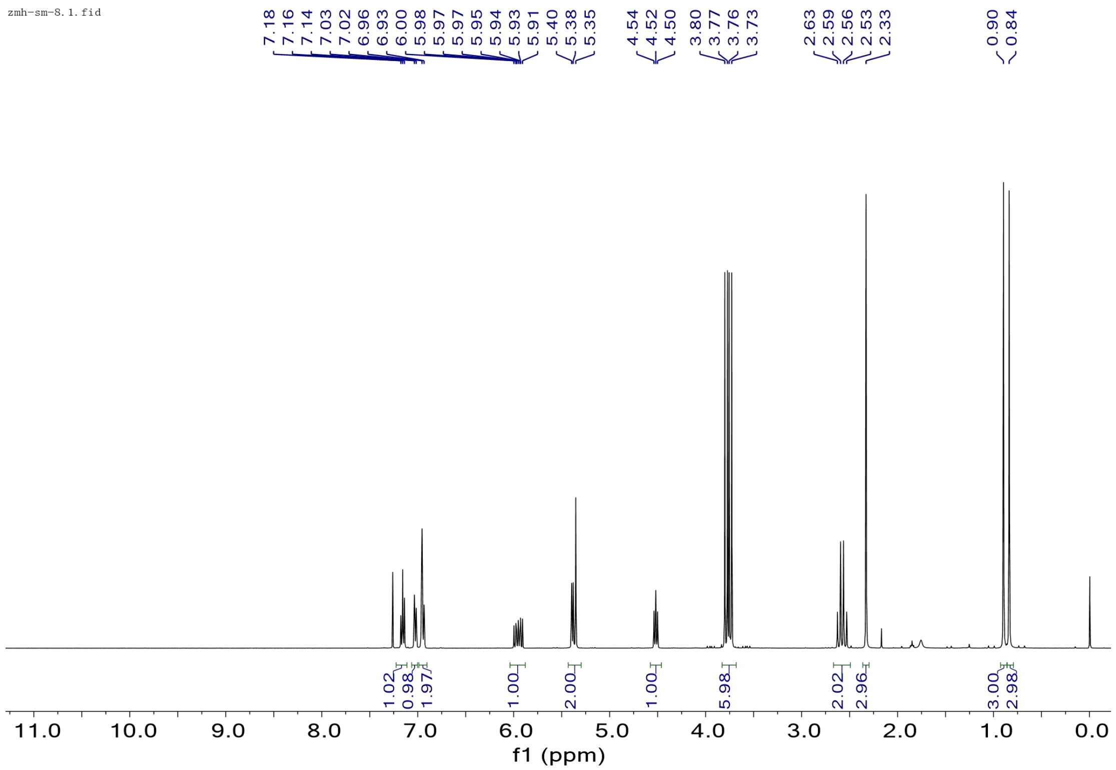

zmh-sm-8.1.fid
7.18 7.16 7.14 7.03 7.02 6.96 6.93 6.00 5.98 5.97 5.97 5.95 5.94 5.93 5.91 5.40 5.38 5.35 4.54 4.52 4.50 3.80 3.77 3.76 3.73 2.63 2.59 2.56 2.53 2.33 0.90 0.84
1.02 0.98 1.97 1.00 2.00 1.00 5.98 2.02 2.96 3.00 2.98
11.0 10.0 9.0 8.0 7.0 6.0 5.0 4.0 3.0 2.0 1.0 0.0
f1 (ppm)

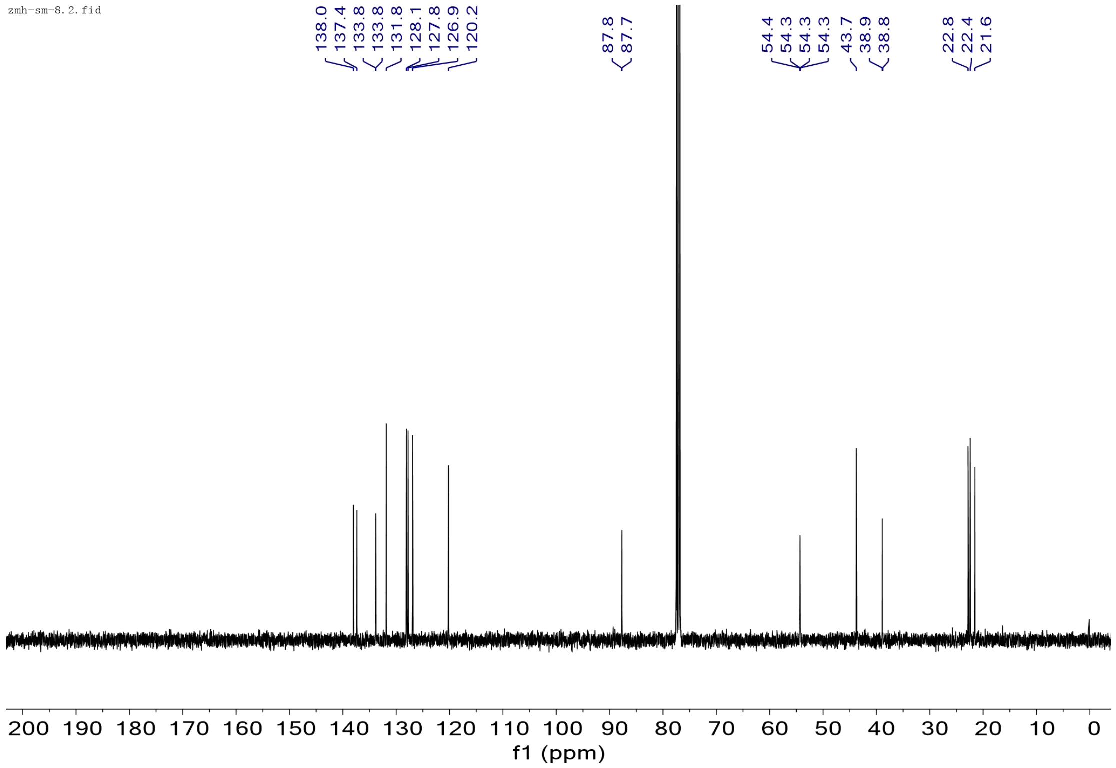

zmh-sm-8.2.fid
138.0 137.4 133.8 133.8 131.8 128.1 127.8 126.9 120.2 87.8 87.7 54.4 54.3 54.3 54.3 43.7 38.9 38.8 22.8 22.4 21.6
200 190 180 170 160 150 140 130 120 110 100 90 80 70 60 50 40 30 20 10 0
f1 (ppm)

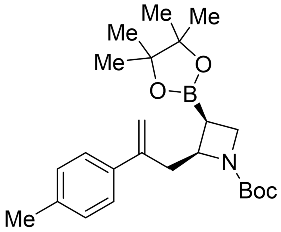
Me
Me
Me
Me
O
O
B
N
Boc
Me

3a

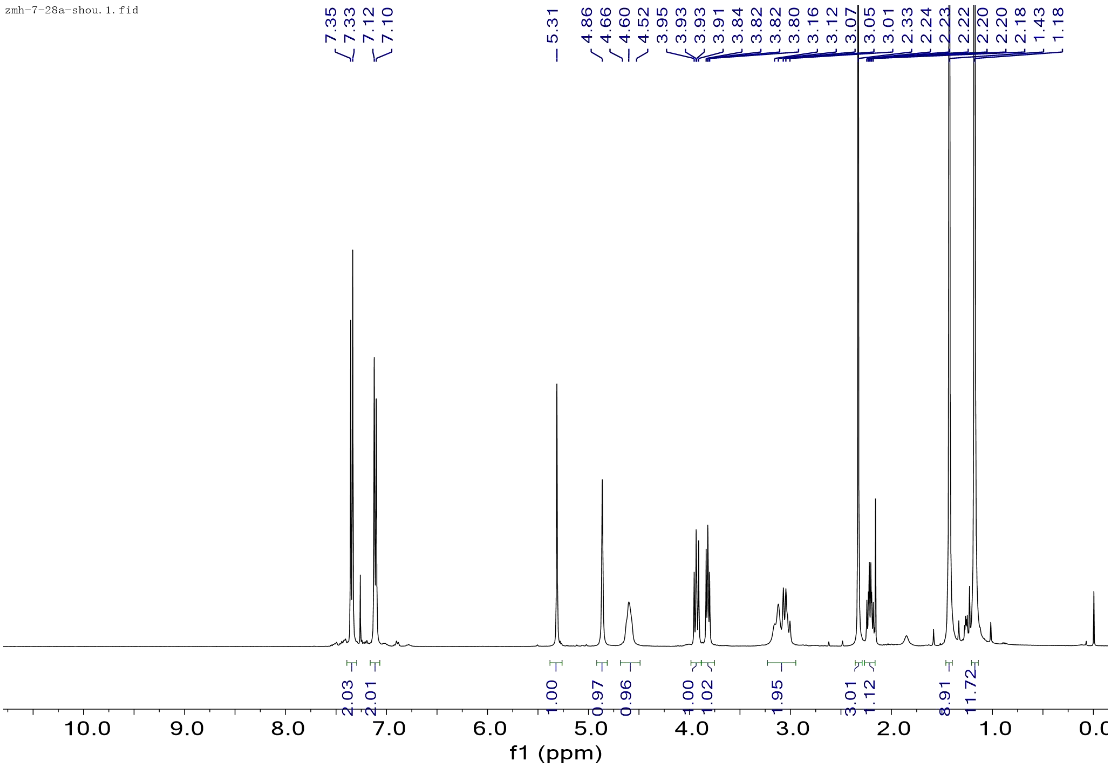
zmh-7-28a-shou.1.fid
7.35
7.33
7.12
7.10
5.31
4.86
4.66
4.60
4.52
3.95
3.93
3.93
3.91
3.84
3.82
3.82
3.80
3.16
3.12
3.07
3.05
3.01
2.33
2.24
2.23
2.22
2.20
2.20
2.18
1.43
1.18
2.03
2.01
1.00
0.97
0.96
1.00
1.02
1.95
3.01
1.12
8.91
11.72
10.0
9.0
8.0
7.0
6.0
5.0
4.0
3.0
2.0
1.0
f1 (ppm)

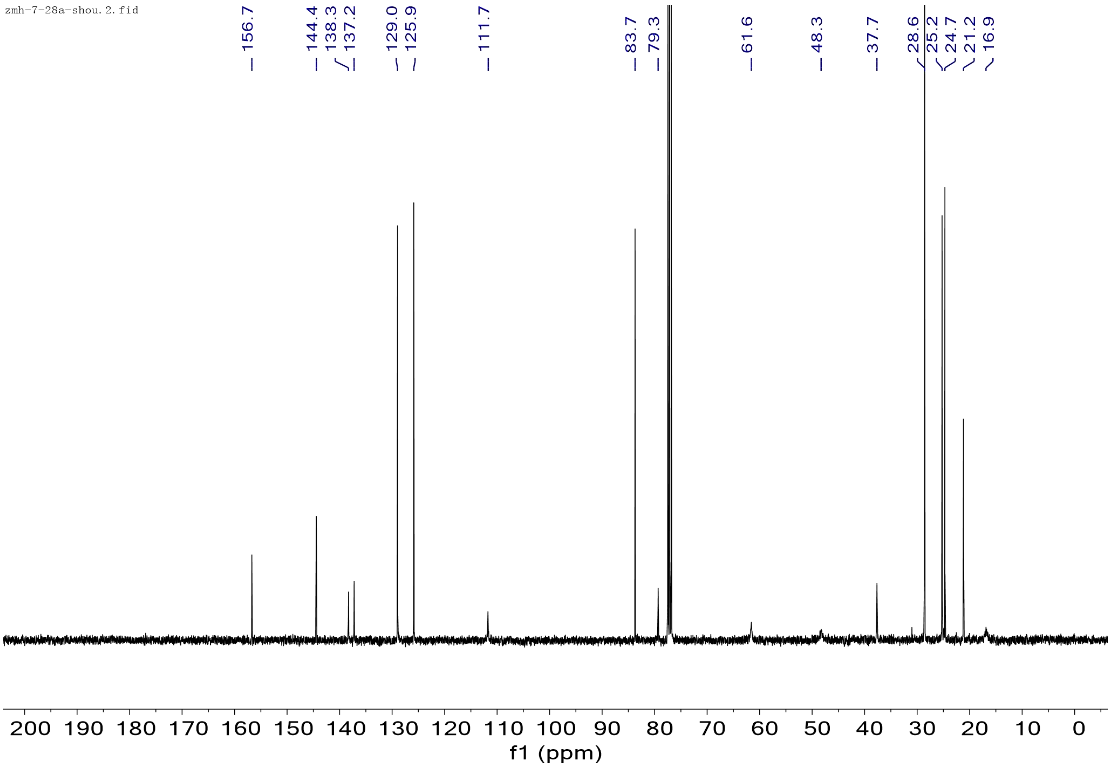
zmh-7-28a-shou.2.fid
156.7
144.4
138.3
137.2
129.0
125.9
111.7
83.7
79.3
61.6
48.3
37.7
28.6
25.2
24.7
21.2
16.9
200
190
180
170
160
150
140
130
120
110
100
90
80
70
60
50
40
30
20
10
0
f1 (ppm)

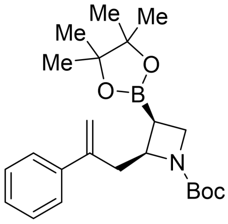

Me
Me
Me
Me
O
O
B
N
Boc

3b

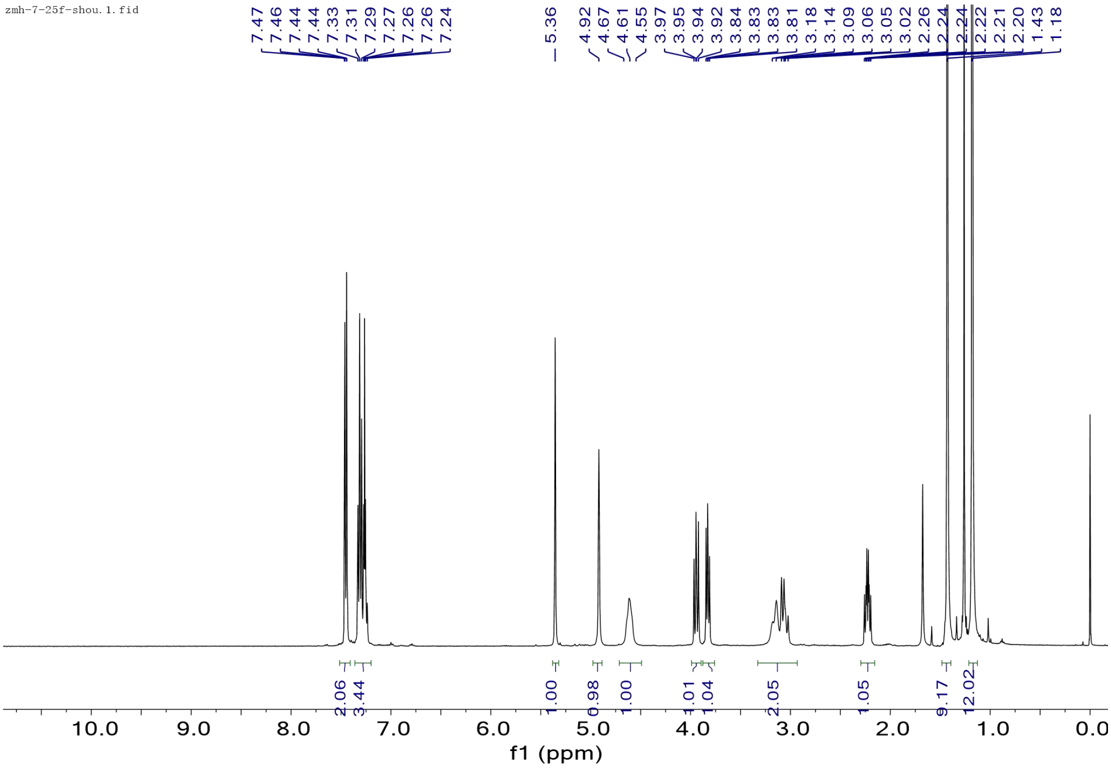

zmh-7-25f-shou.1.fid
7.47
7.46
7.44
7.44
7.33
7.31
7.29
7.27
7.26
7.26
7.24
5.36
4.92
4.67
4.61
4.55
3.97
3.95
3.94
3.92
3.84
3.83
3.83
3.81
3.18
3.14
3.09
3.06
3.05
3.02
2.26
2.24
2.24
2.22
2.21
2.20
1.43
1.18
2.06
3.44
1.00
0.98
1.00
1.01
1.04
2.05
1.05
9.17
12.02
10.0
9.0
8.0
7.0
6.0
5.0
4.0
3.0
2.0
1.0
0.0
f1 (ppm)

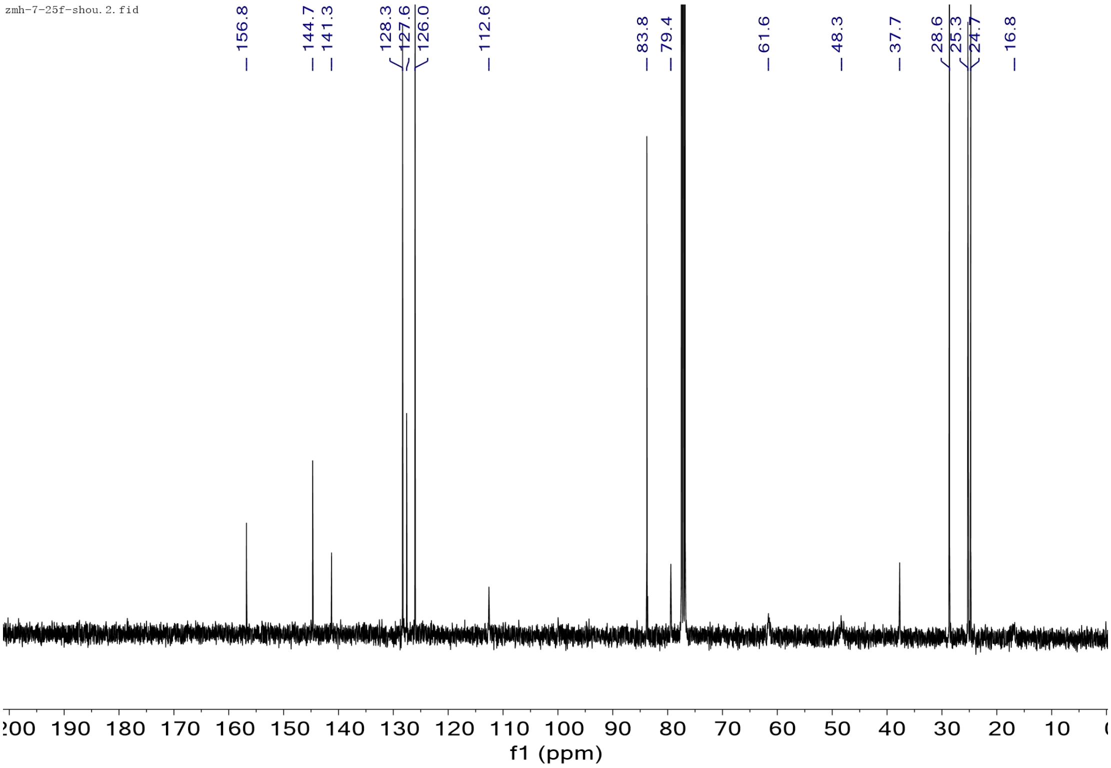

zmh-7-25f-shou.2.fid
156.8
144.7
141.3
128.3
127.6
126.0
112.6
83.8
79.4
61.6
48.3
37.7
28.6
25.3
24.7
16.8
190
180
170
160
150
140
130
120
110
100
90
80
70
60
50
40
30
20
10
f1 (ppm)

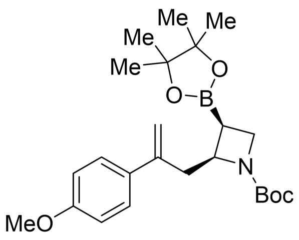
Me
Me
Me
Me
O
O
B
N
Boc
MeO

3c

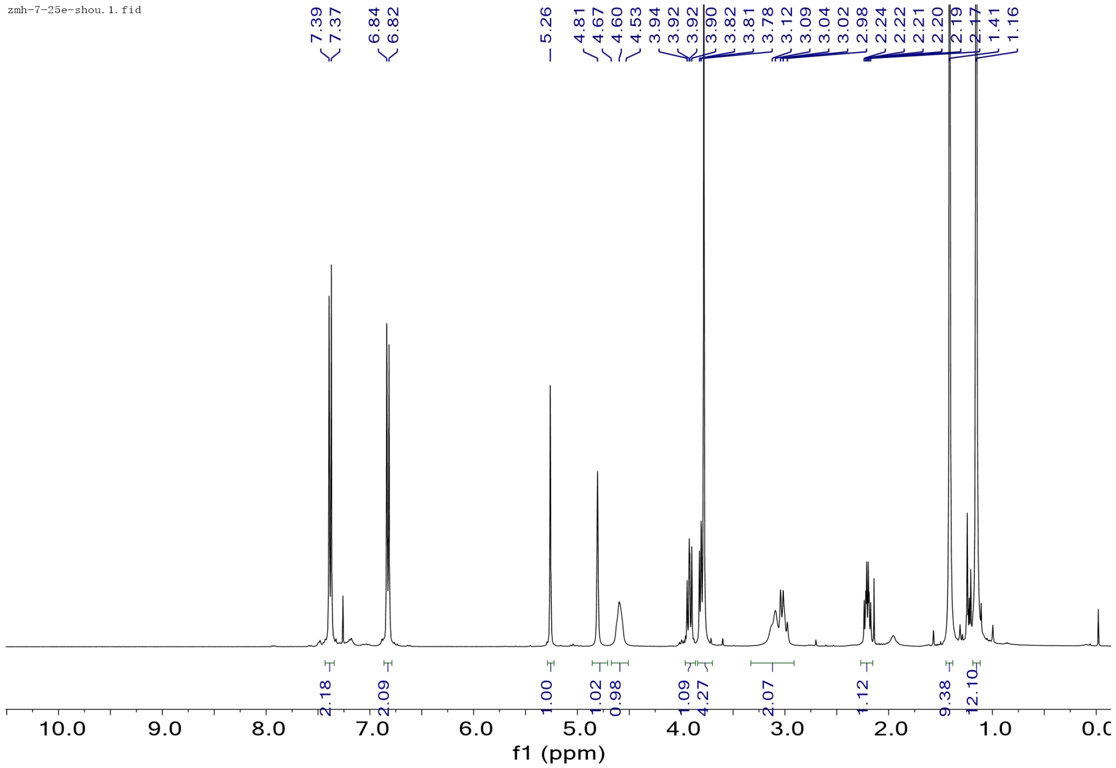
zmh-7-25e-shou.1.fid
7.39
7.37
6.84
6.82
5.26
4.81
4.67
4.60
4.53
3.94
3.92
3.92
3.90
3.82
3.81
3.78
3.12
3.09
3.04
3.02
2.98
2.24
2.22
2.21
2.20
2.19
2.17
1.41
1.16
2.18
2.09
1.00
1.02
0.98
1.09
4.27
2.07
1.12
9.38
12.10
f1 (ppm)

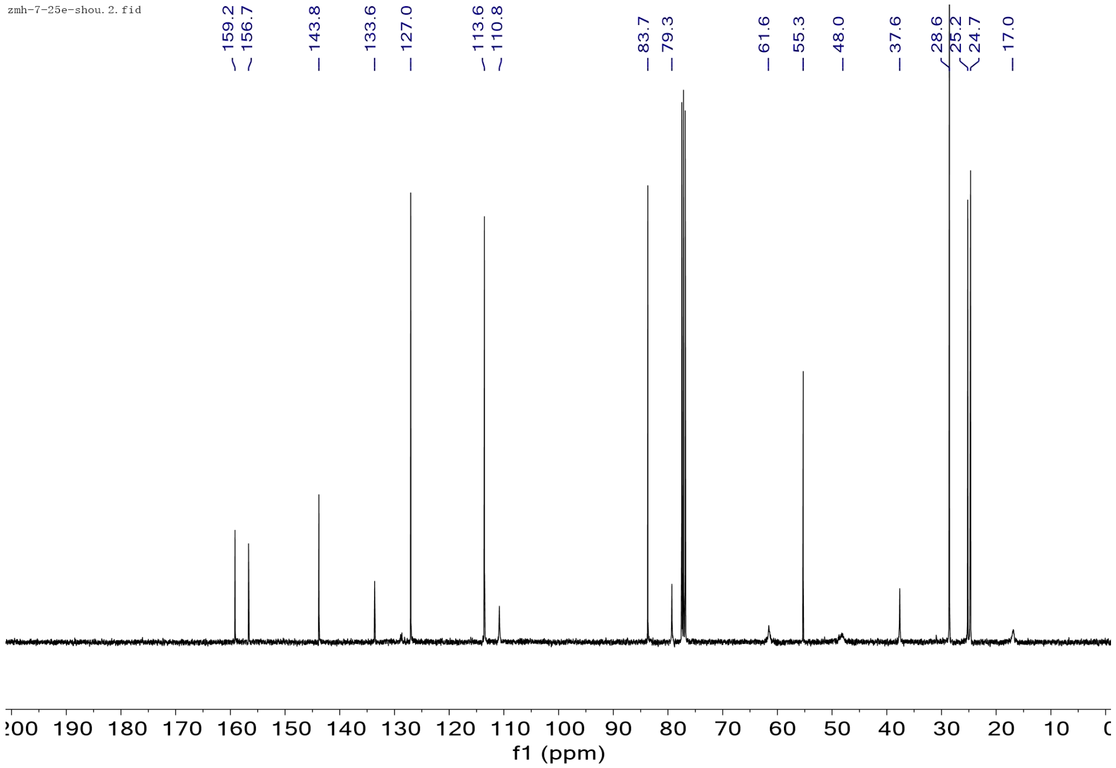
zmh-7-25e-shou.2.fid
159.2
156.7
143.8
133.6
127.0
113.6
110.8
83.7
79.3
61.6
55.3
48.0
37.6
28.6
25.2
24.7
17.0
f1 (ppm)

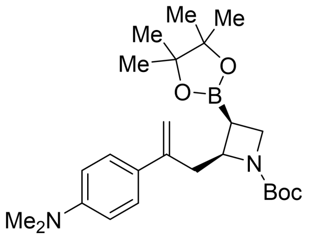
Me
Me
Me
Me
O
O
B
N
Boc
Me2N

3d

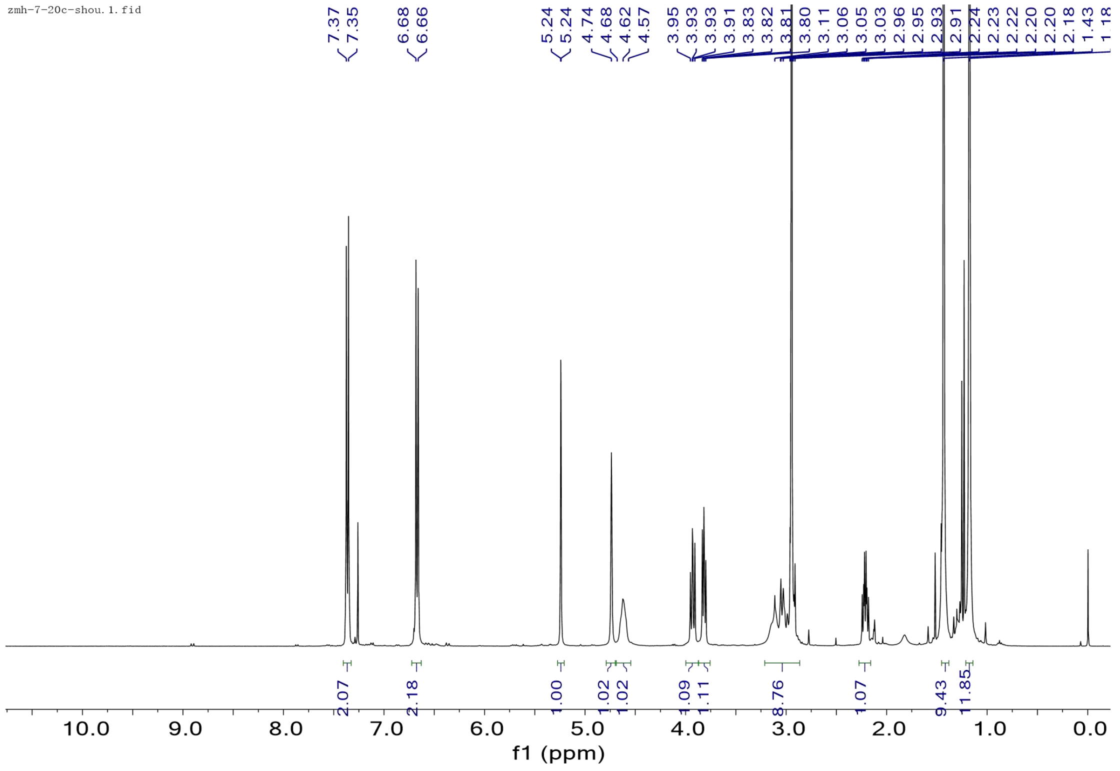
zmh-7-20c-shou.1.fid
7.37
7.35
6.68
6.66
5.24
5.24
4.74
4.68
4.62
4.57
3.95
3.93
3.93
3.91
3.83
3.82
3.81
3.80
3.11
3.06
3.05
3.03
2.96
2.95
2.93
2.91
2.24
2.23
2.22
2.20
2.20
2.18
1.43
2.07
2.18
1.00
1.02
1.02
1.09
1.11
8.76
1.07
9.43
11.85
10.0
9.0
8.0
7.0
6.0
5.0
4.0
3.0
2.0
1.0
0.0
f1 (ppm)

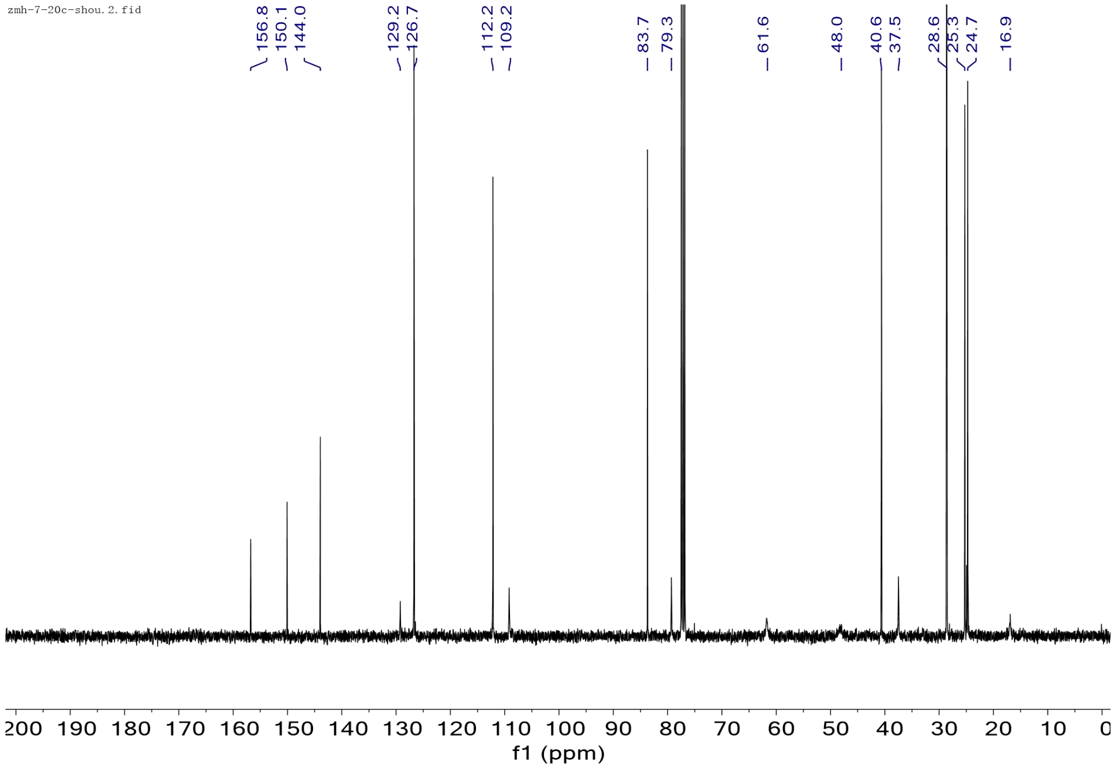
zmh-7-20c-shou.2.fid
156.8
150.1
144.0
129.2
126.7
112.2
109.2
83.7
79.3
61.6
48.0
40.6
37.5
28.6
25.3
24.7
16.9
200
190
180
170
160
150
140
130
120
110
100
90
80
70
60
50
40
30
20
10
f1 (ppm)

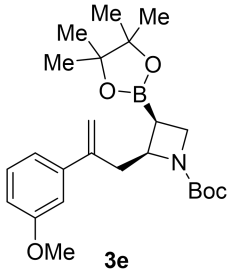
Me
Me
Me
Me
O
O
B
N
Boc
OMe

3e

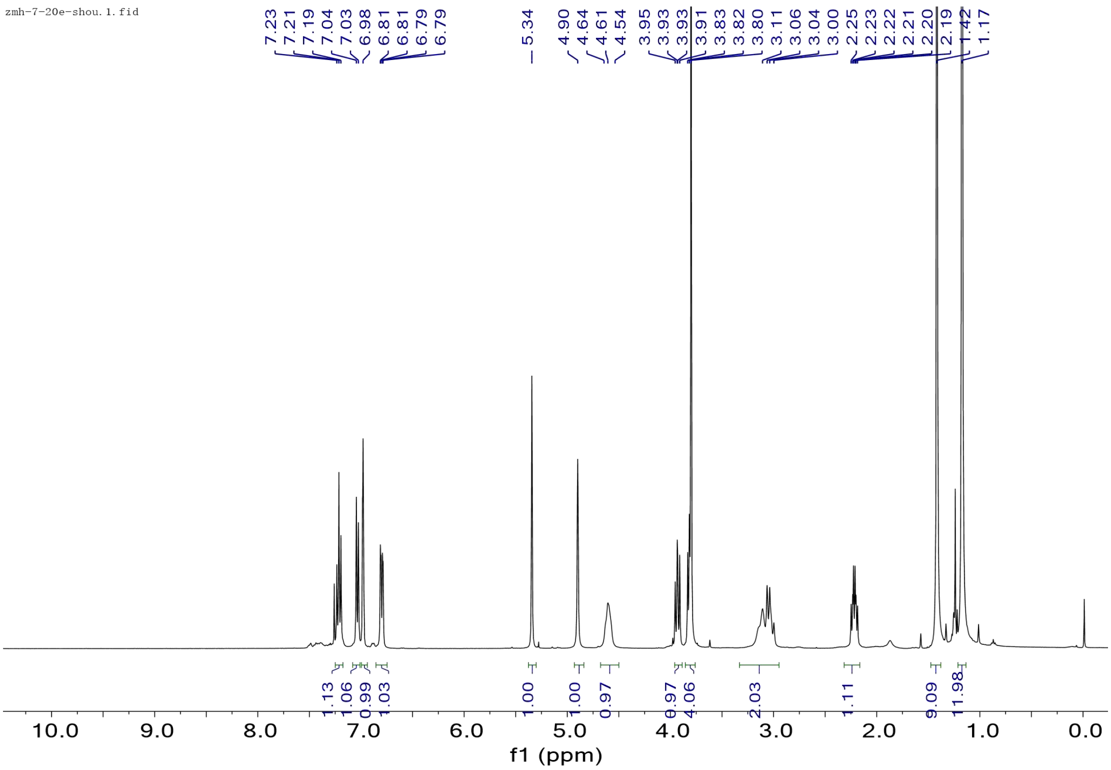
zmh-7-20e-shou.1.fid
7.23 7.21 7.19 7.04 7.03 6.98 6.81 6.81 6.79 6.79
5.34
4.90 4.64 4.61 4.54
3.95 3.93 3.93 3.91 3.83 3.82 3.80
3.11 3.06 3.04 3.00
2.25 2.23 2.22 2.21 2.20 2.19
1.42 1.17
1.13 1.06 0.99 1.03
1.00 1.00 0.97
0.97 4.06
2.03
1.11
9.09 11.98
10.0 9.0 8.0 7.0 6.0 5.0 4.0 3.0 2.0 1.0 0.0
f1 (ppm)

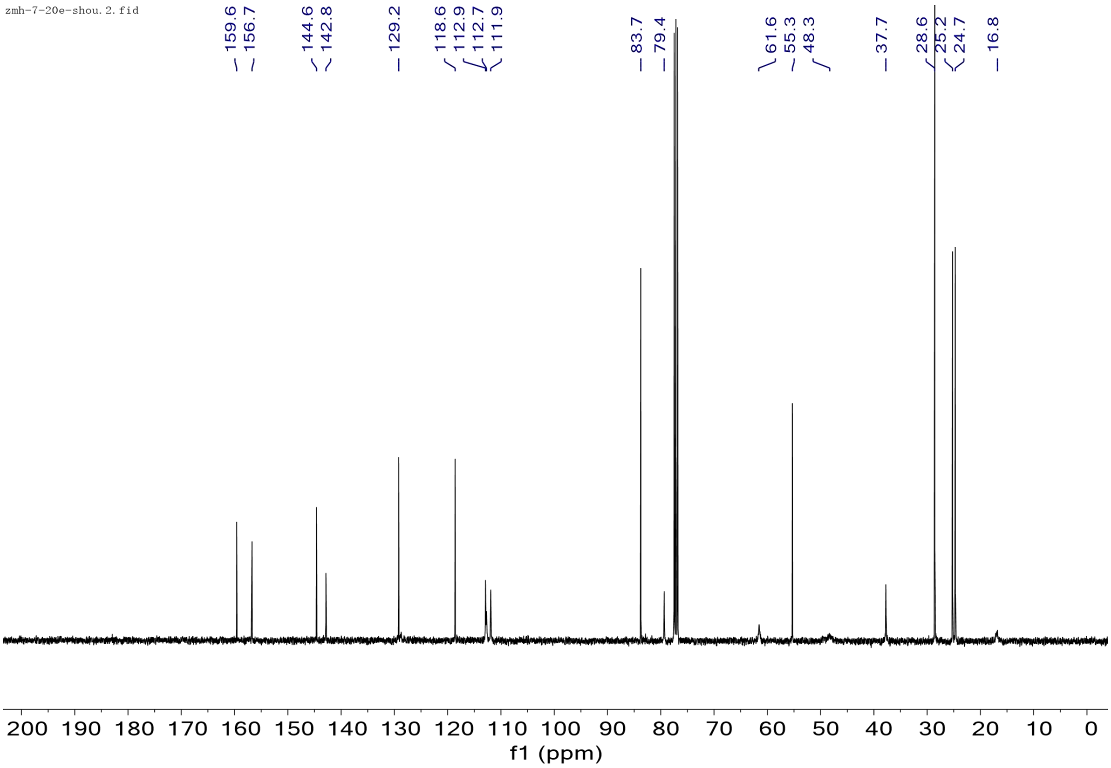
zmh-7-20e-shou.2.fid
159.6 156.7
144.6 142.8
129.2
118.6 112.9 112.7 111.9
83.7 79.4
61.6 55.3 48.3
37.7
28.6 25.2 24.7
16.8
200 190 180 170 160 150 140 130 120 110 100 90 80 70 60 50 40 30 20 10 0
f1 (ppm)

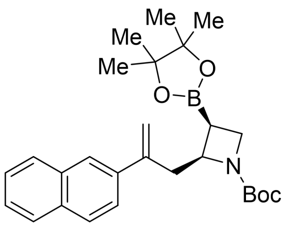
Me
Me
Me
Me
O
B
O
N
Boc

3f

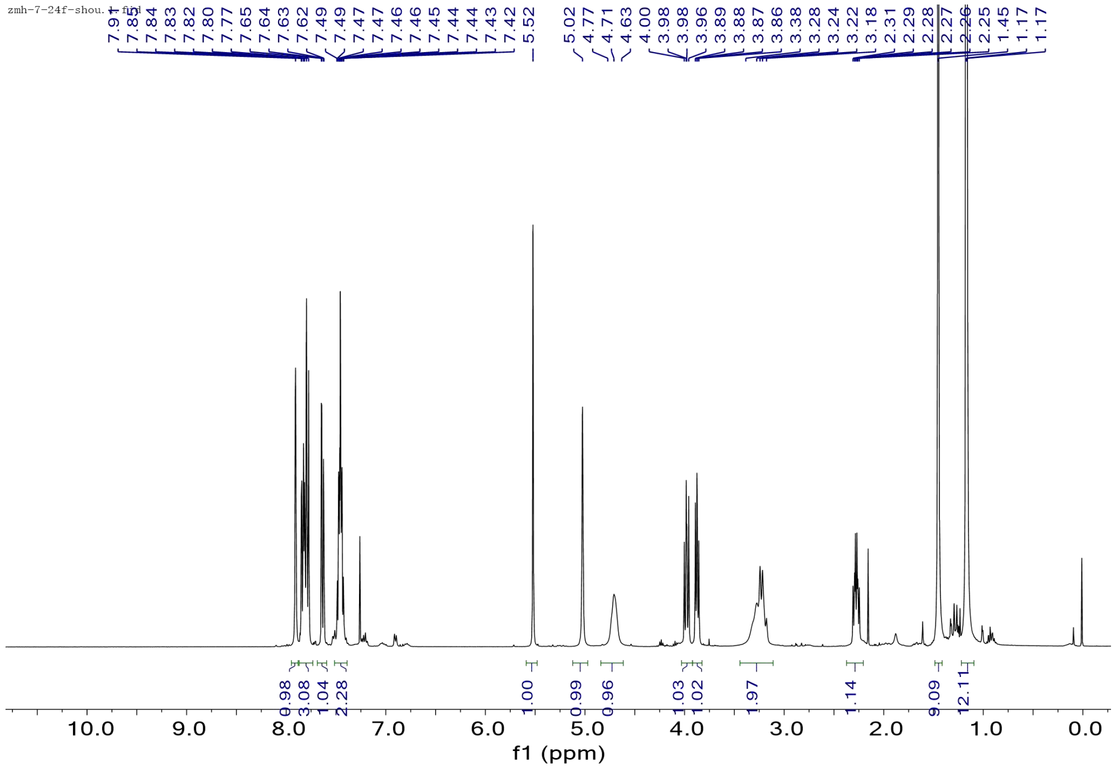
zmh-7-24f-shou.1.fid
7.91 7.85 7.84 7.83 7.82 7.80 7.77 7.65 7.64 7.63 7.62 7.49 7.49 7.47 7.47 7.46 7.46 7.45 7.44 7.44 7.43 7.42 5.52 5.02 4.77 4.71 4.63 4.00 3.98 3.98 3.96 3.89 3.88 3.87 3.86 3.38 3.28 3.24 3.22 3.18 2.31 2.29 2.28 2.27 2.26 2.25 1.45 1.17 1.17
0.98 3.08 1.04 2.28 1.00 0.99 0.96 1.03 1.02 1.97 1.14 9.09 12.11
10.0 9.0 8.0 7.0 6.0 5.0 4.0 3.0 2.0 1.0 0.0
f1 (ppm)

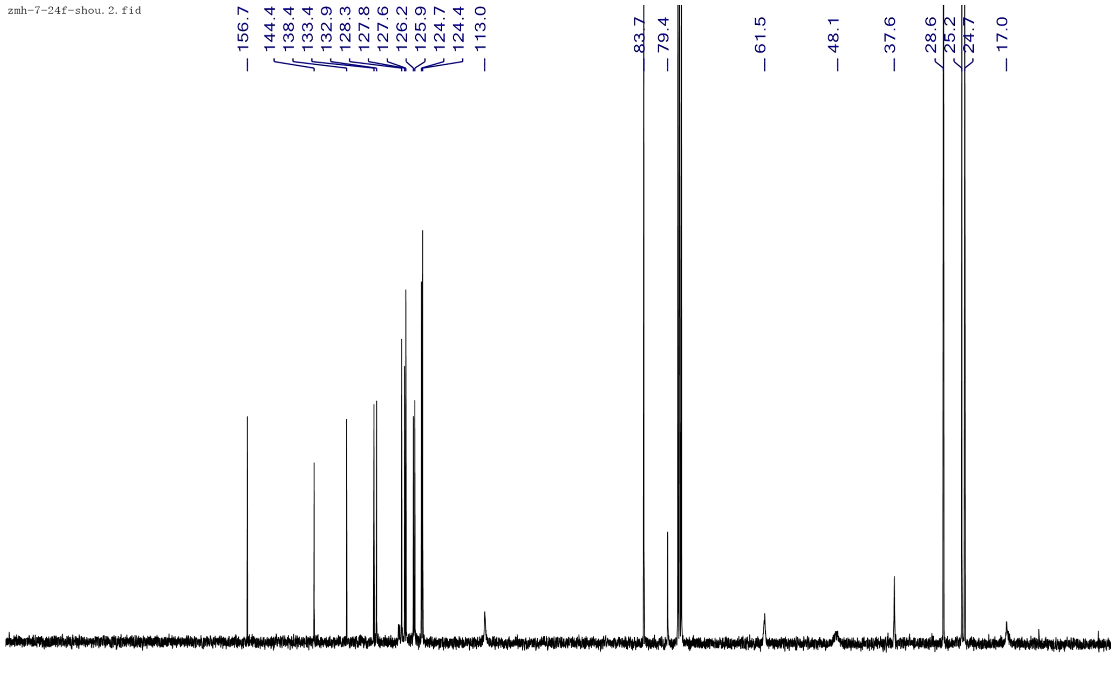
zmh-7-24f-shou.2.fid
156.7 144.4 138.4 133.4 132.9 128.3 127.8 127.6 126.2 125.9 124.7 124.4 113.0 83.7 79.4 61.5 48.1 37.6 28.6 25.2 24.7 17.0
200 190 180 170 160 150 140 130 120 110 100 90 80 70 60 50 40 30 20 10 0
f1 (ppm)

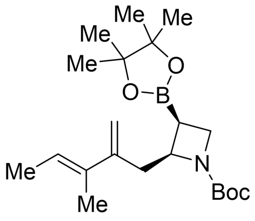

Me
Me
Me
Me
O
O
B
N
Boc
Me
Me

3g

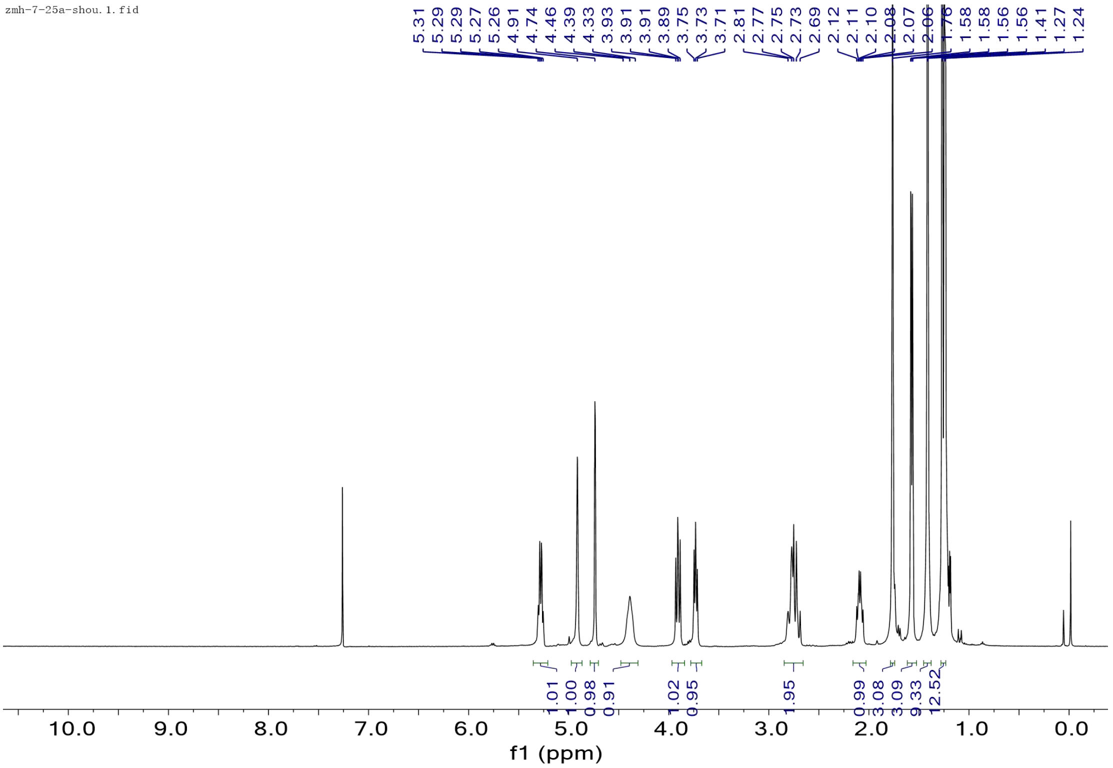

zmh-7-25a-shou. 1. fid
5.31 5.29 5.29 5.27 5.26 4.91 4.74 4.46 4.39 4.33 3.93 3.91 3.91 3.89 3.75 3.73 3.71 2.81 2.77 2.75 2.73 2.69 2.12 2.11 2.10 2.08 2.07 2.06 1.76 1.58 1.58 1.56 1.56 1.41 1.27 1.24
1.01 1.00 0.98 0.91 1.02 0.95 1.95 0.99 3.08 3.09 9.33 12.52
10.0 9.0 8.0 7.0 6.0 5.0 4.0 3.0 2.0 1.0 0.0
f1 (ppm)

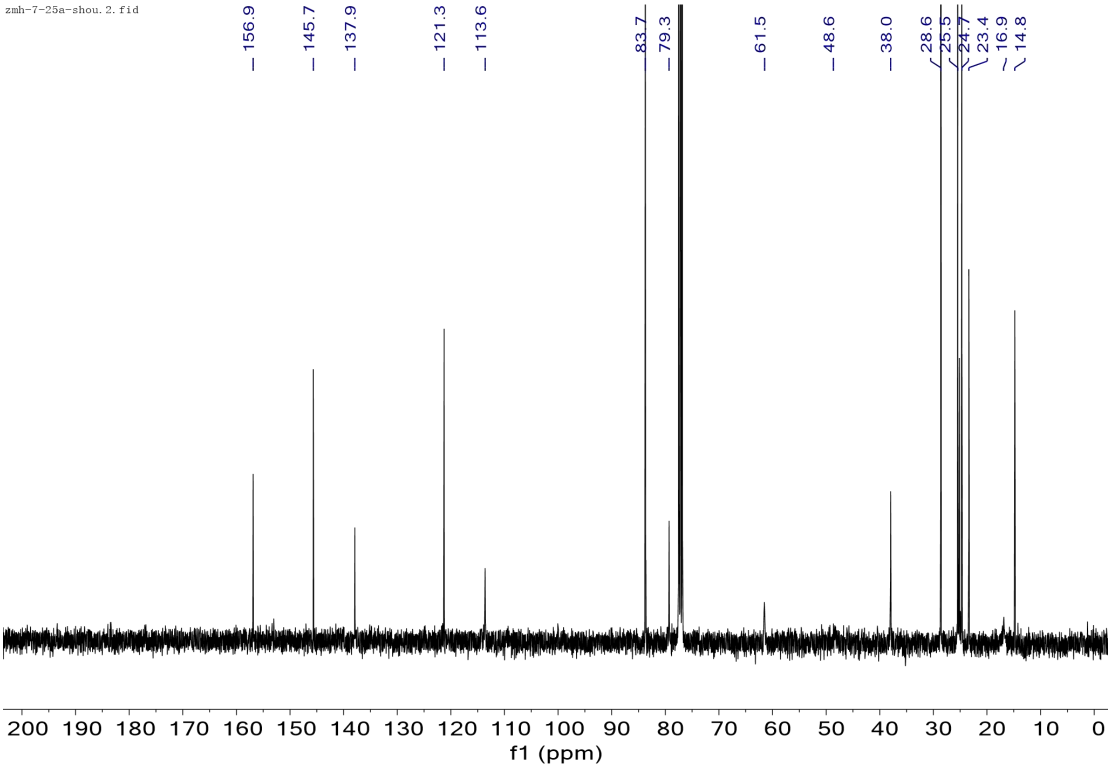

zmh-7-25a-shou. 2. fid
156.9 145.7 137.9 121.3 113.6 83.7 79.3 61.5 48.6 38.0 28.6 25.5 24.7 23.4 16.9 14.8
200 190 180 170 160 150 140 130 120 110 100 90 80 70 60 50 40 30 20 10 0
f1 (ppm)

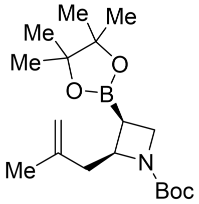
Me
Me
Me
Me
O
O
B
N
Me
Boc

3h

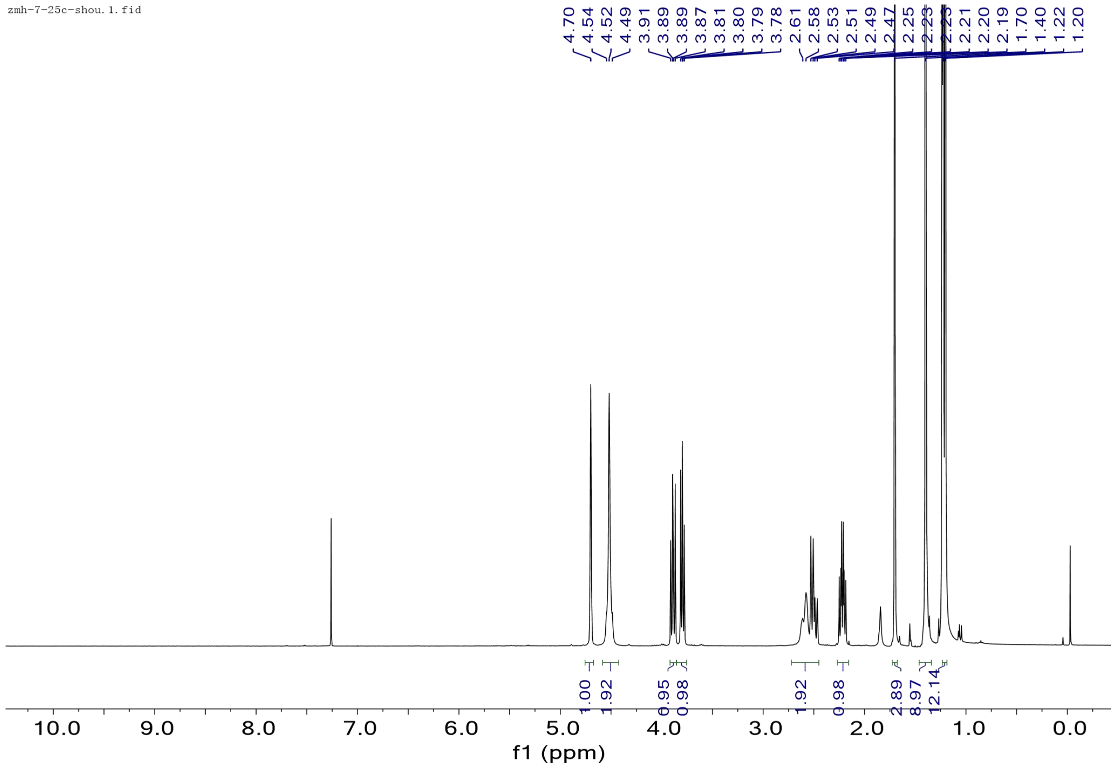
zmh-7-25c-shou.1.fid
4.70
4.54
4.52
4.49
3.91
3.89
3.89
3.87
3.81
3.80
3.79
3.78
2.61
2.58
2.53
2.51
2.49
2.47
2.25
2.23
2.23
2.21
2.20
2.19
1.70
1.40
1.22
1.20
1.00
1.92
0.95
0.98
1.92
0.98
2.89
8.97
12.14
10.0 9.0 8.0 7.0 6.0 5.0 4.0 3.0 2.0 1.0 0.0
f1 (ppm)

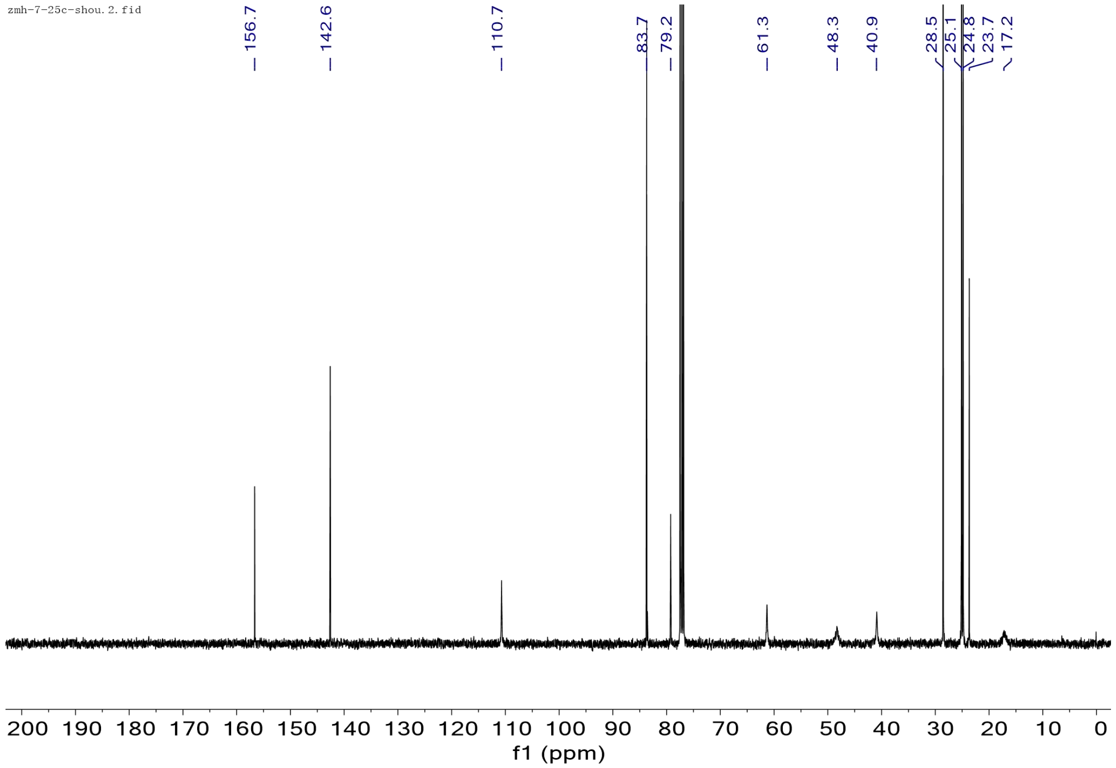
zmh-7-25c-shou.2.fid
156.7
142.6
110.7
83.7
79.2
61.3
48.3
40.9
28.5
25.1
24.8
23.7
17.2
200 190 180 170 160 150 140 130 120 110 100 90 80 70 60 50 40 30 20 10 0
f1 (ppm)

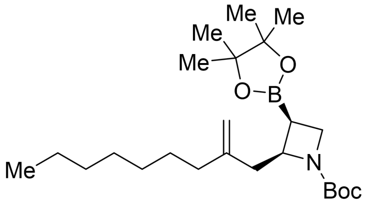
Me
Me
Me
Me
O
O
B
Me
N
Boc

3i

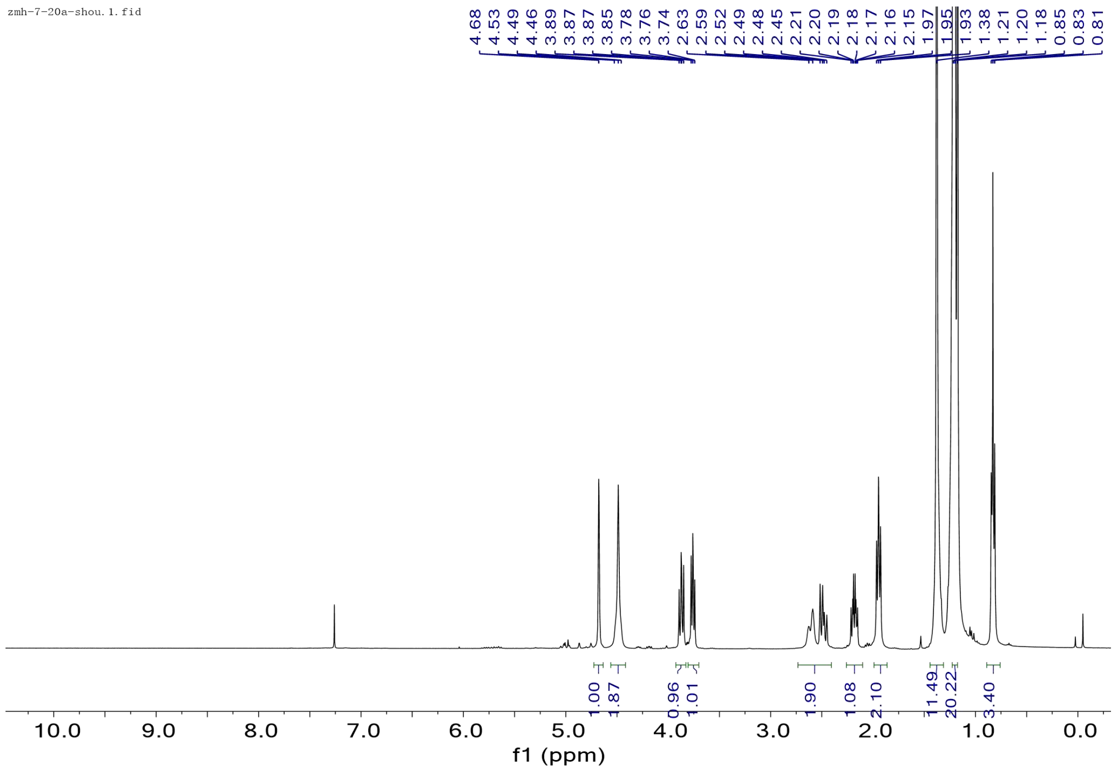
zmh-7-20a-shou.1.fid
4.68 4.53 4.49 4.46 3.89 3.87 3.87 3.85 3.78 3.76 3.74 2.63 2.59 2.52 2.49 2.48 2.45 2.21 2.20 2.19 2.18 2.17 2.16 2.15 1.97 1.95 1.93 1.38 1.21 1.20 1.18 0.85 0.83 0.81
1.00 1.87 0.96 1.01 1.90 1.08 2.10 11.49 20.22 3.40
10.0 9.0 8.0 7.0 6.0 5.0 4.0 3.0 2.0 1.0 0.0
f1 (ppm)

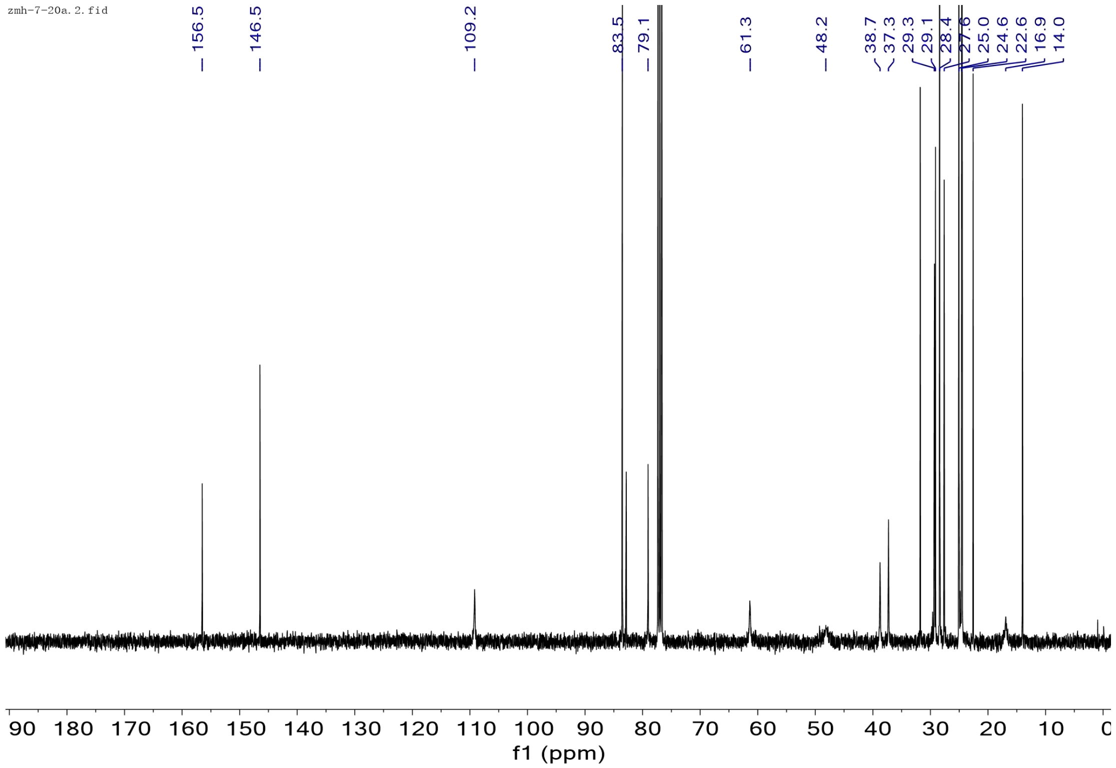
zmh-7-20a.2.fid
156.5 146.5 109.2 83.5 79.1 61.3 48.2 38.7 37.3 29.3 29.1 28.4 27.6 25.0 24.6 22.6 16.9 14.0
90 180 170 160 150 140 130 120 110 100 90 80 70 60 50 40 30 20 10 0
f1 (ppm)

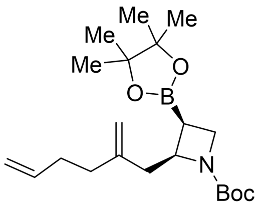
Me
Me
Me
Me
O
O
B
N
Boc

3j

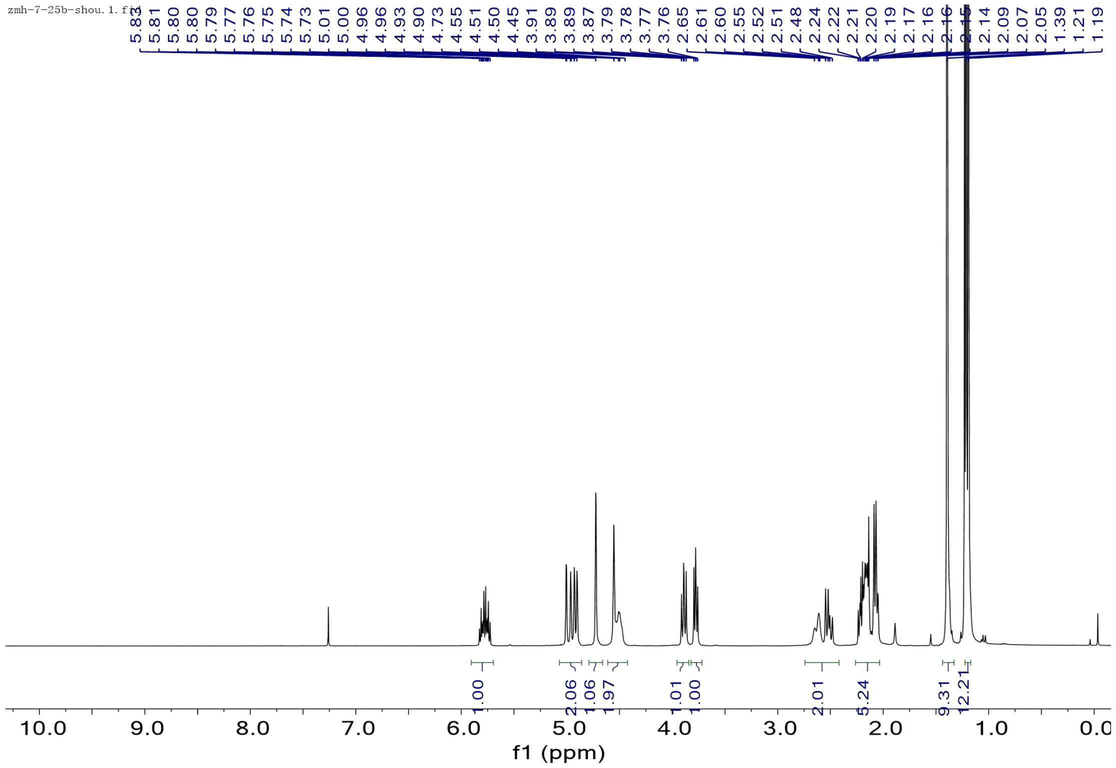
zmh-7-25b-shou.1.fid
5.83 5.81 5.80 5.79 5.77 5.76 5.75 5.74 5.73 5.01 5.00 4.96 4.96 4.93 4.90 4.73 4.55 4.51 4.50 4.45 3.91 3.89 3.87 3.79 3.78 3.77 3.76 2.65 2.61 2.60 2.55 2.52 2.51 2.48 2.24 2.22 2.21 2.20 2.19 2.17 2.16 2.16 2.15 2.14 2.09 2.07 2.05 1.39 1.21 1.19
1.00
2.06
1.06
1.97
1.01
1.00
2.01
5.24
9.31
12.21
10.0 9.0 8.0 7.0 6.0 5.0 4.0 3.0 2.0 1.0 0.0
f1 (ppm)

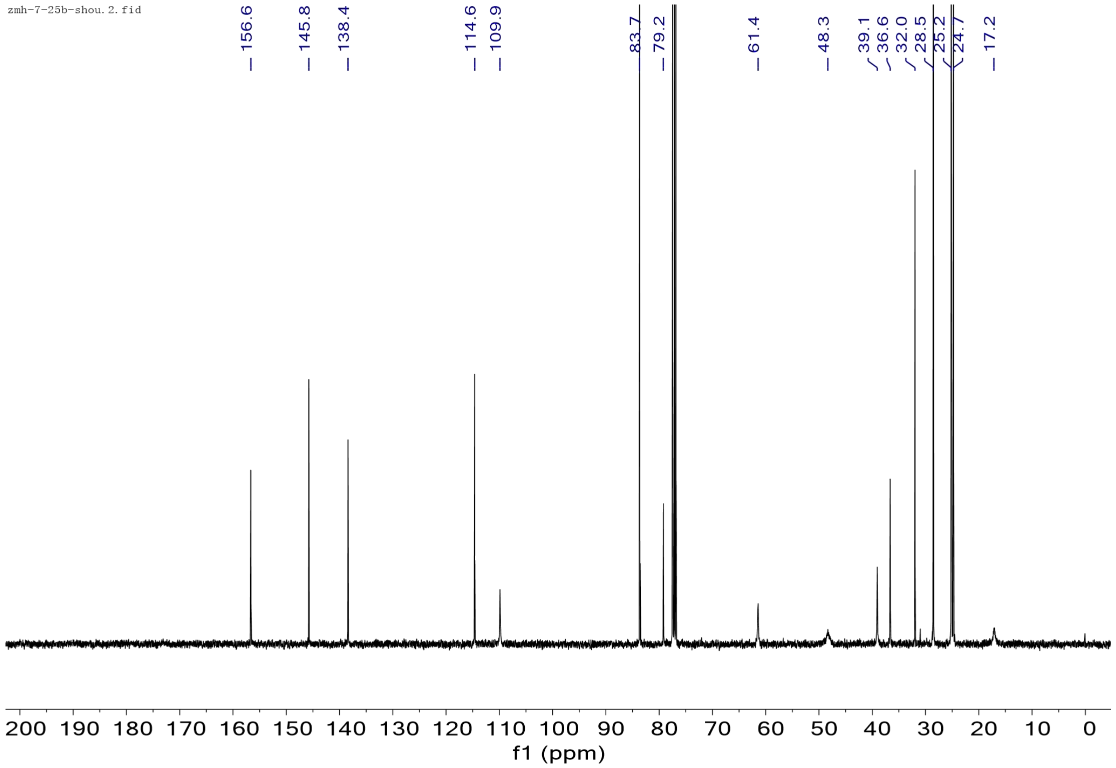
zmh-7-25b-shou.2.fid
156.6
145.8
138.4
114.6
109.9
83.7
79.2
61.4
48.3
39.1
36.6
32.0
28.5
25.2
24.7
17.2
200 190 180 170 160 150 140 130 120 110 100 90 80 70 60 50 40 30 20 10 0
f1 (ppm)

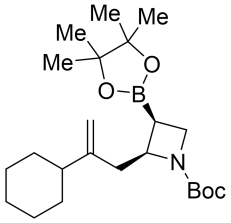
Me
Me
Me
Me
O
O
B
N
Boc

3k

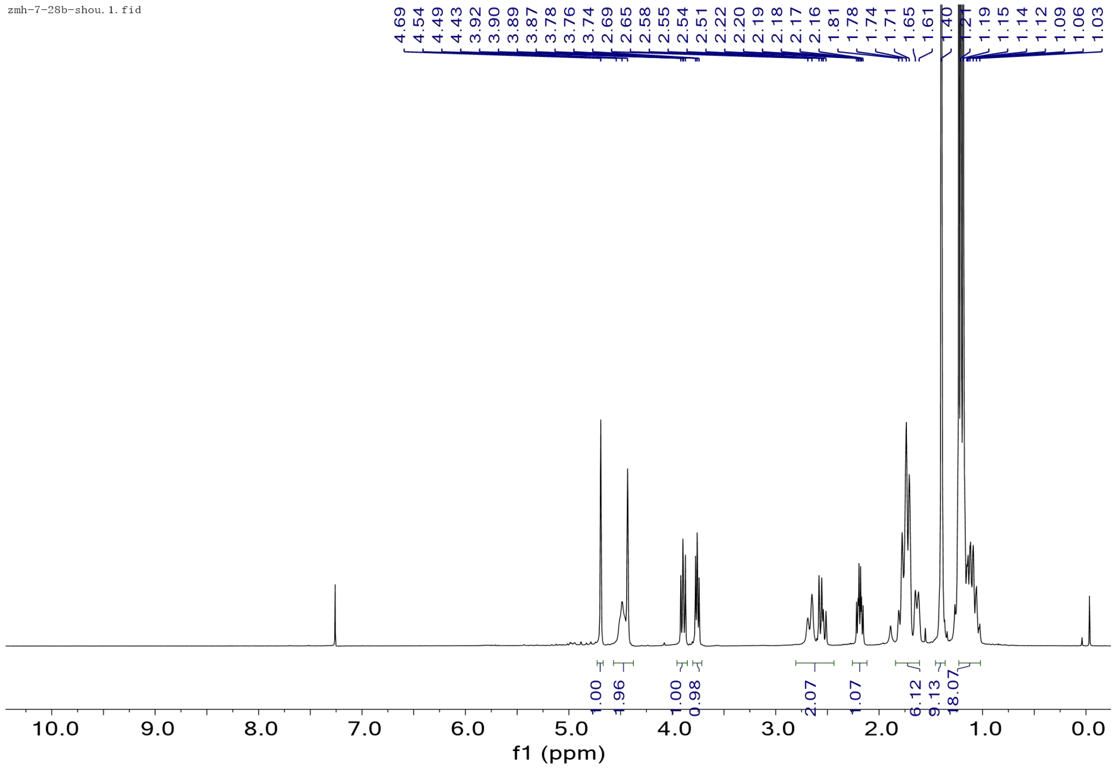
zmh-7-28b-shou.1.fid
4.69
4.54
4.49
4.43
3.92
3.90
3.89
3.87
3.78
3.76
3.74
2.69
2.65
2.58
2.55
2.54
2.51
2.22
2.20
2.19
2.18
2.17
2.16
1.81
1.78
1.74
1.71
1.65
1.61
1.40
1.21
1.19
1.15
1.14
1.12
1.09
1.06
1.03
1.00
1.96
1.00
0.98
2.07
1.07
6.12
9.13
18.07
10.0
9.0
8.0
7.0
6.0
5.0
4.0
3.0
2.0
1.0
0.0
f1 (ppm)

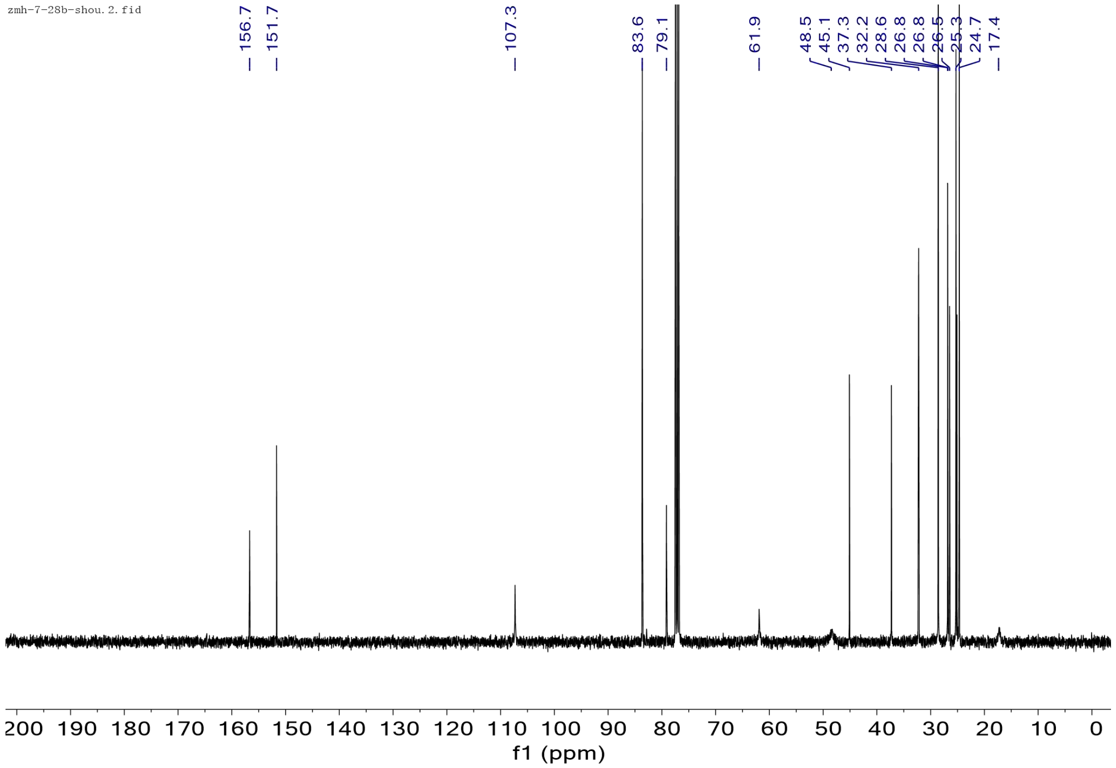
zmh-7-28b-shou.2.fid
156.7
151.7
107.3
83.6
79.1
61.9
48.5
45.1
37.3
32.2
28.6
26.8
26.8
26.5
25.3
24.7
17.4
200
190
180
170
160
150
140
130
120
110
100
90
80
70
60
50
40
30
20
10
0
f1 (ppm)

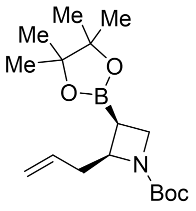
Me
Me
Me
Me
O
O
B
N
Boc

3l

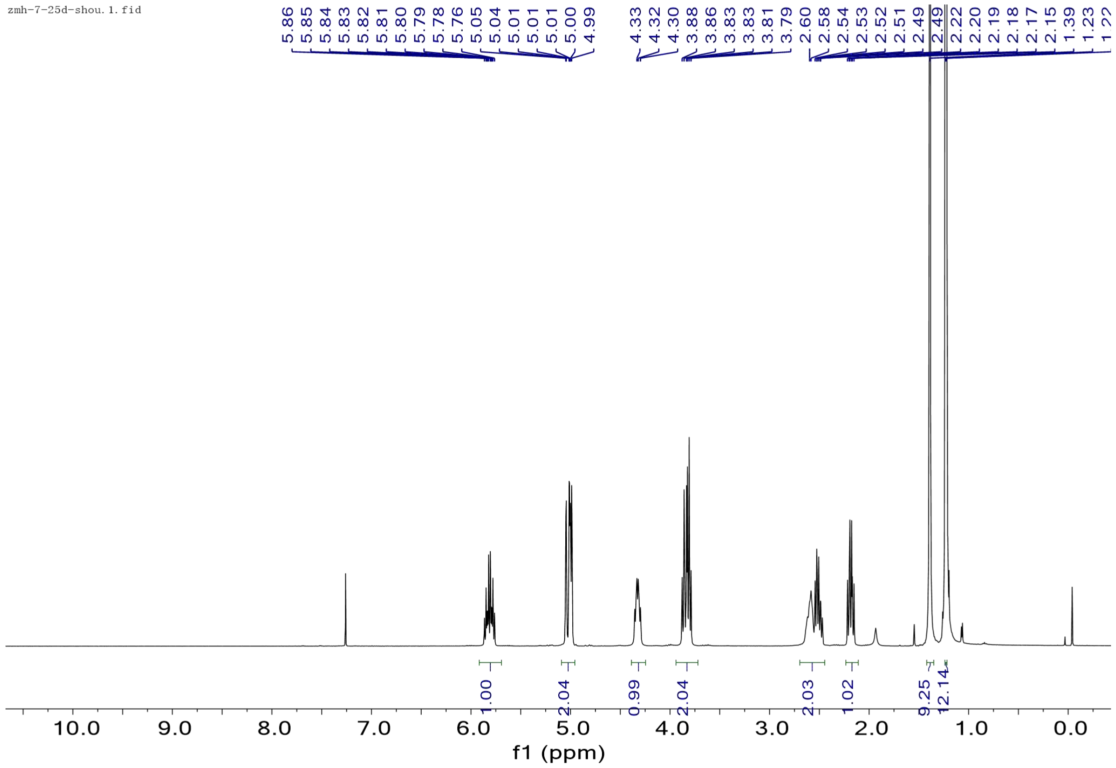
zmh-7-25d-shou.1.fid
5.86 5.85 5.84 5.83 5.82 5.81 5.80 5.79 5.78 5.76 5.05 5.04 5.01 5.01 5.00 4.99 4.33 4.32 4.30 3.88 3.86 3.83 3.83 3.81 3.79 2.60 2.58 2.54 2.53 2.52 2.51 2.49 2.49 2.22 2.20 2.19 2.18 2.17 2.15 1.39 1.23
1.00 2.04 0.99 2.04 2.03 1.02 9.25 12.14
10.0 9.0 8.0 7.0 6.0 5.0 4.0 3.0 2.0 1.0 0.0
f1 (ppm)

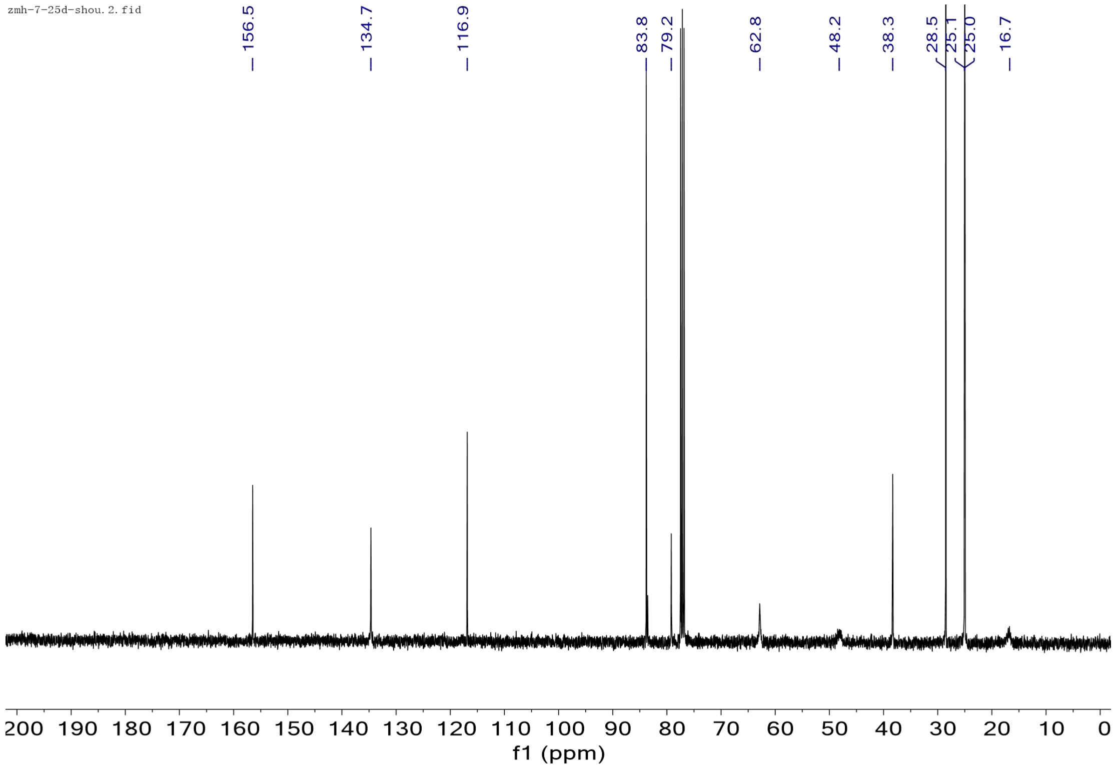
zmh-7-25d-shou.2.fid
156.5 134.7 116.9 83.8 79.2 62.8 48.2 38.3 28.5 25.1 25.0 16.7
200 190 180 170 160 150 140 130 120 110 100 90 80 70 60 50 40 30 20 10 0
f1 (ppm)

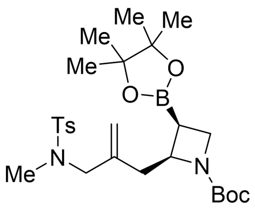

3m

zmh-7-28c-shou.1.fid

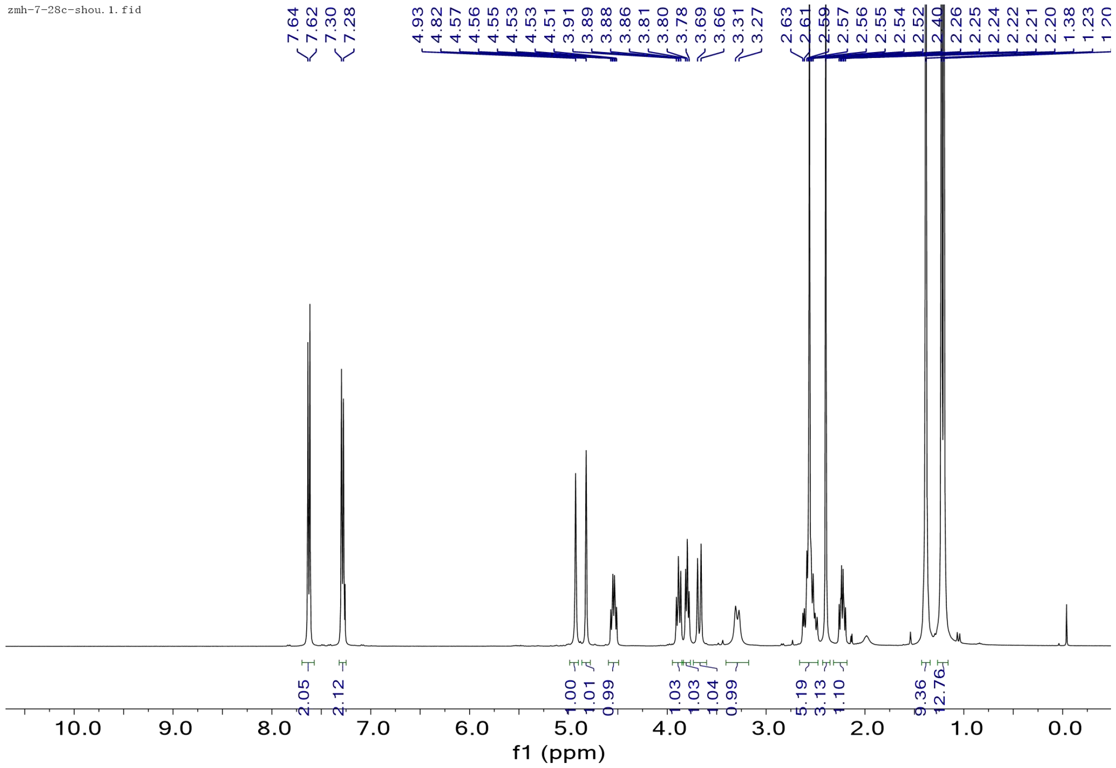

zmh-7-28c-shou.2.fid

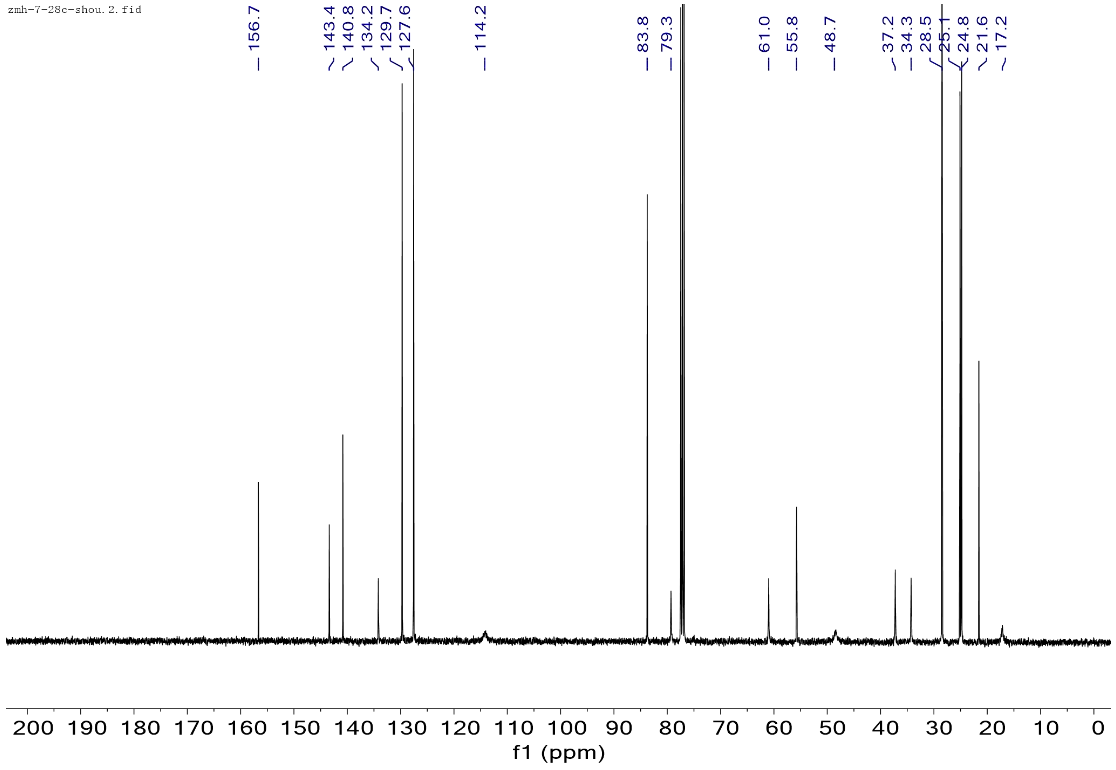

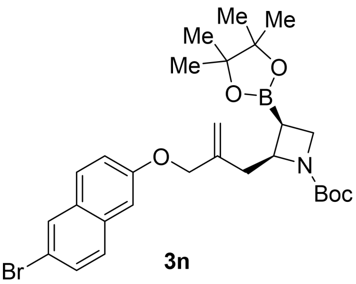

3n

zmh-7-24e-shou.1.fid

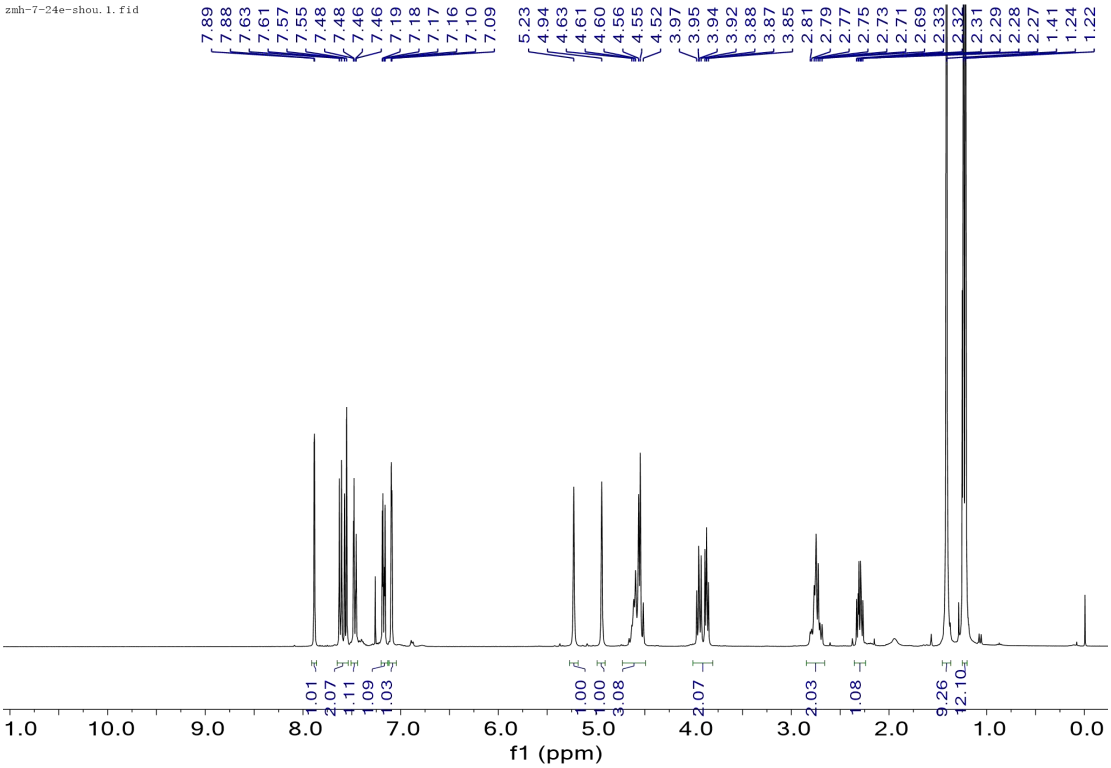

zmh-7-24e-shou.2.fid

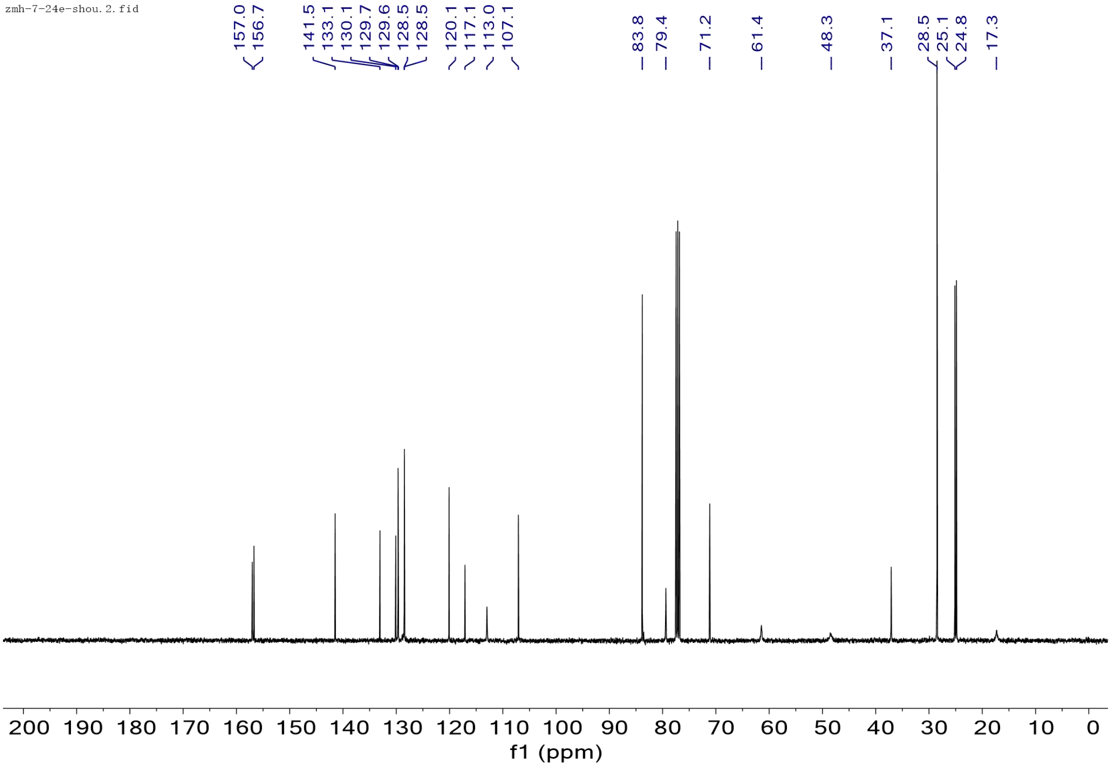

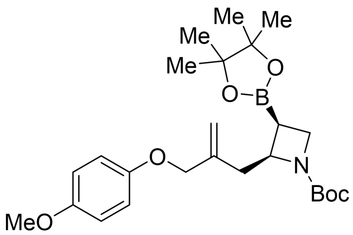

3o

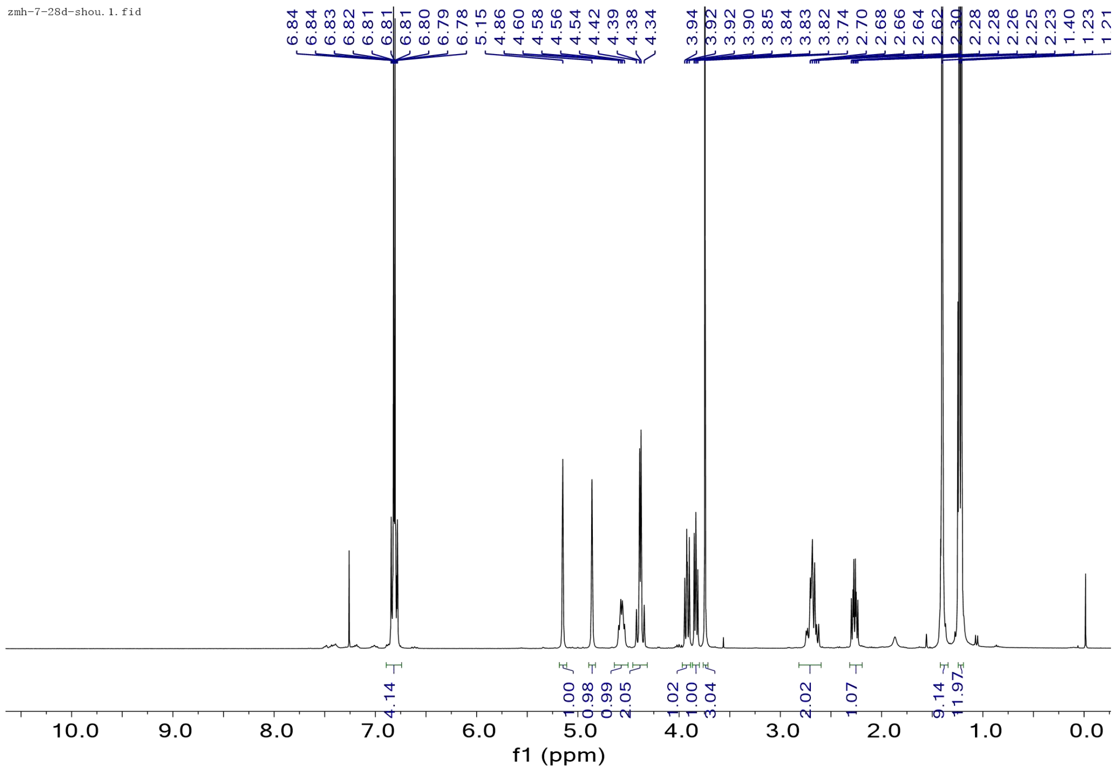

zmh-7-28d-shou.1.fid
6.84
6.84
6.83
6.82
6.81
6.81
6.81
6.80
6.79
6.78
5.15
4.86
4.60
4.58
4.56
4.54
4.42
4.39
4.38
4.34
3.94
3.92
3.92
3.90
3.85
3.84
3.83
3.82
3.74
2.70
2.68
2.66
2.64
2.62
2.30
2.28
2.28
2.26
2.25
2.23
1.40
1.23
1.21
4.14
1.00
0.98
0.99
2.05
1.02
1.00
3.04
2.02
1.07
9.14
11.97
10.0
9.0
8.0
7.0
6.0
5.0
4.0
3.0
2.0
1.0
0.0
f1 (ppm)

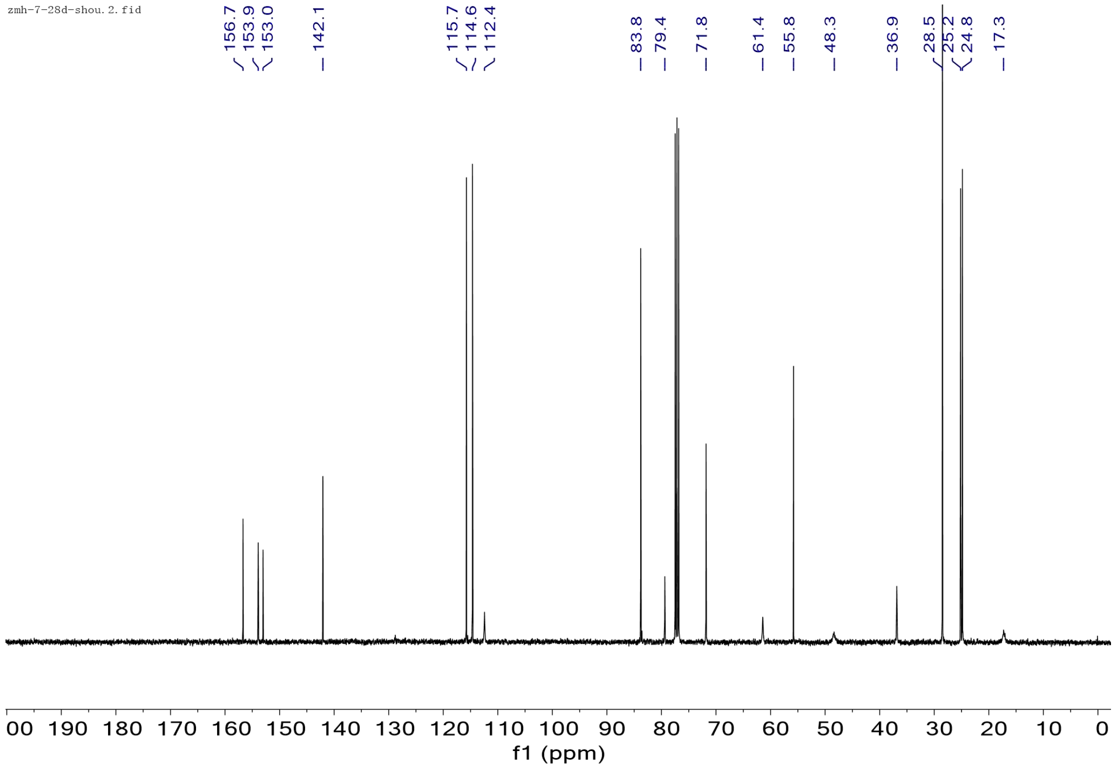

zmh-7-28d-shou.2.fid
156.7
153.9
153.0
142.1
115.7
114.6
112.4
83.8
79.4
71.8
61.4
55.8
48.3
36.9
28.5
25.2
24.8
17.3
00
190
180
170
160
150
140
130
120
110
100
90
80
70
60
50
40
30
20
10
0
f1 (ppm)

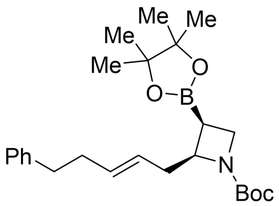

5a

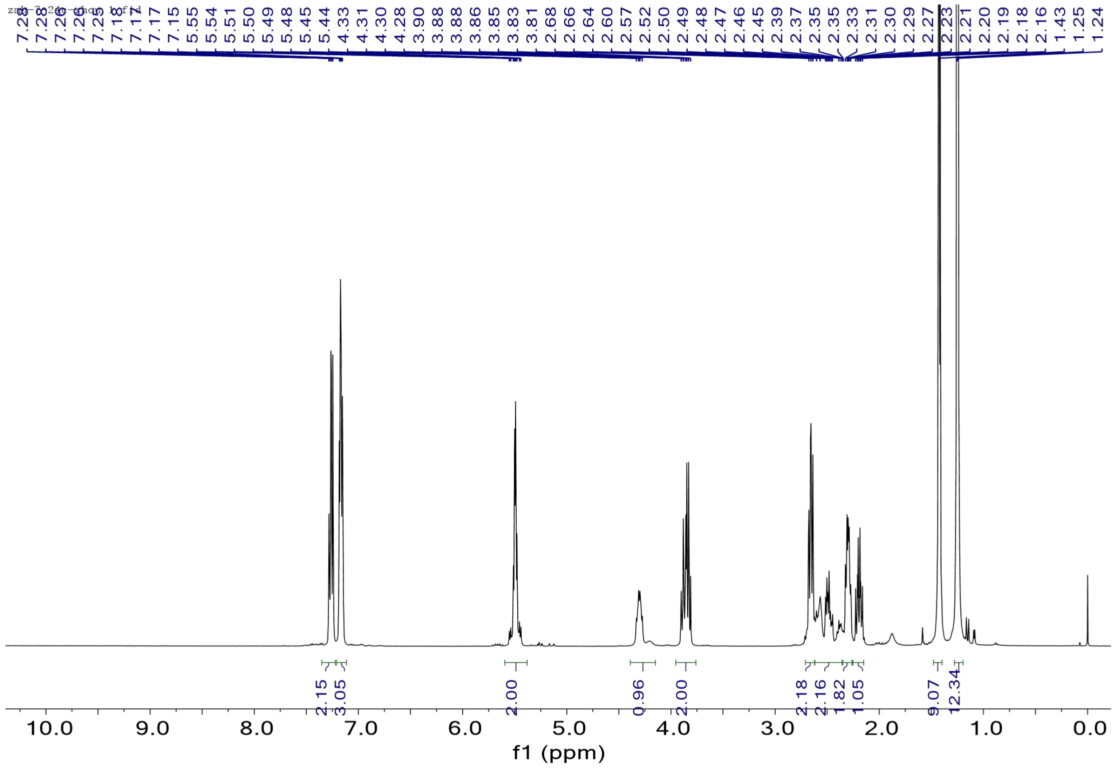

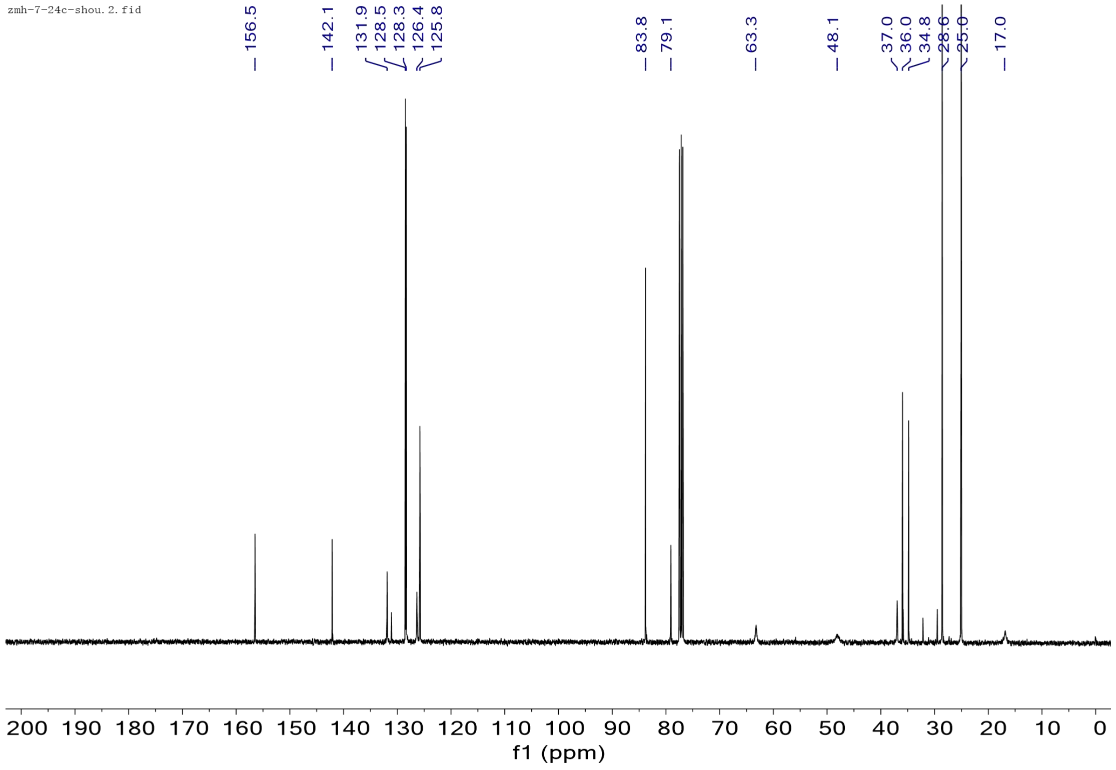

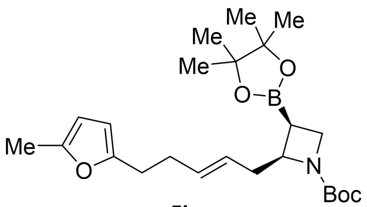
Me
Me
Me
Me
O
O
B
Me
O
N
Boc

5b

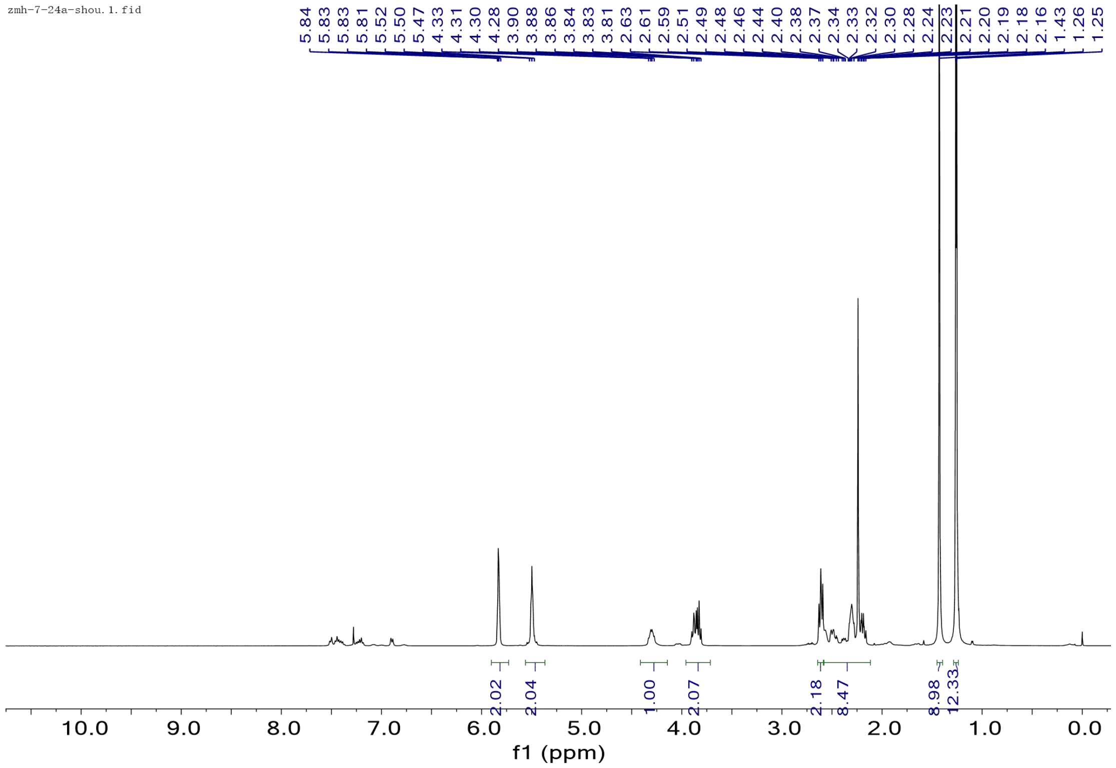
zmh-7-24a-shou.1.fid
5.84 5.83 5.83 5.81 5.52 5.50 5.47 4.33 4.31 4.30 4.28 3.90 3.88 3.86 3.84 3.83 3.81 2.63 2.61 2.59 2.51 2.49 2.48 2.46 2.44 2.40 2.38 2.37 2.34 2.33 2.32 2.30 2.28 2.24 2.23 2.21 2.20 2.19 2.18 2.16 1.43 1.26 1.25
2.02 2.04 1.00 2.07 2.18 8.47 8.98 12.33
10.0 9.0 8.0 7.0 6.0 5.0 4.0 3.0 2.0 1.0 0.0
f1 (ppm)

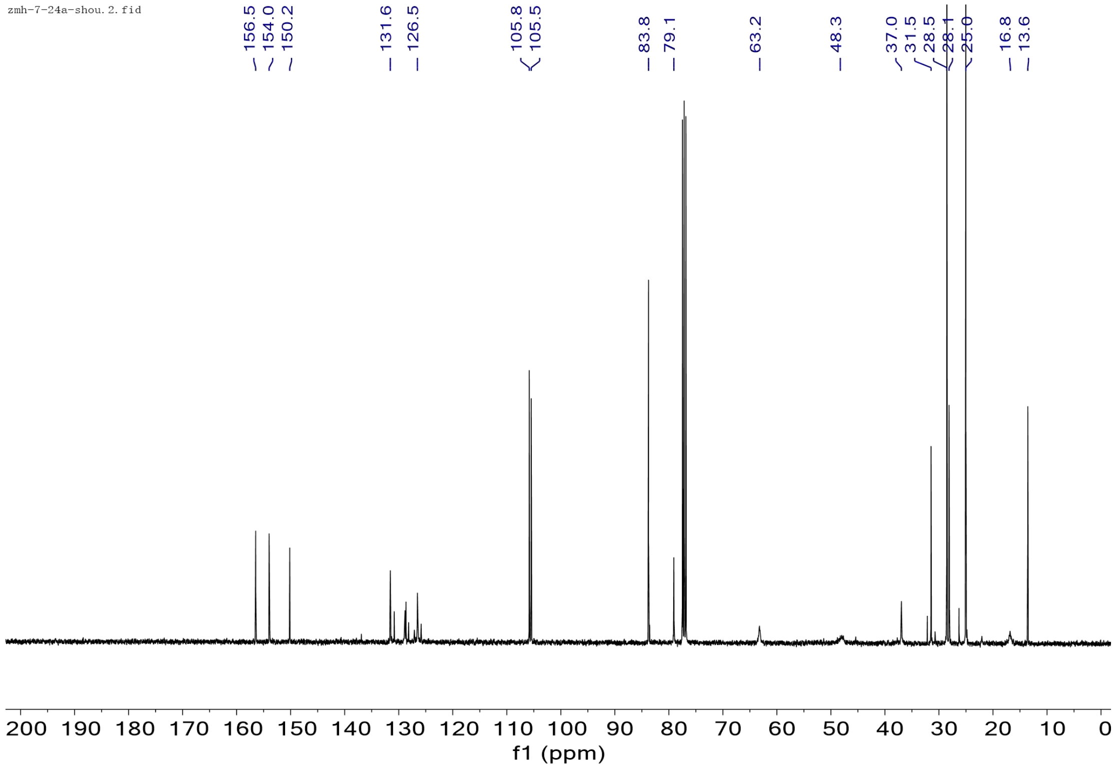
zmh-7-24a-shou.2.fid
156.5 154.0 150.2 131.6 126.5 105.8 105.5 83.8 79.1 63.2 48.3 37.0 31.5 28.5 28.1 25.0 16.8 13.6
200 190 180 170 160 150 140 130 120 110 100 90 80 70 60 50 40 30 20 10 0
f1 (ppm)

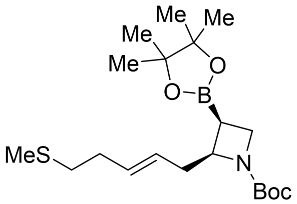
Me
Me
Me
Me
O
O
B
MeS
N
Boc

5c

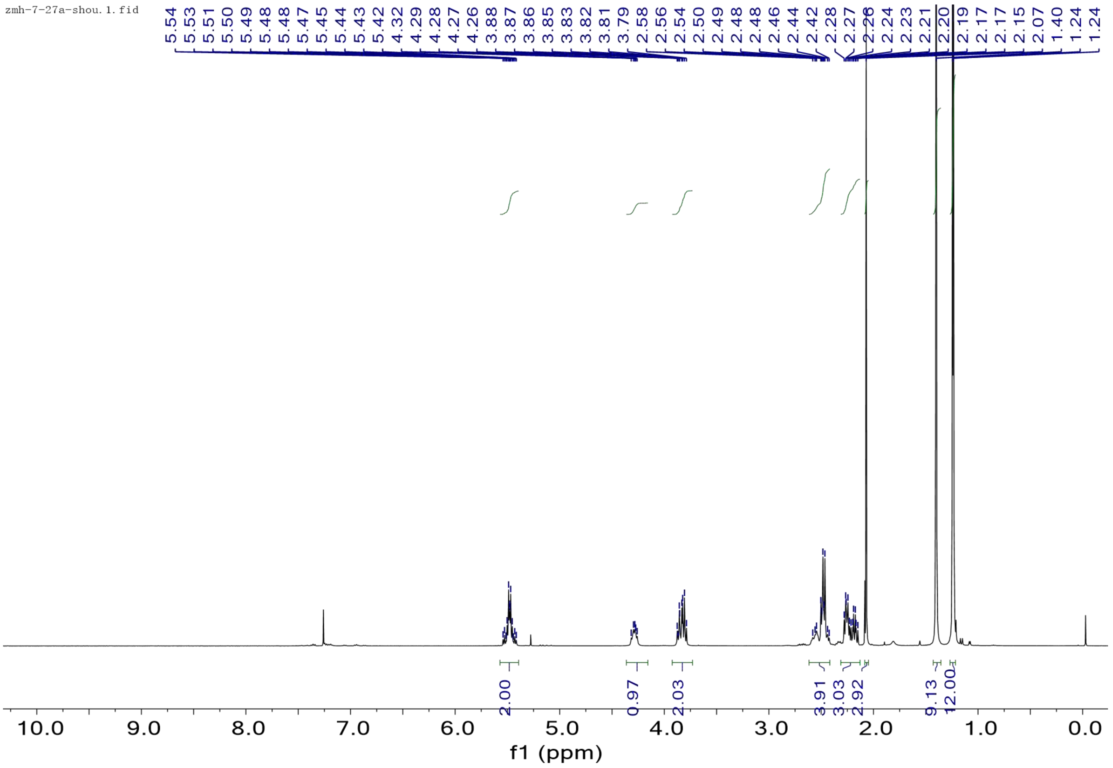
zmh-7-27a-shou.1.fid
5.54 5.53 5.51 5.50 5.49 5.48 5.48 5.47 5.45 5.44 5.43 5.42 4.32 4.29 4.28 4.27 4.26 3.88 3.87 3.86 3.85 3.83 3.82 3.81 3.79 2.58 2.56 2.54 2.50 2.49 2.48 2.48 2.46 2.44 2.42 2.28 2.27 2.26 2.24 2.23 2.21 2.20 2.19 2.17 2.15 2.07 1.40 1.24 1.24
2.00
0.97
2.03
3.91
3.03
2.92
9.13
12.00
10.0 9.0 8.0 7.0 6.0 5.0 4.0 3.0 2.0 1.0 0.0
f1 (ppm)

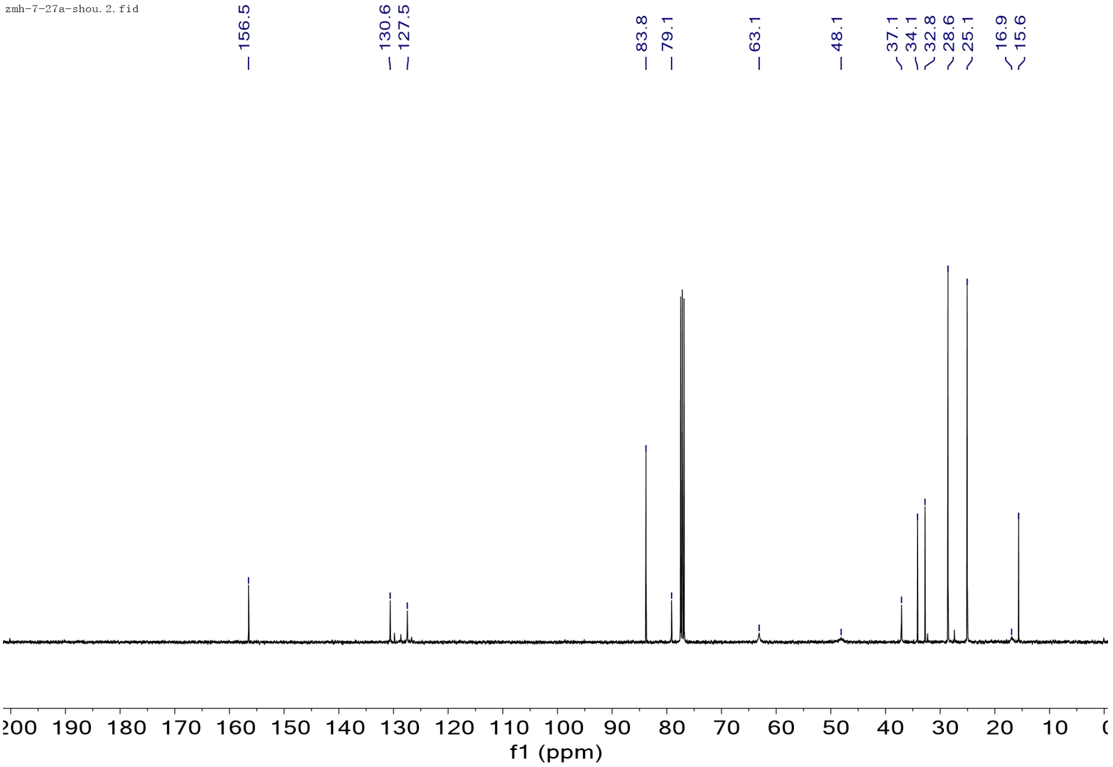
zmh-7-27a-shou.2.fid
156.5
130.6
127.5
83.8
79.1
63.1
48.1
37.1
34.1
32.8
28.6
25.1
16.9
15.6
200 190 180 170 160 150 140 130 120 110 100 90 80 70 60 50 40 30 20 10 0
f1 (ppm)

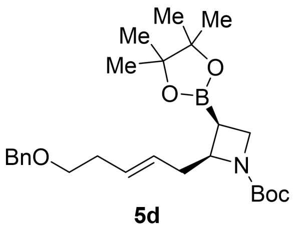
Me
Me
Me
Me
O
B
O
BnO
N
Boc

5d

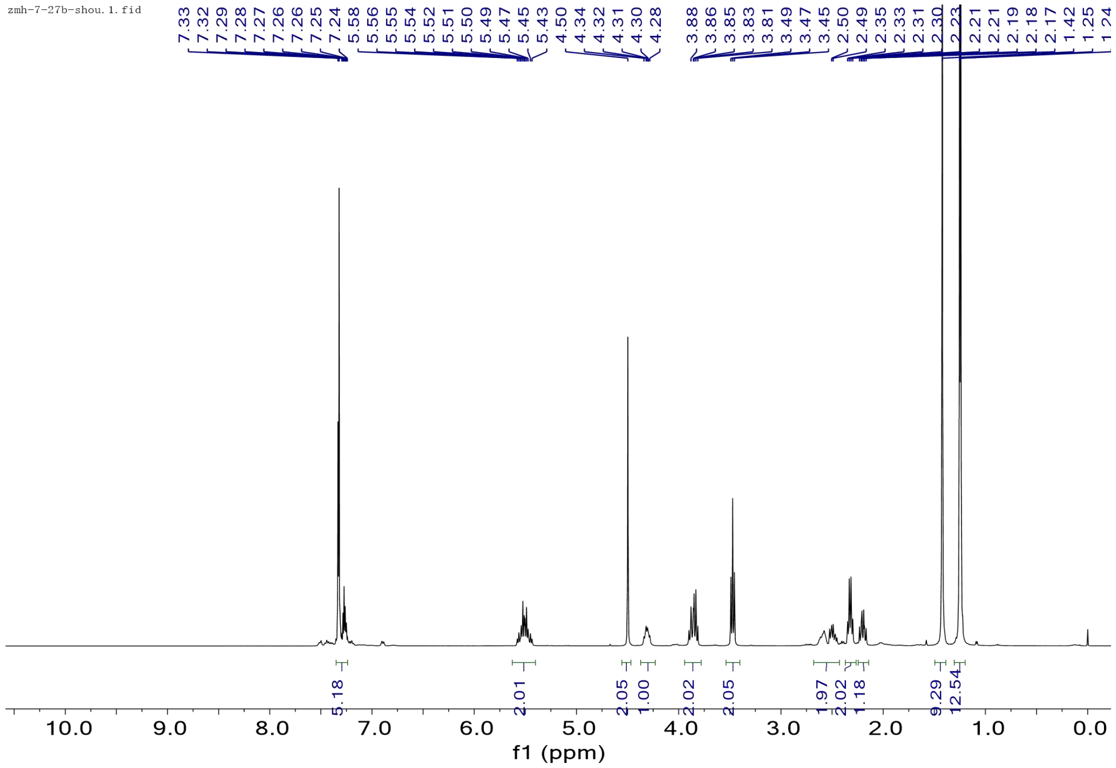
zmh-7-27b-shou.1.fid
7.33 7.32 7.29 7.28 7.27 7.26 7.26 7.25 7.24 5.58 5.56 5.55 5.54 5.52 5.51 5.50 5.49 5.47 5.45 5.43 4.50 4.34 4.32 4.31 4.30 4.28 3.88 3.86 3.85 3.83 3.81 3.49 3.47 3.45 2.50 2.49 2.35 2.33 2.31 2.30 2.23 2.21 2.21 2.19 2.18 2.17 1.42 1.25 1.24
5.18
2.01
2.05
1.00
2.02
2.05
1.97
2.02
1.18
9.29
12.54
10.0
9.0
8.0
7.0
6.0
5.0
4.0
3.0
2.0
1.0
0.0
f1 (ppm)

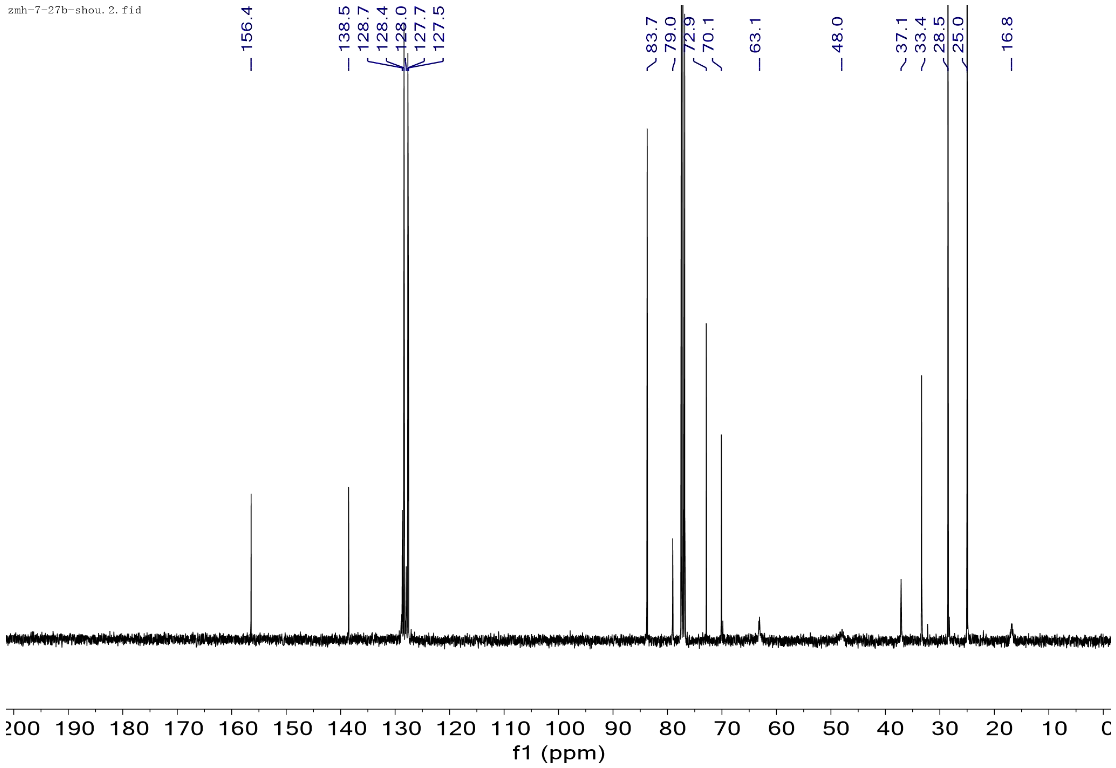
zmh-7-27b-shou.2.fid
156.4
138.5 128.7 128.4 128.0 127.7 127.5
83.7 79.0 72.9 70.1
63.1
48.0
37.1 33.4 28.5 25.0
16.8
200 190 180 170 160 150 140 130 120 110 100 90 80 70 60 50 40 30 20 10 0
f1 (ppm)

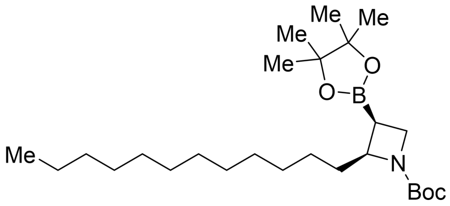
Me
Me
Me
Me
O
O
B
Me
N
Boc

5e'

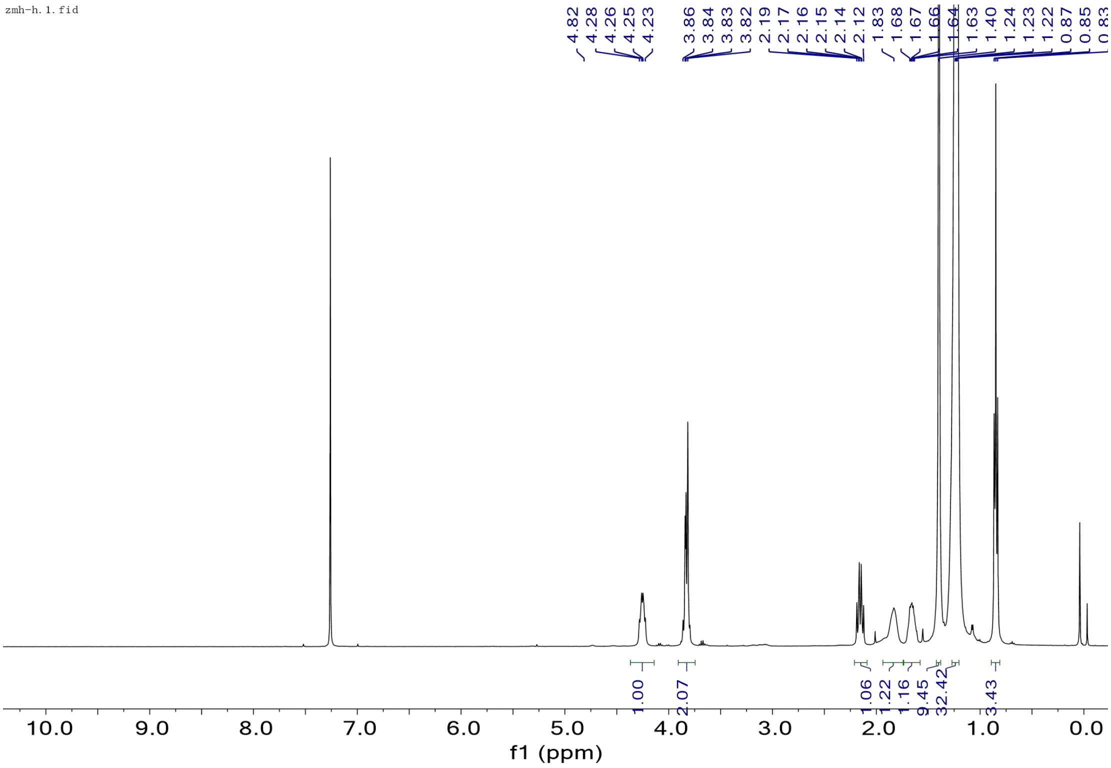
zmh-h.1.fid
4.82
4.28
4.26
4.25
4.23
3.86
3.84
3.83
3.82
2.19
2.17
2.16
2.15
2.14
2.12
1.83
1.68
1.67
1.66
1.64
1.63
1.40
1.24
1.23
1.22
0.87
0.85
0.83
1.00
2.07
1.06
1.22
1.16
9.45
32.42
3.43
10.0 9.0 8.0 7.0 6.0 5.0 4.0 3.0 2.0 1.0 0.0
f1 (ppm)

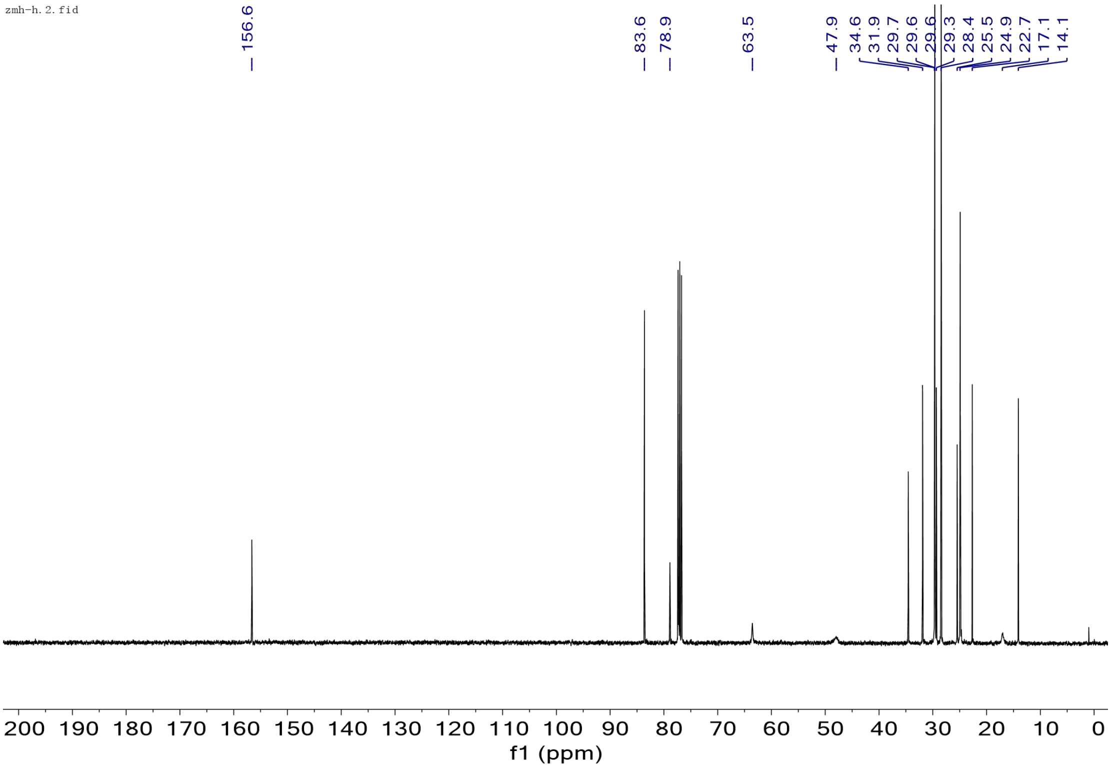
zmh-h.2.fid
156.6
83.6
78.9
63.5
47.9
34.6
31.9
29.7
29.6
29.6
29.3
28.4
25.5
24.9
22.7
17.1
14.1
200 190 180 170 160 150 140 130 120 110 100 90 80 70 60 50 40 30 20 10 0
f1 (ppm)

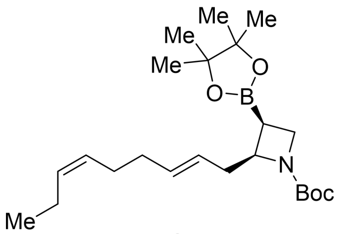
Me
Me
Me
Me
O
O
B
N
Boc
Me

5f

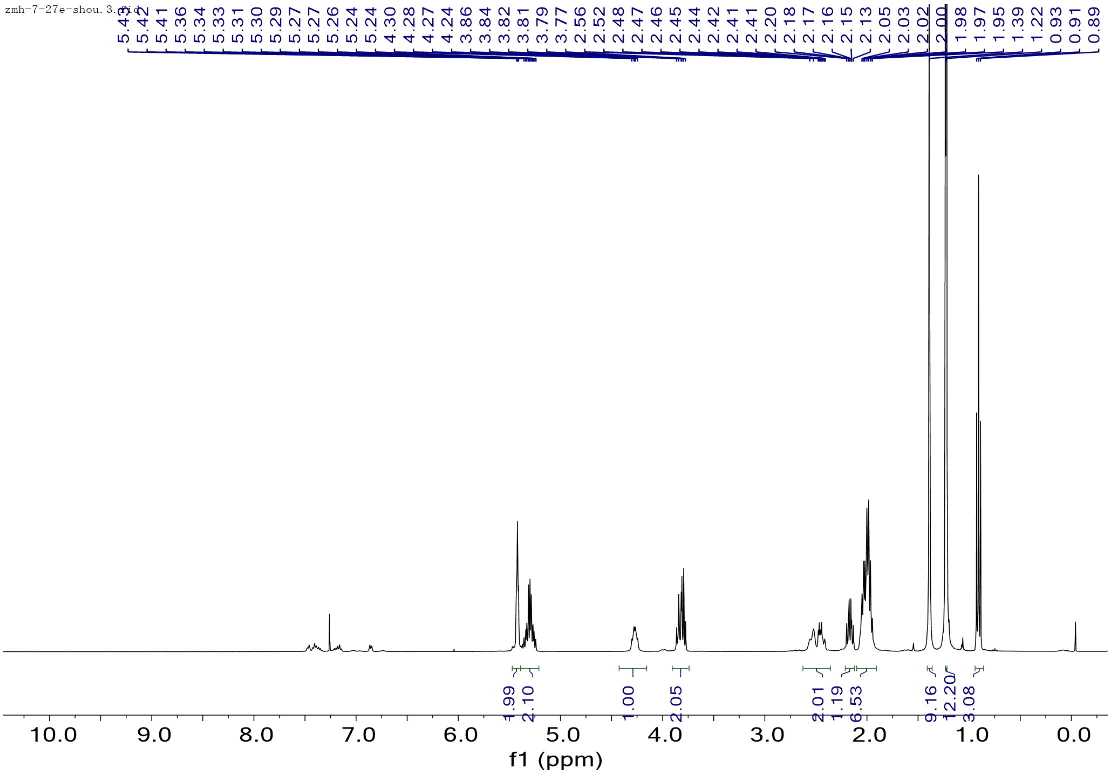
zmh-7-27e-shou.3.fid
5.43 5.42 5.41 5.36 5.34 5.33 5.31 5.30 5.29 5.27 5.27 5.26 5.24 5.24 4.30 4.28 4.27 4.24 3.86 3.84 3.82 3.81 3.79 3.77 2.56 2.52 2.48 2.47 2.46 2.45 2.44 2.42 2.41 2.41 2.20 2.18 2.17 2.16 2.15 2.13 2.05 2.03 2.02 2.00 1.98 1.97 1.95 1.39 1.22 0.93 0.91 0.89
1.99 2.10 1.00 2.05 2.01 1.19 6.53 9.16 12.20 3.08
10.0 9.0 8.0 7.0 6.0 5.0 4.0 3.0 2.0 1.0 0.0
f1 (ppm)

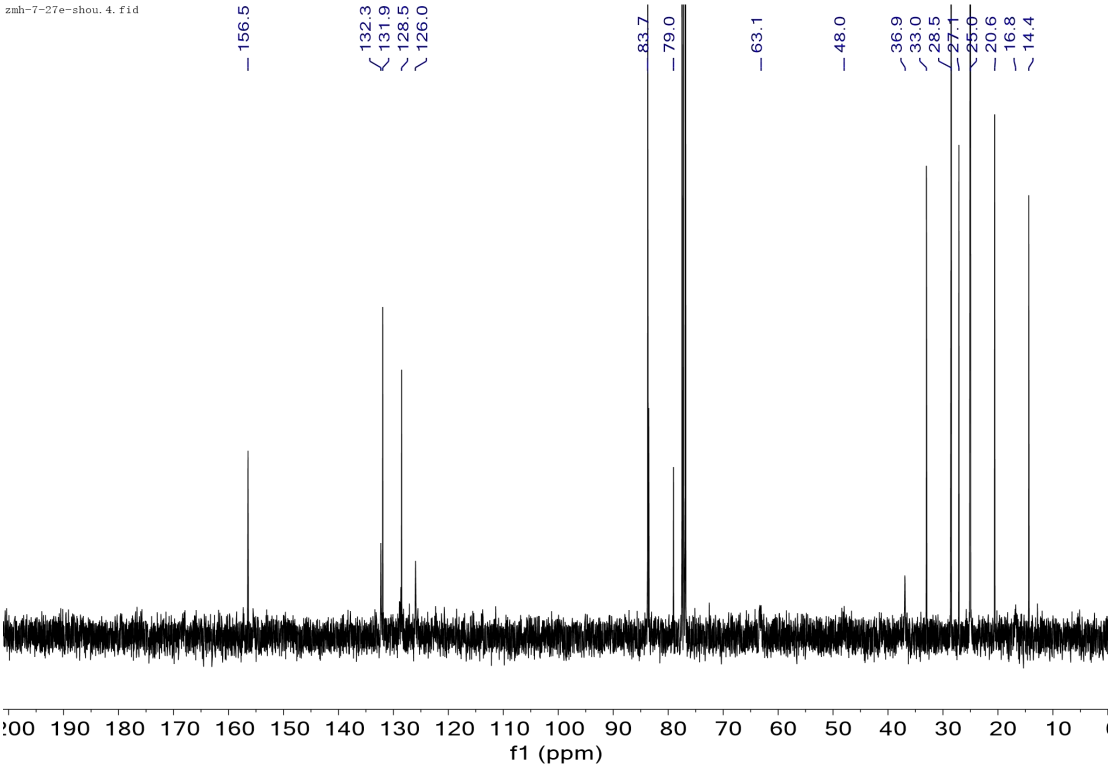
zmh-7-27e-shou.4.fid
156.5
132.3 131.9 128.5 126.0
83.7
79.0
63.1
48.0
36.9 33.0 28.5 27.1 25.0 20.6 16.8 14.4
190 180 170 160 150 140 130 120 110 100 90 80 70 60 50 40 30 20 10
f1 (ppm)

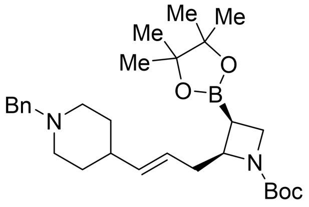

5g

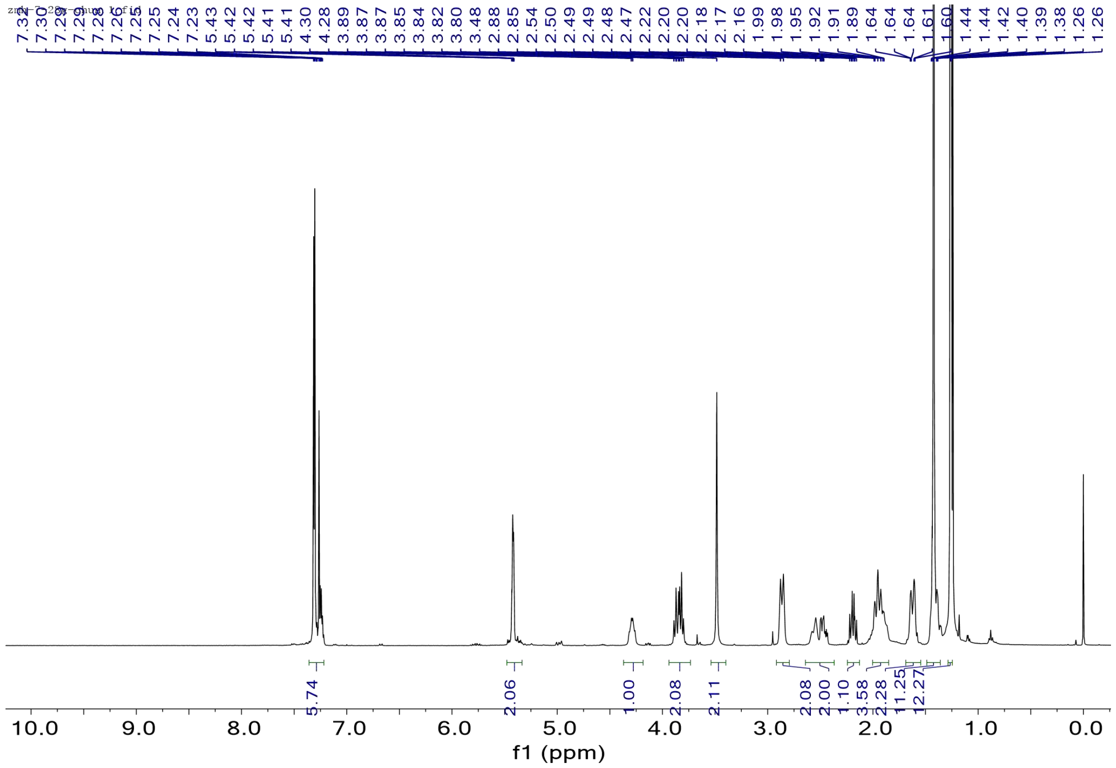
zmh-7-28g-shou.1.fid
7.32 7.30 7.29 7.28 7.26 7.25 7.25 7.24 7.23 5.43 5.42 5.42 5.41 5.41 4.30 4.28 3.89 3.87 3.85 3.84 3.82 3.80 3.48 2.88 2.85 2.54 2.50 2.49 2.49 2.48 2.47 2.22 2.20 2.20 2.18 2.17 2.16 1.99 1.98 1.95 1.92 1.91 1.89 1.64 1.64 1.64 1.61 1.60 1.44 1.44 1.42 1.40 1.39 1.38 1.26 1.26
5.74 2.06 1.00 2.08 2.11 2.08 2.00 1.10 3.58 2.28 11.25 12.27
10.0 9.0 8.0 7.0 6.0 5.0 4.0 3.0 2.0 1.0 0.0
f1 (ppm)

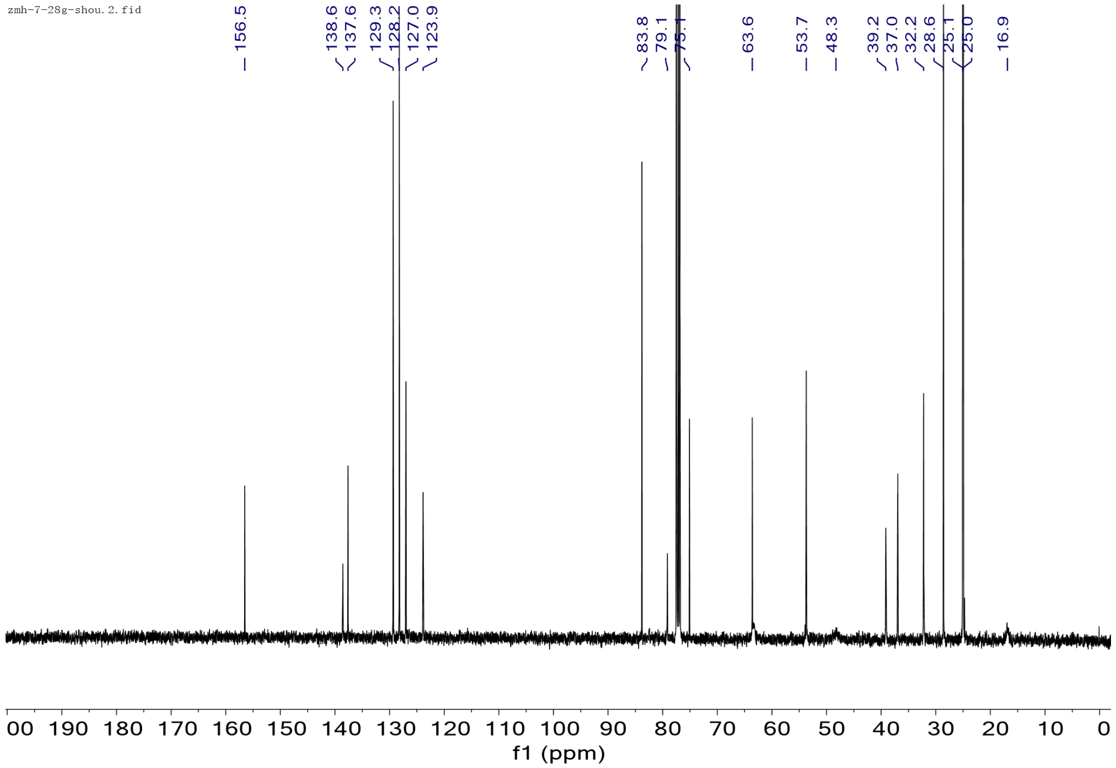
zmh-7-28g-shou.2.fid
156.5 138.6 137.6 129.3 128.2 127.0 123.9 83.8 79.1 75.1 63.6 53.7 48.3 39.2 37.0 32.2 28.6 25.1 25.0 16.9
00 190 180 170 160 150 140 130 120 110 100 90 80 70 60 50 40 30 20 10 0
f1 (ppm)

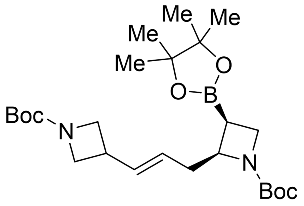
Me
Me
Me
Me
O
O
B
Boc
N
N
Boc

5h

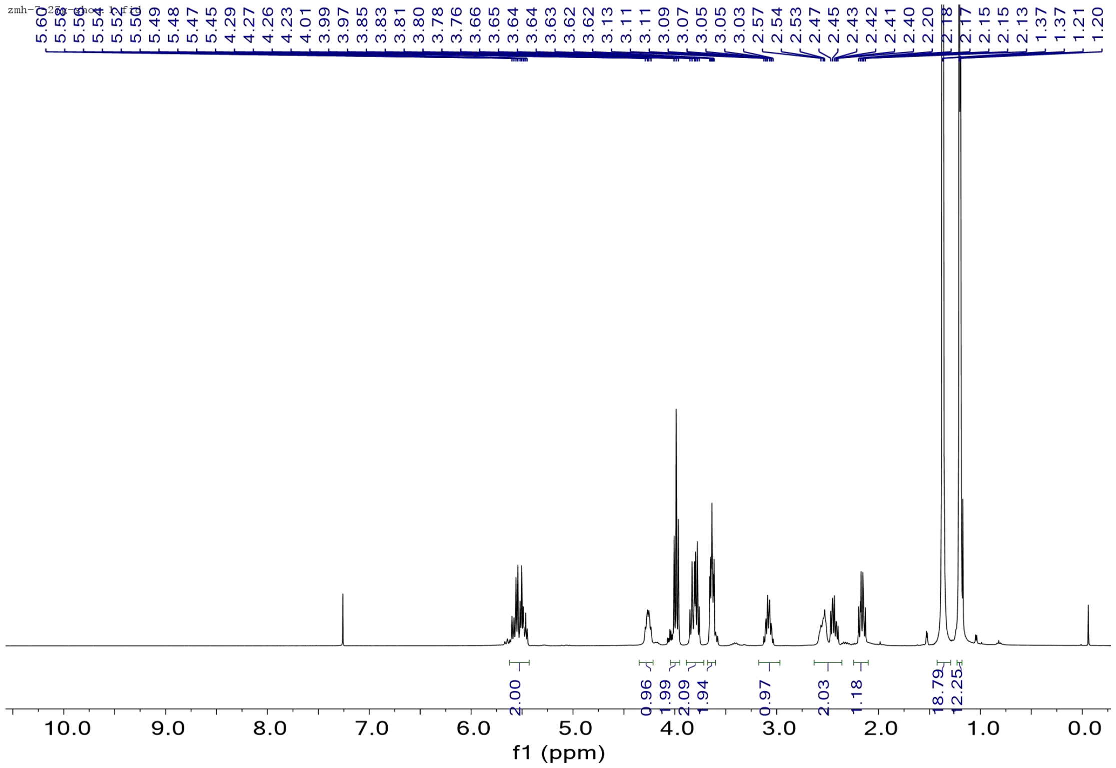
5.60
5.58
5.56
5.54
5.52
5.50
5.49
5.48
5.47
5.45
4.29
4.27
4.26
4.23
4.01
3.99
3.97
3.85
3.83
3.81
3.80
3.78
3.76
3.66
3.65
3.64
3.64
3.63
3.62
3.62
3.13
3.11
3.11
3.09
3.07
3.05
3.03
2.57
2.54
2.53
2.47
2.45
2.43
2.42
2.41
2.40
2.20
2.18
2.17
2.15
2.15
2.13
1.37
1.37
1.21
1.20
2.00
0.96
1.99
2.09
1.94
0.97
2.03
1.18
18.79
12.25
10.0 9.0 8.0 7.0 6.0 5.0 4.0 3.0 2.0 1.0 0.0
f1 (ppm)

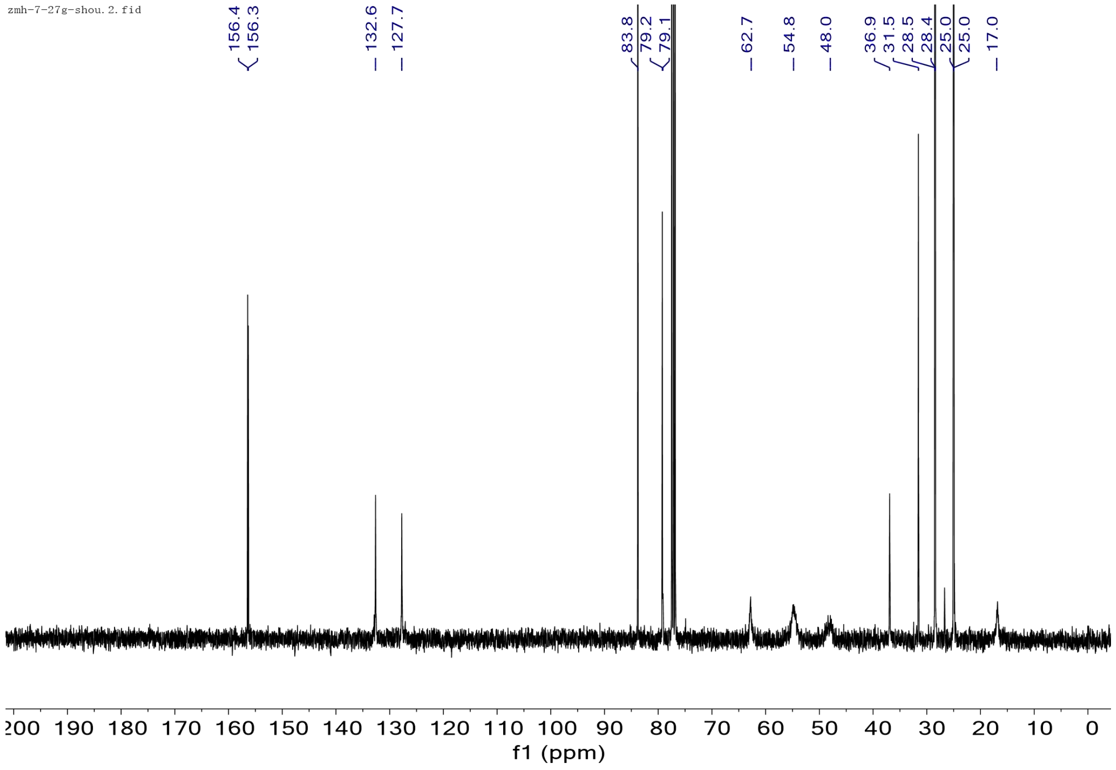
zmh-7-27g-shou.2.fid
156.4
156.3
132.6
127.7
83.8
79.2
79.1
62.7
54.8
48.0
36.9
31.5
28.5
28.4
25.0
25.0
17.0
200 190 180 170 160 150 140 130 120 110 100 90 80 70 60 50 40 30 20 10 0
f1 (ppm)

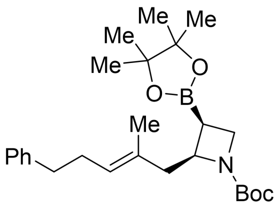

Me
Me
Me
Me
O
O
B
Me
Ph
N
Boc

5i

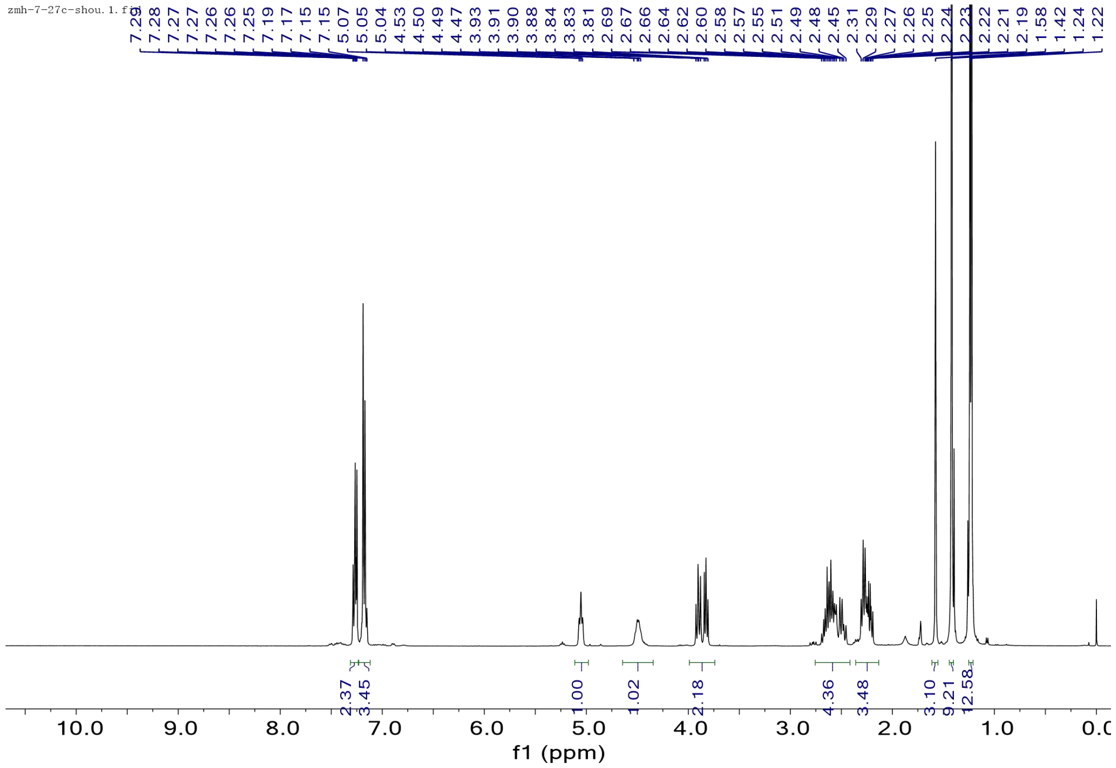

zmh-7-27c-shou.1.fid
7.29
7.28
7.27
7.27
7.26
7.26
7.25
7.19
7.17
7.15
7.15
5.07
5.05
5.04
4.53
4.50
4.49
4.47
3.93
3.91
3.90
3.88
3.84
3.83
3.81
2.69
2.67
2.66
2.64
2.62
2.60
2.58
2.57
2.55
2.51
2.49
2.48
2.45
2.31
2.29
2.27
2.26
2.25
2.24
2.23
2.22
2.21
2.19
1.58
1.42
1.24
1.22
2.37
3.45
1.00
1.02
2.18
4.36
3.48
3.10
9.21
12.58
10.0
9.0
8.0
7.0
6.0
5.0
4.0
3.0
2.0
1.0
0.0
f1 (ppm)

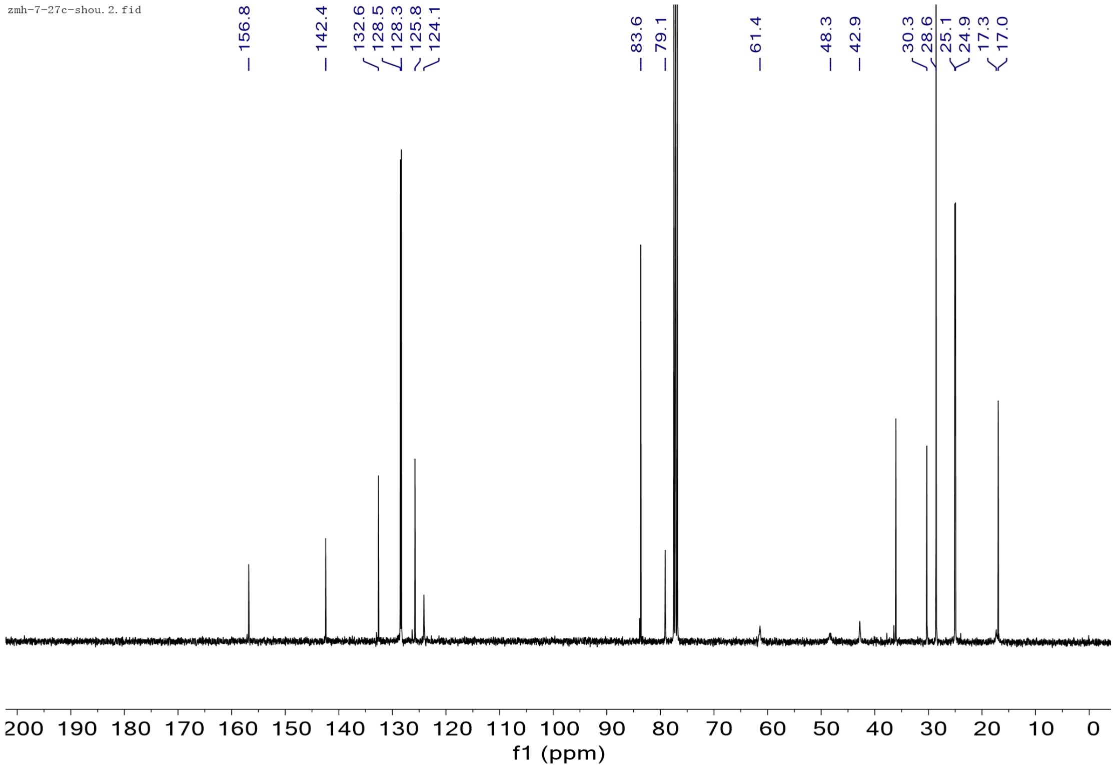

zmh-7-27c-shou.2.fid
156.8
142.4
132.6
128.5
128.3
125.8
124.1
83.6
79.1
61.4
48.3
42.9
30.3
28.6
25.1
24.9
17.3
17.0
200
190
180
170
160
150
140
130
120
110
100
90
80
70
60
50
40
30
20
10
0
f1 (ppm)

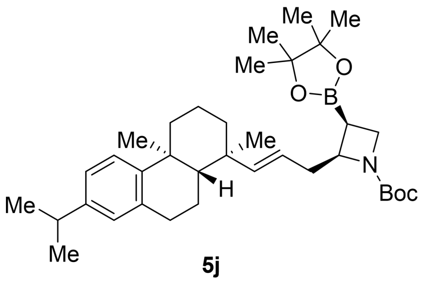

5j

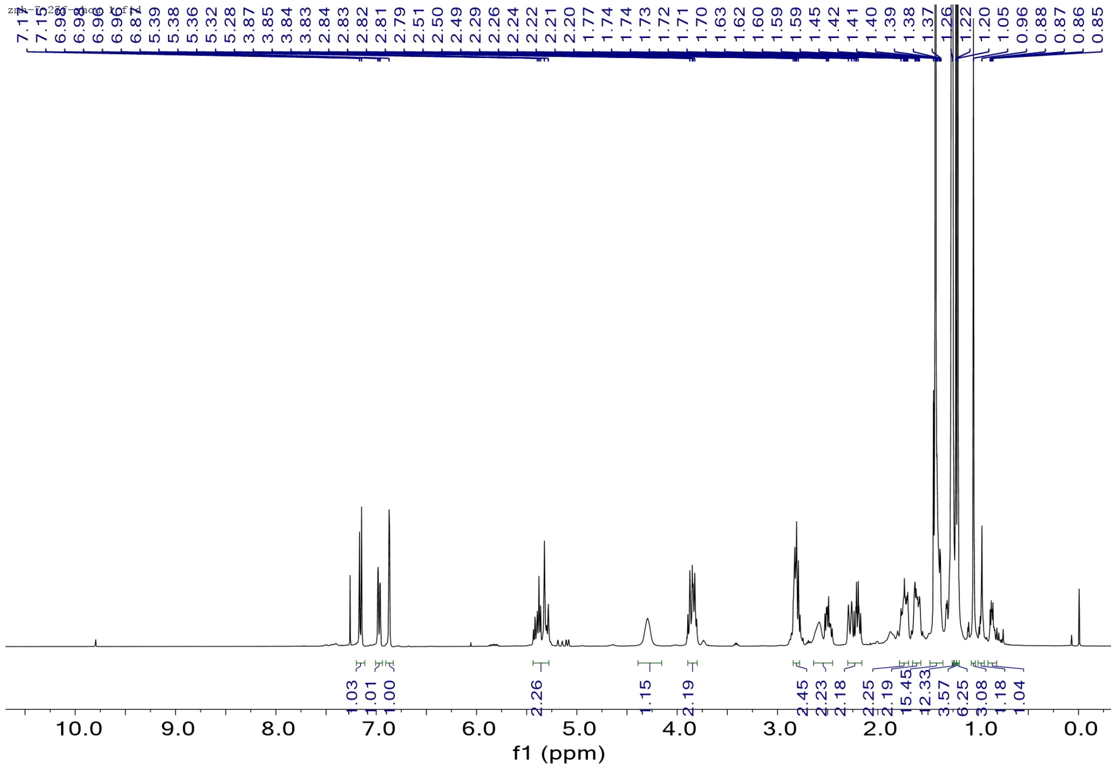

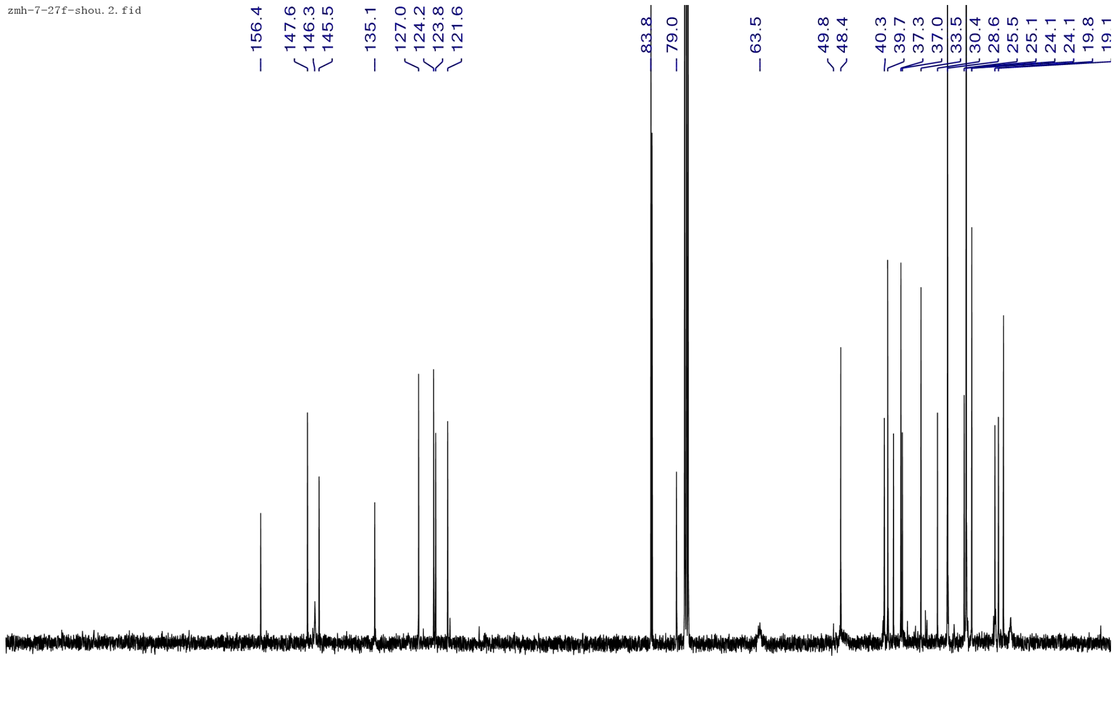

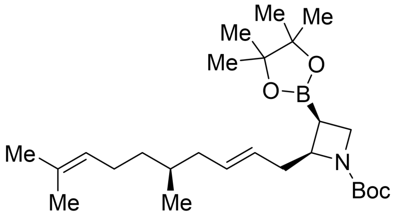
Me
Me
Me
Me
O
O
B
N
Boc
Me
Me
Me

5k

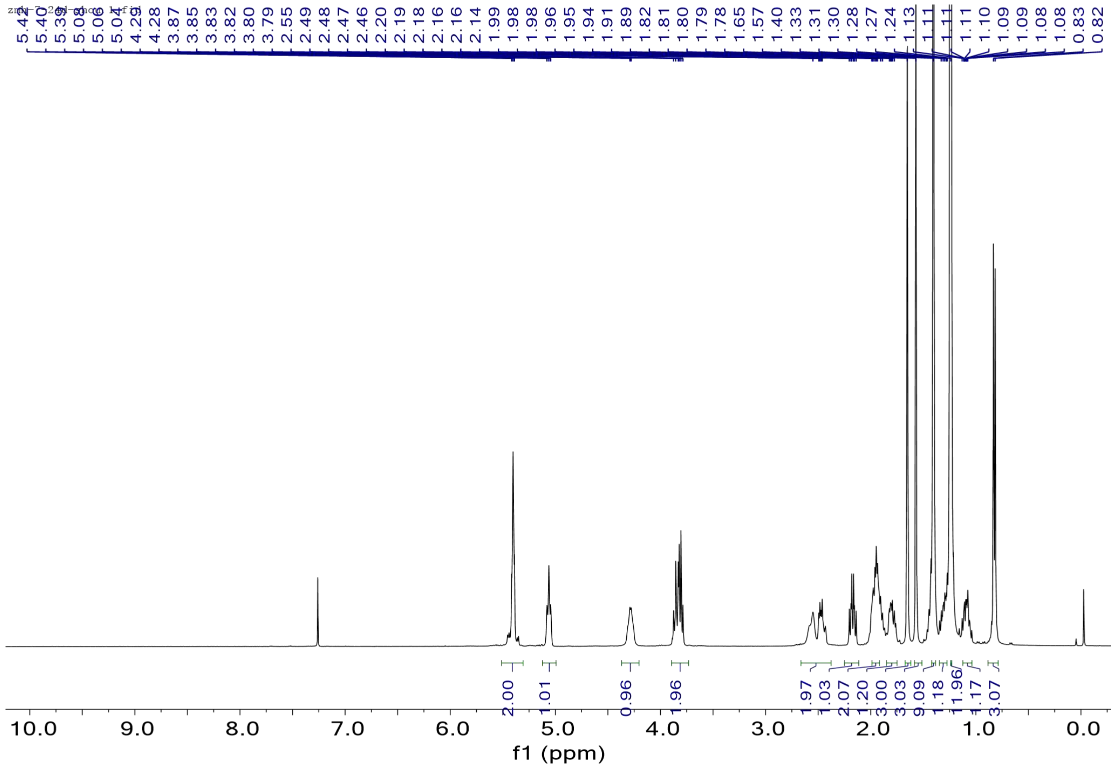
5.42 5.40 5.39 5.08 5.06 5.04 4.29 4.28 3.87 3.85 3.83 3.82 3.80 3.79 2.55 2.49 2.48 2.47 2.46 2.20 2.19 2.18 2.16 2.16 2.14 1.99 1.98 1.98 1.96 1.95 1.94 1.91 1.89 1.82 1.81 1.80 1.79 1.78 1.65 1.57 1.40 1.33 1.31 1.30 1.28 1.27 1.24 1.13 1.11 1.11 1.11 1.10 1.09 1.08 1.08 0.83 0.82
2.00 1.01 0.96 1.96 1.97 1.03 2.07 1.20 3.00 3.03 9.09 1.18 11.96 1.17 3.07
10.0 9.0 8.0 7.0 6.0 5.0 4.0 3.0 2.0 1.0 0.0
f1 (ppm)

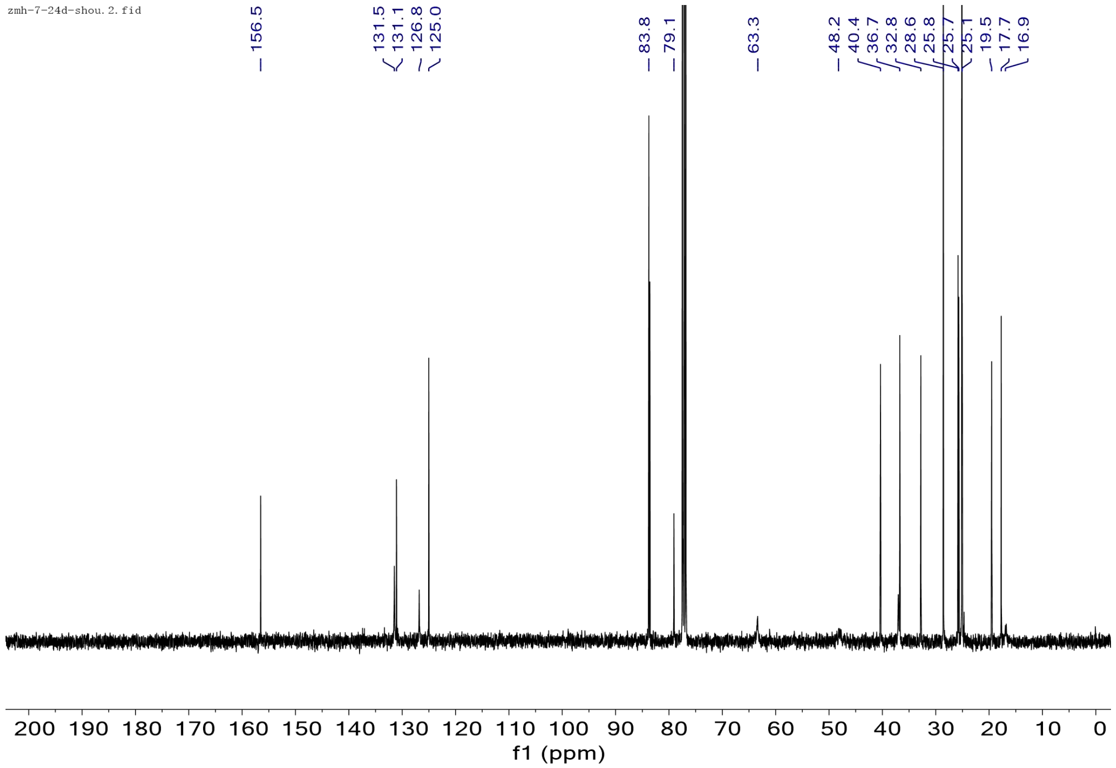
zmh-7-24d-shou.2.fid
156.5
131.5 131.1 126.8 125.0
83.8 79.1
63.3
48.2 40.4 36.7 32.8 28.6 25.8 25.7 25.1 19.5 17.7 16.9
200 190 180 170 160 150 140 130 120 110 100 90 80 70 60 50 40 30 20 10 0
f1 (ppm)

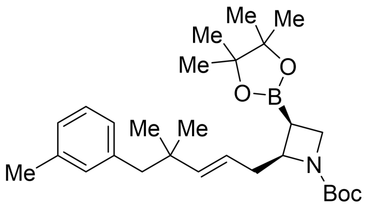
Me Me
Me
Me
O
B
O
Me Me
Me
N
Boc

5l

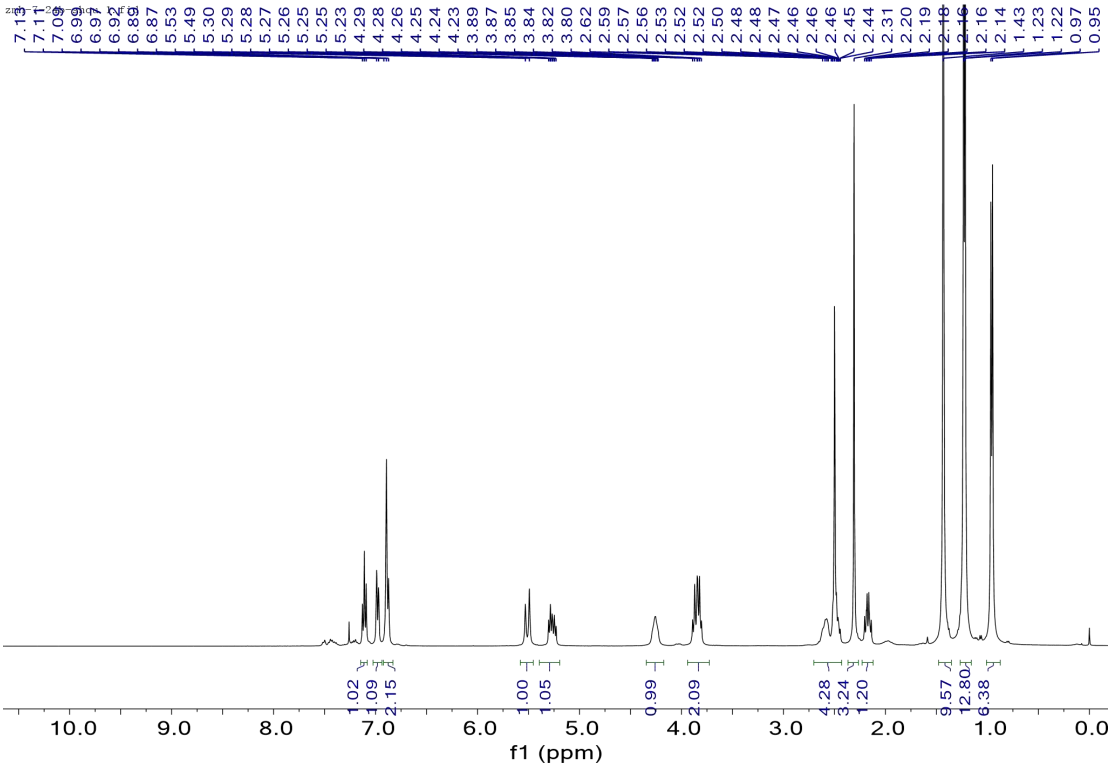
zmh-7-24b-shou.1.fid
7.13 7.11 7.09 6.93 6.92 6.89 6.87 5.53 5.49 5.30 5.29 5.28 5.27 5.26 5.25 5.25 5.23 4.29 4.28 4.26 4.25 4.24 4.23 3.89 3.87 3.85 3.84 3.82 3.80 2.62 2.59 2.57 2.56 2.53 2.52 2.52 2.50 2.48 2.48 2.47 2.46 2.46 2.45 2.44 2.31 2.20 2.19 2.18 2.16 2.16 2.14 1.43 1.23 1.22 0.97 0.95
1.02 1.09 2.15 1.00 1.05 0.99 2.09 4.28 3.24 1.20 9.57 12.80 6.38
10.0 9.0 8.0 7.0 6.0 5.0 4.0 3.0 2.0 1.0 0.0
f1 (ppm)

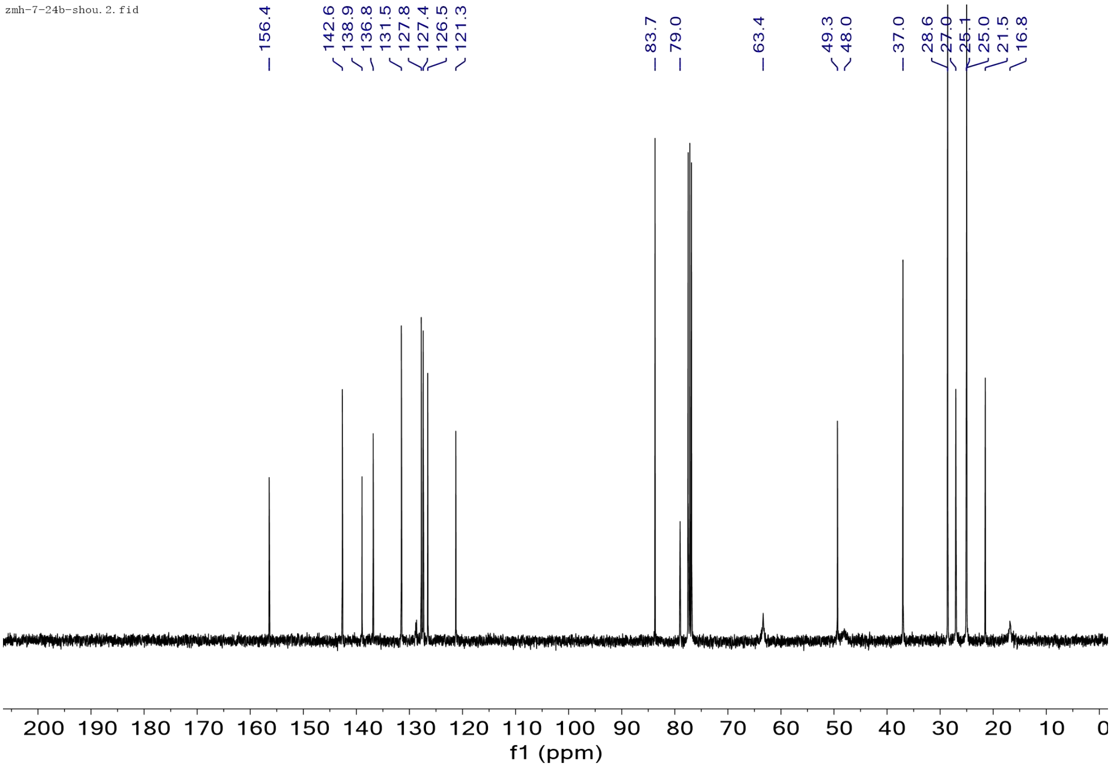
zmh-7-24b-shou.2.fid
156.4
142.6 138.9 136.8 131.5 127.8 127.4 126.5 121.3
83.7 79.0
63.4
49.3 48.0
37.0 28.6 27.0 25.1 25.0 21.5 16.8
200 190 180 170 160 150 140 130 120 110 100 90 80 70 60 50 40 30 20 10 0
f1 (ppm)

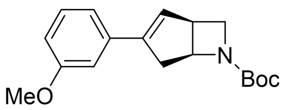
MeO
N
Boc

6

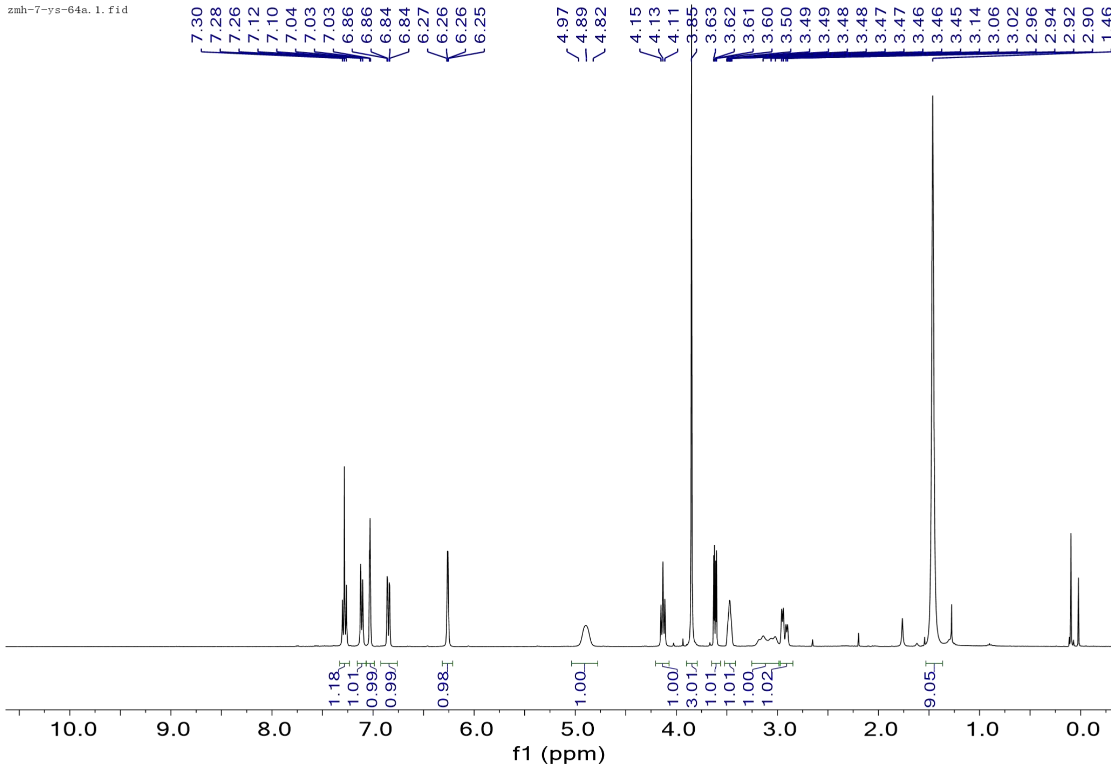
zmh-7-ys-64a.1.fid
7.30 7.28 7.26 7.12 7.10 7.04 7.03 7.03 6.86 6.86 6.84 6.84 6.27 6.26 6.26 6.25
4.97 4.89 4.82
4.15 4.13 4.11 3.85 3.63 3.62 3.61 3.60 3.50 3.49 3.49 3.48 3.48 3.47 3.47 3.46 3.46 3.45 3.14 3.06 3.02 2.96 2.94 2.92 2.90 1.46
1.18 1.01 0.99 0.99 0.98 1.00 1.00 3.01 1.01 1.00 1.02 9.05
10.0 9.0 8.0 7.0 6.0 5.0 4.0 3.0 2.0 1.0 0.0
f1 (ppm)

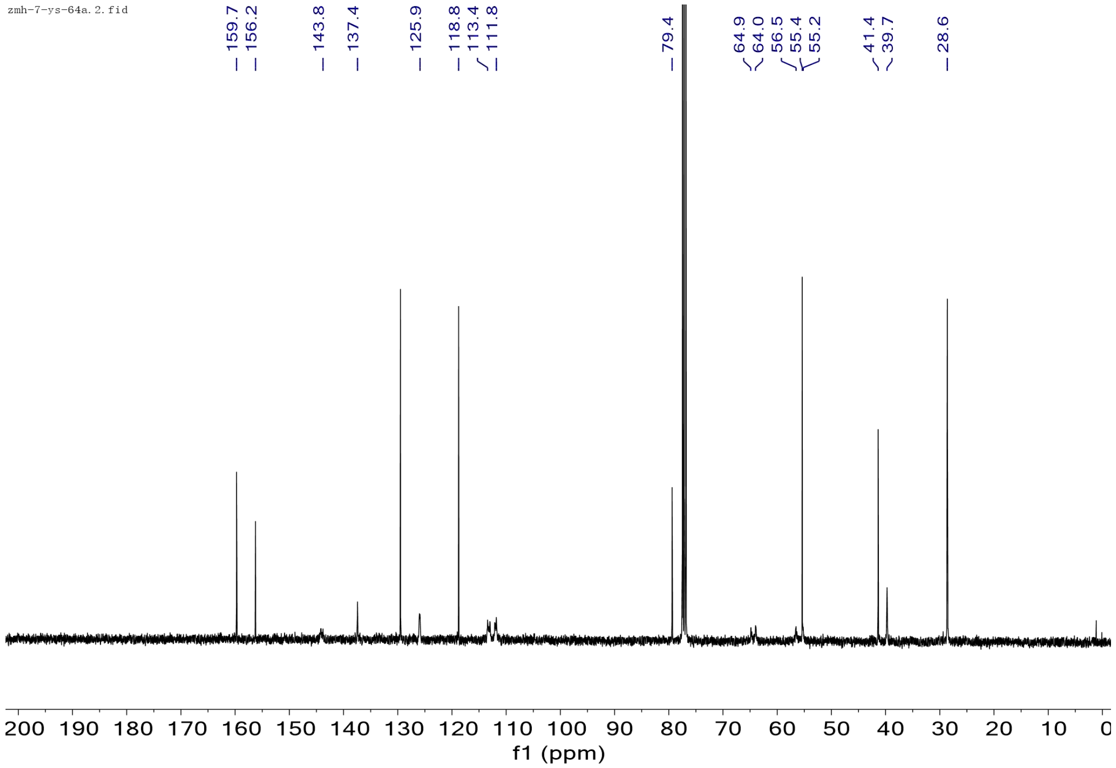
zmh-7-ys-64a.2.fid
159.7 156.2 143.8 137.4 125.9 118.8 113.4 111.8
79.4
64.9 64.0 56.5 55.4 55.2 41.4 39.7 28.6
200 190 180 170 160 150 140 130 120 110 100 90 80 70 60 50 40 30 20 10 0
f1 (ppm)

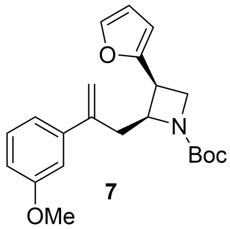
O
N
Boc
OMe
7

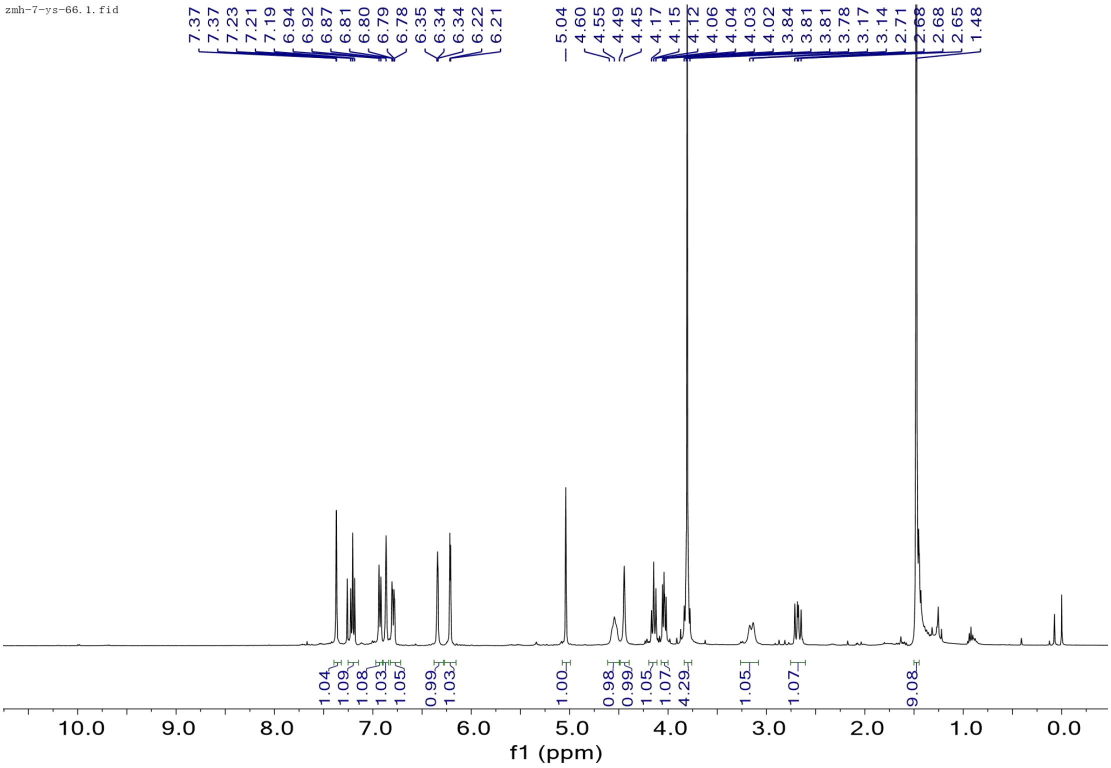
zmh-7-ys-66.1.fid
7.37 7.37 7.23 7.21 7.19 6.94 6.92 6.87 6.81 6.80 6.79 6.78 6.35 6.34 6.34 6.22 6.21
5.04 4.60 4.55 4.49 4.45 4.17 4.15 4.12 4.06 4.04 4.03 4.02 3.84 3.81 3.81 3.78 3.17 3.14 2.71 2.68 2.68 2.65 1.48
1.04 1.09 1.08 1.03 1.05 0.99 1.03 1.00 0.98 0.99 1.05 1.07 4.29 1.05 1.07 9.08
10.0 9.0 8.0 7.0 6.0 5.0 4.0 3.0 2.0 1.0 0.0
f1 (ppm)

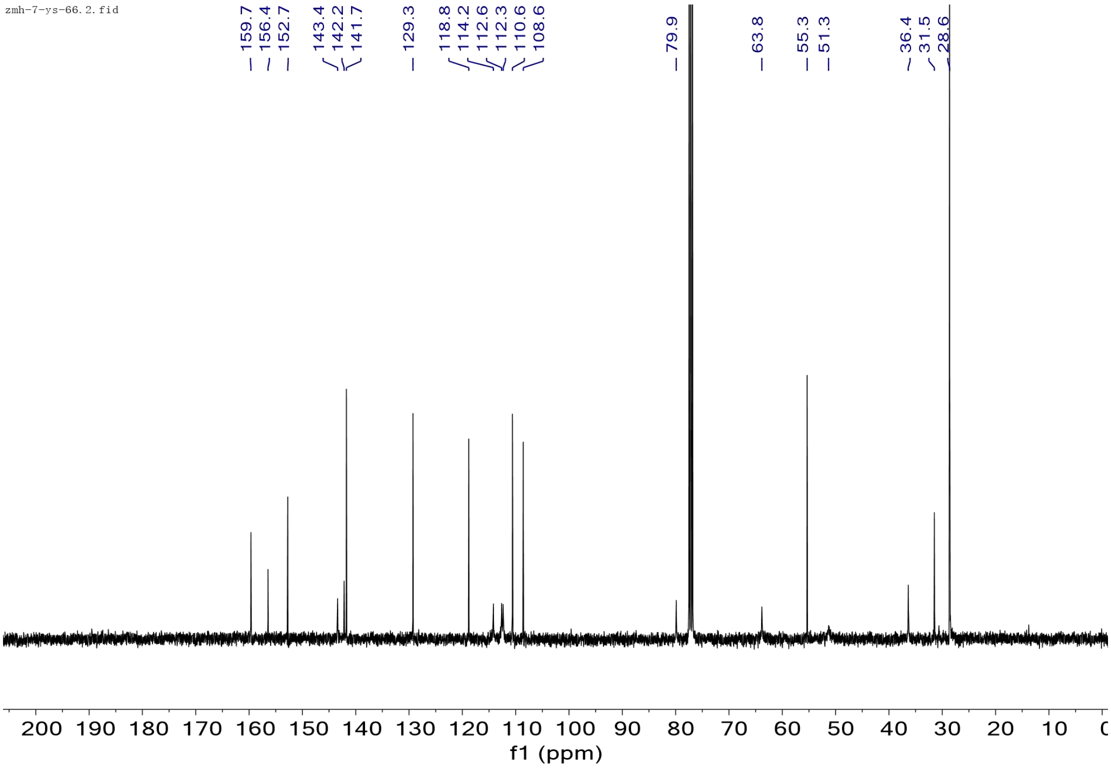
zmh-7-ys-66.2.fid
159.7 156.4 152.7 143.4 142.2 141.7 129.3 118.8 114.2 112.6 112.3 110.6 108.6
79.9 63.8 55.3 51.3 36.4 31.5 28.6
200 190 180 170 160 150 140 130 120 110 100 90 80 70 60 50 40 30 20 10
f1 (ppm)

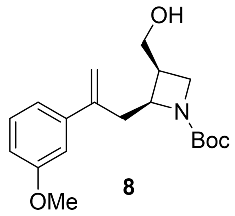
OH
N
Boc
OMe

8

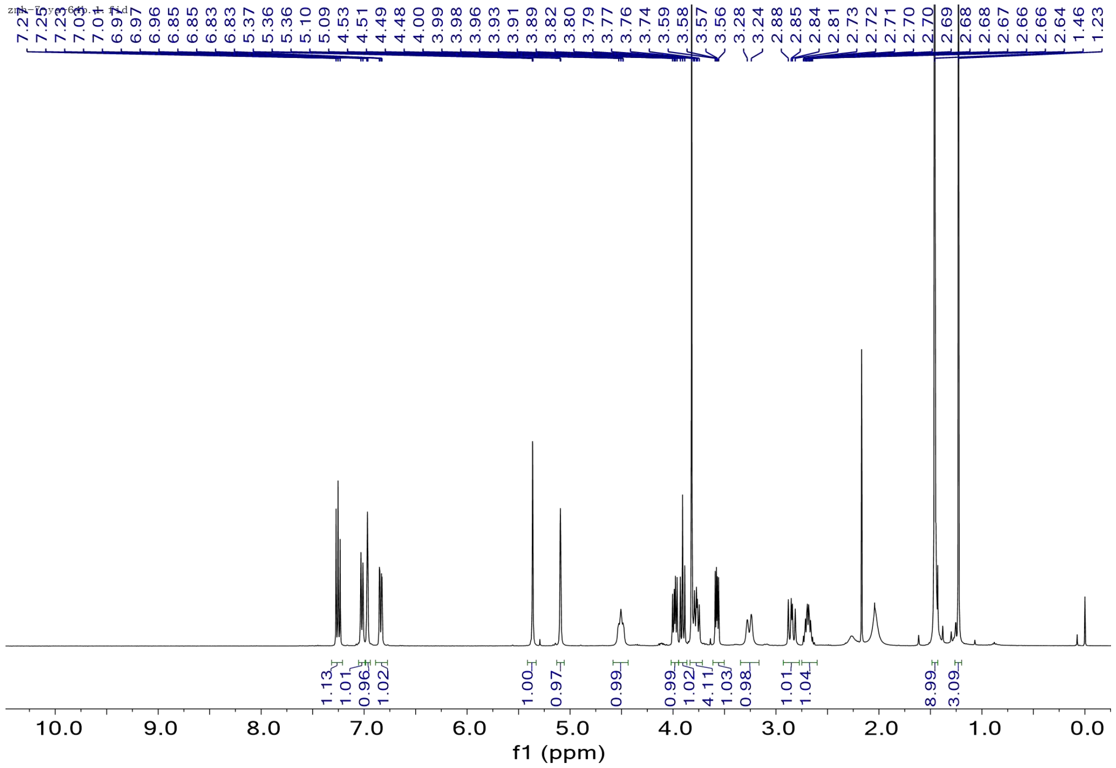
zmh-7-ys-64b.1.fid
7.25 7.23 7.23 7.03 7.01 6.97 6.97 6.96 6.85 6.85 6.83 6.83 5.37 5.36 5.36 5.10 5.09 4.53 4.51 4.49 4.48 4.00 3.99 3.98 3.96 3.93 3.91 3.89 3.82 3.80 3.79 3.77 3.76 3.74 3.59 3.58 3.57 3.56 3.28 3.24 2.88 2.85 2.84 2.81 2.73 2.72 2.71 2.70 2.70 2.69 2.68 2.68 2.67 2.66 2.66 2.64 1.46 1.23
1.13 1.01 0.96 1.02 1.00 0.97 0.99 0.99 1.02 4.11 1.03 0.98 1.01 1.04 8.99 3.09
10.0 9.0 8.0 7.0 6.0 5.0 4.0 3.0 2.0 1.0 0.0
f1 (ppm)

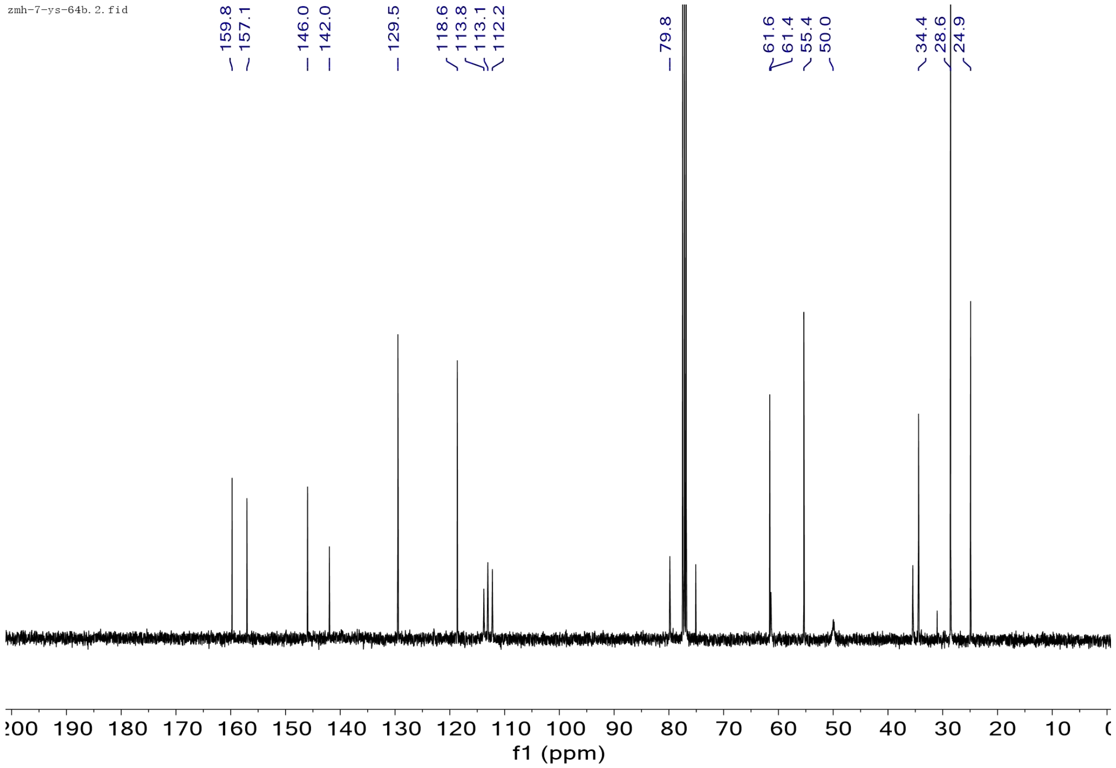
zmh-7-ys-64b.2.fid
159.8 157.1 146.0 142.0 129.5 118.6 113.8 113.1 112.2 79.8 61.6 61.4 55.4 50.0 34.4 28.6 24.9
200 190 180 170 160 150 140 130 120 110 100 90 80 70 60 50 40 30 20 10 0
f1 (ppm)

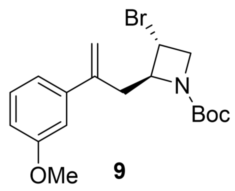

9

zmh--7-ys-br.1.fid

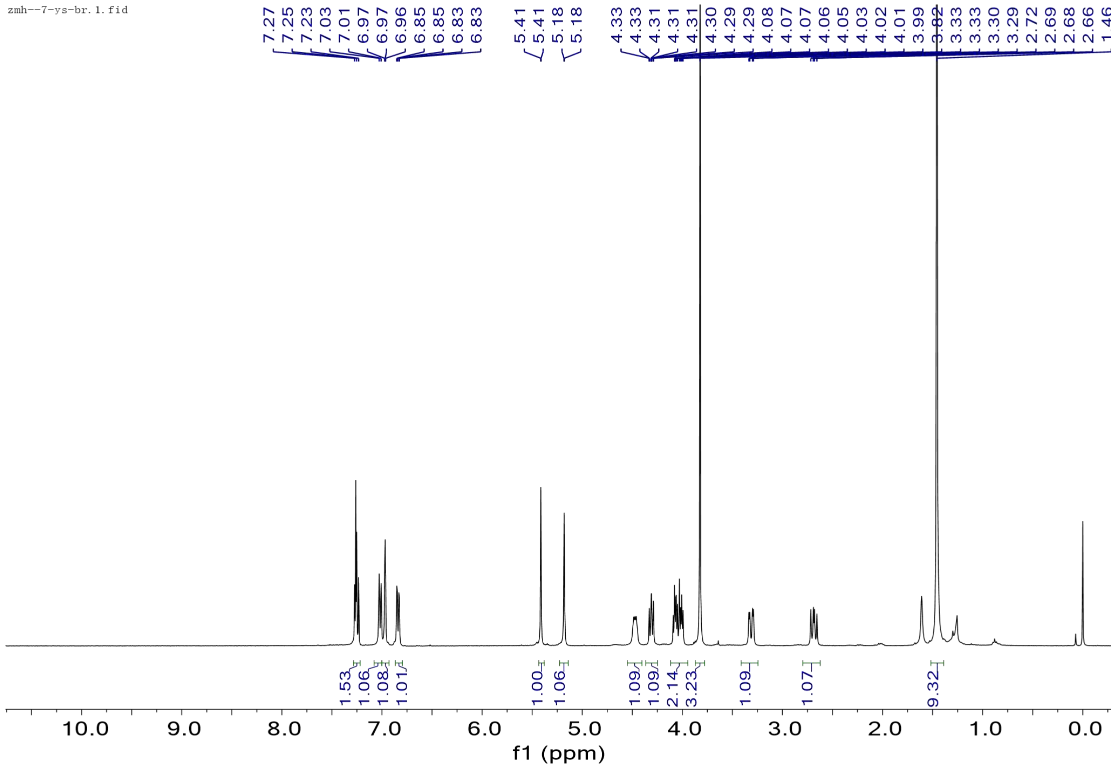

zmh--7-ys-br.2.fid

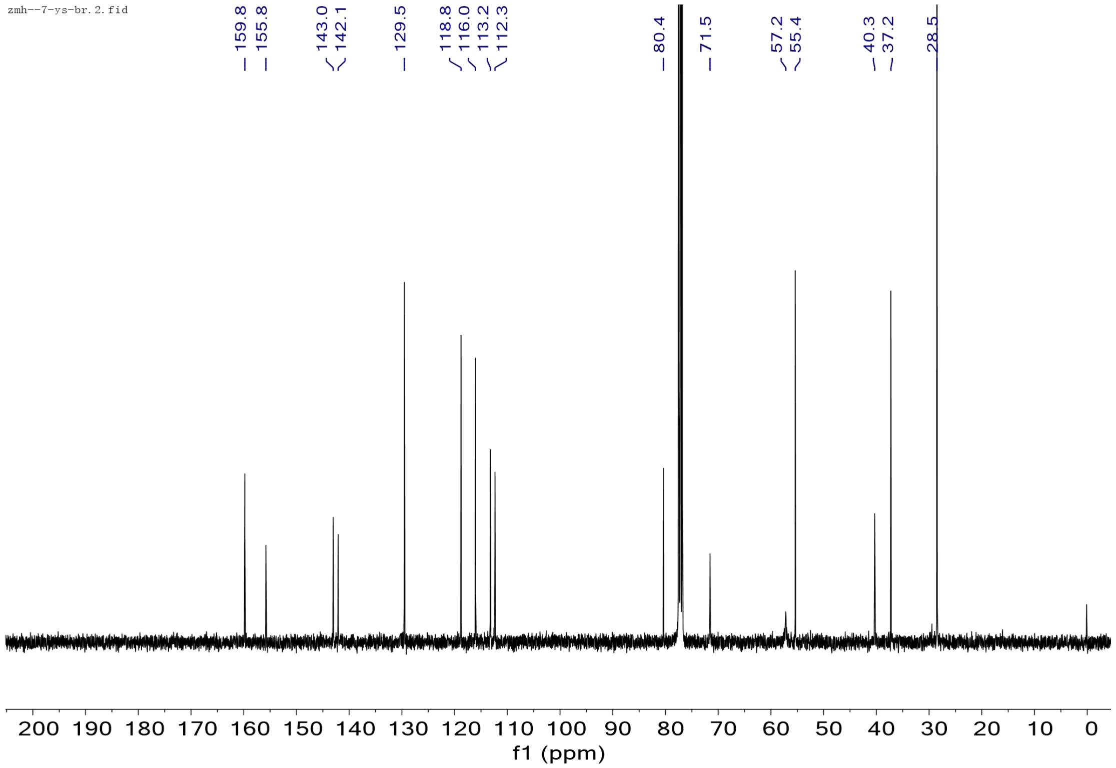

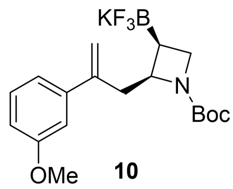

KF3B
N
Boc
OMe

10

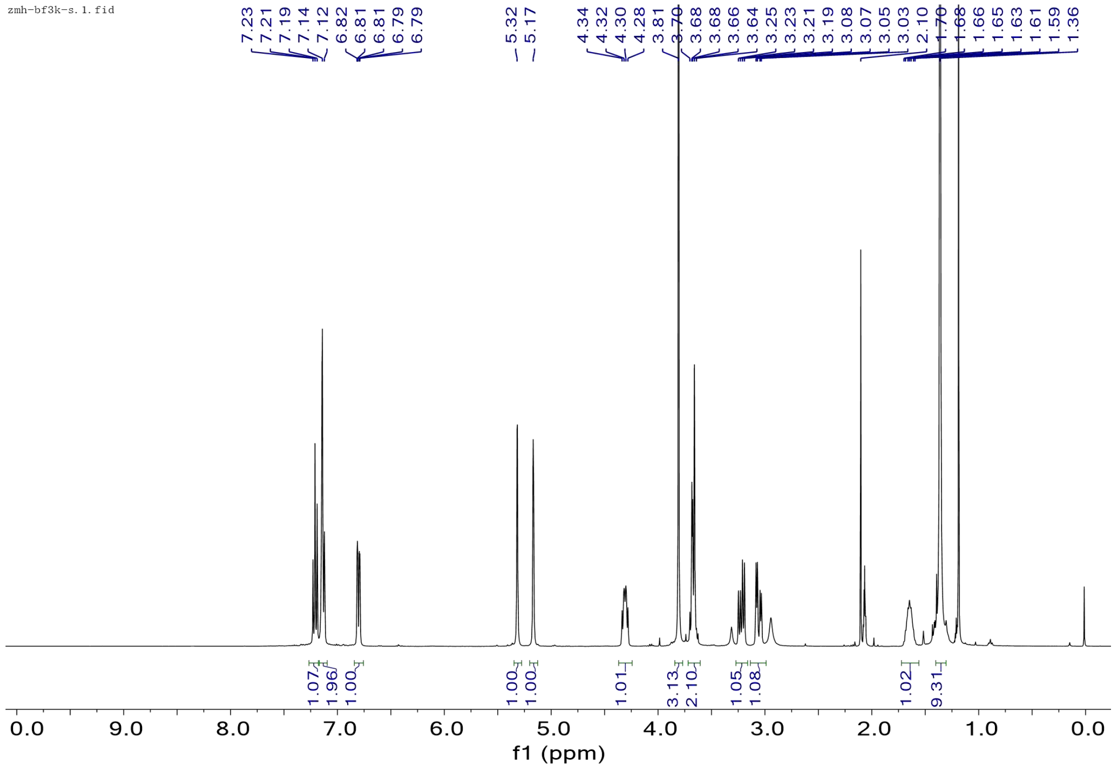

zmh-bf3k-s.1.fid
7.23 7.21 7.19 7.14 7.12 6.82 6.81 6.81 6.79 6.79 5.32 5.17 4.34 4.32 4.30 4.28 3.81 3.70 3.68 3.68 3.66 3.64 3.25 3.23 3.21 3.19 3.08 3.07 3.05 3.03 2.10 1.70 1.68 1.66 1.65 1.63 1.61 1.59 1.36
1.07 1.96 1.00 1.00 1.00 1.01 3.13 2.10 1.05 1.08 1.02 9.31
0.0 9.0 8.0 7.0 6.0 5.0 4.0 3.0 2.0 1.0 0.0
f1 (ppm)

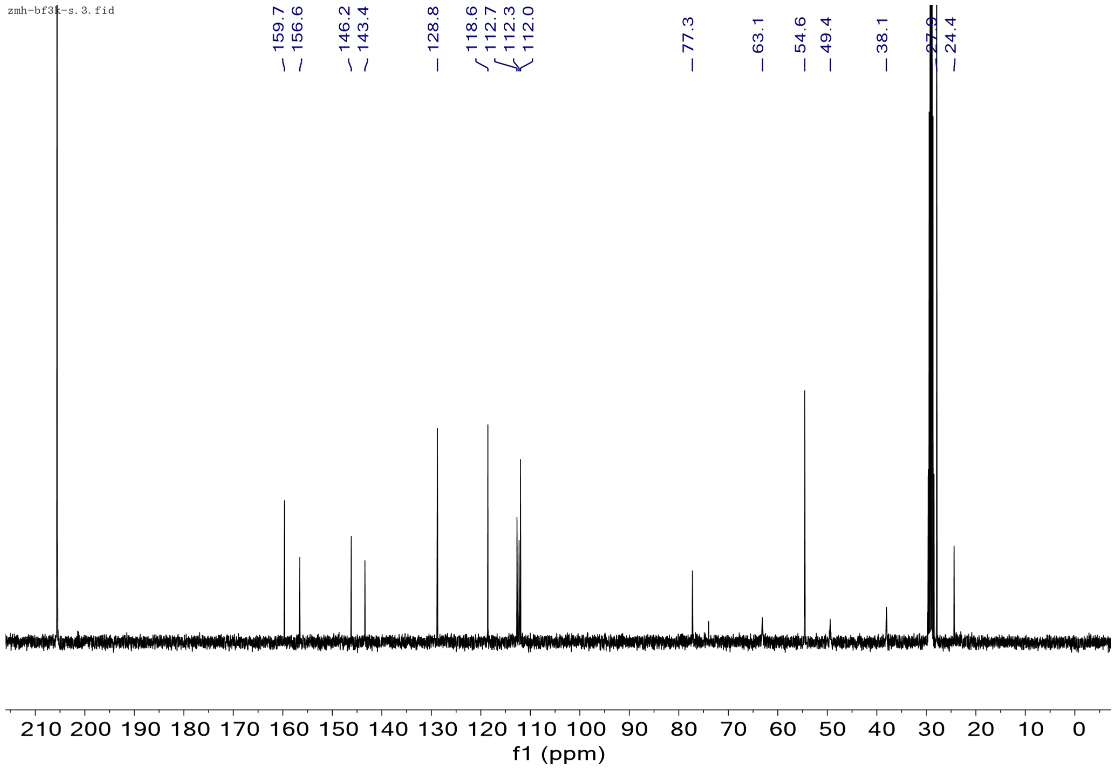

zmh-bf3k-s.3.fid
159.7 156.6 146.2 143.4 128.8 118.6 112.7 112.3 112.0 77.3 63.1 54.6 49.4 38.1 24.4
210 200 190 180 170 160 150 140 130 120 110 100 90 80 70 60 50 40 30 20 10 0
f1 (ppm)

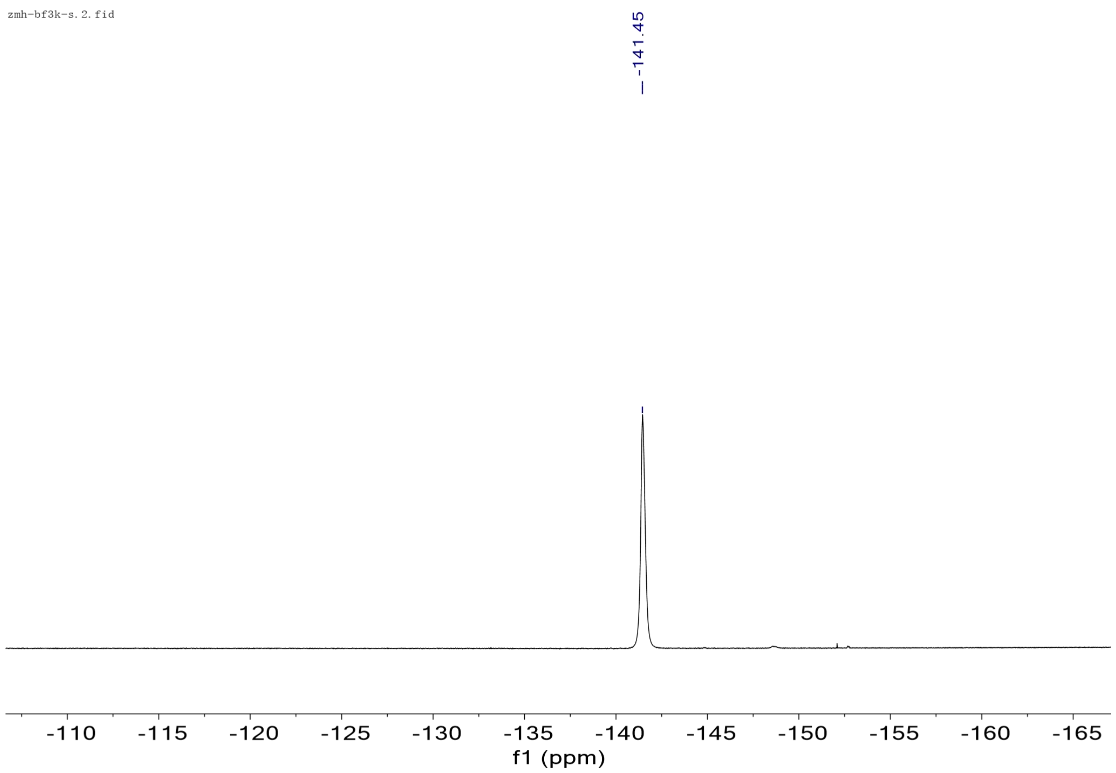
zmh-bf3k-s.2.fid
-141.45
-110
-115
-120
-125
-130
-135
-140
-145
-150
-155
-160
-165
f1 (ppm)

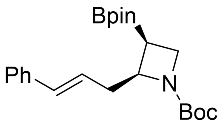
Bpin
Ph
N
Boc

11

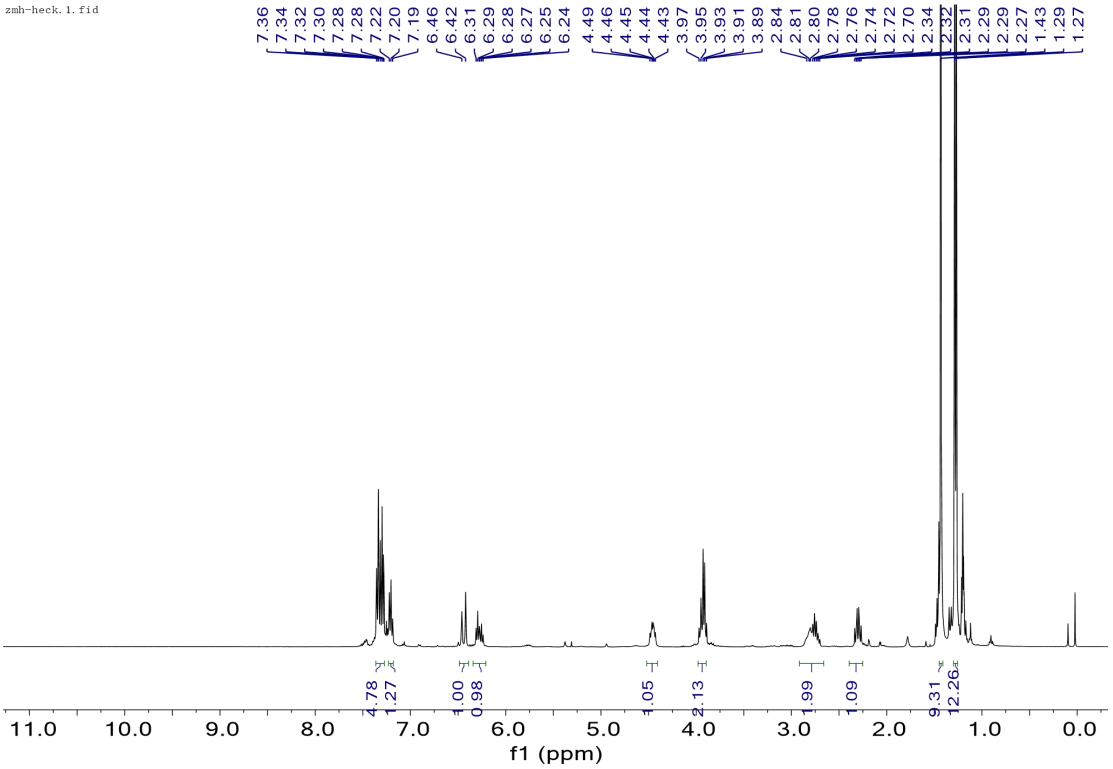
zmh-heck.1.fid
7.36 7.34 7.32 7.30 7.28 7.28 7.22 7.20 7.19 6.46 6.42 6.31 6.29 6.28 6.27 6.25 6.24 4.49 4.46 4.45 4.44 4.43 3.97 3.95 3.93 3.91 3.89 2.84 2.81 2.80 2.78 2.76 2.74 2.72 2.70 2.34 2.32 2.31 2.29 2.27 1.43 1.29 1.27
4.78 1.27 1.00 0.98 1.05 2.13 1.99 1.09 9.31 12.26
11.0 10.0 9.0 8.0 7.0 6.0 5.0 4.0 3.0 2.0 1.0 0.0
f1 (ppm)

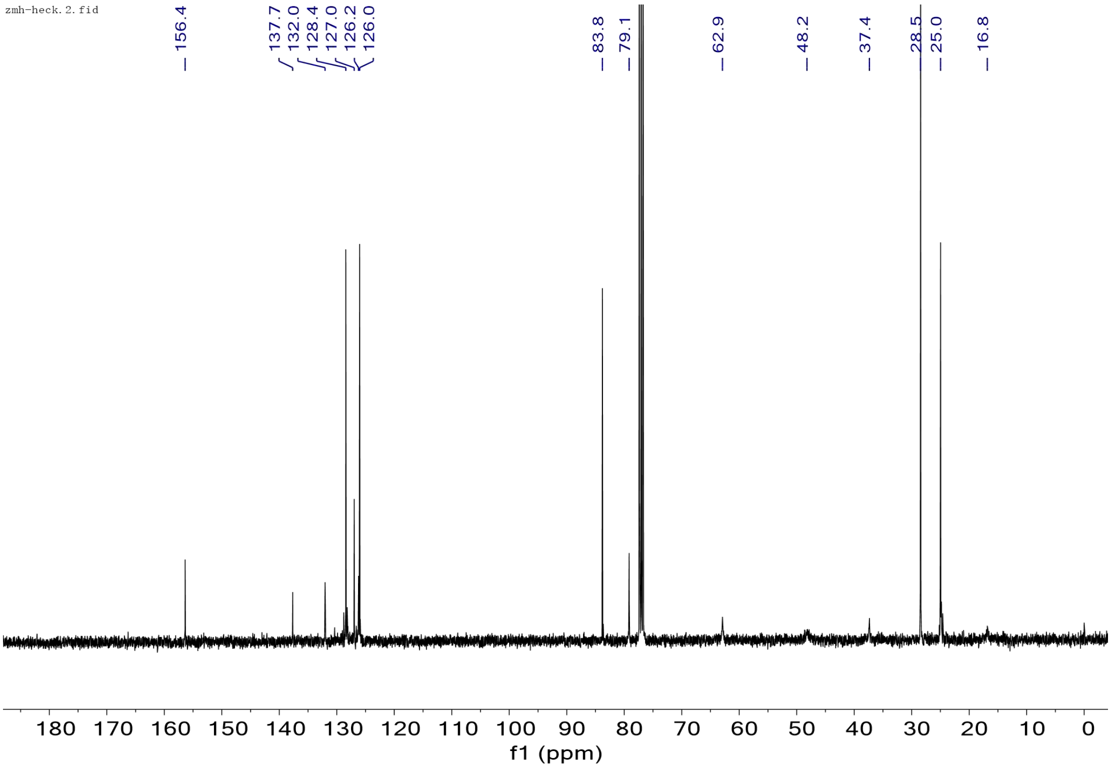
zmh-heck.2.fid
156.4 137.7 132.0 128.4 127.0 126.2 126.0 83.8 79.1 62.9 48.2 37.4 28.5 25.0 16.8
180 170 160 150 140 130 120 110 100 90 80 70 60 50 40 30 20 10 0
f1 (ppm)

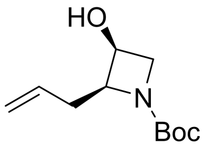
HO
N
Boc

12

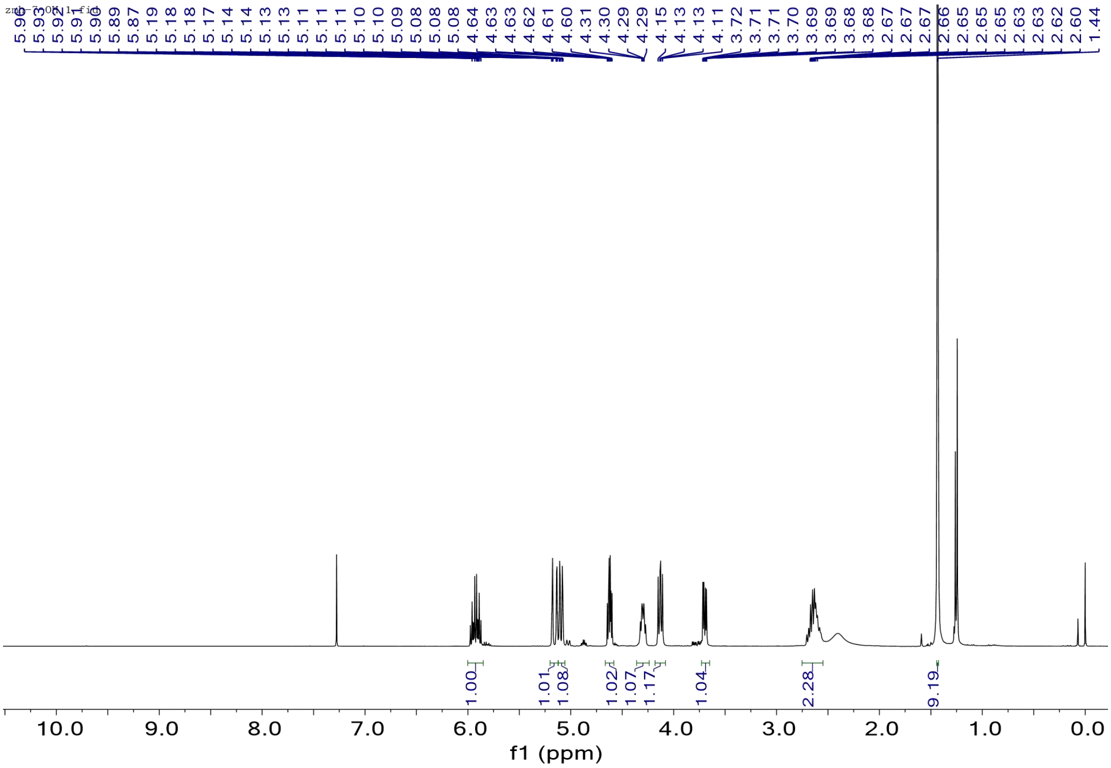
zmh-7-OH.1.fid
5.96 5.93 5.92 5.91 5.90 5.89 5.87 5.19 5.18 5.18 5.17 5.14 5.14 5.13 5.13 5.11 5.11 5.11 5.10 5.10 5.09 5.08 5.08 5.08 4.64 4.63 4.63 4.62 4.61 4.60 4.31 4.30 4.29 4.29 4.15 4.13 4.13 4.11 3.72 3.71 3.71 3.70 3.69 3.69 3.68 3.68 2.67 2.67 2.67 2.66 2.65 2.65 2.65 2.63 2.63 2.62 2.60 1.44
1.00 1.01 1.08 1.02 1.07 1.17 1.04 2.28 9.19
10.0 9.0 8.0 7.0 6.0 5.0 4.0 3.0 2.0 1.0 0.0
f1 (ppm)

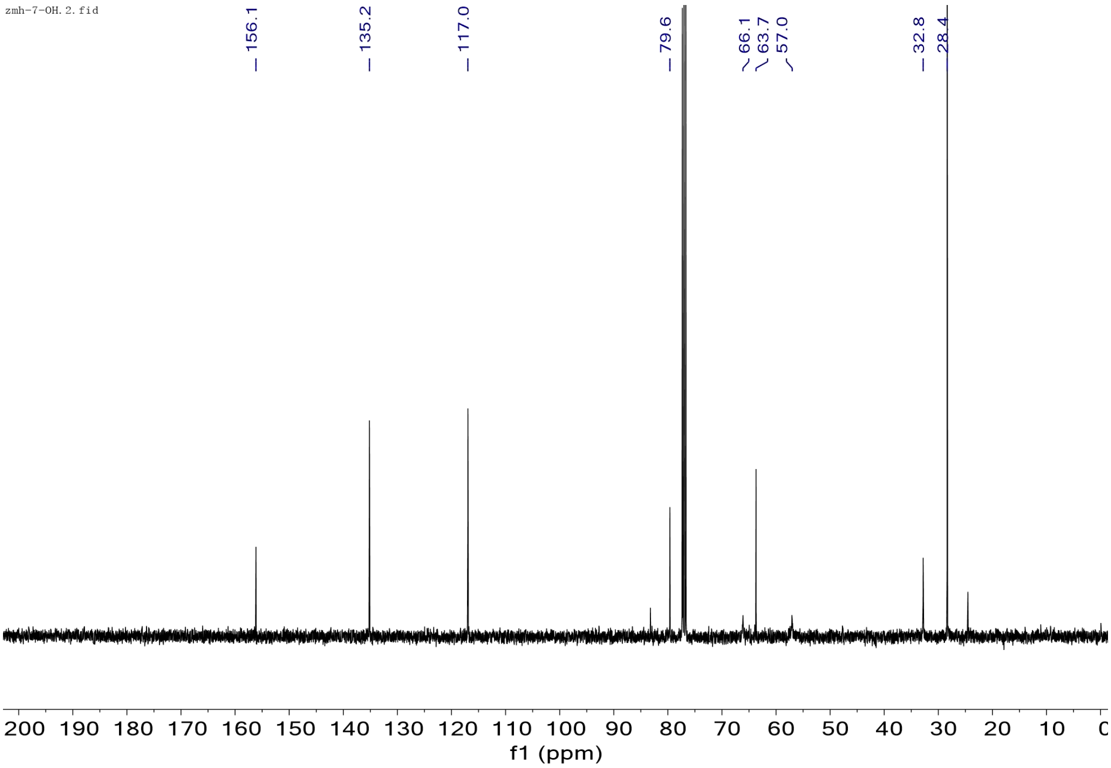
zmh-7-OH.2.fid
156.1
135.2
117.0
79.6
66.1
63.7
57.0
32.8
28.4
200 190 180 170 160 150 140 130 120 110 100 90 80 70 60 50 40 30 20 10 0
f1 (ppm)

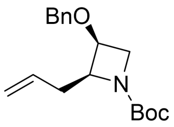
BnO
N
Boc

13

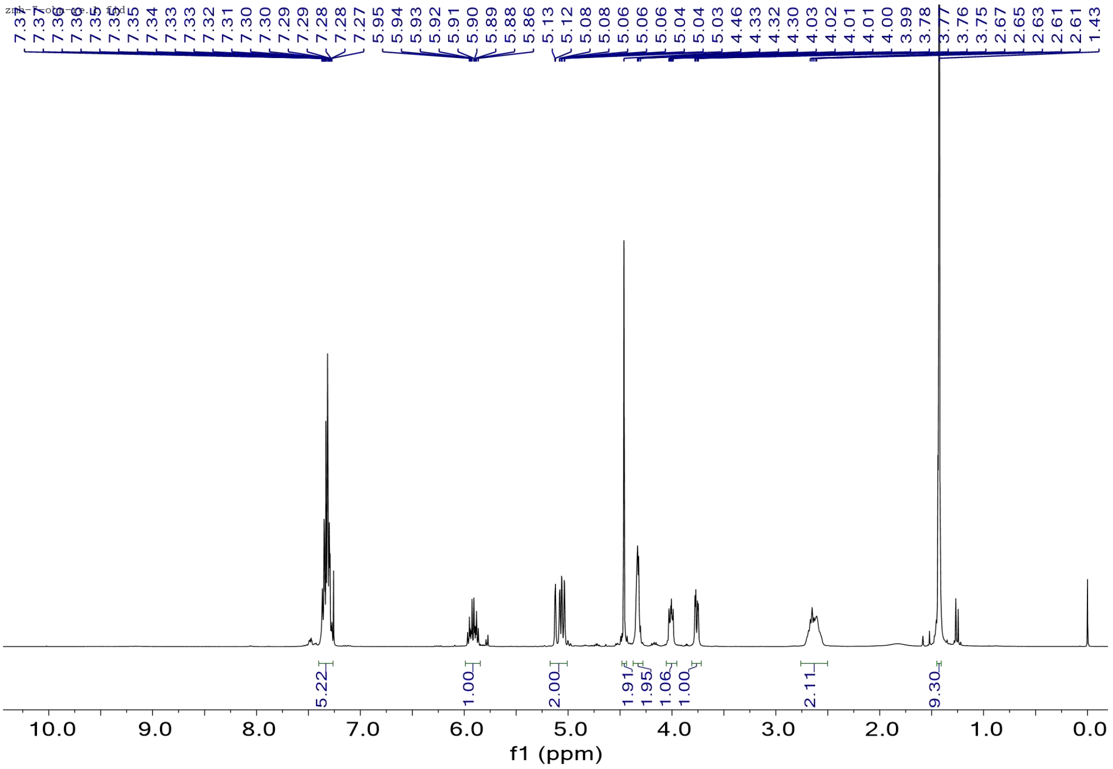
7.37 7.37 7.36 7.36 7.35 7.35 7.35 7.34 7.33 7.33 7.32 7.31 7.30 7.30 7.29 7.29 7.28 7.28 7.27 5.95 5.94 5.93 5.92 5.91 5.90 5.89 5.88 5.86 5.13 5.12 5.08 5.08 5.06 5.06 5.06 5.04 5.04 5.03 4.46 4.33 4.32 4.30 4.03 4.02 4.01 4.01 4.00 3.99 3.78 3.77 3.76 3.75 2.67 2.65 2.63 2.61 2.61 1.43
5.22
1.00
2.00
1.91
1.95
1.06
1.00
2.11
9.30
10.0 9.0 8.0 7.0 6.0 5.0 4.0 3.0 2.0 1.0 0.0
f1 (ppm)

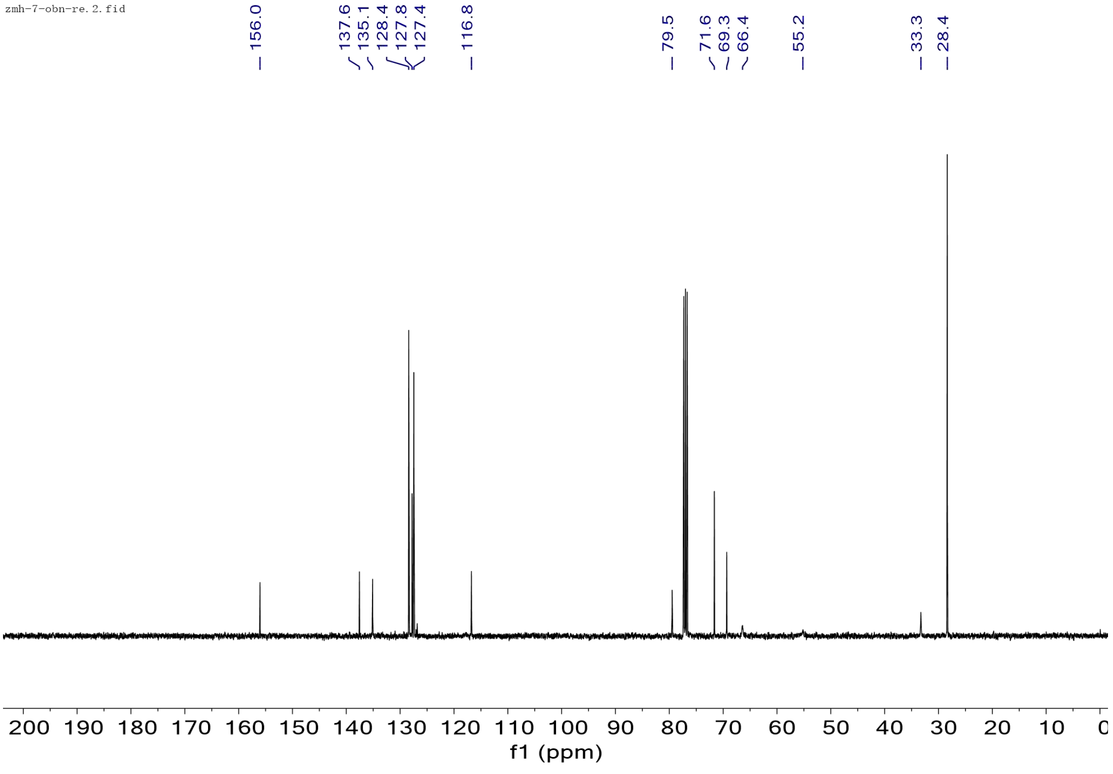
zmh-7-obn-re.2.fid
156.0 137.6 135.1 128.4 127.8 127.4 116.8 79.5 71.6 69.3 66.4 55.2 33.3 28.4
200 190 180 170 160 150 140 130 120 110 100 90 80 70 60 50 40 30 20 10 0
f1 (ppm)

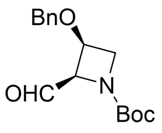

14

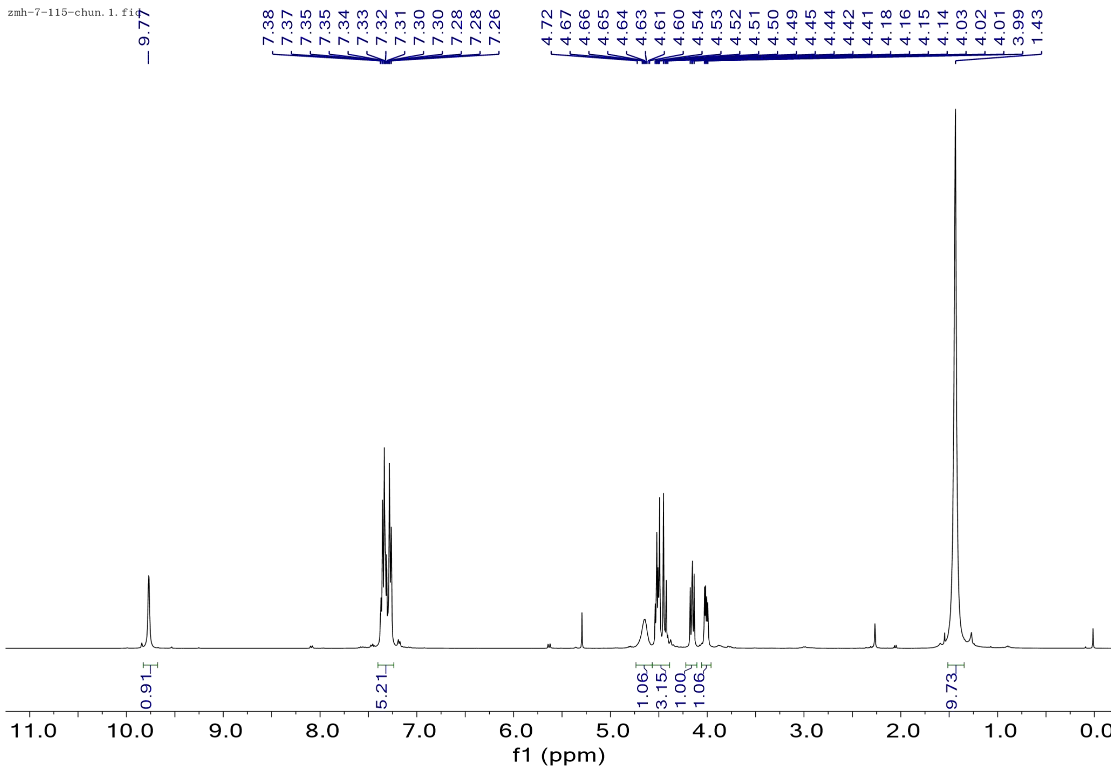

zmh-7-115-chun.1.fid
9.77
7.38 7.37 7.35 7.35 7.34 7.33 7.32 7.31 7.30 7.30 7.28 7.28 7.26
4.72 4.67 4.66 4.65 4.64 4.63 4.61 4.60 4.54 4.53 4.52 4.51 4.50 4.49 4.45 4.44 4.42 4.41 4.18 4.16 4.15 4.14 4.03 4.02 4.01 3.99 1.43
0.91 5.21 1.06 3.15 1.00 1.06 9.73
11.0 10.0 9.0 8.0 7.0 6.0 5.0 4.0 3.0 2.0 1.0 0.0
f1 (ppm)

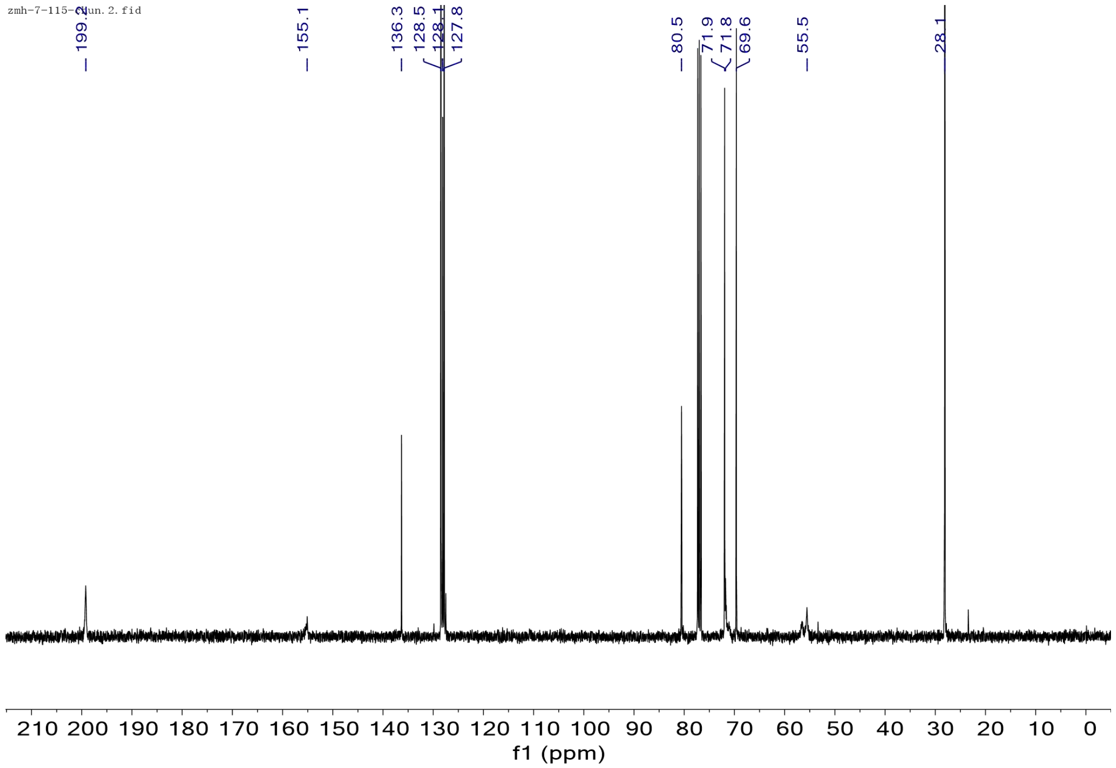

zmh-7-115-chun.2.fid
199.2
155.1
136.3 128.5 128.1 127.8
80.5 71.9 71.8 69.6
55.5
28.1
210 200 190 180 170 160 150 140 130 120 110 100 90 80 70 60 50 40 30 20 10 0
f1 (ppm)

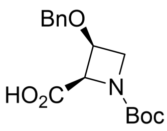
BnO
HO2C
N
Boc

15

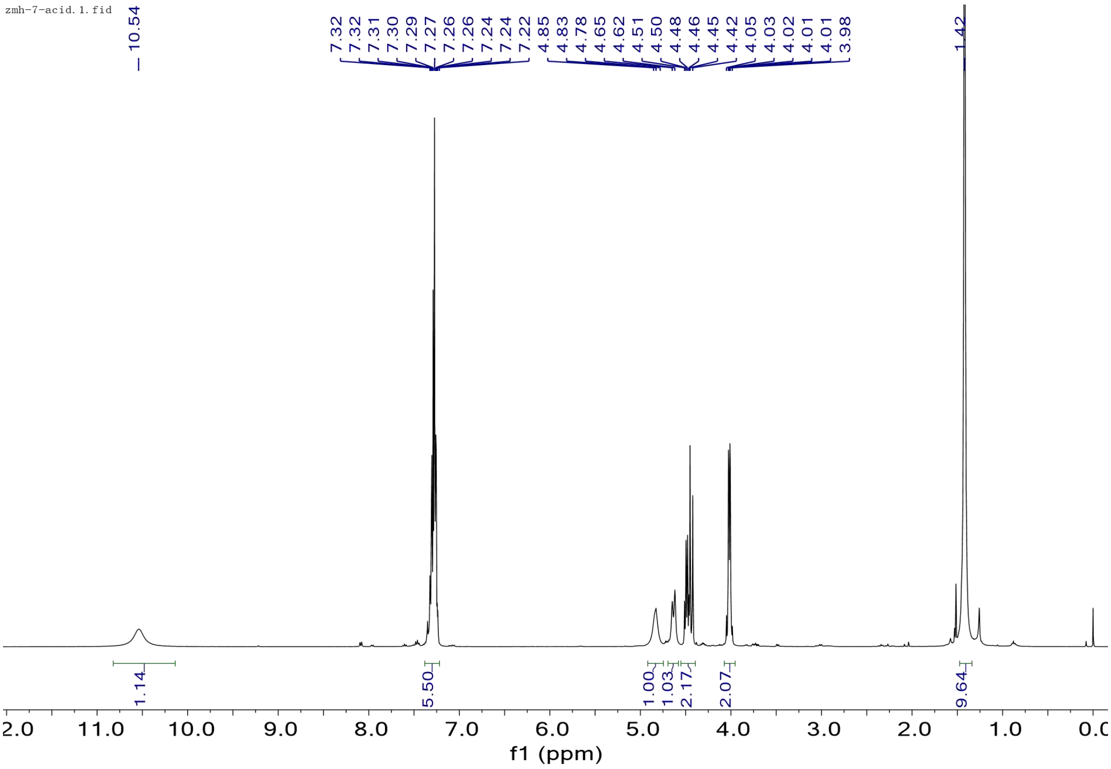
zmh-7-acid.1.fid
10.54
7.32 7.32 7.31 7.30 7.29 7.27 7.26 7.26 7.24 7.24 7.22
4.85 4.83 4.78 4.65 4.62 4.51 4.50 4.48 4.46 4.45 4.42 4.05 4.03 4.02 4.01 4.01 3.98
1.42
1.14 5.50 1.00 1.03 2.17 2.07 9.64
f1 (ppm)

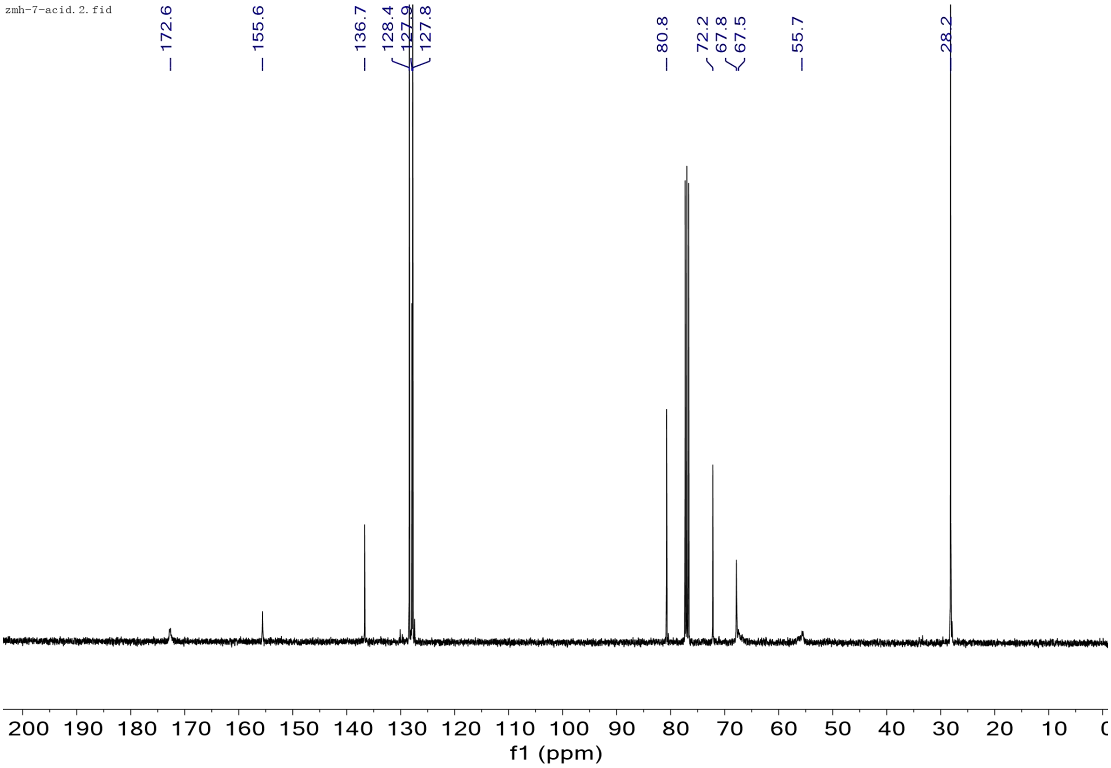
zmh-7-acid.2.fid
172.6
155.6
136.7 128.4 127.9 127.8
80.8
72.2 67.8 67.5
55.7
28.2
f1 (ppm)

===

Acq. Operator : SYSTEM　　Seq. Line : 22
Sample Operator : SYSTEM
Acq. Instrument : HPLC　　Location : P1-A-01
Injection Date : 24/9/2024 1:29:03 am　　Inj : 1
Inj Volume : 2.000 µl
Different Inj Volume from Sample Entry! Actual Inj Volume : 20.000 µl
Acq. Method : C:\Users\Public\Documents\ChemStation\1\Data\SUN\SUN 2024-09-23 19-48-28\OX3-10-20.M
Last changed : 15/8/2022 10:45:05 pm by SYSTEM
Analysis Method : C:\Users\Public\Documents\ChemStation\1\Data\SUN\SUN 2024-09-23 19-48-28\OX3-10-20.M (Sequence Method)
Last changed : 8/5/2025 4:42:25 pm by SYSTEM
(modified after loading)
Additional Info : Peak(s) manually integrated

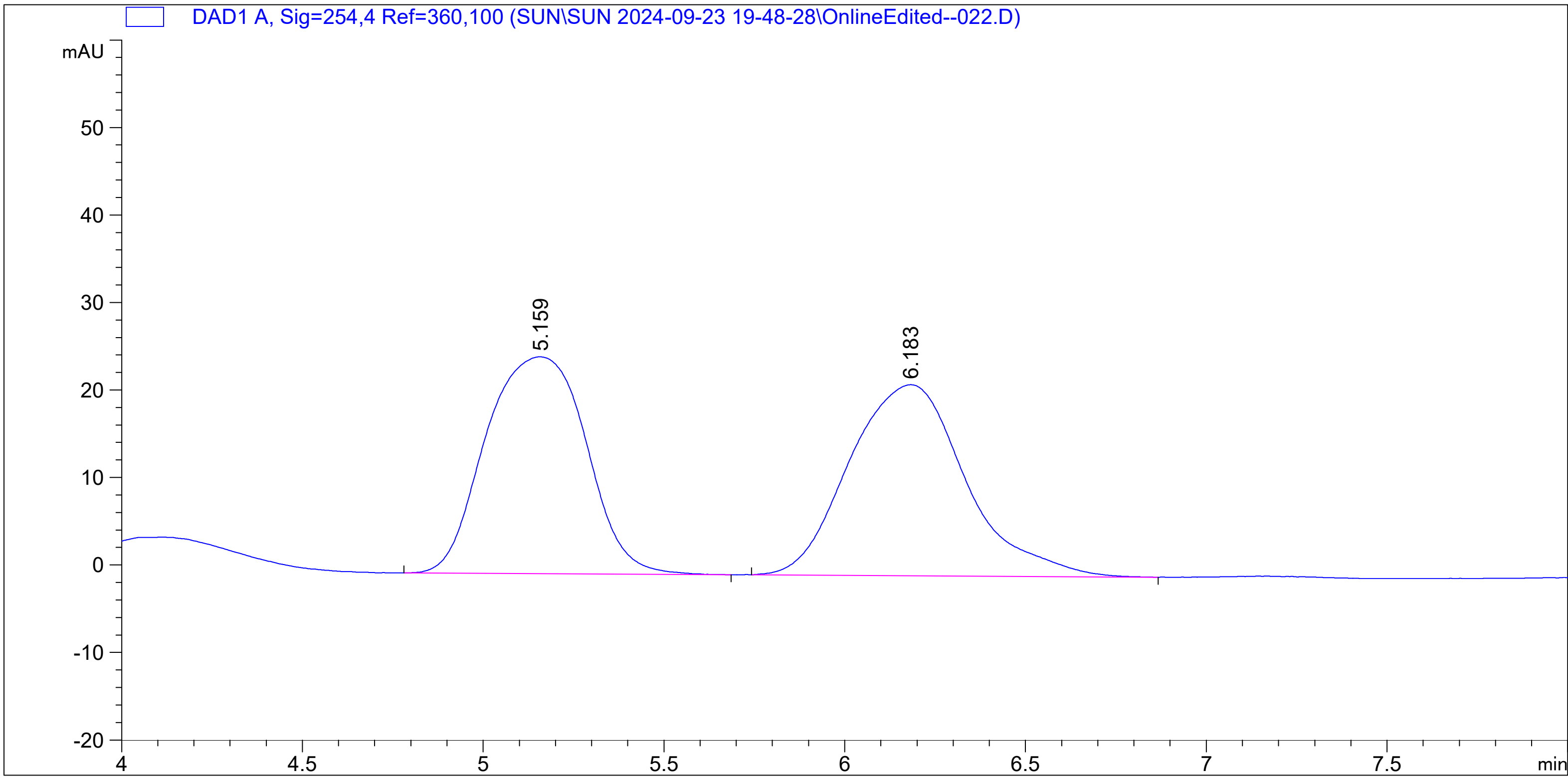

===

Area Percent Report

===

Sorted By : Signal
Multiplier : 1.0000
Dilution : 1.0000
Use Multiplier & Dilution Factor with ISTDs

Signal 1: DAD1 A, Sig=254,4 Ref=360,100

Peak #	RetTime [min]	Type	Width [min]	Area [mAU*s]	Height [mAU]	Area %
1	5.159	BB	0.2386	479.52585	24.79830	49.5336
2	6.183	BB	0.3030	488.55630	21.83618	50.4664

Totals : 968.08215 46.63448

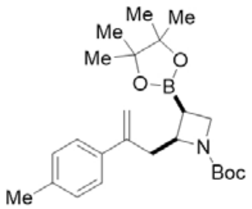

3a
racemic

===

Acq. Operator : SYSTEM Seq. Line : 4
Sample Operator : SYSTEM
Acq. Instrument : HPLC Location : P1-A-01
Injection Date : 26/9/2024 10:42:35 am Inj : 1
Inj Volume : 2.000 µl
Different Inj Volume from Sample Entry! Actual Inj Volume : 20.000 µl
Acq. Method : C:\Users\Public\Documents\ChemStation\1\Data\SUN\SUN 2024-09-26 10-02-23\OX3-10-20.M
Last changed : 15/8/2022 10:45:05 pm by SYSTEM
Analysis Method : C:\Users\Public\Documents\ChemStation\1\Data\SUN\SUN 2024-09-26 10-02-23\OX3-10-20.M (Sequence Method)
Last changed : 8/5/2025 4:45:14 pm by SYSTEM
(modified after loading)
Additional Info : Peak(s) manually integrated

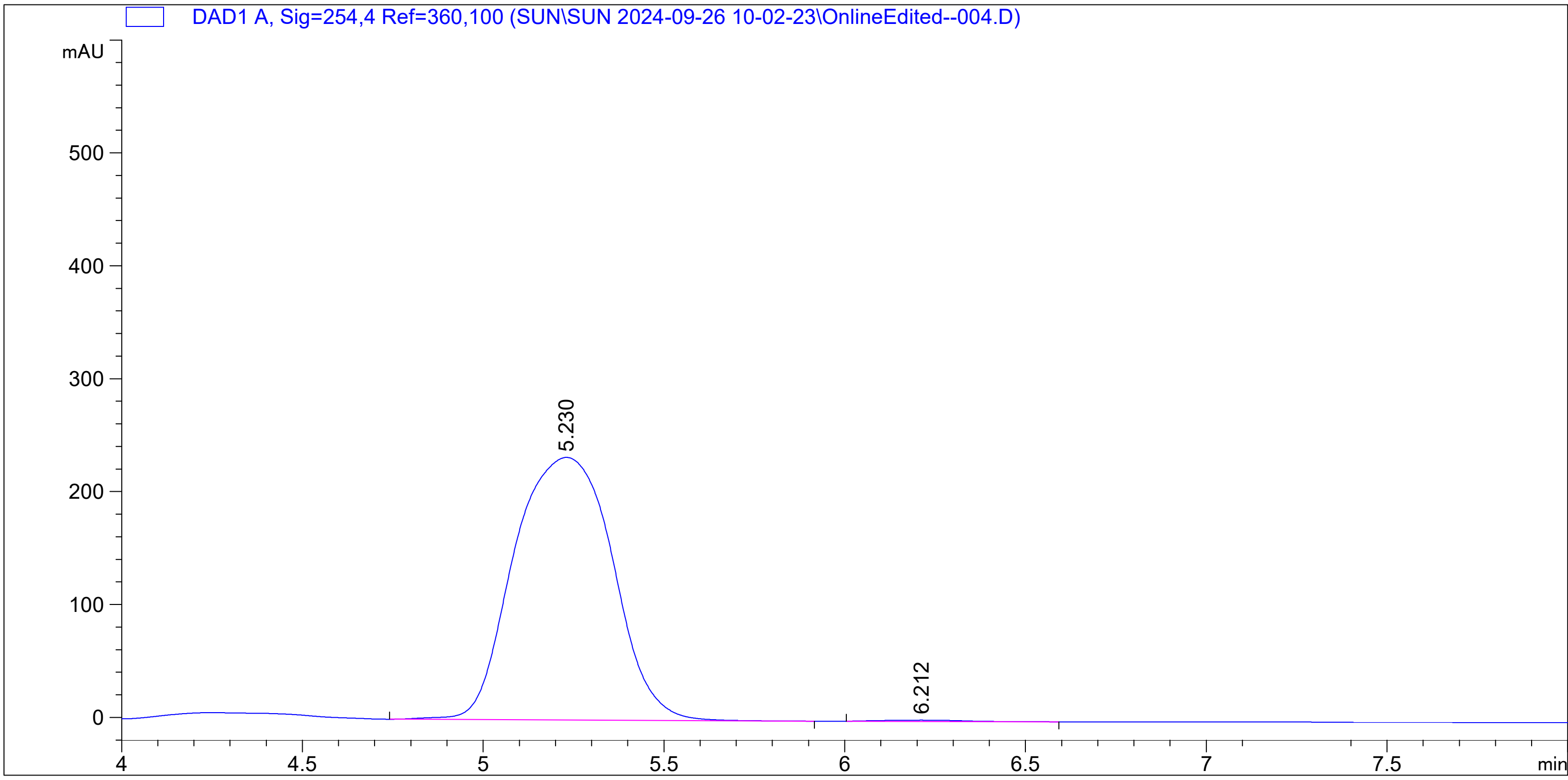

===

Area Percent Report

===

Sorted By : Signal
Multiplier : 1.0000
Dilution : 1.0000
Use Multiplier & Dilution Factor with ISTDs

Signal 1: DAD1 A, Sig=254,4 Ref=360,100

Peak #	RetTime [min]	Type	Width [min]	Area [mAU*s]	Height [mAU]	Area %
1	5.230	BB	0.3156	4419.35889	232.63533	99.6123
2	6.212	BB	0.1829	17.20151	1.11888	0.3877

Totals : 4436.56039 233.75421

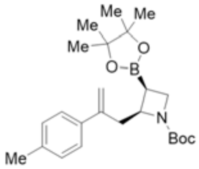

(*S*,*S*)-**3a**
enantioenriched

Data File C:\Users\P...ion\1\Data\SUN\SUN 2025-01-01 13-17-07\008-P2-A5-zmh-7-56a-rac-ic.D
Sample Name: zmh-7-56a-rac-ic

===

Acq. Operator : SYSTEM Seq. Line : 8
Sample Operator : SYSTEM
Acq. Instrument : HPLC Location : P2-A-05
Injection Date : 1/1/2025 3:17:00 pm Inj : 1
Inj Volume : 2.000 µl
Different Inj Volume from Sample Entry! Actual Inj Volume : 10.000 µl
Acq. Method : C:\Users\Public\Documents\ChemStation\1\Data\SUN\SUN 2025-01-01 13-17-07\IC3-10-20.M
Last changed : 15/8/2022 10:26:28 pm by SYSTEM
Analysis Method : C:\Users\Public\Documents\ChemStation\1\Data\SUN\SUN 2025-01-01 13-17-07\IC3-10-20.M (Sequence Method)
Last changed : 8/5/2025 4:52:03 pm by SYSTEM
(modified after loading)
Additional Info : Peak(s) manually integrated

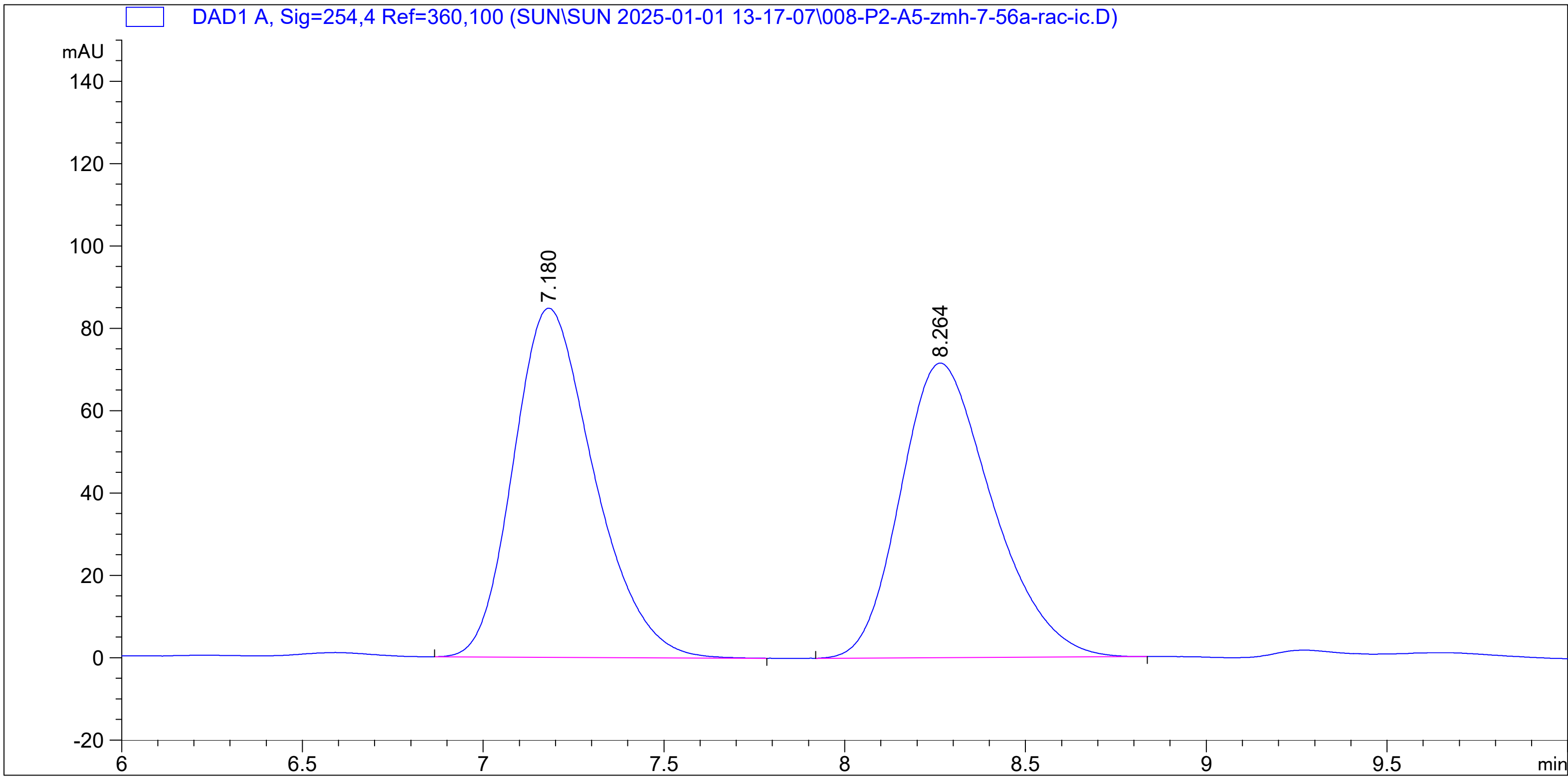

===
Area Percent Report
===

Sorted By : Signal
Multiplier : 1.0000
Dilution : 1.0000
Use Multiplier & Dilution Factor with ISTDs

Signal 1: DAD1 A, Sig=254,4 Ref=360,100

Peak #	RetTime [min]	Type	Width [min]	Area [mAU*s]	Height [mAU]	Area %
1	7.180	BB	0.2371	1316.46838	84.81434	51.0788
2	8.264	BB	0.2661	1260.85779	71.54304	48.9212

Totals : 2577.32617 156.35738

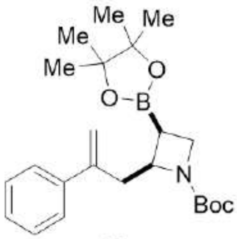

3b
racemic

Data File C:\Users\P...ion\1\Data\SUN\SUN 2025-01-01 13-17-07\094-P1-B4-zmh-7-25f-chiral.D
Sample Name: zmh-7-25f-chiral

===

Acq. Operator : SYSTEM   Seq. Line : 94
Sample Operator : SYSTEM
Acq. Instrument : HPLC   Location : P1-B-04
Injection Date : 2/1/2025 5:19:50 pm   Inj : 1
Inj Volume : 2.000 µl
Different Inj Volume from Sample Entry! Actual Inj Volume : 10.000 µl
Acq. Method : C:\Users\Public\Documents\ChemStation\1\Data\SUN\SUN 2025-01-01 13-17-07\IC3-10-20.M
Last changed : 15/8/2022 10:26:28 pm by SYSTEM
Analysis Method : C:\Users\Public\Documents\ChemStation\1\Data\SUN\SUN 2025-01-01 13-17-07\IC3-10-20.M (Sequence Method)
Last changed : 8/5/2025 4:54:34 pm by SYSTEM
(modified after loading)
Additional Info : Peak(s) manually integrated

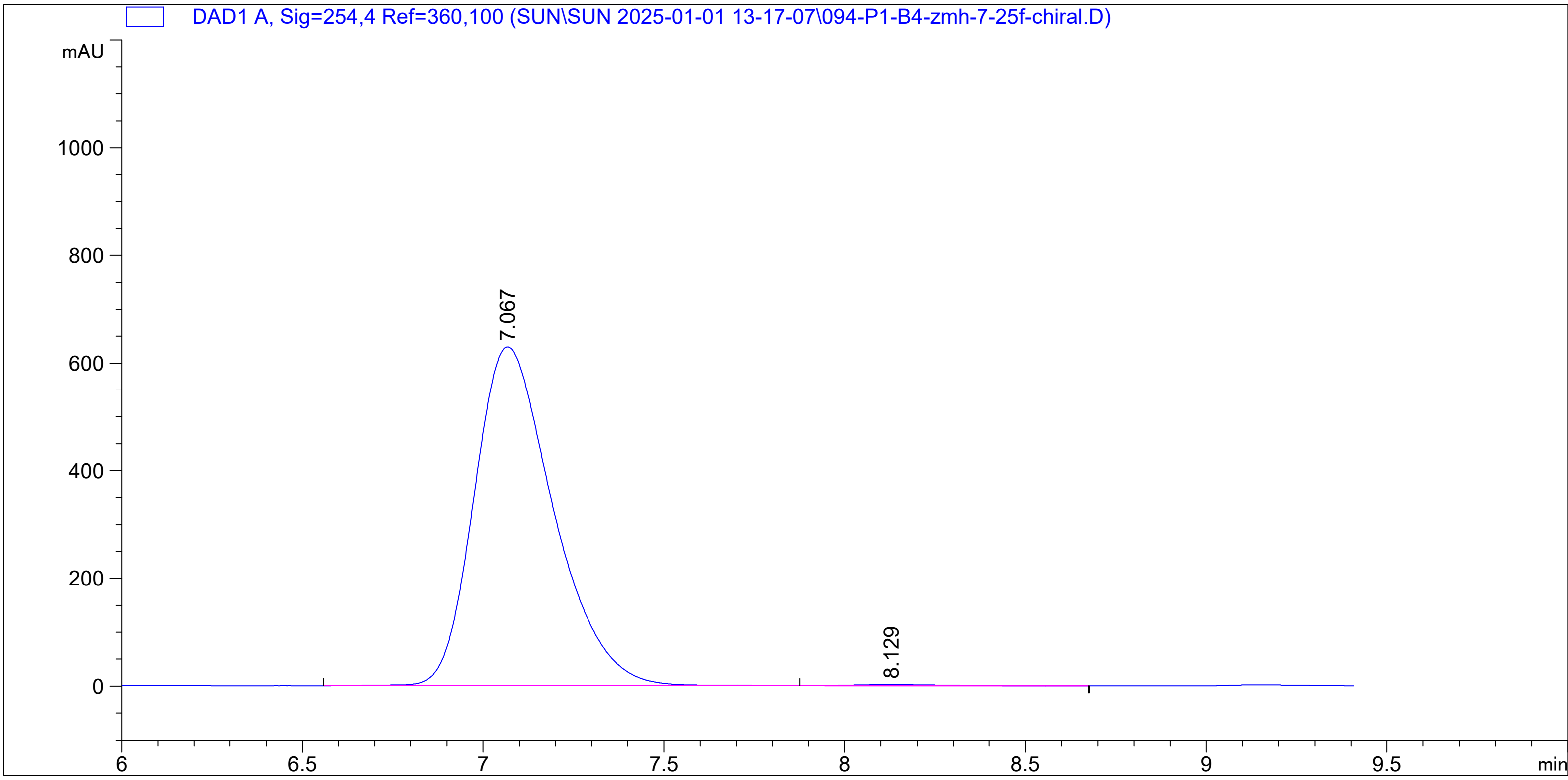

===

Area Percent Report

===

Sorted By : Signal
Multiplier : 1.0000
Dilution : 1.0000
Use Multiplier & Dilution Factor with ISTDs

Signal 1: DAD1 A, Sig=254,4 Ref=360,100

Peak #	RetTime [min]	Type	Width [min]	Area [mAU*s]	Height [mAU]	Area %
1	7.067	BV R	0.2324	9573.48340	629.60498	99.5615
2	8.129	VB E	0.2081	42.16337	2.40402	0.4385

Totals : 9615.64677 632.00900

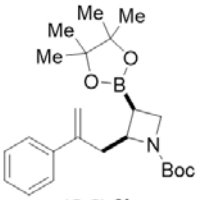

(*S*,*S*)-**3b**
enantioenriched

Data File C:\Users\P...\ChemStation\1\Data\SUN\SUN 2024-10-16 14-58-35\OnlineEdited--006.D
Sample Name: zmh-6-148c-rac-ox

===

Acq. Operator : SYSTEM   Seq. Line : 6
Sample Operator : SYSTEM
Acq. Instrument : HPLC   Location : P1-A-01
Injection Date : 16/10/2024 4:27:58 pm   Inj : 1
Inj Volume : 2.000 µl
Different Inj Volume from Sample Entry! Actual Inj Volume : 20.000 µl
Acq. Method : C:\Users\Public\Documents\ChemStation\1\Data\SUN\SUN 2024-10-16 14-58-35\OX3-10-20.M
Last changed : 15/8/2022 10:45:05 pm by SYSTEM
Analysis Method : C:\Users\Public\Documents\ChemStation\1\Data\SUN\SUN 2024-10-16 14-58-35\OX3-10-20.M (Sequence Method)
Last changed : 8/5/2025 4:57:14 pm by SYSTEM
(modified after loading)
Additional Info : Peak(s) manually integrated

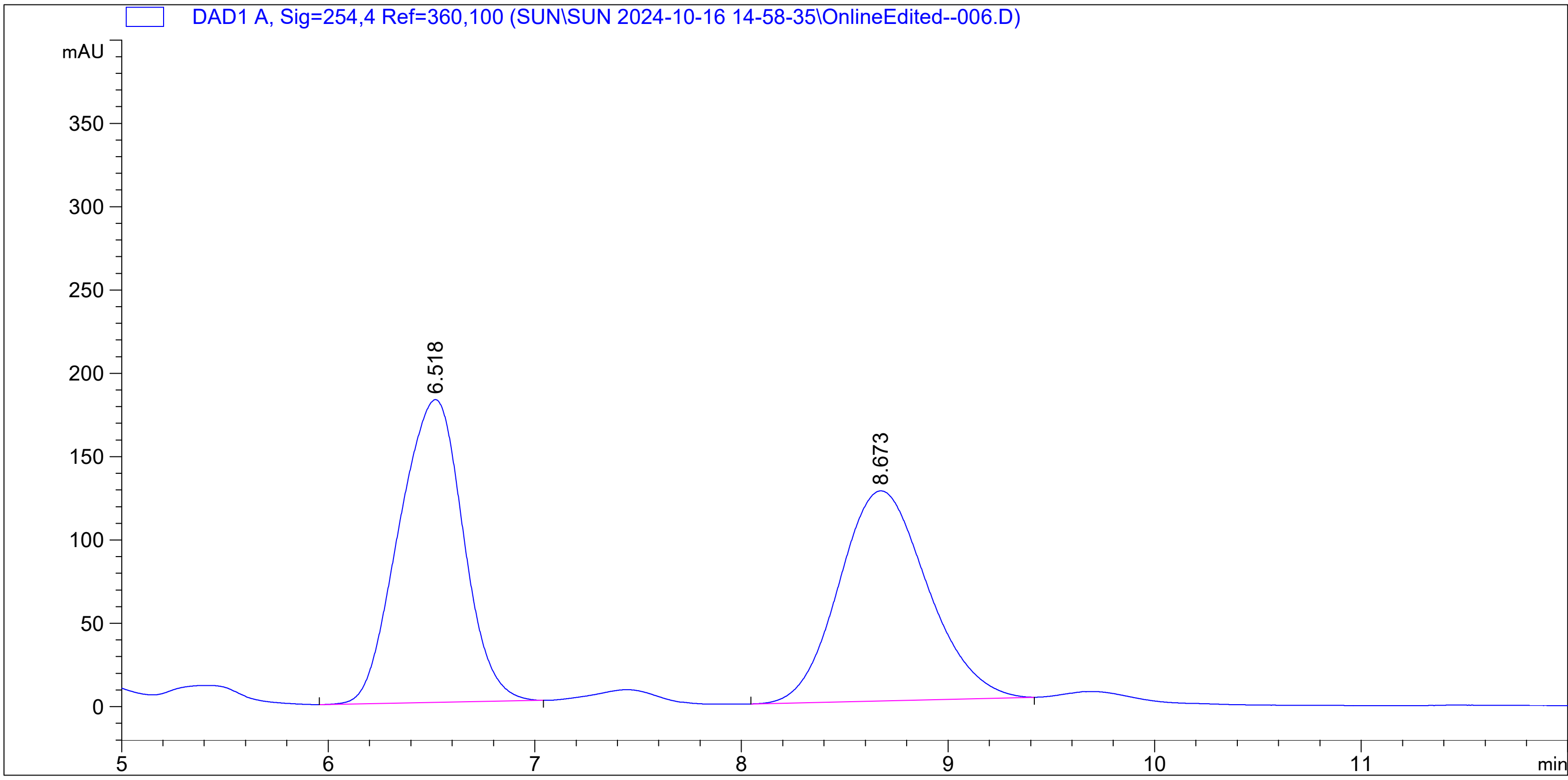

===

Area Percent Report

===

Sorted By : Signal
Multiplier : 1.0000
Dilution : 1.0000
Use Multiplier & Dilution Factor with ISTDs

Signal 1: DAD1 A, Sig=254,4 Ref=360,100

Peak #	RetTime [min]	Type	Width [min]	Area [mAU*s]	Height [mAU]	Area %
1	6.518	BB	0.3103	3921.61768	181.70836	51.1478
2	8.673	BB	0.4548	3745.60938	126.02093	48.8522

Totals : 7667.22705 307.72929

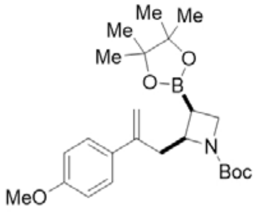

3c
racemic

===

Acq. Operator : SYSTEM Seq. Line : 20
Sample Operator : SYSTEM
Acq. Instrument : HPLC Location : P2-F-01
Injection Date : 17/10/2024 2:41:29 am Inj : 1
Inj Volume : 2.000 µl
Different Inj Volume from Sample Entry! Actual Inj Volume : 20.000 µl
Acq. Method : C:\Users\Public\Documents\ChemStation\1\Data\SUN\SUN 2024-10-16 20-00-36\OX3-10-20.M
Last changed : 15/8/2022 10:45:05 pm by SYSTEM
Analysis Method : C:\Users\Public\Documents\ChemStation\1\Data\SUN\SUN 2024-10-16 20-00-36\OX3-10-20.M (Sequence Method)
Last changed : 8/5/2025 5:00:53 pm by SYSTEM
(modified after loading)
Additional Info : Peak(s) manually integrated

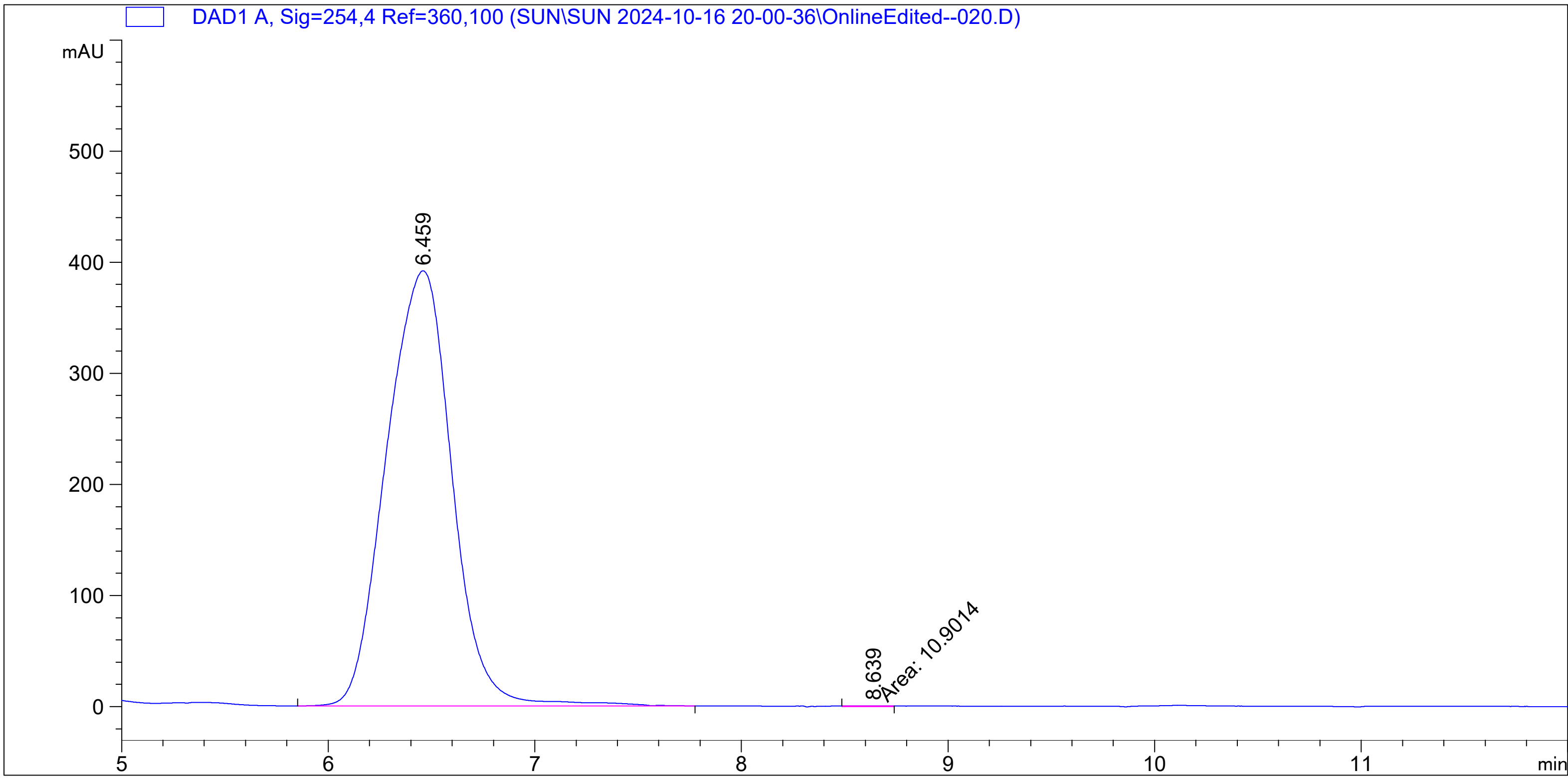

===
Area Percent Report
===

Sorted By : Signal
Multiplier : 1.0000
Dilution : 1.0000
Use Multiplier & Dilution Factor with ISTDs

Signal 1: DAD1 A, Sig=254,4 Ref=360,100

Peak #	RetTime [min]	Type	Width [min]	Area [mAU*s]	Height [mAU]	Area %
1	6.459	BV R	0.3572	8630.69336	391.56131	99.8739
2	8.639	MM	0.2359	10.90135	7.70199e-1	0.1261

Totals : 8641.59471 392.33151

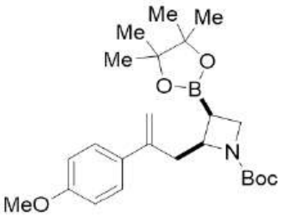

(*S*,*S*)-**3c**
enantioenriched

Data File C:\Users\P...on\1\Data\SUN\SUN 2024-10-28 11-11-06\028-P1-F1-zmh-6-166f-rac-id.D
Sample Name: zmh-6-166f-rac-id

===

Acq. Operator : SYSTEM   Seq. Line : 28
Sample Operator : SYSTEM
Acq. Instrument : HPLC   Location : P1-F-01
Injection Date : 28/10/2024 6:57:04 pm   Inj : 1
Inj Volume : 2.000 µl
Different Inj Volume from Sample Entry! Actual Inj Volume : 20.000 µl
Acq. Method : C:\Users\Public\Documents\ChemStation\1\Data\SUN\SUN 2024-10-28 11-11-06\ID3-10-20.M
Last changed : 2/8/2023 12:18:49 pm by SYSTEM
Analysis Method : C:\Users\Public\Documents\ChemStation\1\Data\SUN\SUN 2024-10-28 11-11-06\ID3-10-20.M (Sequence Method)
Last changed : 8/5/2025 5:06:50 pm by SYSTEM
(modified after loading)
Additional Info : Peak(s) manually integrated

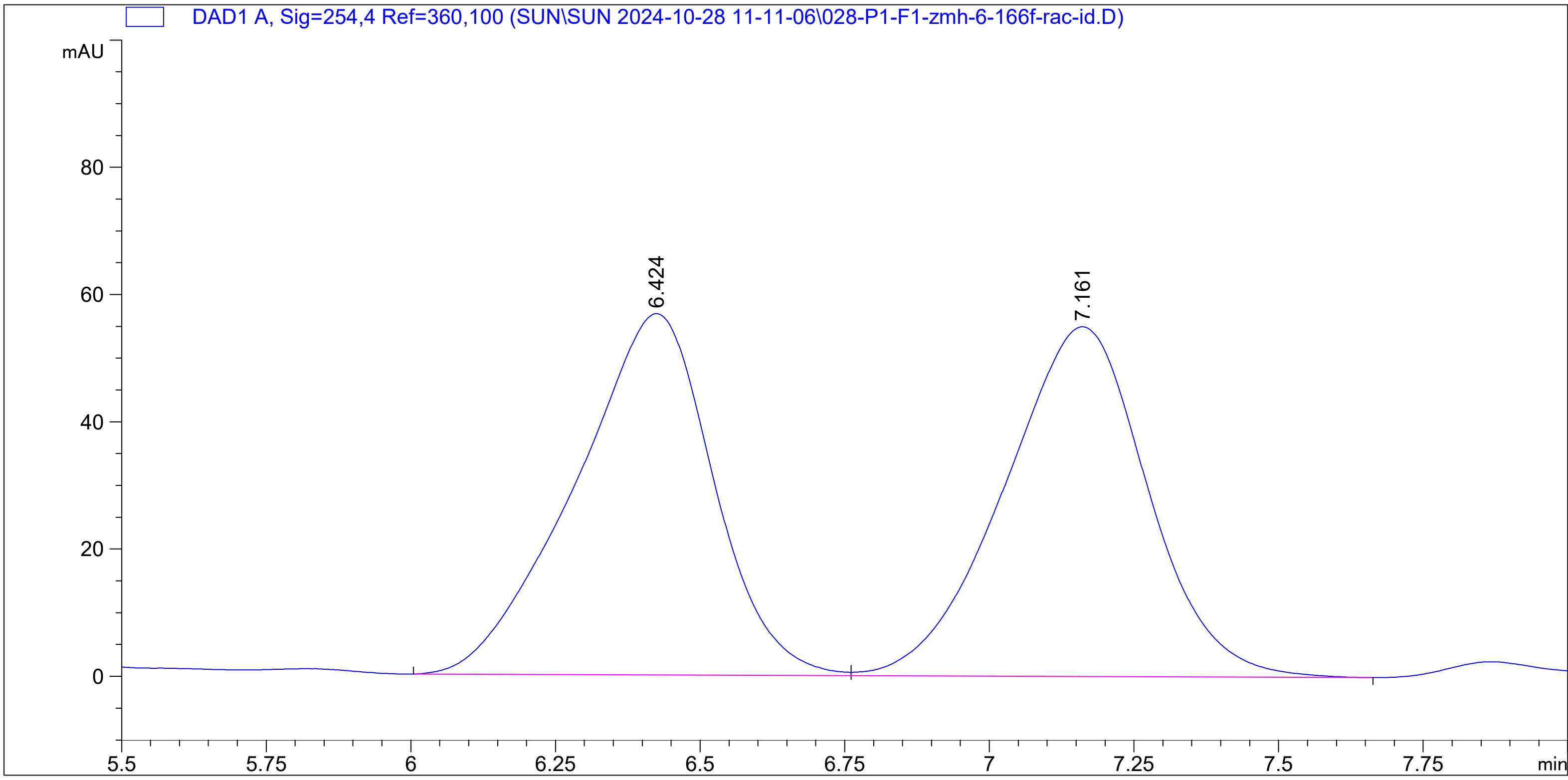

===

Area Percent Report

===

Sorted By : Signal
Multiplier : 1.0000
Dilution : 1.0000
Use Multiplier & Dilution Factor with ISTDs

Signal 1: DAD1 A, Sig=254,4 Ref=360,100

Peak #	RetTime [min]	Type	Width [min]	Area [mAU*s]	Height [mAU]	Area %
1	6.424	BV	0.2407	942.68469	56.78905	49.7955
2	7.161	VB	0.2572	950.42822	54.97268	50.2045

Totals : 1893.11292 111.76173

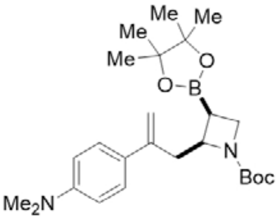

3d
racemic

===

Acq. Operator : SYSTEM Seq. Line : 29
Sample Operator : SYSTEM
Acq. Instrument : HPLC Location : P2-D-01
Injection Date : 28/10/2024 7:18:29 pm Inj : 1
Inj Volume : 2.000 µl
Different Inj Volume from Sample Entry! Actual Inj Volume : 20.000 µl
Acq. Method : C:\Users\Public\Documents\ChemStation\1\Data\SUN\SUN 2024-10-28 11-11-06\ID3-10-20.M
Last changed : 2/8/2023 12:18:49 pm by SYSTEM
Analysis Method : C:\Users\Public\Documents\ChemStation\1\Data\SUN\SUN 2024-10-28 11-11-06\ID3-10-20.M (Sequence Method)
Last changed : 8/5/2025 5:09:31 pm by SYSTEM
(modified after loading)
Additional Info : Peak(s) manually integrated

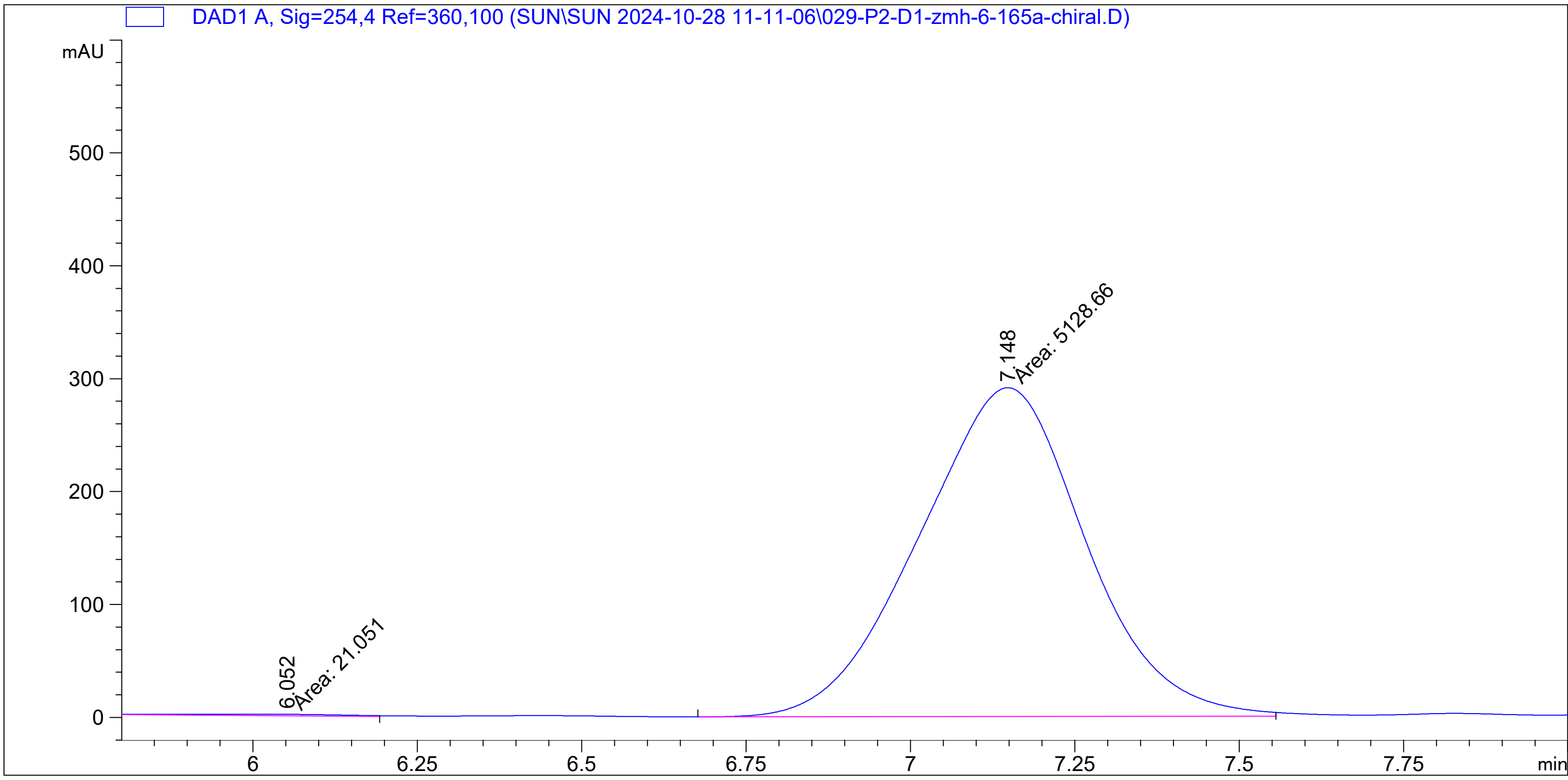

===

Area Percent Report

===

Sorted By : Signal
Multiplier : 1.0000
Dilution : 1.0000
Use Multiplier & Dilution Factor with ISTDs

Signal 1: DAD1 A, Sig=254,4 Ref=360,100

Peak #	RetTime [min]	Type	Width [min]	Area [mAU*s]	Height [mAU]	Area %
1	6.052	MM	0.2700	21.05096	1.29954	0.4088
2	7.148	MF	0.2936	5128.66162	291.13956	99.5912

Totals : 5149.71258 292.43910

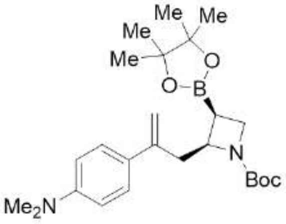

(*S*,*S*)-**3d**
enantioenriched

Data File C:\Users\P...ation\1\Data\SUN\SUN 2025-06-03 15-37-52\028-P2-A1-zmh-ome-rac-oz.D
Sample Name: zmh-ome-rac-oz

===

Acq. Operator : SYSTEM   Seq. Line : 28
Sample Operator : SYSTEM
Acq. Instrument : HPLC   Location : P2-A-01
Injection Date : 3/6/2025 11:09:50 pm   Inj : 1
Inj Volume : 2.000 µl
Different Inj Volume from Sample Entry! Actual Inj Volume : 10.000 µl
Acq. Method : C:\Users\Public\Documents\ChemStation\1\Data\SUN\SUN 2025-06-03 15-37-52\OZ3-10-20.M
Last changed : 15/8/2022 10:41:10 pm by SYSTEM
Analysis Method : C:\Users\Public\Documents\ChemStation\1\Data\SUN\SUN 2025-06-03 15-37-52\OZ3-10-20.M (Sequence Method)
Last changed : 5/6/2025 9:13:28 pm by SYSTEM
(modified after loading)
Additional Info : Peak(s) manually integrated

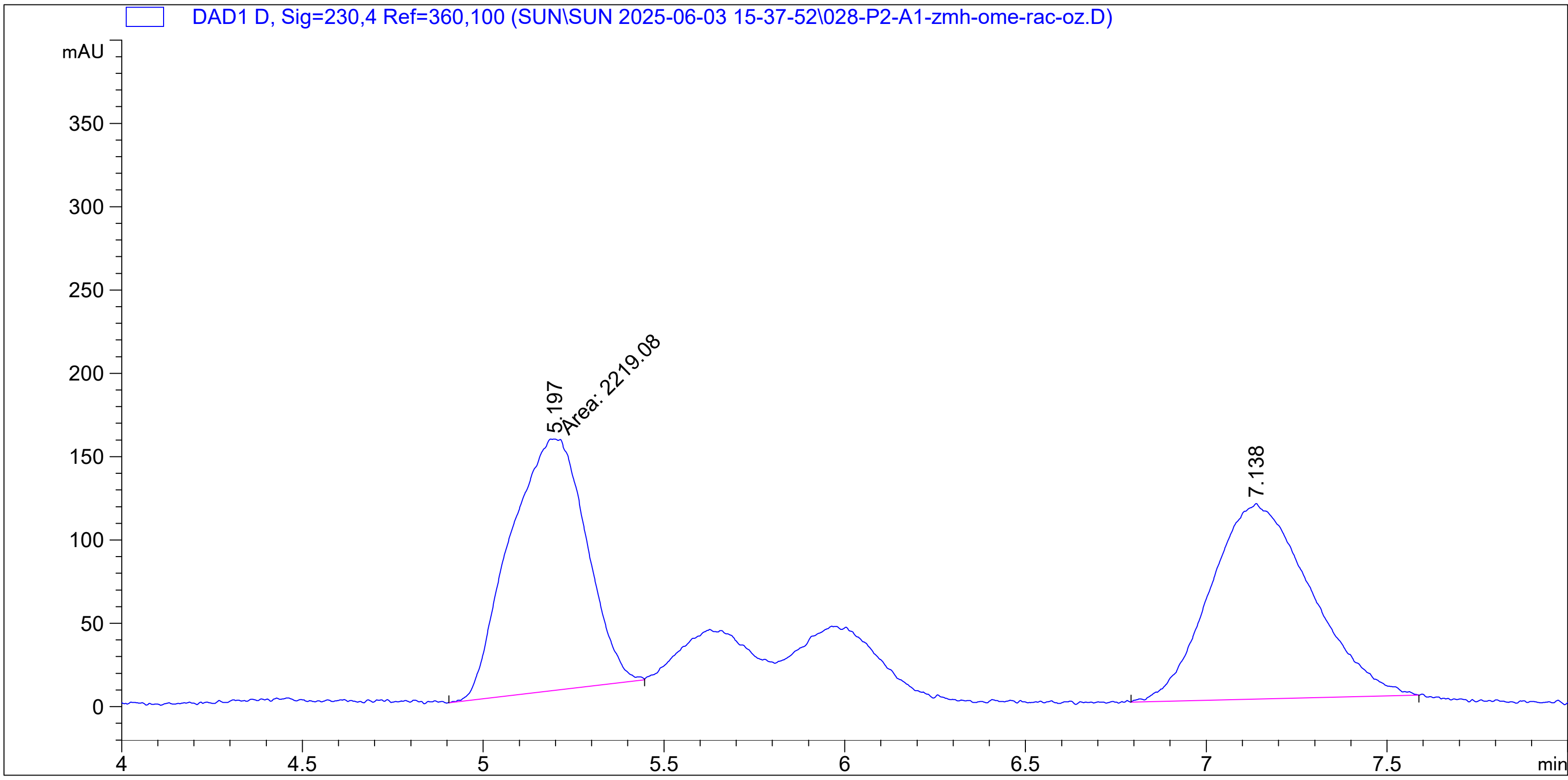

===

Area Percent Report

===

Sorted By : Signal
Multiplier : 1.0000
Dilution : 1.0000
Use Multiplier & Dilution Factor with ISTDs

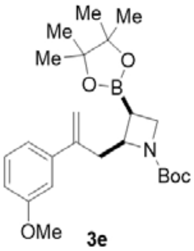

3e
racemic

Signal 1: DAD1 D, Sig=230,4 Ref=360,100

Peak #	RetTime [min]	Type	Width [min]	Area [mAU*s]	Height [mAU]	Area %
1	5.197	MM	0.2452	2219.07715	150.81708	49.3911
2	7.138	VV R	0.2296	2273.79492	117.30549	50.6089

Totals : 4492.87207 268.12257

Data File C:\Users\P...ation\1\Data\SUN\SUN 2025-06-04 19-31-15\005-P1-B1-zmh-ome-chiral.D
Sample Name: zmh-ome-chiral

===

Acq. Operator : SYSTEM Seq. Line : 5
Sample Operator : SYSTEM
Acq. Instrument : HPLC Location : P1-B-01
Injection Date : 4/6/2025 8:34:25 pm Inj : 1
Inj Volume : 2.000 µl
Different Inj Volume from Sample Entry! Actual Inj Volume : 10.000 µl
Acq. Method : C:\Users\Public\Documents\ChemStation\1\Data\SUN\SUN 2025-06-04 19-31-15\OZ3-10-20.M
Last changed : 15/8/2022 10:41:10 pm by SYSTEM
Analysis Method : C:\Users\Public\Documents\ChemStation\1\Data\SUN\SUN 2025-06-04 19-31-15\OZ3-10-20.M (Sequence Method)
Last changed : 5/6/2025 9:17:31 pm by SYSTEM
(modified after loading)
Additional Info : Peak(s) manually integrated

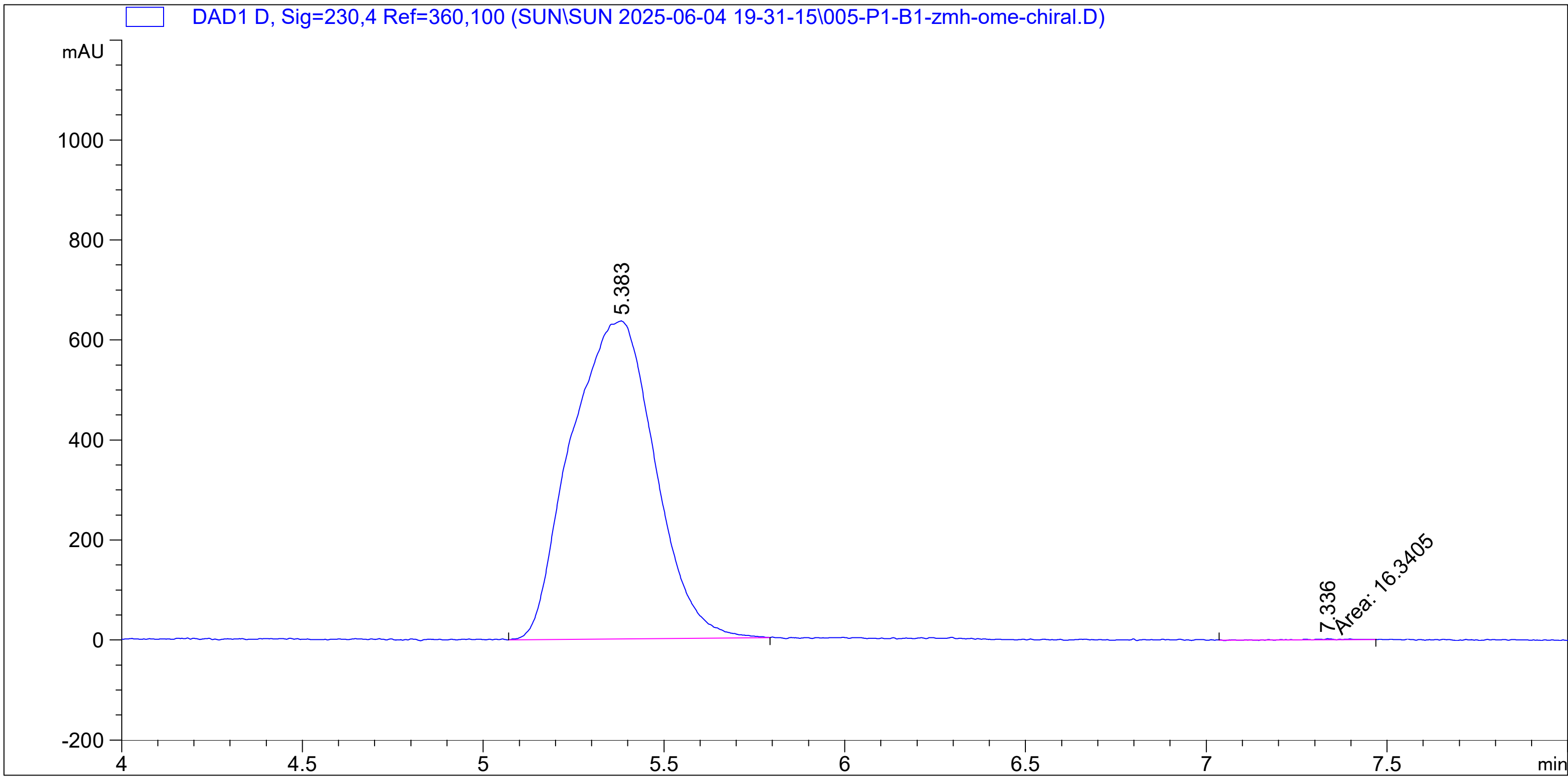

===

Area Percent Report

===

Sorted By : Signal
Multiplier : 1.0000
Dilution : 1.0000
Use Multiplier & Dilution Factor with ISTDs

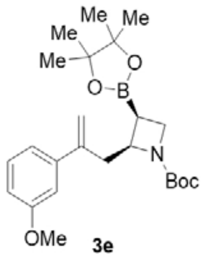

3e
enantioenriched

Signal 1: DAD1 D, Sig=230,4 Ref=360,100

Peak #	RetTime [min]	Type	Width [min]	Area [mAU*s]	Height [mAU]	Area %
1	5.383	BV R	0.1916	1.02530e4	636.06750	99.8409
2	7.336	MM	0.1275	16.34045	2.13558	0.1591

Totals : 1.02693e4 638.20308

===

Acq. Operator : SYSTEM   Seq. Line : 20
Sample Operator : SYSTEM
Acq. Instrument : HPLC   Location : P1-A-01
Injection Date : 9/11/2024 3:50:16 am   Inj : 1
                                          Inj Volume : 2.000 µl
Different Inj Volume from Sample Entry!   Actual Inj Volume : 10.000 µl
Acq. Method : C:\Users\Public\Documents\ChemStation\1\Data\SUN\SUN 2024-11-08 22-26-24\ID3-10-20.M
Last changed : 2/8/2023 12:18:49 pm by SYSTEM
Analysis Method : C:\Users\Public\Documents\ChemStation\1\Data\SUN\SUN 2024-11-08 22-26-24\ID3-10-20.M (Sequence Method)
Last changed : 8/5/2025 5:19:08 pm by SYSTEM
(modified after loading)
Additional Info : Peak(s) manually integrated

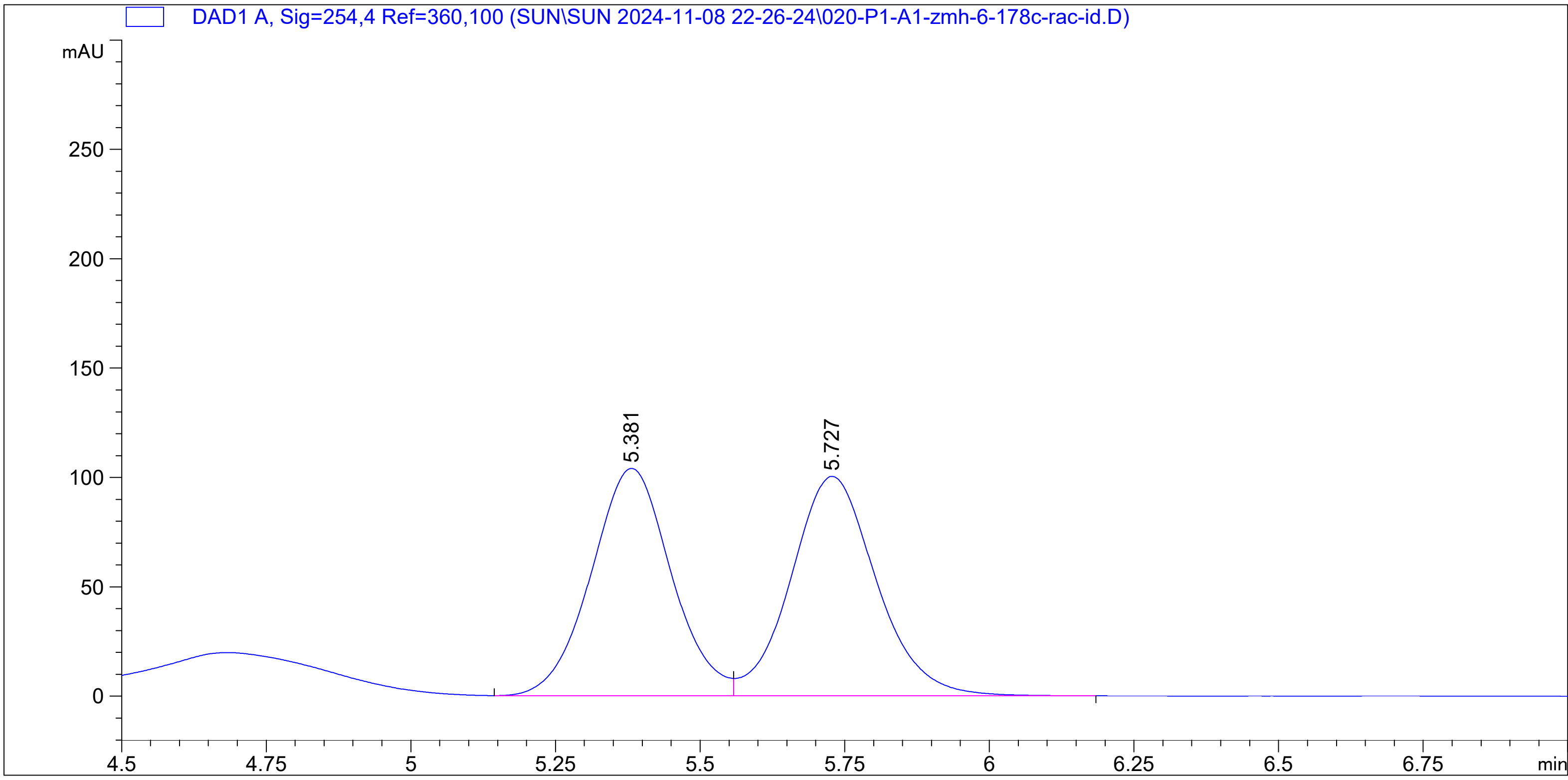

===

Area Percent Report

===

Sorted By : Signal
Multiplier : 1.0000
Dilution : 1.0000
Use Multiplier & Dilution Factor with ISTDs

Signal 1: DAD1 A, Sig=254,4 Ref=360,100

Peak #	RetTime [min]	Type	Width [min]	Area [mAU*s]	Height [mAU]	Area %
1	5.381	BV	0.1464	1001.45251	103.90340	49.3482
2	5.727	VB	0.1550	1027.90588	100.31222	50.6518

Totals : 2029.35840 204.21561

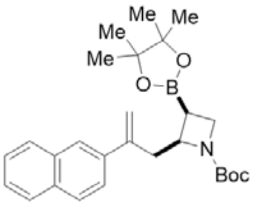

3f
racemic

Data File C:\Users\P...on\1\Data\SUN\SUN 2024-11-09 10-01-45\004-P1-A3-zmh-6-177c-chiral.D
Sample Name: zmh-6-177c-chiral

===

Acq. Operator : SYSTEM Seq. Line : 4
Sample Operator : SYSTEM
Acq. Instrument : HPLC Location : P1-A-03
Injection Date : 9/11/2024 10:47:01 am Inj : 1
Inj Volume : 2.000 µl
Different Inj Volume from Sample Entry! Actual Inj Volume : 10.000 µl
Acq. Method : C:\Users\Public\Documents\ChemStation\1\Data\SUN\SUN 2024-11-09 10-01-45\ID3-10-20.M
Last changed : 2/8/2023 12:18:49 pm by SYSTEM
Analysis Method : C:\Users\Public\Documents\ChemStation\1\Data\SUN\SUN 2024-11-09 10-01-45\ID3-10-20.M (Sequence Method)
Last changed : 8/5/2025 10:57:14 pm by SYSTEM
(modified after loading)
Additional Info : Peak(s) manually integrated

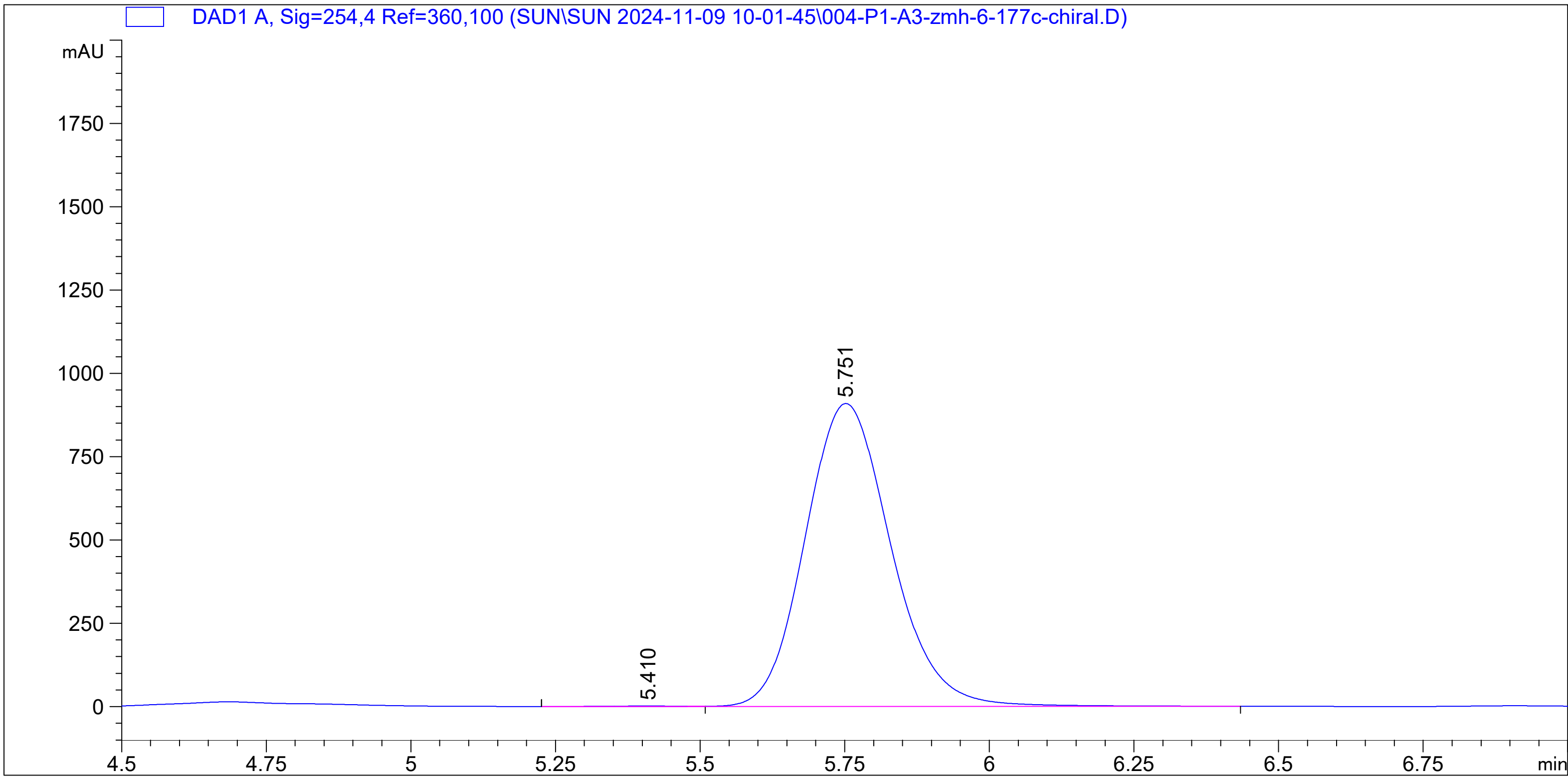

===

Area Percent Report

===

Sorted By : Signal
Multiplier : 1.0000
Dilution : 1.0000
Use Multiplier & Dilution Factor with ISTDs

Signal 1: DAD1 A, Sig=254,4 Ref=360,100

Peak #	RetTime [min]	Type	Width [min]	Area [mAU*s]	Height [mAU]	Area %
1	5.410	BV E	0.0955	10.57833	1.35558	0.1128
2	5.751	VB R	0.1608	9368.06445	908.82202	99.8872

Totals : 9378.64278 910.17760

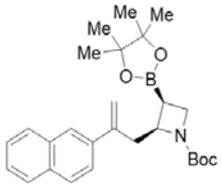

(*S*,*S*)-**3f**
enantioenriched

===

Acq. Operator : SYSTEM Seq. Line : 9
Sample Operator : SYSTEM
Acq. Instrument : HPLC Location : P2-A-06
Injection Date : 1/1/2025 3:38:01 pm Inj : 1
Inj Volume : 2.000 µl
Different Inj Volume from Sample Entry! Actual Inj Volume : 10.000 µl
Acq. Method : C:\Users\Public\Documents\ChemStation\1\Data\SUN\SUN 2025-01-01 13-17-07\IC3-10-20.M
Last changed : 15/8/2022 10:26:28 pm by SYSTEM
Analysis Method : C:\Users\Public\Documents\ChemStation\1\Data\SUN\SUN 2025-01-01 13-17-07\IC3-10-20.M (Sequence Method)
Last changed : 8/5/2025 5:29:14 pm by SYSTEM
(modified after loading)
Additional Info : Peak(s) manually integrated

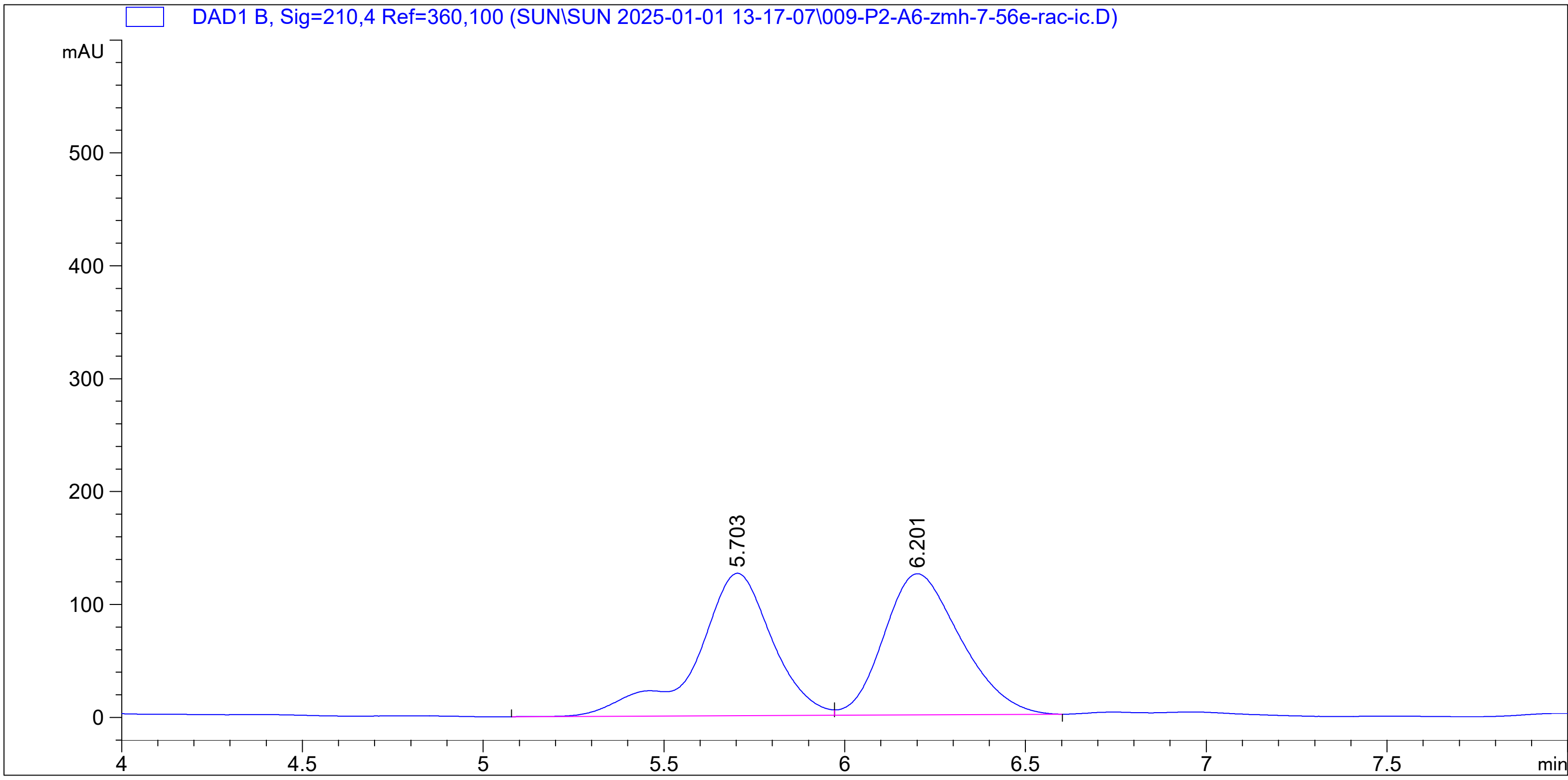

===

Area Percent Report

===

Sorted By : Signal
Multiplier : 1.0000
Dilution : 1.0000
Use Multiplier & Dilution Factor with ISTDs

Signal 1: DAD1 B, Sig=210,4 Ref=360,100

Peak #	RetTime [min]	Type	Width [min]	Area [mAU*s]	Height [mAU]	Area %
1	5.703	VV R	0.2002	1853.17004	126.13860	50.0906
2	6.201	VB	0.2233	1846.46899	125.02837	49.9094

Totals : 3699.63904 251.16697

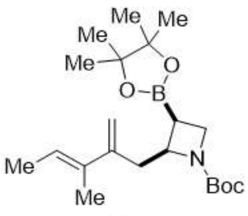

3g
racemic

Data File C:\Users\P...ion\1\Data\SUN\SUN 2025-01-01 13-17-07\096-P1-B5-zmh-7-25a-chiral.D
Sample Name: zmh-7-25a-chiral

===

Acq. Operator : SYSTEM Seq. Line : 96
Sample Operator : SYSTEM
Acq. Instrument : HPLC Location : P1-B-05
Injection Date : 2/1/2025 5:52:19 pm Inj : 1
Inj Volume : 2.000 µl
Different Inj Volume from Sample Entry! Actual Inj Volume : 10.000 µl
Acq. Method : C:\Users\Public\Documents\ChemStation\1\Data\SUN\SUN 2025-01-01 13-17-07\IC3-10-20.M
Last changed : 15/8/2022 10:26:28 pm by SYSTEM
Analysis Method : C:\Users\Public\Documents\ChemStation\1\Data\SUN\SUN 2025-01-01 13-17-07\IC3-10-20.M (Sequence Method)
Last changed : 8/5/2025 5:33:25 pm by SYSTEM
(modified after loading)
Additional Info : Peak(s) manually integrated

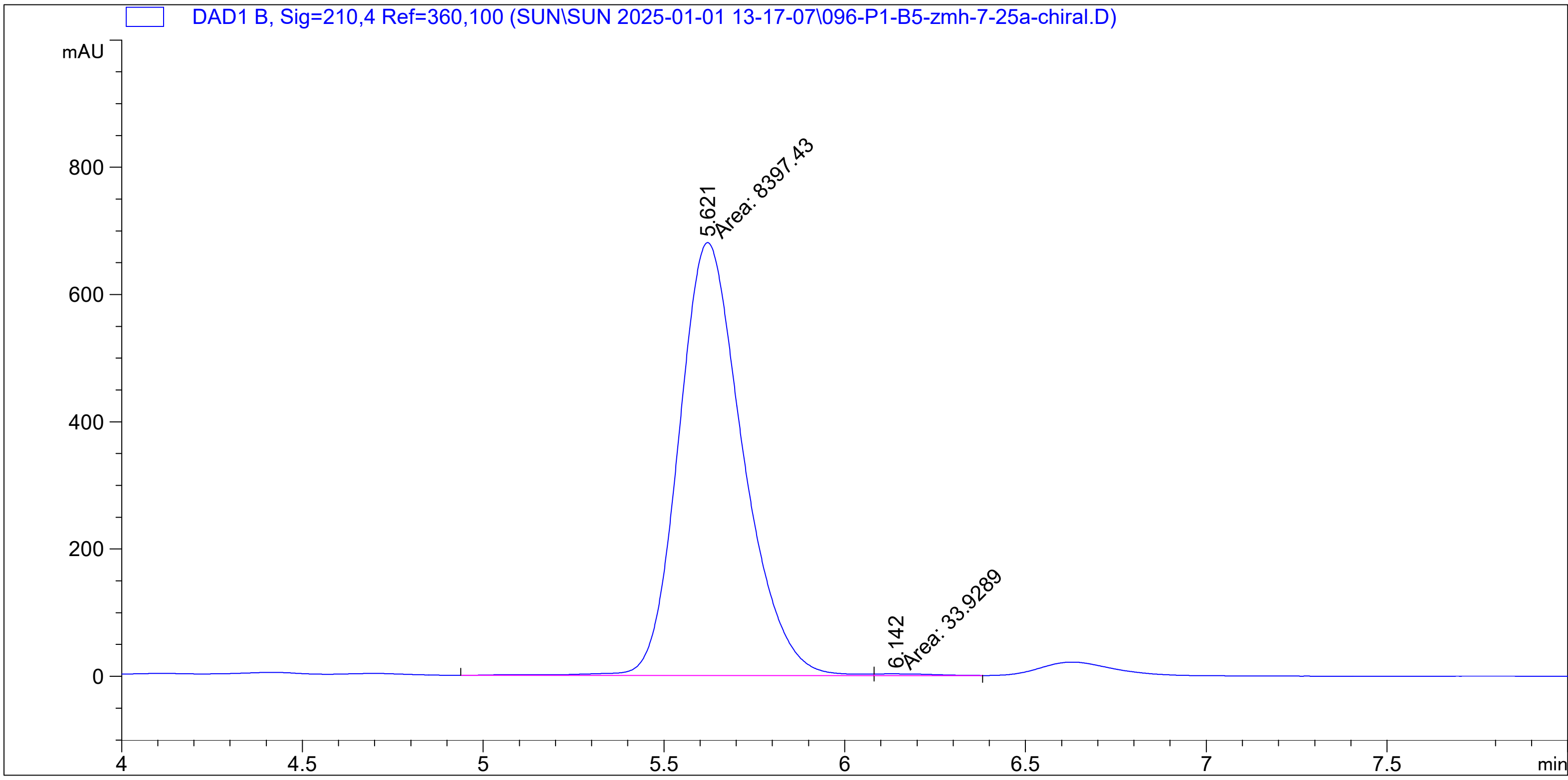

===
Area Percent Report
===

Sorted By : Signal
Multiplier : 1.0000
Dilution : 1.0000
Use Multiplier & Dilution Factor with ISTDs

Signal 1: DAD1 B, Sig=210,4 Ref=360,100

Peak #	RetTime [min]	Type	Width [min]	Area [mAU*s]	Height [mAU]	Area %
1	5.621	MF	0.2057	8397.43262	680.51459	99.5976
2	6.142	FM	0.1906	33.92895	2.96634	0.4024

Totals : 8431.36156 683.48092

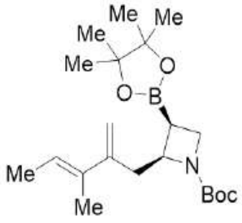

(*S*,*S*)-**3g**
enantioenriched

Data File C:\Users\P...ion\1\Data\SUN\SUN 2025-02-25 19-24-42\008-P1-B1-zmh-7-113-rac-ay.D
Sample Name: zmh-7-113-rac-ay

===

```
Acq. Operator   : SYSTEM                        Seq. Line :    8
Acq. Instrument : LC1260                         Location :   P1-B-01
Injection Date  : 2/25/2025 9:53:36 PM                Inj :    1
                                               Inj Volume : 5.000 µl
Different Inj Volume from Sample Entry!  Actual Inj Volume : 20.000 µl
Acq. Method     : C:\Users\Public\Documents\ChemStation\1\Data\SUN\SUN 2025-02-25 19-24-42
                  \AY3-10-20.M
Last changed    : 9/5/2024 2:31:02 PM by SYSTEM
Analysis Method : C:\Users\Public\Documents\ChemStation\1\Data\SUN\SUN 2025-02-25 19-24-42
                  \AY3-10-20.M (Sequence Method)
Last changed    : 6/2/2025 10:34:51 PM by SYSTEM
                  (modified after loading)
Additional Info : Peak(s) manually integrated
```

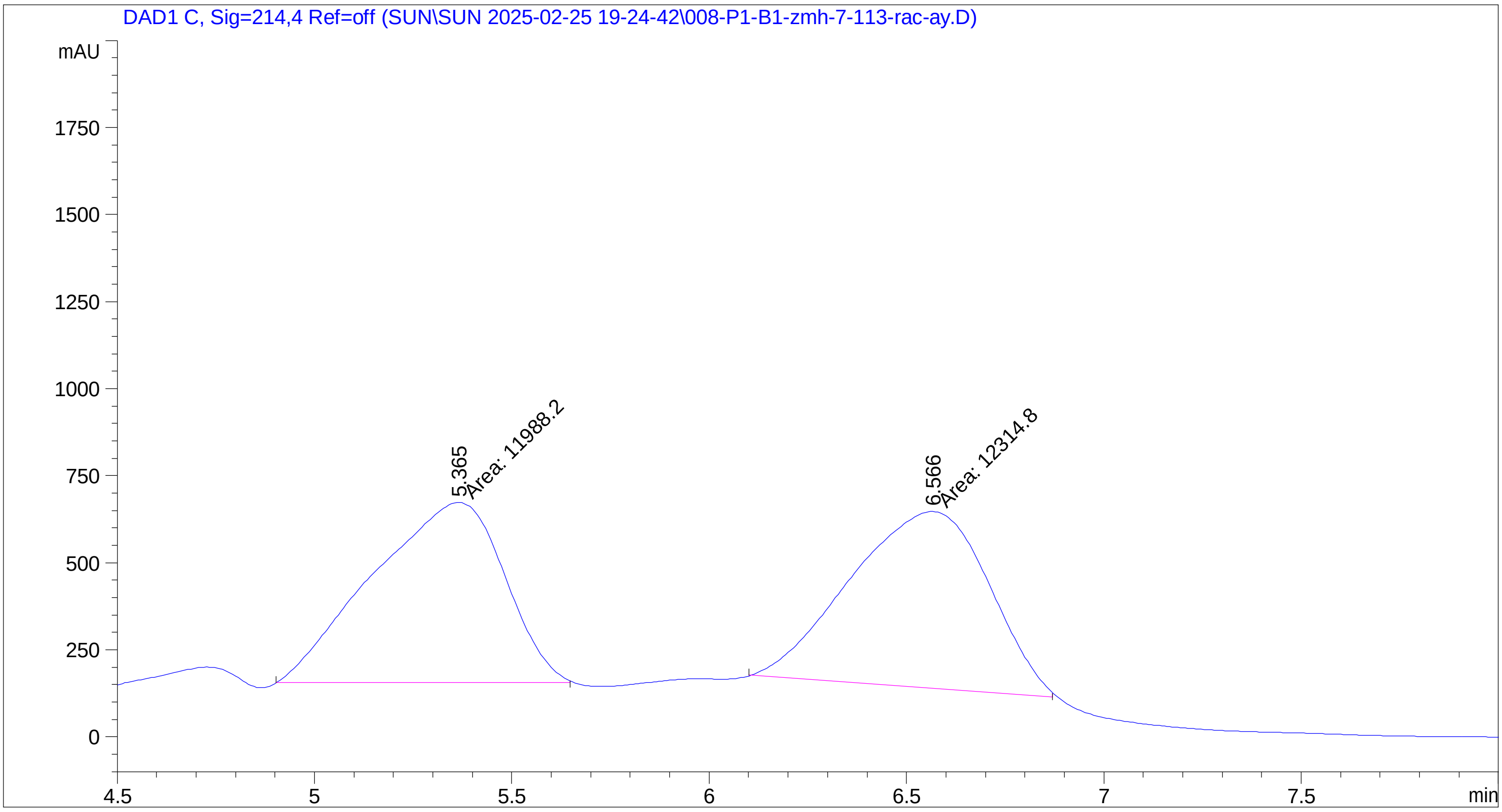

===

Area Percent Report

===

```
Sorted By             :      Signal
Multiplier            :      1.0000
Dilution              :      1.0000
Use Multiplier & Dilution Factor with ISTDs
```

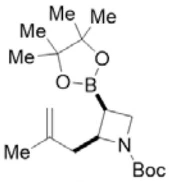

3h
racemic

Signal 1: DAD1 C, Sig=214,4 Ref=off

Peak #	RetTime [min]	Type	Width [min]	Area [mAU*s]	Height [mAU]	Area %
1	5.365	MM	0.3870	1.19882e4	516.27539	49.3280
2	6.566	MM	0.4045	1.23148e4	507.40717	50.6720

Totals : 2.43030e4 1023.68256

Data File C:\Users\P...ion\1\Data\SUN\SUN 2025-02-25 19-24-42\010-P1-B2-zmh-7-113-chiral.D
Sample Name: zmh-7-113-chiral

===

```
Acq. Operator   : SYSTEM                        Seq. Line :  10
                                                 Location :   P1-B-02
Injection Date  : 2/25/2025 10:25:37 PM               Inj :   1
Acq. Method     : AY3-10-20.M
Analysis Method : C:\Users\Public\Documents\ChemStation\1\Data\SUN\SUN 2025-02-25 19-24-42
                  \AY3-10-20.M (Sequence Method)
Last changed    : 5/8/2025 8:07:57 PM by SYSTEM
                  (modified after loading)
Additional Info : Peak(s) manually integrated
```

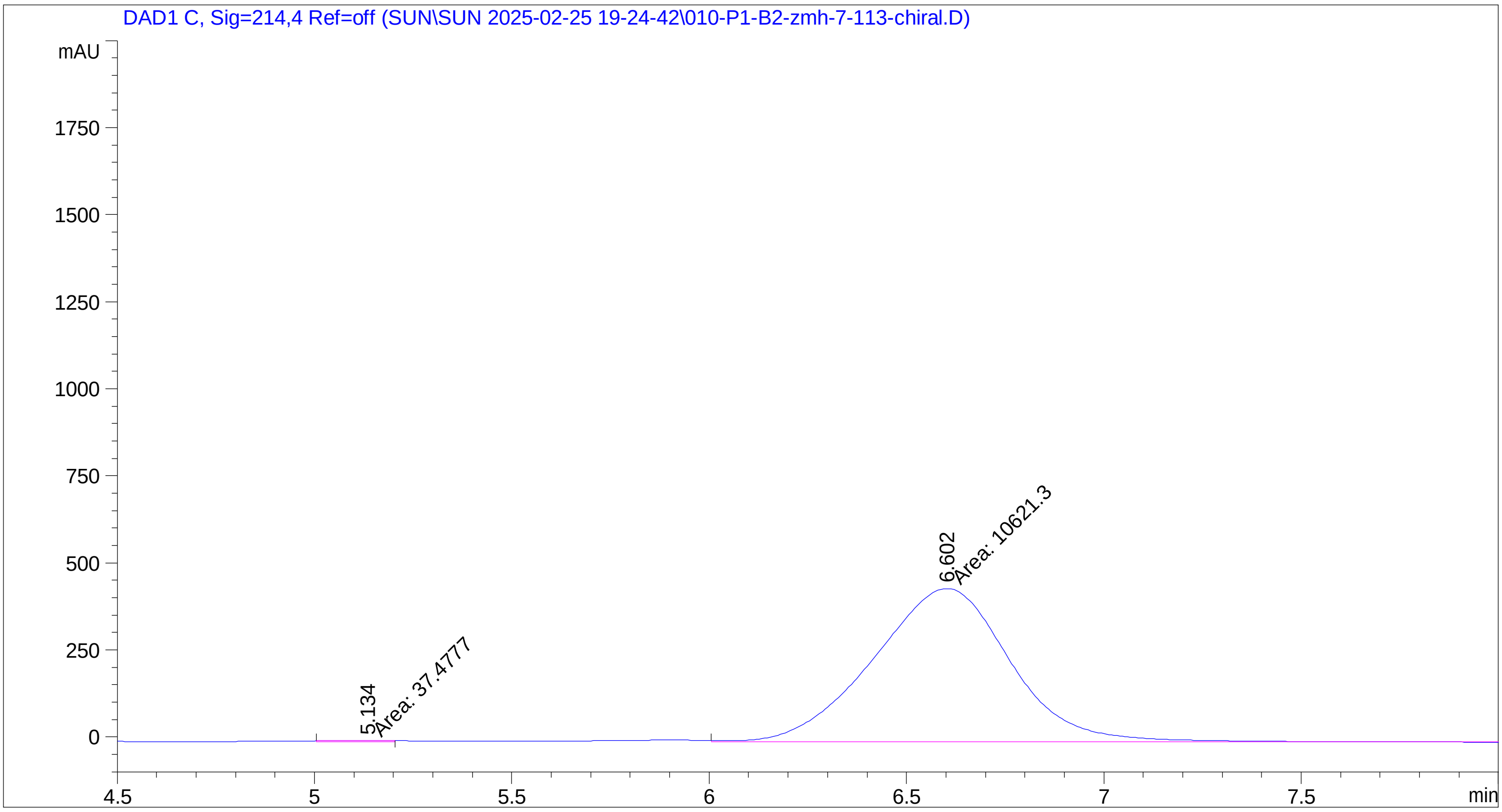

===

Area Percent Report

===

```
Sorted By             :      Signal
Multiplier            :      1.0000
Dilution              :      1.0000
Use Multiplier & Dilution Factor with ISTDs
```

Signal 1: DAD1 C, Sig=214,4 Ref=off

Peak #	RetTime [min]	Type	Width [min]	Area [mAU*s]	Height [mAU]	Area %
1	5.134	MF	0.1734	37.47768	3.60177	0.3516
2	6.602	FM	0.4024	1.06213e4	439.94064	99.6484

Totals : 1.06588e4 443.54242

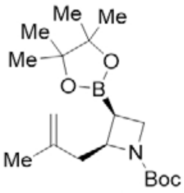

(*S*,*S*)-**3h**
enantioenriched

===

*** End of Report ***

Data File C:\Users\P...on\1\Data\SUN\SUN 2025-02-26 16-02-52\116-P1-E1-zmh-7-114b-rac-ic.D
Sample Name: zmh-7-114b-rac-ic

===

Acq. Operator : SYSTEM   Seq. Line : 116
Acq. Instrument : LC1260   Location : P1-E-01
Injection Date : 2/28/2025 2:56:54 AM   Inj : 1
Inj Volume : 5.000 µl
Different Inj Volume from Sample Entry! Actual Inj Volume : 10.000 µl
Acq. Method : C:\Users\Public\Documents\ChemStation\1\Data\SUN\SUN 2025-02-26 16-02-52\IC3-10-30.M
Last changed : 11/3/2024 4:34:55 PM by SYSTEM
Analysis Method : C:\Users\Public\Documents\ChemStation\1\Data\SUN\SUN 2025-02-26 16-02-52\IC3-10-30.M (Sequence Method)
Last changed : 6/2/2025 10:26:24 PM by SYSTEM
(modified after loading)
Additional Info : Peak(s) manually integrated

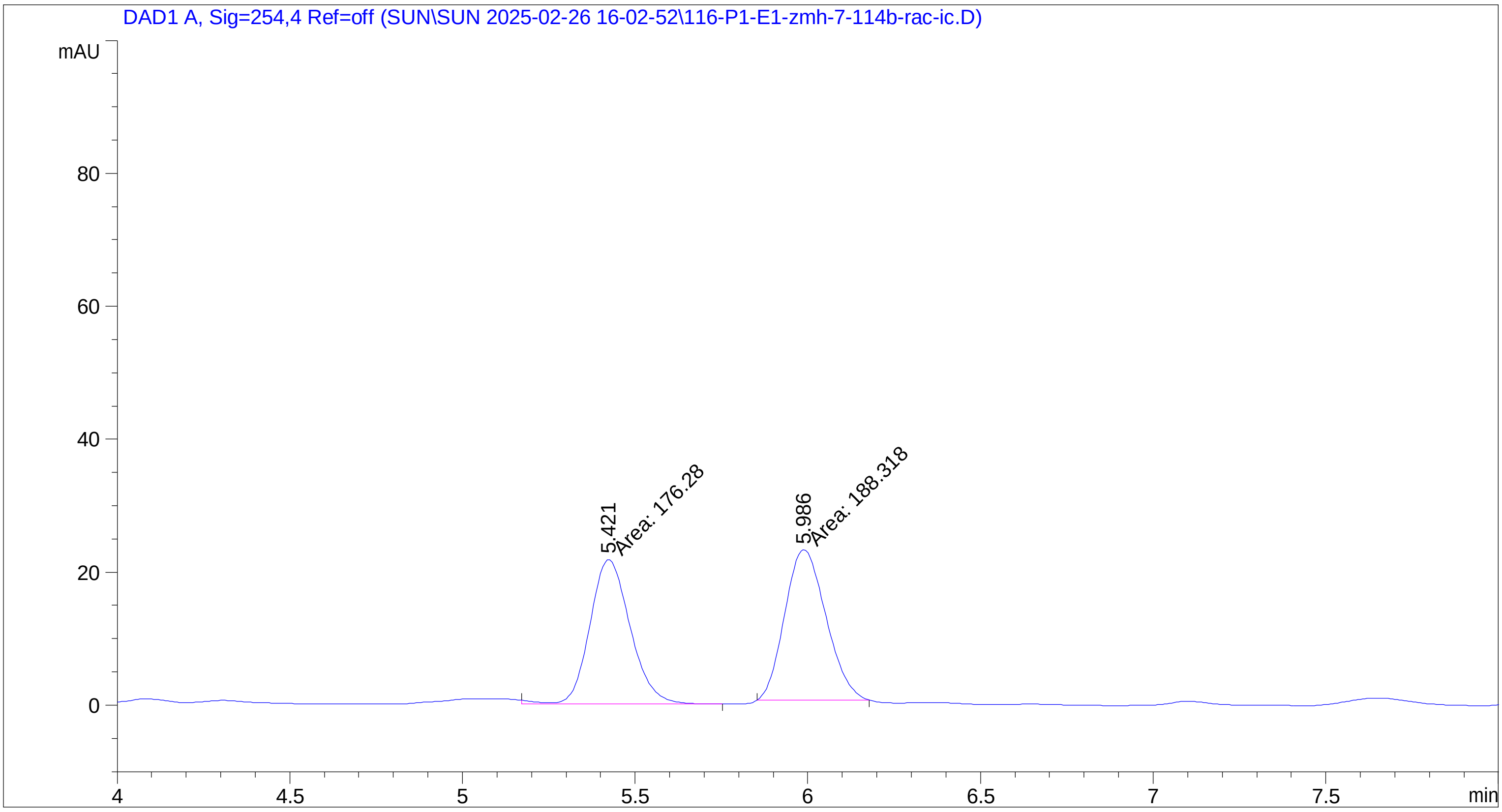

===

Area Percent Report

===

Sorted By : Signal
Multiplier : 1.0000
Dilution : 1.0000
Use Multiplier & Dilution Factor with ISTDs

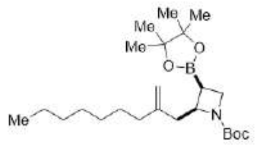

3i
racemic

Signal 1: DAD1 A, Sig=254,4 Ref=off

Peak #	RetTime [min]	Type	Width [min]	Area [mAU*s]	Height [mAU]	Area %
1	5.421	FM	0.1351	176.27971	21.74137	48.3491
2	5.986	MM	0.1384	188.31805	22.67477	51.6509

Totals : 364.59776 44.41613

Data File C:\Users\P...1\Data\SUN\SUN 2025-03-01 09-55-58\020-P1-A1-zmh-7-114b-chiral-ic.D
Sample Name: zmh-7-114b-chiral-ic

===

Acq. Operator : SYSTEM   Seq. Line : 20
Acq. Instrument : LC1260   Location : P1-A-01
Injection Date : 3/1/2025 5:03:17 PM   Inj : 1
Inj Volume : 5.000 µl
Different Inj Volume from Sample Entry! Actual Inj Volume : 10.000 µl
Acq. Method : C:\Users\Public\Documents\ChemStation\1\Data\SUN\SUN 2025-03-01 09-55-58\IC3-10-30.M
Last changed : 11/3/2024 4:34:55 PM by SYSTEM
Analysis Method : C:\Users\Public\Documents\ChemStation\1\Data\SUN\SUN 2025-03-01 09-55-58\IC3-10-30.M (Sequence Method)
Last changed : 5/8/2025 10:35:29 PM by SYSTEM
(modified after loading)
Additional Info : Peak(s) manually integrated

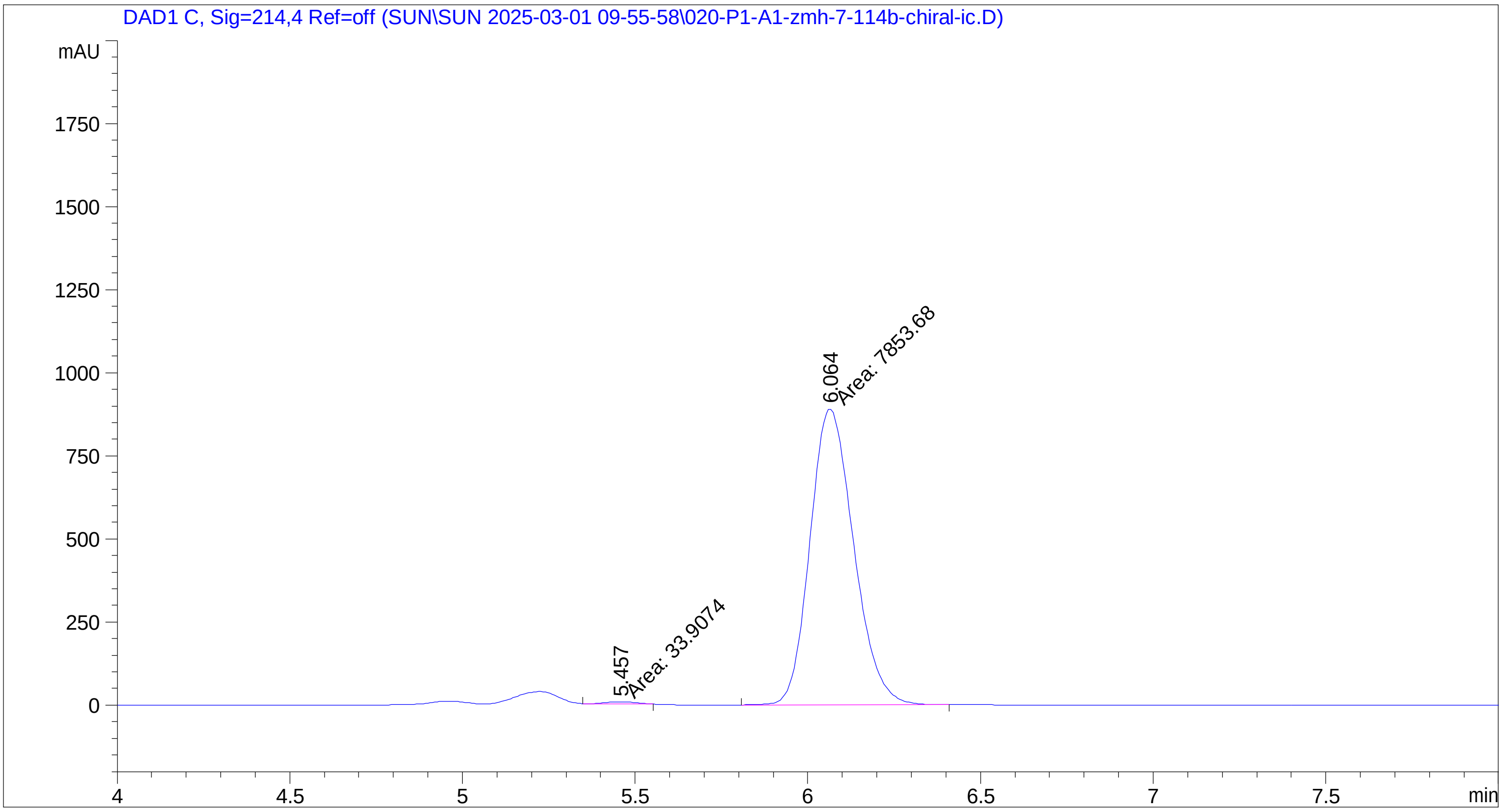

===

Area Percent Report

===

Sorted By : Signal
Multiplier : 1.0000
Dilution : 1.0000
Use Multiplier & Dilution Factor with ISTDs

Signal 1: DAD1 C, Sig=214,4 Ref=off

Peak #	RetTime [min]	Type	Width [min]	Area [mAU*s]	Height [mAU]	Area %
1	5.457	MM	0.0972	33.90743	5.81482	0.4299
2	6.064	MM	0.1469	7853.68262	890.89148	99.5701

Totals : 7887.59005 896.70630

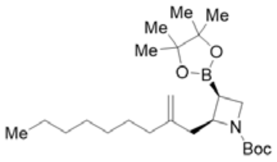

(*S*,*S*)-**3i**
enantioenriched

Data File C:\Users\P...1\Data\SUN\SUN 2024-11-12 19-46-23\028-P1-A1-zmh-7-3bshang-rac-oz.D
Sample Name: zmh-7-3bshang-rac-oz

===

Acq. Operator : SYSTEM   Seq. Line : 28
Sample Operator : SYSTEM
Acq. Instrument : HPLC   Location : P1-A-01
Injection Date : 13/11/2024 3:10:01 am   Inj : 1
Inj Volume : 2.000 µl
Different Inj Volume from Sample Entry! Actual Inj Volume : 20.000 µl
Acq. Method : C:\Users\Public\Documents\ChemStation\1\Data\SUN\SUN 2024-11-12 19-46-23\OZ3-10-20.M
Last changed : 15/8/2022 10:41:10 pm by SYSTEM
Analysis Method : C:\Users\Public\Documents\ChemStation\1\Data\SUN\SUN 2024-11-12 19-46-23\OZ3-10-20.M (Sequence Method)
Last changed : 8/5/2025 5:41:15 pm by SYSTEM
(modified after loading)
Additional Info : Peak(s) manually integrated

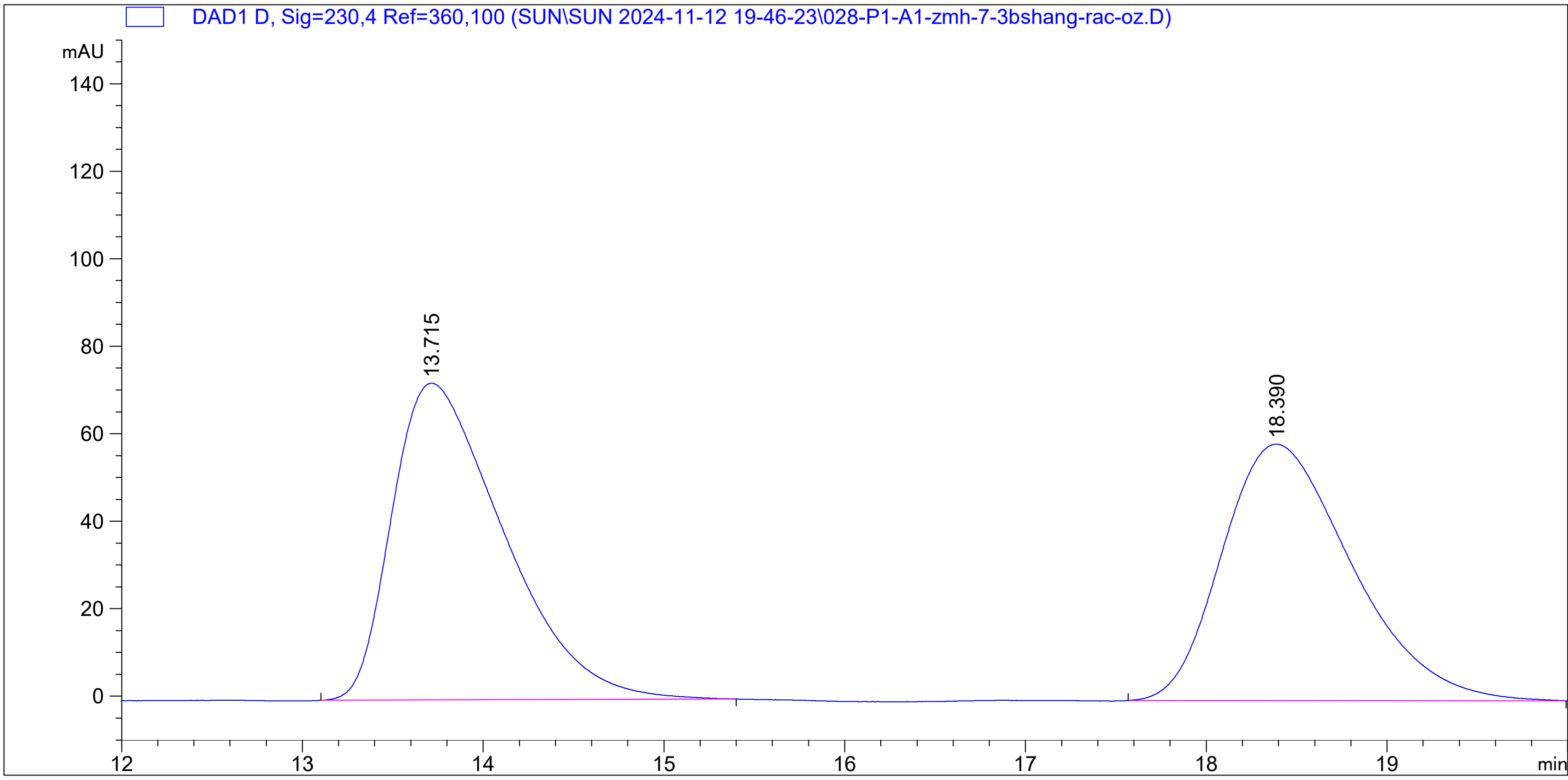

===

Area Percent Report

===

Sorted By : Signal
Multiplier : 1.0000
Dilution : 1.0000
Use Multiplier & Dilution Factor with ISTDs

Signal 1: DAD1 D, Sig=230,4 Ref=360,100

Peak #	RetTime [min]	Type	Width [min]	Area [mAU*s]	Height [mAU]	Area %
1	13.715	BB	0.5117	3105.40088	72.43050	51.5606
2	18.390	BBA	0.5946	2917.42017	58.62402	48.4394
Totals :				6022.82104	131.05452	

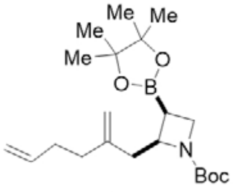

3j
racemic

Data File C:\Users\P...tion\1\Data\SUN\SUN 2024-11-14 10-08-31\029-P1-B1-zmh-7-3c-chiral.D
Sample Name: zmh-7-3c-chiral

===

Acq. Operator : SYSTEM Seq. Line : 29
Sample Operator : SYSTEM
Acq. Instrument : HPLC Location : P1-B-01
Injection Date : 14/11/2024 4:16:22 pm Inj : 1
Inj Volume : 2.000 µl
Different Inj Volume from Sample Entry! Actual Inj Volume : 20.000 µl
Acq. Method : C:\Users\Public\Documents\ChemStation\1\Data\SUN\SUN 2024-11-14 10-08-31\OZ3-10-20.M
Last changed : 15/8/2022 10:41:10 pm by SYSTEM
Analysis Method : C:\Users\Public\Documents\ChemStation\1\Data\SUN\SUN 2024-11-14 10-08-31\OZ3-10-20.M (Sequence Method)
Last changed : 8/5/2025 5:44:00 pm by SYSTEM
(modified after loading)
Additional Info : Peak(s) manually integrated

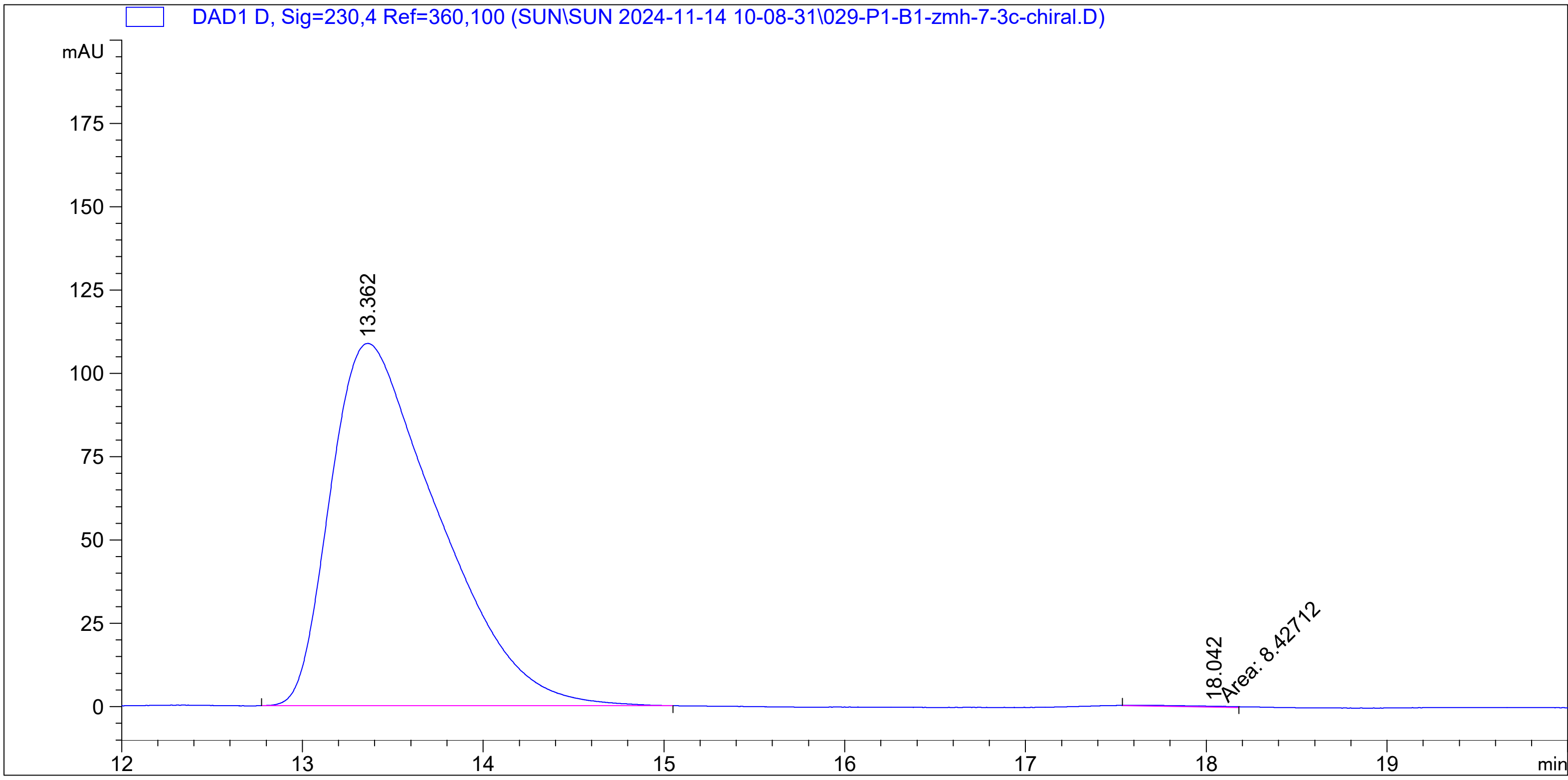

===

Area Percent Report

===

Sorted By : Signal
Multiplier : 1.0000
Dilution : 1.0000
Use Multiplier & Dilution Factor with ISTDs

Signal 1: DAD1 D, Sig=230,4 Ref=360,100

Peak #	RetTime [min]	Type	Width [min]	Area [mAU*s]	Height [mAU]	Area %
1	13.362	BB	0.5717	4506.19385	108.69097	99.8133
2	18.042	MM	0.5052	8.42712	2.78015e-1	0.1867

Totals : 4514.62097 108.96899

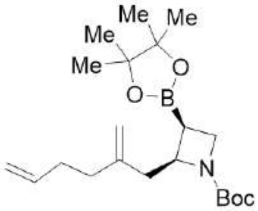

(*S*,*S*)-3j
enantioenriched

Data File C:\Users\P...ion\1\Data\SUN\SUN 2025-01-01 13-17-07\112-P1-E1-zmh-7-57a-rac-ox.D
Sample Name: zmh-7-57a-rac-ox

===

Acq. Operator : SYSTEM Seq. Line : 112
Sample Operator : SYSTEM
Acq. Instrument : HPLC Location : P1-E-01
Injection Date : 2/1/2025 10:17:03 pm Inj : 1
Inj Volume : 2.000 µl
Different Inj Volume from Sample Entry! Actual Inj Volume : 20.000 µl
Acq. Method : C:\Users\Public\Documents\ChemStation\1\Data\SUN\SUN 2025-01-01 13-17-07\OX3-5-30.M
Last changed : 25/8/2024 11:47:40 am by SYSTEM
Analysis Method : C:\Users\Public\Documents\ChemStation\1\Data\SUN\SUN 2025-01-01 13-17-07\OX3-5-30.M (Sequence Method)
Last changed : 3/6/2025 3:58:34 pm by SYSTEM
(modified after loading)
Additional Info : Peak(s) manually integrated

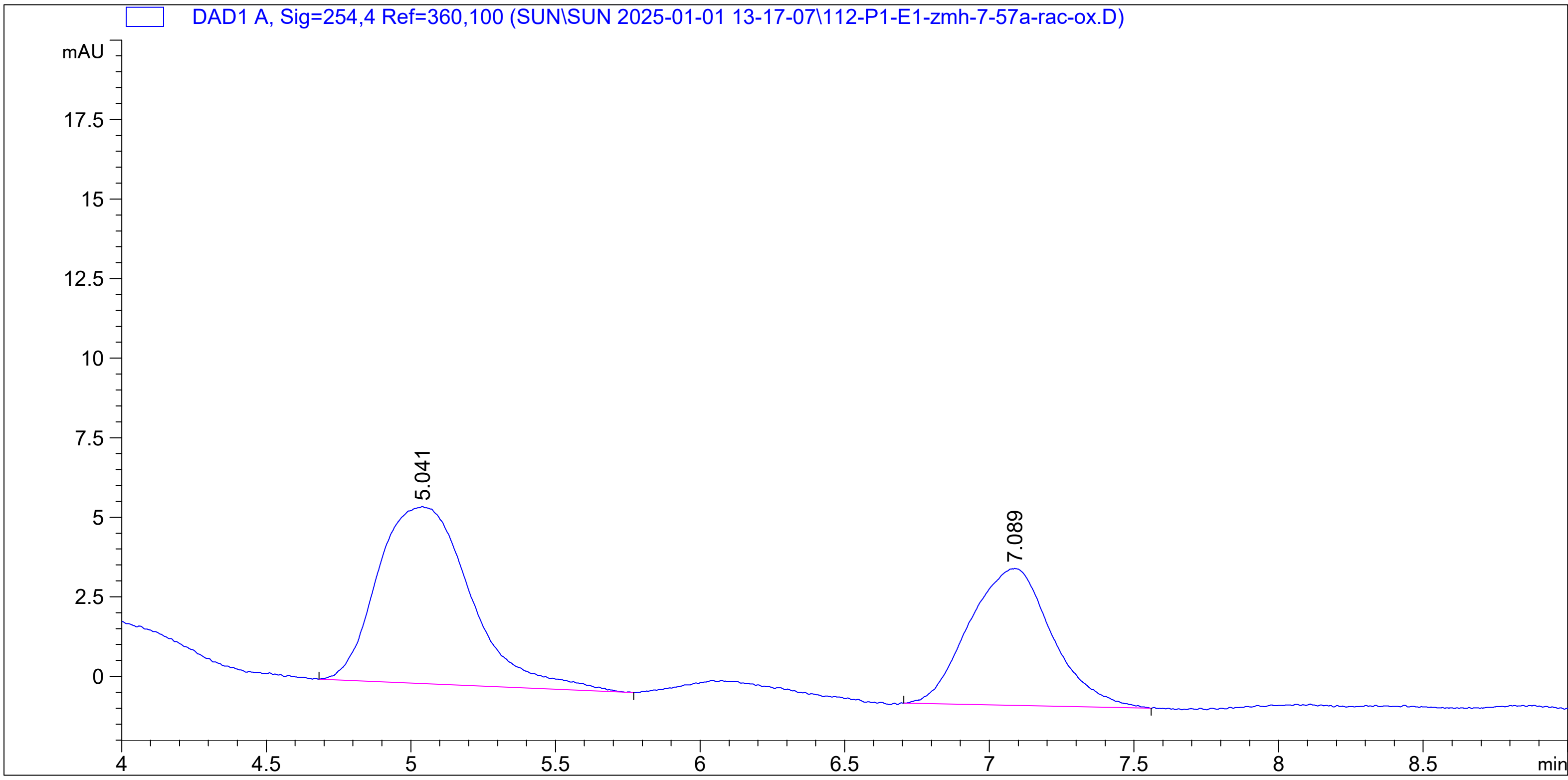

===

Area Percent Report

===

Sorted By : Signal
Multiplier : 1.0000
Dilution : 1.0000
Use Multiplier & Dilution Factor with ISTDs

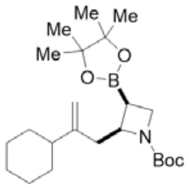

3k
racemic

Signal 1: DAD1 A, Sig=254,4 Ref=360,100

Peak #	RetTime [min]	Type	Width [min]	Area [mAU*s]	Height [mAU]	Area %
1	5.041	BB	0.2638	124.50056	5.56991	59.3029
2	7.089	BB	0.2333	85.43967	4.30111	40.6971

Totals : 209.94024 9.87102

Data File C:\Users\P...ion\1\Data\SUN\SUN 2025-01-01 13-17-07\144-P2-F1-zmh-7-28b-chiral.D
Sample Name: zmh-7-28b-chiral

===

Acq. Operator : SYSTEM   Seq. Line : 144
Sample Operator : SYSTEM
Acq. Instrument : HPLC   Location : P2-F-01
Injection Date : 3/1/2025 10:52:10 am   Inj : 1
Inj Volume : 2.000 µl
Different Inj Volume from Sample Entry! Actual Inj Volume : 20.000 µl
Acq. Method : C:\Users\Public\Documents\ChemStation\1\Data\SUN\SUN 2025-01-01 13-17-07\OX3-5-30.M
Last changed : 25/8/2024 11:47:40 am by SYSTEM
Analysis Method : C:\Users\Public\Documents\ChemStation\1\Data\SUN\SUN 2025-01-01 13-17-07\OX3-5-30.M (Sequence Method)
Last changed : 8/5/2025 5:52:17 pm by SYSTEM
(modified after loading)
Additional Info : Peak(s) manually integrated

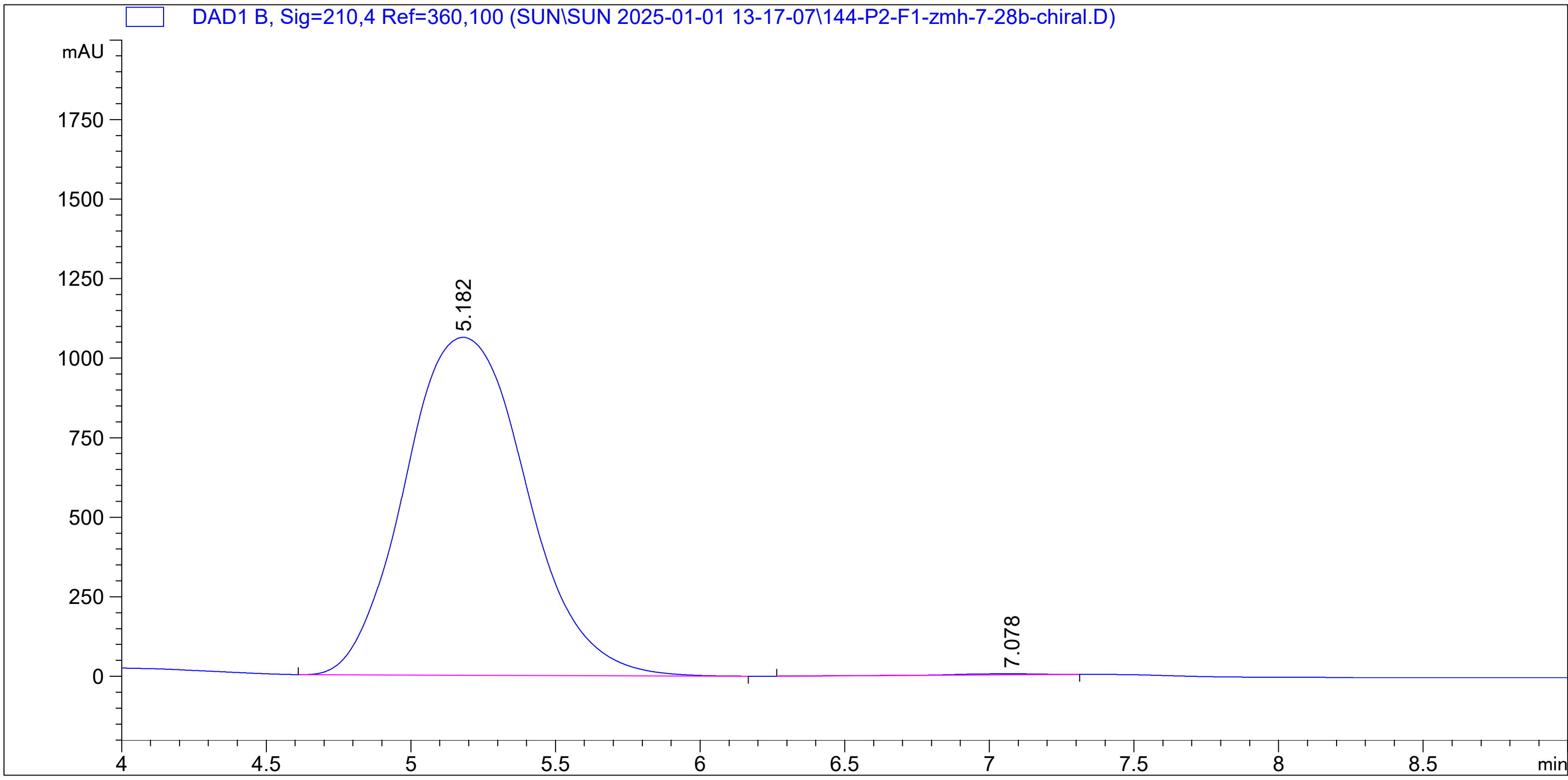

===

Area Percent Report

===

Sorted By : Signal
Multiplier : 1.0000
Dilution : 1.0000
Use Multiplier & Dilution Factor with ISTDs

Signal 1: DAD1 B, Sig=210,4 Ref=360,100

Peak #	RetTime [min]	Type	Width [min]	Area [mAU*s]	Height [mAU]	Area %
1	5.182	BB	0.3547	3.10194e4	1062.26709	99.7614
2	7.078	VB R	0.2539	74.17758	3.43489	0.2386

Totals : 3.10936e4 1065.70198

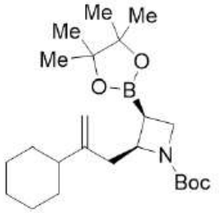

(*S*,*S*)-**3k**
enantioenriched

Data File C:\Users\P...ation\1\Data\SUN\SUN 2025-05-24 10-19-46\009-P1-F1-zmh-obn-rac-ic.D
Sample Name: zmh-obn-rac-ic

===

Acq. Operator : SYSTEM   Seq. Line : 9
Sample Operator : SYSTEM
Acq. Instrument : HPLC   Location : P1-F-01
Injection Date : 24/5/2025 11:51:45 am   Inj : 1
Inj Volume : 2.000 µl
Different Inj Volume from Sample Entry! Actual Inj Volume : 10.000 µl
Acq. Method : C:\Users\Public\Documents\ChemStation\1\Data\SUN\SUN 2025-05-24 10-19-46\IC3-10-20.M
Last changed : 15/8/2022 10:26:28 pm by SYSTEM
Analysis Method : C:\Users\Public\Documents\ChemStation\1\Data\SUN\SUN 2025-05-24 10-19-46\IC3-10-20.M (Sequence Method)
Last changed : 2/6/2025 10:09:13 pm by SYSTEM
(modified after loading)
Additional Info : Peak(s) manually integrated

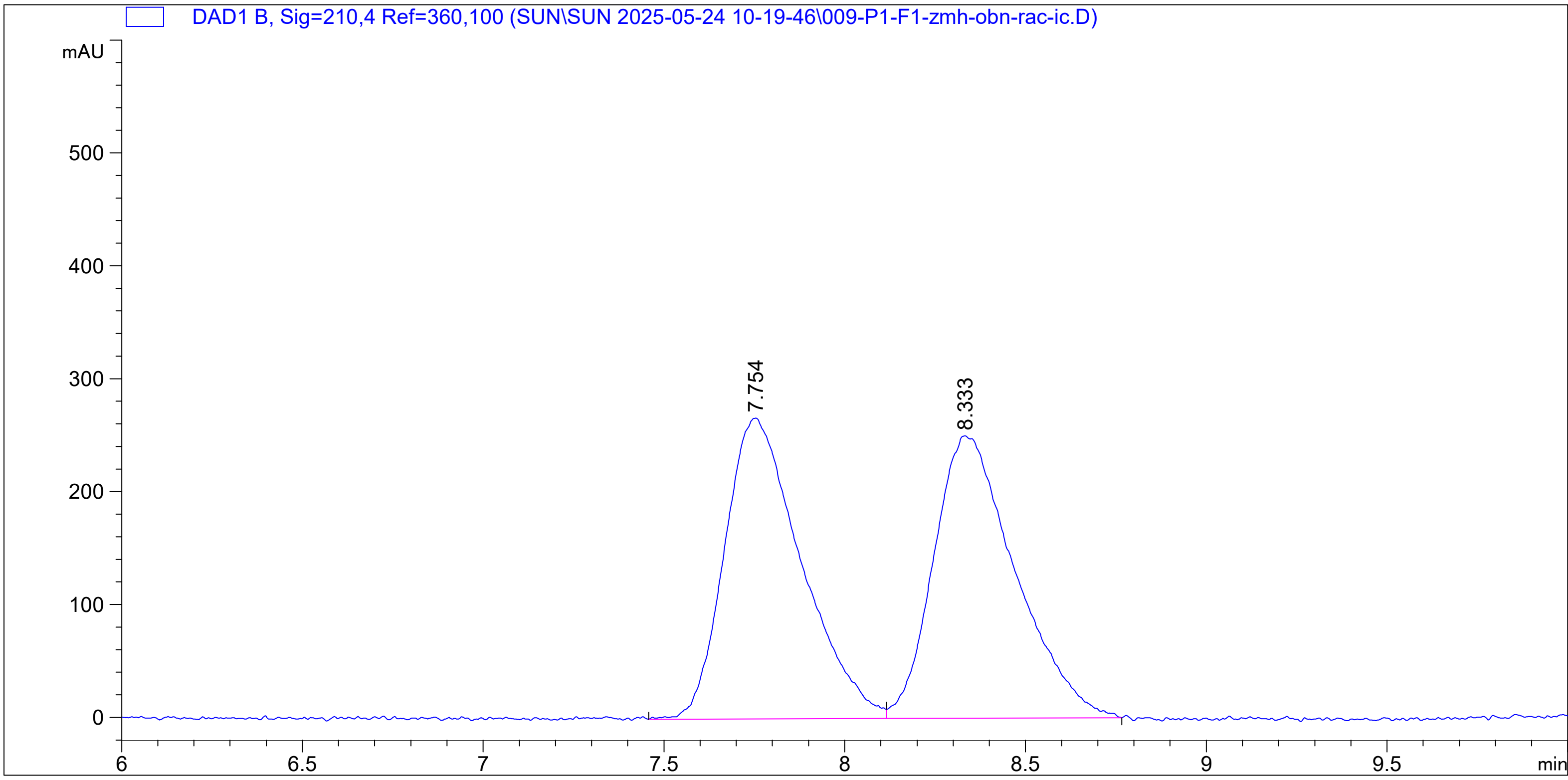

===

Area Percent Report

===

Sorted By : Signal
Multiplier : 1.0000
Dilution : 1.0000
Use Multiplier & Dilution Factor with ISTDs

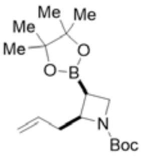

3l
racemic

Signal 1: DAD1 B, Sig=210,4 Ref=360,100

Peak #	RetTime [min]	Type	Width [min]	Area [mAU*s]	Height [mAU]	Area %
1	7.754	VV R	0.1764	3937.34253	266.47623	50.0822
2	8.333	VV R	0.1893	3924.41479	250.11562	49.9178

Totals : 7861.75732 516.59184

Data File C:\Users\P...\1\Data\SUN\SUN 2025-05-24 10-19-46\047-P2-A1-zmh-obn-chiral-re-1.D
Sample Name: zmh-obn-chiral-re-1

===

```
Acq. Operator   : SYSTEM                       Seq. Line :   47
Sample Operator : SYSTEM
Acq. Instrument : HPLC                          Location :   P2-A-01
Injection Date  : 24/5/2025 11:26:03 pm              Inj :   1
                                              Inj Volume : 2.000 µl
Different Inj Volume from Sample Entry!  Actual Inj Volume : 5.000 µl
Acq. Method     : C:\Users\Public\Documents\ChemStation\1\Data\SUN\SUN 2025-05-24 10-19-46\IC3-
                  10-20.M
Last changed    : 15/8/2022 10:26:28 pm by SYSTEM
Analysis Method : C:\Users\Public\Documents\ChemStation\1\Data\SUN\SUN 2025-05-24 10-19-46\IC3-
                  10-20.M (Sequence Method)
Last changed    : 2/6/2025 10:09:13 pm by SYSTEM
                  (modified after loading)
Additional Info : Peak(s) manually integrated
```

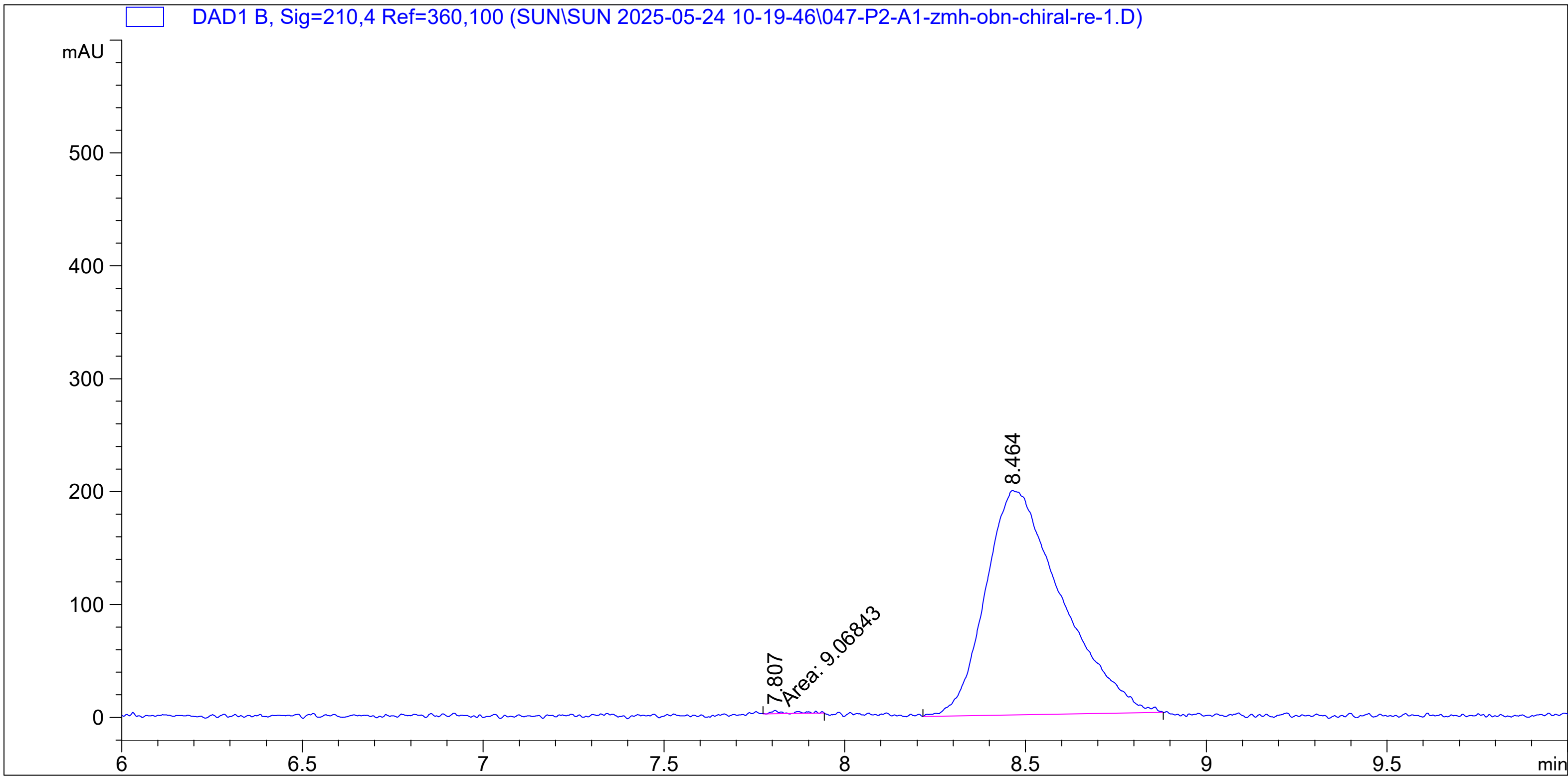

===
Area Percent Report
===

```
Sorted By             :      Signal
Multiplier            :      1.0000
Dilution              :      1.0000
Use Multiplier & Dilution Factor with ISTDs
```

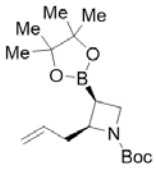

(*S*,*S*)-**3l**
enantioenriched

Signal 1: DAD1 B, Sig=210,4 Ref=360,100

Peak #	RetTime [min]	Type	Width [min]	Area [mAU*s]	Height [mAU]	Area %
1	7.807	MM	0.0505	9.06843	2.99389	0.3093
2	8.464	VV R	0.1750	2922.65430	198.84256	99.6907

Totals : 2931.72273 201.83645

Data File C:\Users\P...ion\1\Data\SUN\SUN 2025-01-01 13-17-07\136-P2-A1-zmh-7-54a-rac-ad.D
Sample Name: zmh-7-54a-rac-ad

===

Acq. Operator : SYSTEM Seq. Line : 136
Sample Operator : SYSTEM
Acq. Instrument : HPLC Location : P2-A-01
Injection Date : 3/1/2025 8:02:56 am Inj : 1
Inj Volume : 2.000 µl
Different Inj Volume from Sample Entry! Actual Inj Volume : 10.000 µl
Acq. Method : C:\Users\Public\Documents\ChemStation\1\Data\SUN\SUN 2025-01-01 13-17-07\AD3-20-30.M
Last changed : 18/8/2022 9:21:31 pm by SYSTEM
Analysis Method : C:\Users\Public\Documents\ChemStation\1\Data\SUN\SUN 2025-01-01 13-17-07\AD3-20-30.M (Sequence Method)
Last changed : 8/5/2025 6:41:19 pm by SYSTEM
(modified after loading)
Additional Info : Peak(s) manually integrated

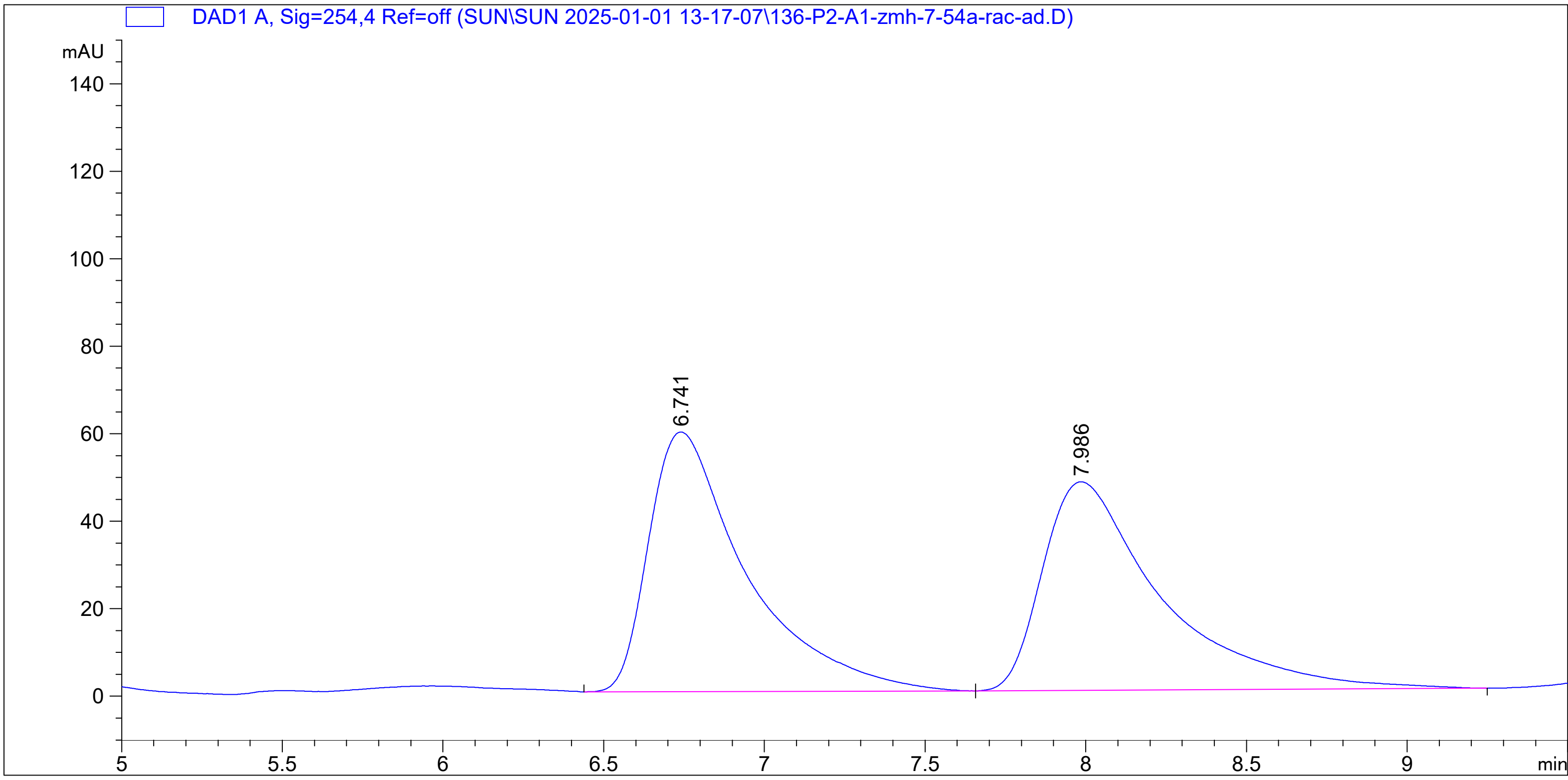

===

Area Percent Report

===

Sorted By : Signal
Multiplier : 1.0000
Dilution : 1.0000
Use Multiplier & Dilution Factor with ISTDs

Signal 1: DAD1 A, Sig=254,4 Ref=off

Peak #	RetTime [min]	Type	Width [min]	Area [mAU*s]	Height [mAU]	Area %
1	6.741	BB	0.3086	1249.89368	59.37252	50.5852
2	7.986	BB	0.3533	1220.97656	47.67228	49.4148

Totals : 2470.87024 107.04480

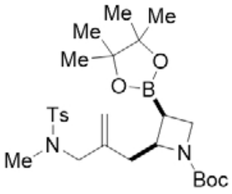

3m
racemic

Data File C:\Users\P...ion\1\Data\SUN\SUN 2025-01-03 11-28-32\008-P2-F4-zmh-7-28c-chiral.D
Sample Name: zmh-7-28c-chiral

===

Acq. Operator : SYSTEM Seq. Line : 8
Sample Operator : SYSTEM
Acq. Instrument : HPLC Location : P2-F-04
Injection Date : 3/1/2025 1:27:48 pm Inj : 1
Inj Volume : 2.000 µl
Different Inj Volume from Sample Entry! Actual Inj Volume : 20.000 µl
Acq. Method : C:\Users\Public\Documents\ChemStation\1\Data\SUN\SUN 2025-01-03 11-28-32\AD3-20-30.M
Last changed : 18/8/2022 9:21:31 pm by SYSTEM
Analysis Method : C:\Users\Public\Documents\ChemStation\1\Data\SUN\SUN 2025-01-03 11-28-32\AD3-20-30.M (Sequence Method)
Last changed : 8/5/2025 6:45:37 pm by SYSTEM
(modified after loading)
Additional Info : Peak(s) manually integrated

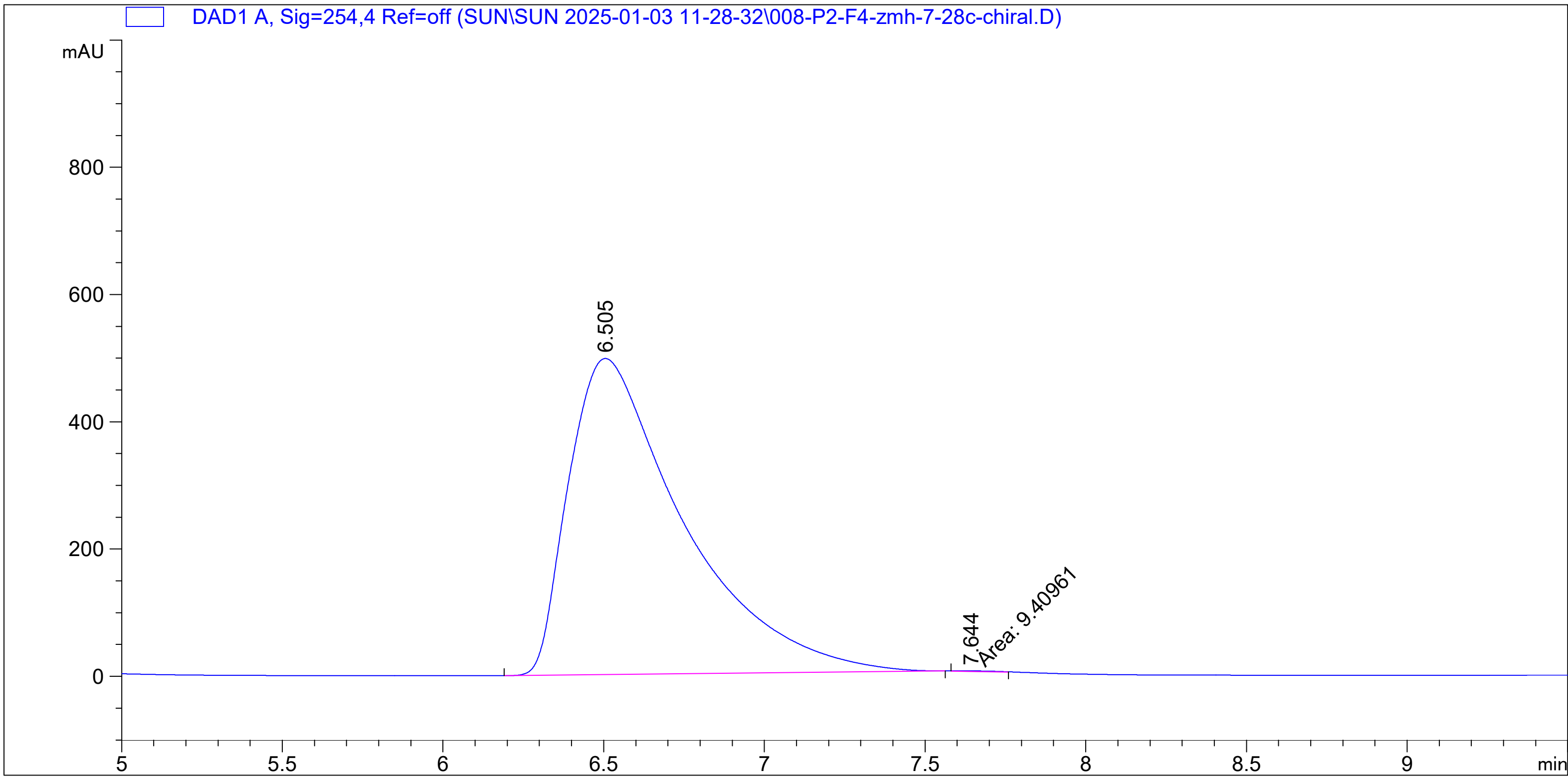

===

Area Percent Report

===

Sorted By : Signal
Multiplier : 1.0000
Dilution : 1.0000
Use Multiplier & Dilution Factor with ISTDs

Signal 1: DAD1 A, Sig=254,4 Ref=off

Peak #	RetTime [min]	Type	Width [min]	Area [mAU*s]	Height [mAU]	Area %
1	6.505	BB	0.3660	1.23252e4	496.74042	99.9237
2	7.644	MM	0.1436	9.40961	1.09192	0.0763

Totals : 1.23346e4 497.83234

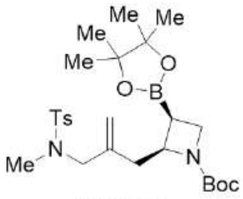

(*S*,*S*)-**3m**
enantioenriched

===

Acq. Operator : SYSTEM Seq. Line : 6
Sample Operator : SYSTEM
Acq. Instrument : HPLC Location : P2-A-02
Injection Date : 1/1/2025 2:34:38 pm Inj : 1
Inj Volume : 2.000 µl
Different Inj Volume from Sample Entry! Actual Inj Volume : 10.000 µl
Acq. Method : C:\Users\Public\Documents\ChemStation\1\Data\SUN\SUN 2025-01-01 13-17-07\IC3-10-20.M
Last changed : 15/8/2022 10:26:28 pm by SYSTEM
Analysis Method : C:\Users\Public\Documents\ChemStation\1\Data\SUN\SUN 2025-01-01 13-17-07\IC3-10-20.M (Sequence Method)
Last changed : 8/5/2025 6:50:19 pm by SYSTEM
(modified after loading)
Additional Info : Peak(s) manually integrated

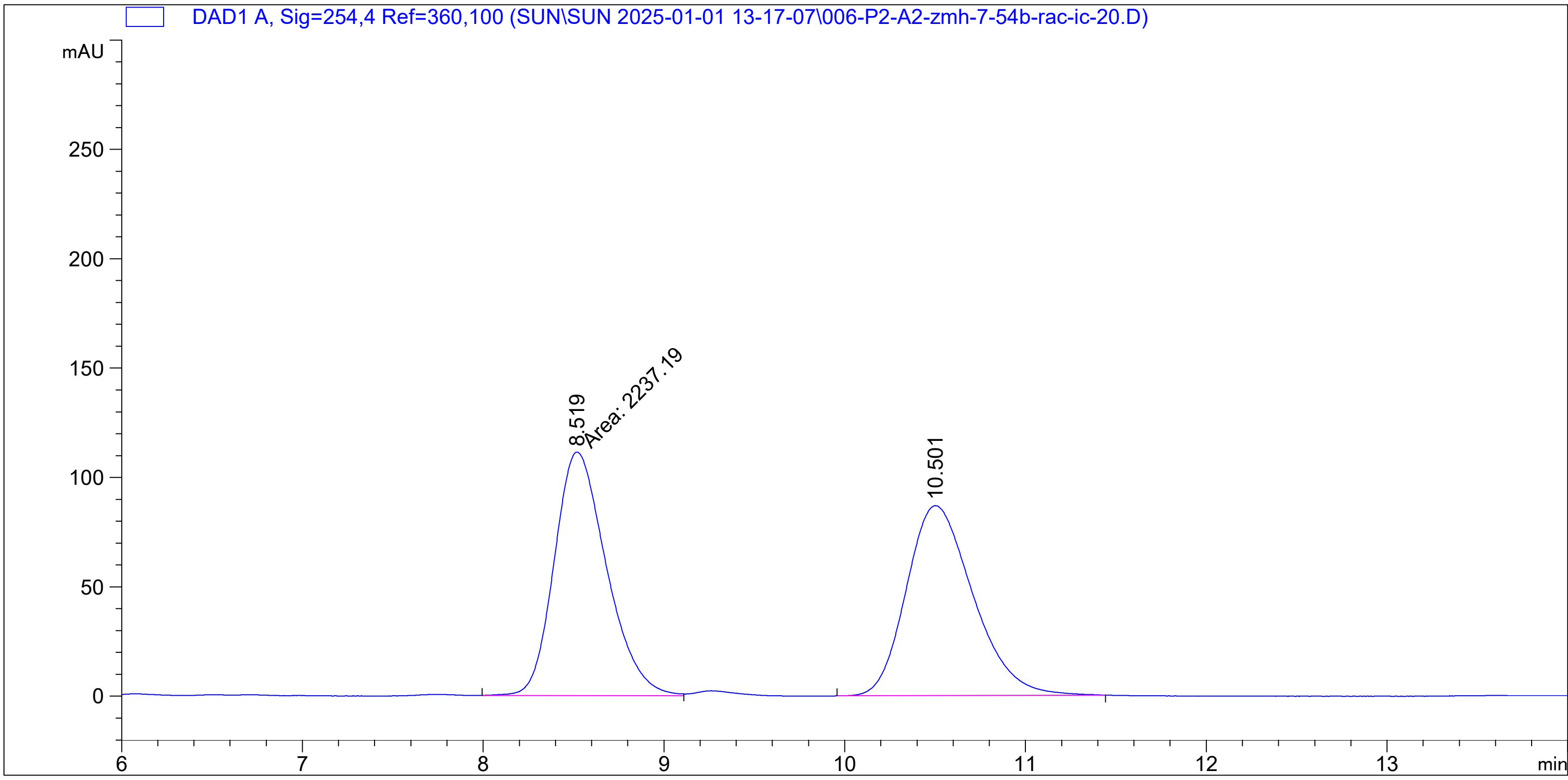

===

Area Percent Report

===

Sorted By : Signal
Multiplier : 1.0000
Dilution : 1.0000
Use Multiplier & Dilution Factor with ISTDs

Signal 1: DAD1 A, Sig=254,4 Ref=360,100

Peak #	RetTime [min]	Type	Width [min]	Area [mAU*s]	Height [mAU]	Area %
1	8.519	MF	0.3349	2237.18652	111.32880	50.1063
2	10.501	BB	0.3678	2227.69702	86.84698	49.8937

Totals : 4464.88354 198.17577

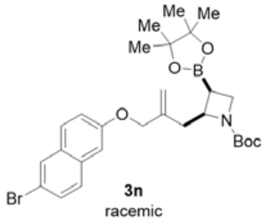

3n
racemic

Data File C:\Users\P...ion\1\Data\SUN\SUN 2025-01-01 13-17-07\088-P1-B1-zmh-7-24e-chiral.D
Sample Name: zmh-7-24e-chiral

===

Acq. Operator : SYSTEM   Seq. Line : 88
Sample Operator : SYSTEM
Acq. Instrument : HPLC   Location : P1-B-01
Injection Date : 2/1/2025 3:43:11 pm   Inj : 1
                                          Inj Volume : 2.000 µl
Different Inj Volume from Sample Entry! Actual Inj Volume : 10.000 µl
Acq. Method : C:\Users\Public\Documents\ChemStation\1\Data\SUN\SUN 2025-01-01 13-17-07\IC3-10-20.M
Last changed : 15/8/2022 10:26:28 pm by SYSTEM
Analysis Method : C:\Users\Public\Documents\ChemStation\1\Data\SUN\SUN 2025-01-01 13-17-07\IC3-10-20.M (Sequence Method)
Last changed : 8/5/2025 6:52:37 pm by SYSTEM
(modified after loading)
Additional Info : Peak(s) manually integrated

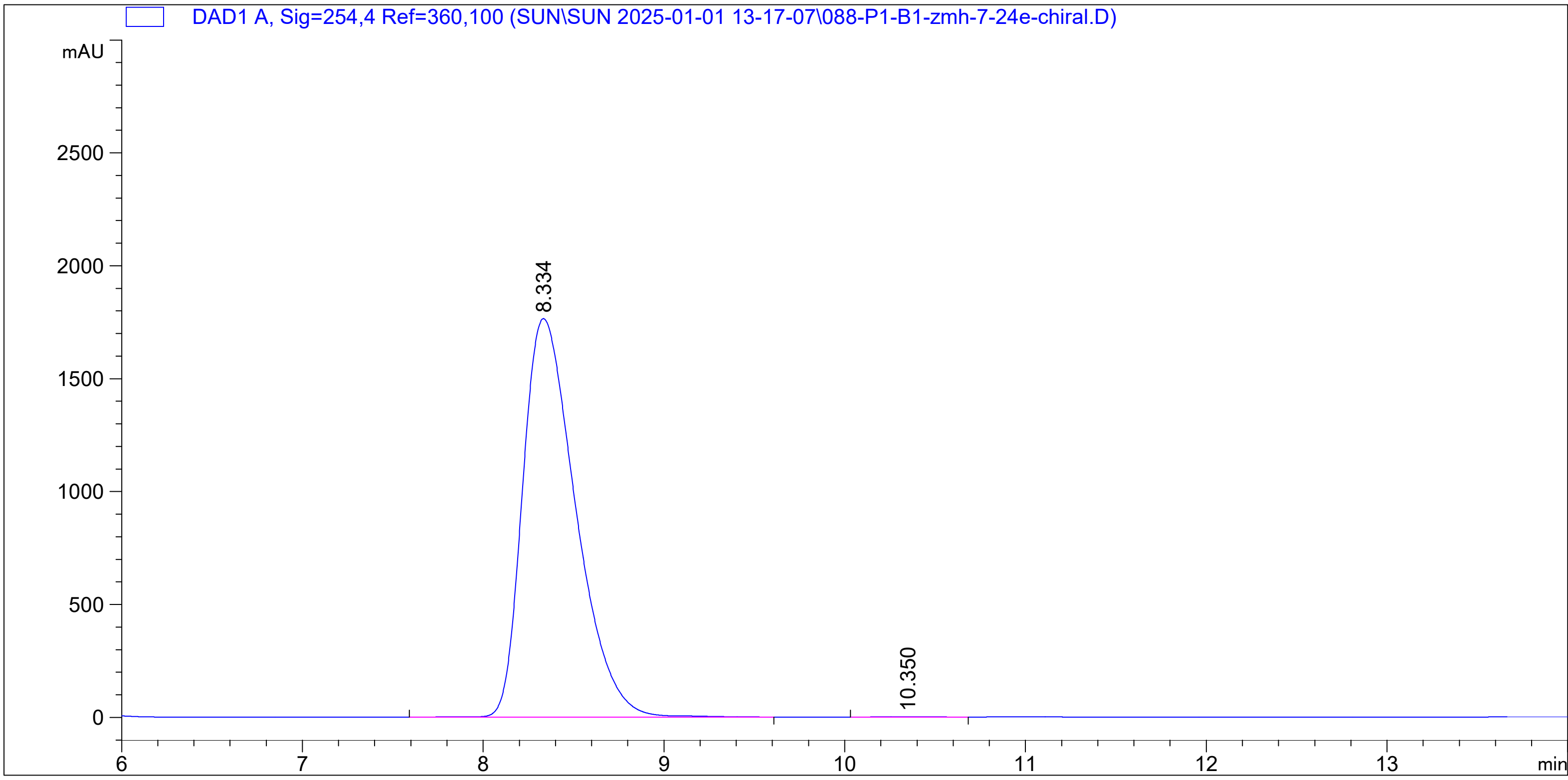

===
Area Percent Report
===

Sorted By : Signal
Multiplier : 1.0000
Dilution : 1.0000
Use Multiplier & Dilution Factor with ISTDs

Signal 1: DAD1 A, Sig=254,4 Ref=360,100

Peak #	RetTime [min]	Type	Width [min]	Area [mAU*s]	Height [mAU]	Area %
1	8.334	BB	0.2768	3.58982e4	1764.75305	99.9282
2	10.350	BB	0.2351	25.79789	1.30253	0.0718

Totals : 3.59240e4 1766.05558

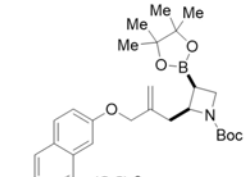

(*S*,*S*)-**3n**
enantioenriched

```
Data File C:\Users\P...ion\1\Data\SUN\SUN 2025-01-01 13-17-07\004-P2-A3-zmh-7-54c-rac-ic.D
Sample Name: zmh-7-54c-rac-ic

    =====================================================================
    Acq. Operator   : SYSTEM                         Seq. Line :    4
    Sample Operator : SYSTEM
    Acq. Instrument : HPLC                            Location :    P2-A-03
    Injection Date  : 1/1/2025 2:02:33 pm                  Inj :    1
                                                    Inj Volume : 2.000 µl
    Different Inj Volume from Sample Entry!  Actual Inj Volume : 10.000 µl
    Acq. Method     : C:\Users\Public\Documents\ChemStation\1\Data\SUN\SUN 2025-01-01 13-17-07\IC3-
                      10-20.M
    Last changed    : 15/8/2022 10:26:28 pm by SYSTEM
    Analysis Method : C:\Users\Public\Documents\ChemStation\1\Data\SUN\SUN 2025-01-01 13-17-07\IC3-
                      10-20.M (Sequence Method)
    Last changed    : 8/5/2025 6:57:03 pm by SYSTEM
                      (modified after loading)
    Additional Info : Peak(s) manually integrated
```

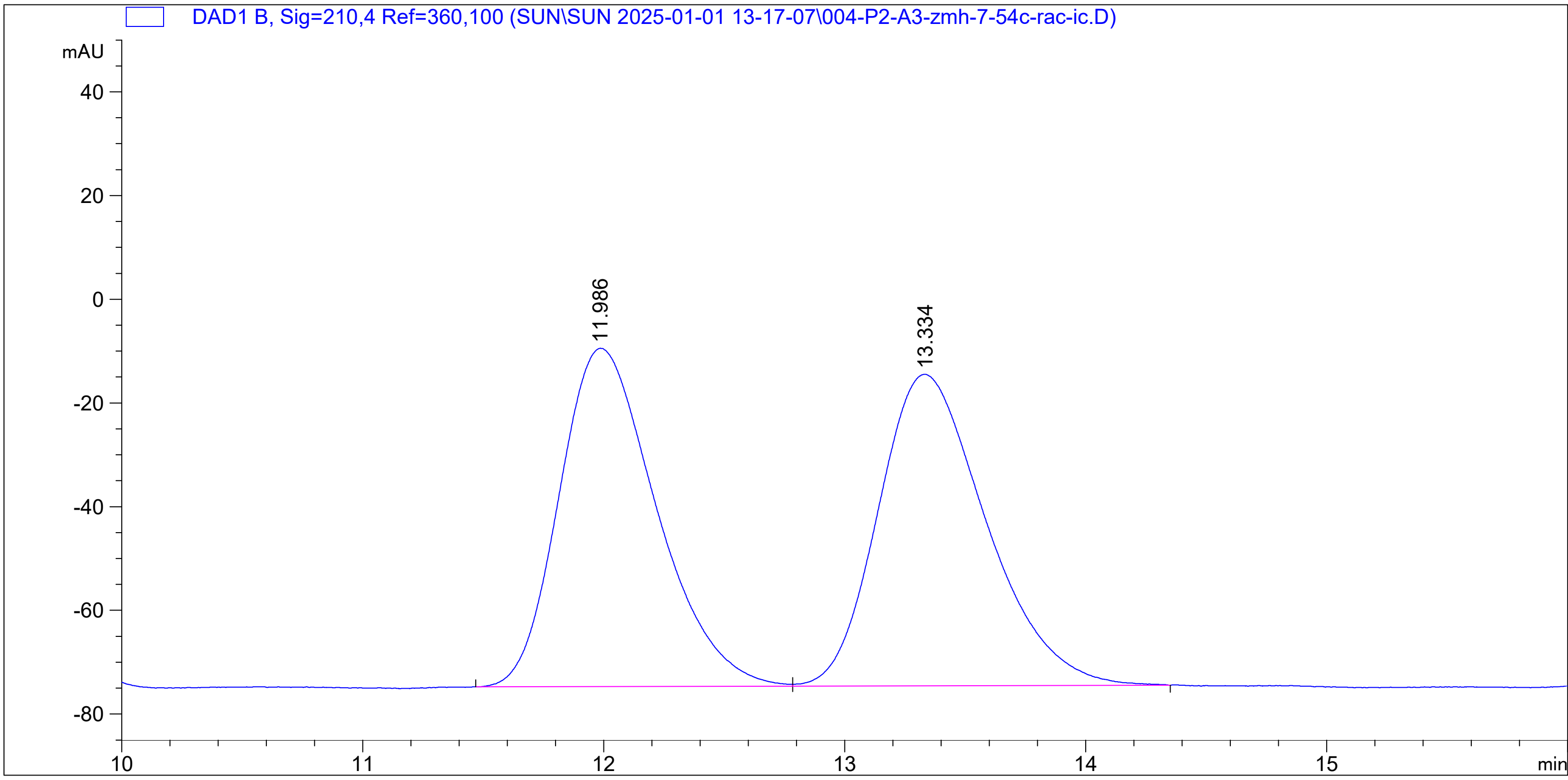


```
    =====================================================================
                             Area Percent Report
    =====================================================================

    Sorted By             :      Signal
    Multiplier            :      1.0000
    Dilution              :      1.0000
    Use Multiplier & Dilution Factor with ISTDs


    Signal 1: DAD1 B, Sig=210,4 Ref=360,100
```

Peak #	RetTime [min]	Type	Width [min]	Area [mAU*s]	Height [mAU]	Area %
1	11.986	BV	0.3475	1825.74866	65.24534	49.4637
2	13.334	VB	0.3798	1865.33948	60.06494	50.5363

Totals : 3691.08813 125.31028

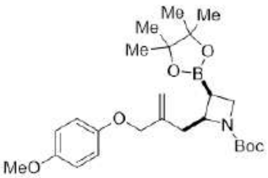

3o
racemic

===

Acq. Operator : SYSTEM Seq. Line : 90
Sample Operator : SYSTEM
Acq. Instrument : HPLC Location : P1-B-02
Injection Date : 2/1/2025 4:15:15 pm Inj : 1
Inj Volume : 2.000 µl
Different Inj Volume from Sample Entry! Actual Inj Volume : 10.000 µl
Acq. Method : C:\Users\Public\Documents\ChemStation\1\Data\SUN\SUN 2025-01-01 13-17-07\IC3-10-20.M
Last changed : 15/8/2022 10:26:28 pm by SYSTEM
Analysis Method : C:\Users\Public\Documents\ChemStation\1\Data\SUN\SUN 2025-01-01 13-17-07\IC3-10-20.M (Sequence Method)
Last changed : 8/5/2025 6:59:08 pm by SYSTEM
(modified after loading)
Additional Info : Peak(s) manually integrated

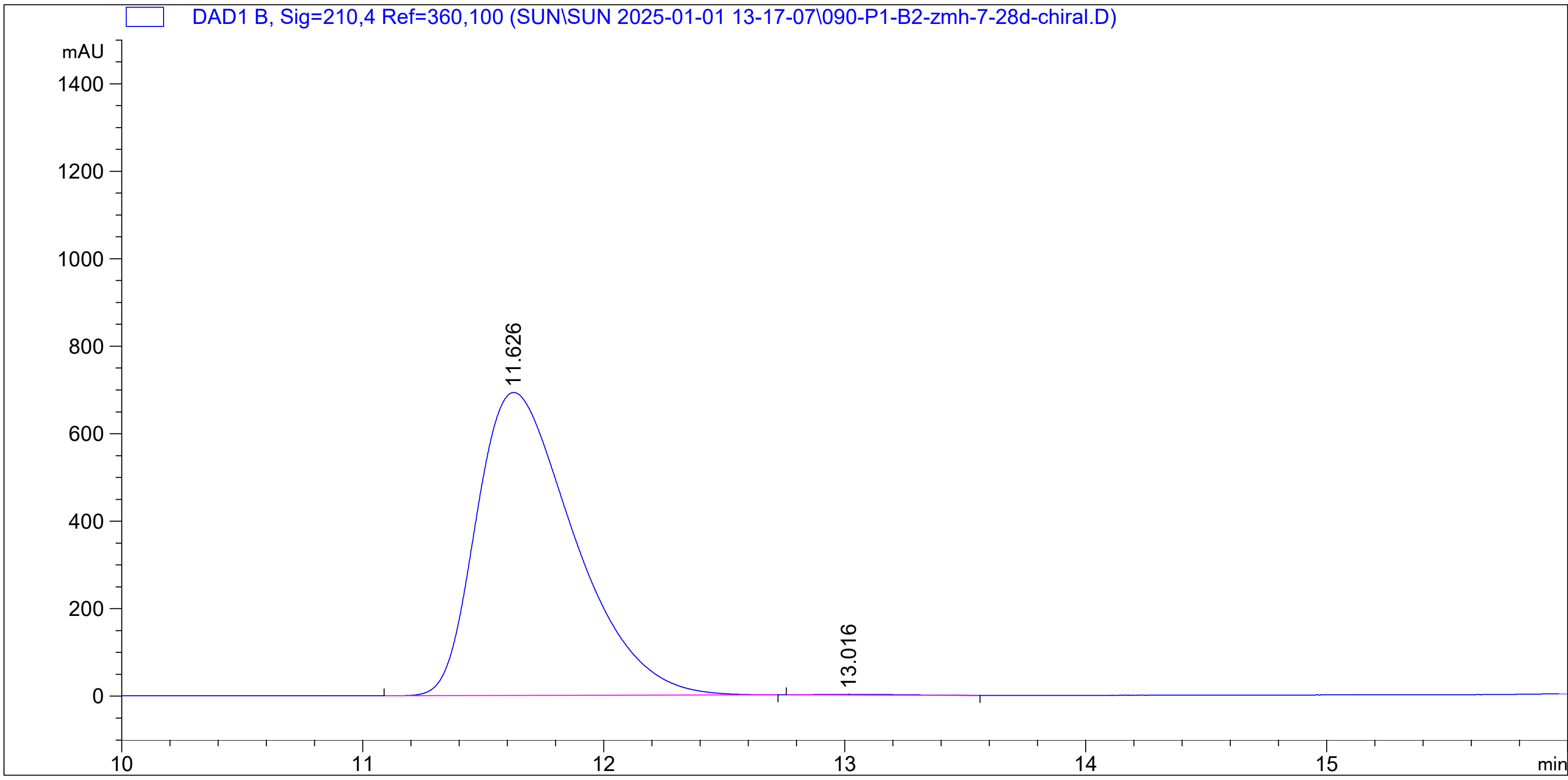

===

Area Percent Report

===

Sorted By : Signal
Multiplier : 1.0000
Dilution : 1.0000
Use Multiplier & Dilution Factor with ISTDs

Signal 1: DAD1 B, Sig=210,4 Ref=360,100

Peak #	RetTime [min]	Type	Width [min]	Area [mAU*s]	Height [mAU]	Area %
1	11.626	BB	0.4156	1.94777e4	692.68976	99.7970
2	13.016	BB	0.2824	39.61664	1.66013	0.2030

Totals : 1.95173e4 694.34989

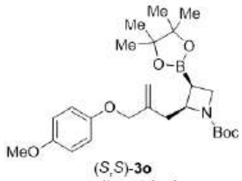

(*S*,*S*)-**3o**
enantioenriched

===

Acq. Operator : SYSTEM   Seq. Line : 12
Sample Operator : SYSTEM
Acq. Instrument : HPLC   Location : P2-C-01
Injection Date : 22/10/2024 8:28:46 pm   Inj : 1
Inj Volume : 2.000 µl
Different Inj Volume from Sample Entry! Actual Inj Volume : 10.000 µl
Acq. Method : C:\Users\Public\Documents\ChemStation\1\Data\SUN\SUN 2024-10-22 17-00-49\IC3-10-20.M
Last changed : 15/8/2022 10:26:28 pm by SYSTEM
Analysis Method : C:\Users\Public\Documents\ChemStation\1\Data\SUN\SUN 2024-10-22 17-00-49\IC3-10-20.M (Sequence Method)
Last changed : 8/5/2025 7:12:30 pm by SYSTEM
(modified after loading)
Additional Info : Peak(s) manually integrated

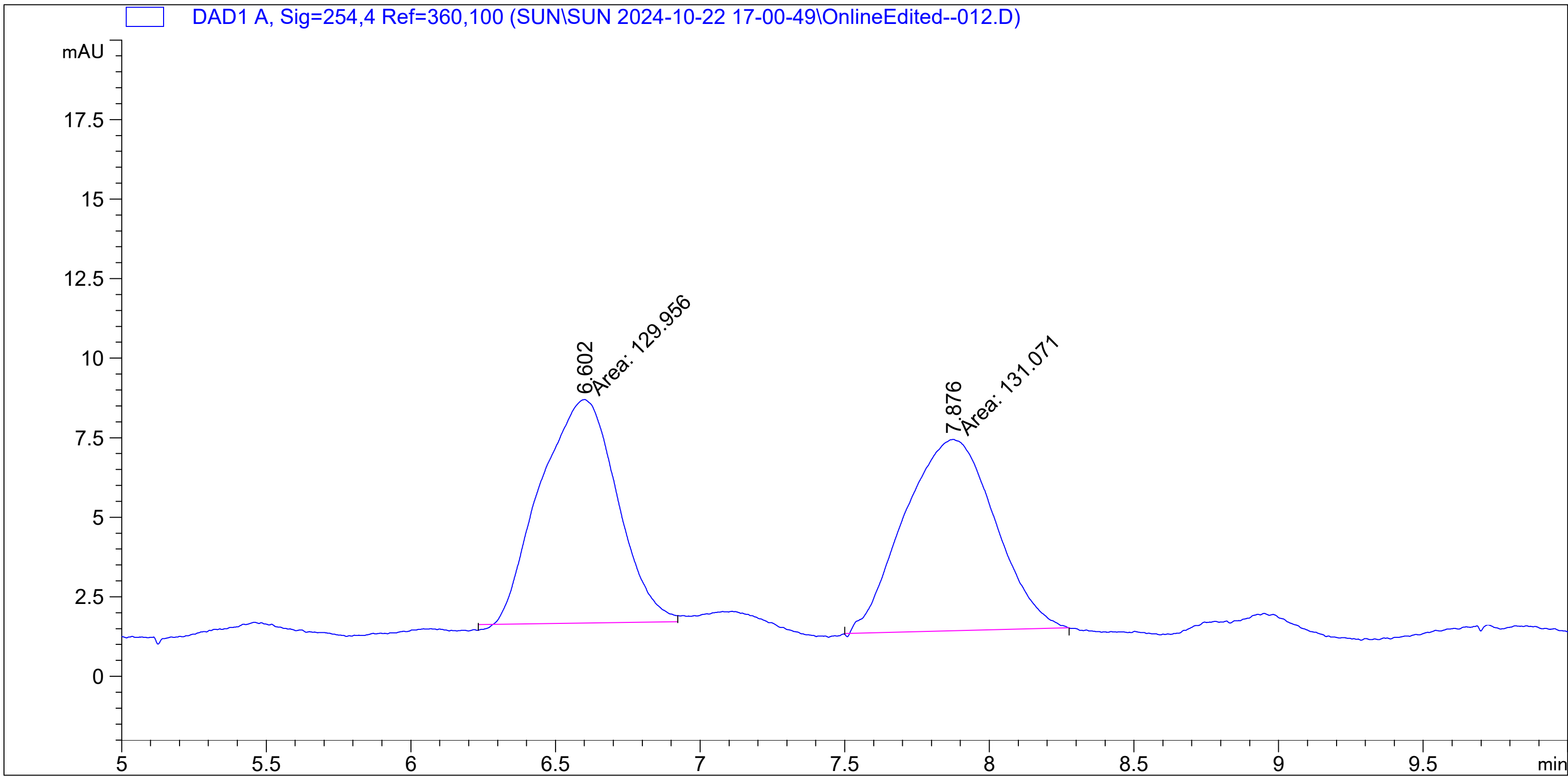

===
Area Percent Report
===

Sorted By : Signal
Multiplier : 1.0000
Dilution : 1.0000
Use Multiplier & Dilution Factor with ISTDs

Signal 1: DAD1 A, Sig=254,4 Ref=360,100

Peak #	RetTime [min]	Type	Width [min]	Area [mAU*s]	Height [mAU]	Area %
1	6.602	MM	0.3081	129.95566	7.02996	49.7864
2	7.876	MM	0.3633	131.07062	6.01315	50.2136

Totals : 261.02628 13.04312

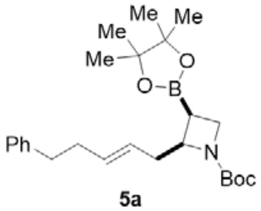

5a
racemic

Data File C:\Users\P...\ChemStation\1\Data\SUN\SUN 2024-10-22 17-00-49\OnlineEdited--016.D
Sample Name: zmh-6-157-chiral

===

```
Acq. Operator   : SYSTEM                         Seq. Line :  16
Sample Operator : SYSTEM
Acq. Instrument : HPLC                            Location :   P1-A-01
Injection Date  : 22/10/2024 9:41:26 pm                Inj :   1
                                                Inj Volume : 2.000 µl
Different Inj Volume from Sample Entry!  Actual Inj Volume : 10.000 µl
Acq. Method     : C:\Users\Public\Documents\ChemStation\1\Data\SUN\SUN 2024-10-22 17-00-49\IC3-
                  10-20.M
Last changed    : 15/8/2022 10:26:28 pm by SYSTEM
Analysis Method : C:\Users\Public\Documents\ChemStation\1\Data\SUN\SUN 2024-10-22 17-00-49\IC3-
                  10-20.M (Sequence Method)
Last changed    : 8/5/2025 7:06:59 pm by SYSTEM
                  (modified after loading)
Additional Info : Peak(s) manually integrated
```

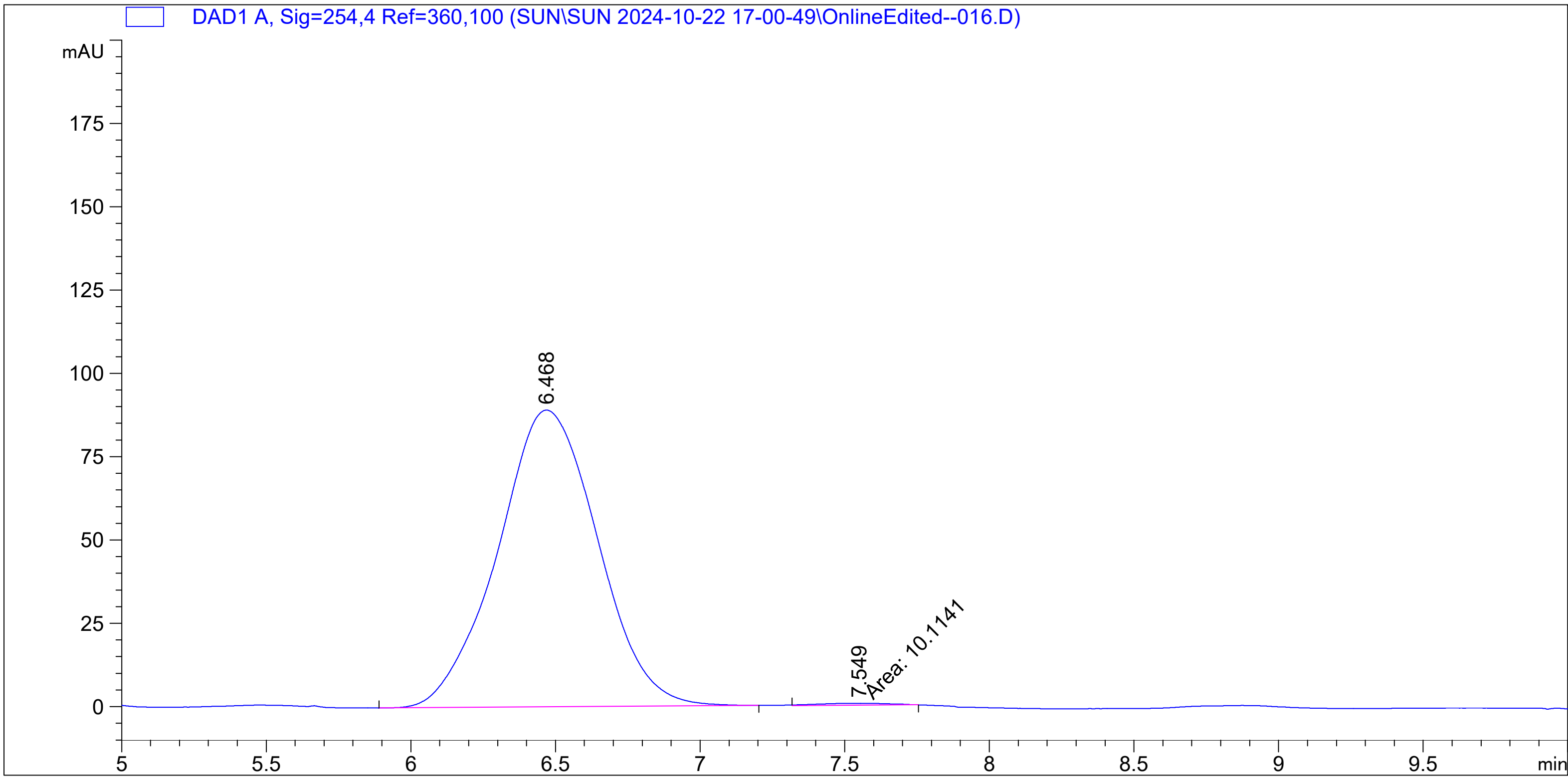

===

Area Percent Report

===

```
Sorted By             :      Signal
Multiplier            :      1.0000
Dilution              :      1.0000
Use Multiplier & Dilution Factor with ISTDs
```

Signal 1: DAD1 A, Sig=254,4 Ref=360,100

Peak #	RetTime [min]	Type	Width [min]	Area [mAU*s]	Height [mAU]	Area %
1	6.468	BB	0.3618	2138.83789	89.03377	99.5293
2	7.549	MM	0.2959	10.11411	5.69657e-1	0.4707

Totals : 2148.95200 89.60342

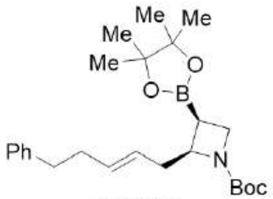

(*S*,*S*)-**5a**
enantioenriched

Data File C:\Users\P...ion\1\Data\SUN\SUN 2024-12-29 22-31-18\012-P1-F1-zmh-7-53a-rac-ic.D
Sample Name: zmh-7-53a-rac-ic

===

Acq. Operator : SYSTEM Seq. Line : 12
Sample Operator : SYSTEM
Acq. Instrument : HPLC Location : P1-F-01
Injection Date : 30/12/2024 1:57:01 am Inj : 1
Inj Volume : 2.000 µl
Different Inj Volume from Sample Entry! Actual Inj Volume : 20.000 µl
Acq. Method : C:\Users\Public\Documents\ChemStation\1\Data\SUN\SUN 2024-12-29 22-31-18\IC3-10-20.M
Last changed : 15/8/2022 10:26:28 pm by SYSTEM
Analysis Method : C:\Users\Public\Documents\ChemStation\1\Data\SUN\SUN 2024-12-29 22-31-18\IC3-10-20.M (Sequence Method)
Last changed : 9/6/2025 12:54:22 pm by SYSTEM
(modified after loading)
Additional Info : Peak(s) manually integrated

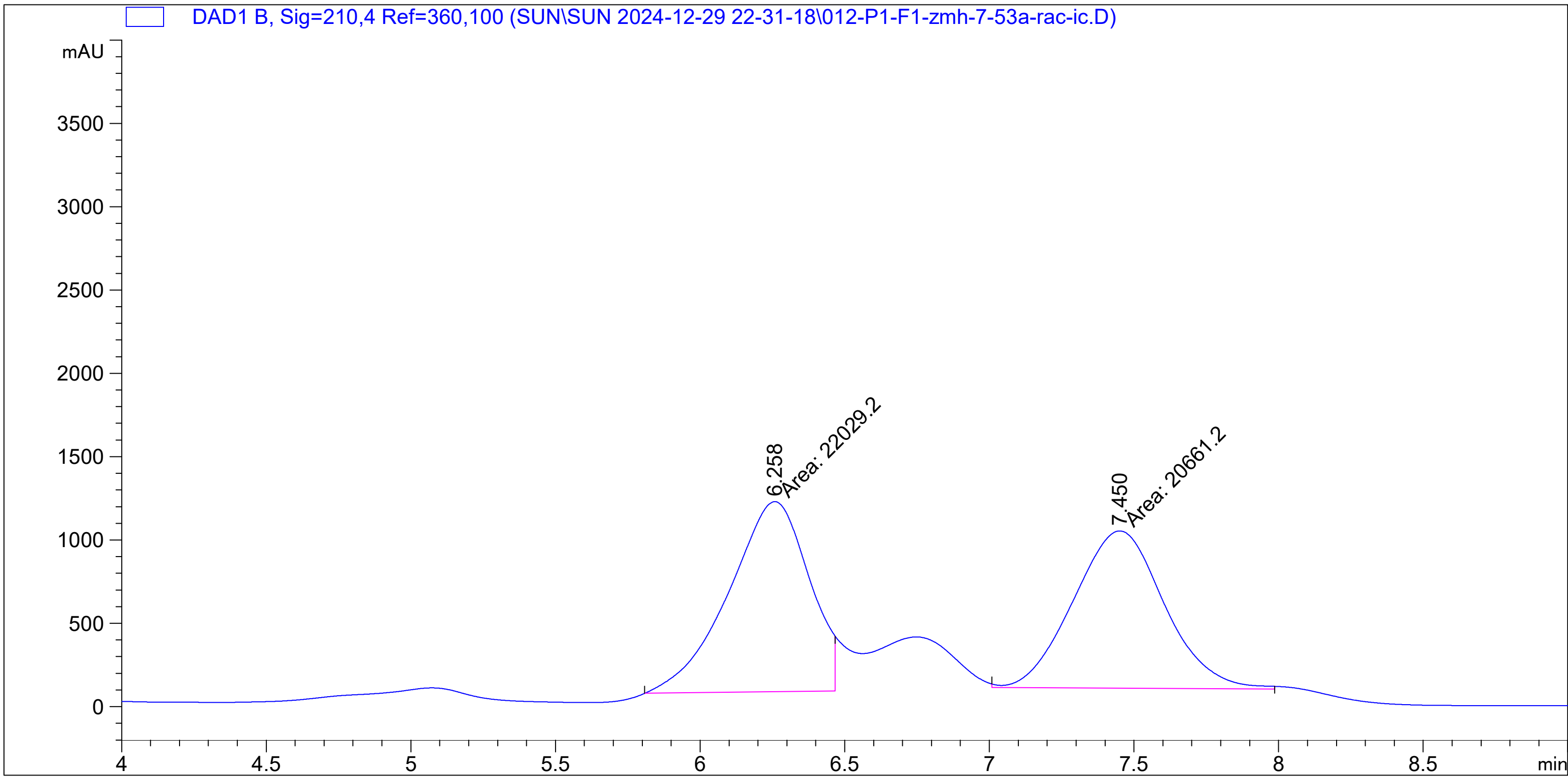

===

Area Percent Report

===

Sorted By : Signal
Multiplier : 1.0000
Dilution : 1.0000
Use Multiplier & Dilution Factor with ISTDs

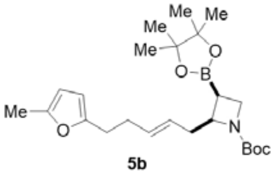

5b
racemic

Signal 1: DAD1 B, Sig=210,4 Ref=360,100

Peak #	RetTime [min]	Type	Width [min]	Area [mAU*s]	Height [mAU]	Area %
1	6.258	MF	0.3221	2.20292e4	1139.95508	51.6022
2	7.450	MM	0.3652	2.06612e4	942.88745	48.3978

Totals : 4.26904e4 2082.84253

Data File C:\Users\P...ion\1\Data\SUN\SUN 2024-12-31 10-21-01\002-P1-A1-zmh-7-24a-chiral.D
Sample Name: zmh-7-24a-chiral

===

Acq. Operator : SYSTEM Seq. Line : 2
Sample Operator : SYSTEM
Acq. Instrument : HPLC Location : P1-A-01
Injection Date : 31/12/2024 10:34:18 am Inj : 1
Inj Volume : 2.000 µl
Different Inj Volume from Sample Entry! Actual Inj Volume : 20.000 µl
Acq. Method : C:\Users\Public\Documents\ChemStation\1\Data\SUN\SUN 2024-12-31 10-21-01\IC3-10-20.M
Last changed : 15/8/2022 10:26:28 pm by SYSTEM
Analysis Method : C:\Users\Public\Documents\ChemStation\1\Data\SUN\SUN 2024-12-31 10-21-01\IC3-10-20.M (Sequence Method)
Last changed : 8/5/2025 7:26:56 pm by SYSTEM
(modified after loading)
Additional Info : Peak(s) manually integrated

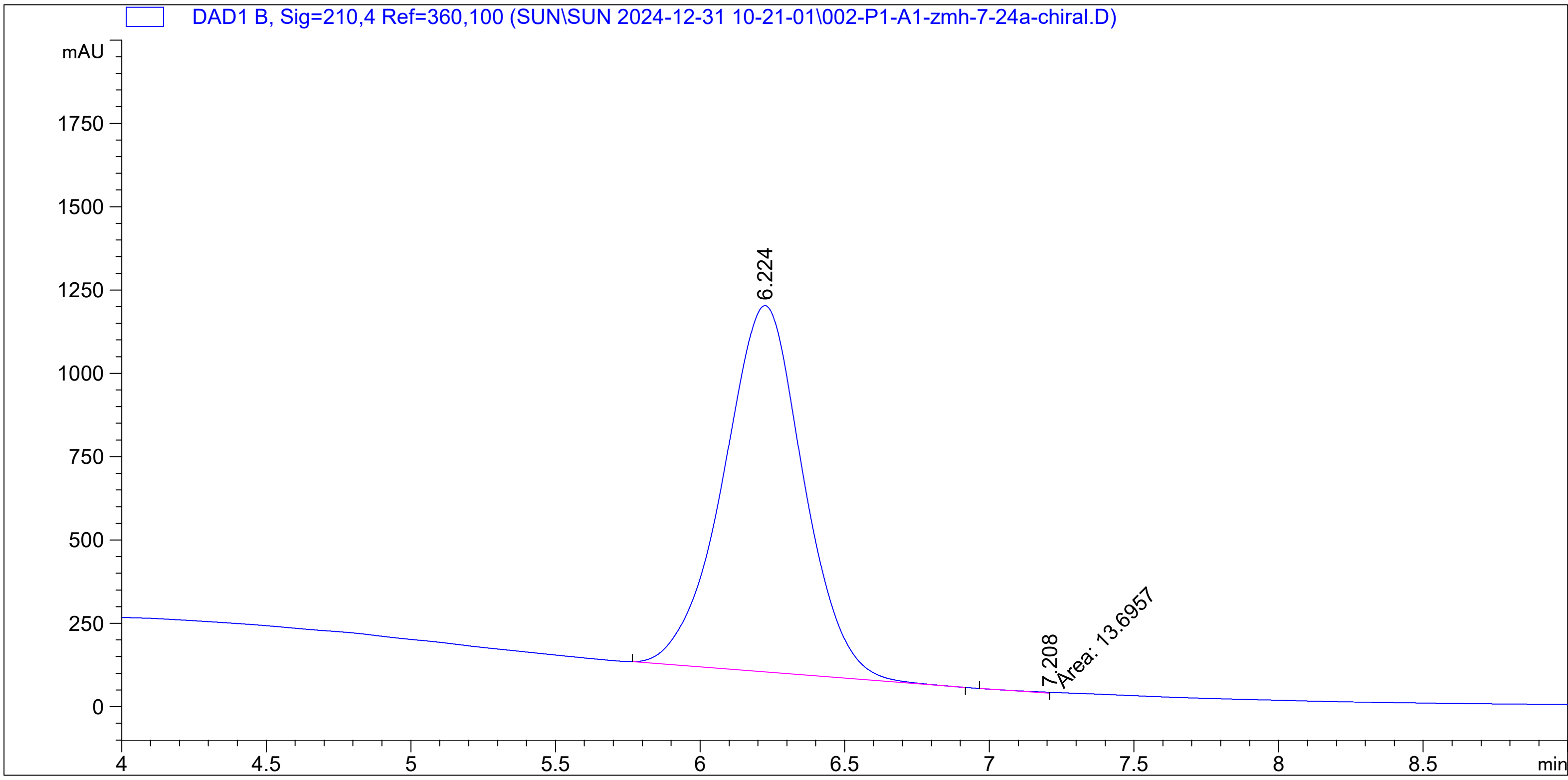

===

Area Percent Report

===

Sorted By : Signal
Multiplier : 1.0000
Dilution : 1.0000
Use Multiplier & Dilution Factor with ISTDs

Signal 1: DAD1 B, Sig=210,4 Ref=360,100

Peak #	RetTime [min]	Type	Width [min]	Area [mAU*s]	Height [mAU]	Area %
1	6.224	BB	0.2851	2.11564e4	1098.84961	99.9353
2	7.208	MM	0.0825	13.69575	2.76568	0.0647

Totals : 2.11701e4 1101.61529

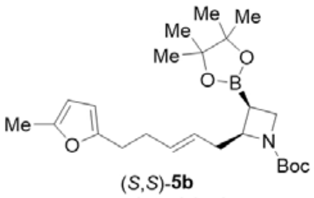

(*S*,*S*)-**5b**
enantioenriched

Data File C:\Users\P...tion\1\Data\SUN\SUN 2024-12-31 10-21-01\024-P1-D1-zmh-7-2c-rac-ic.D
Sample Name: zmh-7-2c-rac-ic

===

Acq. Operator : SYSTEM Seq. Line : 24
Sample Operator : SYSTEM
Acq. Instrument : HPLC Location : P1-D-01
Injection Date : 31/12/2024 4:30:10 pm Inj : 1
Inj Volume : 2.000 µl
Different Inj Volume from Sample Entry! Actual Inj Volume : 20.000 µl
Acq. Method : C:\Users\Public\Documents\ChemStation\1\Data\SUN\SUN 2024-12-31 10-21-01\IC3-10-20.M
Last changed : 15/8/2022 10:26:28 pm by SYSTEM
Analysis Method : C:\Users\Public\Documents\ChemStation\1\Data\SUN\SUN 2024-12-31 10-21-01\IC3-10-20.M (Sequence Method)
Last changed : 3/6/2025 4:10:42 pm by SYSTEM
(modified after loading)
Additional Info : Peak(s) manually integrated

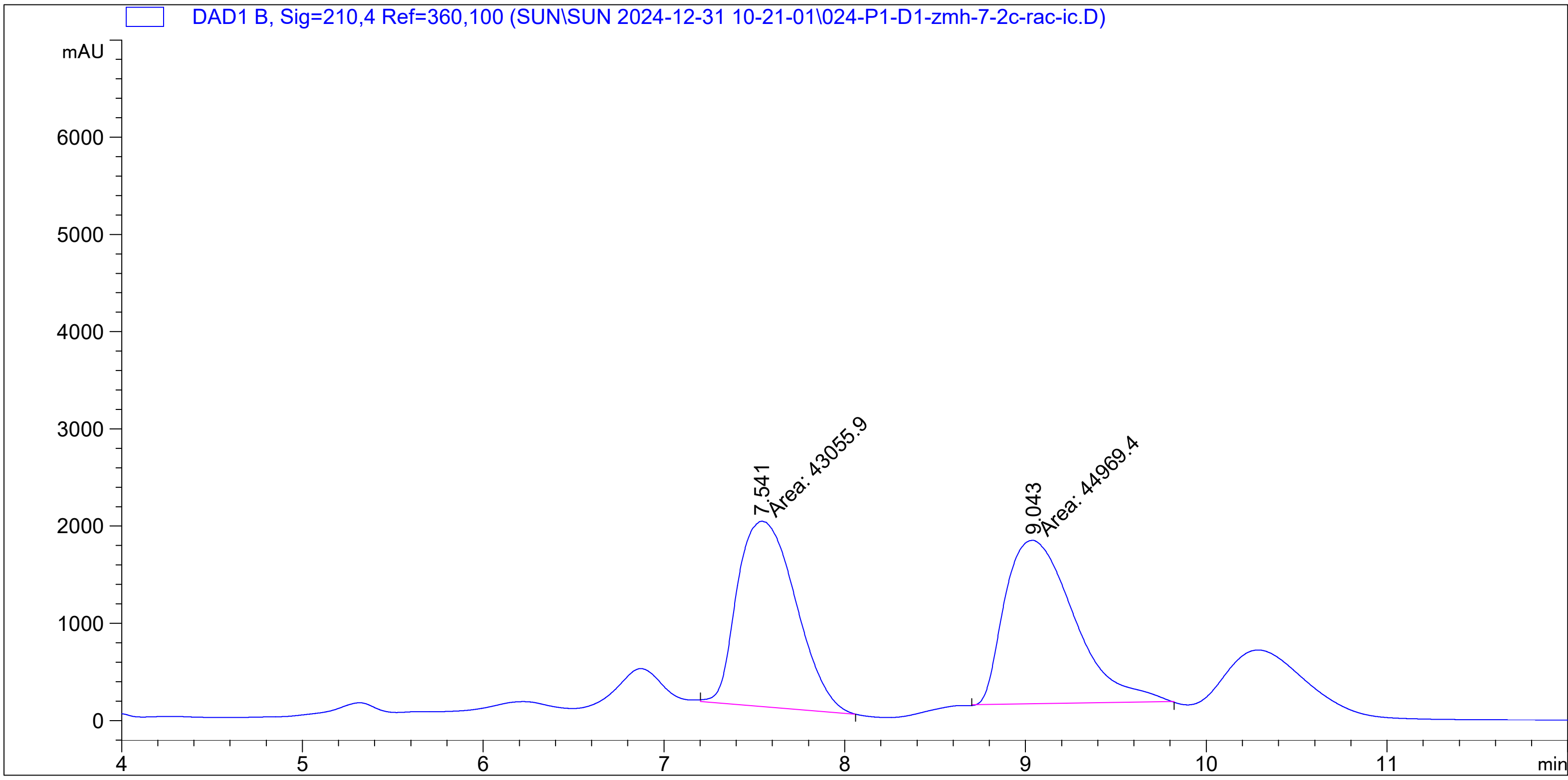

===

Area Percent Report

===

Sorted By : Signal
Multiplier : 1.0000
Dilution : 1.0000
Use Multiplier & Dilution Factor with ISTDs

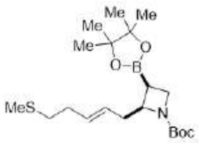

5c
racemic

Signal 1: DAD1 B, Sig=210,4 Ref=360,100

Peak #	RetTime [min]	Type	Width [min]	Area [mAU*s]	Height [mAU]	Area %
1	7.541	MM	0.3761	4.30559e4	1908.17847	48.9131
2	9.043	MM	0.4461	4.49694e4	1680.02087	51.0869

Totals : 8.80253e4 3588.19934

Data File C:\Users\P...ion\1\Data\SUN\SUN 2024-12-31 10-21-01\036-P1-D3-zmh-7-27a-chiral.D
Sample Name: zmh-7-27a-chiral

===

Acq. Operator : SYSTEM   Seq. Line : 36
Sample Operator : SYSTEM
Acq. Instrument : HPLC   Location : P1-D-03
Injection Date : 31/12/2024 7:39:40 pm   Inj : 1
Inj Volume : 2.000 µl
Different Inj Volume from Sample Entry! Actual Inj Volume : 20.000 µl
Acq. Method : C:\Users\Public\Documents\ChemStation\1\Data\SUN\SUN 2024-12-31 10-21-01\IC3-10-20.M
Last changed : 15/8/2022 10:26:28 pm by SYSTEM
Analysis Method : C:\Users\Public\Documents\ChemStation\1\Data\SUN\SUN 2024-12-31 10-21-01\IC3-10-20.M (Sequence Method)
Last changed : 8/5/2025 7:31:00 pm by SYSTEM
(modified after loading)
Additional Info : Peak(s) manually integrated

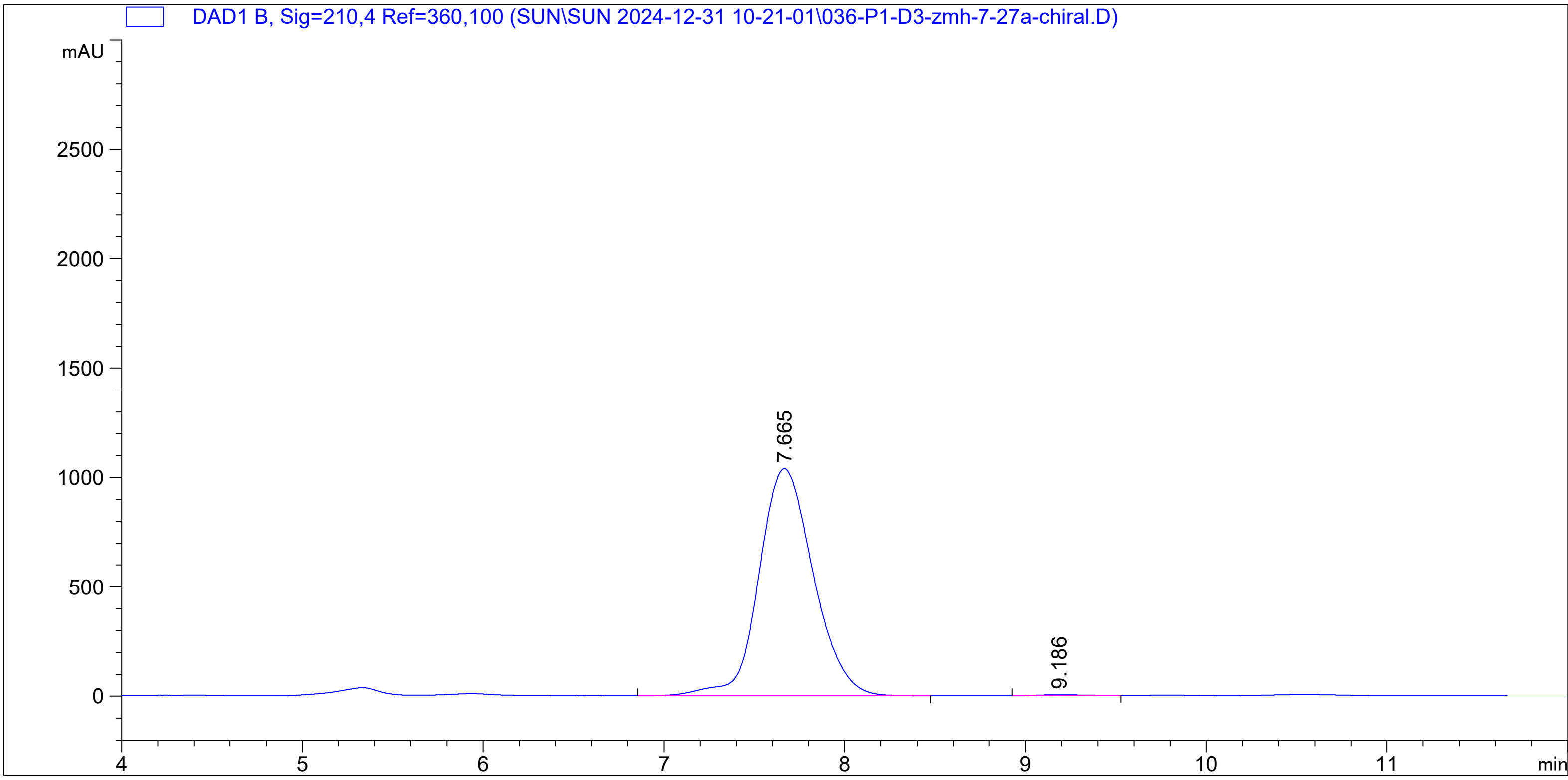

===
Area Percent Report
===

Sorted By : Signal
Multiplier : 1.0000
Dilution : 1.0000
Use Multiplier & Dilution Factor with ISTDs

Signal 1: DAD1 B, Sig=210,4 Ref=360,100

Peak #	RetTime [min]	Type	Width [min]	Area [mAU*s]	Height [mAU]	Area %
1	7.665	BB	0.3195	2.18479e4	1038.32361	99.6802
2	9.186	BB	0.1934	70.08345	4.29429	0.3198

Totals : 2.19180e4 1042.61790

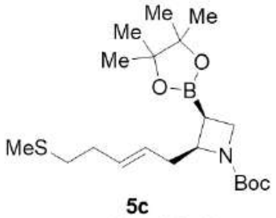

5c
enantioenriched

Data File C:\Users\P...ion\1\Data\SUN\SUN 2024-12-30 19-20-16\030-P1-F2-zmh-7-53b-rac-ie.D
Sample Name: zmh-7-53b-rac-ie

===

Acq. Operator : SYSTEM Seq. Line : 30
Sample Operator : SYSTEM
Acq. Instrument : HPLC Location : P1-F-02
Injection Date : 31/12/2024 3:15:11 am Inj : 1
Inj Volume : 2.000 µl
Different Inj Volume from Sample Entry! Actual Inj Volume : 10.000 µl
Acq. Method : C:\Users\Public\Documents\ChemStation\1\Data\SUN\SUN 2024-12-30 19-20-16\IE3-10-20.M
Last changed : 15/8/2022 10:27:27 pm by SYSTEM
Analysis Method : C:\Users\Public\Documents\ChemStation\1\Data\SUN\SUN 2024-12-30 19-20-16\IE3-10-20.M (Sequence Method)
Last changed : 8/5/2025 7:36:47 pm by SYSTEM
(modified after loading)
Additional Info : Peak(s) manually integrated

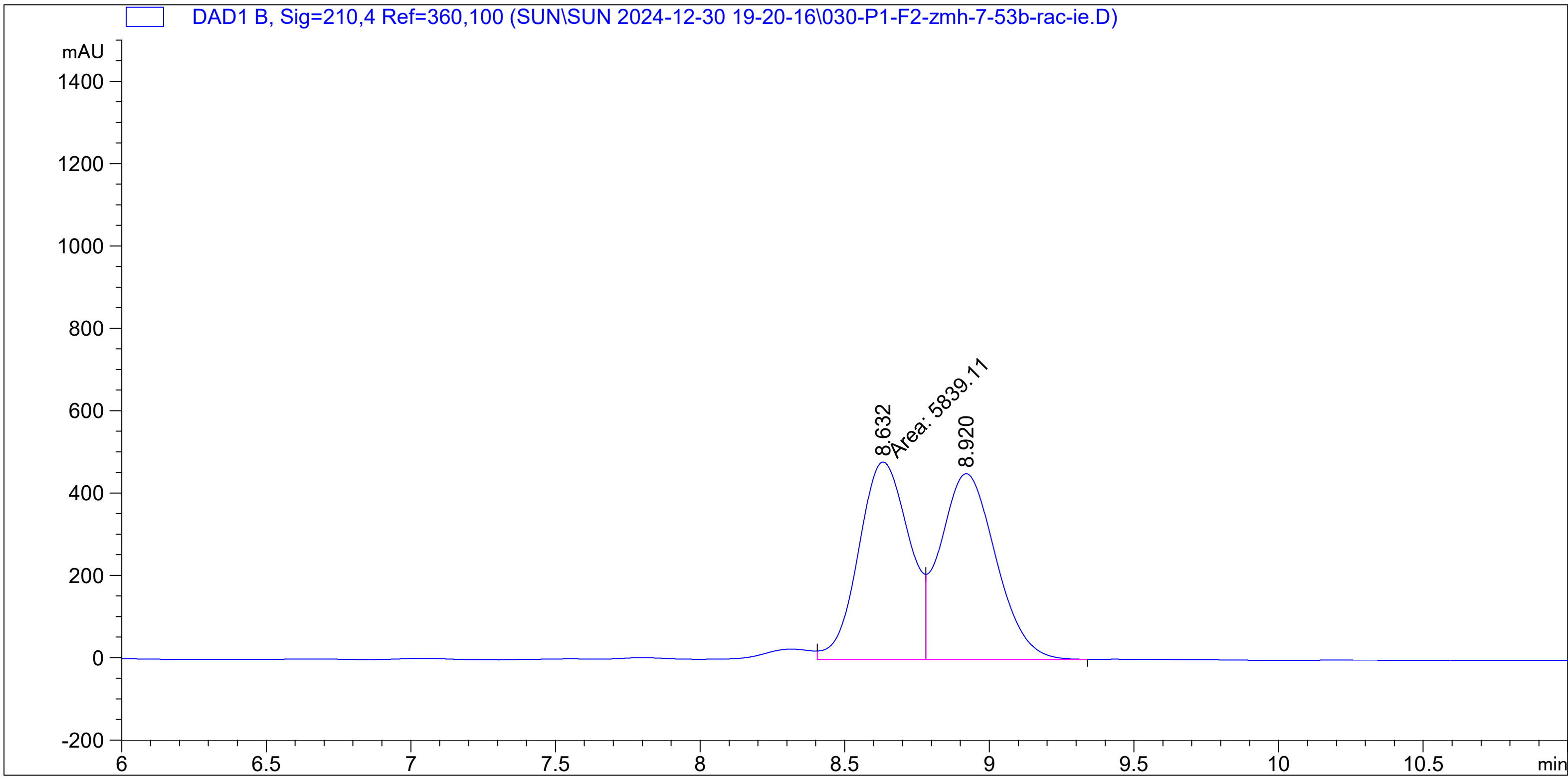

===

Area Percent Report

===

Sorted By : Signal
Multiplier : 1.0000
Dilution : 1.0000
Use Multiplier & Dilution Factor with ISTDs

Signal 1: DAD1 B, Sig=210,4 Ref=360,100

Peak #	RetTime [min]	Type	Width [min]	Area [mAU*s]	Height [mAU]	Area %
1	8.632	FM	0.2031	5839.10742	479.05380	49.2384
2	8.920	VB	0.2023	6019.73047	450.81479	50.7616

Totals : 1.18588e4 929.86859

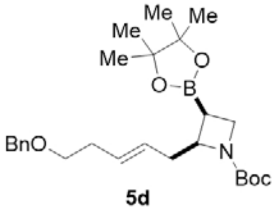

5d
racemic

Data File C:\Users\P...ion\1\Data\SUN\SUN 2024-12-31 10-21-01\008-P1-A4-zmh-7-27b-chiral.D
Sample Name: zmh-7-27b-chiral

===

Acq. Operator : SYSTEM   Seq. Line : 8
Sample Operator : SYSTEM
Acq. Instrument : HPLC   Location : P1-A-04
Injection Date : 31/12/2024 12:11:14 pm   Inj : 1
Inj Volume : 2.000 µl
Different Inj Volume from Sample Entry! Actual Inj Volume : 20.000 µl
Acq. Method : C:\Users\Public\Documents\ChemStation\1\Data\SUN\SUN 2024-12-31 10-21-01\IE3-10-20.M
Last changed : 15/8/2022 10:27:27 pm by SYSTEM
Analysis Method : C:\Users\Public\Documents\ChemStation\1\Data\SUN\SUN 2024-12-31 10-21-01\IE3-10-20.M (Sequence Method)
Last changed : 8/5/2025 7:44:14 pm by SYSTEM
(modified after loading)
Additional Info : Peak(s) manually integrated

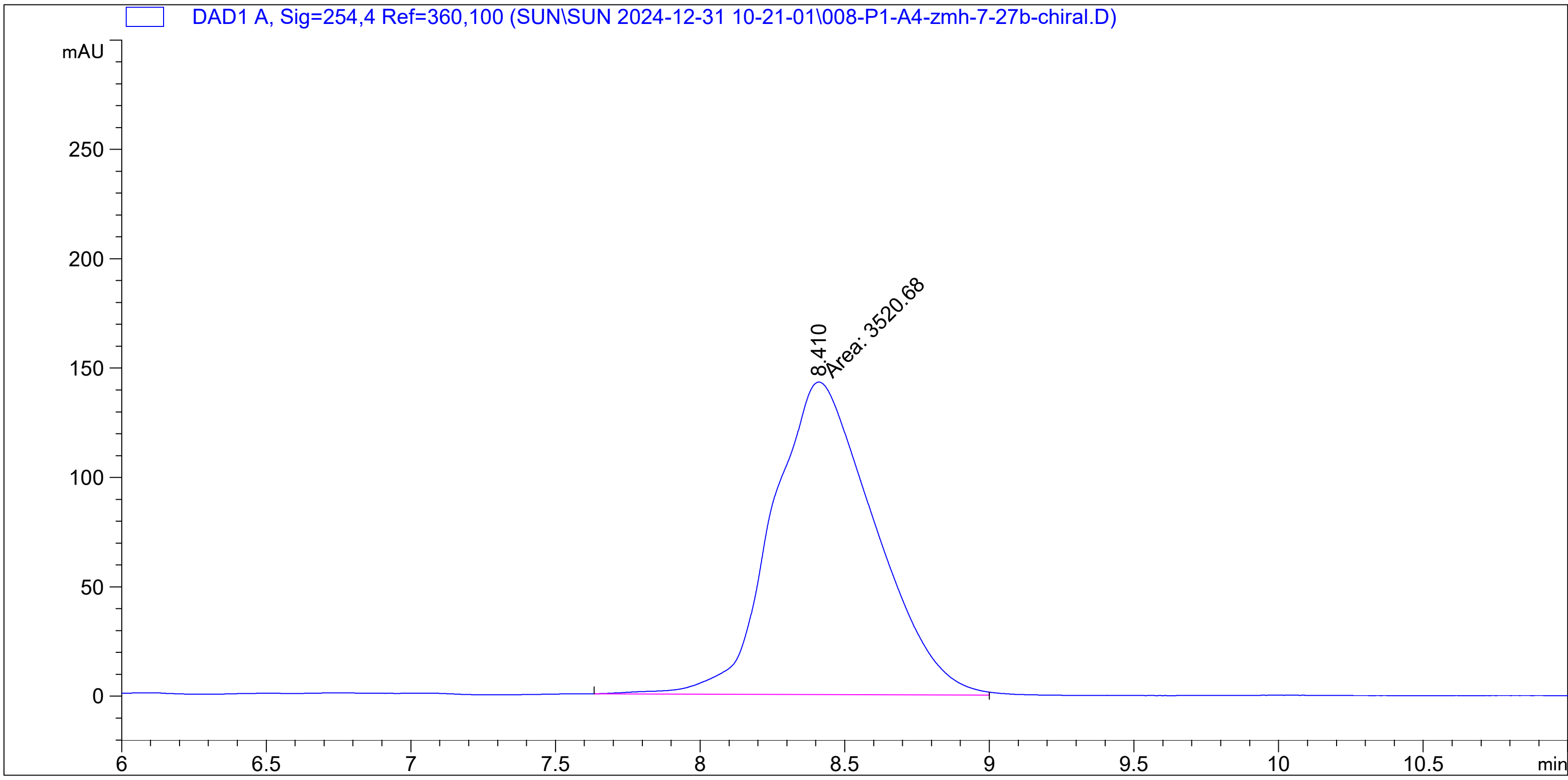

===

Area Percent Report

===

Sorted By : Signal
Multiplier : 1.0000
Dilution : 1.0000
Use Multiplier & Dilution Factor with ISTDs

Signal 1: DAD1 A, Sig=254,4 Ref=360,100

Peak #	RetTime [min]	Type	Width [min]	Area [mAU*s]	Height [mAU]	Area %
1	8.410	MF	0.4107	3520.68091	142.86226	100.0000

Totals : 3520.68091 142.86226

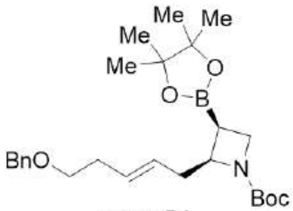

(*S*,*S*)-**5d**
enantioenriched

Data File C:\Users\P...tion\1\Data\SUN\SUN 2025-01-01 13-17-07\117-P1-D1-zmh-7-2a-rac-ic.D
Sample Name: zmh-7-2a-rac-ic

===

Acq. Operator : SYSTEM   Seq. Line : 117
Sample Operator : SYSTEM
Acq. Instrument : HPLC   Location : P1-D-01
Injection Date : 3/1/2025 12:33:57 am   Inj : 1
                Inj Volume : 2.000 µl
Different Inj Volume from Sample Entry! Actual Inj Volume : 20.000 µl
Acq. Method : C:\Users\Public\Documents\ChemStation\1\Data\SUN\SUN 2025-01-01 13-17-07\IC3-05-30.M
Last changed : 18/4/2023 10:37:59 pm by SYSTEM
Analysis Method : C:\Users\Public\Documents\ChemStation\1\Data\SUN\SUN 2025-01-01 13-17-07\IC3-05-30.M (Sequence Method)
Last changed : 8/5/2025 7:47:52 pm by SYSTEM
(modified after loading)
Additional Info : Peak(s) manually integrated

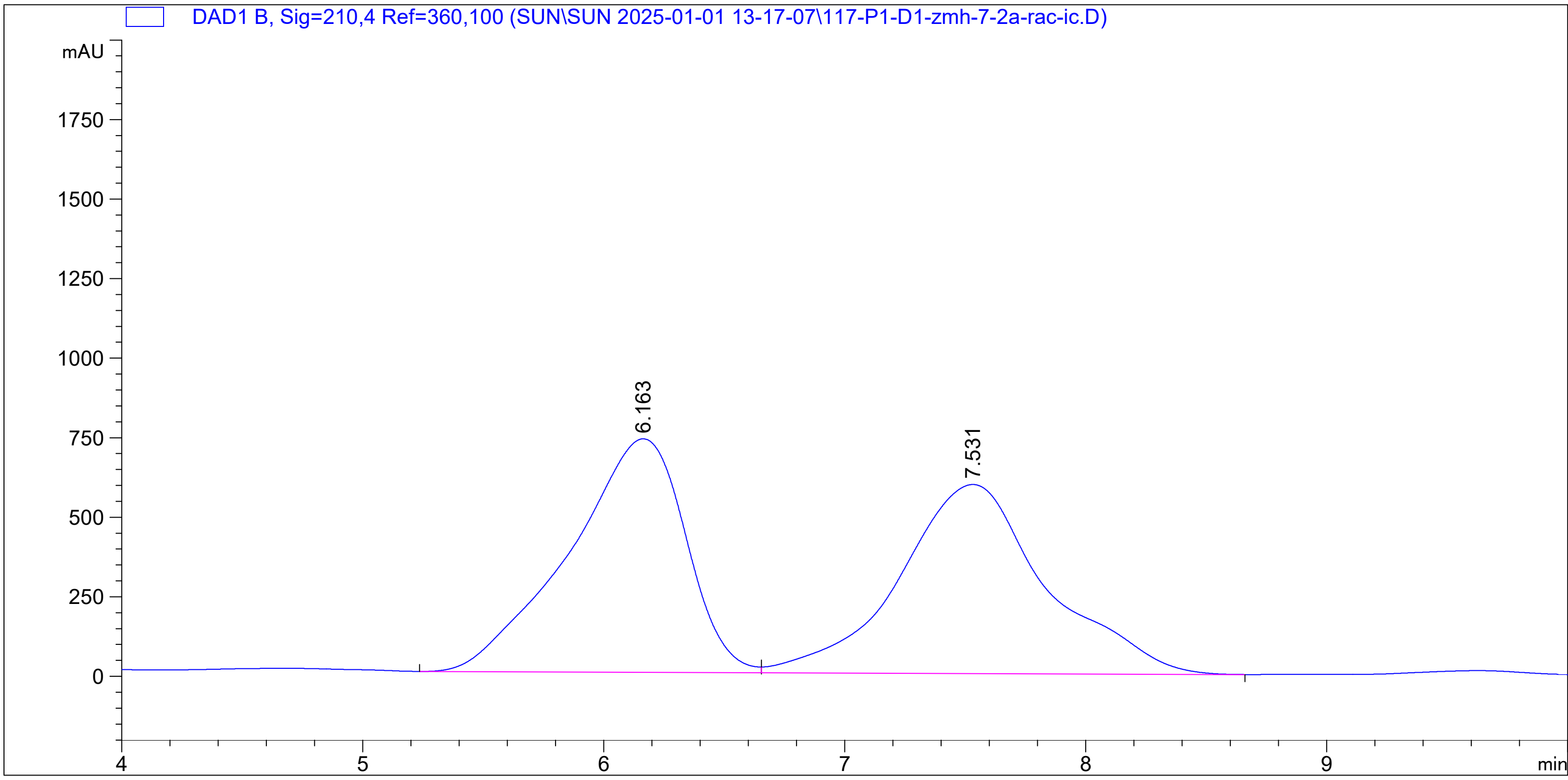

===

Area Percent Report

===

Sorted By : Signal
Multiplier : 1.0000
Dilution : 1.0000
Use Multiplier & Dilution Factor with ISTDs

Signal 1: DAD1 B, Sig=210,4 Ref=360,100

Peak #	RetTime [min]	Type	Width [min]	Area [mAU*s]	Height [mAU]	Area %
1	6.163	BV	0.4717	2.46383e4	733.84662	49.4984
2	7.531	VB	0.5742	2.51377e4	594.06598	50.5016

Totals : 4.97760e4 1327.91260

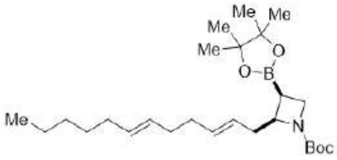

5e
racemic

Data File C:\Users\P...ion\1\Data\SUN\SUN 2025-01-03 11-28-32\002-P2-F2-zmh-7-27d-chiral.D
Sample Name: zmh-7-27d-chiral

===

Acq. Operator : SYSTEM Seq. Line : 2
Sample Operator : SYSTEM
Acq. Instrument : HPLC Location : P2-F-02
Injection Date : 3/1/2025 11:40:53 am Inj : 1
Inj Volume : 2.000 µl
Different Inj Volume from Sample Entry! Actual Inj Volume : 20.000 µl
Acq. Method : C:\Users\Public\Documents\ChemStation\1\Data\SUN\SUN 2025-01-03 11-28-32\IC3-05-30.M
Last changed : 18/4/2023 10:37:59 pm by SYSTEM
Analysis Method : C:\Users\Public\Documents\ChemStation\1\Data\SUN\SUN 2025-01-03 11-28-32\IC3-05-30.M (Sequence Method)
Last changed : 8/5/2025 7:50:08 pm by SYSTEM
(modified after loading)
Additional Info : Peak(s) manually integrated

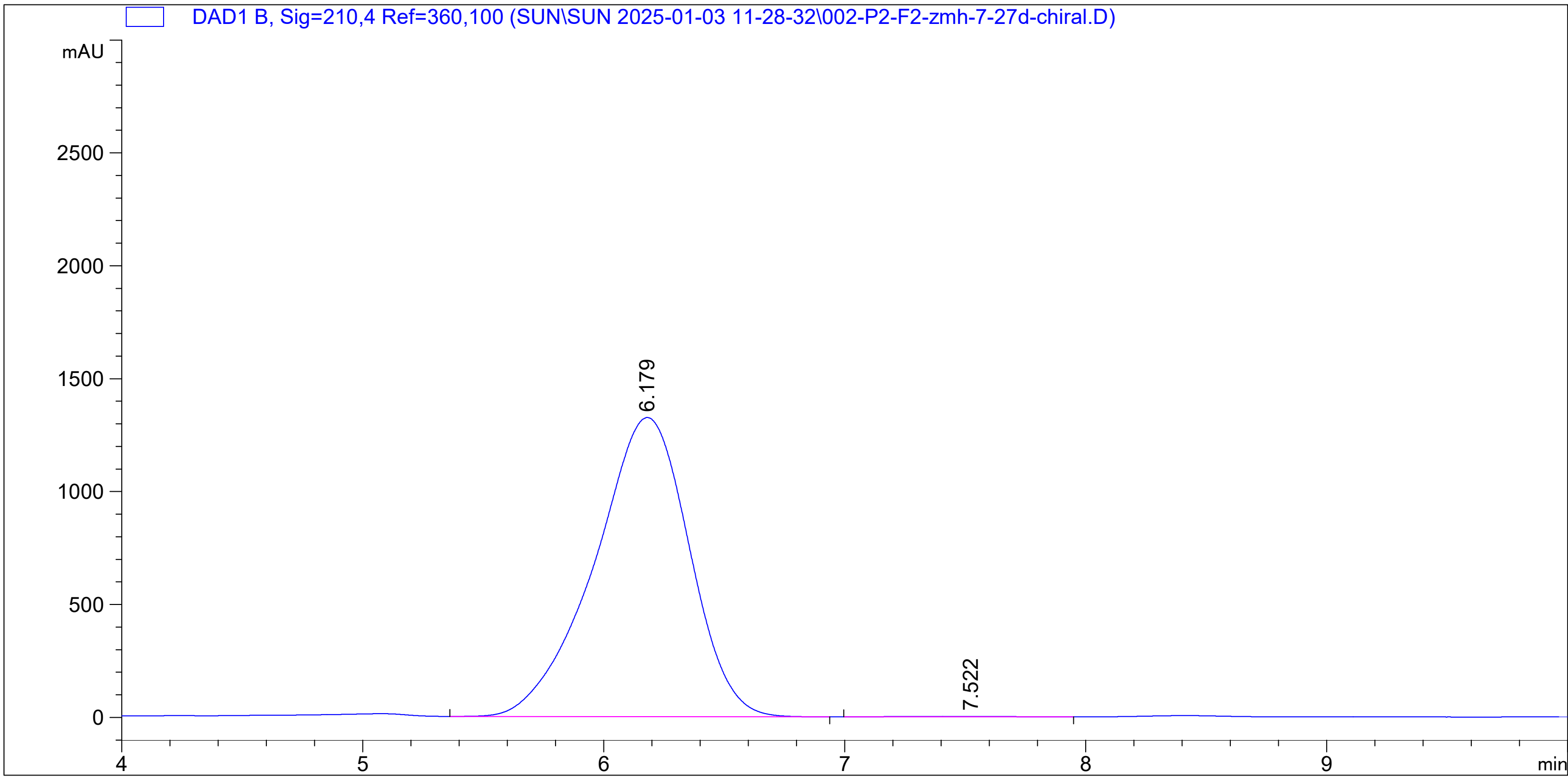

===

Area Percent Report

===

Sorted By : Signal
Multiplier : 1.0000
Dilution : 1.0000
Use Multiplier & Dilution Factor with ISTDs

Signal 1: DAD1 B, Sig=210,4 Ref=360,100

Peak #	RetTime [min]	Type	Width [min]	Area [mAU*s]	Height [mAU]	Area %
1	6.179	BB	0.3506	3.61317e4	1325.08582	99.8277
2	7.522	BV R	0.3521	62.35725	2.07510	0.1723

Totals : 3.61940e4 1327.16091

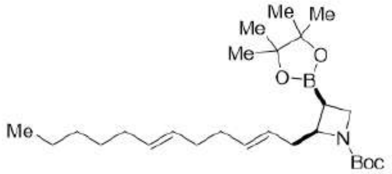

(*S*,*S*)-**5e**
enantioenriched

Data File C:\Users\P...on\1\Data\SUN\SUN 2025-02-26 16-02-52\038-P1-F1-zmh-7-114a-rac-ay.D
Sample Name: zmh-7-114a-rac-ay

===

Acq. Operator : SYSTEM   Seq. Line : 38
Acq. Instrument : LC1260   Location : P1-F-01
Injection Date : 2/26/2025 11:47:38 PM   Inj : 1
Inj Volume : 5.000 µl
Different Inj Volume from Sample Entry! Actual Inj Volume : 10.000 µl
Acq. Method : C:\Users\Public\Documents\ChemStation\1\Data\SUN\SUN 2025-02-26 16-02-52\AY3-10-20.M
Last changed : 9/5/2024 2:31:02 PM by SYSTEM
Analysis Method : C:\Users\Public\Documents\ChemStation\1\Data\SUN\SUN 2025-02-26 16-02-52\AY3-10-20.M (Sequence Method)
Last changed : 5/8/2025 7:59:44 PM by SYSTEM
(modified after loading)
Additional Info : Peak(s) manually integrated

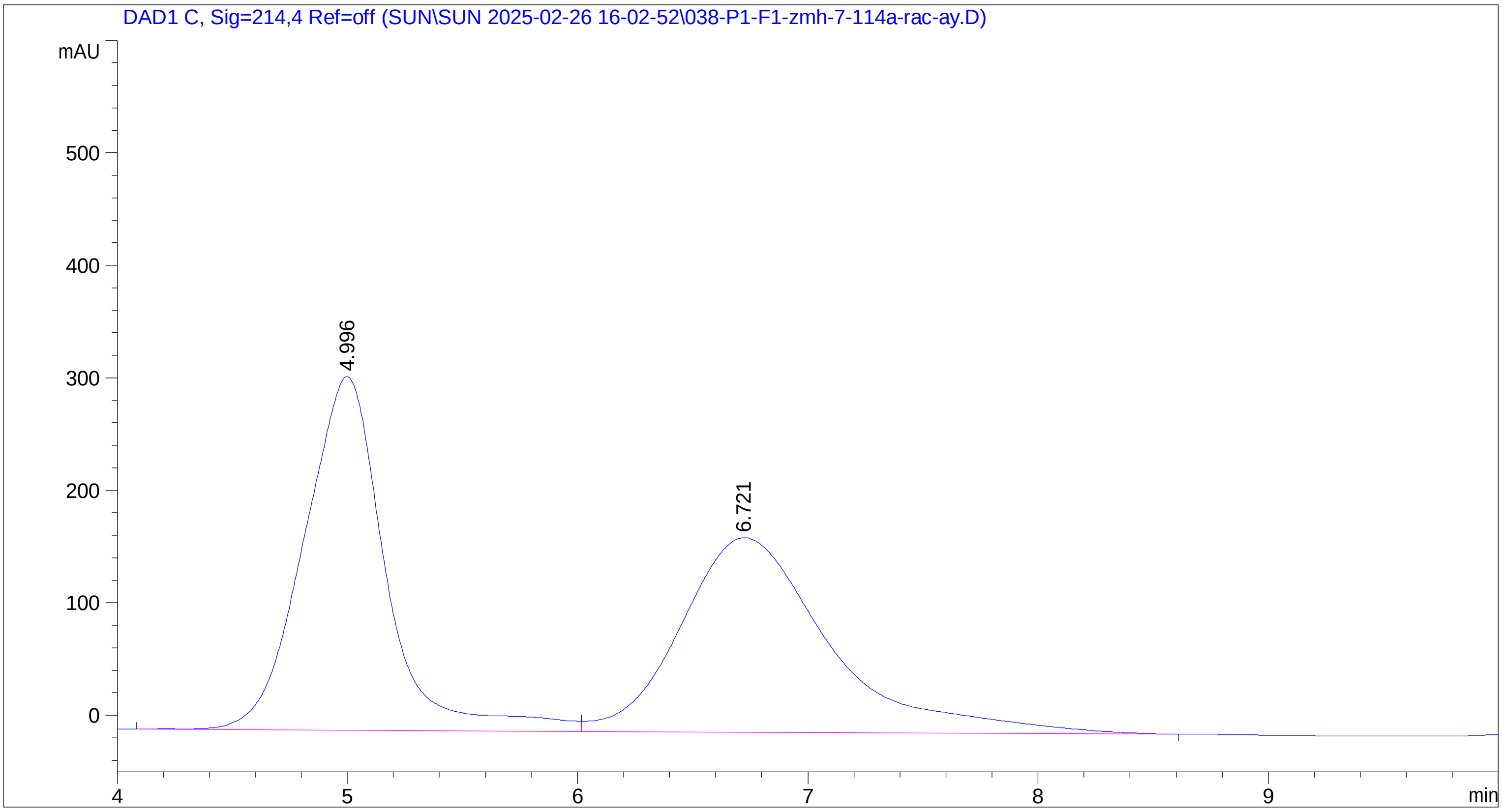

===

Area Percent Report

===

Sorted By : Signal
Multiplier : 1.0000
Dilution : 1.0000
Use Multiplier & Dilution Factor with ISTDs

Signal 1: DAD1 C, Sig=214,4 Ref=off

Peak #	RetTime [min]	Type	Width [min]	Area [mAU*s]	Height [mAU]	Area %
1	4.996	BV R	0.3591	7791.07373	314.36920	50.1279
2	6.721	VB	0.6771	7751.32080	172.88660	49.8721

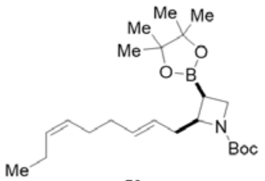

5f
racemic

Totals : 1.55424e4 487.25580

Data File C:\Users\P...on\1\Data\SUN\SUN 2025-02-26 16-02-52\066-P1-F2-zmh-7-114a-chiral.D
Sample Name: zmh-7-114a-chiral

===

Acq. Operator : SYSTEM Seq. Line : 66
Acq. Instrument : LC1260 Location : P1-F-02
Injection Date : 2/27/2025 9:35:27 AM Inj : 1
Inj Volume : 5.000 µl
Different Inj Volume from Sample Entry! Actual Inj Volume : 10.000 µl
Acq. Method : C:\Users\Public\Documents\ChemStation\1\Data\SUN\SUN 2025-02-26 16-02-52 \AY3-10-20.M
Last changed : 9/5/2024 2:31:02 PM by SYSTEM
Analysis Method : C:\Users\Public\Documents\ChemStation\1\Data\SUN\SUN 2025-02-26 16-02-52 \AY3-10-20.M (Sequence Method)
Last changed : 5/8/2025 8:04:59 PM by SYSTEM (modified after loading)
Additional Info : Peak(s) manually integrated

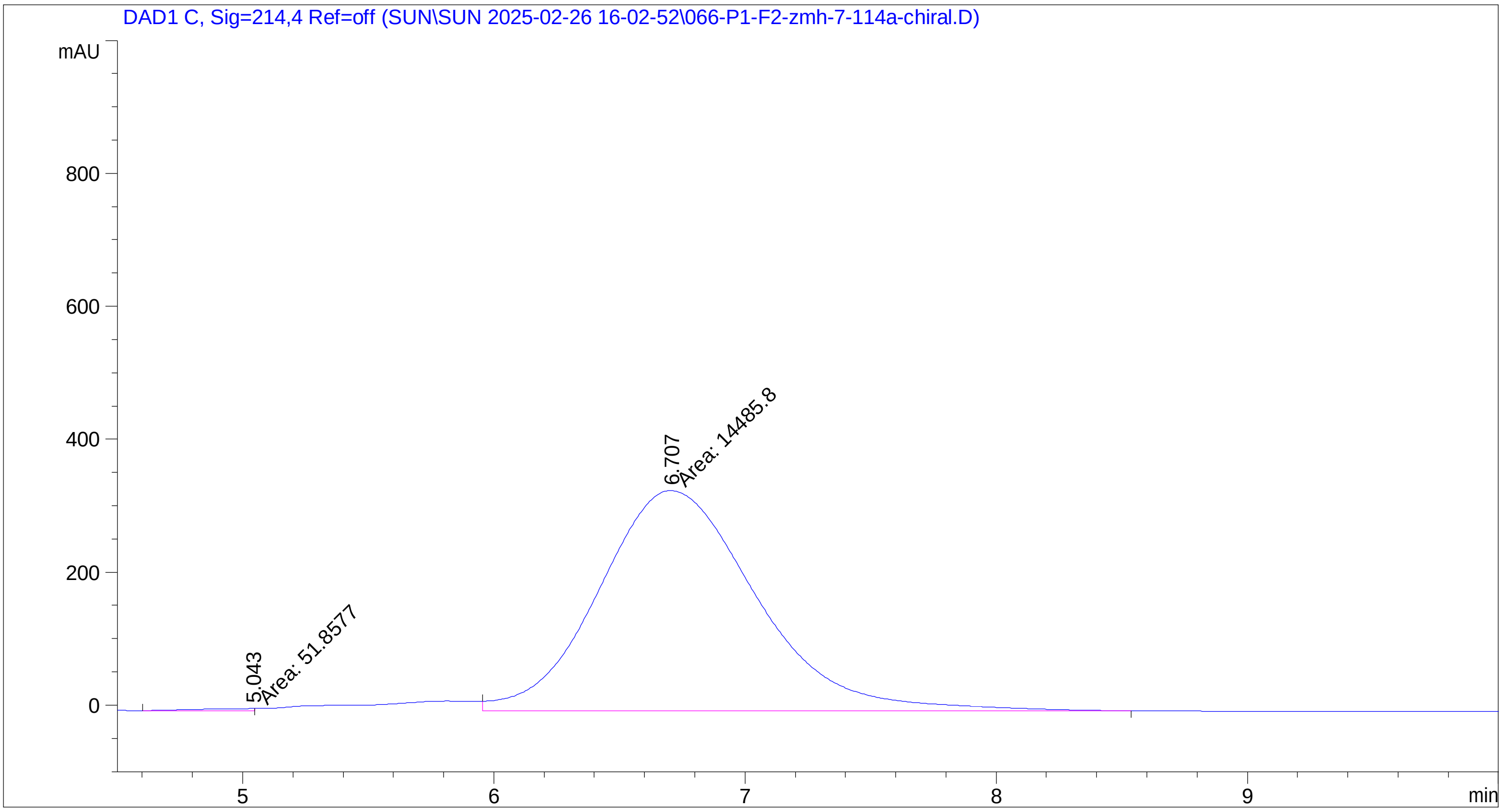

===

Area Percent Report

===

Sorted By : Signal
Multiplier : 1.0000
Dilution : 1.0000
Use Multiplier & Dilution Factor with ISTDs

Signal 1: DAD1 C, Sig=214,4 Ref=off

Peak #	RetTime [min]	Type	Width [min]	Area [mAU*s]	Height [mAU]	Area %
1	5.043	MF	0.2692	51.85771	3.21087	0.3567
2	6.707	FM	0.7288	1.44858e4	331.26526	99.6433

Totals : 1.45376e4 334.47613

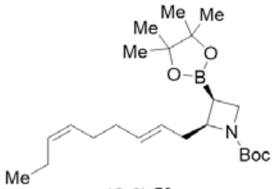

(*S*,*S*)-**5f**
enantioenriched

Data File C:\Users\P...ion\1\Data\SUN\SUN 2024-12-29 22-31-18\014-P1-F3-zmh-7-53c-rac-ic.D
Sample Name: zmh-7-53c-rac-ic

===

```
Acq. Operator   : SYSTEM                          Seq. Line :  14
Sample Operator : SYSTEM
Acq. Instrument : HPLC                             Location :   P1-F-03
Injection Date  : 30/12/2024 2:39:25 am                 Inj :   1
                                                 Inj Volume : 2.000 µl
Different Inj Volume from Sample Entry!  Actual Inj Volume : 20.000 µl
Acq. Method     : C:\Users\Public\Documents\ChemStation\1\Data\SUN\SUN 2024-12-29 22-31-18\IC3-
                  10-20.M
Last changed    : 15/8/2022 10:26:28 pm by SYSTEM
Analysis Method : C:\Users\Public\Documents\ChemStation\1\Data\SUN\SUN 2024-12-29 22-31-18\IC3-
                  10-20.M (Sequence Method)
Last changed    : 9/6/2025 12:57:28 pm by SYSTEM
                  (modified after loading)
Additional Info : Peak(s) manually integrated
```

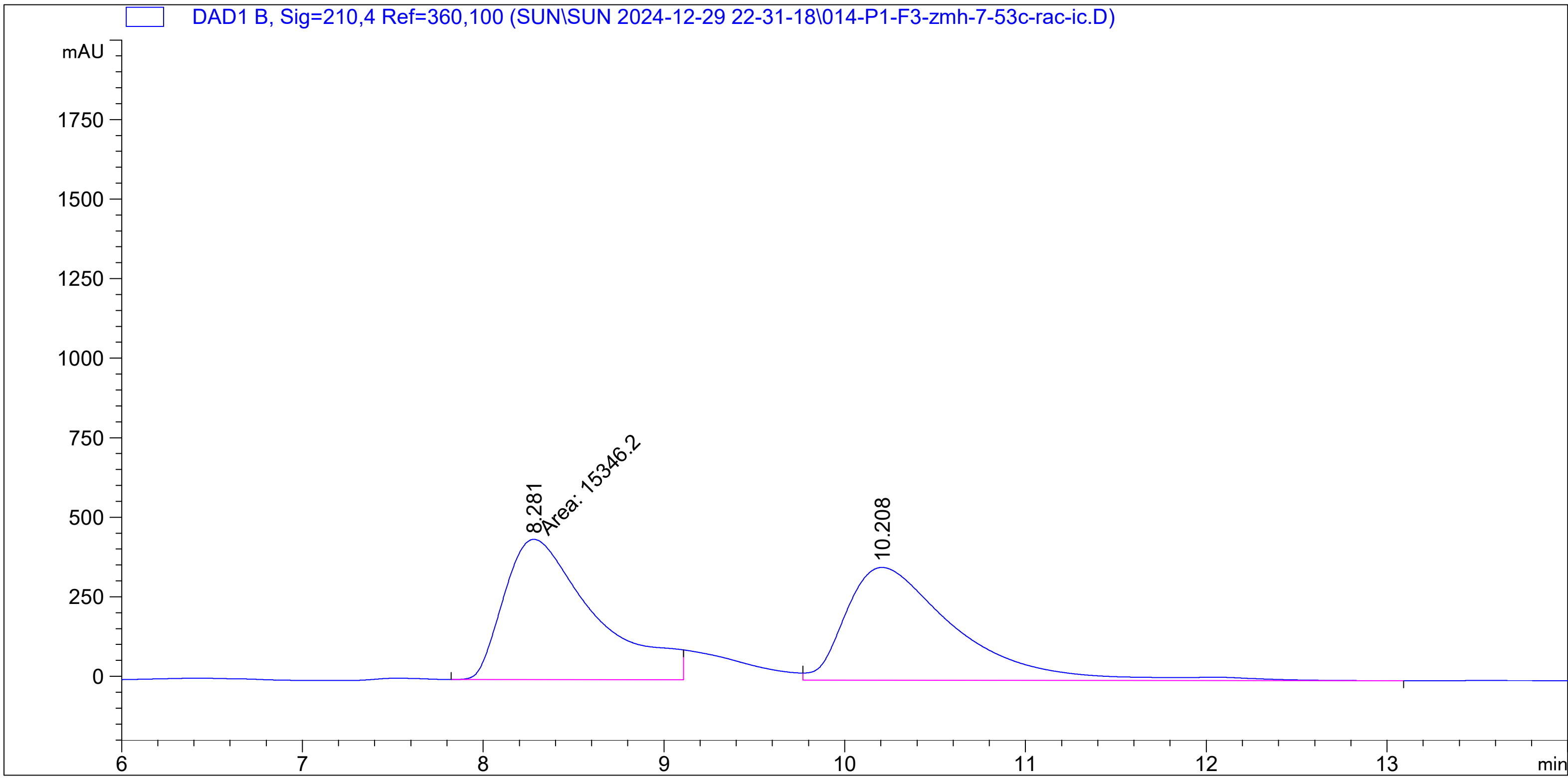

===
Area Percent Report
===

Sorted By : Signal
Multiplier : 1.0000
Dilution : 1.0000
Use Multiplier & Dilution Factor with ISTDs

Signal 1: DAD1 B, Sig=210,4 Ref=360,100

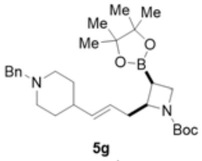

5g
racemic

Peak #	RetTime [min]	Type	Width [min]	Area [mAU*s]	Height [mAU]	Area %
1	8.281	MF	0.5799	1.53462e4	441.02475	50.6067
2	10.208	VV R	0.5744	1.49783e4	354.17252	49.3933

Totals : 3.03245e4 795.19727

Data File C:\Users\P...ion\1\Data\SUN\SUN 2024-12-31 10-21-01\004-P1-A2-zmh-7-28g-chiral.D
Sample Name: zmh-7-28g-chiral

===

Acq. Operator : SYSTEM Seq. Line : 4
Sample Operator : SYSTEM
Acq. Instrument : HPLC Location : P1-A-02
Injection Date : 31/12/2024 11:06:31 am Inj : 1
Inj Volume : 2.000 µl
Different Inj Volume from Sample Entry! Actual Inj Volume : 20.000 µl
Acq. Method : C:\Users\Public\Documents\ChemStation\1\Data\SUN\SUN 2024-12-31 10-21-01\IC3-10-20.M
Last changed : 15/8/2022 10:26:28 pm by SYSTEM
Analysis Method : C:\Users\Public\Documents\ChemStation\1\Data\SUN\SUN 2024-12-31 10-21-01\IC3-10-20.M (Sequence Method)
Last changed : 8/5/2025 7:55:47 pm by SYSTEM
(modified after loading)
Additional Info : Peak(s) manually integrated

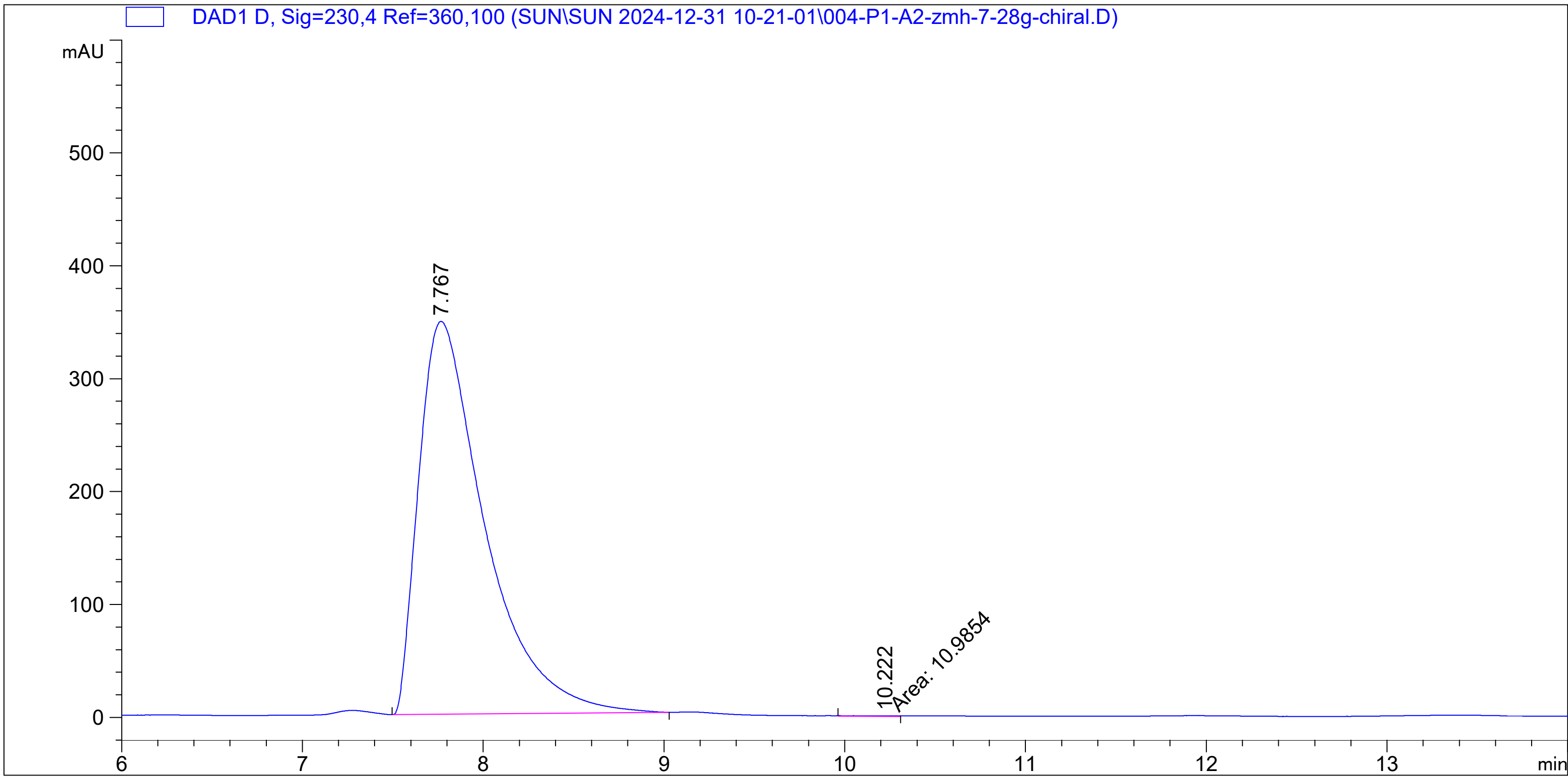

===

Area Percent Report

===

Sorted By : Signal
Multiplier : 1.0000
Dilution : 1.0000
Use Multiplier & Dilution Factor with ISTDs

Signal 1: DAD1 D, Sig=230,4 Ref=360,100

Peak #	RetTime [min]	Type	Width [min]	Area [mAU*s]	Height [mAU]	Area %
1	7.767	BB	0.3799	8825.78613	348.01794	99.8757
2	10.222	MM	0.3120	10.98539	5.86872e-1	0.1243

Totals : 8836.77153 348.60482

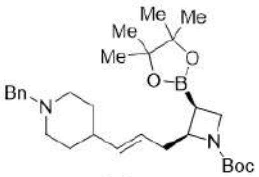

(*S*,*S*)-**5g**
enantioenriched

===

```
Acq. Operator   : SYSTEM                        Seq. Line : 121
Sample Operator : SYSTEM
Acq. Instrument : HPLC                           Location :   P1-D-02
Injection Date  : 3/1/2025 2:19:21 am                 Inj :   1
                                               Inj Volume : 2.000 µl
Different Inj Volume from Sample Entry!  Actual Inj Volume : 20.000 µl
Acq. Method     : C:\Users\Public\Documents\ChemStation\1\Data\SUN\SUN 2025-01-01 13-17-07\ID3-
                  05-30.M
Last changed    : 21/12/2024 9:29:52 pm by SYSTEM
Analysis Method : C:\Users\Public\Documents\ChemStation\1\Data\SUN\SUN 2025-01-01 13-17-07\ID3-
                  05-30.M (Sequence Method)
Last changed    : 8/5/2025 8:00:17 pm by SYSTEM
                  (modified after loading)
Additional Info : Peak(s) manually integrated
```

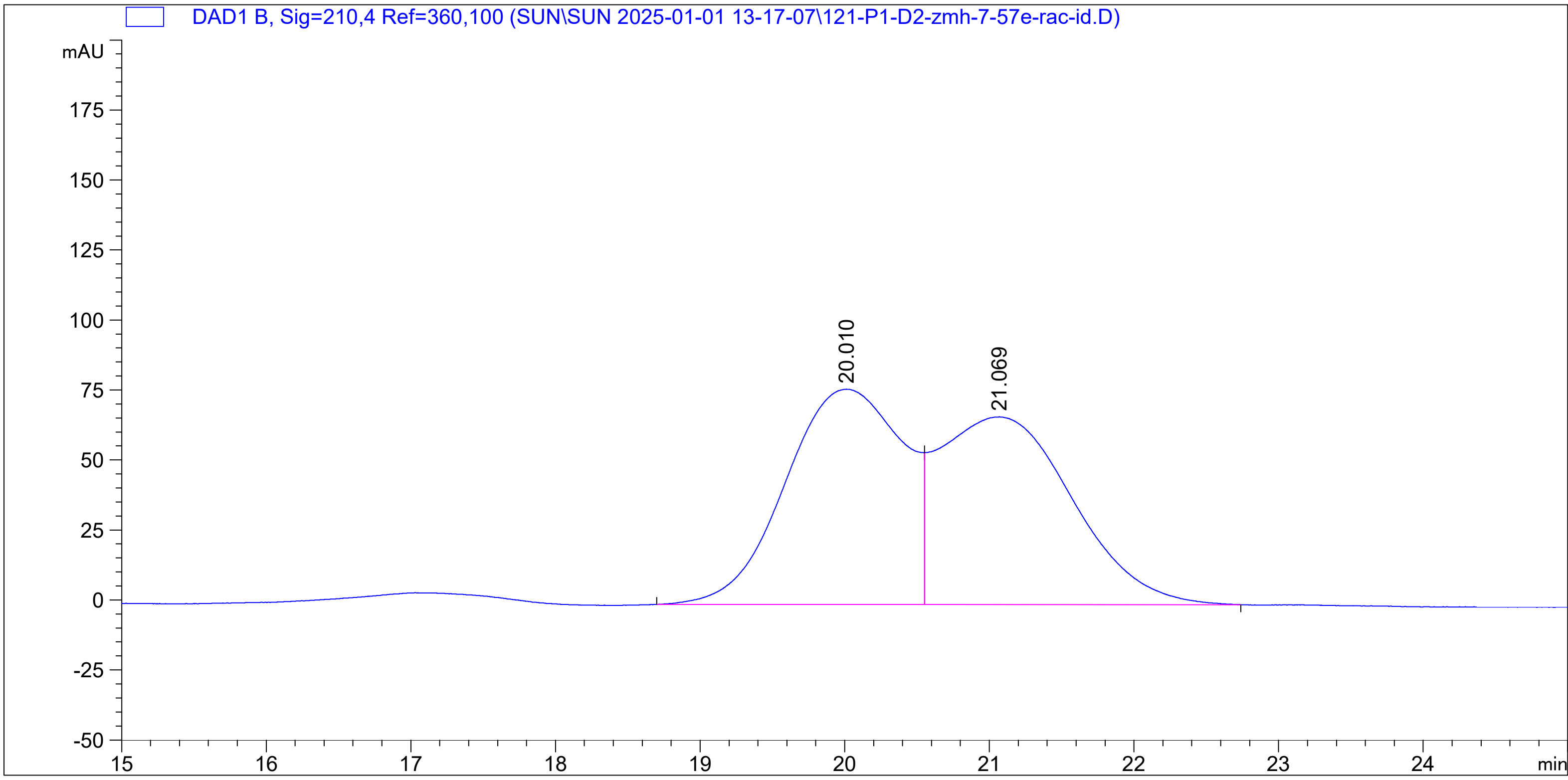

===

Area Percent Report

===

```
Sorted By             :      Signal
Multiplier            :      1.0000
Dilution              :      1.0000
Use Multiplier & Dilution Factor with ISTDs
```

Signal 1: DAD1 B, Sig=210,4 Ref=360,100

Peak #	RetTime [min]	Type	Width [min]	Area [mAU*s]	Height [mAU]	Area %
1	20.010	BV	0.6645	4362.78125	76.94433	50.4725
2	21.069	VB	0.7480	4281.10205	67.04302	49.5275
Totals :				8643.88330	143.98735	

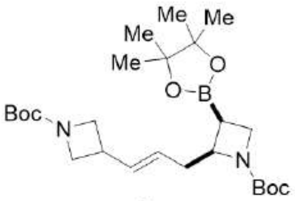

5h
racemic

Data File C:\Users\P...ion\1\Data\SUN\SUN 2025-01-03 11-28-32\006-P2-F3-zmh-7-27g-chiral.D
Sample Name: zmh-7-27g-chiral

===

```
Acq. Operator   : SYSTEM                          Seq. Line :    6
Sample Operator : SYSTEM
Acq. Instrument : HPLC                             Location :   P2-F-03
Injection Date  : 3/1/2025 12:45:12 pm                  Inj :    1
                                                 Inj Volume : 2.000 µl
Different Inj Volume from Sample Entry!  Actual Inj Volume : 20.000 µl
Acq. Method     : C:\Users\Public\Documents\ChemStation\1\Data\SUN\SUN 2025-01-03 11-28-32\ID3-
                  05-30.M
Last changed    : 21/12/2024 9:29:52 pm by SYSTEM
Analysis Method : C:\Users\Public\Documents\ChemStation\1\Data\SUN\SUN 2025-01-03 11-28-32\ID3-
                  05-30.M (Sequence Method)
Last changed    : 8/5/2025 8:03:13 pm by SYSTEM
                  (modified after loading)
Additional Info : Peak(s) manually integrated
```

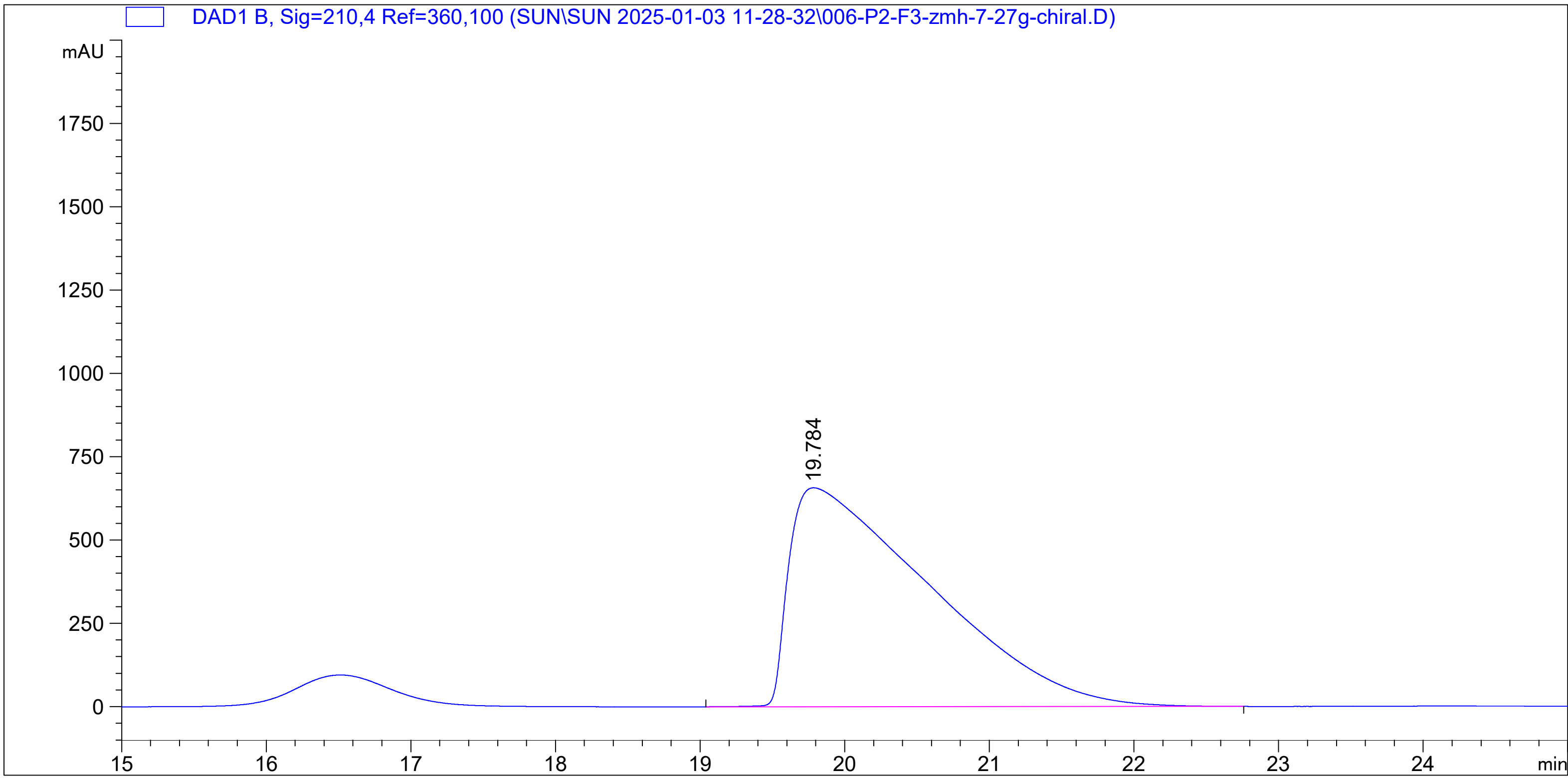

===
Area Percent Report
===

```
Sorted By             :      Signal
Multiplier            :      1.0000
Dilution              :      1.0000
Use Multiplier & Dilution Factor with ISTDs
```

Signal 1: DAD1 B, Sig=210,4 Ref=360,100

Peak #	RetTime [min]	Type	Width [min]	Area [mAU*s]	Height [mAU]	Area %
1	19.784	BB	0.7960	4.45437e4	657.00995	100.0000

Totals : 4.45437e4 657.00995

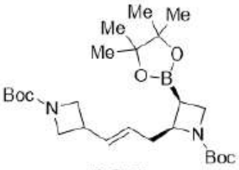

(*S*,*S*)-**5h**
enantioenriched

Data File C:\Users\P...ion\1\Data\SUN\SUN 2024-12-29 22-31-18\015-P1-F4-zmh-7-53e-rac-ic.D
Sample Name: zmh-7-53e-rac-ic

===

Acq. Operator : SYSTEM Seq. Line : 15
Sample Operator : SYSTEM
Acq. Instrument : HPLC Location : P1-F-04
Injection Date : 30/12/2024 3:01:02 am Inj : 1
Inj Volume : 2.000 µl
Different Inj Volume from Sample Entry! Actual Inj Volume : 20.000 µl
Acq. Method : C:\Users\Public\Documents\ChemStation\1\Data\SUN\SUN 2024-12-29 22-31-18\IC3-10-20.M
Last changed : 15/8/2022 10:26:28 pm by SYSTEM
Analysis Method : C:\Users\Public\Documents\ChemStation\1\Data\SUN\SUN 2024-12-29 22-31-18\IC3-10-20.M (Sequence Method)
Last changed : 9/6/2025 1:02:28 pm by SYSTEM
(modified after loading)
Additional Info : Peak(s) manually integrated

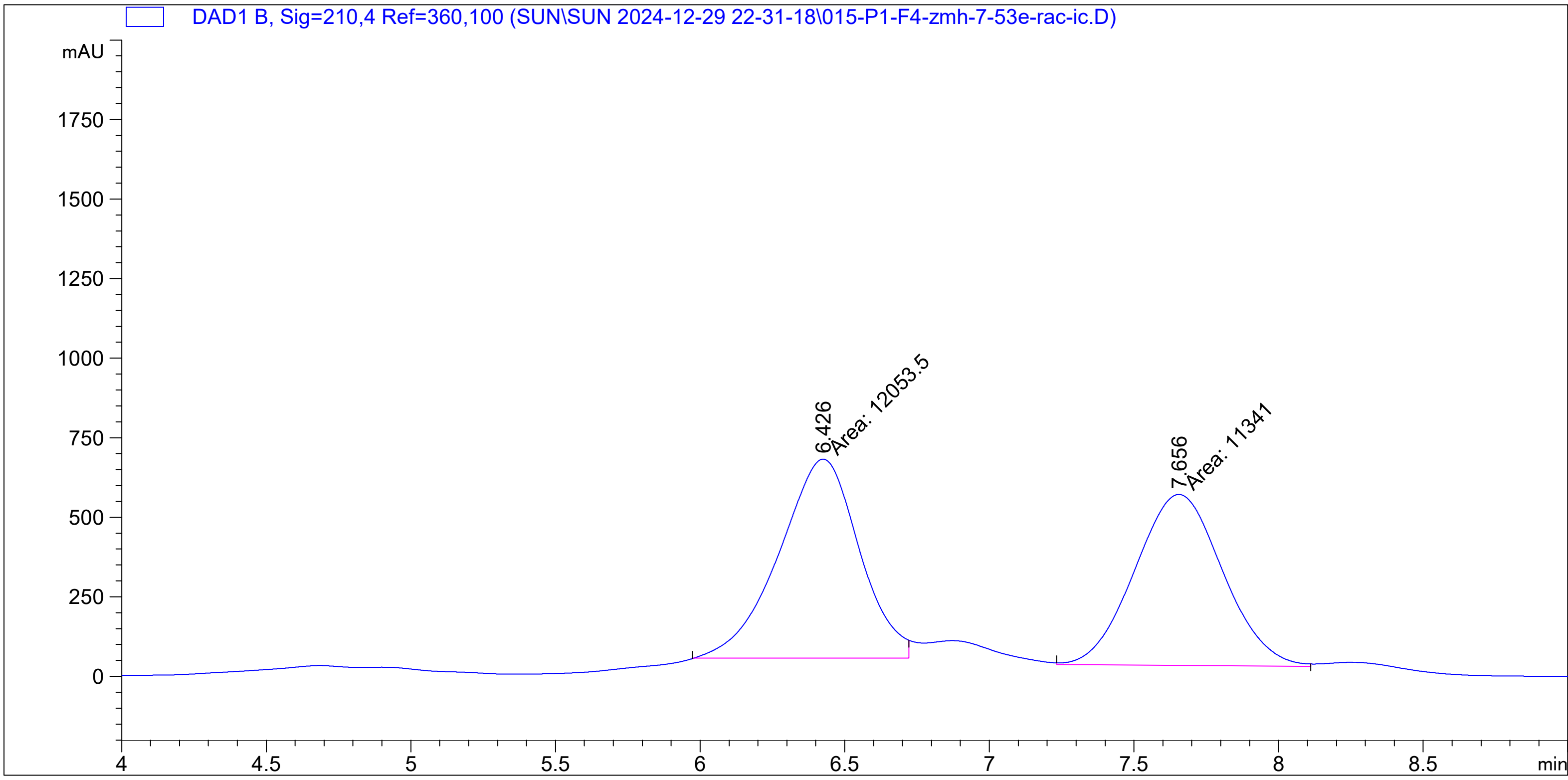

===

Area Percent Report

===

Sorted By : Signal
Multiplier : 1.0000
Dilution : 1.0000
Use Multiplier & Dilution Factor with ISTDs

Signal 1: DAD1 B, Sig=210,4 Ref=360,100

Peak #	RetTime [min]	Type	Width [min]	Area [mAU*s]	Height [mAU]	Area %
1	6.426	MF	0.3213	1.20535e4	625.15833	51.5229
2	7.656	MM	0.3518	1.13410e4	537.33099	48.4771

Totals : 2.33945e4 1162.48932

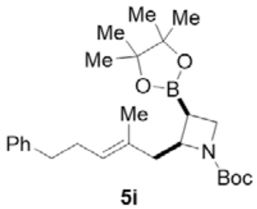

5i
racemic

===

Acq. Operator : SYSTEM   Seq. Line : 14
Sample Operator : SYSTEM
Acq. Instrument : HPLC   Location : P1-D-02
Injection Date : 31/12/2024 1:48:26 pm   Inj : 1
Inj Volume : 2.000 µl
Different Inj Volume from Sample Entry! Actual Inj Volume : 20.000 µl
Acq. Method : C:\Users\Public\Documents\ChemStation\1\Data\SUN\SUN 2024-12-31 10-21-01\IC3-10-20.M
Last changed : 15/8/2022 10:26:28 pm by SYSTEM
Analysis Method : C:\Users\Public\Documents\ChemStation\1\Data\SUN\SUN 2024-12-31 10-21-01\IC3-10-20.M (Sequence Method)
Last changed : 8/5/2025 8:08:56 pm by SYSTEM
(modified after loading)
Additional Info : Peak(s) manually integrated

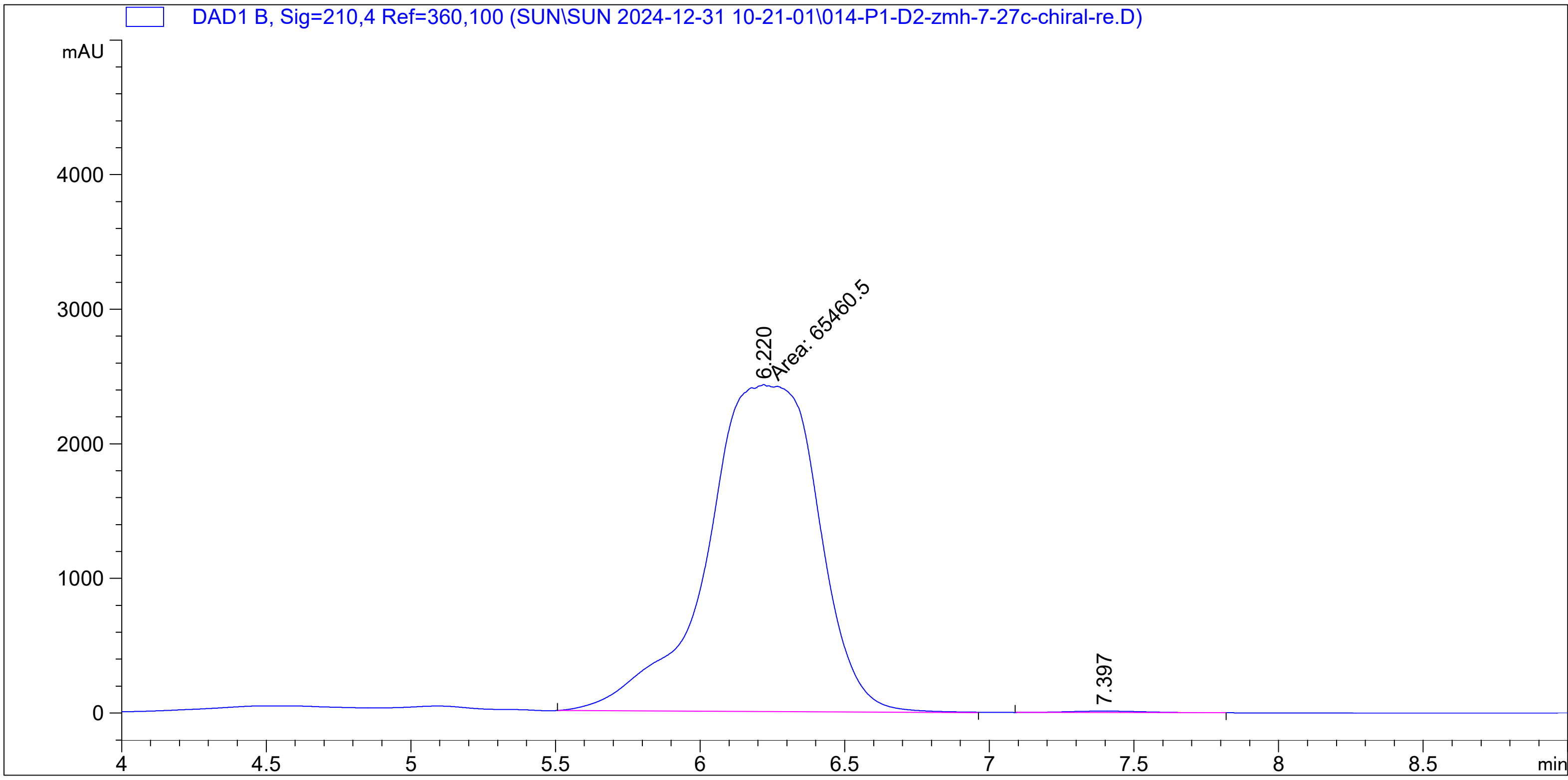

===
Area Percent Report
===

Sorted By : Signal
Multiplier : 1.0000
Dilution : 1.0000
Use Multiplier & Dilution Factor with ISTDs

Signal 1: DAD1 B, Sig=210,4 Ref=360,100

Peak #	RetTime [min]	Type	Width [min]	Area [mAU*s]	Height [mAU]	Area %
1	6.220	MM	0.4489	6.54605e4	2430.52490	99.6807
2	7.397	BB	0.2150	209.67934	11.53820	0.3193

Totals : 6.56702e4 2442.06310

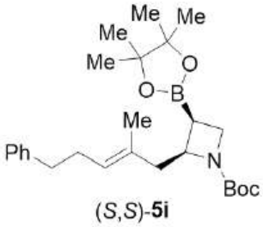

(*S*,*S*)-**5i**
enantioenriched

===

Acq. Operator : SYSTEM Seq. Line : 10
Sample Operator : SYSTEM
Acq. Instrument : HPLC Location : P2-A-04
Injection Date : 3/1/2025 2:13:07 pm Inj : 1
Inj Volume : 2.000 µl
Different Inj Volume from Sample Entry! Actual Inj Volume : 20.000 µl
Acq. Method : C:\Users\Public\Documents\ChemStation\1\Data\SUN\SUN 2025-01-03 11-28-32\IC3-01-40.M
Last changed : 31/1/2024 9:31:55 pm by SYSTEM
Analysis Method : C:\Users\Public\Documents\ChemStation\1\Data\SUN\SUN 2025-01-03 11-28-32\IC3-01-40.M (Sequence Method)
Last changed : 8/5/2025 8:11:16 pm by SYSTEM
(modified after loading)
Additional Info : Peak(s) manually integrated

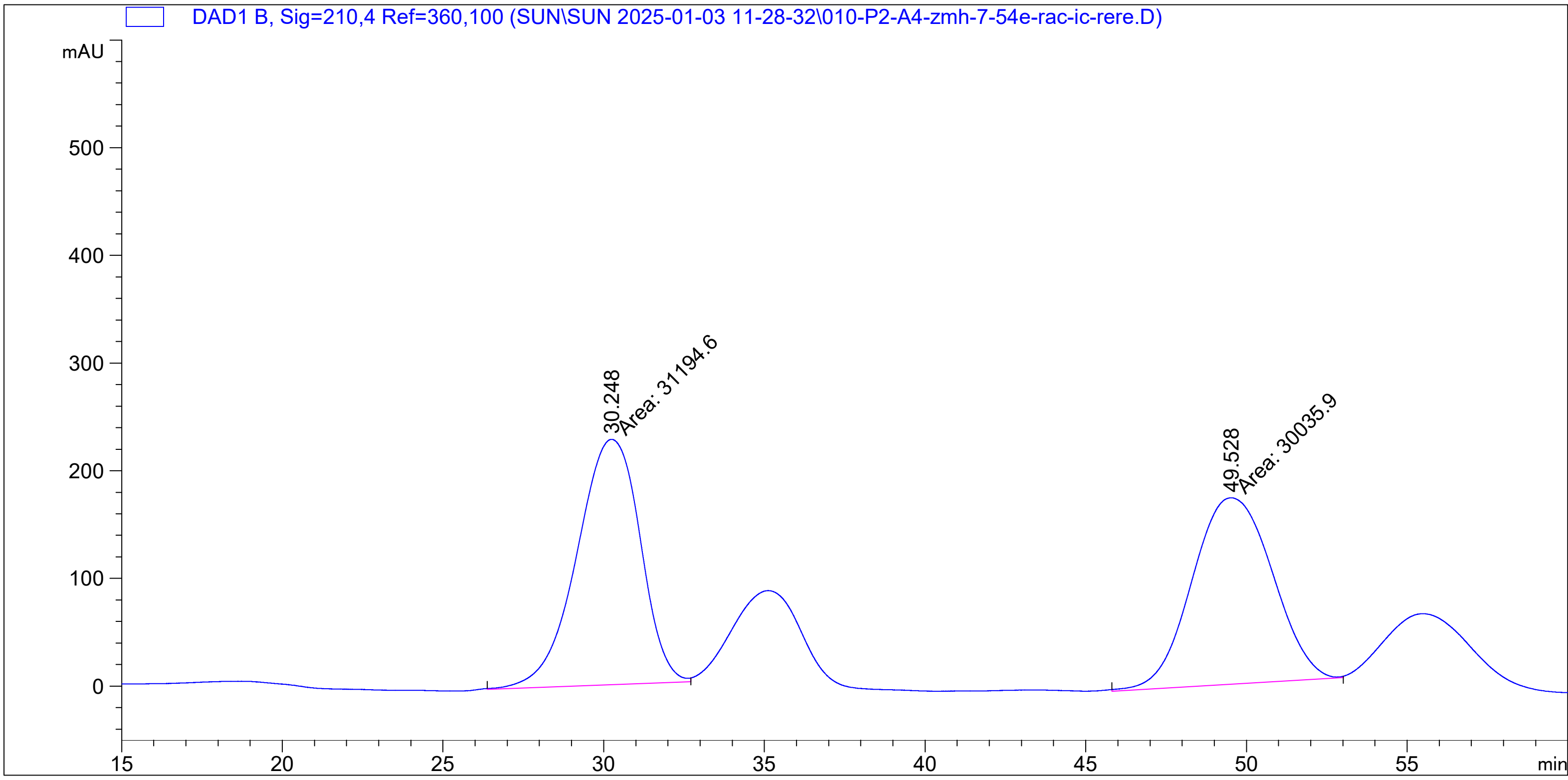

===
Area Percent Report
===

Sorted By : Signal
Multiplier : 1.0000
Dilution : 1.0000
Use Multiplier & Dilution Factor with ISTDs

Signal 1: DAD1 B, Sig=210,4 Ref=360,100

Peak #	RetTime [min]	Type	Width [min]	Area [mAU*s]	Height [mAU]	Area %
1	30.248	MM	2.2822	3.11946e4	227.81538	50.9462
2	49.528	MM	2.8891	3.00359e4	173.27428	49.0538

Totals : 6.12305e4 401.08966

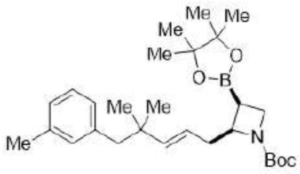

5l
racemic

Data File C:\Users\P...ion\1\Data\SUN\SUN 2025-01-03 11-28-32\016-P1-B3-zmh-7-24b-chiral.D
Sample Name: zmh-7-24b-chiral

===

Acq. Operator	: SYSTEM	Seq. Line :	16
Sample Operator	: SYSTEM		
Acq. Instrument	: HPLC	Location :	P1-B-03
Injection Date	: 3/1/2025 5:00:23 pm	Inj :	1
		Inj Volume :	2.000 µl

Different Inj Volume from Sample Entry! Actual Inj Volume : 10.000 µl

Acq. Method : C:\Users\Public\Documents\ChemStation\1\Data\SUN\SUN 2025-01-03 11-28-32\IC3-01-40.M
Last changed : 31/1/2024 9:31:55 pm by SYSTEM
Analysis Method : C:\Users\Public\Documents\ChemStation\1\Data\SUN\SUN 2025-01-03 11-28-32\IC3-01-40.M (Sequence Method)
Last changed : 8/5/2025 8:12:55 pm by SYSTEM
(modified after loading)
Additional Info : Peak(s) manually integrated

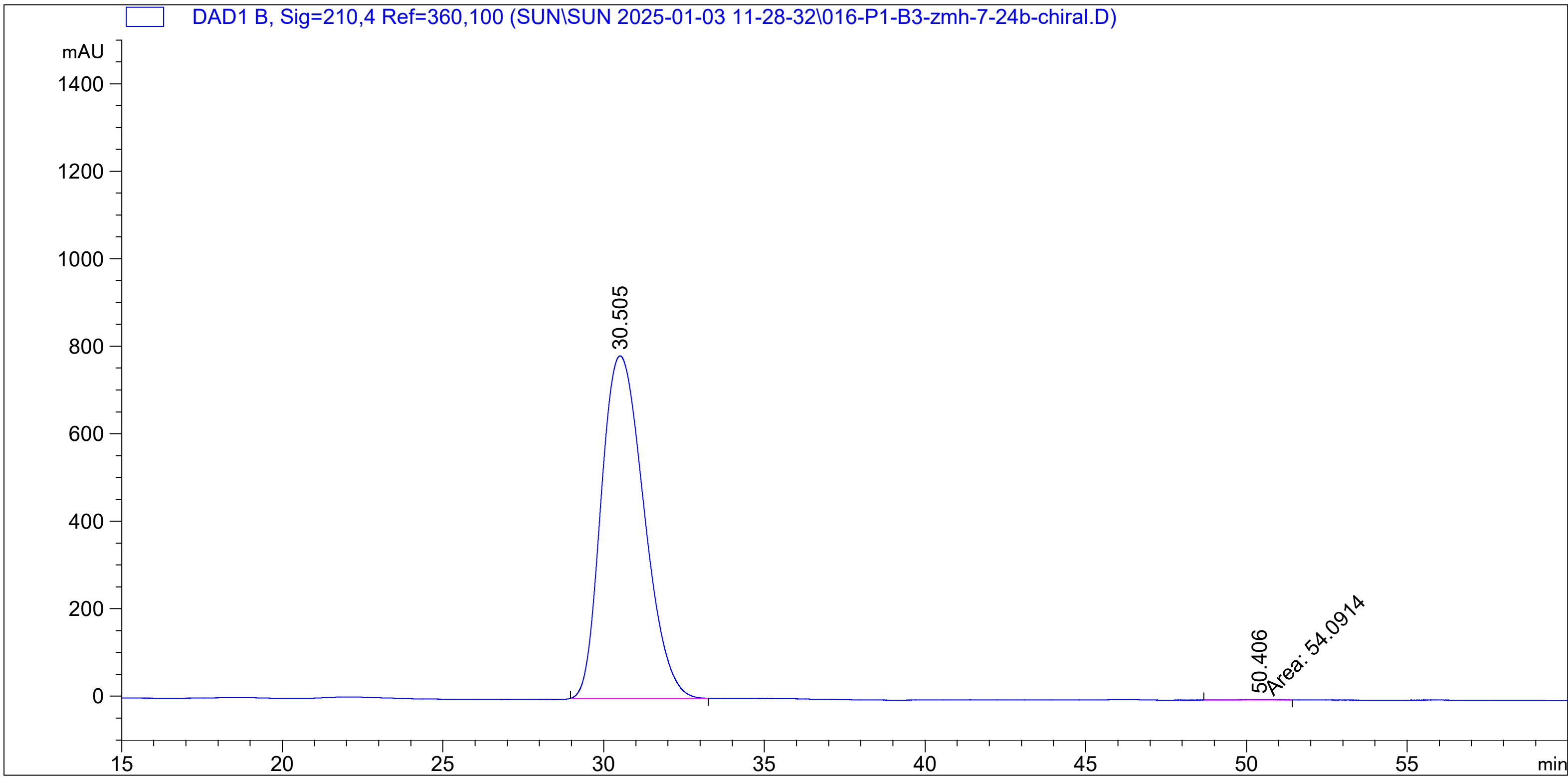

===
Area Percent Report
===

Sorted By : Signal
Multiplier : 1.0000
Dilution : 1.0000
Use Multiplier & Dilution Factor with ISTDs

Signal 1: DAD1 B, Sig=210,4 Ref=360,100

Peak #	RetTime [min]	Type	Width [min]	Area [mAU*s]	Height [mAU]	Area %
1	30.505	BV R	1.0804	7.22204e4	782.77985	99.9252
2	50.406	MM	1.4941	54.09143	6.03373e-1	0.0748
Totals :				7.22745e4	783.38322	

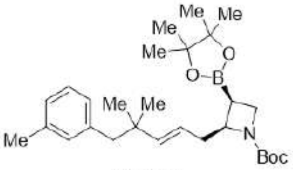

(*S*,*S*)-**5l**
enantioenriched

Data File C:\Users\P...ion\1\Data\SUN\SUN 2025-01-18 10-06-17\013-P1-C1-zmh-7-81a-rac-as.D
Sample Name: zmh-7-81a-rac-as

===

Acq. Operator : SYSTEM Seq. Line : 13
Sample Operator : SYSTEM
Acq. Instrument : HPLC Location : P1-C-01
Injection Date : 18/1/2025 1:01:47 pm Inj : 1
Inj Volume : 2.000 µl
Different Inj Volume from Sample Entry! Actual Inj Volume : 10.000 µl
Acq. Method : C:\Users\Public\Documents\ChemStation\1\Data\SUN\SUN 2025-01-18 10-06-17\AS3-10-20.M
Last changed : 15/8/2022 10:24:04 pm by SYSTEM
Analysis Method : C:\Users\Public\Documents\ChemStation\1\Data\SUN\SUN 2025-01-18 10-06-17\AS3-10-20.M (Sequence Method)
Last changed : 8/5/2025 8:14:45 pm by SYSTEM
(modified after loading)
Additional Info : Peak(s) manually integrated

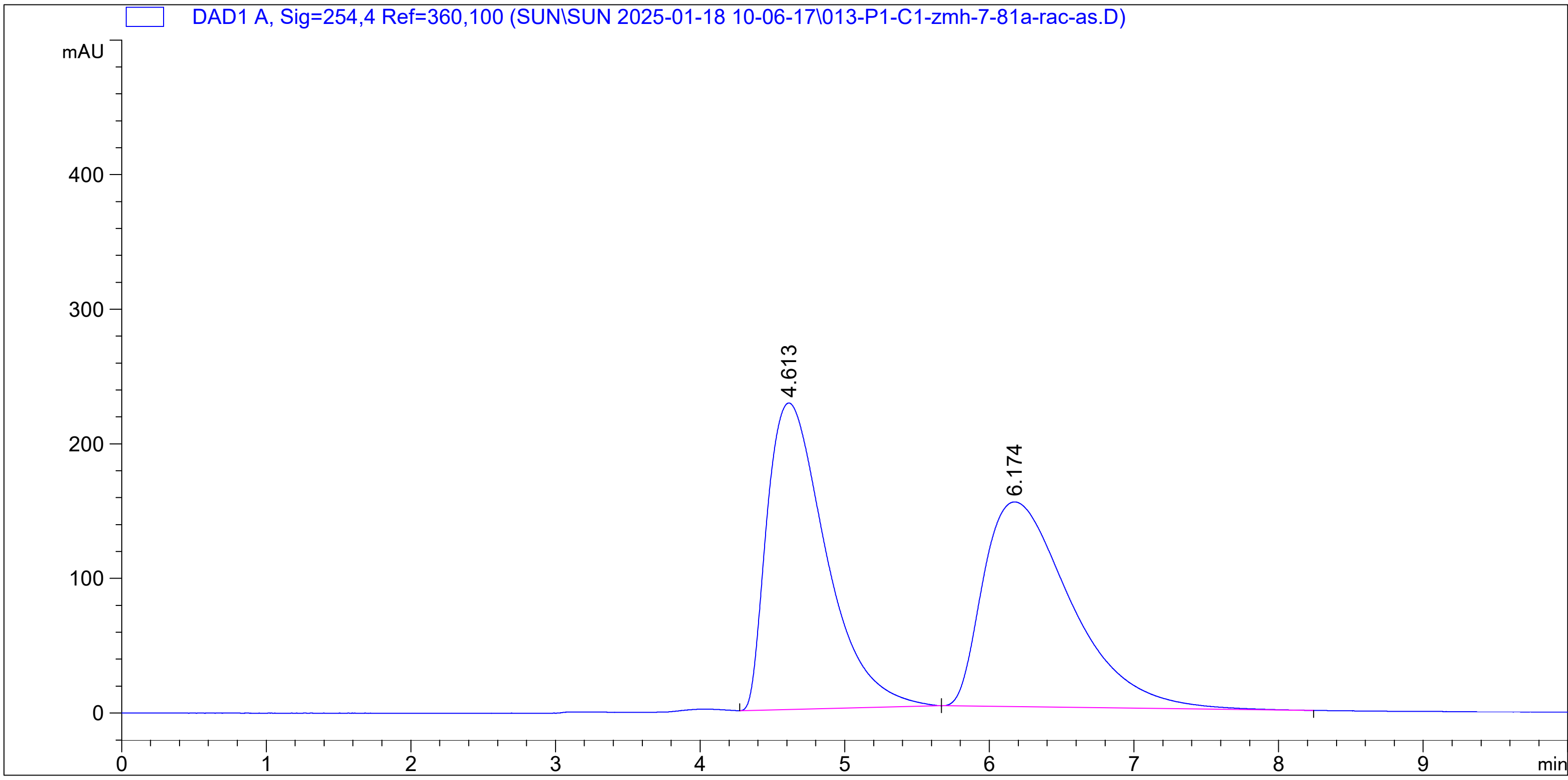

===

Area Percent Report

===

Sorted By : Signal
Multiplier : 1.0000
Dilution : 1.0000
Use Multiplier & Dilution Factor with ISTDs

Signal 1: DAD1 A, Sig=254,4 Ref=360,100

Peak #	RetTime [min]	Type	Width [min]	Area [mAU*s]	Height [mAU]	Area %
1	4.613	BB	0.4224	6448.60986	227.68581	50.0366
2	6.174	BB	0.5842	6439.17920	152.16884	49.9634

Totals : 1.28878e4 379.85464

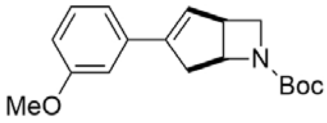

6
racemic

Data File C:\Users\P...ion\1\Data\SUN\SUN 2025-01-18 10-06-17\017-P1-B1-zmh-7-81a-chiral.D
Sample Name: zmh-7-81a-chiral

===

Acq. Operator : SYSTEM Seq. Line : 17
Sample Operator : SYSTEM
Acq. Instrument : HPLC Location : P1-B-01
Injection Date : 18/1/2025 2:06:20 pm Inj : 1
Inj Volume : 2.000 µl
Different Inj Volume from Sample Entry! Actual Inj Volume : 10.000 µl
Acq. Method : C:\Users\Public\Documents\ChemStation\1\Data\SUN\SUN 2025-01-18 10-06-17\AS3-10-20.M
Last changed : 15/8/2022 10:24:04 pm by SYSTEM
Analysis Method : C:\Users\Public\Documents\ChemStation\1\Data\SUN\SUN 2025-01-18 10-06-17\AS3-10-20.M (Sequence Method)
Last changed : 8/5/2025 8:14:45 pm by SYSTEM
(modified after loading)

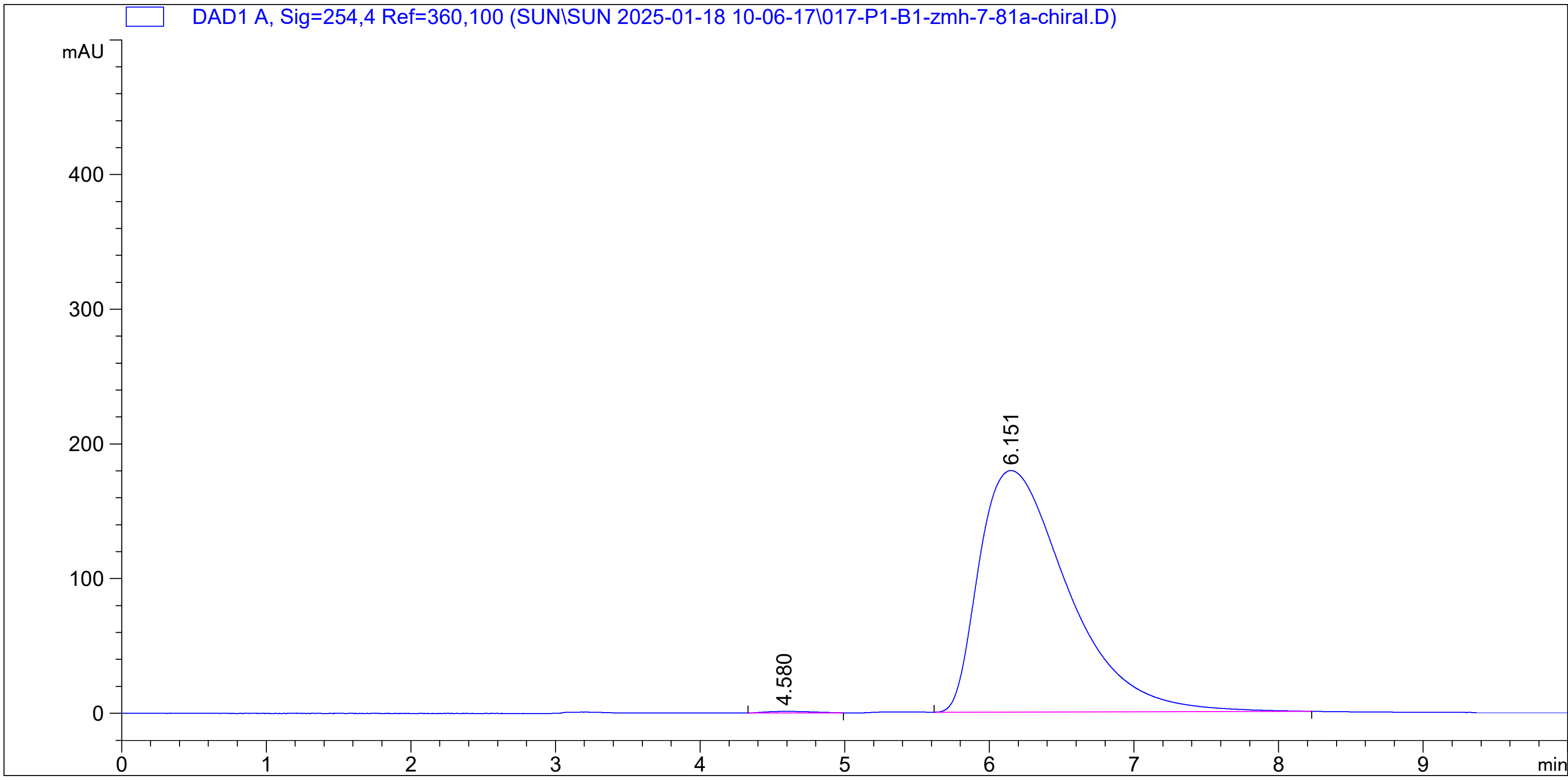

===

Area Percent Report

===

Sorted By : Signal
Multiplier : 1.0000
Dilution : 1.0000
Use Multiplier & Dilution Factor with ISTDs

Signal 1: DAD1 A, Sig=254,4 Ref=360,100

Peak #	RetTime [min]	Type	Width [min]	Area [mAU*s]	Height [mAU]	Area %
1	4.580	BB	0.2636	25.47831	1.14070	0.3310
2	6.151	BB	0.5732	7672.73291	179.30386	99.6690

Totals : 7698.21122 180.44457

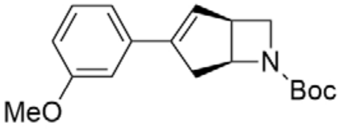

(*S*,*S*)-**6**
enantioenriched

Data File C:\Users\P...tion\1\Data\SUN\SUN 2025-01-17 16-18-28\008-P2-B1-zmh-7-82-rac-ay.D
Sample Name: zmh-7-82-rac-ay

===

Acq. Operator	: SYSTEM	Seq. Line :	8
Sample Operator	: SYSTEM		
Acq. Instrument	: HPLC	Location :	P2-B-01
Injection Date	: 17/1/2025 6:12:05 pm	Inj :	1
		Inj Volume :	2.000 µl

Different Inj Volume from Sample Entry! Actual Inj Volume : 10.000 µl

Acq. Method : C:\Users\Public\Documents\ChemStation\1\Data\SUN\SUN 2025-01-17 16-18-28\AY3-10-20.M
Last changed : 15/8/2022 10:25:04 pm by SYSTEM
Analysis Method : C:\Users\Public\Documents\ChemStation\1\Data\SUN\SUN 2025-01-17 16-18-28\AY3-10-20.M (Sequence Method)
Last changed : 8/5/2025 8:24:39 pm by SYSTEM
(modified after loading)
Additional Info : Peak(s) manually integrated

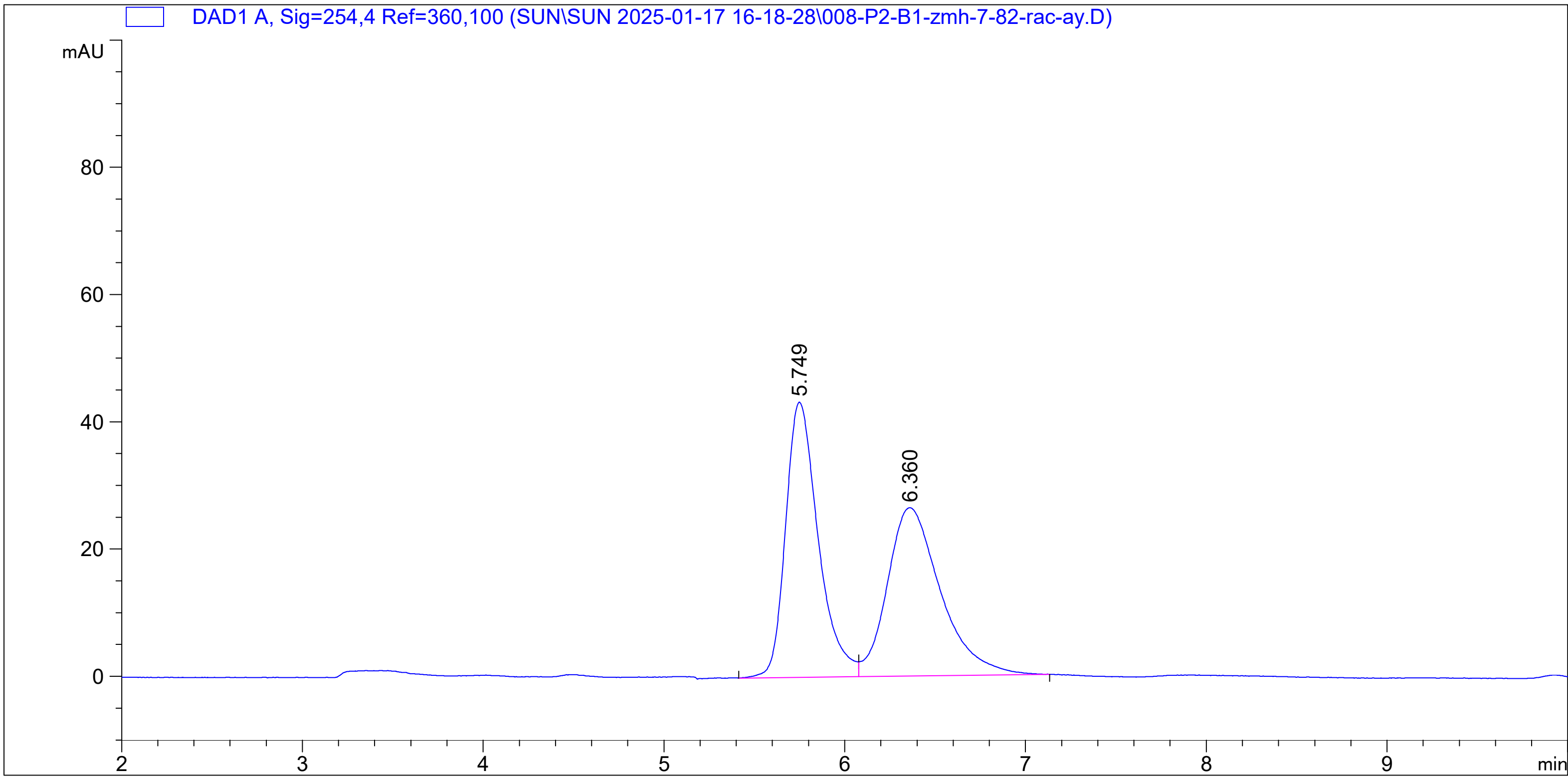

===
Area Percent Report
===

Sorted By : Signal
Multiplier : 1.0000
Dilution : 1.0000
Use Multiplier & Dilution Factor with ISTDs

Signal 1: DAD1 A, Sig=254,4 Ref=360,100

Peak #	RetTime [min]	Type	Width [min]	Area [mAU*s]	Height [mAU]	Area %
1	5.749	BV	0.1912	544.10767	43.27000	50.0700
2	6.360	VV R	0.2741	542.58618	26.47992	49.9300

Totals : 1086.69385 69.74992

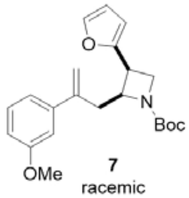

7
racemic

Data File C:\Users\P...tion\1\Data\SUN\SUN 2025-01-17 16-18-28\010-P2-A1-zmh-7-82-chiral.D
Sample Name: zmh-7-82-chiral

===

```
Acq. Operator   : SYSTEM                        Seq. Line :  10
Sample Operator : SYSTEM
Acq. Instrument : HPLC                           Location :  P2-A-01
Injection Date  : 17/1/2025 6:44:31 pm                Inj :  1
                                               Inj Volume : 2.000 µl
Different Inj Volume from Sample Entry!  Actual Inj Volume : 10.000 µl
Acq. Method     : C:\Users\Public\Documents\ChemStation\1\Data\SUN\SUN 2025-01-17 16-18-28\AY3-
                  10-20.M
Last changed    : 15/8/2022 10:25:04 pm by SYSTEM
Analysis Method : C:\Users\Public\Documents\ChemStation\1\Data\SUN\SUN 2025-01-17 16-18-28\AY3-
                  10-20.M (Sequence Method)
Last changed    : 8/5/2025 8:27:09 pm by SYSTEM
                  (modified after loading)
Additional Info : Peak(s) manually integrated
```

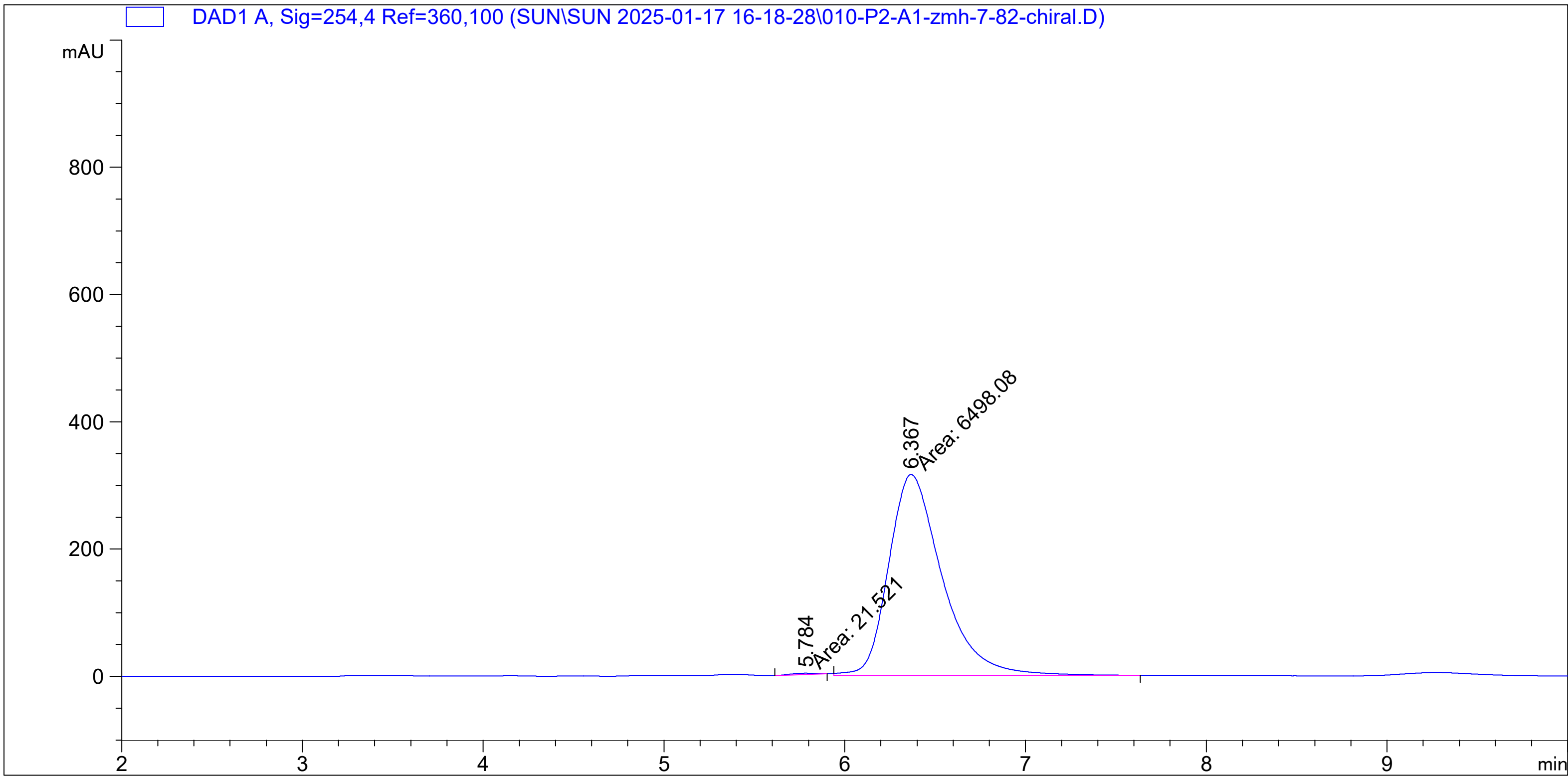

===

Area Percent Report

===

Sorted By : Signal
Multiplier : 1.0000
Dilution : 1.0000
Use Multiplier & Dilution Factor with ISTDs

Signal 1: DAD1 A, Sig=254,4 Ref=360,100

Peak #	RetTime [min]	Type	Width [min]	Area [mAU*s]	Height [mAU]	Area %
1	5.784	MM	0.1230	21.52099	2.10771	0.3301
2	6.367	FM	0.3428	6498.07715	315.96295	99.6699

Totals : 6519.59814 318.07067

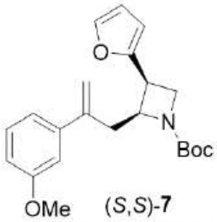

(*S*,*S*)-**7**
enantioenriched

===

Acq. Operator : SYSTEM Seq. Line : 26
Sample Operator : SYSTEM
Acq. Instrument : HPLC Location : P2-A-02
Injection Date : 17/1/2025 11:47:18 pm Inj : 1
Inj Volume : 2.000 µl
Different Inj Volume from Sample Entry! Actual Inj Volume : 10.000 µl
Acq. Method : C:\Users\Public\Documents\ChemStation\1\Data\SUN\SUN 2025-01-17 16-18-28\AD3-10-20.M
Last changed : 15/8/2022 10:21:32 pm by SYSTEM
Analysis Method : C:\Users\Public\Documents\ChemStation\1\Data\SUN\SUN 2025-01-17 16-18-28\AD3-10-20.M (Sequence Method)
Last changed : 8/5/2025 8:18:32 pm by SYSTEM
(modified after loading)
Additional Info : Peak(s) manually integrated

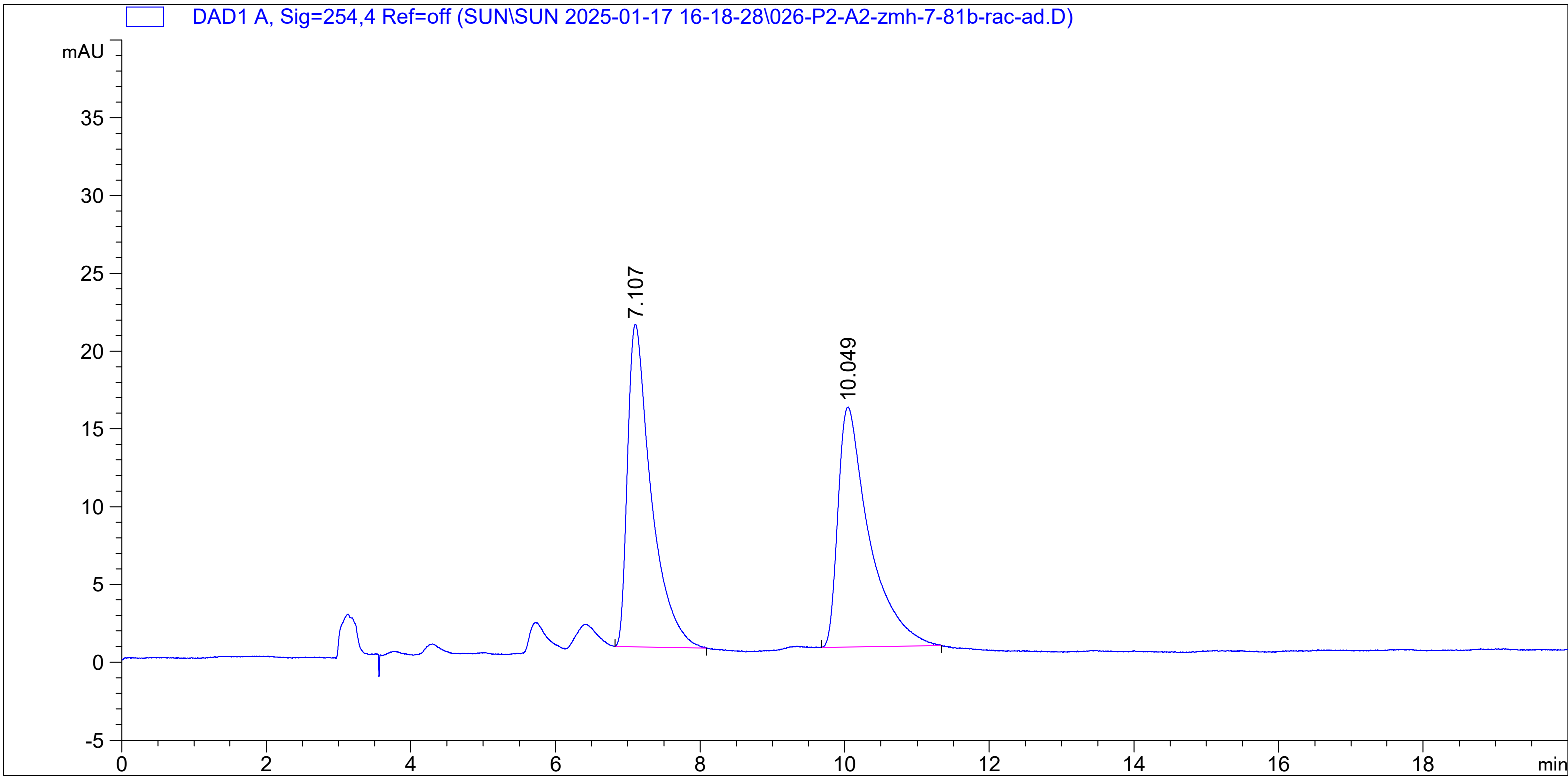

===

Area Percent Report

===

Sorted By : Signal
Multiplier : 1.0000
Dilution : 1.0000
Use Multiplier & Dilution Factor with ISTDs

Signal 1: DAD1 A, Sig=254,4 Ref=off

Peak #	RetTime [min]	Type	Width [min]	Area [mAU*s]	Height [mAU]	Area %
1	7.107	BB	0.2843	470.22406	20.75970	50.4337
2	10.049	BB	0.3526	462.13635	15.42267	49.5663

Totals : 932.36041 36.18237

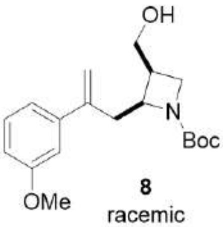

8
racemic

Data File C:\Users\P...ion\1\Data\SUN\SUN 2025-01-18 10-06-17\002-P1-A1-zmh-7-81b-chiral.D
Sample Name: zmh-7-81b-chiral

===

Acq. Operator : SYSTEM Seq. Line : 2
Sample Operator : SYSTEM
Acq. Instrument : HPLC Location : P1-A-01
Injection Date : 18/1/2025 10:19:29 am Inj : 1
Inj Volume : 2.000 µl
Different Inj Volume from Sample Entry! Actual Inj Volume : 10.000 µl
Acq. Method : C:\Users\Public\Documents\ChemStation\1\Data\SUN\SUN 2025-01-18 10-06-17\AD3-10-20.M
Last changed : 15/8/2022 10:21:32 pm by SYSTEM
Analysis Method : C:\Users\Public\Documents\ChemStation\1\Data\SUN\SUN 2025-01-18 10-06-17\AD3-10-20.M (Sequence Method)
Last changed : 8/5/2025 8:20:26 pm by SYSTEM
(modified after loading)
Additional Info : Peak(s) manually integrated

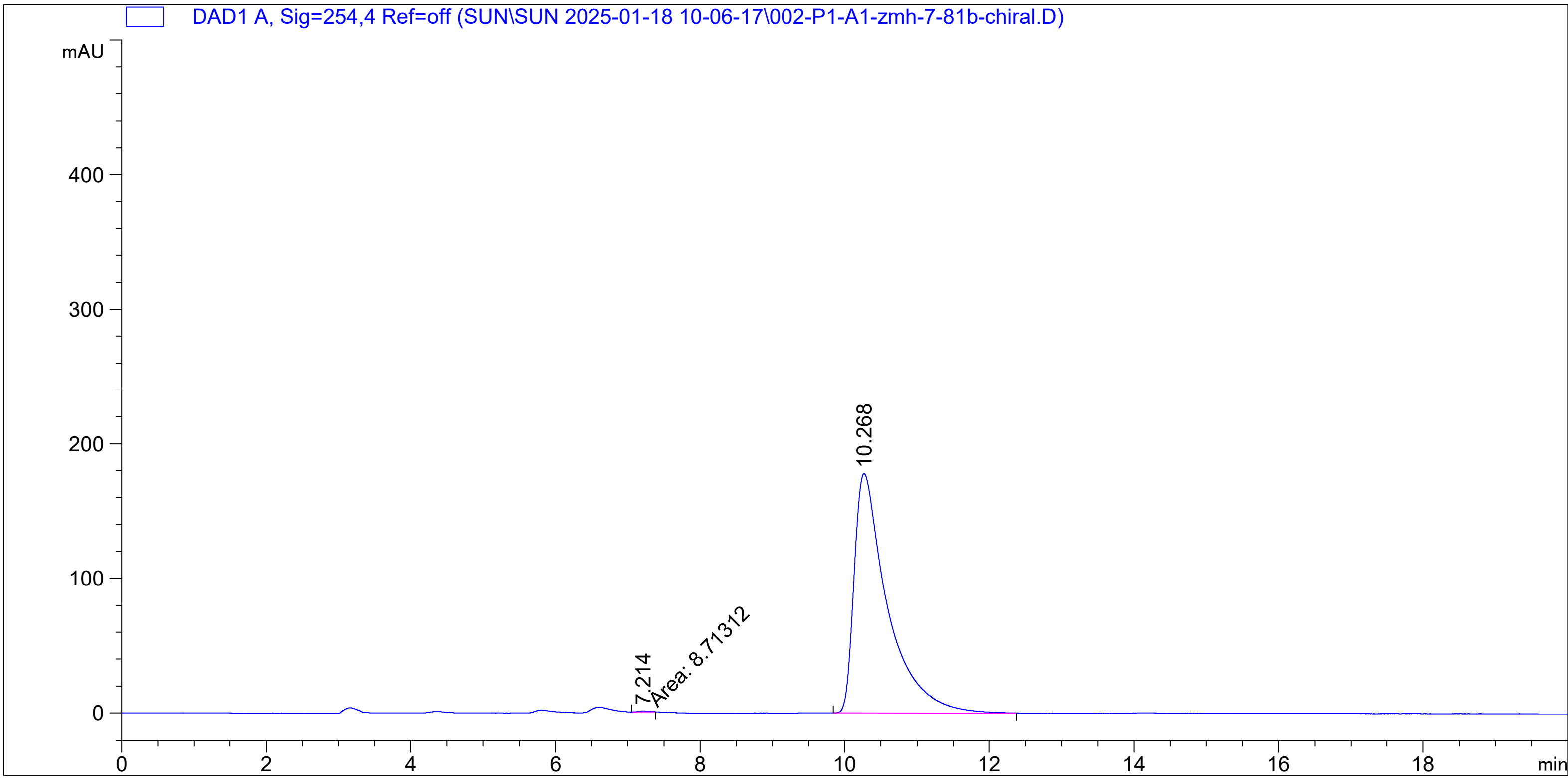

===

Area Percent Report

===

Sorted By : Signal
Multiplier : 1.0000
Dilution : 1.0000
Use Multiplier & Dilution Factor with ISTDs

Signal 1: DAD1 A, Sig=254,4 Ref=off

Peak #	RetTime [min]	Type	Width [min]	Area [mAU*s]	Height [mAU]	Area %
1	7.214	MM	0.1895	8.71312	7.66456e-1	0.1531
2	10.268	BB	0.4501	5682.69238	177.94878	99.8469

Totals : 5691.40550 178.71523

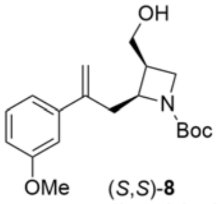

(*S*,*S*)-**8**
enantioenriched

Data File C:\Users\P...tation\1\Data\SUN\SUN 2025-01-20 20-59-37\029-P2-B1-zmh-br-rac-ij.D
Sample Name: zmh-br-rac-ij

===

Acq. Operator : SYSTEM   Seq. Line : 29
Sample Operator : SYSTEM
Acq. Instrument : HPLC   Location : P2-B-01
Injection Date : 21/1/2025 4:27:55 am   Inj : 1
                                          Inj Volume : 2.000 µl
Different Inj Volume from Sample Entry!   Actual Inj Volume : 10.000 µl
Acq. Method : C:\Users\Public\Documents\ChemStation\1\Data\SUN\SUN 2025-01-20 20-59-37\IJ3-10-30.M
Last changed : 29/8/2022 6:14:58 pm by SYSTEM
Analysis Method : C:\Users\Public\Documents\ChemStation\1\Data\SUN\SUN 2025-01-20 20-59-37\IJ3-10-30.M (Sequence Method)
Last changed : 8/5/2025 8:28:59 pm by SYSTEM
(modified after loading)
Additional Info : Peak(s) manually integrated

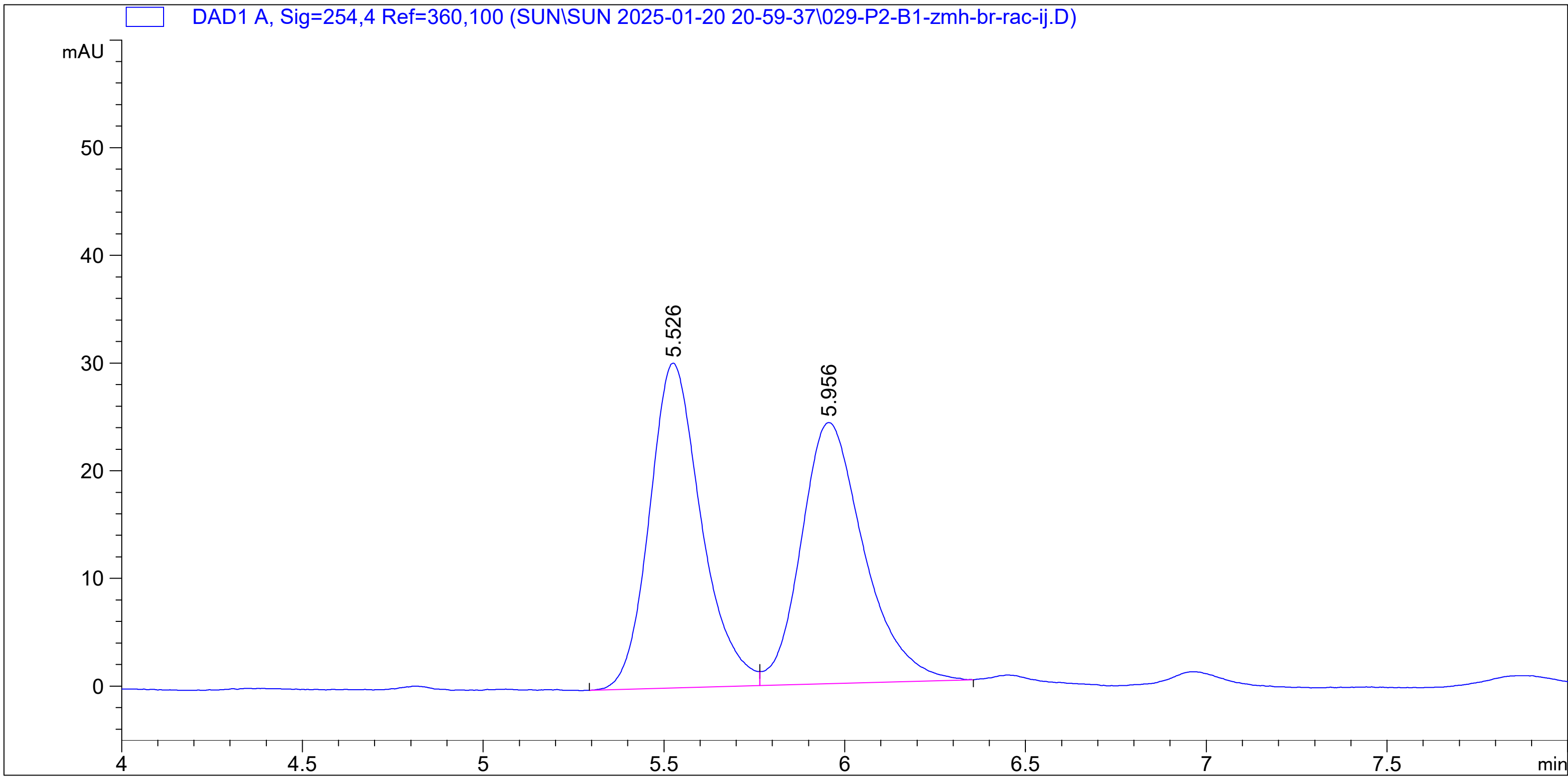

===

Area Percent Report

===

Sorted By : Signal
Multiplier : 1.0000
Dilution : 1.0000
Use Multiplier & Dilution Factor with ISTDs

Signal 1: DAD1 A, Sig=254,4 Ref=360,100

Peak #	RetTime [min]	Type	Width [min]	Area [mAU*s]	Height [mAU]	Area %
1	5.526	BV	0.1490	301.23511	30.16537	50.6479
2	5.956	VB	0.1807	293.52768	24.25106	49.3521

Totals : 594.76279 54.41644

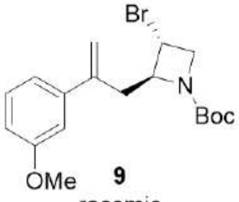

9
racemic

Data File C:\Users\P...tation\1\Data\SUN\SUN 2025-01-21 10-16-06\002-P1-A1-zmh-Br-chiral.D
Sample Name: zmh-Br-chiral

===

Acq. Operator   : SYSTEM   Seq. Line :   2
Sample Operator : SYSTEM
Acq. Instrument : HPLC   Location :   P1-A-01
Injection Date : 21/1/2025 10:29:21 am   Inj :   1
                                               Inj Volume : 2.000 µl
Different Inj Volume from Sample Entry! Actual Inj Volume : 10.000 µl
Acq. Method   : C:\Users\Public\Documents\ChemStation\1\Data\SUN\SUN 2025-01-21 10-16-06\IJ3-10-30.M
Last changed   : 29/8/2022 6:14:58 pm by SYSTEM
Analysis Method : C:\Users\Public\Documents\ChemStation\1\Data\SUN\SUN 2025-01-21 10-16-06\IJ3-10-30.M (Sequence Method)
Last changed   : 8/5/2025 8:31:09 pm by SYSTEM
                  (modified after loading)
Additional Info : Peak(s) manually integrated

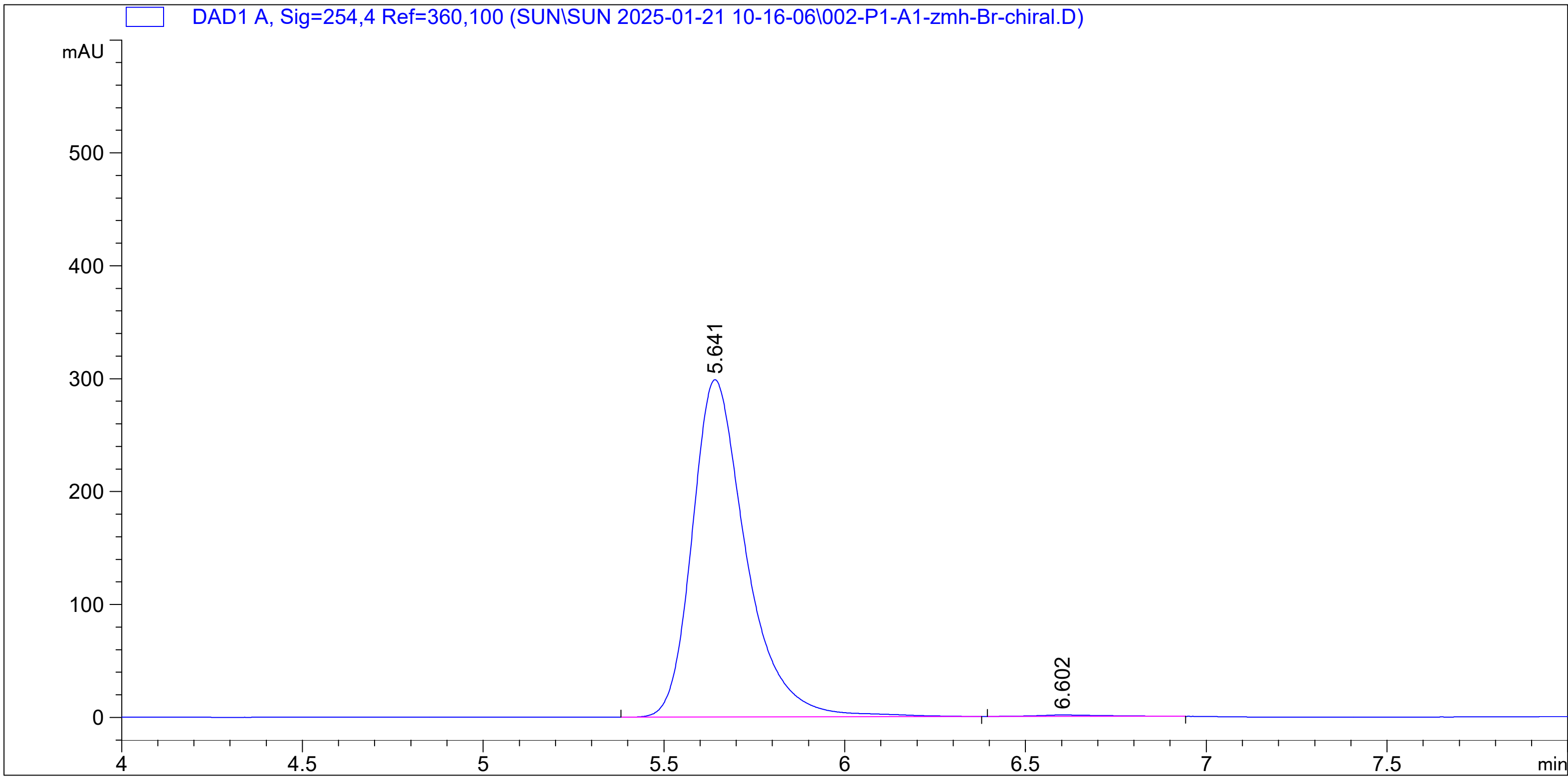

===

Area Percent Report

===

Sorted By   :   Signal
Multiplier   :   1.0000
Dilution   :   1.0000
Use Multiplier & Dilution Factor with ISTDs

Signal 1: DAD1 A, Sig=254,4 Ref=360,100

Peak #	RetTime [min]	Type	Width [min]	Area [mAU*s]	Height [mAU]	Area %
1	5.641	BB	0.1558	3106.13843	298.84854	99.4391
2	6.602	BB	0.1633	17.52086	1.27506	0.5609

Totals : 3123.65929 300.12360

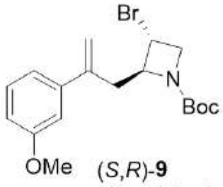

(*S*,*R*)-**9**
enantioenriched

===

Acq. Operator : SYSTEM Seq. Line : 14
Sample Operator : SYSTEM
Acq. Instrument : HPLC Location : P2-A-01
Injection Date : 7/6/2025 9:52:47 pm Inj : 1
Inj Volume : 2.000 µl
Different Inj Volume from Sample Entry! Actual Inj Volume : 20.000 µl
Acq. Method : C:\Users\Public\Documents\ChemStation\1\Data\SUN\SUN 2025-06-07 18-25-44\IC3-10-20.M
Last changed : 15/8/2022 10:26:28 pm by SYSTEM
Analysis Method : C:\Users\Public\Documents\ChemStation\1\Data\SUN\SUN 2025-06-07 18-25-44\IC3-10-20.M (Sequence Method)
Last changed : 8/6/2025 10:59:36 am by SYSTEM
(modified after loading)
Additional Info : Peak(s) manually integrated

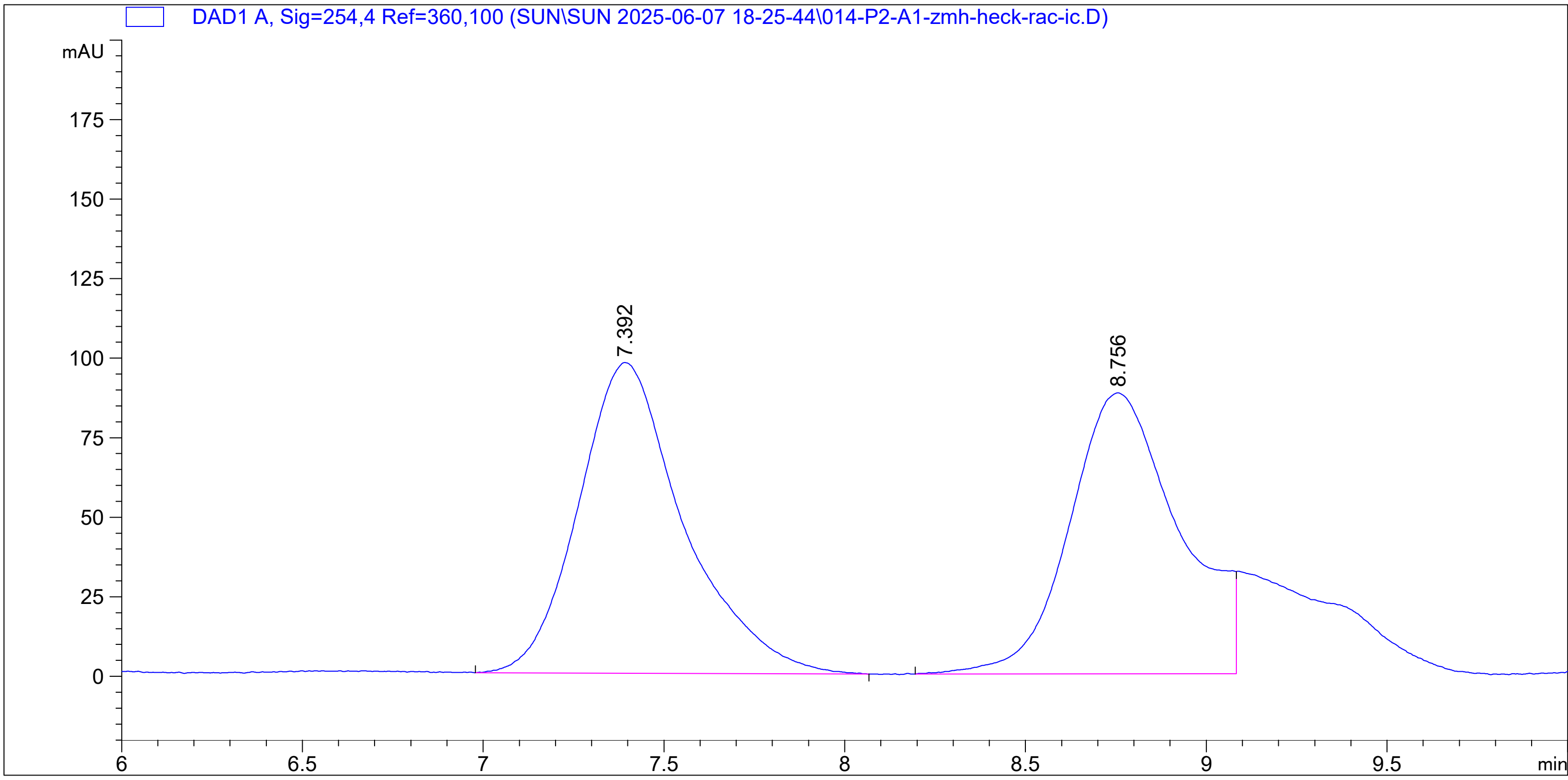

===

Area Percent Report

===

Sorted By : Signal
Multiplier : 1.0000
Dilution : 1.0000
Use Multiplier & Dilution Factor with ISTDs

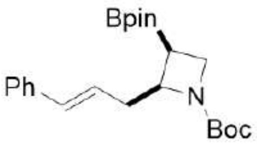

11
racemic

Signal 1: DAD1 A, Sig=254,4 Ref=360,100

Peak #	RetTime [min]	Type	Width [min]	Area [mAU*s]	Height [mAU]	Area %
1	7.392	BV R	0.2493	1972.70898	97.61768	51.4904
2	8.756	VV R	0.2520	1858.51160	88.32236	48.5096

Totals : 3831.22058 185.94004

===

Acq. Operator : SYSTEM   Seq. Line : 18
Sample Operator : SYSTEM
Acq. Instrument : HPLC   Location : P1-F-01
Injection Date : 7/6/2025 10:57:31 pm   Inj : 1
Inj Volume : 2.000 µl
Different Inj Volume from Sample Entry! Actual Inj Volume : 10.000 µl
Acq. Method : C:\Users\Public\Documents\ChemStation\1\Data\SUN\SUN 2025-06-07 18-25-44\IC3-10-20.M
Last changed : 15/8/2022 10:26:28 pm by SYSTEM
Analysis Method : C:\Users\Public\Documents\ChemStation\1\Data\SUN\SUN 2025-06-07 18-25-44\IC3-10-20.M (Sequence Method)
Last changed : 8/6/2025 11:01:27 am by SYSTEM
(modified after loading)
Additional Info : Peak(s) manually integrated

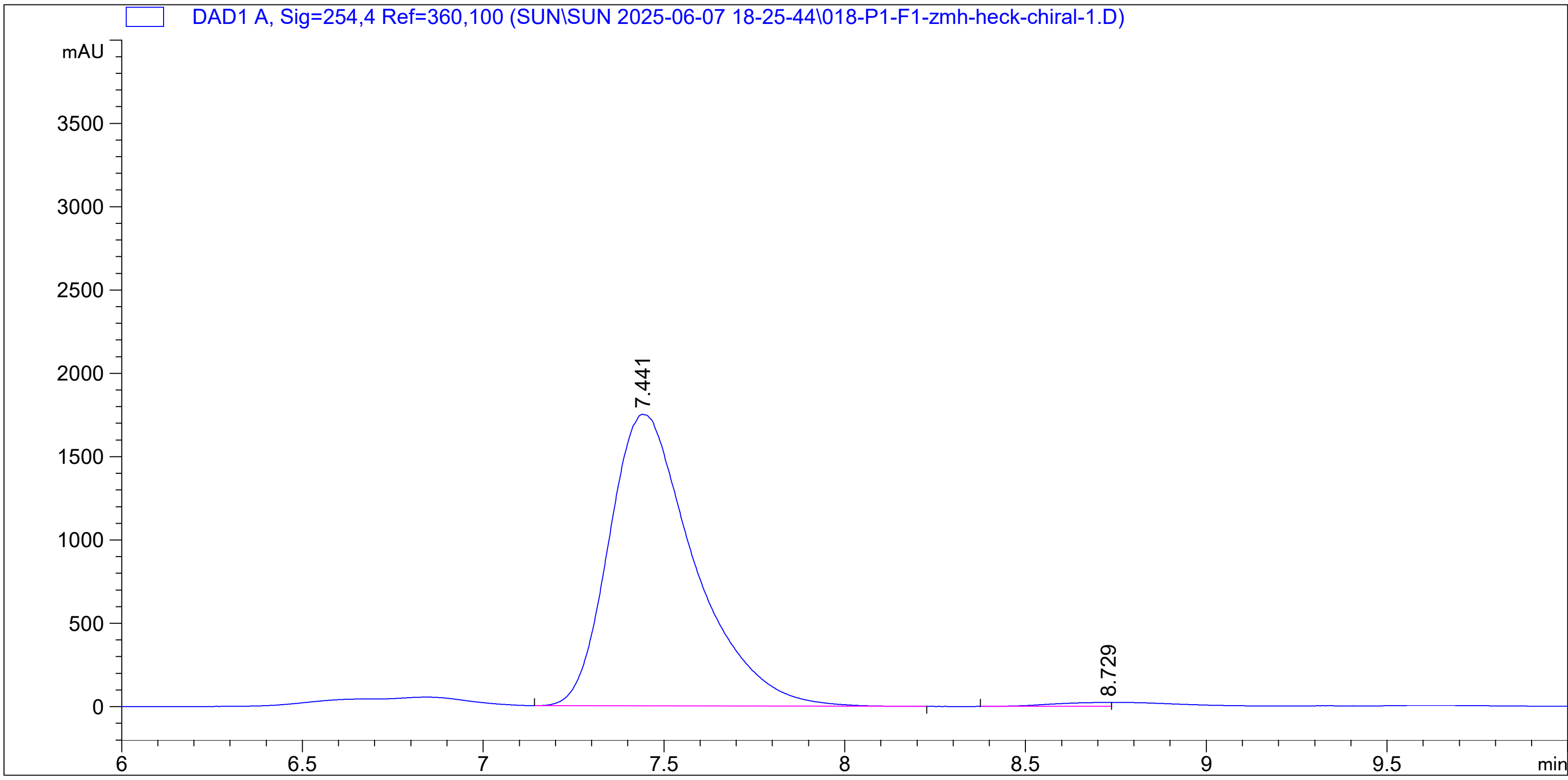

===

Area Percent Report

===

Sorted By : Signal
Multiplier : 1.0000
Dilution : 1.0000
Use Multiplier & Dilution Factor with ISTDs

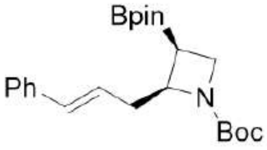

(*S*,*S*)-**11**
enantioenriched

Signal 1: DAD1 A, Sig=254,4 Ref=360,100

Peak #	RetTime [min]	Type	Width [min]	Area [mAU*s]	Height [mAU]	Area %
1	7.441	BV R	0.1976	2.87235e4	1749.17065	99.1516
2	8.729	BV	0.1307	245.78908	22.63249	0.8484

Totals : 2.89693e4 1771.80315
